# Plasticity in plastid redox networks: evolution of glutathione-dependent redox cascades and glutathionylation sites

**DOI:** 10.1186/s12870-021-03087-2

**Published:** 2021-07-05

**Authors:** Stefanie J. Müller-Schüssele, Finja Bohle, Jacopo Rossi, Paolo Trost, Andreas J. Meyer, Mirko Zaffagnini

**Affiliations:** 1 Institute of Crop Science and Resource Conservation (INRES), University of Bonn, Friedrich-Ebert-Allee 144, 53113 Bonn, Germany; 2 Present Address: Department of Biology, Technische Universität Kaiserslautern, 67663 Kaiserslautern, Germany; 3 Department of Pharmacy and Biotechnology, University of Bologna, 40126 Bologna, Italy

**Keywords:** Protein *S*-glutathionylation, Redox regulation, Land plant evolution, Plastid, Cysteine, Glutathione, Glutaredoxin

## Abstract

**Background:**

Flexibility of plant metabolism is supported by redox regulation of enzymes via posttranslational modification of cysteine residues, especially in plastids. Here, the redox states of cysteine residues are partly coupled to the thioredoxin system and partly to the glutathione pool for reduction. Moreover, several plastid enzymes involved in reactive oxygen species (ROS) scavenging and damage repair draw electrons from glutathione. In addition, cysteine residues can be post-translationally modified by forming a mixed disulfide with glutathione (*S*-glutathionylation), which protects thiol groups from further oxidation and can influence protein activity. However, the evolution of the plastid glutathione-dependent redox network in land plants and the conservation of cysteine residues undergoing *S*-glutathionylation is largely unclear.

**Results:**

We analysed the genomes of nine representative model species from streptophyte algae to angiosperms and found that the antioxidant enzymes and redox proteins belonging to the plastid glutathione-dependent redox network are largely conserved, except for lambda- and the closely related iota-glutathione *S*-transferases. Focussing on glutathione-dependent redox modifications, we screened the literature for target thiols of *S*-glutathionylation, and found that 151 plastid proteins have been identified as glutathionylation targets, while the exact cysteine residue is only known for 17% (26 proteins), with one or multiple sites per protein, resulting in 37 known *S*-glutathionylation sites for plastids. However, 38% (14) of the known sites were completely conserved in model species from green algae to flowering plants, with 22% (8) on non-catalytic cysteines. Variable conservation of the remaining sites indicates independent gains and losses of cysteines at the same position during land plant evolution.

**Conclusions:**

We conclude that the glutathione-dependent redox network in plastids is highly conserved in streptophytes with some variability in scavenging and damage repair enzymes. Our analysis of cysteine conservation suggests that *S*-glutathionylation in plastids plays an important and yet under-investigated role in redox regulation and stress response.

**Supplementary Information:**

The online version contains supplementary material available at 10.1186/s12870-021-03087-2.

## Background

### Importance of redox regulation in photosynthetic eukaryotes

Plant evolution has its roots in two major endosymbiotic events, the first leading to the formation of the first eukaryotic cell and the second generating photosynthetic eukaryotes [1]. In the green lineage (Chloroplastida), land plants evolved from streptophyte algae [2]. In chloroplasts, the photosynthetic electron transport (PET) chain allows the generation of reducing equivalents (e.g. NADPH) and ATP that fuel sugar biosynthesis by the Calvin-Benson cycle (CBC). In order to balance the photosynthetic process, a tight regulation of electron flux is required to harmonise light capture with metabolic activities, as well as to prevent extensive energy or electron transfer to oxygen, generating reactive oxygen species (ROS) that can damage DNA, lipids or proteins [3, 4]. Nascent ROS and damaged molecules are addressed by high capacity scavenging and repair systems that draw electrons from either NADPH or PET coupled to stromal redox proteins [5–8].

In order to balance energy demand for metabolic processes, plant cells have a highly flexible metabolism that quickly switches between photosynthetic and non-photosynthetic modes. Moreover, cell compartments can contain metabolic enzymes with conditional or opposing activities, and regulation in time and space may avoid futile cycling of metabolites [9]. These complex regulatory processes are partly mediated by changes in the redox status of cysteine thiol groups in proteins enabling reversible modulation of protein function and structure that is influenced by the intracellular redox state.

### Electron flux in redox cascades

Cysteine thiols influence protein structure and activity as their redox state may have an impact on protein folding and oligomerization, reactivity towards ROS or on substrate binding to active sites of redox-active enzymes [6, 9]. In this regard, dithiol/disulfide exchange reactions play a pivotal role in protein regulation. This redox modification allows the reversible transfer of two electrons, depending on the interaction of redox-sensitive proteins and according to the midpoint potential of involved thiol-switches. Many enzymes involved in scavenging of ROS and lipid peroxides or protein repair contain active site or regulatory cysteines that are coupled to redox cascades. However, the reactivity of the thiol group (especially the thiolate anion, −S^−^) can also favour reactions with oxidant molecules such as hydrogen peroxide (H_2_O_2_) or the free radical nitric oxide (•NO) and NO-derived compounds (also referred to as reactive nitrogen species, RNS). This results in the oxidation of the thiol moiety by reversible (*S*-sulfenylation, *S*-sulfhydration, *S*-nitrosylation or *S*-glutathionylation) or even irreversible modifications (*S*-sulfonylation), that can modulate or block protein function [3].

The redox network in plants comprises several specific reductases that transfer electrons to either the cysteine-containing tripeptide glutathione (GSH) or thioredoxins (TRX). In plastids, glutathione reductase (GR) safeguards a highly reduced glutathione pool relying on NADPH as electron source, whereas ferredoxin TRX reductase (FTR) draws electrons from photosynthetic light reactions to reduce TRX. Plastids also contain a special type of TRX, namely NTRC that can be reduced by NADPH through an NTR domain fused to the TRX module [10, 11]. Thiol switches downstream of TRXs are relatively well characterised with direct evidence for 36 TRX-regulated proteins in plastids [5] whereas proteomics studies suggested a number of approximately 100 putative TRX-regulated proteins in plastids [12, 13]. In chloroplasts, redox control via TRX-dependent dithiol/disulfide exchanges allows enzyme activation under light conditions via the reduction of regulatory protein disulfides on target proteins such as CBC enzymes [6, 14]. TRXs can also transfer electrons to peroxide scavenging enzymes such as 2-Cys peroxiredoxins (PRX) [15] and glutathione peroxidase-like proteins (GPXLs) [16]. Thus, plastidial 2-Cys PRX can serve as a link between H_2_O_2_ scavenging and the oxidation of thiol switches on several target proteins such as *m*- and *f*-type TRXs [17–19] and glucose-6-phosphate dehydrogenase [20]. This redox interaction supports deactivation of CBC enzymes and activation of the first step of the oxidative pentose phosphate pathway, respectively, facilitating the tight regulation of plastid carbon metabolism in light to dark transitions to avoid futile cycling.

The glutathione pool in cell compartments containing a GR is highly reduced (≥50,000:1 GSH:GSSG), leaving only nM concentrations of glutathione disulfide (GSSG). This results in a highly negative glutathione redox potential (*E*_GSH_) ranging from − 310 to − 360 mV [21–23]. Glutathione acts as reductant during the detoxification of potentially toxic organic electrophiles, as well as during ascorbate regeneration via the ascorbate-glutathione-cycle [24]. In addition, some enzymes involved in ROS scavenging and damage repair, such as single cysteine (atypical) methionine sulfoxide reductase B (AtMSRB1), are fully dependent on GSH for reduction [25], whereas others, such as type II peroxiredoxin E (PRXIIE), can be regenerated via GSH/glutaredoxins (GRXs) with a higher efficiency than via TRX [15, 26]. The role of GSH in MSRB1 and PRXIIE activities relies on its nucleophilic attack on sulfenylated active site cysteines formed during the catalytic cycle. This spontaneous reaction leads to the formation of a mixed disulfide (i.e. *S*-glutathionylation) that is specifically controlled by GRXs, enzymes belonging to the TRX superfamily [27]. GRXs catalyse the removal of the glutathione moiety (i.e. deglutathionylation) allowing the regeneration to reduced and active enzymes in a mechanism involving GSH as electron donor. Thus, there is an interrelation with yet unknown biological significance between the local ROS levels, the local detoxification systems, the glutathione redox potential and protein *S*-glutathionylation.

Cross-talk between TRX and the GSH/GRX system has been evidenced in *Arabidopsis thaliana* mutants lacking the cytosolic GR1 or GR2 in mitochondria, where the TRX system can serve as a back-up system for local control of GSSG accumulation in the respective compartment [28, 29]. Vice versa, TRX redox state can be linked to the GSH/GRX system, as cytosolic TRX redox status can be rescued via the glutathione system [30], and as some TRXs such as *f*-type TRXs are *S*-glutathionylation targets [31]. However, studies in *A. thaliana* and *Physcomitrium* (formerly *Physcomitrella* [32]) *patens* have evidenced that the TRX system cannot compensate for a disturbed GSH/GRX system in plastids [29, 33], raising the question of redox cascade organisation and cross-talk in the stroma.

### Evolution shapes redox regulation

In photosynthesis, light-dependent regulation of several enzymes of the CBC has evolved to coordinate active light reactions with carbon fixation in the metabolic phase. Some redox-responsive thiol-switches relying on dithiol/disulfide interchanges were already established in the cyanobacterial cell that became the endosymbiont, whereas others emerged during algal evolution [9, 34, 35]. The set of redox-regulated proteins acquired via endosymbiosis was expanded by gain of cysteines via amino acid changes or the insertion of entire cysteine-containing flexible regulatory loops [9, 34]. During plant evolution, streptophyte algae were able to colonise freshwater habitats and surrounding land, as they exhibited a set of advantageous morphological features such as apical cell growth, biplastidy and branching [2]. Early land plants possessed simple body plans but were facing a habitat with rapidly fluctuating environmental conditions such as flooding, dehydration, rapid temperature changes and high light exposure [36]. This lifestyle is mirrored in extant non-vascular land plant lineages such as mosses that are tolerant to extended submergence and contain several protection mechanisms against light stress [37, 38]. Light and temperature fluctuations as well as photoinhibitory conditions are especially challenging for metabolic regulation and ROS scavenging in chloroplasts due to large changes in electron transport rate, as well as the necessary fast adaptations in enzymatic activities to sustain growth and promote survival.

However, the evolutionary adaptations that occurred in redox cascades during land plant evolution are largely unknown. To date, genomes of streptophyte algae (*Chara braunii*), hornworts (*Anthoceros agrestis* and *Anthoceros punctatus*) and ferns (*Salvinia cucullata, Azolla filiculoides*) have become available [39–42], complementing the existing genomic information from liverworts (*Marchantia polymorpha*), mosses (*P. patens*) and lycophytes (*Selaginella moellendorffii*) model species. This unprecedented coverage of land plant lineages and their algal sister group enables the comparative investigation of redox cascade evolution in the green lineage on several levels, namely regarding the distinct reductases, redox transmitters, as well as target thiol-switches on metabolic enzymes. Based on the importance of glutathione as redox buffer and source of reducing equivalents for antioxidant enzymes, this study investigates the components of glutathione-related redox networks in plastids of streptophyte model species.

Notably, during plant evolution, several redox-relevant protein families, such as the TRX superfamily comprising both TRXs and GRXs [9, 35], have largely expanded supporting functional diversification. Moreover, in seed plants, ROS/RNS are tightly linked to biotic defence responses, e.g. via the NADPH oxidase-mediated ROS bursts at the plasma membrane or via the salicylic acid signalling pathway involving NPR1 [43]. In contrast, the organisation and significance of redox networks for metabolic regulation, ROS scavenging and development in non-seed plants are only starting to emerge [33, 44]. However, understanding the evolution of redox networks may help to reveal ancestral mechanisms in stress resilience. Studies in model organisms allowing reverse genetics show that phenotypes of mutants with defects in plastid glutathione homeostasis are diverse, suggesting modification of redox networks during plant evolution. Thus, the absence of plastid GR is embryo-lethal in *A. thaliana*, whereas *P. patens* can partially compensate this loss but becomes light-sensitive [29, 33].

In order to assess the evolution of both, the effectors and putative target cysteines of glutathione-related redox networks in plastids, we additionally investigate protein *S*-glutathionylation by creating an updated list of known target cysteines of *S*-glutathionylation and their evolutionary conservation.

## Results

### Phylogenetic analysis of glutathione-dependent redox-relevant plastid proteins from streptophyte algae to land plants

On the one hand, many protein families of redox cascade components have expanded during land plant evolution and underwent functional diversification [9]. On the other hand, several proteins are persistently conserved as only one isoform in a compartment, suggesting a potential essential biological function and tight regulation of gene copy number. In order to identify plastidial glutathione-related redox components with interesting phylogenetic patterns, we first did an explorative phylogenetic analysis taking advantage of the available sequence coverage of streptophyte algae and non-seed plant lineages. We included all known scavenging and damage repair enzyme families with members that were reported to (1) localise to plastids and (2) use GSH and produce GSSG: dehydroascorbate reductase (DHAR), glutathione *S*-transferases lambda and iota (GSTL, GSTI), atypical methionine sulfoxide reductase B1 (MSRB1), peroxiredoxin IIE (PRXIIE), glutaredoxins (GRXs), as well as glutathione reductase (GR), which is responsible for reduction of GSSG. All used protein sequences and gene accessions are listed in Table S1 (Additional file 1).

#### Dehydroascorbate reductases

**Fig. 1:**
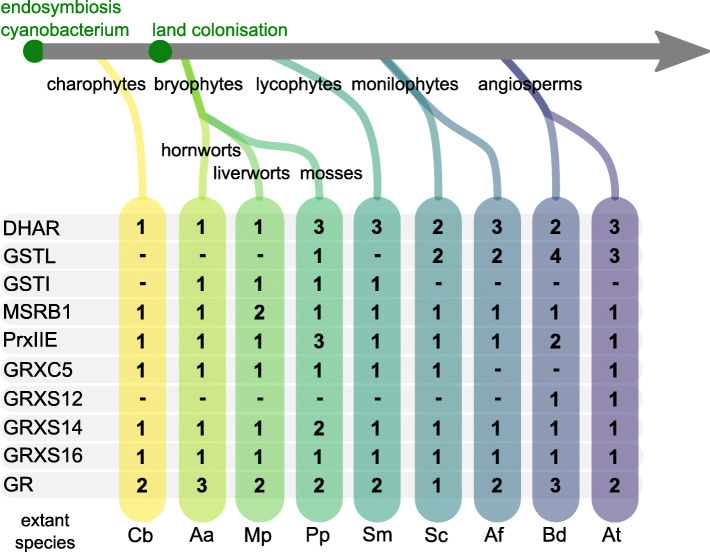
Evolution of protein families related to plastid GSH-dependent redox balance. Numbers indicate the number of isoforms present in the genome. DHAR, dehydroascorbate reductase; GST, glutathione *S*-transferase, L: Lambda, I: Iota; MSRB1, methionine sulfoxide reductase B1 (1 Cys); PRX, peroxiredoxin; GRX, glutatredoxin; GR, glutathione reductase. Model species names are abbreviated: Cb = *Chara braunii*; Aa = *Anthoceros agrestis*; Mp = *Marchantia polymorpha*; Pp = *Physcomitrium patens*; Sm = *Selaginella moellendorffii*; Sc = *Salvinia cucullata*; Af = *Azolla filiculoides*; Bd = *Brachypodium distachyon*; At = *Arabidopsis thaliana*

**Fig. 2:**
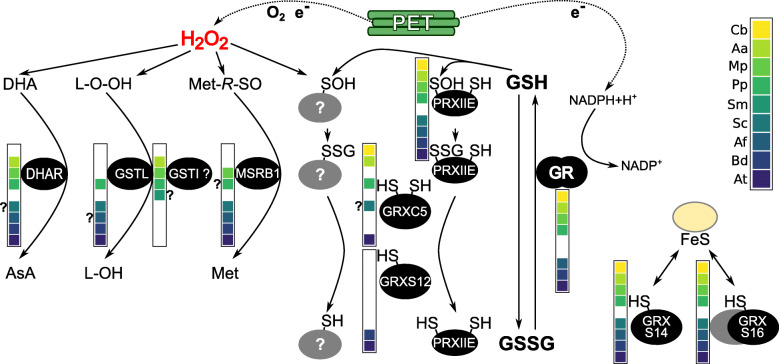
Overview of evolution of plastid glutathione-related redox networks in land plants and streptophyte algae. Schematic overview of the plastid GSH-dependent redox network in land plant model species and the streptophyte alga *C. braunii*. Electrons from photosynthetic electron transport (PET) contribute to ROS generation and at the same time to ROS scavenging, damage repair and redox homeostasis. H_2_O_2_ leads to lipid peroxidation (L-O-O-H) as well as oxidation of protein methionine (Met-*R*-SO), of ascorbic acid (AsA) to dehydroascorbate (DHA) or protein thiol oxidation to the respective sulfenic acid (RS-OH) that can react with GSH to form an *S*-glutathionylated adduct (RS-SG). Glutaredoxins (GRX) can (de) glutathionylate proteins. The balance between the reduced tripeptide glutathione (GSH) and glutathione disulfide (GSSG) is influenced by GSSG generation via enzymes involved in ROS/RNS scavenging or protein as well as lipid repair, such as dehydroascorbate reductase (DHAR), atypical (1 Cys) methionine sulfoxide reductases B1 (MSRB1), lambda and iota-type (?, function not confirmed in vitro) glutathione *S*-transferases and type II peroxiredoxins (PRX). Glutathione reductase (GR, NADPH-dependent) safeguards a highly reduced GSH-pool. The presence of at least one plastid-targeted isoform of a protein in a model species (assessed by presence of an N-terminal extension and targeting predictions, see Additional files 1, 2, 3, 4, 5, 6, 7, 8) is represented by a coloured box in the species legend next to the protein. A coloured box with question mark means the potential presence of an isoform as targeting prediction is unclear, but N-terminal extension indicating a targeting peptide is present (see Additional files 1, 2, 3, 4, 5, 6, 7, 8). Absence of a box can either mean absence of homologs from that species (see Fig. 1), or that all homologs do not have N-terminal extensions or that gene models are fragmentary (see Additional files 1, 2, 3, 4, 5, 6, 7, 8). Species legend: *Chara braunii* (Cb), *Anthoceros agrestis* (Aa), *Marchantia polymorpha* (Mp), *Physcomitrium patens* (Pp)*, Selaginella moellendorffii* (Sm), *Salvinia cucullata* (Sc), *Azolla filiculoides* (Af), *Brachypodium distachyon* (Bd) and *Arabidopsis thaliana* (At)

DHARs belong to the GST superfamily and catalyse the reduction of dehydroascorbate to ascorbate using GSH as electron donor. By maintaining a reduced ascorbate pool, DHARs indirectly assist in the detoxification of H_2_O_2_ catalysed by ascorbate peroxidases (APXs). The phylogenetic tree of plant DHARs (Additional file 2) shows several independent gene duplications resulting in a number of one to three paralogs per species. Targeting predictions (TargetP [45, 46], LOCALIZER [47] and PredAlgo [48], Additional file 1 Table S1) as well as the presence or absence of N-terminal sequence extensions suggest a high variability in subcellular targeting. Notably, only a single DHAR gene is present in *C. braunii* and two bryophyte species (*M. polymorpha*, *A. agrestis*) (Fig. 1, Additional file 2). Whereas the *C. braunii* ortholog is putatively cytosolic, both *M. polymorpha* and *A. agrestis* orthologs possess an N-terminal extension and are predicted to be targeted to plastids. This indicates that a stromal DHAR occurred early in land plant evolution. DHAR1 from *P. patens* (Pp3c22_5470V3) [49] is predicted to be targeted to plastids but proteomic evidence also indicates its presence in mitochondria [49], which may suggest dual-targeting to plastids and mitochondria. All land plant model species except *S. moellendorffii* have at least one DHAR isoform with N-terminal extension and/or a plastid targeting prediction (Fig. 2 and Additional file 2). In *S. moellendorffii*, the situation remains unclear, as at least one gene model is incomplete at the N-terminus.

#### Iota (I)- and lambda (L)-type glutathione *S*-transferases

In addition to DHARs, plastid-targeting has also been reported within the lambda-subclass of GSTs (GSTL) [50]. While canonical GSTs conjugate GSH with electrophilic compounds, the subclasses theta, phi and tau have also been shown to act as GSH-dependent peroxidases that release GSSG [50, 51]. Because a similar reductive activity towards DHA or hydroperoxides has also been reported for plastid-localised PpGSTL1, we investigated isoforms of this subclass [50]. We did not identify any GSTL homologs in *C. braunii*, *A. agrestis*, *M. polymorpha* and *S. moellendorffii* but several homologs with variable N-terminal extensions and predicted targeting to plastids are present in the seed plant models (Additional file 3). On the contrary, the closely related subfamily of iota-type GSTs (GSTI) contains homologs from *A. agrestis*, *M. polymorpha* and *S. moellendorffii*, but is absent in the fern and seed plant models (Fig. 1). The function of GSTIs is yet unclear [50] while our analysis indicates that they may also be targeted to plastids where they might fulfil similar functions to their sister clade GSTL (Additional file 3, Fig. 2). We did not identify a *C. braunii* GSTI, but a *C. reinhardtii* GSTI has been reported [50], suggesting that the GSTI subfamily is present in chlorophytes.

#### Atypical methionine sulfoxide reductase B

Methionine sulfoxide reductases (MSR) catalyse the reduction of methionine sulfoxide (MetSO), repairing ROS-induced damage to proteins (Fig. 2). Oxidation of methionine can generate two diastereomeres that are reduced by two different non-related enzyme families, namely MSRA (reducing methionine-*S*-sulfoxide) and MSRB (reducing methionine-*R*-sulfoxide) [52]. MSRA and MSRB proteins containing two cysteines are dependent on TRX for regeneration, while atypical MSRBs such as plastid-targeted AtMSRB1 (AT1G53670), depend on GSH/GRX for regeneration [53]. In AtMSRB1, the second cysteine is replaced by a threonine (Thr132). Investigating AtMSRB1 homologs (Additional file 4a, c, d), we find a complete conservation of the isoform containing one cysteine as the well-supported MSRB1(1Cys) clade contains *C. braunii*, bryophyte, lycophyte and fern isoforms. The conservation of plastid targeting is unclear but likely variable, based on TargetP [45, 46], LOCALIZER (L) [47] and PredAlgo (P) [48] predictions (Additional file 1 Table S1) and the presence and absence of N-terminal extensions (Additional file 4b, Fig. 2). Notably, *M. polymorpha* does not possess a MSRB2 homolog while the MSRB1 gene was duplicated and the emerging two isoforms show differing N-terminal extensions and targeting predictions (Additional file 4).

#### Peroxiredoxin II E

PRXs are antioxidant enzymes that catalyze the reduction of hydroperoxides into alcohols using a strictly conserved cysteine (i.e. peroxidatic cysteine). Plastids contain canonical 2-Cys PRXs having two active site cysteines (peroxidatic and resolving cysteine), and an atypical 2-Cys PRX named PRXIIE (reviewed in [15]). Whereas the regeneration of canonical 2-Cys PRX is controlled by the plastidial TRX system via a dithiol/disulfide exchange, the recycling of oxidised poplar PRXIIE was demonstrated to be more efficiently catalysed by GRXS12 in vitro [26]. GRXS12 can restore the reduced PRXIIE as the peroxidatic cysteine undergoes glutathionylation during the catalytic cycle, highlighting the dependence of this PRXII subfamily on the GSH/GRX system for functional recycling (Fig. 2).

PRXIIE homologs are evolutionary conserved [54] and our analysis confirms a single highly supported clade of PRXIIE homologs from charophytes to flowering plants (Additional file 5). Most organisms investigated possess a single PRXIIE isoform and all complete protein models confirm an N-terminal extension that is largely predicted to confer targeting to plastids (Fig. 2, Additional file 1 Table S1, Additional file 5). The *S. moellendorffii* homolog did not allow a targeting analysis as the N-terminus of the protein model was fragmentary. We found gene duplications in *B. distachyon* with two isoforms and *P. patens* with three isoforms, all with conserved plastid targeting. Based on the phylogenetic analysis (Additional file 5), plastid GRX-dependent PRXIIE homologs are likely present in all analysed organisms.

#### Glutaredoxins

GRXs are oxidoreductases belonging to the TRX family, which is largely involved in the control of protein redox state. In photosynthetic eukaryotes, the GRX family comprises four classes that are localised in various subcellular compartments and distinguished by their active site signature and domain organization [3]. The nomenclature of the members of these GRX classes is based on the presence of a cysteine or a serine at the last position of the active site signature (CXXC/S) with a limited number of exceptions containing a residue differing from cysteine or serine [3]. In the model plant *A. thaliana*, GRXs are represented by six class I, four class II, and two class IV isoforms, while class III GRXs (also referred to as CC-type GRX or ROXY proteins) have largely expanded and comprise 21 members with multiple functions. One example of class III GRX function is the interaction with bZIP TGA transcription factors, influencing plant development and flowering [55, 56]. Plastids typically contain GRX members belonging to class I and II, namely class I GRXS12 and the close paralog GRXC5 (in *A. thaliana*) and class II GRXS14 and GRXS16 [57–59].

#### Class I GRX

GRXC5 and GRXS12 contain a single GRX domain with an active site signature (YCPYC and WCSYS, respectively) that differs slightly from the typical YC [P/S/G][Y/F] C motif of class I GRXs. Both GRX isoforms are redox-active being involved in the control of protein glutathionylation of plastidial proteins and in the recycling of antioxidant enzymes such as MSRB1 and PRXIIE (see above). The analysis of the phylogenetic tree for the redox-active class I GRXs revealed several evolutionary ancient clades corresponding to isoforms targeted to plastids (C5/S12 clade), the cytosol (C1/C2 clade), and the secretory pathway (C3/C4 clade) (Additional file 6). We found that only in the two angiosperm model species investigated, the second cysteine of the active site was replaced by a serine, giving rise to the GRXS12 isoform. This substitution can increase protein activity because formation of an internal disulfide that would block the active site can be avoided [58, 60]. The relevance of this mutation is emphasized by the fact that GRXS12 homologs became predominant in several angiosperms [61]. While the GRXC5 gene models from *S. moellendorffii* and *M. polymorpha* may be fragmentary and thus not contain the N-terminal targeting sequence, we did not identify any C5/S12 isoform clustering with the highly conserved GRXC5/S12 clade in the fern *A. filiculoides* (Additional file 6, Fig. 2).

#### Class II GRX

Class II GRXS14 and S16 contain a CGFS active site motif that is typically conserved in all other class II GRX members. As observed for plastidial class I GRXs, GRXS14 is formed by a single GRX domain whereas GRXS16 has a modular organization possessing an N-terminal domain (GIY–YIG endonuclease fold) fused to one GRX domain [3, 62, 63]. Class II GRXs are mainly thought to be involved in the coordination and transfer of iron-sulfur clusters [64, 65] (Fig. 2), but may also become redox-active after loss of iron-sulfur coordination in response to an oxidative signal [66]. Our phylogenetic analysis showed that GRXS14 and GRXS16 homologs are conserved in all investigated model species, with only one duplicated isoform of GRXS14 in *P. patens* (Additional file 7). As far as complete gene models are available, N-terminal extensions and predicted plastid targeting confirm the very high conservation of a single plastid-targeted isoform in these class II GRX subfamilies (Additional file 1 Table S1, Additional file 7, Fig. 2).

#### Glutathione reductase

GRs perform the highly efficient NADPH-dependent recovery of GSH from enzymatically or non-enzymatically generated GSSG (Fig. 2), keeping GSSG as low as nanomolar amounts [21]. The resulting highly negative *E*_GSH_ in the plant cytosol, peroxisomes, plastids and mitochondria is based on the activity of two isoforms exhibiting dual targeting to either cytosol and peroxisomes (e.g. AtGR1, AT3G24170) or plastids and mitochondria (e.g. AtGR2, AT3G54660) [28, 29]. These two isoforms were already shown to be evolutionary conserved, including the dual-targeting of one isoform to plastids and mitochondria in *P. patens* [67]. We inferred a phylogeny using our set of model species and found the same conservation of two clades, representing the two GR isoforms, suggesting that these isoforms were established before the emergence of land plants (Additional file 8, Fig. 2). The *S. moellendorffii* gene model for the mitochondria/plastid GR clade was potentially fragmentary at the N-terminus, not allowing for a targeting prediction. We did not identify an isoform in the mitochondria/plastid GR clade for *S. cucullata* nor for *A. agrestis* (Bonn). A BLASTN search revealed a possible locus for *S. cucullata* on scaffold s0092, however without a gene model present. The presence of a plastidial/mitochondrial isoform in *A. agrestis* (Oxford) suggests that the respective gene is present but not correctly predicted for *A. agrestis* (Bonn). Except for *B. distachyon*, that possesses two plastidial/mitochondrial GR isoforms, each investigated species has a single isoform in each clade. Notably, one *A. agrestis* isoform (Sc2ySwM_228.5258.1) did show higher sequence similarity to bacterial than plant GRs. We identified similar isoforms in the other sequenced Anthoceros species and strains, namely *A. agrestis* (Oxford) and *A. punctatus* [40] (Additional file 8), suggesting a horizontal gene transfer (HGT) that occurred before the split of these species. Notably, these isoforms possess N-terminal extensions compared to bacterial sequences (Additional file 8b).

### Evolutionary conservation of putative glutathionylation sites on plastid proteins

Many target thiol switches on cyanobacterial or plastidial proteins were acquired early in evolution, while others were reported to have evolved regulatory cysteines later. For example, the C-terminal extension in the plastidial glyceraldehyde-3-phosphate dehydrogenase isoform B (GAPB) evolved in streptophytes and the N-terminal cysteine pair of the plastidial NADPH-dependent malate dehydrogenase in land plants [9, 14, 68].

**Fig. 3:**
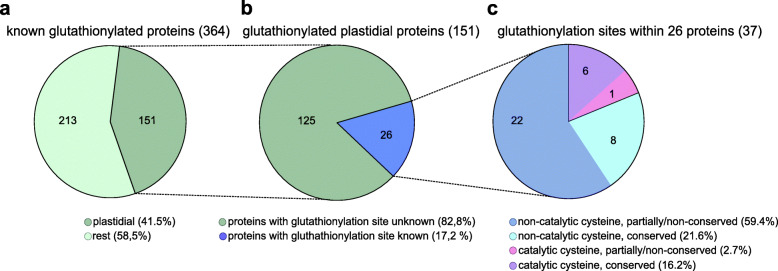
Overview of glutathionylation target proteins. **a** Overview of all known glutathionylated proteins in green eukaryotes with the plastidial glutathionylation target proteins highlighted. **b** Overview of all known plastidial glutathionylation target proteins with known glutathionylation sites highlighted. **c** Overview of all known plastidial glutathionylation sites with classification in evolutionary conserved and non−/partially conserved cysteine sites and subdivision in catalytic and non-catalytic function (for accessions, annotation and references, see Additional file 9 Table S2)

The vast majority of these enzymes are regulated by TRX through dithiol/disulfide interchanges that induce conformational changes either negatively or positively modulating protein activity [5, 6]. Besides TRX-dependent regulation, *S*-glutathionylation has recently emerged as an important regulatory mechanism in plants. It is involved in the recycling of antioxidant enzymes, but it can also protect protein cysteines from irreversible oxidation and modulate protein function/activity [3, 60, 69]. Many glutathionylation target proteins and the exact Cys that undergo *S*-glutathionylation remain unknown (Fig. 2). *S*-glutathionylation is not routinely detected in proteomics experiments in which cysteines are usually reduced, removing reversible modifications such as *S*-glutathionylation, and subsequently treated with Cys-blocking agents as a standard modification for MS/MS. However, several proteomic studies have developed specific protocols to detect *S*-glutathionylation and identified hundreds of putative target proteins highlighting the role of *S*-glutathionylation as thiol switching regulatory mechanism in eukaryotic oxygenic phototrophs [3] (and references therein). A BioGSSG-based (biotinylated glutathione disulfide) proteomic study was carried out in the cyanobacterium *Synechocystis* PCC 6803 and 383 proteins were identified as putative *S*-glutathionylated targets [70]. This study underpins the hypothesis that *S*-glutathionylation might have a regulatory role in all oxygenic phototrophs. Due to the importance of this post-translational redox modification, we decided to examine its relevance for plastidial proteins by analysing data from proteomic studies that used different methodologies, i.e. biotinylated GSH (biotinylated GSH ethyl ester (BioGEE)) [71], biotinylated GSSG (BioGSSG) [72, 73], anti-GSH antibodies [74] or radiolabelling of the glutathione pool using ^35^S-cysteine [75]. In order to obtain an exhaustive and complete list of *S*-glutathionylated proteins, we also considered research studies carried out on purified proteins in vitro [71, 76–93] (see Additional file 9 Table S2). Combining all these studies, we compiled a list of 364 proteins known to undergo *S*-glutathionylation in green eukaryotes (Additional file 9 Table S2, Fig. 3).

We determined the subcellular localisation of glutathionylated proteins based on biological function and prediction tools such as TargetP [45, 46] and SUBA (Subcellular Localisation Database for *Arabidopsis thaliana* [94]). Among the 364 proteins, 151 proteins are localised to plastids (Additional file 9 Table S2, Fig. 3), corresponding to c. 41% of the known plant glutathionylome. Subsequently, we explored for which plastidial proteins the exact site of *S*-glutathionylation was determined. One proteomic work in the chlorophyte *C. reinhardtii* combined the identification of glutathionylated targets with streptavidin enrichment using biotinylated-tagged peptides [73]. This approach allows establishing the exact *S*-glutathionylation sites if the identified peptide contains only one Cys residue. To further extent the analysis, we also considered studies on recombinant plastidial proteins in which the cysteine residues undergoing *S*-glutathionylation were identified by in vitro oxidant treatments coupled to mass spectrometry analysis [76–82]. Among 151 plastidial proteins, we found 37 glutathionylation sites with known target Cys within 26 different proteins (Table 1 and Additional file 9 Table S2), representing c. 17% of the known plastidial glutathionylome (Fig. 3).

**Table 1:** Overview of glutathionylation sites on plastid proteins and evolutionary conservation

	Protein name	Cys	Org.	Cat.	Ref.
*Redox regulation*	2-Cys peroxiredoxin	172	Ps	yes	Calderón et al. 2017 [92]
	Glutaredoxin S12 (GRXS12)	29	Pt	yes	Zaffagnini et al. 2012 [73]
	Thioredoxin f (TRX-f)	60	At	no	Michelet et al. 2005 [31]
*Photosynthesis*	Ferredoxin 1	48	Cr	no	Zaffagnini et al. 2012 [73]
		69	Cr	yes	Zaffagnini et al. 2012 [73]
	Fructose-1,6-bisphosphatase	109	Cr	no	Zaffagnini et al. 2012 [73]
	Fructose-1,6-bisphosphate aldolase	58	Cr	no	Zaffagnini et al. 2012 [73]
	Glyceraldehyde-3-phosphate dehydrogenase, A subunit (GAPA)	156	At	yes	Zaffagnini et al. 2007 [93]
	Plastocyanin	130	Cr	yes	Zaffagnini et al. 2012 [73]
	Phosphoglycerate kinase	159	Cr	no	Zaffagnini et al. 2012 [73]
		412	Cr	no	Zaffagnini et al. 2012 [73]
	Phosphoribulokinase	47	Cr	no	Zaffagnini et al. 2012 [73]
		274	Cr	no	Zaffagnini et al. 2012 [73]
	Photosystem II (PSII) core phosphatase (PBCP)	168	Os	no	Liu et al. 2019 [79]
		176	Os	no	Liu et al. 2019 [79]
		195	Os	no	Liu et al. 2019 [79]
	Ribulose bisphosphate carboxylase large chain	172	Cr	no	Zaffagnini et al. 2012 [73]
		247	Cr	no	Zaffagnini et al. 2012 [73]
		427	Cr	no	Zaffagnini et al. 2012 [73]
	Transketolase	84	Cr	no	Zaffagnini et al. 2012 [73]
	Triose phosphate isomerase (chloro TPI)	15	At	no	López-Castillo et al. 2016 [80]
*Carbohydrate metabolism*	ADP-glucose pyrophosphorylase large subunit	112	Cr	no	Zaffagnini et al. 2012 [73]
	Alpha-amylase 3 (AMY3)	499	At	yes	Gurrieri et al. 2019 [77]
		587	At	yes	Gurrieri et al. 2019 [77]
	Beta-amylase 3 (BAM3)	433	At	no	Storm et al. 2018 [78]
*Biosynthesis*	Acetohydroxy acid isomeroreductase	439	Cr	no	Zaffagnini et al. 2012 [73]
	Full-length thiazole biosynthetic enzyme	106	Cr	no	Zaffagnini et al. 2012 [73]
	Isopropylmalate dehydratase, large subunit	444	Cr	no	Zaffagnini et al. 2012 [73]
	Magnesium-chelatase subunit chlI	184	Cr	no	Zaffagnini et al. 2012 [73]
*others*	3′-phosphoadenosine 5′-phosphate phosphatase SAL1	119	At	no	Chan et al. 2016 [76]
		190	At	no	Chan et al. 2016 [76]
	Chaperonin 60B2	249	Cr	no	Zaffagnini et al. 2012 [73]
		537	Cr	no	Zaffagnini et al. 2012 [73]
	Heat shock protein 70B (HSP70B)	349	Cr	no	Michelet et al. 2008 [75]
	Phosphorylase	171	Cr	no	Zaffagnini et al. 2012 [73]
	Protein tyrosine phosphatases (PTP)	78	At	no	Dixon et al. 2005 [72]
		176	At	no	Dixon et al. 2005 [72]

Protein names and putative functions were partly assigned on sequence similarity and/or phylogenetic trees; please refer to the cited literature and references therein. *Cys* position of identified cysteine, *Org* organism, *Cat* catalytic cysteine, *Ref* literature reference, *At Arabidopsis thaliana*, *Cr Chlamydomonas reinhardtii*, *Os Oryza sativa*, *Ps Pisum sativum*, *Pt Populus trichocarpa*

The number of sites exceeds the number of proteins as several glutathionylated proteins contain multiple *S-*glutathionylation sites (Table 1 and Additional file 9 Table S2). More precisely, seven proteins contain two glutathionylated Cys and two proteins contain three glutathionylated Cys (Table 1 and Additional file 9 Table S2). The proteins with identified *S-*glutathionylation sites are putatively involved in diverse cellular processes such as photosynthesis, carbohydrate metabolism, biosynthetic pathways, redox regulation, signalling and protein homeostasis (Table 1).

To assess the evolutionary conservation of Cys undergoing glutathionylation, we constructed multiple sequence alignments of the 26 plastidial proteins (Additional file 10) from all analysed plant model species. We found that among the 37 known *S-*glutathionylation sites seven sites were involved in catalytic activity, of which six sites were fully conserved from green algae to flowering plants (Fig. 3, Additional file 9 Table S2, Additional file 10). Thirty glutathionylation sites were identified on non-catalytic cysteines (Fig. 3) of which eight Cys were nevertheless fully conserved in all investigated model species. The remaining 22 Cys were not or only partially conserved (Additional file 10), exhibiting different patterns of evolutionary gains and losses (Fig. 3 and Additional file 9 Table S2).

**Fig. 4:**
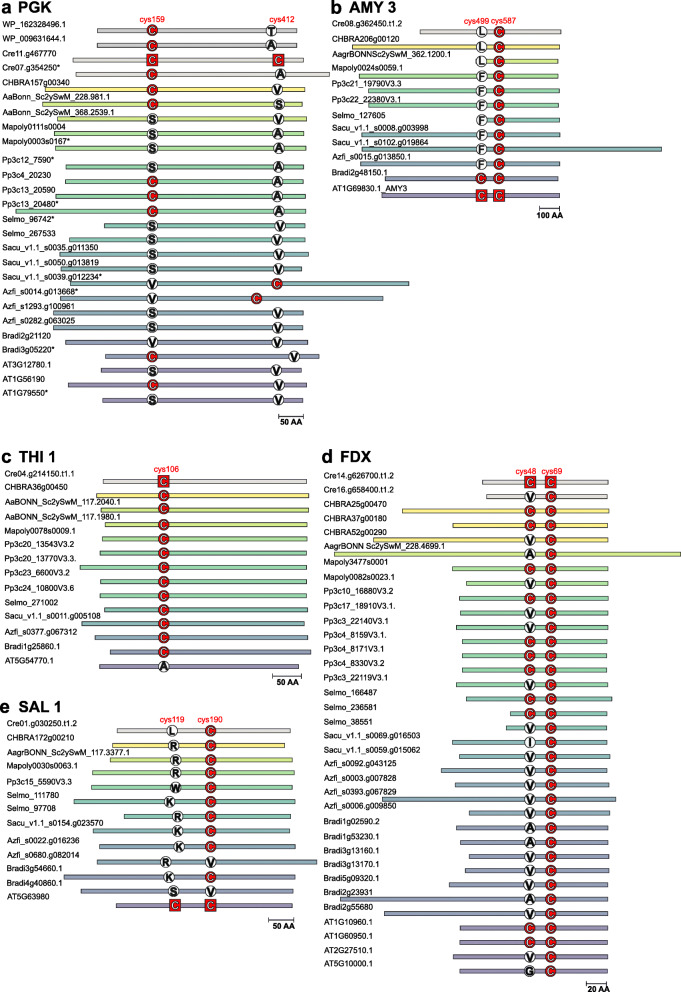
Evolutionary conservation of known *S*-glutathionylation sites on non-catalytic cysteines. Schematic representation of five target proteins with non-conserved and non-catalytic cysteines showing interesting patterns of evolutionary conservation regarding *S-*glutathionylation sites (see Additional file 10 for alignments). To generate the alignments BlastP results were filtered manually by clades based on phylogenetic trees and additionally length of the N-terminus and TargetP [45, 46] predictions to identify the organellar isoforms of phosphoglycerate kinase (**a**), alpha-amylase (AMY3) (**b**), thiamine thiazole synthase (THI1) (**c**), apoferredoxin (FDX) (**d**) and 3’phosphoadenosine 5′ phosphate phosphatase (SAL1) (**e**). The bars indicate the total length of the proteins and are aligned with the position of the glutathionylated cysteine. In the case of two cysteine positions, the proteins were aligned to the cysteine that is conserved in more species. Gaps in the alignments are not indicated in this graph. The known glutathionylated cysteine sites are marked with a red square in the respective organism. Asterisks indicate putative cytosolic isoforms (TargetP [45, 46] predictions) regarding PGK. *Synechocystis sp. (WP), Chara braunii (Chbra), Anthoceros agrestis (Aa), Marchantia polymorpha (Mapoly), Physcomitrium patens (Pp), Selaginella moellendorffii (Selmo), Salvinia cucullata (Sacu), Azolla filiculoides (Azfi), Brachypodium distachyon (Bradi)* and *Arabidopsis thaliana (At)*

Five examples of interesting gains and losses of putative *S-*glutathionylation sites are illustrated in Fig. 4. In alpha-Amylase 3 (AMY3) and SAL1 (3′-phosphoadenosine 5′-phosphate phosphatase) two *S*-glutathionylation sites were identified in each of the *A. thaliana* homologs. Cys499 in AMY3 is only present in the investigated angiosperm models. An additional sequence alignment incorporating basal angiosperm sequences (see Additional file 11) revealed the presence of Cys 499 in *Amborella trichopoda* and *Ananas comosus* AMY3 homologs. Cys119 of AtSAL1 is only conserved in about a third of eudicots and few monocots [76] and we do not identify any Cys at the homologous position in any non-flowering model plant. These data suggest an origin of cysteines at the Cys499 position in AMY3 and Cys119 position in SAL1 at the latest in early angiosperm evolution. In contrast, Cys106 of thiamine thiazole synthase (THI1) is present in *C. reinhardtii* and all homologs of investigated land plant models, except for *A. thaliana*, suggesting a late loss in land plant evolution. Several of the putative *S*-glutathionylation sites show variable conservation patterns that suggest several independent gains and losses in land plant lineages, such as Cys190 of SAL1, Cys159 and Cys412 in phosphoglycerate kinase (PGK) and Cys48 in ferredoxin.

## Discussion

### The plastid glutathione-dependent redox network is largely conserved

In a redox cascade, the steady-state redox status of an individual protein cysteine is influenced by the input of electrons from nucleophiles (i.e. electron donors), as well as the presence of proteins or molecules that interact and behave as electrophiles (i.e. electron acceptors). In cell compartments, thiol-switching networks can thus be limited by kinetic and thermodynamic parameters, such as the composition of the proteome, the ability of the present proteins to interact and the respective midpoint potentials of involved disulfides, as well as the reactivity of individual cysteine residues towards ROS/RNS. Glutathione-dependent reactions can be disturbed by mutants of either GSH biosynthesis enzymes [95, 96] or by mutants of GR, shifting the glutathione redox potential to less reducing values [29, 33]. The reported phenotypes of mutants lacking a stromal GR differed in *A. thaliana* and *P. patens*, as embryo development is aborted in *A. thaliana* but is completed in *P. patens*. Evolutionary changes could in theory either affect the composition and activity of redox-active proteins present, the presence and position of target thiols on metabolic enzymes, or signalling cascades linked to plastid glutathione redox balance. We compiled a list of protein members in the glutathione-dependent plastid redox network in representative streptophyte model species (Fig. 1) and their network relationships and evolution (Fig. 2). We found that plastid targeting of several GSSG-producing enzymes was already established before the transition to land and that no large expansions occurred in the gene families of DHAR, GSTL, GSTI, PRXIIE or class I and class II GRX during land plant evolution. However, several enzymes of ROS scavenging and damage repair families (DHAR, GSTL, GSTI, MRSB1) do not currently have an identified plastid-targeted isoform in the streptophyte alga *C. braunii*. However, as targeting predictions via algorithms for evolutionary distant species are prone to result in false positives and negatives [48, 97], we used three different predictors, namely the two most recently improved tools based on the largest plant training sets TargetP2.0 [46] and LOCALISER [47], as well as PredAlgo, that was trained on *C. reinhardtii* sequences. However, we still likely underestimate the number of plastid-targeted proteins, due to (1) false predictions and (2) fragmentary gene models that do not predict N-terminal sequences correctly. Localisation predictions often yielded different results (Additional file 1 Table S1, sheet2) and differed from experimentally determined localisations (e.g. AtMSRB1). Thus, we additionally screened for the presence of N-terminal extensions to determine the presence or absence of a putative targeting peptide (Additional files 2, 3, 4, 5, 6, 7, 8). However, incorrect or fragmentary gene models, can still lead to misinterpretation of the available data. Thus, the available *S. moellendorffii* protein models do often not allow one to predict N-terminal targeting signals or draw conclusions on presence or absence of N-terminal extensions due to incompleteness (Fig. 2).

In some of the investigated gene families, we found variable targeting to organelles and presence or absence of N-terminal extensions across land plant evolution, whereas others showed very consistent targeting. Thus, we observed variable targeting in the GST subfamilies DHAR, GSTL and GSTI. DHAR couples the regeneration of dehydroascorbate to the glutathione pool in the ascorbate/glutathione cycle, while monodehydroascorbate reductase regenerates ascorbate at the expense of NADH [24]. In *A. thaliana*, stromal DHAR is not necessary for growth, but takes a role in the high light response [98]. Variability in DHAR targeting would indicate evolutionary variability in the coupling of the ascorbate redox state to the *E*_GSH_ in different compartments, which could affect signalling responses. Regarding GSTL and GSTI, targeting may be variable, but these GST subfamilies show a nearly complementary distribution in land plant lineages, with GSTI being present in bryophytes and GSTL in the moss *P. patens*, ferns and seed plants. The function of GSTI has not been established in vitro, but is suspected to be similar to GSTL, acting on the detoxification of peroxides [50]. In plastids, several pathways for hydrogen peroxide and lipid peroxide detoxification co-exist (ascorbate peroxidases [8], TRX-dependent GPXL [99] and PRXs (PRXQ, 2-Cys PRX, PRXIIE [15]), suggesting that redundancy might be a reason for independent gains and losses in the investigated protein families during land plant evolution. Similarly, regarding the repair of oxidised methionine (methionine-*R*-sulfoxide), atypical MSRB with one cysteine (AtMSRB1 homologs) as well as with two cysteines (AtMSRB2 homologs) act redundantly with the difference of regeneration being powered from a different redox cascade (i.e. GSH/GRX and TRX, respectively). Thus, if species are lacking a plastidial 1-Cys MSRB1 homolog, the 2-Cys MSRB2 may compensate and still allow for repair of the *R* diastereomer of MetSO. The biological significance of the variable composition of scavenging and repair enzymes in plastids during streptophyte evolution is yet unclear but raises the question if the only reason is functional redundancy, or if parallel redox cascades with coupling to different electron donors are important for processes such as stress signalling or relate to differences between tissues or light conditions.

On the contrary, PRXIIE, GRXC5/S12, GRXS14, GRXS16 homologs are present as a single plastid isoform in the majority of species, suggesting conserved plastid-specific functions and tight control of gene copy number. Although peroxide detoxification is linked to TRX via several PRXs (PRXQ and 2-Cys PRX, [15]) the plastid isoform PRXIIE that can be more efficiently regenerated via GSH/GRX is likely fully conserved in streptophytes (Fig. 2). The strict conservation of PRXIIE isoforms suggests an important specific biological function, although the abundance of PRXIIE is substantially lower than that of plastid 2-Cys PRX [15]. Its specific properties include that PRXIIE is the only PRX not associated with thylakoids, that it is coupled to GRX for reduction and that it has been linked to RNS signalling [15, 100].

PRXIIE and MSRB1 are regenerated via the action of class I GRX. Besides, GRXC5 can bind a [2Fe-S] cluster that might act in a regulatory mechanism under oxidative stress conditions [66]. We found a conserved clade containing plastid-targeted GRXC5/S12 homologs. As an exception, we did not identify a GRXC5 nor GRXS12 isoform in the fern *A. filiculoides*. However, isoforms in the C1/C2 (Azfi_s0158.g053892; Azfi_s0074.g037496) as well as the C3/C4 (Azfi_s0270.g061184; Azfi_s0004.g008860) clades underwent duplication and are candidates for re-targeting to plastids to compensate for the loss of the plastid-targeted class I isoform. In *C. braunii* and non-seed plants, only one isoform with a CPYC active site is present (GRXC5), indicating that CPYC is the ancestral active site motif of this GRX isoform, while the GRXS12 variant only appeared in angiosperm evolution.

Class II GRX isoforms are involved in the coordination and transfer of Fe-S cluster on target proteins [64, 65] and become only redox-active after loss of the coordinated Fe-S cluster. Without Fe-S cluster, they are able to catalyze *S-*glutathionylation in vitro in the presence of GSSG, but fail to deglutathionylate target proteins such as redox-sensitive GFP [65]. In vivo evidence revealed that GRXS14 levels correlate with tolerance to abiotic stress conditions in either Arabidopsis [62, 101] or tomato [102]. In addition, GRXS14 along with GRXS16 are necessary for functional photosynthesis and chlorophyll biosynthesis [62]. In *C. reinhardtii*, class II GRX3, a GRXS14 homolog, was found to catalyse deglutathionylation of photosynthetic GAPDH from *Arabidopsis thaliana* (AtGAPA) using electrons from PSI-reduced ferredoxin [103]. However, an analogous function was not shown for land plant GRXS14 homologs to date. Our analysis confirmed the strict conservation of one GRXS14 and GRXS16 homolog in plastids in all model organisms, except for *P. patens* with two GRXS14 isoforms. However, the functional and physiological relevance of class II GRX redox-activity remains unclear.

Reduction of class I GRXs is driven by glutathione, while GR maintains a highly reducing *E*_GSH_ in the plastid stroma, re-reducing the generated GSSG. We confirmed two GR clades in streptophytes likely containing the dual-targeted isoform to cytosol/peroxisomes and mitochondria/plastids. At least one isoform of the mitochondrial/plastidial GR clade and one of the cytosolic/peroxisomal clade is conserved in all land plant model species, while one additional isoform originating from horizontal gene transfer (HGT) is present in *Anthoceros* species. The most closely related bacterial sequence in Uniprot [104] is from the cyanobacterium *Nostoc* spec. (Uniprot ID P48638), suggesting that this GR isoform was acquired from cyanobacteria, which are known to colonise hornworts [40, 42]. The presence of putative N-terminal extensions in the GRs originating from HGT is interesting (Additional file 8), as they might confer targeting to organelles, raising the question of the biological function of the additional GR in the investigated hornworts.

### Non-catalytic cysteines sensitive to *S*-glutathionylation are evolutionary conserved

Cysteines are central for the structural integrity and catalytic activity of many proteins, and post-translational modifications of Cys residues control major steps in plant signalling and metabolism [105, 106]. The biological role of *S*-glutathionylation is only resolved for a few exemplary target proteins and ranges from reaction intermediates on active site Cys of scavenging/repair enzymes over protection from Cys overoxidation to regulation of protein activity [3, 60, 69].

To date, 151 different plastid proteins were identified as targets for *S*-glutathionylation by various experimental approaches in different plant systems, suggesting that this post-translational redox modification might be quite common in plastids. However, the exact number of proteins in plastids that are glutathionylated at a given time point in a given organism remains unknown and is potentially rather low, for a number of reasons.

Firstly, high-throughput methods used to identify *S*-glutathionylation partly use reaction conditions that are non-physiological. For instance, BioGSSG is used to induce protein *S*-glutathionylation. However, this mechanism is unlikely as (i) GSSG concentration is physiologically in the nanomolar range while a millimolar concentration of BioGSSG is used in vitro and (ii) most proteins have a *K*_ox_ of around 1 meaning that equimolar concentration of GSH and GSSG are required to reach 50% of glutathionylated protein [107, 108]. GSSG can react with a cysteine in its thiolate state (−S^−^) via a thiol-disulfide exchange reaction. However, the GSSG-dependent mechanism is known to be thermodynamically disadvantageous because of the high in vivo GSH/GSSG ratio, and only some protein species have been shown to be able to undergo *S*-glutathionylation via this pathway [60, 109]. Some proteins undergo *S*-glutathionylation following exposure to the nitrosylated counterpart of glutathione known as *S*-nitrosoglutathione (GSNO). While GSNO is a nitrosylating agent [110], it has been observed to induce *S*-glutathionylation on certain protein species probably depending on the microenvironment of their cysteine [60, 111–113]. Another physiologically likely pathway to *S*-glutathionylation occurs via nucleophilic attack of glutathione on *S*-sulfenylation sites that are formed by the reaction of protein thiols with H_2_O_2_. Using exogenous application of 0.1–20 mM H_2_O_2_, between 68 and 132 different plastid proteins were identified as prone to *S*-sulfenylation [114–116]. How many proteins undergo *S*-sulfenylation and potentially subsequent *S*-glutathionylation under physiologically relevant H_2_O_2_ concentrations in plastids remains unclear.

Secondly, many proteins are potentially rapidly de-glutathionylated in vivo. In plastids, the de-glutathionylation would require interaction with the plastid class I GRXC5/S12 isoforms (Fig. 2). Based on experimental evidence, there is no functional redundancy between TRXs and GRXs despite their structural similarity, and they are specifically dedicated to the redox control of protein disulfides and *S*-glutathionylation, respectively. To date, the reduction of glutathionylated cytosolic glyceraldehyde-3-phosphate dehydrogenase from *Arabidopsis thaliana* (AtGAPC) constitutes the sole exception since both enzymes can efficiently catalyse its deglutathionylation [87, 117]. Increased GSSG levels and/or less reducing stromal glutathione redox potential might influence the level of *S*-glutathionylation on proteins in vivo, e.g. via decreased rates of de-glutathionylation. As we identified a dynamic oxidative response of the stromal glutathione redox potential to a transition from light to dark [33], future studies need to investigate the biological relevance of these redox dynamics and a putative influence on *S*-glutathionylation levels on proteins.

Unfortunately, the exact position of only 37 Cys (on 26 different plastid proteins) susceptible to *S*-glutathionylation is known, representing c. 17% of the different identified glutathionylated proteins in plastids. Catalytic Cys were fully conserved with the exception of Cys499 in AMY3 (Fig. 4, Additional file 9: Table S2). Here, the annotation as catalytic Cys is based on a mutant protein in *A. thaliana* that largely lacks catalytic activity [118]. However, the mechanism is unknown and Cys499 might not be strictly necessary for catalytic activity in other organisms, based on our analysis. Notably, c. 22% of the known sites on non-catalytic Cys were fully conserved from green algae to flowering plants. This suggests an important regulatory role of these Cys, including the potential for modification via *S*-glutathionylation.

### Distinct conservation patterns of putative glutathionylation sites

Our evolutionary analysis on conservation of Cys undergoing *S*-glutathionylation in at least one species revealed that several regulatory Cys are re-appearing in land plant evolution suggesting independent gains and losses of these sites (Fig. 4, FDX, PGK, SAL1).

Regarding phosphoglycerate kinase (PGK), we found a decreasingly complex regulation during evolution. In the green alga *C. reinhardtii* two *S*-glutathionylation sites (CrPGK1 Cys159 and Cys412) were identified and *S*-glutathionylation confirmed in vitro [73]. Interestingly, Cys412 is conserved in human and mouse [75] but not in most land plants, although it forms a regulatory disulfide with Cys278 in CrPGK1 [81]. Cys159 is partially conserved in some land plant homologs. Most investigated species encode for several isoforms of which at least one contains Cys159, except for *M. polymorpha* and the fern model species. In *A. thaliana*, the function of the different isoforms is known, with AtPGK3 (AT1G79550) serving as the cytosolic glycolytic isoform, AtPGK1 (AT3G12780) as the photosynthetic isoform, and AtPGK2 (AT1G56190) as the plastidial glycolytic isoform [119]. AtPGK1 and AtPGK2 are thus participating in the Calvin-Benson cycle and plastidial glycolysis, respectively, but catalysing the inverse reaction. Cys159 is only conserved in the plastidial glycolytic isoform in *A. thaliana*. It is tempting to speculate that *S-*glutathionylation is contributing to differential regulation between photosynthetic and glycolytic isoforms in plastids.

Similarly, a highly conserved Cys is not present in the *A. thaliana* ortholog of thiamine thiazole synthase (THI1) (Fig. 4c). THI1 is synthetizing the thiazole moiety of thiamine (vitamin B1). It is a suicide enzyme undergoing a single catalytic turnover, as the sulfide is transferred from a conserved Cys [120] (corresponding to AtTHI1 Cys216). The Cys of one *S*-glutathionylation site (Cys106 identified in *C. reinhardtii*) is strictly conserved, except for *A. thaliana* (A98). The position of Cys106 is in the alpha 2 helix [121] within a highly conserved motif of unknown function. However, the meaning of a potential redox regulation in single-turnover enzymes remains unclear.

In contrast, the redox regulation of alpha-amylase 3 (AMY3) seems to be increasingly complex in evolution. Whereas two *S*-glutathionylation sites were identified in *A. thaliana*, only one Cys (Cys587) is strictly conserved. The second Cys (Cys499) is only present in the investigated angiosperm models. It was already shown that AtAMY3 is more active in its reduced form [118], with a midpoint redox potential of − 329 mV (pH 7.9) and that it is most efficiently reduced by TRX f, m and y. Whereas the single Cys mutant C587S was retaining activity under oxidising conditions, the Cys499 mutant was nearly inactive [118]. AMY3 plays a role in starch metabolism associated with stomatal opening and is involved in the response to stress [122]. In the presence of H_2_O_2_ it undergoes a partial irreversible inactivation due to the oxidation of cysteines, which is prevented by *S*-glutathionylation on at least three different Cys, including Cys499 and Cys587. Once glutathionylated, the enzyme can be reverted to its active state via GRXs or TRX, if the *S*-glutathionylation is resolved by formation of an intramolecular disulfide [77].

Similarly, two *S*-glutathionylation sites were identified in the *A. thaliana* ortholog of the 3′-phosphoadenosine 5′-phosphate phosphatase SAL1 of which only Cys190 is conserved in at least one homolog of all investigated model species. Cys119 is only present in *A. thaliana* with positively charged AA residue being present in most other species at the equivalent position. SAL1 has an important evolutionarily conserved function in the regulation of PAP levels and thereby in plastid to nucleus retrograde signalling [123] (Zhao et al., 2019). A “moonlighting” signalling function by secondary redox sensing was already described for SAL1 [76] via the fully conserved Cys167 that forms a cross beta-strand disulfide bond with Cys190. Cys119 or Cys190 are required for deactivation under oxidising conditions in *A. thaliana* with Cys119 being involved in intermolecular disulfide and dimer formation, facilitating the cross beta-strand disulfide bridge Cys167-Cys190 [76]. The redox-regulatory mechanism in *A. thaliana* consists of first dimerization and subsequent oxidative inactivation. Alternatively, *S*-glutathionylation on Cys119 or Cys190 did decrease the activity in monomer or dimer (midpoint redox potential − 308 mV monomer, pH 7.5).

Finally, we found variable conservation of putative *S*-glutathionylation sites, with potentially several independent gains and losses in ferredoxins (FDX). While Cys69, that is one of four 2Fe-2S coordinating cysteines, is strictly conserved, there is variable conservation of Cys48. Cys48 *S*-glutathionylation was identified in *C. reinhardtii* and this residue is present in *A. thaliana* leaf-type ferredoxins (FDX1 (AT1G10960) and FDX2 (AT1G60950)), but not in root-type ferredoxin FDX3 (AT2G27510) and FDX4 (AT5G10000) [124]. It is possible that there is a different redox regulation of leaf-type FDX that are reduced by PSI, compared to root-type FDXs, that are reduced by FNR and are more efficient electron donors to sulphite reductase [124]. However, Cys48 is conserved in none of the FDX sequences from fern model species and the monocot model *B. distachyon*. This suggests either independent losses of Cys48 in these plant lineages, or the independent re-appearance of a regulatory Cys at the same position during land plant evolution.

## Conclusions

By analysing enzymes drawing electrons from the glutathione pool and producing GSSG we found that GR and GRX isoforms are largely conserved between streptophyte algae and land plant model species, identifying them as central players of plastid glutathione-dependent redox cascades. Here, GRXC5 is the ancestral isoform likely involved in protein de-glutathionylation. The composition of scavenging and damage repair enzymes in plastids was evolutionary less conserved except for PRXIIE. This indicates variability of ROS-scavenging and damage repair between different species and highlights that PRXIIE might be necessary for plastid redox regulation. As we found evolutionary conservation of many known *S*-glutathionylation sites on plastid proteins, including non-catalytic cysteines, we conclude that protein *S*-glutathionylation in plastids plays an important and yet under-investigated role in redox regulation and stress response. Future challenges are to determine (i) new targets of *S*-glutathionylation, (ii) the exact position of *S*-glutathionylation in more target proteins, (iii) the in vivo dynamics of protein *S*-glutathionylation and the resulting steady-state level of the *S*-glutathionylated fraction of target proteins and (iv) the biological relevance of this modification for plastid function.

## Methods

### Sequence retrieval and alignment

To reconstruct gene diversification of components of plastid and mitochondrial redox cascades, protein sequences were retrieved from OrcAE for *Chara braunii* (http://www.bioinformatics.psb.ugent.be/orcae/overview/Chbra), Phytozome v12.1 [125] for *Chlamydomonas reinhardtii*, *Marchantia polymorpha*, *Physcomitrium* (*Physcomitrella) patens, Selaginella moellendorffii* and *Brachypodium distachyon*, Fernbase (http://www.fernbase.org) for *Salvinia cucullata* and *Azolla filiculoides*, and TAIR10 (www.arabidopsis.org) for *Arabidopsis thaliana*. Additionally, protein sequences encoded by organellar genomes were retrieved from Uniprot [104]. BLAST access to *Anthoceros agrestis* and *Anthoceros punctatus* genomes [42] was provided by Prof. Peter Szövenyi. Alignments were constructed using JalView [126] and the Muscle algorithm with default settings.

### Construction of phylogenetic trees

Phylogenetic trees were generated from manually curated alignments (see Additional file 12) using Bayesian inference with MrBayes (run parameters: mixed protein models, rates = invgamma, number of generations: 2*10^6^, burnin = 20%, end split frequencies< 0.01) [127]. As complementary method, Maximum Likelihood-based trees (see Additional file 13) were generated using iQtree web [128] (run parameters: 1000 bootstrap, standard settings).

Graphical representations of phylogenetic trees were created using the Figtree software (v1.4.2, A. Rambaut, http://tree.bio.ed.ac.uk/software/figtree/).

### Compiling the list of glutathionylated plastid proteins

Proteins found to be glutathionylated in different proteomic and in vitro studies [31, 60, 71–93] were retrieved from the literature and used to assemble a list (Additional file 9: Table S2, sheet1). NCBI reference sequences (www.ncbi.nlm.nih.gov) and TAIR (www.arabidopsis.org) accession numbers were used to unambiguously identify proteins.

Arabidopsis homologs of every protein were identified using the BLASTP tool from NCBI (https://blast.ncbi.nlm.nih.gov/Blast.cgi). The presumed subcellular localization of Arabidopsis homologs was retrieved from SUBA (Subcellular Localisation Database for *A. thaliana*) [94] using the SUBAcon algorithm in order to identify plastidial proteins.

### Identification of catalytic cysteine sites

Catalytic cysteines were identified among those found to be glutathionylated and belonging to plastid proteins (Additional file 9 Table S2, sheet2) using Uniprot [104] and the information found within the individual publications cited.

## Supplementary Information

**Additional file 1: Table S1.** containing protein model information and targeting.

**Additional file 2: Fig. S2**. Phylogenetic tree of DHAR. (a) Phylogenetic tree of DHAR isoforms (*P. patens* nomenclature according to Liu et al. (2013) [50]) constructed using MrBayes, node values and line weights depict posterior probabilities (run parameters: mixed protein models, rates = invgamma, number of generations: 2*10^6^, burnin = 20%, split frequencies< 0.01). ^1^*P. patens* DHAR1 was identified and quantified in mitochondrial and plastid proteomes [49] and is putatively dual targeted. TargetP2.0 (T) [46], LOCALIZER (L) [47] and PredAlgo (P) [48] predictions (Additional file 1 Table S1) indicate highly variable targeting of the multiple DHAR paralogs (M, mitochondria; P, plastid; O, other; S, secretory). The presence (check mark) or absence (X) of an N-terminal extension (ext. N) in the sequence is indicated; NA: not assessed as sequence potentially incomplete. Gene identifiers are given according to the used gene models for *Chara braunii* (CHBRA), *Anthoceros agrestis* strain Bonn (AaBonn), *Marchantia polymorpha* (Mapoly), *Physcomitrium patens* (Pp)*, Selaginella moellendorffii* (Selmo), *Salvinia cucullata* (Sacu), *Azolla filiculoides* (Azfi), *Brachypodium distachyon* (Bradi) and *Arabidopsis thaliana* (At) and are additionally color-coded as in Fig. 1. Colour legend: Cb = *Chara braunii*; Aa = *Anthoceros agrestis*; Mp = *Marchantia polymorpha*; Pp = *Physcomitrium patens*; Sm = *Selaginella moellendorffii*; Sc = *Salvinia cucullata*; Af = *Azolla filiculoides*; Bd = *Brachypodium distachyon*; At = *Arabidopsis thaliana*. (b) N-terminal part of protein alignment (Jalview) showing the presence or absence of N-terminal extensions indicative of putative N-terminal targeting peptides. Colour-scheme: ClustalX.

**Additional file 3: Fig. S3.** Phylogenetic tree of lambda and iota-type GSTs. (a) Phylogenetic tree of lambda- and iota-type glutathione *S*-transferase isoforms (*P. patens* nomenclature according to Liu et al. (2013) [50]) constructed using MrBayes, node values and line weights depict posterior probabilities (run parameters: mixed protein models, rates = invgamma, number of generations: 2*10^6^, burnin = 20%, split frequencies< 0.01). TargetP2.0 (T) [46], LOCALIZER (L) [47] and PredAlgo (P) [48] predictions (Additional file 1 Table S1) indicate variable targeting of GSTL and GSTI isoforms to plastids (M, mitochondria; P, plastid; O, other; S, secretory). The presence (check mark) or absence (X) of an N-terminal extension (ext. N) in the sequence is indicated; NA: not assessed as sequence potentially incomplete. Gene identifiers are given according to the used gene models for *Chlamydomonas reinhardtii* (Cre), *Chara braunii* (CHBRA), *Anthoceros agrestis* strain Bonn (AaBonn), *Marchantia polymorpha* (Mapoly), *Physcomitrium patens* (Pp)*, Selaginella moellendorffii* (Selmo), *Salvinia cucullata* (Sacu), *Azolla filiculoides* (Azfi), *Brachypodium distachyon* (Bradi) and *Arabidopsis thaliana* (At) and are additionally color-coded as in Fig. 1. Colour legend: Cb = *Chara braunii*; Aa = *Anthoceros agrestis*; Mp = *Marchantia polymorpha*; Pp = *Physcomitrium patens*; Sm = *Selaginella moellendorffii*; Sc = *Salvinia cucullata*; Af = *Azolla filiculoides*; Bd = *Brachypodium distachyon*; At = *Arabidopsis thaliana*. (b) N-terminal part of protein alignment (Jalview) showing the presence or absence of N-terminal extensions indicative of putative N-terminal targeting peptides. Colour-scheme: ClustalX.

**Additional file 4: Fig. S4**. Phylogenetic tree of methionine sulfoxide reductases B. (a) Phylogenetic tree of methionine sulfoxide reductase B (MSRB) isoforms constructed using MrBayes, node values and line weights depict posterior probabilities (run parameters: mixed protein models, rates = invgamma, number of generations: 2*10^6^, burnin = 20%, split frequencies< 0.01). TargetP2.0 (T) [46], LOCALIZER (L) [47] and PredAlgo (P) [48] predictions (Additional file 1 Table S1) indicate variable targeting of MSRB1 isoforms to plastids (M, mitochondria; P, plastid; O, other; S, secretory). The presence (check mark) or absence (X) of an N-terminal extension (ext. N) in the sequence is indicated; NA: not assessed as sequence potentially incomplete. Gene identifiers are given according to the used gene models for *Chlamydomonas reinhardtii* (Cre), *Chara braunii* (CHBRA), *Anthoceros agrestis* strain Bonn (AaBonn), *Marchantia polymorpha* (Mapoly), *Physcomitrium patens* (Pp)*, Selaginella moellendorffii* (Selmo), *Salvinia cucullata* (Sacu), *Azolla filiculoides* (Azfi), *Brachypodium distachyon* (Bradi) and *Arabidopsis thaliana* (At) and are additionally color-coded as in Fig. 1. Colour legend: Cb = *Chara braunii*; Aa = *Anthoceros agrestis*; Mp = *Marchantia polymorpha*; Pp = *Physcomitrium patens*; Sm = *Selaginella moellendorffii*; Sc = *Salvinia cucullata*; Af = *Azolla filiculoides*; Bd = *Brachypodium distachyon*; At = *Arabidopsis thaliana*. (b) N-terminal part of protein alignment (Jalview) showing the presence or absence of N-terminal extensions indicative of putative N-terminal targeting peptides. Colour-scheme: ClustalX. (c) In the presence of H_2_O_2_, methionine can be oxidised, a modification that can be resolved by MSRB regarding methionine-*R*-sulfoxide. The GRX-dependent reaction mechanism in atypical (1Cys) MSRB operates via an *S*-glutathionylation intermediate [53]. The GRX-dependent mechanism generates GSSG that in turn requires GR for reduction. (PDF 156 kb). (d) Conservation of threonine in the relative position to AtMSRB1 Thr132 (red arrow). Colour-scheme: ClustalX.

**Additional file 5: Fig. S5.** Phylogenetic tree of peroxiredoxin II E. (a) Phylogenetic tree of peroxiredoxin IIE (PRXIIE) isoforms constructed using MrBayes, node values and line weights depict posterior probabilities (run parameters: mixed protein models, rates = invgamma, number of generations: 2*10^6^, burnin = 20%, split frequencies< 0.01). TargetP2.0 (T) [46] predictions indicate conserved targeting of PRXIIE isoforms to plastids, while LOCALIZER (L) [47] and PredAlgo (P) [48] predictions vary (M, mitochondria; P, plastid; O, other; S, secretory) (Additional file 1 Table S1). The presence (check mark) or absence (X) of an N-terminal extension (ext. N) in the sequence is indicated; NA: not assessed as sequence potentially incomplete. Gene identifiers are given according to the used gene models for *Chara braunii* (CHBRA), *Anthoceros agrestis* strain Bonn (AaBonn), *Marchantia polymorpha* (Mapoly), *Physcomitrium patens* (Pp)*, Selaginella moellendorffii* (Selmo), *Salvinia cucullata* (Sacu), *Azolla filiculoides* (Azfi), *Brachypodium distachyon* (Bradi) and *Arabidopsis thaliana* (At) and are additionally color-coded as in Fig. 1. Colour legend: Cb = *Chara braunii*; Aa = *Anthoceros agrestis*; Mp = *Marchantia polymorpha*; Pp = *Physcomitrium patens*; Sm = *Selaginella moellendorffii*; Sc = *Salvinia cucullata*; Af = *Azolla filiculoides*; Bd = *Brachypodium distachyon*; At = *Arabidopsis thaliana*. (b) N-terminal part of protein alignment (Jalview) showing the presence or absence of N-terminal extensions indicative of putative N-terminal targeting peptides. Colour-scheme: ClustalX.

**Additional file 6: Fig. S6.** Phylogenetic tree of class I GRX. (a) Phylogenetic tree of class I glutaredoxin (GRX) isoforms constructed using MrBayes, node values and line weights depict posterior probabilities; nodes with lower support than 50% are collapsed (run parameters: mixed protein models, rates = invgamma, number of generations: 4*10^6^, burnin = 20%, split frequencies< 0.01). TargetP2.0 (T) [46], LOCALIZER (L) [47] and PredAlgo (P) [48] targeting predictions (Additional file 1 Table S1) are indicated (M, mitochondria; P, plastid; O, other; S, secretory). The presence (check mark) or absence (X) of an N-terminal extension (ext. N) in the sequence is indicated; NA: not assessed as sequence potentially incomplete. Gene identifiers are given according to the used gene models for *Chara braunii* (CHBRA), *Anthoceros agrestis* strain Bonn (AaBonn), *Marchantia polymorpha* (Mapoly), *Physcomitrium patens* (Pp)*, Selaginella moellendorffii* (Selmo), *Salvinia cucullata* (Sacu), *Azolla filiculoides* (Azfi), *Brachypodium distachyon* (Bradi) and *Arabidopsis thaliana* (At) and are additionally color-coded as in Fig. 1. Colour legend: Cb = *Chara braunii*; Aa = *Anthoceros agrestis*; Mp = *Marchantia polymorpha*; Pp = *Physcomitrium patens*; Sm = *Selaginella moellendorffii*; Sc = *Salvinia cucullata*; Af = *Azolla filiculoides*; Bd = *Brachypodium distachyon*; At = *Arabidopsis thaliana*. (b) N-terminal part of protein alignment (Jalview) showing the presence or absence of N-terminal extensions indicative of putative N-terminal targeting peptides. Colour-scheme: ClustalX.

**Additional file 7: Fig. S7**. Phylogenetic tree of plastid-targeted class II GRX. (a) Phylogenetic tree of the plastid class II glutaredoxin (GRX) S14 and S16 isoforms constructed using MrBayes, node values and line weights depict posterior probabilities; nodes with lower support than 50% are collapsed (run parameters: mixed protein models, rates = invgamma, number of generations: 2*10^6^, burnin = 20%, split frequencies< 0.01). TargetP2.0 (T) [46] predictions indicate conserved targeting of GRXS14 and GRXS16 isoforms to plastids, while LOCALIZER (L) [47] and PredAlgo (P) [48] predictions vary (M, mitochondria; P, plastid; O, other; S, secretory) (Additional file 1 Table S1). The presence (check mark) or absence (X) of an N-terminal extension (ext. N) in the sequence is indicated; NA: not assessed as sequence potentially incomplete. Gene identifiers are given according to the used gene models for *Chara braunii* (CHBRA), *Anthoceros agrestis* strain Bonn (AaBonn), *Marchantia polymorpha* (Mapoly), *Physcomitrium patens* (Pp)*, Selaginella moellendorffii* (Selmo), *Salvinia cucullata* (Sacu), *Azolla filiculoides* (Azfi), *Brachypodium distachyon* (Bradi) and *Arabidopsis thaliana* (At) and are additionally color-coded as in Fig. 1. Colour legend: Cb = *Chara braunii*; Aa = *Anthoceros agrestis*; Mp = *Marchantia polymorpha*; Pp = *Physcomitrium patens*; Sm = *Selaginella moellendorffii*; Sc = *Salvinia cucullata*; Af = *Azolla filiculoides*; Bd = *Brachypodium distachyon*; At = *Arabidopsis thaliana*. (b) N-terminal part of GRXS14 protein alignment (Jalview) showing the presence or absence of N-terminal extensions indicative of putative N-terminal targeting peptides. Colour-scheme: ClustalX. (c) N-terminal part of GRXS16 protein alignment (Jalview) showing the presence or absence of N-terminal extensions indicative of putative N-terminal targeting peptides. Colour-scheme: ClustalX.

**Additional file 8: Fig. S8**. Phylogenetic tree of glutathione reductases (GR). (a) Phylogenetic tree of glutathione reductase isoforms constructed using MrBayes, node values and line weights depict posterior probabilities; nodes with lower support than 50% are collapsed (run parameters: mixed protein models, rates = invgamma, number of generations: 0.5*10^6^, burnin = 20%, split frequencies< 0.01). TargetP2.0 (T) [46], LOCALIZER (L) [47] and PredAlgo (P) [48] targeting predictions (Additional file 1 Table S1) are indicated (M, mitochondria; P, plastid; O, other; S, secretory); experimental evidence for conserved dual targeting of GR2 isoforms:^1^ [67], ^2^ [29]. The presence (check mark) or absence (X) of an N-terminal extension (ext. N) in the sequence is indicated. Gene identifiers are given according to the used gene models for *Chlamydomonas reinhardtii* (Cre), *Chara braunii* (CHBRA), *Anthoceros agrestis* strain Bonn (AaBonn), *Marchantia polymorpha* (Mapoly), *Physcomitrium patens* (Pp)*, Selaginella moellendorffii* (Selmo), *Salvinia cucullata* (Sacu), *Azolla filiculoides* (Azfi), *Brachypodium distachyon* (Bradi) and *Arabidopsis thaliana* (At) and are additionally color-coded as in Fig. 1. Additional species: NOSS: *Nostoc* spec.; *Anthoceros agrestis* Oxford (AaOxford), *Anthoceros punctatus* (Ap), [40]. Colour legend: Cb = *Chara braunii*; Aa = *Anthoceros agrestis*; Mp = *Marchantia polymorpha*; Pp = *Physcomitrium patens*; Sm = *Selaginella moellendorffii*; Sc = *Salvinia cucullata*; Af = *Azolla filiculoides*; Bd = *Brachypodium distachyon*; At = *Arabidopsis thaliana*. (b) N-terminal part of GR protein alignment (Jalview) showing the presence or absence of N-terminal extensions indicative of putative N-terminal targeting peptides. Red bar marks prominent example of sequence lacking in bacterial and Anthoceros GRs, supporting an origin by horizontal gene transfer (HGT). GRs acquired by HGT possess putative N-terminal extensions, compared to bacterial GRs. Colour-scheme: ClustalX.

**Additional file 9: Table S2**. Lists of *S*-glutathionylation sites and organisms.

**Additional file 10 **Word-file containing all alignments used to assess conservation of known *S*-glutathionylation sites on plastid proteins in fasta format.

**Additional file 11.** Word-file containing AMY3 alignment with additional angiosperm sequences.

**Additional file 12.** Word-file containing all alignments used to build phylogenetic trees in FASTA format.

**Additional file 13.** Word-file containing phylogenetic trees generated with alternative method (Maximum Likelihood).

## Data Availability

All data generated or analysed during this study are included in this published article and its supplementary information files. The used databases are publicly accessible and available online: https://bioinformatics.psb.ugent.be/orcae/, https://phytozome.jgi.doe.gov/pz/portal.html, https://www.fernbase.org, https://www.arabidopsis.org, https://www.uniprot.org/, https://suba.live/, https://www.ncbi.nlm.nih.gov/.

## Abbreviations

ROS: reactive oxygen species
CBC: Calvin-Benson cycle
RNS: reactive nitrogen species
GSH: glutathione
TRX: thioredoxin
GR: glutathione reductase
FTR: ferredoxin-thioredoxin reductase
NTR: NADPH-dependent thioredoxin reductase
PRX: peroxiredoxin
GPXL: glutathione peroxidase-like protein
GSSG: glutathione disulfide
*E*_GSH_: glutathione redox potential
MSR: methionine sulfoxide reductase
GRX: glutaredoxin
NPR1: nonexpressor of pathogenesis-related genes 1
DHAR: dehydroascorbate reductase
GST: glutathione *S*-transferase
APX: ascorbate peroxidase
Cb: *Chara braunii*
Aa: *Anthoceros agrestis*
Mp: *Marchantia polymorpha*
Pp: *Physcomitrium patens*
Sm: *Selaginella moellendorffii*
Sc: *Salvinia cucullata*
Af: *Azolla filiculoides*
Bd: *Brachypodium distachyon*
At: *Arabidopsis thaliana*
MetSO: methionine sulfoxide
PET: photosynthetic electron transport
AsA: ascorbic acid
GAP: glyceraldehyde-3-phosphate dehydrogenase
MS/MS: tandem mass spectrometry
BioGEE: biotinylated GSH ethyl ester
BioGSSG: biotinylated glutathione disulfide
AMY: alpha-amylase
THI: thiamine thiazole synthase
PGK: phosphoglycerate kinase
FDX: ferredoxin
HGT: horizontal gene transfer
GSNO: *S*-nitrosoglutathione
