# Supplementary figures and images for: Plasticity in plastid redox networks: evolution of glutathione-dependent redox cascades and glutathionylation sites
Source: BMC Plant Biol. 2021 Jul 5;21:322. doi: 10.1186/s12870-021-03087-2 (PMC8256493; doi:10.1186/s12870-021-03087-2)

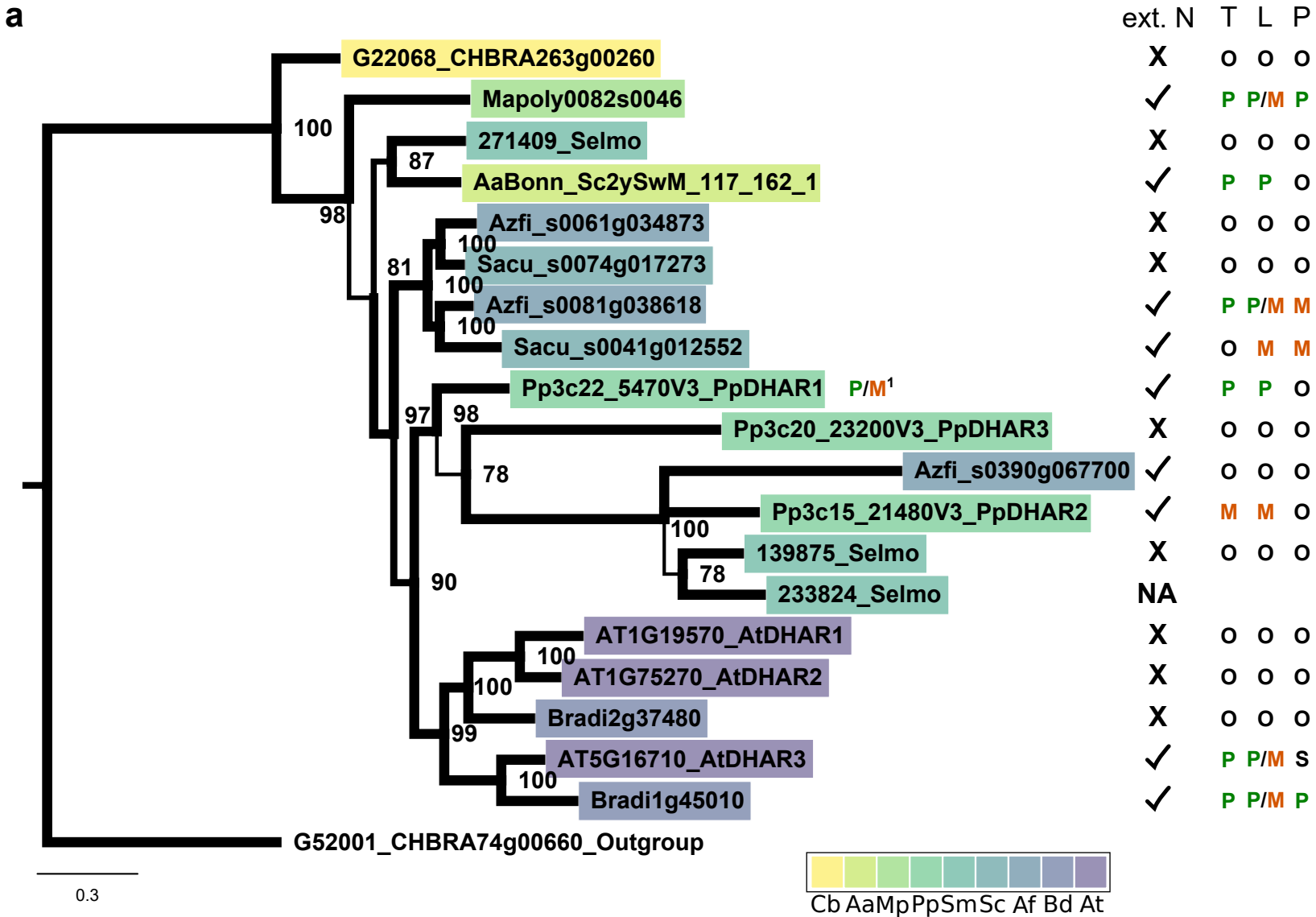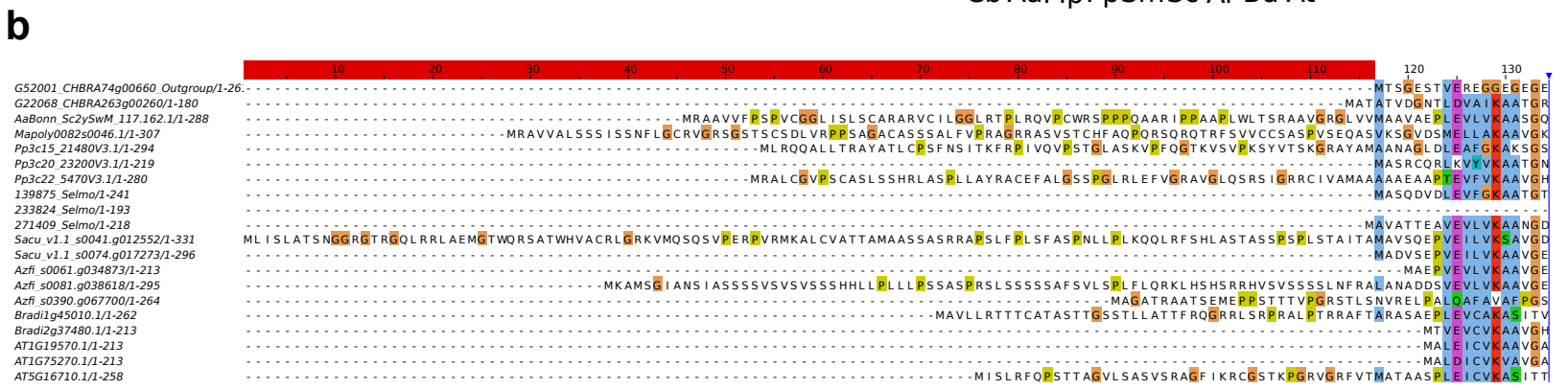

Supplement: Supplementary file 2 — Additional file 2: Fig. S2. Phylogenetic tree of DHAR. (a) Phylogenetic tree of DHAR isoforms (P. patens nomenclature according to Liu et al. (2013) [50]) constructed using MrBayes, node values and line weights depict posterior probabilities (run parameters: mixed protein models, rates = invgamma, number of generations: 2*106, burnin = 20%, split frequencies< 0.01). 1P. patens DHAR1 was identified and quantified in mitochondrial and plastid proteomes [49] and is putatively dual targeted. TargetP2.0 (T) [46], LOCALIZER (L) [47] and PredAlgo (P) [48] predictions (Additional file 1 Table S1) indicate highly variable targeting of the multiple DHAR paralogs (M, mitochondria; P, plastid; O, other; S, secretory). The presence (check mark) or absence (X) of an N-terminal extension (ext. N) in the sequence is indicated; NA: not assessed as sequence potentially incomplete. Gene identifiers are given according to the used gene models for Chara braunii (CHBRA), Anthoceros agrestis strain Bonn (AaBonn), Marchantia polymorpha (Mapoly), Physcomitrium patens (Pp), Selaginella moellendorffii (Selmo), Salvinia cucullata (Sacu), Azolla filiculoides (Azfi), Brachypodium distachyon (Bradi) and Arabidopsis thaliana (At) and are additionally color-coded as in Fig. 1. Colour legend: Cb = Chara braunii; Aa = Anthoceros agrestis; Mp = Marchantia polymorpha; Pp = Physcomitrium patens; Sm = Selaginella moellendorffii; Sc = Salvinia cucullata; Af = Azolla filiculoides; Bd = Brachypodium distachyon; At = Arabidopsis thaliana. (b) N-terminal part of protein alignment (Jalview) showing the presence or absence of N-terminal extensions indicative of putative N-terminal targeting peptides. Colour-scheme: ClustalX. [file 12870_2021_3087_MOESM2_ESM.pdf]

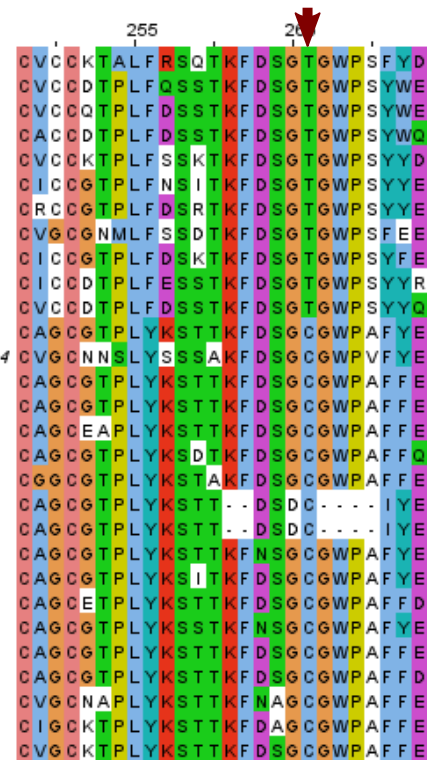

Supplement: Supplementary file 4 — Additional file 4: Fig. S4. Phylogenetic tree of methionine sulfoxide reductases B. (a) Phylogenetic tree of methionine sulfoxide reductase B (MSRB) isoforms constructed using MrBayes, node values and line weights depict posterior probabilities (run parameters: mixed protein models, rates = invgamma, number of generations: 2*106, burnin = 20%, split frequencies< 0.01). TargetP2.0 (T) [46], LOCALIZER (L) [47] and PredAlgo (P) [48] predictions (Additional file 1 Table S1) indicate variable targeting of MSRB1 isoforms to plastids (M, mitochondria; P, plastid; O, other; S, secretory). The presence (check mark) or absence (X) of an N-terminal extension (ext. N) in the sequence is indicated; NA: not assessed as sequence potentially incomplete. Gene identifiers are given according to the used gene models for Chlamydomonas reinhardtii (Cre), Chara braunii (CHBRA), Anthoceros agrestis strain Bonn (AaBonn), Marchantia polymorpha (Mapoly), Physcomitrium patens (Pp), Selaginella moellendorffii (Selmo), Salvinia cucullata (Sacu), Azolla filiculoides (Azfi), Brachypodium distachyon (Bradi) and Arabidopsis thaliana (At) and are additionally color-coded as in Fig. 1. Colour legend: Cb = Chara braunii; Aa = Anthoceros agrestis; Mp = Marchantia polymorpha; Pp = Physcomitrium patens; Sm = Selaginella moellendorffii; Sc = Salvinia cucullata; Af = Azolla filiculoides; Bd = Brachypodium distachyon; At = Arabidopsis thaliana. (b) N-terminal part of protein alignment (Jalview) showing the presence or absence of N-terminal extensions indicative of putative N-terminal targeting peptides. Colour-scheme: ClustalX. (c) In the presence of H2O2, methionine can be oxidised, a modification that can be resolved by MSRB regarding methionine-R-sulfoxide. The GRX-dependent reaction mechanism in atypical (1Cys) MSRB operates via an S-glutathionylation intermediate [53]. The GRX-dependent mechanism generates GSSG that in turn requires GR for reduction. (PDF 156 kb). (d) Conservation of threonine in the r [file 12870_2021_3087_MOESM4_ESM.pdf]

**a**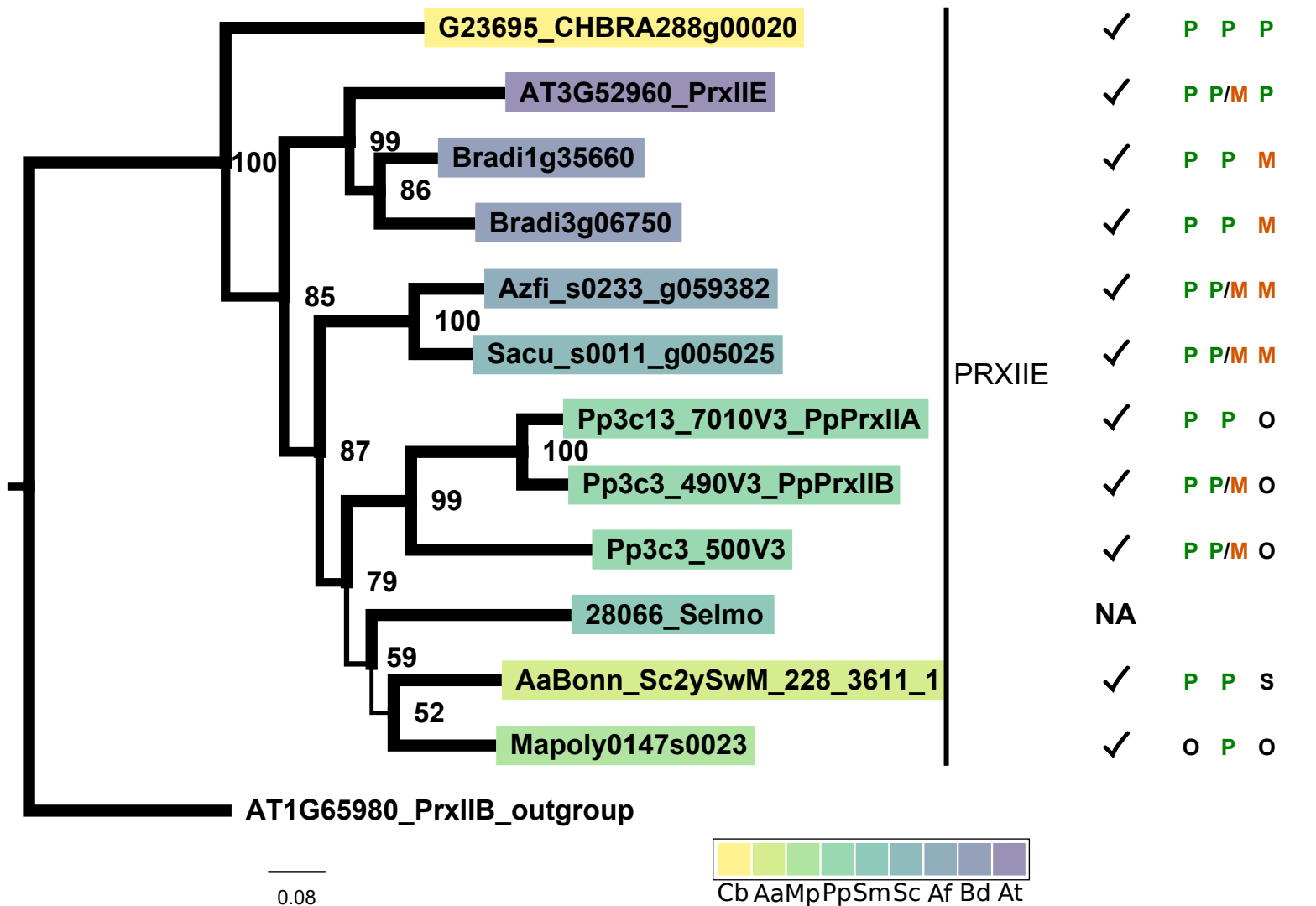**b**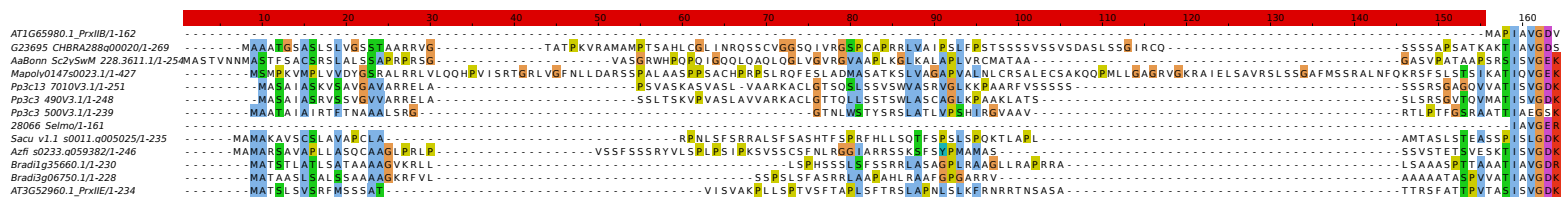

Supplement: Supplementary file 5 — Additional file 5: Fig. S5. Phylogenetic tree of peroxiredoxin II E. (a) Phylogenetic tree of peroxiredoxin IIE (PRXIIE) isoforms constructed using MrBayes, node values and line weights depict posterior probabilities (run parameters: mixed protein models, rates = invgamma, number of generations: 2*106, burnin = 20%, split frequencies< 0.01). TargetP2.0 (T) [46] predictions indicate conserved targeting of PRXIIE isoforms to plastids, while LOCALIZER (L) [47] and PredAlgo (P) [48] predictions vary (M, mitochondria; P, plastid; O, other; S, secretory) (Additional file 1 Table S1). The presence (check mark) or absence (X) of an N-terminal extension (ext. N) in the sequence is indicated; NA: not assessed as sequence potentially incomplete. Gene identifiers are given according to the used gene models for Chara braunii (CHBRA), Anthoceros agrestis strain Bonn (AaBonn), Marchantia polymorpha (Mapoly), Physcomitrium patens (Pp), Selaginella moellendorffii (Selmo), Salvinia cucullata (Sacu), Azolla filiculoides (Azfi), Brachypodium distachyon (Bradi) and Arabidopsis thaliana (At) and are additionally color-coded as in Fig. 1. Colour legend: Cb = Chara braunii; Aa = Anthoceros agrestis; Mp = Marchantia polymorpha; Pp = Physcomitrium patens; Sm = Selaginella moellendorffii; Sc = Salvinia cucullata; Af = Azolla filiculoides; Bd = Brachypodium distachyon; At = Arabidopsis thaliana. (b) N-terminal part of protein alignment (Jalview) showing the presence or absence of N-terminal extensions indicative of putative N-terminal targeting peptides. Colour-scheme: ClustalX. [file 12870_2021_3087_MOESM5_ESM.pdf]

**a**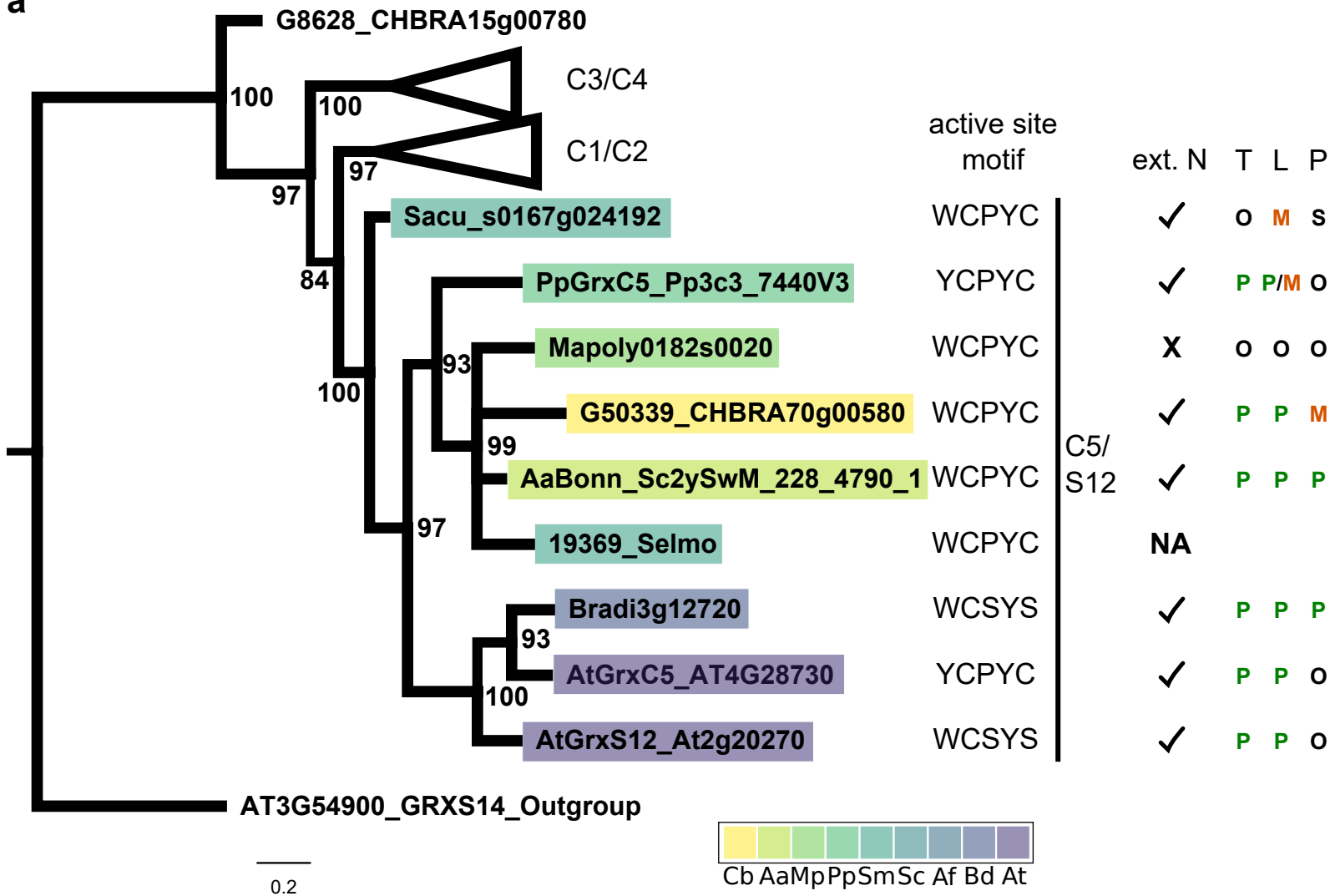**b**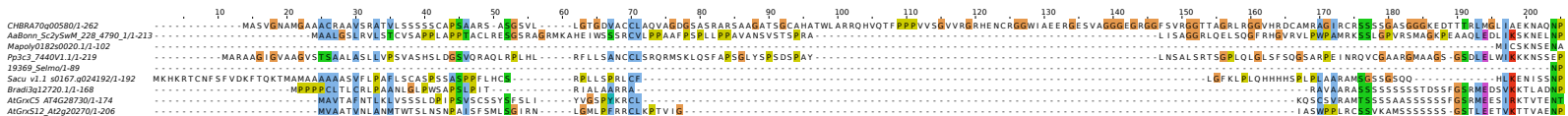

Supplement: Supplementary file 6 — Additional file 6: Fig. S6. Phylogenetic tree of class I GRX. (a) Phylogenetic tree of class I glutaredoxin (GRX) isoforms constructed using MrBayes, node values and line weights depict posterior probabilities; nodes with lower support than 50% are collapsed (run parameters: mixed protein models, rates = invgamma, number of generations: 4*106, burnin = 20%, split frequencies< 0.01). TargetP2.0 (T) [46], LOCALIZER (L) [47] and PredAlgo (P) [48] targeting predictions (Additional file 1 Table S1) are indicated (M, mitochondria; P, plastid; O, other; S, secretory). The presence (check mark) or absence (X) of an N-terminal extension (ext. N) in the sequence is indicated; NA: not assessed as sequence potentially incomplete. Gene identifiers are given according to the used gene models for Chara braunii (CHBRA), Anthoceros agrestis strain Bonn (AaBonn), Marchantia polymorpha (Mapoly), Physcomitrium patens (Pp), Selaginella moellendorffii (Selmo), Salvinia cucullata (Sacu), Azolla filiculoides (Azfi), Brachypodium distachyon (Bradi) and Arabidopsis thaliana (At) and are additionally color-coded as in Fig. 1. Colour legend: Cb = Chara braunii; Aa = Anthoceros agrestis; Mp = Marchantia polymorpha; Pp = Physcomitrium patens; Sm = Selaginella moellendorffii; Sc = Salvinia cucullata; Af = Azolla filiculoides; Bd = Brachypodium distachyon; At = Arabidopsis thaliana. (b) N-terminal part of protein alignment (Jalview) showing the presence or absence of N-terminal extensions indicative of putative N-terminal targeting peptides. Colour-scheme: ClustalX. [file 12870_2021_3087_MOESM6_ESM.pdf]

**a**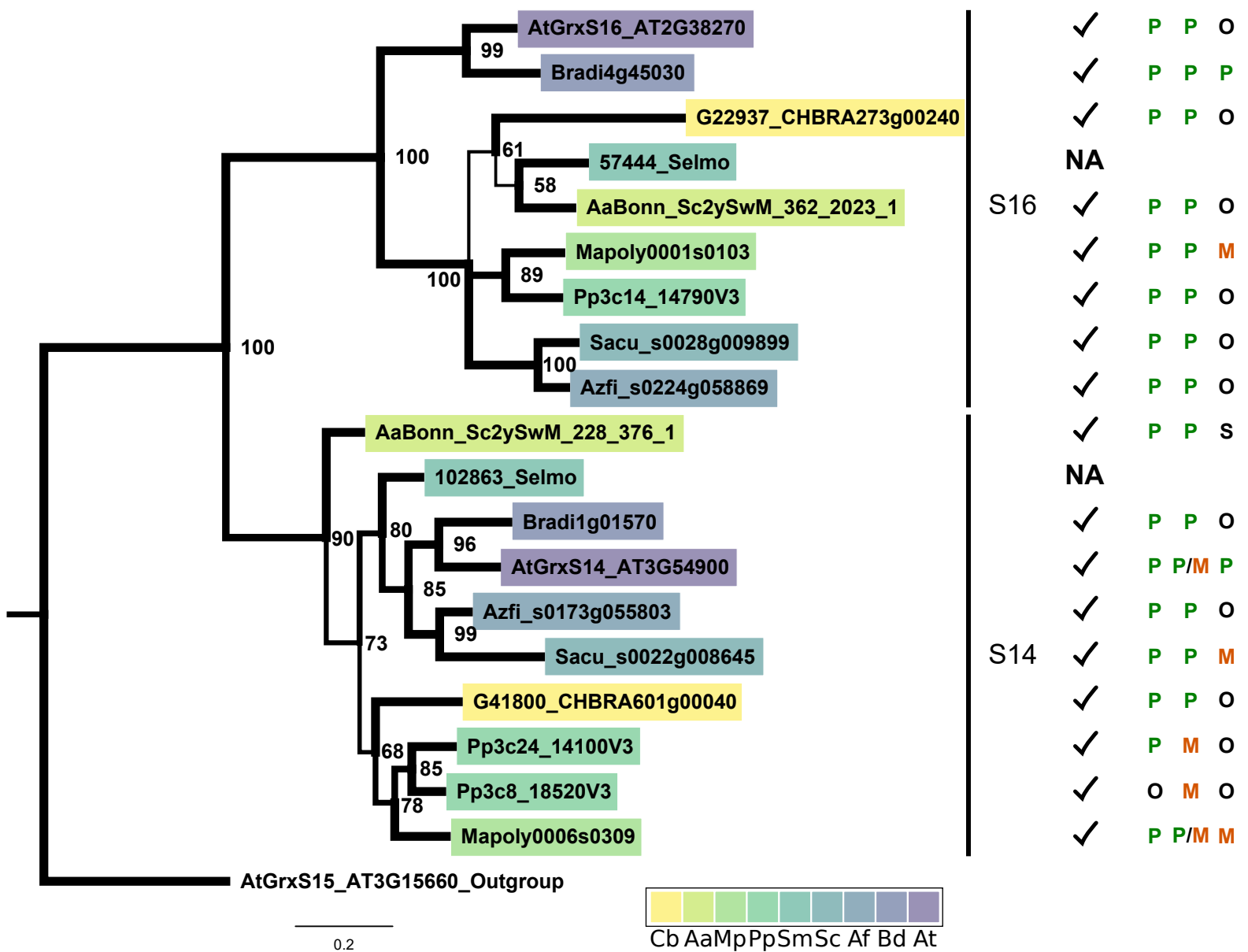**b** S14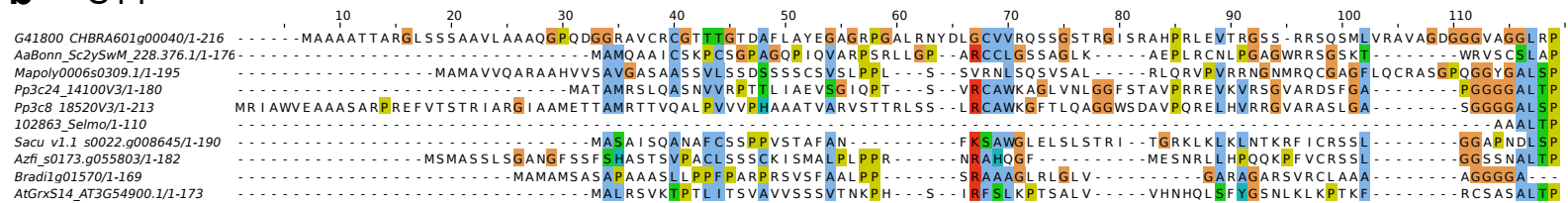**c** S16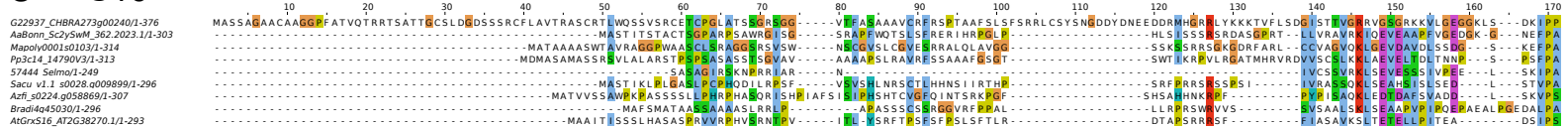

Supplement: Supplementary file 7 — Additional file 7: Fig. S7. Phylogenetic tree of plastid-targeted class II GRX. (a) Phylogenetic tree of the plastid class II glutaredoxin (GRX) S14 and S16 isoforms constructed using MrBayes, node values and line weights depict posterior probabilities; nodes with lower support than 50% are collapsed (run parameters: mixed protein models, rates = invgamma, number of generations: 2*106, burnin = 20%, split frequencies< 0.01). TargetP2.0 (T) [46] predictions indicate conserved targeting of GRXS14 and GRXS16 isoforms to plastids, while LOCALIZER (L) [47] and PredAlgo (P) [48] predictions vary (M, mitochondria; P, plastid; O, other; S, secretory) (Additional file 1 Table S1). The presence (check mark) or absence (X) of an N-terminal extension (ext. N) in the sequence is indicated; NA: not assessed as sequence potentially incomplete. Gene identifiers are given according to the used gene models for Chara braunii (CHBRA), Anthoceros agrestis strain Bonn (AaBonn), Marchantia polymorpha (Mapoly), Physcomitrium patens (Pp), Selaginella moellendorffii (Selmo), Salvinia cucullata (Sacu), Azolla filiculoides (Azfi), Brachypodium distachyon (Bradi) and Arabidopsis thaliana (At) and are additionally color-coded as in Fig. 1. Colour legend: Cb = Chara braunii; Aa = Anthoceros agrestis; Mp = Marchantia polymorpha; Pp = Physcomitrium patens; Sm = Selaginella moellendorffii; Sc = Salvinia cucullata; Af = Azolla filiculoides; Bd = Brachypodium distachyon; At = Arabidopsis thaliana. (b) N-terminal part of GRXS14 protein alignment (Jalview) showing the presence or absence of N-terminal extensions indicative of putative N-terminal targeting peptides. Colour-scheme: ClustalX. (c) N-terminal part of GRXS16 protein alignment (Jalview) showing the presence or absence of N-terminal extensions indicative of putative N-terminal targeting peptides. Colour-scheme: ClustalX. [file 12870_2021_3087_MOESM7_ESM.pdf]
