# Supplementary material for: Plasticity in plastid redox networks: evolution of glutathione-dependent redox cascades and glutathionylation sites
Source: BMC Plant Biol. 2021 Jul 5;21:322. doi: 10.1186/s12870-021-03087-2 (PMC8256493; doi:10.1186/s12870-021-03087-2)
Supplement: Supplementary file 3 — Additional file 3: Fig. S3. Phylogenetic tree of lambda and iota-type GSTs. (a) Phylogenetic tree of lambda- and iota-type glutathione S-transferase isoforms (P. patens nomenclature according to Liu et al. (2013) [50]) constructed using MrBayes, node values and line weights depict posterior probabilities (run parameters: mixed protein models, rates = invgamma, number of generations: 2*106, burnin = 20%, split frequencies< 0.01). TargetP2.0 (T) [46], LOCALIZER (L) [47] and PredAlgo (P) [48] predictions (Additional file 1 Table S1) indicate variable targeting of GSTL and GSTI isoforms to plastids (M, mitochondria; P, plastid; O, other; S, secretory). The presence (check mark) or absence (X) of an N-terminal extension (ext. N) in the sequence is indicated; NA: not assessed as sequence potentially incomplete. Gene identifiers are given according to the used gene models for Chlamydomonas reinhardtii (Cre), Chara braunii (CHBRA), Anthoceros agrestis strain Bonn (AaBonn), Marchantia polymorpha (Mapoly), Physcomitrium patens (Pp), Selaginella moellendorffii (Selmo), Salvinia cucullata (Sacu), Azolla filiculoides (Azfi), Brachypodium distachyon (Bradi) and Arabidopsis thaliana (At) and are additionally color-coded as in Fig. 1. Colour legend: Cb = Chara braunii; Aa = Anthoceros agrestis; Mp = Marchantia polymorpha; Pp = Physcomitrium patens; Sm = Selaginella moellendorffii; Sc = Salvinia cucullata; Af = Azolla filiculoides; Bd = Brachypodium distachyon; At = Arabidopsis thaliana. (b) N-terminal part of protein alignment (Jalview) showing the presence or absence of N-terminal extensions indicative of putative N-terminal targeting peptides. Colour-scheme: ClustalX. [file 12870_2021_3087_MOESM3_ESM.pdf]

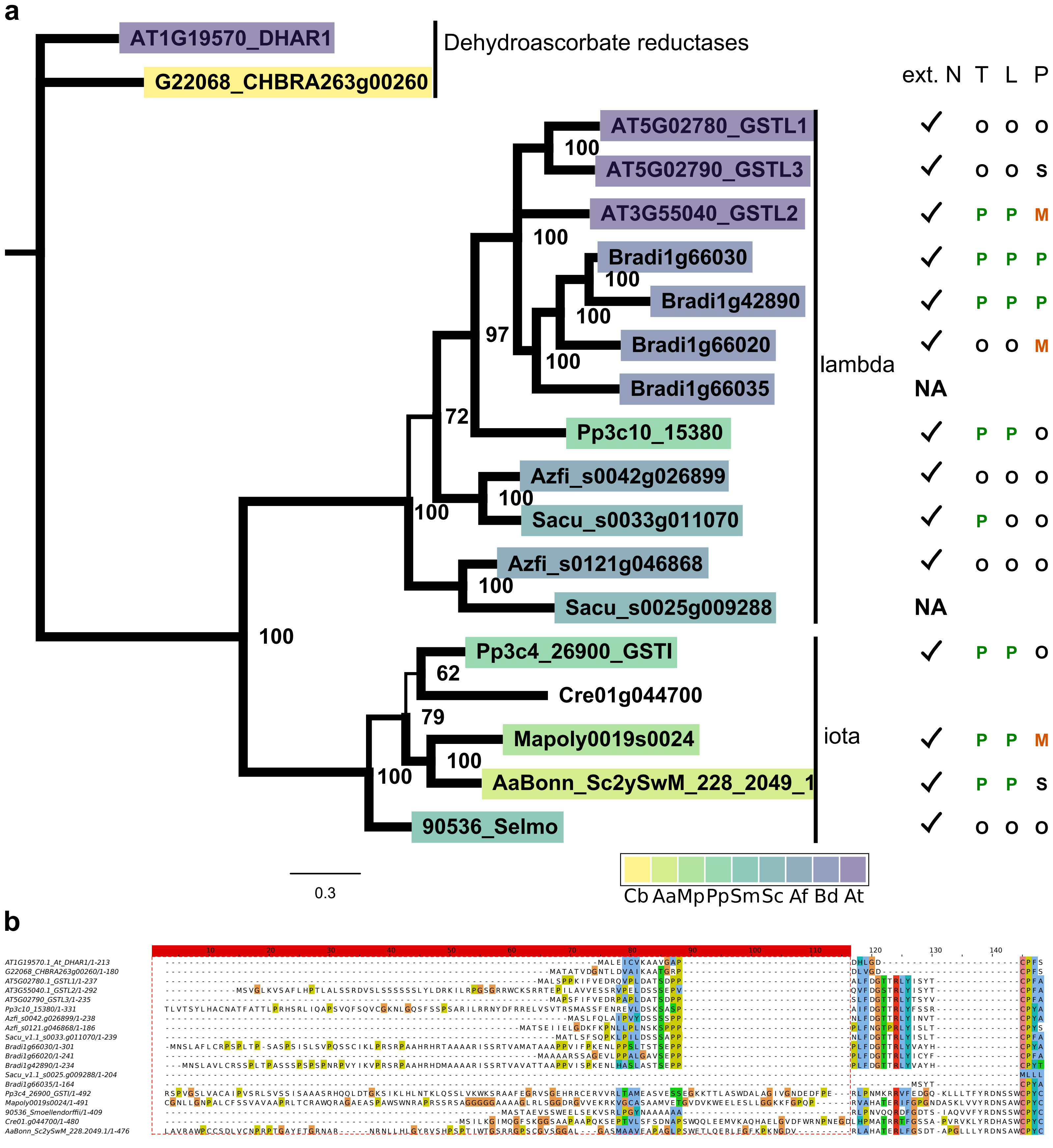
a
AT1G19570_DHAR1
G22068_CHBRA263g00260
Dehydroascorbate reductases
ext. N T L P
AT5G02780_GSTL1
AT5G02790_GSTL3
AT3G55040_GSTL2
Bradi1g66030
Bradi1g42890
Bradi1g66020
Bradi1g66035
Pp3c10_15380
Azfi_s0042g026899
Sacu_s0033g011070
Azfi_s0121g046868
Sacu_s0025g009288
lambda
Pp3c4_26900_GSTI
Cre01g044700
Mapoly0019s0024
AaBonn_Sc2ySwM_228_2049_1
90536_Selmo
iota
NA
100
97
72
62
79
0.3
Cb Aa Mp Pp Sm Sc Af Bd At
b
AT1G19570.1_At_DHAR1/1-213
G22068_CHBRA263g00260/1-180
AT5G02780.1_GSTL1/1-237
AT3G55040.1_GSTL2/1-292
AT5G02790_GSTL3/1-235
Pp3c10_15380/1-331
Azfi_s0042.g026899/1-238
Azfi_s0121.g046868/1-186
Sacu_v1.1_s0033.g011070/1-239
Bradi1g66030/1-301
Bradi1g66020/1-241
Bradi1g42890/1-234
Sacu_v1.1_s0025.g009288/1-204
Bradi1g66035/1-164
Pp3c4_26900_GSTI/1-492
Mapoly0019s0024/1-491
90536_Smoellendorffii/1-409
Cre01.g044700/1-480
AaBonn_Sc2ySwM_228.2049.1/1-476
