# Supplementary material for: Plasticity in plastid redox networks: evolution of glutathione-dependent redox cascades and glutathionylation sites
Source: BMC Plant Biol. 2021 Jul 5;21:322. doi: 10.1186/s12870-021-03087-2 (PMC8256493; doi:10.1186/s12870-021-03087-2)
Supplement: Supplementary file 10 — Additional file 10 Word-file containing all alignments used to assess conservation of known S-glutathionylation sites on plastid proteins in fasta format. [file 12870_2021_3087_MOESM10_ESM.docx]

**Additional File 10**

**Protein sequence alignments used to determine the evolutionary conservation of known glutathionylation sites on plastid proteins in fasta format**

Alignments were generated in Jalview 2.11.1.0 using the Muscle algorithm.

Alignments were exported to Word using Mview (EMBL-EBI search and sequence analysis tools APIs) <https://www.ebi.ac.uk/Tools/msa/mview/>

A multi-fasta format is additionally provided for each alignment.

**2-Cys Peroxiredoxin**

cov pid  **1** **[ . . . . : . . .** **80**

1 Selmo_36651/1-114 100.0% 100.0%  **--------------------------------------------------------------------------------**

2 Selmo_420519/1-272 100.0% 35.3%  **--------------------------MATAGAAAFAATGHVVGGGATSSSSSAASAAPCRSSLFKAFDARLGASKISHLR**

3 Selmo_96390/1-190 98.2% 37.4%  **--------------------------------------------------------------------------------**

4 AT3G11630/1-266 100.0% 32.0%  **-------------------------------MASVASSTTLISSPSSRVFPAKSSLSSPSVSFLRTL-----SSPSASAS**

5 AT5G06290/1-273 100.0% 31.5%  **----------------------------MSMASIASSSSTTLLSSSRVLLPSKSSLLSPTVSFPRIIPSSSASSSSLCSG**

6 Bradi3g45140/1-260 100.0% 30.8%  **-------------------------------MACSFATSTVVSSTP-TPKPLATALTPQCLSISRAP-------------**

7 Bradi5g09650/1-260 100.0% 31.9%  **-------------------------------MACAFSASTVSPAAA-LVASPKPTAAPQFLSFPRAF-------------**

8 Cre02.g114600/1-198 100.0% 28.8%  **--------------------------------------------------------------------------------**

9 Cre06.g257601/1-235 100.0% 30.2%  **----------------------------MAALQSASRSSAVAFSRQARVAPRVAASVA----------------------**

10 Cre17.g743897/1-387 96.5% 12.5%  **---------------MSRHLRRASTLLARLAAHGANKEPLVASMAGRALAQSVVQMPMWQIVQPRGFASSSSSDDEFFEE**

11 Mapoly0014s0048/1-303 100.0% 24.8%  **-----------------------------MATACAARSAVALPLASVAATTASSSTAAPSVAIPRSFDGLNKSFGARLAT**

12 Mapoly0014s0048/1-223 100.0% 33.6%  **------------------------------------------MAKPPAPSNEIRDLCVRLSSL-----------------**

13 Mapoly0014s0048/1-275 100.0% 27.3%  **-----------------------------MATACAARSAVALPLASVAATTASSSTAAPSVAIPRSFDGLNKSFGARLAT**

14 Pp3c1_21200/1-282 100.0% 28.0%  **-------------------------MACAAAASGVAASSVVIPRAPSAACSSSASGTSTSLAVPKSFCGLGKSFGARVAT**

15 Pp3c2_16260/1-282 100.0% 28.0%  **-------------------------MACAAAASGVTLPAGNIAHARPAAFSSSASGASSSLSVRKSFSGLSKSFAARLAA**

16 Azfi_s2786.g113096/1-270 99.1% 29.0%  **----------------------------MAMAACAASSAAILSSASASPSSSPLVASVPRSHLRSSFVSTVSKLRSSSSP**

17 Azfi_s0101.g044406/1-270 100.0% 29.3%  **----------------------------MAMAACAASSAAILSSASASPSSSPLVASVPRSHLRSSFVSTVSKLRSSSSP**

18 Azfi_s0006.g009576/1-294 89.5% 20.6%  **IFFLFLFLSLSLSLSLSLIRLVSVSKPRLPSCLCFHHGYLPPSPFLSSPSSSSSSSSIVTASAPRLLYPRVRSLWSSSFS**

19 Sacu_v1.1_s0011.g005300/1-273 100.0% 28.9%  **---------------------------MAISASSIITASCFNPPSLASPYSSSSSILAPRVSRDVDLRSGFISPVSQFRP**

20 Sacu_v1.1_s0003.g001962/1-685 99.1% 12.1%  **-------------------------MASTCSPAAVSSFSTPAAISSPSSSSSSIAFPPSRPALLKSP-------------**

21 Sacu_v1.1_s0025.g009359/1-292 100.0% 25.7%  **-----------------------MLQTVQIAVPCQMQAFAPPSPRPTYCLSSSATAILSSARLPHCLLQKKQPHRRGTKK**

22 CHBRA41g00500/1-284 100.0% 28.5%  **-------------------------MAATTLHASLSTAAVGLSHNGGIGTQCQRRSATSALPAVKLTTYEGLGKASRLTA**

23 AagrBONN_Sc2ySwM_368.1690.4/1-204 77.2% 1.7%  **MAMAVAAATAAPALSPAASAAVSSASSSASTSLVAVPRRFSGLSTSFEARLTAGVPVQHKGRSASR----------QAAS**

24 AagrBONN_/1-213 100.0% 34.7%  **----------------------------------------MKGVSKYMLLQGDQPQPPPKLPI-----------------**

cov pid  **81**  **. 1 . . . . : .** **160**

1 Selmo_36651/1-114 100.0% 100.0%  **---------------------------------------DVPLVGNKGLDFEAESVFNQEFIK--VKLSDYF----YVML**

2 Selmo_420519/1-272 100.0% 35.3%  **RPAARIQPRRAFR------------------SLAASASLDVPLVGNKAPDFEAESVFDQEFIK--VKLSDYLGK-KYVVL**

3 Selmo_96390/1-190 98.2% 37.4%  **-----------------------------------------PLVGSMAPNFEAEAVHHQEFIK--VKLSNYIGK-KFVVL**

4 AT3G11630/1-266 100.0% 32.0%  **LRSGFARRSSLSS-------------TSRRSFAVKAQADDLPLVGNKAPDFEAEAVFDQEFIK--VKLSDYIGK-KYVIL**

5 AT5G06290/1-273 100.0% 31.5%  **FSSLGSLTTNRSA--------------SRRNFAVKAQADDLPLVGNKAPDFEAEAVFDQEFIK--VKLSEYIGK-KYVIL**

6 Bradi3g45140/1-260 100.0% 30.8%  **-L-ATVRPLRLAA--------ASRSARTSGFVARAGGVDELPLVGNKAPDFDAEAVFDQEFIN--VKLSDYIGK-KYVIL**

7 Bradi5g09650/1-260 100.0% 31.9%  **-VGGAARPSRLAA---------SRTARARNFVARAGGEDSLPLVGNKAPDFDAEAVFDQEFIN--VKLSDYIGK-KYVIL**

8 Cre02.g114600/1-198 100.0% 28.8%  **---------------------------------------MVAKIGAPAPKFKAQAVVNGEIKE--ISLDDYKGK--YVVL**

9 Cre06.g257601/1-235 100.0% 30.2%  **----------------------------RRSLVVRASHAEKPLVGSVAPDFKAQAVFDQEFQE--ITLSKYRGK--YVVL**

10 Cre17.g743897/1-387 96.5% 12.5%  **DQMPIIYPPSQVR------------------------------VGERAPNFSAPAVVDGEITK--ISLSDYKGK--YVCL**

11 Mapoly0014s0048/1-303 100.0% 24.8%  **RSTSAFCKPVTGV-----------SLSQSARRFVVSASKEAPLVGNVAPDFEAEAVFDQEFVK--IKLSEYIGK-KYVVL**

12 Mapoly0014s0048/1-223 100.0% 33.6%  **---------------------------------SGSQSKEAPLVGNVAPDFEAEAVFDQEFVK--IKLSEYIGK-KYVVL**

13 Mapoly0014s0048/1-275 100.0% 27.3%  **RSTSAFCKPVTGV-----------SLSQSARRFVVSASKEAPLVGNVAPDFEAEAVFDQEFVK--IKLSEYIGK-KYVVL**

14 Pp3c1_21200/1-282 100.0% 28.0%  **ANVAVAKAGAVAG--------AKAASQKRGVARASAEDDYAPLVGNVAPDFEAEAVFDQEFIK--IKLSEYIGK-KYVVL**

15 Pp3c2_16260/1-282 100.0% 28.0%  **TNAPTGKAASAAG--------VKIASQKRGVITASAQDSYEPLIGNVAPDFEAEAVFDQEFIK--VKLSEYIGK-KYVVL**

16 Azfi_s2786.g113096/1-270 99.1% 29.0%  **SPASFSSLSCRAS-----------------PRPLVVRAGEIPLVGNVAPDFEAEAVFDQEFTK--IKLSDYRGK-KYVVL**

17 Azfi_s0101.g044406/1-270 100.0% 29.3%  **SPASFSSLSCRAS-----------------PRPLVVRAGEIPLVGNVAPDFEAEAVFDQEFTK--IKLSDYRGK-KYVVL**

18 Azfi_s0006.g009576/1-294 89.5% 20.6%  **PPLPGLRQRPVSS--------SSLCFKDSSRSSSSSSS-----SSSSSPAFVVRA----------IKLSDYRGK-KYVVL**

19 Sacu_v1.1_s0011.g005300/1-273 100.0% 28.9%  **LKRPPLCRQNSSR-------------------SLVARAGELPLVGNVAPDFEAEAVFDQEFVN--IKLSDYRGK-KYVVL**

20 Sacu_v1.1_s0003.g001962/1-685 99.1% 12.1%  **FLPRLSHLRPLSS--------SARTSPSSRPLVVRAGE--LPLVGNVAPDFEAEAVFDQEFINVLVKLSDYRGK-KYVVL**

21 Sacu_v1.1_s0025.g009359/1-292 100.0% 25.7%  **NRYSPRNHRNRSSFPFPSYFDDSSSIFSFTPMSASNAISENTLVGNAAPDFEAEAVLNREFVK--VKLSDYIGK-SYVIL**

22 CHBRA41g00500/1-284 100.0% 28.5%  **DVVPRVSTRKNLK-------CGAVSSPDSSSKTLDIRAHCQPLVGNKAPDFEAKAVFDQEFIE--VKLSDYQAQGKYVVL**

23 AagrBONN_Sc2ySwM_368.1690.4/1-204 77.2% 1.7%  **AGRRTVVSAYEAEFPLVGNVAPDFEAEAVFDQEFVK--IKLSEYIGK-KYVVLFFYPLDFTFVCPTEITAFSDRYSEFEK**

24 AagrBONN_/1-213 100.0% 34.7%  **---------------------------------------HDPLVGHIAPDFEAEAVFEQEFVK--IKLSSYFGK-KFVVL**

cov pid **161**  **. . . 2 . . . .** **240**

1 Selmo_36651/1-114 100.0% 100.0%  **FFYPLDFMIICPIEIASFCDRYSEFEKINTEVLKVSVDSVFSHLSWVQTERKLGGLGNLHYPLVSDISKSISKAYNVHI-**

2 Selmo_420519/1-272 100.0% 35.3%  **FFYPLDFTFVCPTEITAFSDRYSEFEKINTEVLGVSVDSVFSHLAWVQTERKSGGLGDLRYPLVSDISKSISKAYNVLIP**

3 Selmo_96390/1-190 98.2% 37.4%  **FFYPLNFTFVCPTELTAFSDRYGEFQKLDTEVLAVSSDSVFSHLAWIQTDRKSGGLGELNYPVVSDLTKAITKAYRVLLP**

4 AT3G11630/1-266 100.0% 32.0%  **FFYPLDFTFVCPTEITAFSDRHSEFEKLNTEVLGVSVDSVFSHLAWVQTDRKSGGLGDLNYPLISDVTKSISKSFGVLIH**

5 AT5G06290/1-273 100.0% 31.5%  **FFYPLDFTFVCPTEITAFSDRYEEFEKLNTEVLGVSVDSVFSHLAWVQTDRKSGGLGDLNYPLVSDITKSISKSFGVLIP**

6 Bradi3g45140/1-260 100.0% 30.8%  **FFYPLDFTFVCPTEITAFSDRHDEFEKINTQVLGVSIDSVFSHLAWVQTDRKSGGLGDLKYPLISDVTKSISKSFGVLIP**

7 Bradi5g09650/1-260 100.0% 31.9%  **FFYPLDFTFVCPTEITAFSDRHEEFEKINTEILGVSVDSVFSHLAWVQTERKSGGLGDLKYPLVSDVTKSISKSFGVLIP**

8 Cre02.g114600/1-198 100.0% 28.8%  **FFYPLDFTFVCPTEIVAFSDRVEEFRAINTEVIGASIDSQFTHLAFSNTPRTKGGLGGCKYPLVADLTKQIAKDYGVLIE**

9 Cre06.g257601/1-235 100.0% 30.2%  **FFYPLDFTFVCPTEITAFSDRYKEFKDINTEVLGVSVDSQFTHLAWIQTDRKEGGLGDLAYPLVADLKKEISKAYGVLTE**

10 Cre17.g743897/1-387 96.5% 12.5%  **FFYPKDFTFVCPTEIIAFSDRAKEFAAANCQLIAASTDTEETHLAWIRTPRNRGGLGYMQIPILADTTKDISARYGVLIE**

11 Mapoly0014s0048/1-303 100.0% 24.8%  **FFYPLDFTFVCPTEITAFSDKHAEFEKLNTEVIGVSTDSVFSHLAWIQTDRKAGGLGDLNYPLVSDLTKKISKDFGVLIP**

12 Mapoly0014s0048/1-223 100.0% 33.6%  **FFYPLDFTFVCPTEITAFSDKHAEFEKLNTEVIGVSTDSVFSHLAWIQTDRKAGGLGDLNYPLVSDLTKKISKDFGVLIP**

13 Mapoly0014s0048/1-275 100.0% 27.3%  **FFYPLDFTFVCPTEITAFSDKHAEFEKLNTEVIGVSTDSVFSHLAWIQTDRKAGGLGDLNYPLVSDLTKKISKDFGVLIP**

14 Pp3c1_21200/1-282 100.0% 28.0%  **FFYPLDFTFVCPTEITAFSDRYSEFEKLDTEVIGVSIDSVFSHLAWVQTDRKSGGLGELSYPIVSDITKKISKSFGVLIP**

15 Pp3c2_16260/1-282 100.0% 28.0%  **FFYPLDFTFVCPTEITAFSDRYSEFEKLSTEVIGVSTDSVFSHLAWIQTDRKAGGLGDLHYPIVSDITKKISRSFNVLIP**

16 Azfi_s2786.g113096/1-270 99.1% 29.0%  **FFYPLDFTFVCPTEITAFSDRYEEFEKVNTEVIGVSVDSVFSHLAWIQTDRKSGGLGDLKYPLVADLTKTVAKA-RGLFI**

17 Azfi_s0101.g044406/1-270 100.0% 29.3%  **FFYPLDFTFVCPTEITAFSDRYEEFEKVNTEVIGVSVDSVFSHLAWIQTDRKSGGLGDLKYPLVADLTKTVAKAYNVLIP**

18 Azfi_s0006.g009576/1-294 89.5% 20.6%  **FFYPLDFTFVCPTEITAFSDRYAEFEELNTEVIGISIDSVFSHLAWIQTDRKSGGLGDLKYPLVADLTKSISKSYNVLIP**

19 Sacu_v1.1_s0011.g005300/1-273 100.0% 28.9%  **FFYPLDFTFVCPTEITAFSDRHSEFEKLNTEVIGVSIDSVFSHLAWIQTDRKLGGLGDLKYPLVADLTKTISKAFNVLIP**

20 Sacu_v1.1_s0003.g001962/1-685 99.1% 12.1%  **FFYPLDFTFVCPTEITAFSDRYEEFEKLNTEVLGVSIDSVFSHLAWVQTDRKSGGLGDLKYPLVADLTKSISKAFNVLIP**

21 Sacu_v1.1_s0025.g009359/1-292 100.0% 25.7%  **FFYPLDFTFVCPTEITAFSDCYSEFEKLETKIIGISTDSVFSHLAWVQVDRKEGGLGDLQYPLVSDITKGISRNYNVLIR**

22 CHBRA41g00500/1-284 100.0% 28.5%  **FFYPLDFTFVCPTEITAFSDRYAEFAALNTEVLGVSVDSVFSHLAWIQTERKSGGLGDLSYPLVSDITKDIARQYNVLIE**

23 AagrBONN_Sc2ySwM_368.1690.4/1-204 77.2% 1.7%  **LNTEVIGVSTDSVFSHLAWIQTDRKSGGLGDLLFPLVSDLTKCISKKYQVLIP---D-----------------------**

24 AagrBONN_/1-213 100.0% 34.7%  **FFYPLDFTFVCPTEITQFSDRYDEFQKLNTEVIAVSTDSVFSHLAWVQTDRKAGGLGDLKYPIASDLTKVISRRYGVLIA**

cov pid **241**  **: . . . . 3 . .** **320**

1 Selmo_36651/1-114 100.0% 100.0%  **--------------------------------------------------------------------------------**

2 Selmo_420519/1-272 100.0% 35.3%  **---D----------------------------QGIAL--RGLFIIDKEGIIQHATVNNLAIGRSVDETLRTLQAVQYVQD**

3 Selmo_96390/1-190 98.2% 37.4%  **---D----------------------------QGISL--RGLFIIDKEGIVQHATINNLAVGRNVDEALRLVQAVQYVKV**

4 AT3G11630/1-266 100.0% 32.0%  **---D----------------------------QGIAL--RGLFIIDKEGVIQHSTINNLGIGRSVDETMRTLQALQYIQE**

5 AT5G06290/1-273 100.0% 31.5%  **---D----------------------------QGIAL--RGLFIIDKEGVIQHSTINNLGIGRSVDETMRTLQALQYVQE**

6 Bradi3g45140/1-260 100.0% 30.8%  **---H----------------------------QGIAL--RGLFIIDKEGVIQHSTINNLAIGRSVDETMRTLQALQYVQE**

7 Bradi5g09650/1-260 100.0% 31.9%  **---D----------------------------QGIAL--RGLFIIDKEGVIQHSTINNLGIGRSVDETLRTLQALQYVQE**

8 Cre02.g114600/1-198 100.0% 28.8%  **DGPD----------------------------AGVTL--RGLFIISPTGVLRQITINDLPVGRSVDETLRLVKAFQFTDE**

9 Cre06.g257601/1-235 100.0% 30.2%  **---D-----------------------------GISL--RGLFIIDKEGVVQHATINNLAFGRSVDETKRVLQAIQYVQS**

10 Cre17.g743897/1-387 96.5% 12.5%  **---K----------------------------LGVAL--RGLFIINPQGVVQHVTINDLPIGRSVDEALRTLQAIQYHAE**

11 Mapoly0014s0048/1-303 100.0% 24.8%  **---DQRMWEIQMKDQNSRKSVEKTVTAHSGPSQGIAL--RGLFIIDKEGVIQHATINNLAIGRSVEETLRTLQAVQYVQE**

12 Mapoly0014s0048/1-223 100.0% 33.6%  **---D----------------------------QGIAL--RGLFIIDKEGVIQHATINNLAIGRSVEETLRTLQAVQYVQE**

13 Mapoly0014s0048/1-275 100.0% 27.3%  **---D----------------------------QGIAL--RGLFIIDKEGVIQHATINNLAIGRSVEETLRTLQAVQYVQE**

14 Pp3c1_21200/1-282 100.0% 28.0%  **---D----------------------------QGIAL--RGLFIIDKQGVIQHATINNLGIGRSVDETLRTLQAVQYVQD**

15 Pp3c2_16260/1-282 100.0% 28.0%  **---E----------------------------QGIAL--RGLFIIDKQGVIQHATVNNLGIGRSVDETLRTLQAVQYVQD**

16 Azfi_s2786.g113096/1-270 99.1% 29.0%  **IDKEGVIQHATINNLGIGRSVDETLRTLQAVQYVQDNPDEVCPAGWKPGEK-----------------------------**

17 Azfi_s0101.g044406/1-270 100.0% 29.3%  **---D----------------------------QGVAL--RGLFIIDKEGVIQHATINNLGIGRSVDETLRTLQAVQYVQD**

18 Azfi_s0006.g009576/1-294 89.5% 20.6%  **---G----------------------------QGVAL--RGLFILDKEGVIQHATINNLAIGRSVEETLRTLQAVQYVQD**

19 Sacu_v1.1_s0011.g005300/1-273 100.0% 28.9%  **---E------------------------QAGRNGIAL--RGLFIIDKDGVIQHATINNLAIGRSVDETLRTLQAVQYVQE**

20 Sacu_v1.1_s0003.g001962/1-685 99.1% 12.1%  **---S----------------------------QGVAL--RGLFIIDKEGVIQHATINNLAIGRSVDETLRTLQAVQYVQD**

21 Sacu_v1.1_s0025.g009359/1-292 100.0% 25.7%  **---D----------------------------QGVAL--RGLFIIDKSGIIQYATINNLRFGRSVDETLRILQAIQYVQE**

22 CHBRA41g00500/1-284 100.0% 28.5%  **---E----------------------------EGVAL--RGLFIIDTEGVIQHATINNLAIGRSVDETLRTLQAVQYVQE**

23 AagrBONN_Sc2ySwM_368.1690.4/1-204 77.2% 1.7%  **-----Q-VS-----------------------------------------------------------------------**

24 AagrBONN_/1-213 100.0% 34.7%  **---D----------------------------QGVAL--RGLFIIDKQGVIQHATINNLSFGRSVDETIRTLHAVQHVQQ**

cov pid **321**  **. . : . . . . 4** **400**

1 Selmo_36651/1-114 100.0% 100.0%  **--------------------------------------------------------------------------------**

2 Selmo_420519/1-272 100.0% 35.3%  **NPDEVCPAGWKPGDK-----------------------------------------------------------------**

3 Selmo_96390/1-190 98.2% 37.4%  **HPDELCPAGWQPGEH-----------------------------------------------------------------**

4 AT3G11630/1-266 100.0% 32.0%  **NPDEVCPAGWKPGEK-----------------------------------------------------------------**

5 AT5G06290/1-273 100.0% 31.5%  **NPDEVCPAGWKPGEK-----------------------------------------------------------------**

6 Bradi3g45140/1-260 100.0% 30.8%  **NPDEVCPAGWKPGEK-----------------------------------------------------------------**

7 Bradi5g09650/1-260 100.0% 31.9%  **NPDEVCPAGWKPGEK-----------------------------------------------------------------**

8 Cre02.g114600/1-198 100.0% 28.8%  **H-GEVCPANWNPGAK-----------------------------------------------------------------**

9 Cre06.g257601/1-235 100.0% 30.2%  **NPDEVCPAGWKPGDK-----------------------------------------------------------------**

10 Cre17.g743897/1-387 96.5% 12.5%  **H-GEVCPANWKPGSK-----------------------------------------------------------------**

11 Mapoly0014s0048/1-303 100.0% 24.8%  **NPDEVCPAGWKPGEK-----------------------------------------------------------------**

12 Mapoly0014s0048/1-223 100.0% 33.6%  **NPDEVCPAGWKPGEK-----------------------------------------------------------------**

13 Mapoly0014s0048/1-275 100.0% 27.3%  **NPDEVCPAGWKPGEK-----------------------------------------------------------------**

14 Pp3c1_21200/1-282 100.0% 28.0%  **NPDEVCPAGWKPGEK-----------------------------------------------------------------**

15 Pp3c2_16260/1-282 100.0% 28.0%  **NPDEVCPAGWKPGEK-----------------------------------------------------------------**

16 Azfi_s2786.g113096/1-270 99.1% 29.0%  **--------------------------------------------------------------------------------**

17 Azfi_s0101.g044406/1-270 100.0% 29.3%  **NPDEVCPAGWKPGEK-----------------------------------------------------------------**

18 Azfi_s0006.g009576/1-294 89.5% 20.6%  **NPDEVCPAGWKPGEK-----------------------------------------------------------------**

19 Sacu_v1.1_s0011.g005300/1-273 100.0% 28.9%  **NPDEVCPAGWKPGDK-----------------------------------------------------------------**

20 Sacu_v1.1_s0003.g001962/1-685 99.1% 12.1%  **NPDEVCPAGWKPGQRRVPFVGSSATFDPFPLTVPVAGDGSTDRFSVYPLDMGTQALWYAQCSPSHVAEQCGTGSLSHAVG**

21 Sacu_v1.1_s0025.g009359/1-292 100.0% 25.7%  **HPAQVCPAGWKPGDK-----------------------------------------------------------------**

22 CHBRA41g00500/1-284 100.0% 28.5%  **NPDEVCPAGWKPGEK-----------------------------------------------------------------**

23 AagrBONN_Sc2ySwM_368.1690.4/1-204 77.2% 1.7%  **--------------------------------------------------------------------------------**

24 AagrBONN_/1-213 100.0% 34.7%  **HPEEGCPAGWQPGDK-----------------------------------------------------------------**

cov pid **401**  **. . . . : . . .** **480**

1 Selmo_36651/1-114 100.0% 100.0%  **--------------------------------------------------------------------------------**

2 Selmo_420519/1-272 100.0% 35.3%  **--------------------------------------------------------------------------------**

3 Selmo_96390/1-190 98.2% 37.4%  **--------------------------------------------------------------------------------**

4 AT3G11630/1-266 100.0% 32.0%  **--------------------------------------------------------------------------------**

5 AT5G06290/1-273 100.0% 31.5%  **--------------------------------------------------------------------------------**

6 Bradi3g45140/1-260 100.0% 30.8%  **--------------------------------------------------------------------------------**

7 Bradi5g09650/1-260 100.0% 31.9%  **--------------------------------------------------------------------------------**

8 Cre02.g114600/1-198 100.0% 28.8%  **--------------------------------------------------------------------------------**

9 Cre06.g257601/1-235 100.0% 30.2%  **--------------------------------------------------------------------------------**

10 Cre17.g743897/1-387 96.5% 12.5%  **--------------------------------------------------------------------------------**

11 Mapoly0014s0048/1-303 100.0% 24.8%  **--------------------------------------------------------------------------------**

12 Mapoly0014s0048/1-223 100.0% 33.6%  **--------------------------------------------------------------------------------**

13 Mapoly0014s0048/1-275 100.0% 27.3%  **--------------------------------------------------------------------------------**

14 Pp3c1_21200/1-282 100.0% 28.0%  **--------------------------------------------------------------------------------**

15 Pp3c2_16260/1-282 100.0% 28.0%  **--------------------------------------------------------------------------------**

16 Azfi_s2786.g113096/1-270 99.1% 29.0%  **--------------------------------------------------------------------------------**

17 Azfi_s0101.g044406/1-270 100.0% 29.3%  **--------------------------------------------------------------------------------**

18 Azfi_s0006.g009576/1-294 89.5% 20.6%  **--------------------------------------------------------------------------------**

19 Sacu_v1.1_s0011.g005300/1-273 100.0% 28.9%  **--------------------------------------------------------------------------------**

20 Sacu_v1.1_s0003.g001962/1-685 99.1% 12.1%  **FIKEFDYNGHASYGPSRKTVPIGPEYQADIPNVIICNEKGNGDFEEKTETERWIGKRIWPQRDDKLGYSFEEHSTEHPEL**

21 Sacu_v1.1_s0025.g009359/1-292 100.0% 25.7%  **--------------------------------------------------------------------------------**

22 CHBRA41g00500/1-284 100.0% 28.5%  **--------------------------------------------------------------------------------**

23 AagrBONN_Sc2ySwM_368.1690.4/1-204 77.2% 1.7%  **--------------------------------------------------------------------------------**

24 AagrBONN_/1-213 100.0% 34.7%  **--------------------------------------------------------------------------------**

cov pid **481**  **. 5 . . . . : .** **560**

1 Selmo_36651/1-114 100.0% 100.0%  **--------------------------------------------------------------------------------**

2 Selmo_420519/1-272 100.0% 35.3%  **--------------------------------------------------------------------------------**

3 Selmo_96390/1-190 98.2% 37.4%  **--------------------------------------------------------------------------------**

4 AT3G11630/1-266 100.0% 32.0%  **--------------------------------------------------------------------------------**

5 AT5G06290/1-273 100.0% 31.5%  **--------------------------------------------------------------------------------**

6 Bradi3g45140/1-260 100.0% 30.8%  **--------------------------------------------------------------------------------**

7 Bradi5g09650/1-260 100.0% 31.9%  **--------------------------------------------------------------------------------**

8 Cre02.g114600/1-198 100.0% 28.8%  **--------------------------------------------------------------------------------**

9 Cre06.g257601/1-235 100.0% 30.2%  **--------------------------------------------------------------------------------**

10 Cre17.g743897/1-387 96.5% 12.5%  **--------------------------------------------------------------------------------**

11 Mapoly0014s0048/1-303 100.0% 24.8%  **--------------------------------------------------------------------------------**

12 Mapoly0014s0048/1-223 100.0% 33.6%  **--------------------------------------------------------------------------------**

13 Mapoly0014s0048/1-275 100.0% 27.3%  **--------------------------------------------------------------------------------**

14 Pp3c1_21200/1-282 100.0% 28.0%  **--------------------------------------------------------------------------------**

15 Pp3c2_16260/1-282 100.0% 28.0%  **--------------------------------------------------------------------------------**

16 Azfi_s2786.g113096/1-270 99.1% 29.0%  **--------------------------------------------------------------------------------**

17 Azfi_s0101.g044406/1-270 100.0% 29.3%  **--------------------------------------------------------------------------------**

18 Azfi_s0006.g009576/1-294 89.5% 20.6%  **--------------------------------------------------------------------------------**

19 Sacu_v1.1_s0011.g005300/1-273 100.0% 28.9%  **--------------------------------------------------------------------------------**

20 Sacu_v1.1_s0003.g001962/1-685 99.1% 12.1%  **CFCAIQGTIECVRLHIQEKRKNLRNELGDAFSLWGFDEMGEVVAEHWTNEEQLAFEDFFRRNPVSFGRELLDIFPSKSIA**

21 Sacu_v1.1_s0025.g009359/1-292 100.0% 25.7%  **--------------------------------------------------------------------------------**

22 CHBRA41g00500/1-284 100.0% 28.5%  **--------------------------------------------------------------------------------**

23 AagrBONN_Sc2ySwM_368.1690.4/1-204 77.2% 1.7%  **--------------------------------------------------------------------------------**

24 AagrBONN_/1-213 100.0% 34.7%  **--------------------------------------------------------------------------------**

cov pid **561**  **. . . 6 . . . .** **640**

1 Selmo_36651/1-114 100.0% 100.0%  **--------------------------------------------------------------------------------**

2 Selmo_420519/1-272 100.0% 35.3%  **--------------------------------------------------------------------------------**

3 Selmo_96390/1-190 98.2% 37.4%  **--------------------------------------------------------------------------------**

4 AT3G11630/1-266 100.0% 32.0%  **--------------------------------------------------------------------------------**

5 AT5G06290/1-273 100.0% 31.5%  **--------------------------------------------------------------------------------**

6 Bradi3g45140/1-260 100.0% 30.8%  **--------------------------------------------------------------------------------**

7 Bradi5g09650/1-260 100.0% 31.9%  **--------------------------------------------------------------------------------**

8 Cre02.g114600/1-198 100.0% 28.8%  **--------------------------------------------------------------------------------**

9 Cre06.g257601/1-235 100.0% 30.2%  **--------------------------------------------------------------------------------**

10 Cre17.g743897/1-387 96.5% 12.5%  **--------------------------------------------------------------------------------**

11 Mapoly0014s0048/1-303 100.0% 24.8%  **--------------------------------------------------------------------------------**

12 Mapoly0014s0048/1-223 100.0% 33.6%  **--------------------------------------------------------------------------------**

13 Mapoly0014s0048/1-275 100.0% 27.3%  **--------------------------------------------------------------------------------**

14 Pp3c1_21200/1-282 100.0% 28.0%  **--------------------------------------------------------------------------------**

15 Pp3c2_16260/1-282 100.0% 28.0%  **--------------------------------------------------------------------------------**

16 Azfi_s2786.g113096/1-270 99.1% 29.0%  **--------------------------------------------------------------------------------**

17 Azfi_s0101.g044406/1-270 100.0% 29.3%  **--------------------------------------------------------------------------------**

18 Azfi_s0006.g009576/1-294 89.5% 20.6%  **--------------------------------------------------------------------------------**

19 Sacu_v1.1_s0011.g005300/1-273 100.0% 28.9%  **--------------------------------------------------------------------------------**

20 Sacu_v1.1_s0003.g001962/1-685 99.1% 12.1%  **DLVSYYFNVFVLRRRAIQNRIDPENVDSDSDEMILDSEEKESDSLLGSDNDDESQVESQVDDGVCDEEEEEEDEEDEDED**

21 Sacu_v1.1_s0025.g009359/1-292 100.0% 25.7%  **--------------------------------------------------------------------------------**

22 CHBRA41g00500/1-284 100.0% 28.5%  **--------------------------------------------------------------------------------**

23 AagrBONN_Sc2ySwM_368.1690.4/1-204 77.2% 1.7%  **--------------------------------------------------------------------------------**

24 AagrBONN_/1-213 100.0% 34.7%  **--------------------------------------------------------------------------------**

cov pid **641**  **: . . . . 7 . .** **720**

1 Selmo_36651/1-114 100.0% 100.0%  **--------------------------------------------------------------------------------**

2 Selmo_420519/1-272 100.0% 35.3%  **-----------------------------------------------SMKPDPK--------------------------**

3 Selmo_96390/1-190 98.2% 37.4%  **-----------------------------------------------GIKPDPK--------------------------**

4 AT3G11630/1-266 100.0% 32.0%  **-----------------------------------------------SMKPDPK--------------------------**

5 AT5G06290/1-273 100.0% 31.5%  **-----------------------------------------------SMKPDPK--------------------------**

6 Bradi3g45140/1-260 100.0% 30.8%  **-----------------------------------------------SMKPDPK--------------------------**

7 Bradi5g09650/1-260 100.0% 31.9%  **-----------------------------------------------SMKPDPK--------------------------**

8 Cre02.g114600/1-198 100.0% 28.8%  **-----------------------------------------------TMKADPT--------------------------**

9 Cre06.g257601/1-235 100.0% 30.2%  **-----------------------------------------------TMKPDPK--------------------------**

10 Cre17.g743897/1-387 96.5% 12.5%  **-----------------------------------------------TMVADAE--------------------------**

11 Mapoly0014s0048/1-303 100.0% 24.8%  **-----------------------------------------------TMKPDSK--------------------------**

12 Mapoly0014s0048/1-223 100.0% 33.6%  **-----------------------------------------------TMKPDSK--------------------------**

13 Mapoly0014s0048/1-275 100.0% 27.3%  **-----------------------------------------------TMKPDSK--------------------------**

14 Pp3c1_21200/1-282 100.0% 28.0%  **-----------------------------------------------TMKPDSK--------------------------**

15 Pp3c2_16260/1-282 100.0% 28.0%  **-----------------------------------------------TMKPDSK--------------------------**

16 Azfi_s2786.g113096/1-270 99.1% 29.0%  **---TMKPDTK----------------------------------------------------------LSKEYFSAI---**

17 Azfi_s0101.g044406/1-270 100.0% 29.3%  **-----------------------------------------------TMKPDTK--------------------------**

18 Azfi_s0006.g009576/1-294 89.5% 20.6%  **-----------------------------------------------TMKPDTK--------------------------**

19 Sacu_v1.1_s0011.g005300/1-273 100.0% 28.9%  **-----------------------------------------------TMKPDTK--------------------------**

20 Sacu_v1.1_s0003.g001962/1-685 99.1% 12.1%  **GCIMWSMKTNATHEEMLTNSRSFMVDSTEPEEMSVDSSSIKYNSIPEHLKPITKVLPENCYRGDVSQMVKSWEMLSWEQT**

21 Sacu_v1.1_s0025.g009359/1-292 100.0% 25.7%  **-----------------------------------------------TITSNPM--------------------------**

22 CHBRA41g00500/1-284 100.0% 28.5%  **-----------------------------------------------SMKPDPK--------------------------**

23 AagrBONN_Sc2ySwM_368.1690.4/1-204 77.2% 1.7%  **--------------------------------------------------------------------------------**

24 AagrBONN_/1-213 100.0% 34.7%  **-----------------------------------------------PLK------------------------------**

cov pid **721**  **. . : . . . . 8** **800**

1 Selmo_36651/1-114 100.0% 100.0%  **--------------------------------------------------------------------------------**

2 Selmo_420519/1-272 100.0% 35.3%  **--------------------------------LSKDYFAAIA--------------------------------------**

3 Selmo_96390/1-190 98.2% 37.4%  **------------------------------------HFAAV---------------------------------------**

4 AT3G11630/1-266 100.0% 32.0%  **--------------------------------LSKEYFSAI---------------------------------------**

5 AT5G06290/1-273 100.0% 31.5%  **--------------------------------LSKEYFSAI---------------------------------------**

6 Bradi3g45140/1-260 100.0% 30.8%  **--------------------------------GSKEYFAAI---------------------------------------**

7 Bradi5g09650/1-260 100.0% 31.9%  **--------------------------------GSKEYFAAI---------------------------------------**

8 Cre02.g114600/1-198 100.0% 28.8%  **--------------------------------KSLEYFSTLS--------------------------------------**

9 Cre06.g257601/1-235 100.0% 30.2%  **--------------------------------GSKEYFSAV---------------------------------------**

10 Cre17.g743897/1-387 96.5% 12.5%  **--------------------------------KSLEYFSEVKEADDAGAFGSKLHAIASRAEYDKLTRESQGLVVVDFYA**

11 Mapoly0014s0048/1-303 100.0% 24.8%  **--------------------------------LSKEYFAAI---------------------------------------**

12 Mapoly0014s0048/1-223 100.0% 33.6%  **--------------------------------LSKEYFAAI---------------------------------------**

13 Mapoly0014s0048/1-275 100.0% 27.3%  **--------------------------------LSKEYFAAI---------------------------------------**

14 Pp3c1_21200/1-282 100.0% 28.0%  **--------------------------------LSKEYFEAI---------------------------------------**

15 Pp3c2_16260/1-282 100.0% 28.0%  **--------------------------------LSKEYFEAI---------------------------------------**

16 Azfi_s2786.g113096/1-270 99.1% 29.0%  **--------------------------------------------------------------------------------**

17 Azfi_s0101.g044406/1-270 100.0% 29.3%  **--------------------------------LSKEYFSAI---------------------------------------**

18 Azfi_s0006.g009576/1-294 89.5% 20.6%  **--------------------------------LSKEYFAAI---------------------------------------**

19 Sacu_v1.1_s0011.g005300/1-273 100.0% 28.9%  **--------------------------------LSKEYFAAI---------------------------------------**

20 Sacu_v1.1_s0003.g001962/1-685 99.1% 12.1%  **ENVAEGCVKEIWSSPNPISNQDDVDKLISTNGLIEEFFGSEIWKSRK---------------------------------**

21 Sacu_v1.1_s0025.g009359/1-292 100.0% 25.7%  **--------------------------------FSKGYFSAT---------------------------------------**

22 CHBRA41g00500/1-284 100.0% 28.5%  **--------------------------------GSKEYFAAI---------------------------------------**

23 AagrBONN_Sc2ySwM_368.1690.4/1-204 77.2% 1.7%  **--------------------------------------------------------------------------------**

24 AagrBONN_/1-213 100.0% 34.7%  **--------------------------------QGKDYLK-----------------------------------------**

cov pid **801**  **. . . . : . . ]** **880**

1 Selmo_36651/1-114 100.0% 100.0%  **--------------------------------------------------------------------------------**

2 Selmo_420519/1-272 100.0% 35.3%  **--------------------------------------------------------------------------------**

3 Selmo_96390/1-190 98.2% 37.4%  **--------------------------------------------------------------------------------**

4 AT3G11630/1-266 100.0% 32.0%  **--------------------------------------------------------------------------------**

5 AT5G06290/1-273 100.0% 31.5%  **--------------------------------------------------------------------------------**

6 Bradi3g45140/1-260 100.0% 30.8%  **--------------------------------------------------------------------------------**

7 Bradi5g09650/1-260 100.0% 31.9%  **--------------------------------------------------------------------------------**

8 Cre02.g114600/1-198 100.0% 28.8%  **--------------------------------------------------------------------------------**

9 Cre06.g257601/1-235 100.0% 30.2%  **--------------------------------------------------------------------------------**

10 Cre17.g743897/1-387 96.5% 12.5%  **PWCGKCRQIGPFLDTLVDKYPGVTFAKFDTTAPELEVAAGELGIKALPAFRFFKGGKEVGSPVTGYKKKPLEDAVAELAK**

11 Mapoly0014s0048/1-303 100.0% 24.8%  **--------------------------------------------------------------------------------**

12 Mapoly0014s0048/1-223 100.0% 33.6%  **--------------------------------------------------------------------------------**

13 Mapoly0014s0048/1-275 100.0% 27.3%  **--------------------------------------------------------------------------------**

14 Pp3c1_21200/1-282 100.0% 28.0%  **--------------------------------------------------------------------------------**

15 Pp3c2_16260/1-282 100.0% 28.0%  **--------------------------------------------------------------------------------**

16 Azfi_s2786.g113096/1-270 99.1% 29.0%  **--------------------------------------------------------------------------------**

17 Azfi_s0101.g044406/1-270 100.0% 29.3%  **--------------------------------------------------------------------------------**

18 Azfi_s0006.g009576/1-294 89.5% 20.6%  **--------------------------------------------------------------------------------**

19 Sacu_v1.1_s0011.g005300/1-273 100.0% 28.9%  **--------------------------------------------------------------------------------**

20 Sacu_v1.1_s0003.g001962/1-685 99.1% 12.1%  **--------------------------------------------------------------------------------**

21 Sacu_v1.1_s0025.g009359/1-292 100.0% 25.7%  **--------------------------------------------------------------------------------**

22 CHBRA41g00500/1-284 100.0% 28.5%  **--------------------------------------------------------------------------------**

23 AagrBONN_Sc2ySwM_368.1690.4/1-204 77.2% 1.7%  **--------------------------------------------------------------------------------**

24 AagrBONN_/1-213 100.0% 34.7%  **--------------------------------------------------------------------------------**

>Selmo_36651/1-114

------------------------------------------------------------------------

-----------------------------------------------DVPLVGNKGLDFEAESVFNQEFIK-

-VKLSDYF----YVMLFFYPLDFMIICPIEIASFCDRYSEFEKINTEVLKVSVDSVFSHLSWVQTERKLGGL

GNLHYPLVSDISKSISKAYNVHI-------------------------------------------------

------------------------------------------------------------------------

------------------------------------------------------------------------

------------------------------------------------------------------------

------------------------------------------------------------------------

------------------------------------------------------------------------

------------------------------------------------------------------------

------------------------------------------------------------------------

------------------------------------------------------------------------

----------------

>Selmo_420519/1-272

--------------------------MATAGAAAFAATGHVVGGGATSSSSSAASAAPCRSSLFKAFDARLG

ASKISHLRRPAARIQPRRAFR------------------SLAASASLDVPLVGNKAPDFEAESVFDQEFIK-

-VKLSDYLGK-KYVVLFFYPLDFTFVCPTEITAFSDRYSEFEKINTEVLGVSVDSVFSHLAWVQTERKSGGL

GDLRYPLVSDISKSISKAYNVLIP---D----------------------------QGIAL--RGLFIIDKE

GIIQHATVNNLAIGRSVDETLRTLQAVQYVQDNPDEVCPAGWKPGDK-------------------------

------------------------------------------------------------------------

------------------------------------------------------------------------

------------------------------------------------------------------------

------------------------------------------------------------------------

---------------------------------------SMKPDPK--------------------------

--------------------------------LSKDYFAAIA------------------------------

------------------------------------------------------------------------

----------------

>Selmo_96390/1-190

------------------------------------------------------------------------

-------------------------------------------------PLVGSMAPNFEAEAVHHQEFIK-

-VKLSNYIGK-KFVVLFFYPLNFTFVCPTELTAFSDRYGEFQKLDTEVLAVSSDSVFSHLAWIQTDRKSGGL

GELNYPVVSDLTKAITKAYRVLLP---D----------------------------QGISL--RGLFIIDKE

GIVQHATINNLAVGRNVDEALRLVQAVQYVKVHPDELCPAGWQPGEH-------------------------

------------------------------------------------------------------------

------------------------------------------------------------------------

------------------------------------------------------------------------

------------------------------------------------------------------------

---------------------------------------GIKPDPK--------------------------

------------------------------------HFAAV-------------------------------

------------------------------------------------------------------------

----------------

>AT3G11630/1-266

-------------------------------MASVASSTTLISSPSSRVFPAKSSLSSPSVSFLRTL-----

SSPSASASLRSGFARRSSLSS-------------TSRRSFAVKAQADDLPLVGNKAPDFEAEAVFDQEFIK-

-VKLSDYIGK-KYVILFFYPLDFTFVCPTEITAFSDRHSEFEKLNTEVLGVSVDSVFSHLAWVQTDRKSGGL

GDLNYPLISDVTKSISKSFGVLIH---D----------------------------QGIAL--RGLFIIDKE

GVIQHSTINNLGIGRSVDETMRTLQALQYIQENPDEVCPAGWKPGEK-------------------------

------------------------------------------------------------------------

------------------------------------------------------------------------

------------------------------------------------------------------------

------------------------------------------------------------------------

---------------------------------------SMKPDPK--------------------------

--------------------------------LSKEYFSAI-------------------------------

------------------------------------------------------------------------

----------------

>AT5G06290/1-273

----------------------------MSMASIASSSSTTLLSSSRVLLPSKSSLLSPTVSFPRIIPSSSA

SSSSLCSGFSSLGSLTTNRSA--------------SRRNFAVKAQADDLPLVGNKAPDFEAEAVFDQEFIK-

-VKLSEYIGK-KYVILFFYPLDFTFVCPTEITAFSDRYEEFEKLNTEVLGVSVDSVFSHLAWVQTDRKSGGL

GDLNYPLVSDITKSISKSFGVLIP---D----------------------------QGIAL--RGLFIIDKE

GVIQHSTINNLGIGRSVDETMRTLQALQYVQENPDEVCPAGWKPGEK-------------------------

------------------------------------------------------------------------

------------------------------------------------------------------------

------------------------------------------------------------------------

------------------------------------------------------------------------

---------------------------------------SMKPDPK--------------------------

--------------------------------LSKEYFSAI-------------------------------

------------------------------------------------------------------------

----------------

>Bradi3g45140/1-260

-------------------------------MACSFATSTVVSSTP-TPKPLATALTPQCLSISRAP-----

---------L-ATVRPLRLAA--------ASRSARTSGFVARAGGVDELPLVGNKAPDFDAEAVFDQEFIN-

-VKLSDYIGK-KYVILFFYPLDFTFVCPTEITAFSDRHDEFEKINTQVLGVSIDSVFSHLAWVQTDRKSGGL

GDLKYPLISDVTKSISKSFGVLIP---H----------------------------QGIAL--RGLFIIDKE

GVIQHSTINNLAIGRSVDETMRTLQALQYVQENPDEVCPAGWKPGEK-------------------------

------------------------------------------------------------------------

------------------------------------------------------------------------

------------------------------------------------------------------------

------------------------------------------------------------------------

---------------------------------------SMKPDPK--------------------------

--------------------------------GSKEYFAAI-------------------------------

------------------------------------------------------------------------

----------------

>Bradi5g09650/1-260

-------------------------------MACAFSASTVSPAAA-LVASPKPTAAPQFLSFPRAF-----

---------VGGAARPSRLAA---------SRTARARNFVARAGGEDSLPLVGNKAPDFDAEAVFDQEFIN-

-VKLSDYIGK-KYVILFFYPLDFTFVCPTEITAFSDRHEEFEKINTEILGVSVDSVFSHLAWVQTERKSGGL

GDLKYPLVSDVTKSISKSFGVLIP---D----------------------------QGIAL--RGLFIIDKE

GVIQHSTINNLGIGRSVDETLRTLQALQYVQENPDEVCPAGWKPGEK-------------------------

------------------------------------------------------------------------

------------------------------------------------------------------------

------------------------------------------------------------------------

------------------------------------------------------------------------

---------------------------------------SMKPDPK--------------------------

--------------------------------GSKEYFAAI-------------------------------

------------------------------------------------------------------------

----------------

>Cre02.g114600/1-198

------------------------------------------------------------------------

-----------------------------------------------MVAKIGAPAPKFKAQAVVNGEIKE-

-ISLDDYKGK--YVVLFFYPLDFTFVCPTEIVAFSDRVEEFRAINTEVIGASIDSQFTHLAFSNTPRTKGGL

GGCKYPLVADLTKQIAKDYGVLIEDGPD----------------------------AGVTL--RGLFIISPT

GVLRQITINDLPVGRSVDETLRLVKAFQFTDEH-GEVCPANWNPGAK-------------------------

------------------------------------------------------------------------

------------------------------------------------------------------------

------------------------------------------------------------------------

------------------------------------------------------------------------

---------------------------------------TMKADPT--------------------------

--------------------------------KSLEYFSTLS------------------------------

------------------------------------------------------------------------

----------------

>Cre06.g257601/1-235

----------------------------MAALQSASRSSAVAFSRQARVAPRVAASVA--------------

------------------------------------RRSLVVRASHAEKPLVGSVAPDFKAQAVFDQEFQE-

-ITLSKYRGK--YVVLFFYPLDFTFVCPTEITAFSDRYKEFKDINTEVLGVSVDSQFTHLAWIQTDRKEGGL

GDLAYPLVADLKKEISKAYGVLTE---D-----------------------------GISL--RGLFIIDKE

GVVQHATINNLAFGRSVDETKRVLQAIQYVQSNPDEVCPAGWKPGDK-------------------------

------------------------------------------------------------------------

------------------------------------------------------------------------

------------------------------------------------------------------------

------------------------------------------------------------------------

---------------------------------------TMKPDPK--------------------------

--------------------------------GSKEYFSAV-------------------------------

------------------------------------------------------------------------

----------------

>Cre17.g743897/1-387

---------------MSRHLRRASTLLARLAAHGANKEPLVASMAGRALAQSVVQMPMWQIVQPRGFASSSS

SDDEFFEEDQMPIIYPPSQVR------------------------------VGERAPNFSAPAVVDGEITK-

-ISLSDYKGK--YVCLFFYPKDFTFVCPTEIIAFSDRAKEFAAANCQLIAASTDTEETHLAWIRTPRNRGGL

GYMQIPILADTTKDISARYGVLIE---K----------------------------LGVAL--RGLFIINPQ

GVVQHVTINDLPIGRSVDEALRTLQAIQYHAEH-GEVCPANWKPGSK-------------------------

------------------------------------------------------------------------

------------------------------------------------------------------------

------------------------------------------------------------------------

------------------------------------------------------------------------

---------------------------------------TMVADAE--------------------------

--------------------------------KSLEYFSEVKEADDAGAFGSKLHAIASRAEYDKLTRESQG

LVVVDFYAPWCGKCRQIGPFLDTLVDKYPGVTFAKFDTTAPELEVAAGELGIKALPAFRFFKGGKEVGSPVT

GYKKKPLEDAVAELAK

>Mapoly0014s0048/1-303

-----------------------------MATACAARSAVALPLASVAATTASSSTAAPSVAIPRSFDGLNK

SFGARLATRSTSAFCKPVTGV-----------SLSQSARRFVVSASKEAPLVGNVAPDFEAEAVFDQEFVK-

-IKLSEYIGK-KYVVLFFYPLDFTFVCPTEITAFSDKHAEFEKLNTEVIGVSTDSVFSHLAWIQTDRKAGGL

GDLNYPLVSDLTKKISKDFGVLIP---DQRMWEIQMKDQNSRKSVEKTVTAHSGPSQGIAL--RGLFIIDKE

GVIQHATINNLAIGRSVEETLRTLQAVQYVQENPDEVCPAGWKPGEK-------------------------

------------------------------------------------------------------------

------------------------------------------------------------------------

------------------------------------------------------------------------

------------------------------------------------------------------------

---------------------------------------TMKPDSK--------------------------

--------------------------------LSKEYFAAI-------------------------------

------------------------------------------------------------------------

----------------

>Mapoly0014s0048/1-223

------------------------------------------MAKPPAPSNEIRDLCVRLSSL---------

-----------------------------------------SGSQSKEAPLVGNVAPDFEAEAVFDQEFVK-

-IKLSEYIGK-KYVVLFFYPLDFTFVCPTEITAFSDKHAEFEKLNTEVIGVSTDSVFSHLAWIQTDRKAGGL

GDLNYPLVSDLTKKISKDFGVLIP---D----------------------------QGIAL--RGLFIIDKE

GVIQHATINNLAIGRSVEETLRTLQAVQYVQENPDEVCPAGWKPGEK-------------------------

------------------------------------------------------------------------

------------------------------------------------------------------------

------------------------------------------------------------------------

------------------------------------------------------------------------

---------------------------------------TMKPDSK--------------------------

--------------------------------LSKEYFAAI-------------------------------

------------------------------------------------------------------------

----------------

>Mapoly0014s0048/1-275

-----------------------------MATACAARSAVALPLASVAATTASSSTAAPSVAIPRSFDGLNK

SFGARLATRSTSAFCKPVTGV-----------SLSQSARRFVVSASKEAPLVGNVAPDFEAEAVFDQEFVK-

-IKLSEYIGK-KYVVLFFYPLDFTFVCPTEITAFSDKHAEFEKLNTEVIGVSTDSVFSHLAWIQTDRKAGGL

GDLNYPLVSDLTKKISKDFGVLIP---D----------------------------QGIAL--RGLFIIDKE

GVIQHATINNLAIGRSVEETLRTLQAVQYVQENPDEVCPAGWKPGEK-------------------------

------------------------------------------------------------------------

------------------------------------------------------------------------

------------------------------------------------------------------------

------------------------------------------------------------------------

---------------------------------------TMKPDSK--------------------------

--------------------------------LSKEYFAAI-------------------------------

------------------------------------------------------------------------

----------------

>Pp3c1_21200/1-282

-------------------------MACAAAASGVAASSVVIPRAPSAACSSSASGTSTSLAVPKSFCGLGK

SFGARVATANVAVAKAGAVAG--------AKAASQKRGVARASAEDDYAPLVGNVAPDFEAEAVFDQEFIK-

-IKLSEYIGK-KYVVLFFYPLDFTFVCPTEITAFSDRYSEFEKLDTEVIGVSIDSVFSHLAWVQTDRKSGGL

GELSYPIVSDITKKISKSFGVLIP---D----------------------------QGIAL--RGLFIIDKQ

GVIQHATINNLGIGRSVDETLRTLQAVQYVQDNPDEVCPAGWKPGEK-------------------------

------------------------------------------------------------------------

------------------------------------------------------------------------

------------------------------------------------------------------------

------------------------------------------------------------------------

---------------------------------------TMKPDSK--------------------------

--------------------------------LSKEYFEAI-------------------------------

------------------------------------------------------------------------

----------------

>Pp3c2_16260/1-282

-------------------------MACAAAASGVTLPAGNIAHARPAAFSSSASGASSSLSVRKSFSGLSK

SFAARLAATNAPTGKAASAAG--------VKIASQKRGVITASAQDSYEPLIGNVAPDFEAEAVFDQEFIK-

-VKLSEYIGK-KYVVLFFYPLDFTFVCPTEITAFSDRYSEFEKLSTEVIGVSTDSVFSHLAWIQTDRKAGGL

GDLHYPIVSDITKKISRSFNVLIP---E----------------------------QGIAL--RGLFIIDKQ

GVIQHATVNNLGIGRSVDETLRTLQAVQYVQDNPDEVCPAGWKPGEK-------------------------

------------------------------------------------------------------------

------------------------------------------------------------------------

------------------------------------------------------------------------

------------------------------------------------------------------------

---------------------------------------TMKPDSK--------------------------

--------------------------------LSKEYFEAI-------------------------------

------------------------------------------------------------------------

----------------

>Azfi_s2786.g113096/1-270

----------------------------MAMAACAASSAAILSSASASPSSSPLVASVPRSHLRSSFVSTVS

KLRSSSSPSPASFSSLSCRAS-----------------PRPLVVRAGEIPLVGNVAPDFEAEAVFDQEFTK-

-IKLSDYRGK-KYVVLFFYPLDFTFVCPTEITAFSDRYEEFEKVNTEVIGVSVDSVFSHLAWIQTDRKSGGL

GDLKYPLVADLTKTVAKA-RGLFIIDKEGVIQHATINNLGIGRSVDETLRTLQAVQYVQDNPDEVCPAGWKP

GEK---------------------------------------------------------------------

------------------------------------------------------------------------

------------------------------------------------------------------------

------------------------------------------------------------------------

-------------------------------------------------------------------TMKPD

TK----------------------------------------------------------LSKEYFSAI---

------------------------------------------------------------------------

------------------------------------------------------------------------

----------------

>Azfi_s0101.g044406/1-270

----------------------------MAMAACAASSAAILSSASASPSSSPLVASVPRSHLRSSFVSTVS

KLRSSSSPSPASFSSLSCRAS-----------------PRPLVVRAGEIPLVGNVAPDFEAEAVFDQEFTK-

-IKLSDYRGK-KYVVLFFYPLDFTFVCPTEITAFSDRYEEFEKVNTEVIGVSVDSVFSHLAWIQTDRKSGGL

GDLKYPLVADLTKTVAKAYNVLIP---D----------------------------QGVAL--RGLFIIDKE

GVIQHATINNLGIGRSVDETLRTLQAVQYVQDNPDEVCPAGWKPGEK-------------------------

------------------------------------------------------------------------

------------------------------------------------------------------------

------------------------------------------------------------------------

------------------------------------------------------------------------

---------------------------------------TMKPDTK--------------------------

--------------------------------LSKEYFSAI-------------------------------

------------------------------------------------------------------------

----------------

>Azfi_s0006.g009576/1-294

IFFLFLFLSLSLSLSLSLIRLVSVSKPRLPSCLCFHHGYLPPSPFLSSPSSSSSSSSIVTASAPRLLYPRVR

SLWSSSFSPPLPGLRQRPVSS--------SSLCFKDSSRSSSSSSS-----SSSSSPAFVVRA---------

-IKLSDYRGK-KYVVLFFYPLDFTFVCPTEITAFSDRYAEFEELNTEVIGISIDSVFSHLAWIQTDRKSGGL

GDLKYPLVADLTKSISKSYNVLIP---G----------------------------QGVAL--RGLFILDKE

GVIQHATINNLAIGRSVEETLRTLQAVQYVQDNPDEVCPAGWKPGEK-------------------------

------------------------------------------------------------------------

------------------------------------------------------------------------

------------------------------------------------------------------------

------------------------------------------------------------------------

---------------------------------------TMKPDTK--------------------------

--------------------------------LSKEYFAAI-------------------------------

------------------------------------------------------------------------

----------------

>Sacu_v1.1_s0011.g005300/1-273

---------------------------MAISASSIITASCFNPPSLASPYSSSSSILAPRVSRDVDLRSGFI

SPVSQFRPLKRPPLCRQNSSR-------------------SLVARAGELPLVGNVAPDFEAEAVFDQEFVN-

-IKLSDYRGK-KYVVLFFYPLDFTFVCPTEITAFSDRHSEFEKLNTEVIGVSIDSVFSHLAWIQTDRKLGGL

GDLKYPLVADLTKTISKAFNVLIP---E------------------------QAGRNGIAL--RGLFIIDKD

GVIQHATINNLAIGRSVDETLRTLQAVQYVQENPDEVCPAGWKPGDK-------------------------

------------------------------------------------------------------------

------------------------------------------------------------------------

------------------------------------------------------------------------

------------------------------------------------------------------------

---------------------------------------TMKPDTK--------------------------

--------------------------------LSKEYFAAI-------------------------------

------------------------------------------------------------------------

----------------

>Sacu_v1.1_s0003.g001962/1-685

-------------------------MASTCSPAAVSSFSTPAAISSPSSSSSSIAFPPSRPALLKSP-----

--------FLPRLSHLRPLSS--------SARTSPSSRPLVVRAGE--LPLVGNVAPDFEAEAVFDQEFINV

LVKLSDYRGK-KYVVLFFYPLDFTFVCPTEITAFSDRYEEFEKLNTEVLGVSIDSVFSHLAWVQTDRKSGGL

GDLKYPLVADLTKSISKAFNVLIP---S----------------------------QGVAL--RGLFIIDKE

GVIQHATINNLAIGRSVDETLRTLQAVQYVQDNPDEVCPAGWKPGQRRVPFVGSSATFDPFPLTVPVAGDGS

TDRFSVYPLDMGTQALWYAQCSPSHVAEQCGTGSLSHAVGFIKEFDYNGHASYGPSRKTVPIGPEYQADIPN

VIICNEKGNGDFEEKTETERWIGKRIWPQRDDKLGYSFEEHSTEHPELCFCAIQGTIECVRLHIQEKRKNLR

NELGDAFSLWGFDEMGEVVAEHWTNEEQLAFEDFFRRNPVSFGRELLDIFPSKSIADLVSYYFNVFVLRRRA

IQNRIDPENVDSDSDEMILDSEEKESDSLLGSDNDDESQVESQVDDGVCDEEEEEEDEEDEDEDGCIMWSMK

TNATHEEMLTNSRSFMVDSTEPEEMSVDSSSIKYNSIPEHLKPITKVLPENCYRGDVSQMVKSWEMLSWEQT

ENVAEGCVKEIWSSPNPISNQDDVDKLISTNGLIEEFFGSEIWKSRK-------------------------

------------------------------------------------------------------------

----------------

>Sacu_v1.1_s0025.g009359/1-292

-----------------------MLQTVQIAVPCQMQAFAPPSPRPTYCLSSSATAILSSARLPHCLLQKKQ

PHRRGTKKNRYSPRNHRNRSSFPFPSYFDDSSSIFSFTPMSASNAISENTLVGNAAPDFEAEAVLNREFVK-

-VKLSDYIGK-SYVILFFYPLDFTFVCPTEITAFSDCYSEFEKLETKIIGISTDSVFSHLAWVQVDRKEGGL

GDLQYPLVSDITKGISRNYNVLIR---D----------------------------QGVAL--RGLFIIDKS

GIIQYATINNLRFGRSVDETLRILQAIQYVQEHPAQVCPAGWKPGDK-------------------------

------------------------------------------------------------------------

------------------------------------------------------------------------

------------------------------------------------------------------------

------------------------------------------------------------------------

---------------------------------------TITSNPM--------------------------

--------------------------------FSKGYFSAT-------------------------------

------------------------------------------------------------------------

----------------

>CHBRA41g00500/1-284

-------------------------MAATTLHASLSTAAVGLSHNGGIGTQCQRRSATSALPAVKLTTYEGL

GKASRLTADVVPRVSTRKNLK-------CGAVSSPDSSSKTLDIRAHCQPLVGNKAPDFEAKAVFDQEFIE-

-VKLSDYQAQGKYVVLFFYPLDFTFVCPTEITAFSDRYAEFAALNTEVLGVSVDSVFSHLAWIQTERKSGGL

GDLSYPLVSDITKDIARQYNVLIE---E----------------------------EGVAL--RGLFIIDTE

GVIQHATINNLAIGRSVDETLRTLQAVQYVQENPDEVCPAGWKPGEK-------------------------

------------------------------------------------------------------------

------------------------------------------------------------------------

------------------------------------------------------------------------

------------------------------------------------------------------------

---------------------------------------SMKPDPK--------------------------

--------------------------------GSKEYFAAI-------------------------------

------------------------------------------------------------------------

----------------

>AagrBONN_Sc2ySwM_368.1690.4/1-204

MAMAVAAATAAPALSPAASAAVSSASSSASTSLVAVPRRFSGLSTSFEARLTAGVPVQHKGRSASR------

----QAASAGRRTVVSAYEAEFPLVGNVAPDFEAEAVFDQEFVK--IKLSEYIGK-KYVVLFFYPLDFTFVC

PTEITAFSDRYSEFEKLNTEVIGVSTDSVFSHLAWIQTDRKSGGLGDLLFPLVSDLTKCISKKYQVLIP---

D----------------------------Q-VS---------------------------------------

------------------------------------------------------------------------

------------------------------------------------------------------------

------------------------------------------------------------------------

------------------------------------------------------------------------

------------------------------------------------------------------------

------------------------------------------------------------------------

------------------------------------------------------------------------

------------------------------------------------------------------------

----------------

>AagrBONN_/1-213

----------------------------------------MKGVSKYMLLQGDQPQPPPKLPI---------

-----------------------------------------------HDPLVGHIAPDFEAEAVFEQEFVK-

-IKLSSYFGK-KFVVLFFYPLDFTFVCPTEITQFSDRYDEFQKLNTEVIAVSTDSVFSHLAWVQTDRKAGGL

GDLKYPIASDLTKVISRRYGVLIA---D----------------------------QGVAL--RGLFIIDKQ

GVIQHATINNLSFGRSVDETIRTLHAVQHVQQHPEEGCPAGWQPGDK-------------------------

------------------------------------------------------------------------

------------------------------------------------------------------------

------------------------------------------------------------------------

------------------------------------------------------------------------

---------------------------------------PLK------------------------------

--------------------------------QGKDYLK---------------------------------

------------------------------------------------------------------------

----------------

**SAL1**

cov pid  **1** **[ . . . . : . . .** **80**

1 Cre01.g030250/1-347 100.0% 100.0%  **--------------------------------------------------------------------------------**

2 CHBRA172g00210/1-345 96.0% 47.6%  **--------------------------------------------------------------------------------**

3 AagrBONN/1-300 82.7% 46.7%  **--------------------------------------------------------------------------------**

4 Mapoly0030s0063/1-404 97.4% 42.1%  **-------------------------------------------MQALGARRAFLRSSFPCSRSSSSWKALGNCRAPSIVR**

5 Pp3c15_5590/1-402 97.4% 44.0%  **------------------------------------------MLKEVVHRSHHLWQCFPRHLHCHDWSPVNSKARSRSGR**

6 Selmo/1-334 93.9% 46.5%  **--------------------------------------------------------------------------------**

7 Selmo/1-443 99.4% 34.8%  **------------------------MLGNFDSLREISRNARGWSVGSLACPRARSWRPSAVSSETTGNIDSRAQPRPQCLE**

8 Sacu_v1.1_s0154.g023570/1-394 96.3% 42.8%  **----------------------------------MEELILVHNVLYIVCLRIAMQQLNFRLYSRIPIAPPFLCSRSSVSR**

9 Azfi_s0022.g016236/1-392 95.4% 41.4%  **-----------------------------------MQYLRGNTLLVYNFPRSSLNSLCRCSYTCRTTTTMSLPST-----**

10 Bradi4g40860/1-424 97.1% 38.0%  **----------------------------MAARVGLSHALLASSSLARNPPPRARLLPYPTLLPLPTYRRSVSASASALPS**

11 AT5G63980_AtSAL1/1-353 97.7% 46.5%  **--------------------------------------------------------------------------------**

cov pid  **81**  **. 1 . . . . : .** **160**

1 Cre01.g030250/1-347 100.0% 100.0%  **--------------------------------------------------------------------------------**

2 CHBRA172g00210/1-345 96.0% 47.6%  **--------------------------------------------------------------------------------**

3 AagrBONN/1-300 82.7% 46.7%  **--------------------------------------------------------------------------------**

4 Mapoly0030s0063/1-404 97.4% 42.1%  **LGRRGVGAIRS---------------------------------------------------------------------**

5 Pp3c15_5590/1-402 97.4% 44.0%  **GSVSWRKPLFCM--------------------------------------------------------------------**

6 Selmo/1-334 93.9% 46.5%  **--------------------------------------------------------------------------------**

7 Selmo/1-443 99.4% 34.8%  **HNRDGNDKKITR--------------------------------------------------------------------**

8 Sacu_v1.1_s0154.g023570/1-394 96.3% 42.8%  **TTMSL---------------------------------------------------------------------------**

9 Azfi_s0022.g016236/1-392 95.4% 41.4%  **--------------------------------------------------------------------------------**

10 Bradi4g40860/1-424 97.1% 38.0%  **LRRRRPFAARAMSQPPEAA-------------------------------------------------------------**

11 AT5G63980_AtSAL1/1-353 97.7% 46.5%  **--------------------------------------------------------------------------------**

cov pid **161**  **. . . 2 . . . .** **240**

1 Cre01.g030250/1-347 100.0% 100.0%  **--------------------------------------------------------------------------------**

2 CHBRA172g00210/1-345 96.0% 47.6%  **--------------------------------------------------------------------------------**

3 AagrBONN/1-300 82.7% 46.7%  **--------------------------------------------------------------------------------**

4 Mapoly0030s0063/1-404 97.4% 42.1%  **--------------------------------------------------------------------------------**

5 Pp3c15_5590/1-402 97.4% 44.0%  **--------------------------------------------------------------------------------**

6 Selmo/1-334 93.9% 46.5%  **--------------------------------------------------------------------------------**

7 Selmo/1-443 99.4% 34.8%  **--------------------------------------------------------------------------------**

8 Sacu_v1.1_s0154.g023570/1-394 96.3% 42.8%  **--------------------------------------------------------------------------------**

9 Azfi_s0022.g016236/1-392 95.4% 41.4%  **--------------------------------------------------------------------------------**

10 Bradi4g40860/1-424 97.1% 38.0%  **--------------------------------------------------------------------------------**

11 AT5G63980_AtSAL1/1-353 97.7% 46.5%  **--------------------------------------------------------------------------------**

cov pid **241**  **: . . . . 3 . .** **320**

1 Cre01.g030250/1-347 100.0% 100.0%  **--------------------------------------------------------------------------------**

2 CHBRA172g00210/1-345 96.0% 47.6%  **--------------------------------------------------------------------------MALYQK**

3 AagrBONN/1-300 82.7% 46.7%  **---------------------------------------------------------------------------AAYEE**

4 Mapoly0030s0063/1-404 97.4% 42.1%  **---------------------------------------------------------------------------MAYED**

5 Pp3c15_5590/1-402 97.4% 44.0%  **---------------------------------------------------------------------------ASYHR**

6 Selmo/1-334 93.9% 46.5%  **-----------------------------------------------------------------------------MKR**

7 Selmo/1-443 99.4% 34.8%  **---------------------------------------------------------------------------GVYAQ**

8 Sacu_v1.1_s0154.g023570/1-394 96.3% 42.8%  **---------------------------------------------------------------------------PSYEQ**

9 Azfi_s0022.g016236/1-392 95.4% 41.4%  **---------------------------------------------------------------------------HNHDL**

10 Bradi4g40860/1-424 97.1% 38.0%  **--------------------------------------------------------------------------GSPYAA**

11 AT5G63980_AtSAL1/1-353 97.7% 46.5%  **---------------------------------------------------------------------------MAYEK**

cov pid **321**  **. . : . . . . 4** **400**

1 Cre01.g030250/1-347 100.0% 100.0%  **-MASAKEAVRLASRLCQEVQRQLS---AEERVDKKDDSPVT---------------------------------------**

2 CHBRA172g00210/1-345 96.0% 47.6%  **EVEMGCRAVRLAALLCQTVQRRLL---AEETAAKSDKSPVT---------------------------------------**

3 AagrBONN/1-300 82.7% 46.7%  **ELVLAVRAVKLASRLCQAVQRG-----------KADKSPVT---------------------------------------**

4 Mapoly0030s0063/1-404 97.4% 42.1%  **DVRLAIKAVSLASRLCQAVQRKLV---ANETQAKADQSPVT---------------------------------------**

5 Pp3c15_5590/1-402 97.4% 44.0%  **DVVLATNAVRLASRLCQTVQRGLL---TQETQTKSDKSPVT---------------------------------------**

6 Selmo/1-334 93.9% 46.5%  **ELIDRF----FFPGVWQSVQRKLL---ENETQSKTDNSPVT---------------------------------------**

7 Selmo/1-443 99.4% 34.8%  **ELEVAARAVQLGCMLAQRVQERIL-RKEENAGSKDDKSLVT---------------------------------------**

8 Sacu_v1.1_s0154.g023570/1-394 96.3% 42.8%  **DLHHAVGAVRLAARLCQTVQKNLL---SKETQAKADKSPVT---------------------------------------**

9 Azfi_s0022.g016236/1-392 95.4% 41.4%  **DIRRAVRAVRLAARLCQAVQKKLL---SEETQAKADKSPVT---------------------------------------**

10 Bradi4g40860/1-424 97.1% 38.0%  **ELAAAKKAVALAARLCQTVQQELV---QSDVQSKADKTPVT---------------------------------------**

11 AT5G63980_AtSAL1/1-353 97.7% 46.5%  **ELDAAKKAASLAARLCQKVQKALL---QSDVQSKSDKSPVT---------------------------------------**

cov pid **401**  **. . . . : . . .** **480**

1 Cre01.g030250/1-347 100.0% 100.0%  **------------VADYGAQVVVAWTLQRADPST-RLSMVAEEDSTELRTPAGRPMLDRITALVNSVVAAAA--PGEV---**

2 CHBRA172g00210/1-345 96.0% 47.6%  **------------VADYGSQALVSWALQTALPPGETLSLVAEEDSEDLRTESGAAMLQRITQLVNEAIKAED--GAQPAPG**

3 AagrBONN/1-300 82.7% 46.7%  **------------VADYGSQALVSWALQRELPAGVSFSMVAEEDSEDLRTKEGESMLERITQLVNTAI-------------**

4 Mapoly0030s0063/1-404 97.4% 42.1%  **------------VADYGSQALVSWVLERELPPG-TFHLIAEEDSEDLRAEDGSEMLKRITQLVNETIAADG--SFGS--T**

5 Pp3c15_5590/1-402 97.4% 44.0%  **------------VADYGSQALVNWSLAREFPPG-TFSMVAEEGSEDLRTEAGAPMLERITQLVNDAIASDA--ALDV--A**

6 Selmo/1-334 93.9% 46.5%  **------------IADYGSQAVVSWALERQLPAG-TFSMIAEEDSEDLRKEDGKAMLQRITELVNSIPSKDA---------**

7 Selmo/1-443 99.4% 34.8%  **------------VADWGVQAVVSWVLSQAFGEE--VSIIAEEDTKGLKGMNGIQTLQRVVAVVNECLSQASVVGLTPPSR**

8 Sacu_v1.1_s0154.g023570/1-394 96.3% 42.8%  **------------VADYGSQALVNWALERASPPG-TFSMVAEEDADDLRGEEGMSMLLRITDLVNEAIAAEG--GYIG--P**

9 Azfi_s0022.g016236/1-392 95.4% 41.4%  **------------VADYGSQALVNWALERETSTKETFSMVAEEDAASLCGEEGMDMLLRITDLVNETLAAEA--IYNA--P**

10 Bradi4g40860/1-424 97.1% 38.0%  **------------VADYGSQILVSLVLNMEVTSG-SFSMVAEEDSEDLRKEGAEEILEHITDLVNETLAEDG--SFNI---**

11 AT5G63980_AtSAL1/1-353 97.7% 46.5%  **------------VADYGSQAVVSLVLEKELSSE-PFSLVAEEDSGDLRKDGSQDTLERITKLVNDTLATEE--SFNG--S**

cov pid **481**  **. 5 . . . . : .** **560**

1 Cre01.g030250/1-347 100.0% 100.0%  **-LSPEQVLDIIDLGASQGGPS----GRHWVLDPIDGTRGFVGMRQ--YSVCLGMLQDGEVVLGVLGCPNLPQGPV----G**

2 CHBRA172g00210/1-345 96.0% 47.6%  **PLTPEDVATAIDRGLSNGGPL----GRHWVLDPIDGTKGFMRGEQ--YAVALGLLDEGEVVLGILGCPNLPMASI-----**

3 AagrBONN/1-300 82.7% 46.7%  **-LSKDEVVGAIDRG-SPGGPT----GRHWVLDPIDGTRGFVRGDQ--YAVALGLLDDGEVVAGVLGCPNLPL--------**

4 Mapoly0030s0063/1-404 97.4% 42.1%  **VLSEDDVLTAISRGDSPGGPS----GRYWVLDPIDGTRGFVRGDQ--YAVALGLLDNGEVVAGVLGCPNLPLASI-----**

5 Pp3c15_5590/1-402 97.4% 44.0%  **PLSKEDVLEAIDWGNSEGGSN----GRHWVLDPIDGTRGFVRGDQ--YAIALGLLDNGKVVAGVLGCPNLPMGSI-----**

6 Selmo/1-334 93.9% 46.5%  **VLSSEDVLCAIDRGKAEGGVQ----GRHWVLDPIDGTKGFLRGEQ--YAIALALLDRGSVVLGVLGCPNLPLSGL-----**

7 Selmo/1-443 99.4% 34.8%  **KLGTIEVLRAINKGNSEVRS-----SRSWVLDPVDGTLGFVRGDQ--YAIALGMIEDGRVVLGVLGCPNYPMRPQWLNYH**

8 Sacu_v1.1_s0154.g023570/1-394 96.3% 42.8%  **KLSKEDVLDAIDKGRSQGGEN----GRHWVLDPIDGTKGFLRGEQ--YAVALALLDNGEVVLGVLGCPNLPLKGV-----**

9 Azfi_s0022.g016236/1-392 95.4% 41.4%  **RLTTEDVLKAIDKGKSEGGKS----GRHWVLDPIDGTKG---GDQ--YAIALALLDNGEVVLGVLACPNLPLKGV-----**

10 Bradi4g40860/1-424 97.1% 38.0%  **SLSQEGILSAIDSGKSEGGPS----GRHWVLDPIDGTKGFVRGGQ--YAIALALLDEGKVVLGVLGCPNLPLTSI-----**

11 AT5G63980_AtSAL1/1-353 97.7% 46.5%  **TLSTDDLLRAIDCGTSEGGPN----GRHWVLDPIDGTKGFLRGDQ--YAVALGLLEEGKVVLGVLACPNLPLASI-----**

cov pid **561**  **. . . 6 . . . .** **640**

1 Cre01.g030250/1-347 100.0% 100.0%  **DDDGATGSAQRLSGDAD--VGCLFFSERGQG-AWVEPLQNAGDA-----APAQ-----------VRVAEVTEGAEARFME**

2 CHBRA172g00210/1-345 96.0% 47.6%  **--SVPGGVMAAATSGQP--VGCLFAARKGCG-ATVEPM--DCSL-----PPQK-----------VSVSKVSDPAWASFCE**

3 AagrBONN/1-300 82.7% 46.7%  **--------------------GCLFSARKGAG-TFMESL--------------------------VTVSDEDDTARATFCE**

4 Mapoly0030s0063/1-404 97.4% 42.1%  **--AANG----AASDDKA--VGCVFAASRGAG-TVMQSM--DGSR-----EAQR-----------VYVSSVDDSKLAAFCE**

5 Pp3c15_5590/1-402 97.4% 44.0%  **--ANGI----PANSSEP--VGCLFVASLGAG-TTVEPL--DGSG-----EPKR-----------VHVSDVEDTAIATFCE**

6 Selmo/1-334 93.9% 46.5%  **--SD--------DGSSP--VGCLFTAVRGAG-TTVHAI--DRSV-----QPRK-----------VRVSDLSDPALAAFCE**

7 Selmo/1-443 99.4% 34.8%  **QKYYRLASKIAPPPPGKWHKGCVLTSAKGAGQAWMEPLVWNSDGSFLLNPPRV-----------VAVSPVDDPAQATFCE**

8 Sacu_v1.1_s0154.g023570/1-394 96.3% 42.8%  **--------TVQDSES----IGCLFSARKGAG-TMLQSL--DGLV-----TPKR-----------VHVSDVDDPLLARFCE**

9 Azfi_s0022.g016236/1-392 95.4% 41.4%  **----------SIQDSEP--VGCLFSARKGEG-TMLQSL--DGRF-----TPKKACDASSYTYLVVHVSDVDDPALATFCE**

10 Bradi4g40860/1-424 97.1% 38.0%  **--CN----LNGNSSGDQ--TGVLFSATIGCG-AEVQSL--DG-S-----PPQK-----------ISVCSIDNPVNASFFE**

11 AT5G63980_AtSAL1/1-353 97.7% 46.5%  **--AGN---NKNKSSSDE--IGCLFFATIGSG-TYMQLL--DSKS-----SPVK-----------VQVSSVENPEEASFFE**

cov pid **641**  **: . . . . 7 . .** **720**

1 Cre01.g030250/1-347 100.0% 100.0%  **SVESRHSSHSINAALARELG--------VVRPPLRMDSQVKYGLLSRGCGTIFMRFPPATYKEKIWDHAAGFVIVEEAGG**

2 CHBRA172g00210/1-345 96.0% 47.6%  **SYESAHSLQDLTANIARVLG--------VTSPPVRIDSQAKYGAMARGDAAIYLRFPHLGYREKIWDHCAGAMVIQEAGG**

3 AagrBONN/1-300 82.7% 46.7%  **SFESAHS---LTANIAKLLG--------VIAPPVRIDSQAKYGAMARGDAVIYLR-------EKIWDHAAGAIVITEAGG**

4 Mapoly0030s0063/1-404 97.4% 42.1%  **SFEAAHSKQGLTANIARILG--------VSAPPIRIDSQAKYGAMARGDAVIYLRFPHAGYREKIWDHAAGLIVITEAGG**

5 Pp3c15_5590/1-402 97.4% 44.0%  **SYESAHTMQDLTANIAGTLG--------VKAPPVRIDSQAKYGAMARGDAVIYLRFPHFGYREKIWDHAAGAIVITEAGG**

6 Selmo/1-334 93.9% 46.5%  **SYESAHSKHDLTSGIAKMLG--------VTASPIRMDSQAKYGAMARGDAAIYLRFPHKGYREKIWDHAAGSIVVEEAGG**

7 Selmo/1-443 99.4% 34.8%  **PVEKANSSHSFTEGVADSLG--------LRKRPLRVYSMAKYAAIARGDAEIFMKFARAGYKEKIWDHAAGVLIVEEAGG**

8 Sacu_v1.1_s0154.g023570/1-394 96.3% 42.8%  **SYESAHSRQNLAERIAKIMG--------VSAPPVRIDSQAKYGAMARGDAAIYLRFPHPGYREKIWDHAAGCIVIEEAGG**

9 Azfi_s0022.g016236/1-392 95.4% 41.4%  **SFESAHSLQNLTGNIAKIIG--------VTAPPVRIDSQAKYGAMARGDAAIYLRFPHPGYREKIWDHAAGCIVIEEAGG**

10 Bradi4g40860/1-424 97.1% 38.0%  **SYEGAHNMRDVTGSIAEKLG--------VQAPPVRIDSQAKYGALARGDGAIYLRFPHKGYKEKIWDHAAGSIVVTEAGG**

11 AT5G63980_AtSAL1/1-353 97.7% 46.5%  **SFEGAHSLHDLSSSIANKLG--------VKAPPVRIDSQAKYGALSRGDGAIYLRFPHKGYREKIWDHVAGAIVVTEAGG**

cov pid **721**  **. . : . . . ]** **788**

1 Cre01.g030250/1-347 100.0% 100.0%  **RVTDAAGVRLDFSKG---RFL-ALDRGIIAA-PPALHEKLVAAAAKVAPKA-----------------**

2 CHBRA172g00210/1-345 96.0% 47.6%  **VVVDAAGRPLDFSKD-------SAARG-----TAPLRKLHFSRGSDIPS-------------------**

3 AagrBONN/1-300 82.7% 46.7%  **EVFDAAGQPLDFSRG---RFL-DLE-GIIAT-NSKLKPRVLAAVQAALKAQQLSSKV-----------**

4 Mapoly0030s0063/1-404 97.4% 42.1%  **EVFDAAGKPLDFSMG---RFL-DLEKGIIAT-NPSLRSAVLAAVQTAIKEEESALKGVSPL-------**

5 Pp3c15_5590/1-402 97.4% 44.0%  **EVFDAAGEPLDFSRG---RWL-DLDTGIIAT-NKELKPVVLSAVQKCVKDLKIPAKH-----------**

6 Selmo/1-334 93.9% 46.5%  **VVVDAAGRALDFSKG---RYL-DLDTGIIAT-NPSLLTAVLTAVETCVGSKSFL--------------**

7 Selmo/1-443 99.4% 34.8%  **VVTDAGGRPLDFSKG---RYVEGLDRGIIVSCGTGLHRRIISAVDASWNSSKL---------------**

8 Sacu_v1.1_s0154.g023570/1-394 96.3% 42.8%  **VVVDATGKKLDFSKG---RYL-DVNHGIMAT-NSKLLQRLLAAAQAAIEAEK----------------**

9 Azfi_s0022.g016236/1-392 95.4% 41.4%  **VVVDASGKKLDFSKG---RYL-DVEHGIIAT-NPKLMAQVLAAAQIAIKGDK----------------**

10 Bradi4g40860/1-424 97.1% 38.0%  **IVTDASGKDLDFSKG---RCLDDLDTGIVAT-NKQLMPSLLKAVQEAIKEKNQAPSPL----------**

11 AT5G63980_AtSAL1/1-353 97.7% 46.5%  **IVTDAAGKPLDFSKG---KYL-DLDTGIIVA-NEKLMPLLLKAVRDSIAEQEKASAL-----------**

>Cre01.g030250/1-347

------------------------------------------------------------------------

------------------------------------------------------------------------

------------------------------------------------------------------------

------------------------------------------------------------------------

---------------------------------MASAKEAVRLASRLCQEVQRQLS---AEERVDKKDDSPV

T---------------------------------------------------VADYGAQVVVAWTLQRADPS

T-RLSMVAEEDSTELRTPAGRPMLDRITALVNSVVAAAA--PGEV----LSPEQVLDIIDLGASQGGPS---

-GRHWVLDPIDGTRGFVGMRQ--YSVCLGMLQDGEVVLGVLGCPNLPQGPV----GDDDGATGSAQRLSGDA

D--VGCLFFSERGQG-AWVEPLQNAGDA-----APAQ-----------VRVAEVTEGAEARFMESVESRHSS

HSINAALARELG--------VVRPPLRMDSQVKYGLLSRGCGTIFMRFPPATYKEKIWDHAAGFVIVEEAGG

RVTDAAGVRLDFSKG---RFL-ALDRGIIAA-PPALHEKLVAAAAKVAPKA-----------------

>CHBRA172g00210/1-345

------------------------------------------------------------------------

------------------------------------------------------------------------

------------------------------------------------------------------------

------------------------------------------------------------------------

--------------------------MALYQKEVEMGCRAVRLAALLCQTVQRRLL---AEETAAKSDKSPV

T---------------------------------------------------VADYGSQALVSWALQTALPP

GETLSLVAEEDSEDLRTESGAAMLQRITQLVNEAIKAED--GAQPAPGPLTPEDVATAIDRGLSNGGPL---

-GRHWVLDPIDGTKGFMRGEQ--YAVALGLLDEGEVVLGILGCPNLPMASI-------SVPGGVMAAATSGQ

P--VGCLFAARKGCG-ATVEPM--DCSL-----PPQK-----------VSVSKVSDPAWASFCESYESAHSL

QDLTANIARVLG--------VTSPPVRIDSQAKYGAMARGDAAIYLRFPHLGYREKIWDHCAGAMVIQEAGG

VVVDAAGRPLDFSKD-------SAARG-----TAPLRKLHFSRGSDIPS-------------------

>AagrBONN/1-300 Sc2ySwM_117.3377.1

------------------------------------------------------------------------

------------------------------------------------------------------------

------------------------------------------------------------------------

------------------------------------------------------------------------

---------------------------AAYEEELVLAVRAVKLASRLCQAVQRG-----------KADKSPV

T---------------------------------------------------VADYGSQALVSWALQRELPA

GVSFSMVAEEDSEDLRTKEGESMLERITQLVNTAI--------------LSKDEVVGAIDRG-SPGGPT---

-GRHWVLDPIDGTRGFVRGDQ--YAVALGLLDDGEVVAGVLGCPNLPL------------------------

----GCLFSARKGAG-TFMESL--------------------------VTVSDEDDTARATFCESFESAHS-

--LTANIAKLLG--------VIAPPVRIDSQAKYGAMARGDAVIYLR-------EKIWDHAAGAIVITEAGG

EVFDAAGQPLDFSRG---RFL-DLE-GIIAT-NSKLKPRVLAAVQAALKAQQLSSKV-----------

>Mapoly0030s0063/1-404

-------------------------------------------MQALGARRAFLRSSFPCSRSSSSWKALGN

CRAPSIVRLGRRGVGAIRS-----------------------------------------------------

------------------------------------------------------------------------

------------------------------------------------------------------------

---------------------------MAYEDDVRLAIKAVSLASRLCQAVQRKLV---ANETQAKADQSPV

T---------------------------------------------------VADYGSQALVSWVLERELPP

G-TFHLIAEEDSEDLRAEDGSEMLKRITQLVNETIAADG--SFGS--TVLSEDDVLTAISRGDSPGGPS---

-GRYWVLDPIDGTRGFVRGDQ--YAVALGLLDNGEVVAGVLGCPNLPLASI-------AANG----AASDDK

A--VGCVFAASRGAG-TVMQSM--DGSR-----EAQR-----------VYVSSVDDSKLAAFCESFEAAHSK

QGLTANIARILG--------VSAPPIRIDSQAKYGAMARGDAVIYLRFPHAGYREKIWDHAAGLIVITEAGG

EVFDAAGKPLDFSMG---RFL-DLEKGIIAT-NPSLRSAVLAAVQTAIKEEESALKGVSPL-------

>Pp3c15_5590/1-402

------------------------------------------MLKEVVHRSHHLWQCFPRHLHCHDWSPVNS

KARSRSGRGSVSWRKPLFCM----------------------------------------------------

------------------------------------------------------------------------

------------------------------------------------------------------------

---------------------------ASYHRDVVLATNAVRLASRLCQTVQRGLL---TQETQTKSDKSPV

T---------------------------------------------------VADYGSQALVNWSLAREFPP

G-TFSMVAEEGSEDLRTEAGAPMLERITQLVNDAIASDA--ALDV--APLSKEDVLEAIDWGNSEGGSN---

-GRHWVLDPIDGTRGFVRGDQ--YAIALGLLDNGKVVAGVLGCPNLPMGSI-------ANGI----PANSSE

P--VGCLFVASLGAG-TTVEPL--DGSG-----EPKR-----------VHVSDVEDTAIATFCESYESAHTM

QDLTANIAGTLG--------VKAPPVRIDSQAKYGAMARGDAVIYLRFPHFGYREKIWDHAAGAIVITEAGG

EVFDAAGEPLDFSRG---RWL-DLDTGIIAT-NKELKPVVLSAVQKCVKDLKIPAKH-----------

>Selmo/1-334 97708

------------------------------------------------------------------------

------------------------------------------------------------------------

------------------------------------------------------------------------

------------------------------------------------------------------------

-----------------------------MKRELIDRF----FFPGVWQSVQRKLL---ENETQSKTDNSPV

T---------------------------------------------------IADYGSQAVVSWALERQLPA

G-TFSMIAEEDSEDLRKEDGKAMLQRITELVNSIPSKDA---------VLSSEDVLCAIDRGKAEGGVQ---

-GRHWVLDPIDGTKGFLRGEQ--YAIALALLDRGSVVLGVLGCPNLPLSGL-------SD--------DGSS

P--VGCLFTAVRGAG-TTVHAI--DRSV-----QPRK-----------VRVSDLSDPALAAFCESYESAHSK

HDLTSGIAKMLG--------VTASPIRMDSQAKYGAMARGDAAIYLRFPHKGYREKIWDHAAGSIVVEEAGG

VVVDAAGRALDFSKG---RYL-DLDTGIIAT-NPSLLTAVLTAVETCVGSKSFL--------------

>Selmo/1-443 111780

------------------------MLGNFDSLREISRNARGWSVGSLACPRARSWRPSAVSSETTGNIDSRA

QPRPQCLEHNRDGNDKKITR----------------------------------------------------

------------------------------------------------------------------------

------------------------------------------------------------------------

---------------------------GVYAQELEVAARAVQLGCMLAQRVQERIL-RKEENAGSKDDKSLV

T---------------------------------------------------VADWGVQAVVSWVLSQAFGE

E--VSIIAEEDTKGLKGMNGIQTLQRVVAVVNECLSQASVVGLTPPSRKLGTIEVLRAINKGNSEVRS----

-SRSWVLDPVDGTLGFVRGDQ--YAIALGMIEDGRVVLGVLGCPNYPMRPQWLNYHQKYYRLASKIAPPPPG

KWHKGCVLTSAKGAGQAWMEPLVWNSDGSFLLNPPRV-----------VAVSPVDDPAQATFCEPVEKANSS

HSFTEGVADSLG--------LRKRPLRVYSMAKYAAIARGDAEIFMKFARAGYKEKIWDHAAGVLIVEEAGG

VVTDAGGRPLDFSKG---RYVEGLDRGIIVSCGTGLHRRIISAVDASWNSSKL---------------

>Sacu_v1.1_s0154.g023570/1-394

----------------------------------MEELILVHNVLYIVCLRIAMQQLNFRLYSRIPIAPPFL

CSRSSVSRTTMSL-----------------------------------------------------------

------------------------------------------------------------------------

------------------------------------------------------------------------

---------------------------PSYEQDLHHAVGAVRLAARLCQTVQKNLL---SKETQAKADKSPV

T---------------------------------------------------VADYGSQALVNWALERASPP

G-TFSMVAEEDADDLRGEEGMSMLLRITDLVNEAIAAEG--GYIG--PKLSKEDVLDAIDKGRSQGGEN---

-GRHWVLDPIDGTKGFLRGEQ--YAVALALLDNGEVVLGVLGCPNLPLKGV-------------TVQDSES-

---IGCLFSARKGAG-TMLQSL--DGLV-----TPKR-----------VHVSDVDDPLLARFCESYESAHSR

QNLAERIAKIMG--------VSAPPVRIDSQAKYGAMARGDAAIYLRFPHPGYREKIWDHAAGCIVIEEAGG

VVVDATGKKLDFSKG---RYL-DVNHGIMAT-NSKLLQRLLAAAQAAIEAEK----------------

>Azfi_s0022.g016236/1-392

-----------------------------------MQYLRGNTLLVYNFPRSSLNSLCRCSYTCRTTTTMSL

PST---------------------------------------------------------------------

------------------------------------------------------------------------

------------------------------------------------------------------------

---------------------------HNHDLDIRRAVRAVRLAARLCQAVQKKLL---SEETQAKADKSPV

T---------------------------------------------------VADYGSQALVNWALERETST

KETFSMVAEEDAASLCGEEGMDMLLRITDLVNETLAAEA--IYNA--PRLTTEDVLKAIDKGKSEGGKS---

-GRHWVLDPIDGTKG---GDQ--YAIALALLDNGEVVLGVLACPNLPLKGV---------------SIQDSE

P--VGCLFSARKGEG-TMLQSL--DGRF-----TPKKACDASSYTYLVVHVSDVDDPALATFCESFESAHSL

QNLTGNIAKIIG--------VTAPPVRIDSQAKYGAMARGDAAIYLRFPHPGYREKIWDHAAGCIVIEEAGG

VVVDASGKKLDFSKG---RYL-DVEHGIIAT-NPKLMAQVLAAAQIAIKGDK----------------

>Bradi4g40860/1-424

----------------------------MAARVGLSHALLASSSLARNPPPRARLLPYPTLLPLPTYRRSVS

ASASALPSLRRRRPFAARAMSQPPEAA---------------------------------------------

------------------------------------------------------------------------

------------------------------------------------------------------------

--------------------------GSPYAAELAAAKKAVALAARLCQTVQQELV---QSDVQSKADKTPV

T---------------------------------------------------VADYGSQILVSLVLNMEVTS

G-SFSMVAEEDSEDLRKEGAEEILEHITDLVNETLAEDG--SFNI---SLSQEGILSAIDSGKSEGGPS---

-GRHWVLDPIDGTKGFVRGGQ--YAIALALLDEGKVVLGVLGCPNLPLTSI-------CN----LNGNSSGD

Q--TGVLFSATIGCG-AEVQSL--DG-S-----PPQK-----------ISVCSIDNPVNASFFESYEGAHNM

RDVTGSIAEKLG--------VQAPPVRIDSQAKYGALARGDGAIYLRFPHKGYKEKIWDHAAGSIVVTEAGG

IVTDASGKDLDFSKG---RCLDDLDTGIVAT-NKQLMPSLLKAVQEAIKEKNQAPSPL----------

>AT5G63980_AtSAL1/1-353

------------------------------------------------------------------------

------------------------------------------------------------------------

------------------------------------------------------------------------

------------------------------------------------------------------------

---------------------------MAYEKELDAAKKAASLAARLCQKVQKALL---QSDVQSKSDKSPV

T---------------------------------------------------VADYGSQAVVSLVLEKELSS

E-PFSLVAEEDSGDLRKDGSQDTLERITKLVNDTLATEE--SFNG--STLSTDDLLRAIDCGTSEGGPN---

-GRHWVLDPIDGTKGFLRGDQ--YAVALGLLEEGKVVLGVLACPNLPLASI-------AGN---NKNKSSSD

E--IGCLFFATIGSG-TYMQLL--DSKS-----SPVK-----------VQVSSVENPEEASFFESFEGAHSL

HDLSSSIANKLG--------VKAPPVRIDSQAKYGALSRGDGAIYLRFPHKGYREKIWDHVAGAIVVTEAGG

IVTDAAGKPLDFSKG---KYL-DLDTGIIVA-NEKLMPLLLKAVRDSIAEQEKASAL-----------

**Acetohydroxy acid isomeroreductase**

cov pid  **1** **[ . . . . : . . .** **80**

1 157966/1-517 100.0% 100.0%  **--------------------------------------------------------------------------------**

2 AT3G58610/1-591 100.0% 67.2%  **MAAATSSIAPSLSCPSPSSSS-------KTLWSSKARTLALPNIGFLSSSSKSLR-------------SLTATVAGNGAT**

3 Bradi2g15790/1-581 100.0% 68.3%  **------MAASTISFSHPKTLA---------AAAAVPKTLPIPTASSVAFPASHP--------------AWVLSAARRRAV**

4 Bradi2g45330/1-579 100.0% 68.7%  **-MAAATSSASSLAFSHPKTLN---------------PASKTPAVGSVSFPAAQ---------------TPCILASSAGRC**

5 Cre10.g434750/1-555 98.6% 57.1%  **--MQLLNSKSRVLSGSRQQAA-----------------------------------------------AKAVRVAPSGRR**

6 Mapoly0019s0065/1-595 100.0% 69.6%  **MAAASCTAALSSSCVVLHSKDAGAVSRNGAMQQQIRSFDGLHRHQLLIAKTAEKCL------------RAAVPAARNGRK**

7 Pp3c19_22360/1-589 100.0% 68.4%  **MAAVTLSHCAAPSSSVAHRSS----EVLGSAGPKMTSFAGLRSVAFAPKLEKSL--------------RNAVAAVPCWRR**

8 Pp3c21_20900/1-590 100.0% 69.3%  **MAAVTLSHCATASAGVAHRSS---ETVVGSANAKMASFSGLRIVAFAPKLEKSL--------------SAAVAAVPSRMR**

9 Sacu_v1.1_s0008.g004218/1-581 100.0% 69.7%  **-MACSAAAAAASSTSLASTFN------------AVSSRLPAPRASTAKLSFSRSV-------------SESSSIAPASGN**

10 G28546/1-603 92.1% 57.8%  **MAAAVSQGISSQVFSIAARQDQSAVLASTSSSSDCAATLVIPRFTGLQKSSTSAIAAGRHVSHREGFSKQAQRLSRRTRA**

11 Anthoceros/1-326 63.1% 82.8%  **--------------------------------------------------------------------------------**

12 Anthoceros/1-290 55.7% 44.2%  **--------------------------------------------------------------------------------**

13 Anthoceros/1-193 35.8% 26.2%  **--------------------------------------------------------------------------------**

cov pid  **81**  **. 1 . . . . : .** **160**

1 157966/1-517 100.0% 100.0%  **----------MVATRG-AEVEFETKVFTKEKVTLGGKDEFIVRGGRHLFEKL--PEAFKGIKQIGVLGWGSQGPAQAQNL**

2 AT3G58610/1-591 100.0% 67.2%  **GSSLAARMVSSSAVKAPVSLDFETSVFKKEKVSLAGYEEYIVRGGRDLFKHL--PDAFKGIKQIGVIGWGSQGPAQAQNL**

3 Bradi2g15790/1-581 100.0% 68.3%  **AAMVAAPTTTASVGAAMPSLDFDTSVFNKEKVSLAGHEEYIVRGGRNLFPLL--HEAFKGVKQIGVIGWGSQGPAQAQNL**

4 Bradi2g45330/1-579 100.0% 68.7%  **RAVVAKVASPSVIGATMPSLDFETSVFKKEKVSLAGHDEYIVRGGRNLFPLL--PEAFKGIKQIGVLGWGSQGPAQAQNL**

5 Cre10.g434750/1-555 98.6% 57.1%  **S---------AVRVSAAVHLDFNTKVFQKEHAKFGPTEEYIVRGGRDKYPLL--KEAFKGIKKVSVIGWGSQAPAQAQNL**

6 Mapoly0019s0065/1-595 100.0% 69.6%  **GGAMCTQAVAMPATRG-TEVVFESKVFKKEKITLAGGDEYIVRGGRDLFPLL--PKALQGIKKIGVIGWGSQGPAQAQNF**

7 Pp3c19_22360/1-589 100.0% 68.4%  **GGAMSINMVATPAVRG-VDVEFQTEIFKKEKITPAGRDEYIVRGGRDLFHLL--PKALTGIKKIGVIGWGSQGPAQAMNI**

8 Pp3c21_20900/1-590 100.0% 69.3%  **GGAIAASMVASPALRG-ADVEFQSEVFKKEKITPAGRDEYIVRGGRDLFHLL--PEAFKGIKKIGVIGWGSQGPAQAMNI**

9 Sacu_v1.1_s0008.g004218/1-581 100.0% 69.7%  **VGRVRMTVATRTADRG-IDVQFDSAIFKKEKITLAGRDEYIVRGGRNLFYLL--PEAFKGIKQIGVIGWGSQAPAQAQNL**

10 G28546/1-603 92.1% 57.8%  **GGAVTVEMVVAVPSANKVESDFETKVFKKEKITLAGNDEYIVRGGRDLFHLL--SKAFQGIKKIGVIGWGSQGPAQSQNL**

11 Anthoceros/1-326 63.1% 82.8%  **-------------------------------------MQYIVRGGRDLFELL--PKAFKGIKQIGVIGWGSQGPAQAQNL**

12 Anthoceros/1-290 55.7% 44.2%  **------------------------------MTVPATSHQLHITASRPLFISMHNPTSKFKLQHKGIV-------------**

13 Anthoceros/1-193 35.8% 26.2%  **--------------------------------------------------------------------------------**

cov pid **161**  **. . . 2 . . . .** **240**

1 157966/1-517 100.0% 100.0%  **RDSLEAVKSDIVVKIGLRKGSKSAADARAAGFTEEN----GTLG-DVIETVAGSDLVLLLISDAAQADNFREIFAALKPK**

2 AT3G58610/1-591 100.0% 67.2%  **RDSLVEAKSDIVVKIGLRKGSRSFEEARAAGFTEES----GTLG-DIWETIAGSDLVLLLISDAAQADNYEKIFSHMKPN**

3 Bradi2g15790/1-581 100.0% 68.3%  **RDSLAEAKSDIVVKIGLRKGSKSFEEARGAGFSEEN----GTLG-DIWETISGSDLVLLLISDSAQADNYEKIFSHMKPN**

4 Bradi2g45330/1-579 100.0% 68.7%  **RDSLAEAKSDIVVKIGLRKGSKSFQDARAAGFTEEN----GTLG-DIWETVSSSDLLLLLISDAAQADNYEKIFSHMKPN**

5 Cre10.g434750/1-555 98.6% 57.1%  **RDSIAEAGMDIKVAIGLRPDSPSWAEAEACGFSKTD----GTLG-EVFEQISSSDFVILLISDAAQAKLYPRILAAMKPG**

6 Mapoly0019s0065/1-595 100.0% 69.6%  **RDSLNAIGSDIVVKVGLRKGSKSCQEARAAGFTEET----GTLG-DVLETVSESDLVMLLISDAAQADNYKEIFAAMKDN**

7 Pp3c19_22360/1-589 100.0% 68.4%  **RDSLAEIKSDIVVKIGLRKGSKSCEDARSVGFTEES----GTLG-DVIETVAESDLVLLLISDAAQADNYKEIFKAMKPN**

8 Pp3c21_20900/1-590 100.0% 69.3%  **RDSLAEIKSDIVVKIGLRKGSKSCADARAVGFTEES----GTLG-DVLETVAESDLVLLLISDAAQADNYQDIFKAMKPK**

9 Sacu_v1.1_s0008.g004218/1-581 100.0% 69.7%  **RDSLLEAKSNIKVKIGLRKGSKSVSEARAAGFTEES----GTLG-DVLDTVSESDLVLLLISDAAQADNYKQIFAAMKPK**

10 G28546/1-603 92.1% 57.8%  **RDSLEAANSDIVVKVGLRTNSKSRVEARNAGFTEEN----GTLG-DVLEVVKESDLVLLLISDAAQADEYESILGAMKPG**

11 Anthoceros/1-326 63.1% 82.8%  **RDSLADAKSDIVVKIGLRKGSKSVAEAREAGFTEAD----GTLG-DVVETVSGSDLVLLLISDAAQADNYKEVFAALKPK**

12 Anthoceros/1-290 55.7% 44.2%  **--------------IGLRKGSKSVAEASEAGFTEAD----GTLG-DVVETVSGSDLVLLLISDAAQAYNYKEIFSALKAK**

13 Anthoceros/1-193 35.8% 26.2%  **----------------MTRGSKLVAEAREAGYTEADRDAWGCRGYCVWQRFGPAADYGMPHGQITTGN-----LLCSEAE**

cov pid **241**  **: . . . . 3 . .** **320**

1 157966/1-517 100.0% 100.0%  **AILGLSHGFLLGHLESIGEKFPKDISVIAVCPKGMGPSVRRLYVQGKEVNGAGINASFAVHQDVDGRATDVALGWSVALG**

2 AT3G58610/1-591 100.0% 67.2%  **SILGLSHGFLLGHLQSSGLDFPKNISVVAVCPKGMGPSVRRLYVQGKEINGAGINASFAVHQDVDGRAADVALGWSVALG**

3 Bradi2g15790/1-581 100.0% 68.3%  **SILGLSHGFLLGHLQSHGLDFPKNISVVAVCPKGMGPSVRRLYVQGKEVNGAGINASFAVHQDVDGRATDVALGWSVALG**

4 Bradi2g45330/1-579 100.0% 68.7%  **SILGLSHGFLLGHLQSVGLDFPQNISVIAVCPKGMGPSVRRLYVQGKEVNGAGINSSFAVHQDVDGRATDVALGWSIALG**

5 Cre10.g434750/1-555 98.6% 57.1%  **ATLGLSHGFLLGVMRNDGVDFRKDINVVLVAPKGMGPSVRRLYEQGKSVNGAGINCSFAIQQDATGQAADIAIGWAIGVG**

6 Mapoly0019s0065/1-595 100.0% 69.6%  **AILGLSHGFLLGHLQSLREEFPKNISVIAVCPKGMGPSVRRLYVQGKEVNGAGINASFAVHQDVDGRATDVALGWSVALG**

7 Pp3c19_22360/1-589 100.0% 68.4%  **SVLGLSHGFLLGHLNSVGDSFPDDISVIAVCPKGMGPSVRRLYVQGKEVNGAGINASFAVHQDVDGRATDVALGWSIALG**

8 Pp3c21_20900/1-590 100.0% 69.3%  **SVLGLSHGFLLGHLNSVNDSFPEDISVIAVCPKGMGPSVRRLYVQGKEVNGAGINASFAVHQDVDGRATDVALGWSVALG**

9 Sacu_v1.1_s0008.g004218/1-581 100.0% 69.7%  **SILGLSHGFLLGHLQSVREDFRKDISVIAVCPKGMGPSVRRLYVQGKEINGAGINSSFAVHQDVDGRASDVALGWSVALG**

10 G28546/1-603 92.1% 57.8%  **SCLGLSHGFLLGHMKSVGDDFRKDISVVAVCPKGMGPSVRRLYEQGKEVNGAGINASFAVHQDVDGRATDIALGWSVALG**

11 Anthoceros/1-326 63.1% 82.8%  **SILGLSHGFLLGHLQSTGDTFPKDISVIAVCPKGMGPSVRRLYVQGKQINGAGINSSFAVHQDADGRATDVALGWSVALG**

12 Anthoceros/1-290 55.7% 44.2%  **AILGLSHGFLLGHLQSIGDTFPKDMSVIAVCPKGMGLSVRRLYVQGKQINGAGINSSFAVHQDDDGKAADVAL-------**

13 Anthoceros/1-193 35.8% 26.2%  **VCFGTVSWFPPGTSAVNLDPFPKDISVMAFCPKGTGSSVRRP---GQANRWSWDQLELAVHQDADGRATDIALGWS---R**

cov pid **321**  **. . : . . . . 4** **400**

1 157966/1-517 100.0% 100.0%  **SPFTFVTTLEDEYKSDIYGERGILLGAVHGLVEALYRRYTGNG------------------------------------M**

2 AT3G58610/1-591 100.0% 67.2%  **SPFTFATTLEQEYRSDIFGERGILLGAVHGIVESLFRRYTENG------------------------------------M**

3 Bradi2g15790/1-581 100.0% 68.3%  **SPFTFATTLEQEYKSDIFGERGILLGAVHGIVEALFRRYTEQG------------------------------------M**

4 Bradi2g45330/1-579 100.0% 68.7%  **SPFTFATTLEQEYRSDIFGERGILLGAVHGIVEALFRRYTEQG------------------------------------M**

5 Cre10.g434750/1-555 98.6% 57.1%  **APFAFPTTLESEYKSDIYGERCVLLGAVHGIVEALFRRYTRQG------------------------------------M**

6 Mapoly0019s0065/1-595 100.0% 69.6%  **SPFTFVTTLEDEYKSDIFGERGILLGAVHGIVESLFRRYTSNG------------------------------------M**

7 Pp3c19_22360/1-589 100.0% 68.4%  **SPFTFATTLEDEYKSDIYGERGILLGAVHGIVESLFRRYTAQG------------------------------------M**

8 Pp3c21_20900/1-590 100.0% 69.3%  **SPFTFATTLEDEYKSDIYGERGILLGAVHGIVESLFRRYTACG------------------------------------M**

9 Sacu_v1.1_s0008.g004218/1-581 100.0% 69.7%  **SPFTFATTLEDEYKSDIYGERGILLGAVHGLVEALFRRYTDAG------------------------------------M**

10 G28546/1-603 92.1% 57.8%  **SPFTFRTTLESEYKSDIYGERGILLGAVHGVVEALFRRYTEQGMSDEDAFMMIFWLLSILLGAVHGVVEALFRRYTEQGM**

11 Anthoceros/1-326 63.1% 82.8%  **SPFTFVTTLEDEYRSDIYGERGILLGAVHGIVEALFRRYTENG------------------------------------M**

12 Anthoceros/1-290 55.7% 44.2%  **-------------RSDIYGERGIRLGAVHGIVAALFRRYTENG------------------------------------M**

13 Anthoceros/1-193 35.8% 26.2%  **EAFSWVQLL------------------------ALFRSYTENG---------------------------------ENRT**

cov pid **401**  **. . . . : . . .** **480**

1 157966/1-517 100.0% 100.0%  **SEEAAYKNTVESVTGTISKTISSKGMVSLYRSLSDEGKKEFEAAFCASYYPAMDVLYEIYDEVSSGNEIRSVVLAGRRFS**

2 AT3G58610/1-591 100.0% 67.2%  **SEDLAYKNTVECITGTISRTISTQGMLAVYNSLSEEGKKDFETAYSASFYPCMEILYECYEDVQSGSEIRSVVLAGRRFY**

3 Bradi2g15790/1-581 100.0% 68.3%  **DEALTYKSTVEGITGIISKTISKKGMLEVYNSLSEEGKKEFNKAYSASFYPCMDILYECYEDVASGSEIRSVVLAGRRFY**

4 Bradi2g45330/1-579 100.0% 68.7%  **DEVLAYKNTVECITGIVSKTISKKGMLEVYNSFTEEGKKQFKEAYSAAFYPSMDILYECYEDVSSGSEIRSVVLAGRRFY**

5 Cre10.g434750/1-555 98.6% 57.1%  **SDEEAFKQSVESITGPISRTISTKGMLSVYNSFNEADKKIFEQAYSASYKPALDICFEIYEDVASGNEIKSVVQAVQRFD**

6 Mapoly0019s0065/1-595 100.0% 69.6%  **QEEAAYKNTVESITGIISRTISAKGMLAVYRSLSDEGKKEFEAAYSASYLPAMDILYECYEDVASGNEIRSVVLAGRRFS**

7 Pp3c19_22360/1-589 100.0% 68.4%  **SEEDAYKNTVEGITGVISKIISTKGILAVYEALSEEGKKEFEAAYSASFYPSMDILYECYEDVASGNEIRSVVLAGRRFS**

8 Pp3c21_20900/1-590 100.0% 69.3%  **SEEDAYKNTVESITGVISKTISTQGILAVYESLSEEGKKEFEAAYSASFYPSMDILYECYEDVASGNEIRSVVLAGRRFS**

9 Sacu_v1.1_s0008.g004218/1-581 100.0% 69.7%  **PEEDAYKNTVESITGIISKTISTKGMKAVYNSLDEQGKRDFKVAYSASYYPCMDILYECYEDVASGNEIRSVVLAGRRFQ**

10 G28546/1-603 92.1% 57.8%  **SDEDAFTNSVESITGVISQTISRKGMLAVYESLSKEGKREFEAAYSASYLPAMDILYECYEDVESGNEIRSVVLAGRRFR**

11 Anthoceros/1-326 63.1% 82.8%  **AEDAAYKNTVESVTGVISRIISTKGMLAVYRSLSDEGKKEFEAAYSASYLPSMDILYECYEDVASGNEIRSVVLAGRRFS**

12 Anthoceros/1-290 55.7% 44.2%  **AEDAAYKNTVESAAGVISRIISTKGMLVVYEFLT------------------------------SGNEIRSVVLAGRRFP**

13 Anthoceros/1-193 35.8% 26.2%  **AEDAAYKNTVESVSRIISTKLRSFGL------------------------------------------------------**

cov pid **481**  **. 5 . . . . : .** **560**

1 157966/1-517 100.0% 100.0%  **ARGGLPAFPMGTIDQTHMWKVGEKVRAARPEGDLGPLHPFTAGVYVAVMMAQIEILRNKGHSYSEMVNESIIEAVDSLNP**

2 AT3G58610/1-591 100.0% 67.2%  **EKEGLPAFPMGNIDQTRMWKVGERVRKSRPAGDLGPLYPFTAGVYVALMMAQIEILRKKGHSYSEIINESVIESVDSLNP**

3 Bradi2g15790/1-581 100.0% 68.3%  **DKEGLPAFPMGKIDQTRMWKVGEKVRLTRPDGDLGPLHPFTAGVYVALMMAQIEVLRKKGHSYSEIINESVIESVDSLNP**

4 Bradi2g45330/1-579 100.0% 68.7%  **EKEGLPAFPMGNIDQTRMWKVGERVRATRPQGDLGPLHPFTAGVFVALMMAQIEVLRKKGHSYSEIINESVIESVDSLNP**

5 Cre10.g434750/1-555 98.6% 57.1%  **------RFPMGKIDQTYMWKVGQKVRAERDESKI-PVNPFTAGVYVAVMMATVEVLREKGHPFSEICNESIIEAVDSLNP**

6 Mapoly0019s0065/1-595 100.0% 69.6%  **PKEGLPAFPMGIIDNTHMWRVGDKVRSTRDENDLGPLHPFTAGVFVATMMAQIEVLRKKGHSYSEIINESVIEAVDSLNP**

7 Pp3c19_22360/1-589 100.0% 68.4%  **EKEGLPAFPMGKIDGTRMWQVGEKVRASRPKGDMGPLHPFTAGVYCALMMAQIEVLRRKGHSYSEMVNESVIEAVDSLNP**

8 Pp3c21_20900/1-590 100.0% 69.3%  **EKEGLPSFPMGKIDGTRMWQVGEKVRASRPKGDMGPLHPFTAGVYCALMMAQIEVLRKKGHSYSEMVNESVIEAVDSLNP**

9 Sacu_v1.1_s0008.g004218/1-581 100.0% 69.7%  **EKDGLPAFPMGKIDQTKMWKVGERVRSVRPEGDLGPLYGFTAGVYIALMMAQIEVLRKKGHSYSEIVNESVIEAVDSLNP**

10 G28546/1-603 92.1% 57.8%  **EKEGLPAFPMGKIDQTKMWKVGEKVRAARKEGALGPLNPFTAGVYIAVMMAQIEVLKKKGHLYSEIVNESLIEAVDSLNP**

11 Anthoceros/1-326 63.1% 82.8%  **VSDSLQ--------------------------------------------------------------------------**

12 Anthoceros/1-290 55.7% 44.2%  **AKEGL-AFPMGKIDGTRMWKVGEKVRSTRL--------------------------------------------------**

13 Anthoceros/1-193 35.8% 26.2%  **-----------------TWSVC--WRSTGP--------------------------------------------------**

cov pid **561**  **. . . 6 . . . .** **640**

1 157966/1-517 100.0% 100.0%  **FMHARGVSFMVDNCSTTARLGSRKWAPRFDYILTQQAFVSVDAGAPLDKELFDKFLSDPVHQAMQVCSELRPTVDISVTA**

2 AT3G58610/1-591 100.0% 67.2%  **FMHARGVSFMVDNCSTTARLGSRKWAPRFDYILTQQALVAVDSGAAINRDLISNFFSDPVHGAIEVCAQLRPTVDISVPA**

3 Bradi2g15790/1-581 100.0% 68.3%  **FMHARGVAFMVDNCSTTARLGSRKWAPRFDYILTQQAFVTVDKDAPINQDLISNFMSDPVHGAIEVCAELRPTVDISVTA**

4 Bradi2g45330/1-579 100.0% 68.7%  **FMHARGVAFMVDNCSTTARLGSRKWAPRFDYVLTQQAFVTVDKNAPINQDLISNFLSDPVHGAIEICAQLRPTVDISVTA**

5 Cre10.g434750/1-555 98.6% 57.1%  **YMHARGVAFMVDNCSYTARLGSRKWAPRFDYIIEQQAFVDIDSGKAADKEVMAEFLAHPVHSALATCSSMRPSVDISVGG**

6 Mapoly0019s0065/1-595 100.0% 69.6%  **FMNARGVSFMVDNCSTTARLGSRKWAPRFDYILTQQAYVAVDQGAPINQELISSFVSDPVHKSIEICAELRPTVNIAVTA**

7 Pp3c19_22360/1-589 100.0% 68.4%  **FMHARGVAFMVDNCSTTARLGSRKWAPRFDYILTQQAYTAVDNGTPINKDVLESFRADPVHQAIAVCAELRPSVDIAVAE**

8 Pp3c21_20900/1-590 100.0% 69.3%  **FMHARGVAFMVDNCSTTARLGSRKWAPRFDYILTQQAYTAVDNGAAVNKDVLESFKADPVHQAIAVCAQLRPSVDIAVTE**

9 Sacu_v1.1_s0008.g004218/1-581 100.0% 69.7%  **FMHARGVAFMVDNCSTTARLGSRKWAPRFDYILTQQAFVDVDAKVPVNEDLIGNFFSDPVHNALEVCAELRPTLDIAVTA**

10 G28546/1-603 92.1% 57.8%  **YMHAKGVAYMIDNCSTTARLGARKWAPRFDYNLTQQG------------------RKDMVVDQIALFS------------**

11 Anthoceros/1-326 63.1% 82.8%  **--------------------------------------------------------------------------------**

12 Anthoceros/1-290 55.7% 44.2%  **------------------------------YNMNKQNNK-----------------------------------------**

13 Anthoceros/1-193 35.8% 26.2%  **--------------------------------------------------------------------------------**

cov pid **641**  **: . ]** **663**

1 157966/1-517 100.0% 100.0%  **T-------ADYVRQELRH-----**

2 AT3G58610/1-591 100.0% 67.2%  **D-------ADFVRPELRQSSN--**

3 Bradi2g15790/1-581 100.0% 68.3%  **D-------ADFVRPELRQSS---**

4 Bradi2g45330/1-579 100.0% 68.7%  **D-------ADFVRPELRQSS---**

5 Cre10.g434750/1-555 98.6% 57.1%  **ENSSVGVGAGAARTEFRSTAAKV**

6 Mapoly0019s0065/1-595 100.0% 69.6%  **D-------AGFVRPELRQ-----**

7 Pp3c19_22360/1-589 100.0% 68.4%  **D-------ADYVRAELRQ-----**

8 Pp3c21_20900/1-590 100.0% 69.3%  **D-------ADYVRAELRQ-----**

9 Sacu_v1.1_s0008.g004218/1-581 100.0% 69.7%  **D-------ADYVRPELRQ-----**

10 G28546/1-603 92.1% 57.8%  **-----------------------**

11 Anthoceros/1-326 63.1% 82.8%  **-----------------------**

12 Anthoceros/1-290 55.7% 44.2%  **-----------------------**

13 Anthoceros/1-193 35.8% 26.2%  **-----------------------**

>157966/1-517

------------------------------------------------------------------------

------------------MVATRG-AEVEFETKVFTKEKVTLGGKDEFIVRGGRHLFEKL--PEAFKGIKQI

GVLGWGSQGPAQAQNLRDSLEAVKSDIVVKIGLRKGSKSAADARAAGFTEEN----GTLG-DVIETVAGSDL

VLLLISDAAQADNFREIFAALKPKAILGLSHGFLLGHLESIGEKFPKDISVIAVCPKGMGPSVRRLYVQGKE

VNGAGINASFAVHQDVDGRATDVALGWSVALGSPFTFVTTLEDEYKSDIYGERGILLGAVHGLVEALYRRYT

GNG------------------------------------MSEEAAYKNTVESVTGTISKTISSKGMVSLYRS

LSDEGKKEFEAAFCASYYPAMDVLYEIYDEVSSGNEIRSVVLAGRRFSARGGLPAFPMGTIDQTHMWKVGEK

VRAARPEGDLGPLHPFTAGVYVAVMMAQIEILRNKGHSYSEMVNESIIEAVDSLNPFMHARGVSFMVDNCST

TARLGSRKWAPRFDYILTQQAFVSVDAGAPLDKELFDKFLSDPVHQAMQVCSELRPTVDISVTAT-------

ADYVRQELRH-----

>AT3G58610/1-591

MAAATSSIAPSLSCPSPSSSS-------KTLWSSKARTLALPNIGFLSSSSKSLR-------------SLTA

TVAGNGATGSSLAARMVSSSAVKAPVSLDFETSVFKKEKVSLAGYEEYIVRGGRDLFKHL--PDAFKGIKQI

GVIGWGSQGPAQAQNLRDSLVEAKSDIVVKIGLRKGSRSFEEARAAGFTEES----GTLG-DIWETIAGSDL

VLLLISDAAQADNYEKIFSHMKPNSILGLSHGFLLGHLQSSGLDFPKNISVVAVCPKGMGPSVRRLYVQGKE

INGAGINASFAVHQDVDGRAADVALGWSVALGSPFTFATTLEQEYRSDIFGERGILLGAVHGIVESLFRRYT

ENG------------------------------------MSEDLAYKNTVECITGTISRTISTQGMLAVYNS

LSEEGKKDFETAYSASFYPCMEILYECYEDVQSGSEIRSVVLAGRRFYEKEGLPAFPMGNIDQTRMWKVGER

VRKSRPAGDLGPLYPFTAGVYVALMMAQIEILRKKGHSYSEIINESVIESVDSLNPFMHARGVSFMVDNCST

TARLGSRKWAPRFDYILTQQALVAVDSGAAINRDLISNFFSDPVHGAIEVCAQLRPTVDISVPAD-------

ADFVRPELRQSSN--

>Bradi2g15790/1-581

------MAASTISFSHPKTLA---------AAAAVPKTLPIPTASSVAFPASHP--------------AWVL

SAARRRAVAAMVAAPTTTASVGAAMPSLDFDTSVFNKEKVSLAGHEEYIVRGGRNLFPLL--HEAFKGVKQI

GVIGWGSQGPAQAQNLRDSLAEAKSDIVVKIGLRKGSKSFEEARGAGFSEEN----GTLG-DIWETISGSDL

VLLLISDSAQADNYEKIFSHMKPNSILGLSHGFLLGHLQSHGLDFPKNISVVAVCPKGMGPSVRRLYVQGKE

VNGAGINASFAVHQDVDGRATDVALGWSVALGSPFTFATTLEQEYKSDIFGERGILLGAVHGIVEALFRRYT

EQG------------------------------------MDEALTYKSTVEGITGIISKTISKKGMLEVYNS

LSEEGKKEFNKAYSASFYPCMDILYECYEDVASGSEIRSVVLAGRRFYDKEGLPAFPMGKIDQTRMWKVGEK

VRLTRPDGDLGPLHPFTAGVYVALMMAQIEVLRKKGHSYSEIINESVIESVDSLNPFMHARGVAFMVDNCST

TARLGSRKWAPRFDYILTQQAFVTVDKDAPINQDLISNFMSDPVHGAIEVCAELRPTVDISVTAD-------

ADFVRPELRQSS---

>Bradi2g45330/1-579

-MAAATSSASSLAFSHPKTLN---------------PASKTPAVGSVSFPAAQ---------------TPCI

LASSAGRCRAVVAKVASPSVIGATMPSLDFETSVFKKEKVSLAGHDEYIVRGGRNLFPLL--PEAFKGIKQI

GVLGWGSQGPAQAQNLRDSLAEAKSDIVVKIGLRKGSKSFQDARAAGFTEEN----GTLG-DIWETVSSSDL

LLLLISDAAQADNYEKIFSHMKPNSILGLSHGFLLGHLQSVGLDFPQNISVIAVCPKGMGPSVRRLYVQGKE

VNGAGINSSFAVHQDVDGRATDVALGWSIALGSPFTFATTLEQEYRSDIFGERGILLGAVHGIVEALFRRYT

EQG------------------------------------MDEVLAYKNTVECITGIVSKTISKKGMLEVYNS

FTEEGKKQFKEAYSAAFYPSMDILYECYEDVSSGSEIRSVVLAGRRFYEKEGLPAFPMGNIDQTRMWKVGER

VRATRPQGDLGPLHPFTAGVFVALMMAQIEVLRKKGHSYSEIINESVIESVDSLNPFMHARGVAFMVDNCST

TARLGSRKWAPRFDYVLTQQAFVTVDKNAPINQDLISNFLSDPVHGAIEICAQLRPTVDISVTAD-------

ADFVRPELRQSS---

>Cre10.g434750/1-555

--MQLLNSKSRVLSGSRQQAA-----------------------------------------------AKAV

RVAPSGRRS---------AVRVSAAVHLDFNTKVFQKEHAKFGPTEEYIVRGGRDKYPLL--KEAFKGIKKV

SVIGWGSQAPAQAQNLRDSIAEAGMDIKVAIGLRPDSPSWAEAEACGFSKTD----GTLG-EVFEQISSSDF

VILLISDAAQAKLYPRILAAMKPGATLGLSHGFLLGVMRNDGVDFRKDINVVLVAPKGMGPSVRRLYEQGKS

VNGAGINCSFAIQQDATGQAADIAIGWAIGVGAPFAFPTTLESEYKSDIYGERCVLLGAVHGIVEALFRRYT

RQG------------------------------------MSDEEAFKQSVESITGPISRTISTKGMLSVYNS

FNEADKKIFEQAYSASYKPALDICFEIYEDVASGNEIKSVVQAVQRFD------RFPMGKIDQTYMWKVGQK

VRAERDESKI-PVNPFTAGVYVAVMMATVEVLREKGHPFSEICNESIIEAVDSLNPYMHARGVAFMVDNCSY

TARLGSRKWAPRFDYIIEQQAFVDIDSGKAADKEVMAEFLAHPVHSALATCSSMRPSVDISVGGENSSVGVG

AGAARTEFRSTAAKV

>Mapoly0019s0065/1-595

MAAASCTAALSSSCVVLHSKDAGAVSRNGAMQQQIRSFDGLHRHQLLIAKTAEKCL------------RAAV

PAARNGRKGGAMCTQAVAMPATRG-TEVVFESKVFKKEKITLAGGDEYIVRGGRDLFPLL--PKALQGIKKI

GVIGWGSQGPAQAQNFRDSLNAIGSDIVVKVGLRKGSKSCQEARAAGFTEET----GTLG-DVLETVSESDL

VMLLISDAAQADNYKEIFAAMKDNAILGLSHGFLLGHLQSLREEFPKNISVIAVCPKGMGPSVRRLYVQGKE

VNGAGINASFAVHQDVDGRATDVALGWSVALGSPFTFVTTLEDEYKSDIFGERGILLGAVHGIVESLFRRYT

SNG------------------------------------MQEEAAYKNTVESITGIISRTISAKGMLAVYRS

LSDEGKKEFEAAYSASYLPAMDILYECYEDVASGNEIRSVVLAGRRFSPKEGLPAFPMGIIDNTHMWRVGDK

VRSTRDENDLGPLHPFTAGVFVATMMAQIEVLRKKGHSYSEIINESVIEAVDSLNPFMNARGVSFMVDNCST

TARLGSRKWAPRFDYILTQQAYVAVDQGAPINQELISSFVSDPVHKSIEICAELRPTVNIAVTAD-------

AGFVRPELRQ-----

>Pp3c19_22360/1-589

MAAVTLSHCAAPSSSVAHRSS----EVLGSAGPKMTSFAGLRSVAFAPKLEKSL--------------RNAV

AAVPCWRRGGAMSINMVATPAVRG-VDVEFQTEIFKKEKITPAGRDEYIVRGGRDLFHLL--PKALTGIKKI

GVIGWGSQGPAQAMNIRDSLAEIKSDIVVKIGLRKGSKSCEDARSVGFTEES----GTLG-DVIETVAESDL

VLLLISDAAQADNYKEIFKAMKPNSVLGLSHGFLLGHLNSVGDSFPDDISVIAVCPKGMGPSVRRLYVQGKE

VNGAGINASFAVHQDVDGRATDVALGWSIALGSPFTFATTLEDEYKSDIYGERGILLGAVHGIVESLFRRYT

AQG------------------------------------MSEEDAYKNTVEGITGVISKIISTKGILAVYEA

LSEEGKKEFEAAYSASFYPSMDILYECYEDVASGNEIRSVVLAGRRFSEKEGLPAFPMGKIDGTRMWQVGEK

VRASRPKGDMGPLHPFTAGVYCALMMAQIEVLRRKGHSYSEMVNESVIEAVDSLNPFMHARGVAFMVDNCST

TARLGSRKWAPRFDYILTQQAYTAVDNGTPINKDVLESFRADPVHQAIAVCAELRPSVDIAVAED-------

ADYVRAELRQ-----

>Pp3c21_20900/1-590

MAAVTLSHCATASAGVAHRSS---ETVVGSANAKMASFSGLRIVAFAPKLEKSL--------------SAAV

AAVPSRMRGGAIAASMVASPALRG-ADVEFQSEVFKKEKITPAGRDEYIVRGGRDLFHLL--PEAFKGIKKI

GVIGWGSQGPAQAMNIRDSLAEIKSDIVVKIGLRKGSKSCADARAVGFTEES----GTLG-DVLETVAESDL

VLLLISDAAQADNYQDIFKAMKPKSVLGLSHGFLLGHLNSVNDSFPEDISVIAVCPKGMGPSVRRLYVQGKE

VNGAGINASFAVHQDVDGRATDVALGWSVALGSPFTFATTLEDEYKSDIYGERGILLGAVHGIVESLFRRYT

ACG------------------------------------MSEEDAYKNTVESITGVISKTISTQGILAVYES

LSEEGKKEFEAAYSASFYPSMDILYECYEDVASGNEIRSVVLAGRRFSEKEGLPSFPMGKIDGTRMWQVGEK

VRASRPKGDMGPLHPFTAGVYCALMMAQIEVLRKKGHSYSEMVNESVIEAVDSLNPFMHARGVAFMVDNCST

TARLGSRKWAPRFDYILTQQAYTAVDNGAAVNKDVLESFKADPVHQAIAVCAQLRPSVDIAVTED-------

ADYVRAELRQ-----

>Sacu_v1.1_s0008.g004218/1-581

-MACSAAAAAASSTSLASTFN------------AVSSRLPAPRASTAKLSFSRSV-------------SESS

SIAPASGNVGRVRMTVATRTADRG-IDVQFDSAIFKKEKITLAGRDEYIVRGGRNLFYLL--PEAFKGIKQI

GVIGWGSQAPAQAQNLRDSLLEAKSNIKVKIGLRKGSKSVSEARAAGFTEES----GTLG-DVLDTVSESDL

VLLLISDAAQADNYKQIFAAMKPKSILGLSHGFLLGHLQSVREDFRKDISVIAVCPKGMGPSVRRLYVQGKE

INGAGINSSFAVHQDVDGRASDVALGWSVALGSPFTFATTLEDEYKSDIYGERGILLGAVHGLVEALFRRYT

DAG------------------------------------MPEEDAYKNTVESITGIISKTISTKGMKAVYNS

LDEQGKRDFKVAYSASYYPCMDILYECYEDVASGNEIRSVVLAGRRFQEKDGLPAFPMGKIDQTKMWKVGER

VRSVRPEGDLGPLYGFTAGVYIALMMAQIEVLRKKGHSYSEIVNESVIEAVDSLNPFMHARGVAFMVDNCST

TARLGSRKWAPRFDYILTQQAFVDVDAKVPVNEDLIGNFFSDPVHNALEVCAELRPTLDIAVTAD-------

ADYVRPELRQ-----

>G28546/1-603

MAAAVSQGISSQVFSIAARQDQSAVLASTSSSSDCAATLVIPRFTGLQKSSTSAIAAGRHVSHREGFSKQAQ

RLSRRTRAGGAVTVEMVVAVPSANKVESDFETKVFKKEKITLAGNDEYIVRGGRDLFHLL--SKAFQGIKKI

GVIGWGSQGPAQSQNLRDSLEAANSDIVVKVGLRTNSKSRVEARNAGFTEEN----GTLG-DVLEVVKESDL

VLLLISDAAQADEYESILGAMKPGSCLGLSHGFLLGHMKSVGDDFRKDISVVAVCPKGMGPSVRRLYEQGKE

VNGAGINASFAVHQDVDGRATDIALGWSVALGSPFTFRTTLESEYKSDIYGERGILLGAVHGVVEALFRRYT

EQGMSDEDAFMMIFWLLSILLGAVHGVVEALFRRYTEQGMSDEDAFTNSVESITGVISQTISRKGMLAVYES

LSKEGKREFEAAYSASYLPAMDILYECYEDVESGNEIRSVVLAGRRFREKEGLPAFPMGKIDQTKMWKVGEK

VRAARKEGALGPLNPFTAGVYIAVMMAQIEVLKKKGHLYSEIVNESLIEAVDSLNPYMHAKGVAYMIDNCST

TARLGARKWAPRFDYNLTQQG------------------RKDMVVDQIALFS--------------------

---------------

>Anthoceros/1-326

------------------------------------------------------------------------

---------------------------------------------MQYIVRGGRDLFELL--PKAFKGIKQI

GVIGWGSQGPAQAQNLRDSLADAKSDIVVKIGLRKGSKSVAEAREAGFTEAD----GTLG-DVVETVSGSDL

VLLLISDAAQADNYKEVFAALKPKSILGLSHGFLLGHLQSTGDTFPKDISVIAVCPKGMGPSVRRLYVQGKQ

INGAGINSSFAVHQDADGRATDVALGWSVALGSPFTFVTTLEDEYRSDIYGERGILLGAVHGIVEALFRRYT

ENG------------------------------------MAEDAAYKNTVESVTGVISRIISTKGMLAVYRS

LSDEGKKEFEAAYSASYLPSMDILYECYEDVASGNEIRSVVLAGRRFSVSDSLQ------------------

------------------------------------------------------------------------

------------------------------------------------------------------------

---------------

>Anthoceros/1-290

------------------------------------------------------------------------

--------------------------------------MTVPATSHQLHITASRPLFISMHNPTSKFKLQHK

GIV---------------------------IGLRKGSKSVAEASEAGFTEAD----GTLG-DVVETVSGSDL

VLLLISDAAQAYNYKEIFSALKAKAILGLSHGFLLGHLQSIGDTFPKDMSVIAVCPKGMGLSVRRLYVQGKQ

INGAGINSSFAVHQDDDGKAADVAL--------------------RSDIYGERGIRLGAVHGIVAALFRRYT

ENG------------------------------------MAEDAAYKNTVESAAGVISRIISTKGMLVVYEF

LT------------------------------SGNEIRSVVLAGRRFPAKEGL-AFPMGKIDGTRMWKVGEK

VRSTRL------------------------------------------------------------------

--------------YNMNKQNNK-------------------------------------------------

---------------

>Anthoceros/1-193

------------------------------------------------------------------------

------------------------------------------------------------------------

--------------------------------MTRGSKLVAEAREAGYTEADRDAWGCRGYCVWQRFGPAAD

YGMPHGQITTGN-----LLCSEAEVCFGTVSWFPPGTSAVNLDPFPKDISVMAFCPKGTGSSVRRP---GQA

NRWSWDQLELAVHQDADGRATDIALGWS---REAFSWVQLL------------------------ALFRSYT

ENG---------------------------------ENRTAEDAAYKNTVESVSRIISTKLRSFGL------

-----------------------------------------------------------------TWSVC--

WRSTGP------------------------------------------------------------------

---------------------------------------------------------------------------------------

**ADP glucose pyrophosphorylase large subunit**

cov pid  **1** **[ . . . . : . . .** **80**

1 Selmo_138695/1-447 100.0% 100.0%  **----------------------------------------------------------------APAIGK----------**

2 Selmo_149205/1-533 99.3% 63.2%  **--------------------------------------MANAAISSSPMGSVSAGGRKDYLHACKSASSS----------**

3 Selmo_169778/1-463 100.0% 95.2%  **-------------------------------------------------------MQGFHRRGCLGRYCQ----------**

4 AT1G27680/1-518 100.0% 64.7%  **------------------------------------------MESCFPAMKLNQCTFGLNNEIVSERVSA----------**

5 Bradi1g09537/1-504 100.0% 63.5%  **----------------------------------------MDLRVAAPASAAAAARRGVPGGGVVRLRAL----------**

6 Bradi1g53500/1-512 100.0% 63.5%  **-----------------------------------------MATSTGPMAPHAACPFSPVVLRRAPRLVR----------**

7 Bradi2g14970/1-522 99.6% 54.2%  **----------------------------------MSRMQFSSVLPLEGKACVSPLRRNVESSGSERIRIG----------**

8 Cre07.g331300/1-719 98.7% 23.6%  **-----------------------------MLLRHSAPGRGNAADRHRCTRLPTPPHAGTVAAGTGASAAR----------**

9 Cre13.g567950/1-512 99.6% 53.9%  **------------------------------------------MATLQAKSRAAASSARSEKAVPASFSSGS---------**

10 Cre16.g683450/1-560 99.3% 33.2%  **------------------------------------------------------------MKQLQQFQER----------**

11 Mapoly0023s0119/1-534 100.0% 66.7%  **------------------------------------MAAAAVTSVGWHCAVKNVAVASTSGVAERSAEAS----------**

12 Mapoly0033s0108/1-520 98.7% 47.7%  **-------------------------------------MAGVATAAVSCMSVHSVLAETKLGRGTKSVTAS----------**

13 Mapoly0101s0020/1-543 100.0% 67.6%  **----------------------------------------MAVAASNVRSAVGLSQCSVSGSTAAAPVSS----------**

14 Mapoly0132s0055/1-569 98.9% 47.0%  **-------------------------MAVATIVPFNLKLHSAQLNGTGPFRSSSNSLRNLFSSSSDRFFGRTDQIWKLIKT**

15 Pp3c11_5720/1-539 99.8% 63.3%  **----------------------------------MAATMACVSTSCCSITSSRDSYGRQGQQQQQGGQGA----------**

16 Pp3c11_8270/1-524 98.7% 47.0%  **------------------------------------------MAATLASTAAAVTSARLSNGSTTPRGGG----------**

17 Pp3c1_3040/1-532 99.8% 64.5%  **----------------------------------MAAATACVSTGCCSITSAREGYGRQQQGSPSNAAAK----------**

18 Pp3c1_31540/1-543 100.0% 59.3%  **------------------------------------MAAAGMFASTSYCTLAGPSCYEPILDGDSSLRSG----------**

19 Pp3c14_12850/1-541 100.0% 59.9%  **------------------------------------MAAAGMFASTSYCTVARPAHCEALSGDVPFRSGF----------**

20 Pp3c17_13940/1-555 100.0% 58.7%  **------------------------------------MAATGTFASTRYTALARPARCEAVGYDATMRSGF----------**

21 Pp3c19_8410/1-532 100.0% 65.6%  **--------------------------------MAGAAILRSAQSAIVGTVACSSSQQKHSVNQLAAPPCS----------**

22 Pp3c2_35010/1-524 98.7% 46.6%  **---------------------------------------MAARTAAAAAAALAVPSTSVRLSSGRAPRDS----------**

23 Pp3c7_20320/1-526 98.7% 46.4%  **--------------------------------------MAAALAAVAAAAASTAAASPARLSVERTTRGA----------**

24 Pp3c7_20690/1-533 98.7% 45.8%  **---------------------------------MMAVAYTAAVMPTAIINSETASLNVTSKLSVRSRICS----------**

25 Pp3c7_22280/1-536 99.8% 63.5%  **----------------------------------MAAGTACVYTSCSSIKCVQEGCGRTWQQLNVATIPS----------**

26 Pp3s127_20/1-546 100.0% 58.2%  **------------------------------------MAATGIIASTSYCTLTRPSRQEAFWGVDSCLRSG----------**

27 Sacu_v1.1_s0067.g016264/1-532 100.0% 65.4%  **------------------------------------------MASTAADSLQFLSRAPFFSTSSSSSTAS----------**

28 Sacu_v1.1_s0095.g019244/1-550 100.0% 64.0%  **-------------------------------MASSSSAPPTSSSSSSISRTLLLRSPSSIASSRSPFLSP----------**

29 Sacu_v1.1_s0157.g023711/1-526 100.0% 65.4%  **------------------------------------------MAYAASVPSPLPFSSPSTTTPPTFSSSF----------**

30 Sacu_v1.1_s0001.g000380/1-536 98.7% 45.8%  **-------------------------------------MANAAAIASSSRSTTHPPHLQFSSLLCTTTPTH----------**

31 Sacu_v1.1_s0076.g017538/1-573 98.7% 43.5%  **---------------------------------------MAAASASAPCFRHHHRSFPSLPRHCPSSANL----------**

32 Azfi_s0120.g046768/1-488 100.0% 71.5%  **--------------------------------------------MTHMINNIGTNVVPIPRGVPESESQG----------**

33 Azfi_s0003.g008048/1-504 98.7% 48.4%  **----------------------------------------MAGTTISPVSCFCNPRRSKLTSSFIPPRPS----------**

34 Azfi_s0106.g045016/1-528 98.7% 46.6%  **-----------------------------------MAEASMAIFSASSSSCIANLNVRWQPSSPSSLSVT----------**

35 Azfi_s0012.g012923/1-564 98.7% 43.5%  **--------------------------MSMAAETPSALAMVAPYSSCSSSSDSCICRTLSMATTTTMMTRSTRRYQRYNAS**

36 CHBRA475g00200/1-614 99.8% 51.2%  **MAAAMAHALPMPVVADGSTSFSLRPSGPVPTHAVTASAAMVKSAARAPSSAAGWSSALVPQSVSRASRDLISAREAFVAG**

37 AaBONN_Sc2ySwM_340.122.1/1-513 98.7% 48.0%  **--------------------------------------MAAGGCFCNVAGKSCDTLRPVLRSSSRSLSST----------**

38 Anthoceros/1-550 100.0% 66.0%  **-------------------------------MAAAAAGGLATMSSTAPAACCSPSGSASSASSSSSSTSYSS--------**

cov pid  **81**  **. 1 . . . . : .** **160**

1 Selmo_138695/1-447 100.0% 100.0%  **--------------------------------------------------------------------------------**

2 Selmo_149205/1-533 99.3% 63.2%  **---------LAKRDLFGAAL---------HLKKIP------------EKSKPRIAKCQAAQKGGNAALSSAVLVDYPPRE**

3 Selmo_169778/1-463 100.0% 95.2%  **-------------GNRGIGK------------------------------------------------------------**

4 AT1G27680/1-518 100.0% 64.7%  **---------FWGTQVVKPNH---------LRTTKL---------------------RSAPQKKIQTNLIRSVLTPFVDQE**

5 Bradi1g09537/1-504 100.0% 63.5%  **--------------------------------------------------------AAGGRQRRLSVRVSVATTEAATAA**

6 Bradi1g53500/1-512 100.0% 63.5%  **---------LAAPRRAAAAT---------TSCVLT-----------------------DAPQGIKMEPSGAPSPESSTVV**

7 Bradi2g14970/1-522 99.6% 54.2%  **-----------------DSS---------TIKHER------------------ALRRMCFGARGAASGAQCVLTSDASPD**

8 Cre07.g331300/1-719 98.7% 23.6%  **---------RSRGALRVLAF---------REQETRQQDKNDDQSKTHVKEKAGSAAAAAAAAAGRRPQTRSHFPPVGRGG**

9 Cre13.g567950/1-512 99.6% 53.9%  **---------FRGQAVARPAA---------TRAVR----------------------SVAARSRRAQAVKAIIEAPL----**

10 Cre16.g683450/1-560 99.3% 33.2%  **---------WSGDMGGASGS---------SGLGSG---------------------GAATPGTGLGEHSGSFGDPELSRS**

11 Mapoly0023s0119/1-534 100.0% 66.7%  **---------FGRSAFRGDAV---------SGFLKQ--------------GNRVLDSSRSGSSRQQSVFKRAPISSVITEV**

12 Mapoly0033s0108/1-520 98.7% 47.7%  **---------SSDLRSASSSL---------RGARLS--------------------SQCTQRSHARMASKTLVAPRAVSDS**

13 Mapoly0101s0020/1-543 100.0% 67.6%  **---------RAGSGLGRKAF---------CGESVT---------GSFGKGRVDFQVGESKSRRRSGSLVQPVASSLLAEI**

14 Mapoly0132s0055/1-569 98.9% 47.0%  **---------CSATAYVSSTS---------KSCNCR------WTTSKSSNGSPVVAVAGKHNRRVNKAATTSLLYDIAKE-**

15 Pp3c11_5720/1-539 99.8% 63.3%  **---------APASPPFRSGF---------SGEKLA------ASLKAQAWLPVAPLKQRKDSKGPLSVSSVLMEPRPLEDT**

16 Pp3c11_8270/1-524 98.7% 47.0%  **---------FAKRGSALVGS---------NSSSLR------------GARLAVSSSYSSASARAQRPRKGVVSPRNVSDS**

17 Pp3c1_3040/1-532 99.8% 64.5%  **-------------SLSRSSF---------CGDKVA-------------ASLKAQAWLPATPKRQNAAMGPIQVSSVLAEL**

18 Pp3c1_31540/1-543 100.0% 59.3%  **---FKGDKKLSSAGFLAPGG---------DGLSFK---------------SNGCSKAAAIRSKSSTLNVSAVLAERPMET**

19 Pp3c14_12850/1-541 100.0% 59.9%  **---------RGEQKMSSAGFM--------CGGDCA------------ALKSNVSPKPTAARTKSSGLDISAILAERPLEA**

20 Pp3c17_13940/1-555 100.0% 58.7%  **---------KGDLKPASSGFLA-------GGGRLALVTSVGPKRTVARAKTIGLEVSAVLAERPMGALTRQVTREMEKEM**

21 Pp3c19_8410/1-532 100.0% 65.6%  **---------FSVSSFLHAGI---------CSPLRN--------------------DARSSTWCRRVSKPNVVESLFVETT**

22 Pp3c2_35010/1-524 98.7% 46.6%  **---------FRSGGCAQVGS---------RSSSLR---------------GAGVVCPSNVTKITQRTRKVVVSPRNVSDS**

23 Pp3c7_20320/1-526 98.7% 46.4%  **---------FVSGGNAQLGS---------SSSSLR--------------GTGVALSSSNVTLRRQRHRKVVVSPRNVSDS**

24 Pp3c7_20690/1-533 98.7% 45.8%  **---------FAKCSYSSLGS---------CESFLG--------------GTRMADSSWNLFSSGKQSSRKMLLSPRAISD**

25 Pp3c7_22280/1-536 99.8% 63.5%  **--------------LPRSAF---------CGKKLA------SSLKAQAWLPVAPLKRRNDSKHALKVSSVLMEPRPLENT**

26 Pp3s127_20/1-546 100.0% 58.2%  **---------FKGRG--KLSS---------AGFLAP------GVNSLRLRGNTCPRPATAARSKSNTLSVSAVLAERPIET**

27 Sacu_v1.1_s0067.g016264/1-532 100.0% 65.4%  **--------SFLGTSLLPPSR---------HGLPSH---------PVHHHFNSGCQKPPLHRRSCLVSASSATVLQNVSQH**

28 Sacu_v1.1_s0095.g019244/1-550 100.0% 64.0%  **---------HHSLPFPSPPS---------LRLPSP------LLPISSHECSDRLSHPPPPRASTSGNSAILQLERRLHML**

29 Sacu_v1.1_s0157.g023711/1-526 100.0% 65.4%  **---------YGSCLFPFHAY---------SVASFP----------------RTIRHRQHRDRSSLCIRSSASTVSLLSDT**

30 Sacu_v1.1_s0001.g000380/1-536 98.7% 45.8%  **---------YRPRCSRLLSS---------SSSSFL----LSSTSPFLLHLHRGSASSSASNSCTTTLAPFIVAPRAVSDS**

31 Sacu_v1.1_s0076.g017538/1-573 98.7% 43.5%  **---------ASLHAFSSPRL---------FASPLL--------------------------HRRRAPSFPSFSPRAVSDS**

32 Azfi_s0120.g046768/1-488 100.0% 71.5%  **---------------------------------------------------------------------VELEVRAAPES**

33 Azfi_s0003.g008048/1-504 98.7% 48.4%  **-----------------SSS---------HSPSSS-------------------------SLYFSSRRVFLVSPRAVSDS**

34 Azfi_s0106.g045016/1-528 98.7% 46.6%  **---------FIRSNNLASSN---------SISDIS-------------SLTARPSVGSSLHRQGRPNHFQLVSPRAVSDS**

35 Azfi_s0012.g012923/1-564 98.7% 43.5%  **SLSSSSLLSPITHATIRPCF---------HGSAPP------KSLASCKAFSDTSFLSMRSTNGYSRRHINTISPRAVSDS**

36 CHBRA475g00200/1-614 99.8% 51.2%  **GGSAEGAGFFRGQMFGAAGIPGAASFKSPSGEKLPLEMATVLCAAEPPARPGSDRGPPAARKGRGKMEVNAVLADMAKRT**

37 AaBONN_Sc2ySwM_340.122.1/1-513 98.7% 48.0%  **---------AAVGSFHALVA---------NIDSTP----------------------------WRSSKLRNLHIRAVSDS**

38 Anthoceros/1-550 100.0% 66.0%  **---------LPATRLGRAAF---------CGTPVS------GGFFSAALGGSRLCAGSGQPRRAERASKTVRATAVLADL**

cov pid **161**  **. . . 2 . . . .** **240**

1 Selmo_138695/1-447 100.0% 100.0%  **----------------------PRADPR-TVVSLILGGGAGT--RLFPLTNRRAKPAVPIGGAYRLIDVPMSNCINSGIN**

2 Selmo_149205/1-533 99.3% 63.2%  **AKQVELPFSV---------FETPRVDPK-SVVSIILGGGVGT--RLFPLTKQRAKPAVPIGGGYRLIDVPMSNCINSGIN**

3 Selmo_169778/1-463 100.0% 95.2%  **----------------------PRADPR-TVVSLILGGGAGT--RLFPLTNRRAKPAVPIGGAYRLIDVPMSNCINSGIN**

4 AT1G27680/1-518 100.0% 64.7%  **---SHEPLLR-----------TQNADPK-NVASIILGGGAGT--RLFPLTSKRAKPAVPIGGCYRLIDIPMSNCINSGIR**

5 Bradi1g09537/1-504 100.0% 63.5%  **VGASEDADT-------------ETRNAR-TVVAVILGGGAGT--RLFPLTKRRAKPAVPIGGAYRLIDVPMSNCINSGIN**

6 Bradi1g53500/1-512 100.0% 63.5%  **APR-------------------RDVGPD-TVASIILGGGAGT--RLFPLTRTRAKPAVPVGGCYRLIDIPMSNCINSKIN**

7 Bradi2g14970/1-522 99.6% 54.2%  **TLVVRTSFRR------------NYADPN-EVAAVILGGGTGT--QLFPLTSTRATPAVPIGGCYRLIDIPMSNCFNSGIN**

8 Cre07.g331300/1-719 98.7% 23.6%  **WEAADVSGRR------EDEGHGPPRISS-STLAVILGGGESD-RRLFPLTEKRALPAVPVGGRYRLIDVPISNCLHACIN**

9 Cre13.g567950/1-512 99.6% 53.9%  **--LRYEP--------------ATKARTS-SVLSIILGGGAGT--RLFPLTKSRAKPAVPIGGAYRLIDVPMSNCINSGIS**

10 Cre16.g683450/1-560 99.3% 33.2%  **---------------------SLSIWPRGNLTAVVLGGGESDSRRLFPLTQYRTLPAVPFGGAYRIIDLLMSNMLNSGIN**

11 Mapoly0023s0119/1-534 100.0% 66.7%  **AKKTVQPVDE-----------SPRADPK-TVVSLILGGGAGT--RLFPLTKRRAKPAVPIGGGYRLIDVPMSNCINSGIN**

12 Mapoly0033s0108/1-520 98.7% 47.7%  **--QNGESCL-------------TPEAGR-SVLGIILGGGAGT--RLYPLTKKRAKPAVPLGANYRLIDIPVSNCINSNVQ**

13 Mapoly0101s0020/1-543 100.0% 67.6%  **AKEIAQAQAP---AQAPLTEFRPRVDPQ-TVASLILGGGAGT--RLFPLTRRRAKPAVPIGGAYRLIDVPMSNCINSGIN**

14 Mapoly0132s0055/1-569 98.9% 47.0%  **--IAAQASLSTDSEV------RSKVDPK-SVACMILGGEAGS--RLFPLTKRRAKSAVPMGGAYRLIDIQMSNCINSGIN**

15 Pp3c11_5720/1-539 99.8% 63.3%  **IVNTGEA---------------TRVDPR-TVLSIILGGGAGT--RLYPLTKRRAKPAVPIGGAYRLIDVPMSNCINSGIN**

16 Pp3c11_8270/1-524 98.7% 47.0%  **-PVVGEACL-------------DPDASR-SVLGIILGGGAGS--RLYPLTKKRAKPAVPLGANYRLIDIPVSNCINSNIN**

17 Pp3c1_3040/1-532 99.8% 64.5%  **DEALEDGTEE-----------KARVDPR-TVLSIILGGGAGT--RLYPLTKRRAKPAVPIGGAYRLIDVPMSNCINSRIN**

18 Pp3c1_31540/1-543 100.0% 59.3%  **LQGQATRERD-------TKVPKTQVNMT-KVFSIILGGGAGT--RLNPLTLRRAKPAVPLGGAYRLIDVPMSNCINSGIN**

19 Pp3c14_12850/1-541 100.0% 59.9%  **LIEKTTRERV-------KKTPKQPVNMT-KVFSIILGGGAGT--RLNPLTLRRAKPAVPLGGGYRLIDVPMSNCINSGIN**

20 Pp3c17_13940/1-555 100.0% 58.7%  **EREREKEKSR------ESMSSKEQVNMT-KVFSIILGGGAGT--RLQPLTLRRAKPAVPLGGGYRLIDVPMSNCINSGIN**

21 Pp3c19_8410/1-532 100.0% 65.6%  **EESVESPPAQ-----------KPSVQAR-SVVSLILGGGAGT--RLFPLTHRRAKPAVPIGGGYRLIDVPMSNCINSGIN**

22 Pp3c2_35010/1-524 98.7% 46.6%  **-PVVGETCL-------------DPDASR-SVLGIILGGGAGS--RLYPLTKKRAKPAVPLGANYRLIDIPVSNCINSNIN**

23 Pp3c7_20320/1-526 98.7% 46.4%  **-PVSVEACL-------------DPDASR-SVLGIILGGGAGS--RLYPLTKKRAKPAVPLGANYRLIDIPVSNCINSNIN**

24 Pp3c7_20690/1-533 98.7% 45.8%  **YPVGGDASLY------------SVDASR-SVLGIILGGGAGT--RLYPLTKKRAKPAVPIGANYRLIDIPVSNCINSNIN**

25 Pp3c7_22280/1-536 99.8% 63.5%  **IVNVGTGEE-------------KRVDPR-TVLSIILGGGAGT--RLYPLTKRRAKPAVPIGGGYRLIDVPMSNCINSGIN**

26 Pp3s127_20/1-546 100.0% 58.2%  **LFQQEQITREREVKTP-----KSQVNMK-KVFSIILGGGAGT--RLNPLTLRRAKPAVPLGGAYRLIDVPMSNCINSGIN**

27 Sacu_v1.1_s0067.g016264/1-532 100.0% 65.4%  **VVQTSETQK-------------SRVDPR-TVVSIILGGGAGT--RLYPLTKQRAKPAVPIGGAYRLIDVPMSNCINSGIN**

28 Sacu_v1.1_s0095.g019244/1-550 100.0% 64.0%  **DPNQWQANNG--------SIIGPRADPK-SVVSVILGGGAGT--RLFPLTSRRAKPAVPIGGAYRLIDVPMSNCINSGIN**

29 Sacu_v1.1_s0157.g023711/1-526 100.0% 65.4%  **YQNAQTSETD-----------KSRVDPR-NVASIILGGGAGT--RLFPLTRRRAKPAVPIGGAYRLIDVPMSNCINSRIN**

30 Sacu_v1.1_s0001.g000380/1-536 98.7% 45.8%  **--PTSEACL-------------DPPARE-SVLSIILGGGAGT--RLYPLTKKRAKPAVPLGANYRLIDIPVSNCINSDIR**

31 Sacu_v1.1_s0076.g017538/1-573 98.7% 43.5%  **--RNDEVCL-------------DPDAGD-SVLGIILGGGAGT--RLYPLTKKRAKPAVPLGANYRLIDIPVSNCINSNIR**

32 Azfi_s0120.g046768/1-488 100.0% 71.5%  **EGLTSAAVQK------------TPVDPR-SVLSIILGGGAGT--RLFPLTKQRAKPAVPIGGAYRLIDVPMSNCINSGIS**

33 Azfi_s0003.g008048/1-504 98.7% 48.4%  **--RNDDACL-------------DPAAGE-SVLSIILGGGAGT--RLYPLTKKRAKPAVPLGANYRLIDIPVSNCINSDIR**

34 Azfi_s0106.g045016/1-528 98.7% 46.6%  **--RNDEVCL-------------DPDAGD-SVLGIILGGGAGT--RLYPLTKKRAKPAVPLGANYRLIDIPVSNCINSNIR**

35 Azfi_s0012.g012923/1-564 98.7% 43.5%  **--RNDEVCL-------------DPDAGD-SVLGIILGGGAGT--RLYPLTKKRAKPAVPLGANYRLIDIPVSNCINSNIR**

36 CHBRA475g00200/1-614 99.8% 51.2%  **TPEEELTHVI---------QLRERALPR-NVISVILGGGAGT--RLYPLTRHRAKPAVPIGGAYRLIDVPMSNCINCGIK**

37 AaBONN_Sc2ySwM_340.122.1/1-513 98.7% 48.0%  **QSASGDSCL-------------DPDAGR-SVLGIILGGGAGT--RLYPLTKKRAKPAVPLGANYRLIDIPVSNCINSNIQ**

38 Anthoceros/1-550 100.0% 66.0%  **VAKETVATSV----------DQSRVDPR-TVVSVILGGGAGT--RLFPLTRRRAKPAVPIGGNYRLIDVPMSNCINSGIN**

cov pid **241**  **: . . . . 3 . .** **320**

1 Selmo_138695/1-447 100.0% 100.0%  **KIFILTQFNSASLNRHLARTYNFGNGVNF-GDGFVEVLAATQTPGEAGMNWFQGTADAVRQFTWVFE-DTRSKEIENVLV**

2 Selmo_149205/1-533 99.3% 63.2%  **RVFVLTQFNSASLNRHLARTYNFINA----GEGFVEVLAATQTPGESGMNWFQGTADAVRQFTWLFE-DVRNKDVDYVLV**

3 Selmo_169778/1-463 100.0% 95.2%  **KIFILTQFNSASLNRHLARTYNFGNGVNF-GDGFVEVLAATQTPGEAGMNWFQGTADAVRQFTWVFE-DTRSKEIENVLV**

4 AT1G27680/1-518 100.0% 64.7%  **KIFILTQFNSFSLNRHLSRTYNFGNGVNF-GDGFVEVLAATQTSGDAGKKWFQGTADAVRQFIWVFE-DAKTKNVEHVLI**

5 Bradi1g09537/1-504 100.0% 63.5%  **KVYVLTQFNSASLNRHLSRAYNFSNGVGF-GDGFVEVLAATQRPGLEGKRWFQGTADAVRQFDWLFD-DAKAKDIEDVII**

6 Bradi1g53500/1-512 100.0% 63.5%  **KIYVLTQFNSQSLNRHIARTYNFGEGVGF-GNGFVEVLAATQTAGESGKRWFQGTADAVRQFLWLFE-DARLKRIENILI**

7 Bradi2g14970/1-522 99.6% 54.2%  **KIFVMTQFNSASLNRHIHRTY-LGGGINF-TDGSVEVLAATQMPGEA-AGWFRGTADAVRKFIWVLEDYYKHKSIEHILI**

8 Cre07.g331300/1-719 98.7% 23.6%  **KMFVLTQYNSQSLNKYINRAYGNREGVPNGGDGFVEVLATTQYPA--GARWSEGNADAVRMMAWLLD-QPRLRRIEDVLI**

9 Cre13.g567950/1-512 99.6% 53.9%  **KIYILTQFNSTSLNRHLGRAYNMGSGVRFGGDGFVEVLAATQTPTD--KEWFQGTADAVRQYSWLLE-DTKNRAIEDVLI**

10 Cre16.g683450/1-560 99.3% 33.2%  **KIHILTAFNSYSLNRHLQRTYDMSGGVPYGGDGYIEVVANSMSPDS--QNWVTGTAGCVRQFMSYFDSNSKNRFIEDIMI**

11 Mapoly0023s0119/1-534 100.0% 66.7%  **KVFILTQFNSASLNRHLARTYNFGNGINF-GDGFVEVLAATQTPGEGGKEWFQGTADAVRQYLWLFE-DAKNKNLEDVLI**

12 Mapoly0033s0108/1-520 98.7% 47.7%  **KIYVLTQFNSASLNRHLSRAYGSNMG-GYKNEGFVEVLAAQQSPEN--PNWFQGTADAVRQYLWLFE----EQNVLEFLV**

13 Mapoly0101s0020/1-543 100.0% 67.6%  **KVFILTQFNSASLNRHLARTYNFGNGVNF-GNGFVEVLAATQTPGESGMNWFMGTADAVRQFTWLFE-DVKNKGVEHVLI**

14 Mapoly0132s0055/1-569 98.9% 47.0%  **KVYVLTQFNSASLNRHISRTYNSVTC----RDGFVEVLAATQTLGD--KRWFMGTADAVRRFSWIFD-NVRSEAVEHVLV**

15 Pp3c11_5720/1-539 99.8% 63.3%  **KVFILTQFNSTSLNRHLARTYNFGK-INF-GDGFVEVLAATQTPGDKGAEWFQGTADAVRQYLWLLE-DAKNKVVEDVVI**

16 Pp3c11_8270/1-524 98.7% 47.0%  **KIYVLTQFNSASLNRHLSRAYASNMG-GYKNEGFVEVLAAQQSPEN--PNWFQGTADAVRQYLWLFE----EAQVLEYVI**

17 Pp3c1_3040/1-532 99.8% 64.5%  **KVFILTQFNSTSLNRHLARTYNFGK-INF-GDGFVEVLAATQTPGDRGADWFQGTADAVRQYLWLFE-DAKNKVVEDVVI**

18 Pp3c1_31540/1-543 100.0% 59.3%  **KIYVLTQFNSTSLNRHLARTYNFGNGCNF-GDGYVEVLAAAQRPGFGGDKWFEGTADAVRQYLWLLE-DAKNKDVEDVII**

19 Pp3c14_12850/1-541 100.0% 59.9%  **KIYVLTQFNSTSLNRHLARTYNFGNGCNF-GDGYVEVLAAAQRPGSGGDKWFEGTADAVRQYLWLLE-DAKNKDVEDVII**

20 Pp3c17_13940/1-555 100.0% 58.7%  **KIYVLTQFNSTSLNRHLANTYNFGNGCNF-GDGYVEVLAAAQRPGFGGDRWFEGTADAVRQYMWLLE-DAKNKDVEDVVI**

21 Pp3c19_8410/1-532 100.0% 65.6%  **KIFILTQFNSASLNRHLARTYTFGNGVNF-GDGFVEVLAATQTPGEAGMNWFQGTADAVRQFTWLFE-DAKNKQVEHVLI**

22 Pp3c2_35010/1-524 98.7% 46.6%  **KIYVLTQFNSASLNRHLSRAYASNMG-GYKNEGFVEVLAAQQSPEN--PNWFQGTADAVRQYLWLFE----EAQVLEYVI**

23 Pp3c7_20320/1-526 98.7% 46.4%  **KIYVLTQFNSASLNRHLSRAYASNMG-GYKNEGFVEVLAAQQSPEN--PNWFQGTADAVRQYLWLFE----EAQVLEYVI**

24 Pp3c7_20690/1-533 98.7% 45.8%  **KIYVLTQYNSASLNKHVSRAYATNLG-SYRNEGFVEILAAQQSHDN--PNWFQGTADAVRQYLWLFE----EAQVMDYVI**

25 Pp3c7_22280/1-536 99.8% 63.5%  **KVFILTQFNSTSLNRHLARTYNFGK-INF-GDGFVEVLAATQTPGDRGADWFQGTADAVRQYLWLFE-DAKNKVVEDVVI**

26 Pp3s127_20/1-546 100.0% 58.2%  **KIYVLTQFNSTSLNRHLSRTYNLGNGSSF-GDGYVEVLAAAQRPGFGGDKWFGGTADAVRQYLWLLE-DAKNKDVEEVVI**

27 Sacu_v1.1_s0067.g016264/1-532 100.0% 65.4%  **KVFILTQFNSASLNRHLARTYNFGNGVNF-GDGFVEVLAATQRPGETGMNWFQGTADAVRQFTWLLE-DPKNADIEHVVI**

28 Sacu_v1.1_s0095.g019244/1-550 100.0% 64.0%  **KIYILTQFNSFSLNRHLARTYSTGNGVHF-GDGFVEVLAATQTPGETGMNWFQGTADAVRQFTWLFE-DAKNKNIEHVLI**

29 Sacu_v1.1_s0157.g023711/1-526 100.0% 65.4%  **KIYILTQFNSASLNRHIARTYNLGNGVNL-GDSFVEVLAATQRPGEAGMQWFQGTADAVRQFAWLFE-DTKNSDIEHVII**

30 Sacu_v1.1_s0001.g000380/1-536 98.7% 45.8%  **KIYVLTQFNSASLNRHLSRAYASNMG-GYKHEGFVEVLAAQQSPEN--PNWFQGTADAVRQYLWLFE----EQQVMEYLI**

31 Sacu_v1.1_s0076.g017538/1-573 98.7% 43.5%  **MIYVLTQFNSASLNRHLSRAYSSNMG-GYKHEGFVEVLAAQQSPEN--PNWFQGTADAVRQYLWLFE----EQQVMEFLV**

32 Azfi_s0120.g046768/1-488 100.0% 71.5%  **KVFILTQFNSASLNRHLARTYNFGNGVNF-GDGFVEVLAATQRPGETGMNWFQGTADAVRQFTWLLE-DPKNTDLEHVVI**

33 Azfi_s0003.g008048/1-504 98.7% 48.4%  **KIYVLTQFNSASLNRHLSRAYASNMG-GYKREGFVEVLAAQQSPEN--PNWFQGTADAVRQYLWLLE----EQQVMEFLI**

34 Azfi_s0106.g045016/1-528 98.7% 46.6%  **MIYVLTQFNSASLNRHLSRAYSSNMG-GYKHEGFVEVLAAQQSPEN--PNWFQGTADAVRQYLWLFE----EQQVMEFLI**

35 Azfi_s0012.g012923/1-564 98.7% 43.5%  **MIYVLTQFNSASLNRHLSRAYSSNMG-GYKHEGFVEVLAAQQSPEN--PNWFQGTADAVRQYLWLFE----EQQVMEFLI**

36 CHBRA475g00200/1-614 99.8% 51.2%  **KIFILTQFNSTSLNRHLAKTYNSG-GITF-ADGFVEVLAATQTPGEGGQEWFQGTADAVRQYLWLLEQDLSHRDVEDILI**

37 AaBONN_Sc2ySwM_340.122.1/1-513 98.7% 48.0%  **KIYVLTQFNSASLNRHLSRAYASNMG-GYKNEGFVEVLAAQQSPEN--PNWFQGTADAVRQYLWLFE----EQQVLEFLV**

38 Anthoceros/1-550 100.0% 66.0%  **KVFILTQFNSASLNRHLARTYNFGNGVNF-GDGFVEVLAATQTPGEGGMEWFQGTADAVRQYLWLFE-DAKNKQIENVLI**

cov pid **321**  **. . : . . . . 4** **400**

1 Selmo_138695/1-447 100.0% 100.0%  **LSGDHLYRMDYMEFIQKHQDTGADITIGCVP--MDDS--RASDFGLMKIDANGQ-ILYFSEKPKGAD-LK-AMQVD----**

2 Selmo_149205/1-533 99.3% 63.2%  **LSGDHLYRMDYMDFVQKHKDSGADITISCVP--VDES--RASDFGLVKTDARGR-IISFSEKPKGMD-LK-AMQVD----**

3 Selmo_169778/1-463 100.0% 95.2%  **LSGDHLYRMDYMEFIQKHQDTGADITIGCVP--MDDS--RASDFGLMKIDANGQ-ILYFSEKPKGAD-LK-AMQVD----**

4 AT1G27680/1-518 100.0% 64.7%  **LSGDHLYRMDYMNFVQKHIESNADITVSCLP--MDES--RASDFGLLKIDQSGK-IIQFSEKPKGDD-LK-AMQVD----**

5 Bradi1g09537/1-504 100.0% 63.5%  **LSGDHLYRMDYMDFVQSHRQRDAGISICCLP--IDDS--RASDFGLMKIDDTGR-VISFSEKPKGDD-LK-AMQVD----**

6 Bradi1g53500/1-512 100.0% 63.5%  **LSGDHLYRMDYMDFVQKHVDSGADISVACVP--MDES--RASDFGLMKTDRNGR-ITDFLEKPKGES-LK-SMVVD----**

7 Bradi2g14970/1-522 99.6% 54.2%  **LSGDQLYRMDYMELVQKHVDDNADITLSCAP--VGES--RASEYGLVKFDSSGR-VIQFSEKPKGVD-LE-AMKVD----**

8 Cre07.g331300/1-719 98.7% 23.6%  **LPADQLYHTDLEALMEWHRGNEACLTVVAHG--VEGG--DTEHVGLLKVRPSTRELLDYVEKPRTAR-EREPFRVP----**

9 Cre13.g567950/1-512 99.6% 53.9%  **LSGDHLYRMDYMKFVNYHRETNADITIGCIA--YGSD--RAKEFGLMKIDEKRR-VTSFAEKPKTQEALD-AMKVD----**

10 Cre16.g683450/1-560 99.3% 33.2%  **LPGDHVYSADYTPIIAYHRSTGADLTIVCRP--VSGE--QACRLGVVKLDAQNR-IKTFSEKPSASELPELAMSDDEMRP**

11 Mapoly0023s0119/1-534 100.0% 66.7%  **LSGDHLYRMDYMDFVEKHRNSGADITISCVP--MDDS--RASDYGLMKIDDTGR-VLYFSEKPKGAD-LK-AMEVD----**

12 Mapoly0033s0108/1-520 98.7% 47.7%  **LAGDHLYRMDYQNFIQAHRDTNADITVAALP--MDEA--RAEAFGLMKINEKGR-IIEFAEKPKGDE-LK-AMAVD----**

13 Mapoly0101s0020/1-543 100.0% 67.6%  **LSGDHLYRMDYMDFVQKHKDSGADITISCVP--MDDS--RASDYGLMKIDHKGQ-VLYFNEKPKGEQ-LK-SMQVD----**

14 Mapoly0132s0055/1-569 98.9% 47.0%  **LSGDHLYRMNYMDLVQSHHNSGADITVSCVPLSMDDSSCQVSAGGLLRLDHKGR-VLSIHDKAMADNGYGLSMLLE----**

15 Pp3c11_5720/1-539 99.8% 63.3%  **LSGDHLYRMDYMDFVQKHRDSGADITISCVP--MDDS--RASDYGLMKIDDEGR-VLYFSEKPKGND-LK-NMQVD----**

16 Pp3c11_8270/1-524 98.7% 47.0%  **LAGDHLYRMDYQKFIQVHRETNADITVAALP--MDET--RATAFGLMKINDQGR-IIEFAEKPKGDE-LR-AMQVD----**

17 Pp3c1_3040/1-532 99.8% 64.5%  **LSGDHLYRMDYMDFVQKHRDSGADITISCVP--MDDS--RASDYGLMKIDDEGR-VLYFNEKPKGDA-LK-SMQVD----**

18 Pp3c1_31540/1-543 100.0% 59.3%  **LSGDHLYRMDYEDFVQKHKDSGADVTVSCVP--MDDS--RASDYGLMKIDGKGR-INYFNEKPKGDD-LQ-AMQVD----**

19 Pp3c14_12850/1-541 100.0% 59.9%  **LSGDHLYRMDYEDFVQKHKDSGADVTVSCVP--IDDS--RASDYGLMKIDGKGQ-IRHFSEKPKGDD-LH-AMQVD----**

20 Pp3c17_13940/1-555 100.0% 58.7%  **LSGDHLYRMDYQDFVQKHKDSGADVTVSCIP--MDDS--RASDFGLMKIDGKGR-INHFSEKPKGKD-LQ-SMQVD----**

21 Pp3c19_8410/1-532 100.0% 65.6%  **LSGDHLYRMDYMDFIQKHKDSGADITISCVP--MDES--RASDYGLMKIDDKGR-VLYFNEKPRGVD-LE-SMQVD----**

22 Pp3c2_35010/1-524 98.7% 46.6%  **LAGDHLYRMDYQHFIQIHRETNADITVAALP--MDEA--RATAFGLMKINDQGR-IIEFAEKPKGDE-LR-AMQVD----**

23 Pp3c7_20320/1-526 98.7% 46.4%  **LAGDHLYRMDYQHFIQVHRETNADITVAALP--MDEA--RATAFGLMKINDQGR-IIEFAEKPKGDE-LK-AMQVD----**

24 Pp3c7_20690/1-533 98.7% 45.8%  **LGGDHLYRMDYQKFIQVHRDTGADITVAALP--MDEA--RASAFGLMKIDGKGR-IYEFAEKPKEDE-LR-AMQVD----**

25 Pp3c7_22280/1-536 99.8% 63.5%  **LSGDHLYRMDYMDFVQKHRDSGADITISCVP--MDDS--RASDYGLMKIDGEGR-VMSFSEKPKGDD-LK-KMQVD----**

26 Pp3s127_20/1-546 100.0% 58.2%  **LSGDHLYRMDYEDFVQKHKDSGADITVSCVP--MDDS--RASDYGLMKINDKGR-IHYFNEKPKGDE-LQ-SMQVD----**

27 Sacu_v1.1_s0067.g016264/1-532 100.0% 65.4%  **LSGDHLYRMDYMDFVQKHKDTEADITISCVP--IDDS--RASDYGLMKIDDNGR-VLYFSEKPKGDD-LK-AMQVD----**

28 Sacu_v1.1_s0095.g019244/1-550 100.0% 64.0%  **LSGDHLYRMDYMDFIQKHQDTGADITISCVP--MDES--RASDYGLMKIDTRGR-VLYFSEKPTGTD-LK-SMQVD----**

29 Sacu_v1.1_s0157.g023711/1-526 100.0% 65.4%  **LSGDHLYRMDYMDFVQKHKDNNADITISCVP--IDNS--RASDYGLMKIDSNGR-VLYFSEKPKGED-LK-AMEVD----**

30 Sacu_v1.1_s0001.g000380/1-536 98.7% 45.8%  **LAGDHLYRMDYQRFIQVHRETDADITVAALP--MDEK--KATAFGLMKIDSEGR-IIEFAEKPKGEF-LK-AMKVD----**

31 Sacu_v1.1_s0076.g017538/1-573 98.7% 43.5%  **LAGDHLYRMDYQKFIQVHRETNADITVAALP--MDEK--RATGFGLMKIDSEGR-IVEFAEKPKGDF-LK-AMKVD----**

32 Azfi_s0120.g046768/1-488 100.0% 71.5%  **LSGDHLYRMDYMDFVQKHKDSAADITISCVP--IDDS--RASDYGLMKIDSNGR-VLNFAEKPKGDE-LK-AMQVD----**

33 Azfi_s0003.g008048/1-504 98.7% 48.4%  **LAGDHLYRMDYQKFIQVHRESNADITVAALP--MDEK--RATAFGLMKIDSEGR-IVEFAEKPKGEF-LK-AMKVD----**

34 Azfi_s0106.g045016/1-528 98.7% 46.6%  **LAGDHLYRMDYQKFIQVHRETNADITVAALP--MDEE--RATAFGLMKIDSEGR-IVEFAEKPKGDF-LR-GMKVD----**

35 Azfi_s0012.g012923/1-564 98.7% 43.5%  **LAGDHLYRMDYQKFIQVHRETNADITVAALP--MDEK--RATGFGLMKIDSEGR-IVEFAEKPKGDF-LR-AMKVD----**

36 CHBRA475g00200/1-614 99.8% 51.2%  **LSGDHLYRMDYMDFVQRHKDTGADITISVLP--IDDS--RASDYGLMKTDDKGR-VVSFAEKPKGQE-LA-AMAVD----**

37 AaBONN_Sc2ySwM_340.122.1/1-513 98.7% 48.0%  **LAGDHLYRMDYQKFIQVHRETNADITVAALP--MDEK--RATAFGLMKIDDQGR-IIEFAEKPKGDA-LS-AMKVD----**

38 Anthoceros/1-550 100.0% 66.0%  **LSGDHLYRMDYMDFVQKHKDSGADITISCVP--MDDS--RASDFGLMKIDDKGQ-IVSFSEKPKGAD-LR-AMQVD----**

cov pid **401**  **. . . . : . . .** **480**

1 Selmo_138695/1-447 100.0% 100.0%  **-----------------TTVL---GLTP-------------------------------EEAIEKP----YIASMGIYVF**

2 Selmo_149205/1-533 99.3% 63.2%  **-----------------TTAL---GLSR-------------------------------EEAKKMP----YIASMGIYVF**

3 Selmo_169778/1-463 100.0% 95.2%  **-----------------TTVL---GLTP-------------------------------EEAIEKP----YIASMGIYVF**

4 AT1G27680/1-518 100.0% 64.7%  **-----------------TSIL---GLPP-------------------------------KEAAESP----YIASMGVYVF**

5 Bradi1g09537/1-504 100.0% 63.5%  **-----------------TTVL---GLSK-------------------------------EEAEEKP----YIASMGVYIF**

6 Bradi1g53500/1-512 100.0% 63.5%  **-----------------MEIF---GLSP-------------------------------EVANVYN----YLASMGIYVF**

7 Bradi2g14970/1-522 99.6% 54.2%  **-----------------TSFL---NFAI-------------------------------DDPAKFP----YIASMGVYVF**

8 Cre07.g331300/1-719 98.7% 23.6%  **-----------------AEHV---G----------------------------------KLTNGRP----FLASCGIYVF**

9 Cre13.g567950/1-512 99.6% 53.9%  **-----------------TTVL---GLTP-------------------------------EEAAEKP----YIASMGIYVF**

10 Cre16.g683450/1-560 99.3% 33.2%  **FMMPTEEALRQQARRRMQTTLASRGSGPLGRLSAGAAKGGARMGGGGGGRVVLDTMTSVDETETRPGTTGYVGSCGIYIF**

11 Mapoly0023s0119/1-534 100.0% 66.7%  **-----------------TSVL---GLSA-------------------------------EDAKSKP----YIASMGIYVF**

12 Mapoly0033s0108/1-520 98.7% 47.7%  **-----------------TTVL---GLDE-------------------------------ARAKEMP----YIASMGIYVV**

13 Mapoly0101s0020/1-543 100.0% 67.6%  **-----------------TTVL---GLSP-------------------------------EEAKKMP----YIASMGIYVF**

14 Mapoly0132s0055/1-569 98.9% 47.0%  **-----------------SAAAAGSGLPE-------------------------------GAAKNSP----FVASMGLYVF**

15 Pp3c11_5720/1-539 99.8% 63.3%  **-----------------TTVL---GLSP-------------------------------EEAVEKP----YIASMGIYVF**

16 Pp3c11_8270/1-524 98.7% 47.0%  **-----------------TTVL---GLDA-------------------------------ARAKEMP----YIASMGIYVV**

17 Pp3c1_3040/1-532 99.8% 64.5%  **-----------------TTVL---GLTP-------------------------------EEAAAKP----YIASMGIYVF**

18 Pp3c1_31540/1-543 100.0% 59.3%  **-----------------TSVL---GLSS-------------------------------EEAKKKP----YIASMGIYVF**

19 Pp3c14_12850/1-541 100.0% 59.9%  **-----------------TTVL---GLSG-------------------------------EEAKKKP----YIASMGVYVF**

20 Pp3c17_13940/1-555 100.0% 58.7%  **-----------------TTVL---GLSA-------------------------------EEAQKKP----YIASMGIYVF**

21 Pp3c19_8410/1-532 100.0% 65.6%  **-----------------TSVL---GLSP-------------------------------EEAKKMP----YIASMGIYVF**

22 Pp3c2_35010/1-524 98.7% 46.6%  **-----------------TTVL---GLDA-------------------------------ERAKEMP----YIASMGIYVV**

23 Pp3c7_20320/1-526 98.7% 46.4%  **-----------------TTVL---GLDA-------------------------------ERAKEMP----YIASMGIYVV**

24 Pp3c7_20690/1-533 98.7% 45.8%  **-----------------TTVL---GLDS-------------------------------ERAKLKS----YIASMGIYVV**

25 Pp3c7_22280/1-536 99.8% 63.5%  **-----------------TTIL---GLSP-------------------------------EEAAEKP----YIASMGIYVF**

26 Pp3s127_20/1-546 100.0% 58.2%  **-----------------TTVL---GLSP-------------------------------DEAKKKP----YIASMGIYVF**

27 Sacu_v1.1_s0067.g016264/1-532 100.0% 65.4%  **-----------------TSVL---GLDP-------------------------------VAAKESP----YIASMGVYVF**

28 Sacu_v1.1_s0095.g019244/1-550 100.0% 64.0%  **-----------------TTVL---GLSS-------------------------------QEARKKP----YIASMGIYAF**

29 Sacu_v1.1_s0157.g023711/1-526 100.0% 65.4%  **-----------------TTVL---GLSP-------------------------------AQAEKTP----YIASMGVYVF**

30 Sacu_v1.1_s0001.g000380/1-536 98.7% 45.8%  **-----------------TTIL---GLDE-------------------------------ERAKEQP----FIASMGIYVI**

31 Sacu_v1.1_s0076.g017538/1-573 98.7% 43.5%  **-----------------TTIL---GLDE-------------------------------ERAKELP----YIASMGIYVV**

32 Azfi_s0120.g046768/1-488 100.0% 71.5%  **-----------------TTVL---GLDP-------------------------------EAAKESP----YIASMGVYVF**

33 Azfi_s0003.g008048/1-504 98.7% 48.4%  **-----------------TTIL---GLDE-------------------------------DRAKELP----FIASMGIYVI**

34 Azfi_s0106.g045016/1-528 98.7% 46.6%  **-----------------TTIL---GLDE-------------------------------ERAKELP----YIASMGIYVV**

35 Azfi_s0012.g012923/1-564 98.7% 43.5%  **-----------------TTIL---GLDE-------------------------------ERAKDLP----YIASMGIYVV**

36 CHBRA475g00200/1-614 99.8% 51.2%  **-----------------TTIL---GLSA-------------------------------AEAKEKP----YIASMGIYVF**

37 AaBONN_Sc2ySwM_340.122.1/1-513 98.7% 48.0%  **-----------------TTVL---GLDE-------------------------------ERAKELP----YIASMGIYVV**

38 Anthoceros/1-550 100.0% 66.0%  **-----------------TTVL---GLSA-------------------------------EEAKKKP----YIASMGIYVF**

cov pid **481**  **. 5 . . . . : .** **560**

1 Selmo_138695/1-447 100.0% 100.0%  **KKDILLKLLRWRYPTANDFGSEILPASAKE--------------------------------------YNVQAYLFND-Y**

2 Selmo_149205/1-533 99.3% 63.2%  **RKDVLLKLLRWRYPTSNDFGSEIIPAAANE--------------------------------------YNVQAYLFND-Y**

3 Selmo_169778/1-463 100.0% 95.2%  **KKDILLKLLRWRYPTANDFGSEILPASAKE--------------------------------------YNVQAYLFND-Y**

4 AT1G27680/1-518 100.0% 64.7%  **RKEVLLKLLRSSYPTSNDFGSEIIPLAVGE--------------------------------------HNVQAFLFND-Y**

5 Bradi1g09537/1-504 100.0% 63.5%  **KKEILLNLLRWRFPTANDFGSEIIPAAAKE--------------------------------------INVKAYLFND-Y**

6 Bradi1g53500/1-512 100.0% 63.5%  **KTDVLLRLLRGHYPTANDFGSEVIPMAAKD--------------------------------------YNVQAYLFDG-Y**

7 Bradi2g14970/1-522 99.6% 54.2%  **KRDVLLNLLKSRYAELHDFGSEILPRALHE--------------------------------------HNVQAYVFTD-Y**

8 Cre07.g331300/1-719 98.7% 23.6%  **DKHALLDVLS-AHPRAHDFGRGVIPHAFDMAARSLKRRHQQARQRQERQERAAAAGVQLRPGYEYVHPYKVLTWTMAGRY**

9 Cre13.g567950/1-512 99.6% 53.9%  **KKSVLLQLLNDSYAKANDFGGEIIPSAAKD--------------------------------------HNVVAYPFYG-Y**

10 Cre16.g683450/1-560 99.3% 33.2%  **KRSVLSEALKRHF-KMQDFGRQIIPELIREG-------------------------------------VKVHAYRLPG-Y**

11 Mapoly0023s0119/1-534 100.0% 66.7%  **KKEILQKLLRWRYPTANDFGSEIIPASAKE--------------------------------------FNVQAYLFDD-Y**

12 Mapoly0033s0108/1-520 98.7% 47.7%  **SKDAMIKLLRDDFPEANDFGSEVIPGATKLG-------------------------------------MSVQAYLYDG-Y**

13 Mapoly0101s0020/1-543 100.0% 67.6%  **KKEILLKLLRWRYPTANDFGSEIIPASAKE--------------------------------------FNVQAYLFND-Y**

14 Mapoly0132s0055/1-569 98.9% 47.0%  **KKDVLIKLLKWIYPNSNDFASEIIPAAAKD--------------------------------------FYVQAYLFRN-Y**

15 Pp3c11_5720/1-539 99.8% 63.3%  **KKDVLMKLLRWRYPTANDFGSEIIPASAKE--------------------------------------FNVQAYLFND-Y**

16 Pp3c11_8270/1-524 98.7% 47.0%  **SKEAMISLLRNDFPEANDFGSEVIPGATEMG-------------------------------------MKVQAYLYDG-Y**

17 Pp3c1_3040/1-532 99.8% 64.5%  **KKDVLLKLLRWRYPTANDFGSEIIPASAKE--------------------------------------FNVQAYLFND-Y**

18 Pp3c1_31540/1-543 100.0% 59.3%  **KKSVLTKLLRWRYPLSNDFGSEIIPQAAKE--------------------------------------FNVHAYLYND-Y**

19 Pp3c14_12850/1-541 100.0% 59.9%  **KKSVLAKLLRWRYPLANDFGSEIIPQAAKE--------------------------------------FNVQAHLFNG-Y**

20 Pp3c17_13940/1-555 100.0% 58.7%  **KKSVLAKLLRWRYPLANDFGSEIIPKAAKE--------------------------------------FNVNAYLFND-Y**

21 Pp3c19_8410/1-532 100.0% 65.6%  **RKDILLKLLRWRYPTSNDFGSEIIPAAAKE--------------------------------------YNVQAYLFND-Y**

22 Pp3c2_35010/1-524 98.7% 46.6%  **SKEAMISLLRNDFPEANDFGSEVIPGATKMG-------------------------------------MKVQAYLYDG-Y**

23 Pp3c7_20320/1-526 98.7% 46.4%  **SKEAMITLLRNEFPEANDFGSEVIPGATKMG-------------------------------------MKVQAYLYDG-Y**

24 Pp3c7_20690/1-533 98.7% 45.8%  **RKEAMVSLLRKDFPEANDFGSEVIPGATKSG-------------------------------------MKVQAYLFDG-Y**

25 Pp3c7_22280/1-536 99.8% 63.5%  **KKDVLMKLLRWRYPTANDFGSEIIPASAKE--------------------------------------FNVQAYLFNS-Y**

26 Pp3s127_20/1-546 100.0% 58.2%  **KKSVLSKLLKWRYPLANDFGSEIIPQAAKE--------------------------------------FYVHAYLFND-Y**

27 Sacu_v1.1_s0067.g016264/1-532 100.0% 65.4%  **RKDILLKLLRWRFPTANDFGSEIIPASAKE--------------------------------------YNMRAYLFDG-Y**

28 Sacu_v1.1_s0095.g019244/1-550 100.0% 64.0%  **KMDVLLKLLRWRYPTSNDFGSEIIPASTKE--------------------------------------YNVQAYLFNG-Y**

29 Sacu_v1.1_s0157.g023711/1-526 100.0% 65.4%  **KKDILLKLLRWRYPTANDFGSEIIPASAKE--------------------------------------CNVQAYLFDD-Y**

30 Sacu_v1.1_s0001.g000380/1-536 98.7% 45.8%  **NKDAMLRLLRDEFPQANDFGSEVIPGATSLG-------------------------------------MKVQAYLYDG-Y**

31 Sacu_v1.1_s0076.g017538/1-573 98.7% 43.5%  **SKDVMIKLLRDEFPQANDFGSEVIPGATSLG-------------------------------------MKVQAYLYDG-Y**

32 Azfi_s0120.g046768/1-488 100.0% 71.5%  **KKDILLKLLRWRYPTANDFGSEIIPASAKE--------------------------------------YNVKAYLFKG-Y**

33 Azfi_s0003.g008048/1-504 98.7% 48.4%  **SKDAMIKLLRDEFPQANDFGSEVIPGATSLG-------------------------------------MRVQAYLFDG-Y**

34 Azfi_s0106.g045016/1-528 98.7% 46.6%  **NKDAMIKLLKDDFPNANDFGSEVIPGATALG-------------------------------------MKVQAYLYDG-Y**

35 Azfi_s0012.g012923/1-564 98.7% 43.5%  **SKDAMIKLLRDDFPQANDFGSEVIPGATSIG-------------------------------------MKVQAYLYDG-Y**

36 CHBRA475g00200/1-614 99.8% 51.2%  **KKKTLIELLRSRYPHANDFGSEIIPSSAGE--------------------------------------FNVQAYLFND-Y**

37 AaBONN_Sc2ySwM_340.122.1/1-513 98.7% 48.0%  **SKEAMIKLLRNDFPEANDFGSEVIPGATRIG-------------------------------------MKVQAYLYDG-Y**

38 Anthoceros/1-550 100.0% 66.0%  **KKDILLKLLRWRYPLANDFGSEIIPASAKE--------------------------------------FNVQAYLFND-Y**

cov pid **561**  **. . . 6 . . . .** **640**

1 Selmo_138695/1-447 100.0% 100.0%  **WEDIG-TIKSFYEA--NLALTCQP-PKFRFYDA-A---KPIYTSPRYLPPTKIEK-CRVLDSIVSHGCFLQ---------**

2 Selmo_149205/1-533 99.3% 63.2%  **WEDIG-TIKSFFDA--NLALTAQP-PKFSFYDA-S---NPIFTSPRFLPPTKMEK-CRIIDSIVSHGCFLK---------**

3 Selmo_169778/1-463 100.0% 95.2%  **WEDIG-TIKSFYEA--NLALTCQP-PKFRFYDA-A---KPIYTSPRYLPPTKIEK-CRVLDSIVSHGCFLQ---------**

4 AT1G27680/1-518 100.0% 64.7%  **WEDIG-TIGSFFDA--NLALTEQP-PKFQFYDQ-K---TPFFTSPRFLPPTKVDK-CRILDSIVSHGCFLR---------**

5 Bradi1g09537/1-504 100.0% 63.5%  **WEDIG-TIKSFFEA--NLALAEQP-PRFSFYDA-S---KPMYTSRRNLPPSMISS-SKITDSIISHGCFLD---------**

6 Bradi1g53500/1-512 100.0% 63.5%  **WEDIG-TIKSFFEA--NLALTDQS-PNFHFYEP-V---KPIFTSPRFLPPTKVED-CKVLNSIVSHGCFLT---------**

7 Bradi2g14970/1-522 99.6% 54.2%  **WEDIG-TIRSFFDA--NMALCEQP-PKFEFYDP-K---TPFFTSPRYLPPTKSDK-CRIKEAIISHGCFLR---------**

8 Cre07.g331300/1-719 98.7% 23.6%  **WADVGNSIRTYKEATQHVLLGRGPGDAETPFDT-AYHHELIITGSALLPPAQLSPGCRLVRSALSPGCRVG---------**

9 Cre13.g567950/1-512 99.6% 53.9%  **WEDIG-TIKSFFEE--NLKLCRHP-ATFEFYDP-Q---SPIYTSPRVLPPATVRN-CKVTDAIIAQGSFVS---------**

10 Cre16.g683450/1-560 99.3% 33.2%  **WADVGGSVGDFYAA--NMSLLSDP-PSISFNAPIN---SPFFKFPLTIPASQMMH-SRVSGALVSAGCIVN---------**

11 Mapoly0023s0119/1-534 100.0% 66.7%  **WEDIG-TIKSFFDA--NLGLTAQP-PKFSFYDA-V---KPIFTSPRYLPPSKIEK-CRVEDSIISHGCFLR---------**

12 Mapoly0033s0108/1-520 98.7% 47.7%  **WEDIG-TIEAFYNA--NLGLTKKPVPDFSFYDR-T---SPIYTQARFLPPSKMLD-ADVTDSVIGEGCVIK---------**

13 Mapoly0101s0020/1-543 100.0% 67.6%  **WEDIG-TIKSFFDA--NLALTEQP-PKFKFYDV-A---KPIFTSPRYLPPTKVEK-CRIIDSIVSHGCFLR---------**

14 Mapoly0132s0055/1-569 98.9% 47.0%  **WQDVG-SIESYFEA--NLALTEES-PKFEFYDV-S---NPIYTSPRYLPPTTVDH-CRIVDSIVSHGCFLR---------**

15 Pp3c11_5720/1-539 99.8% 63.3%  **WEDIG-TIKSFFDA--NLALTAQP-PQFSFYDA-A---KPIFTSPRYLPPTSIEQ-CMIKDSIVSHGCFLK---------**

16 Pp3c11_8270/1-524 98.7% 47.0%  **WEDIG-TIEAFYNA--NLGITKKPVPDFSFYDR-S---APIYTQARFLPPSKMLD-ADVTDSVIGEGCVIK---------**

17 Pp3c1_3040/1-532 99.8% 64.5%  **WEDIG-TIKSFFDA--NLALTSQP-PQFSFYDA-T---KPIFTSPRYLPPTKIEQ-CQVVDSIISHGCILK---------**

18 Pp3c1_31540/1-543 100.0% 59.3%  **WEDIG-TIKSFFDA--NLALTSPN-SKFSFYDA-A---KPTYTSARYLPPTKIEK-CIVKDSIVSHGCFLR---------**

19 Pp3c14_12850/1-541 100.0% 59.9%  **WEDIG-TIKSFFDA--NLALTAEN-PKFSFFDA-A---KPIYTSARYLPPTKIEK-CRVKDSIVSHGCFLR---------**

20 Pp3c17_13940/1-555 100.0% 58.7%  **WEDIG-TIKSFFDA--NLALAAEN-PNFSFYDA-E---KPIYTSARYLPPTKIEK-CRVKDSIVSHGCFLR---------**

21 Pp3c19_8410/1-532 100.0% 65.6%  **WEDIG-TIKSFFDA--NLALAAQP-PKFKFYDA-T---KPIFTSPRYLPPTKVEQ-CRIIHSIVSHGCFLR---------**

22 Pp3c2_35010/1-524 98.7% 46.6%  **WEDIG-TIEAFYNA--NLGITKKPVPDFSFYDR-S---SPIYTQARFLPPSKMLD-ADVTDSVIGEGCVIK---------**

23 Pp3c7_20320/1-526 98.7% 46.4%  **WEDIG-TIEAFYNA--NLGITKKPVPDFSFYDR-S---APIYTQARFLPPSKMLD-ADVTDSVIGEGCVIK---------**

24 Pp3c7_20690/1-533 98.7% 45.8%  **WEDIG-TIEAFYNA--NLGITKKPVPDFSFYDR-I---SPIYTQPRFLPPSKMMD-ADIRDSVIGEGCIIR---------**

25 Pp3c7_22280/1-536 99.8% 63.5%  **WEDIG-TIKSFFDA--NLALTAQP-PQFSFYDA-A---KPIFTSPRYLPPTSIEQ-CMVKDSIISHGCFLK---------**

26 Pp3s127_20/1-546 100.0% 58.2%  **WEDIG-TIKSFFDA--NLALTSPD-SKFSFYDA-A---KPTYTSARYLPPTKIER-CRVKDSIVSHGCFLR---------**

27 Sacu_v1.1_s0067.g016264/1-532 100.0% 65.4%  **WEDIG-TIRSFYEA--NLALTAQP-PKFSFYDA-T---KPIYTSPRYLPPTKVDS-CRVVTSIISHGCFLQ---------**

28 Sacu_v1.1_s0095.g019244/1-550 100.0% 64.0%  **WEDIG-TIRSFFDA--NLALTAES-PNFEFYDA-A---KPIYTSPRYLPPTKIER-CRVIDSIISHGCFLR---------**

29 Sacu_v1.1_s0157.g023711/1-526 100.0% 65.4%  **WEDIG-TIRSFYEA--NLALTAQP-AKFSFYDA-A---KPIYTSPRYLPPTKVDR-CKVVGSIISHGCFLR---------**

30 Sacu_v1.1_s0001.g000380/1-536 98.7% 45.8%  **WEDIG-TIEAFYNA--NLGITKKPFPDFSFYDQ-S---SPIYTQARFLPPSKMVD-ADVSESVIGDGCIIK---------**

31 Sacu_v1.1_s0076.g017538/1-573 98.7% 43.5%  **WEDIG-TIEAFYNA--NLGITKKPFPDFSFYDR-S---APIYTQPRFLPPSKVID-ADVTDSVIGEGCLIKVQELITIEV**

32 Azfi_s0120.g046768/1-488 100.0% 71.5%  **WEDIG-TIRSFYEA--NLALTAQP-PKFSFYDA-A---KPIYTSARYLPPTKVDS-CRILGSILSHGCFLQ---------**

33 Azfi_s0003.g008048/1-504 98.7% 48.4%  **WEDIG-TIEAFYNA--NLGITKKPFPDFSFYDR-S---SPIYTQARFLPPSKMVD-ADITESVIGDGCIIK---------**

34 Azfi_s0106.g045016/1-528 98.7% 46.6%  **WEDIG-TIEAFYNA--NLGITKKP-PDFSFYDR-S---APIYTQPRFLPPSKVVD-ADVTDSVIGEGCLIK---------**

35 Azfi_s0012.g012923/1-564 98.7% 43.5%  **WEDIG-TIEAFYNA--NLGVTKKPFPDFSFYDR-S---APIYTQPRFLPPSKVVD-ADVTDSVIGEGCLIK---------**

36 CHBRA475g00200/1-614 99.8% 51.2%  **WEDIG-TMRSFFEA--NLGLTRHP-ARFSFYDA-Q---KPIYTSPRNLPPSKIEK-CRVKDSIISHGCFLR---------**

37 AaBONN_Sc2ySwM_340.122.1/1-513 98.7% 48.0%  **WEDIG-TIEAFYNA--NLGITKKPIPDFSFYDR-S---APIYTLARFLPPSKMLD-ADVTDSVIGEGCVIK---------**

38 Anthoceros/1-550 100.0% 66.0%  **WEDIG-TIKSFYDA--NLALTAQP-PKFSFYDA-N---KPIFTSPRYLPPTKVDK-CRIQDSIVSHGCFLR---------**

cov pid **641**  **: . . . . 7 . .** **720**

1 Selmo_138695/1-447 100.0% 100.0%  **----------------------------------------------------ECSVTHSVIGIRSRVEAGAEIQDTMMLG**

2 Selmo_149205/1-533 99.3% 63.2%  **----------------------------------------------------SCSVEHSLIGVRSRLESGVELKDTIIMG**

3 Selmo_169778/1-463 100.0% 95.2%  **----------------------------------------------------ECSVTHSVIGIRSRVEAGAEIQDTMMLG**

4 AT1G27680/1-518 100.0% 64.7%  **----------------------------------------------------ECSVQHSIVGIRSRLESGVELQDTMMMG**

5 Bradi1g09537/1-504 100.0% 63.5%  **----------------------------------------------------NCRVEHSVVGVRSRVGSNVHLKDTVMLG**

6 Bradi1g53500/1-512 100.0% 63.5%  **----------------------------------------------------ECSVEHSVIGIRSRLQPGVQLKDTMMMG**

7 Bradi2g14970/1-522 99.6% 54.2%  **----------------------------------------------------ECTIEHSIVGVRSRLNSGCELKNAMMMG**

8 Cre07.g331300/1-719 98.7% 23.6%  **---------------------------------------------------PGAVVENSVLGPRAVVGAGAVVRNSVVMG**

9 Cre13.g567950/1-512 99.6% 53.9%  **----------------------------------------------------DCTINNAVIGIRSIIGQNCTIQDALVMG**

10 Cre16.g683450/1-560 99.3% 33.2%  **----------------------------------------------------RSDIRNSVIGSRSIIGPNVTIEDSVVFG**

11 Mapoly0023s0119/1-534 100.0% 66.7%  **----------------------------------------------------DCSIKHSIVGIRSQMASGSALKDTMMLG**

12 Mapoly0033s0108/1-520 98.7% 47.7%  **----------------------------------------------------NCKIYHSVVGLRSWIAEGAIVEDALLMG**

13 Mapoly0101s0020/1-543 100.0% 67.6%  **----------------------------------------------------DCSVEHSIVGIRSRIESGVELQDTMMMG**

14 Mapoly0132s0055/1-569 98.9% 47.0%  **----------------------------------------------------SCSVQHSIIGIRSRIETGVELKDVVMIG**

15 Pp3c11_5720/1-539 99.8% 63.3%  **----------------------------------------------------NCSVEHSIVGVRSRLEFGSVLKDTMMMG**

16 Pp3c11_8270/1-524 98.7% 47.0%  **----------------------------------------------------NARIYHSVVGLRSWVAEGAVIEDALLMG**

17 Pp3c1_3040/1-532 99.8% 64.5%  **----------------------------------------------------GCSVNHSIVGIRSRLQEGIVLKDTMMMG**

18 Pp3c1_31540/1-543 100.0% 59.3%  **----------------------------------------------------DCSVENSIVGIRSRLESGCDVKRAMIMG**

19 Pp3c14_12850/1-541 100.0% 59.9%  **----------------------------------------------------ECSLENSVIGVRSRLESGCDVKRSMVMG**

20 Pp3c17_13940/1-555 100.0% 58.7%  **----------------------------------------------------ECSVEDSVIGIRSRLEAGCDVKRAMVMG**

21 Pp3c19_8410/1-532 100.0% 65.6%  **----------------------------------------------------DCKVEHSLIGLRSRLESGVEVKNTMMLG**

22 Pp3c2_35010/1-524 98.7% 46.6%  **----------------------------------------------------NARIFHSVVGLRSWVSEGAVIEDALLMG**

23 Pp3c7_20320/1-526 98.7% 46.4%  **----------------------------------------------------NAKIFHSVVGLRSWVAEGAVVEDALLMG**

24 Pp3c7_20690/1-533 98.7% 45.8%  **----------------------------------------------------NAAIYHSSLGLRSWVSEGAVVEDTLLMG**

25 Pp3c7_22280/1-536 99.8% 63.5%  **----------------------------------------------------KCSVEHSIVGVRSRLESGSVLKDTMMMG**

26 Pp3s127_20/1-546 100.0% 58.2%  **----------------------------------------------------DCSVENSVIGIRSRLESGCDVKRAMIMG**

27 Sacu_v1.1_s0067.g016264/1-532 100.0% 65.4%  **----------------------------------------------------DCSVEHSIVGIRSRIENGVELKDTVMVG**

28 Sacu_v1.1_s0095.g019244/1-550 100.0% 64.0%  **----------------------------------------------------DCSVNHCIIGVRSRIDSGCELEDVMMMG**

29 Sacu_v1.1_s0157.g023711/1-526 100.0% 65.4%  **----------------------------------------------------ECTVEHSIVGIRSRIEDGVELKDTVMLG**

30 Sacu_v1.1_s0001.g000380/1-536 98.7% 45.8%  **----------------------------------------------------NCKIHHSVIGLRSWISEGAIIEDALLMG**

31 Sacu_v1.1_s0076.g017538/1-573 98.7% 43.5%  **ADVDLTTVKRKIKICWNSWTRFRGLFLLKLFFGELILLPVRYITARIFMVLQNCKIHHSVIGLRSWISEGAIIEDALLMG**

32 Azfi_s0120.g046768/1-488 100.0% 71.5%  **----------------------------------------------------SCSVEHSIVGIRSRIENGVELKDTVMLG**

33 Azfi_s0003.g008048/1-504 98.7% 48.4%  **----------------------------------------------------NCKIHHSVIGLRSWISEGAIIEDTLLMG**

34 Azfi_s0106.g045016/1-528 98.7% 46.6%  **----------------------------------------------------NCKIHHSVIGLRSWISEGAIIEDALLMG**

35 Azfi_s0012.g012923/1-564 98.7% 43.5%  **----------------------------------------------------NCKIHHSVIGLRSWISEGAIIEDALLMG**

36 CHBRA475g00200/1-614 99.8% 51.2%  **----------------------------------------------------SCAVKHSIIGLRSRVEQGVDIADTMMMG**

37 AaBONN_Sc2ySwM_340.122.1/1-513 98.7% 48.0%  **----------------------------------------------------NCKIHHSVVGLRSWVGEGAVIEDALLMG**

38 Anthoceros/1-550 100.0% 66.0%  **----------------------------------------------------ECSVVHSIVGVRSRLENGVELKDTLMLG**

cov pid **721**  **. . : . . . . 8** **800**

1 Selmo_138695/1-447 100.0% 100.0%  **ADFYETEAEIASMV------------------------------------------------------------------**

2 Selmo_149205/1-533 99.3% 63.2%  **ADSYETEAEIAALR------------------------------------------------------------------**

3 Selmo_169778/1-463 100.0% 95.2%  **ADFYETEAEIASMV------------------------------------------------------------------**

4 AT1G27680/1-518 100.0% 64.7%  **ADFYQTEAEIASLL------------------------------------------------------------------**

5 Bradi1g09537/1-504 100.0% 63.5%  **ADFYETDVERSDQL------------------------------------------------------------------**

6 Bradi1g53500/1-512 100.0% 63.5%  **ADYYQTEAERFSEL------------------------------------------------------------------**

7 Bradi2g14970/1-522 99.6% 54.2%  **ADLYETEDEISRLL------------------------------------------------------------------**

8 Cre07.g331300/1-719 98.7% 23.6%  **ADYYDEDLRKGALRRRYGSGAAAGAGTGGTGSSHDSMEGPSLPPAPTPTPTTTTSSGGGGGSSSSSSGGGSSGGGSSSSA**

9 Cre13.g567950/1-512 99.6% 53.9%  **ADYYESDDQRATLL------------------------------------------------------------------**

10 Cre16.g683450/1-560 99.3% 33.2%  **ASHYDHEKPLPRPL------------------------------------------------------------------**

11 Mapoly0023s0119/1-534 100.0% 66.7%  **ADYYETDAERAALI------------------------------------------------------------------**

12 Mapoly0033s0108/1-520 98.7% 47.7%  **ADYYETDEQRSELL------------------------------------------------------------------**

13 Mapoly0101s0020/1-543 100.0% 67.6%  **ADYYETDAEMASLL------------------------------------------------------------------**

14 Mapoly0132s0055/1-569 98.9% 47.0%  **ADDYETEEERSRLL------------------------------------------------------------------**

15 Pp3c11_5720/1-539 99.8% 63.3%  **ADYYETEDEVAAML------------------------------------------------------------------**

16 Pp3c11_8270/1-524 98.7% 47.0%  **ADYYETDDQRNELL------------------------------------------------------------------**

17 Pp3c1_3040/1-532 99.8% 64.5%  **ADFYQTEEEVAQLL------------------------------------------------------------------**

18 Pp3c1_31540/1-543 100.0% 59.3%  **ADYYETDPEAAALL------------------------------------------------------------------**

19 Pp3c14_12850/1-541 100.0% 59.9%  **ADFYETDPEAAALL------------------------------------------------------------------**

20 Pp3c17_13940/1-555 100.0% 58.7%  **ADSYETDPEAAALL------------------------------------------------------------------**

21 Pp3c19_8410/1-532 100.0% 65.6%  **ADFYETDEERVALI------------------------------------------------------------------**

22 Pp3c2_35010/1-524 98.7% 46.6%  **ADYYETDKQRNELL------------------------------------------------------------------**

23 Pp3c7_20320/1-526 98.7% 46.4%  **ADYYETDEQRNELL------------------------------------------------------------------**

24 Pp3c7_20690/1-533 98.7% 45.8%  **ADYYETDQQRRELL------------------------------------------------------------------**

25 Pp3c7_22280/1-536 99.8% 63.5%  **ADFYDTEKEVADML------------------------------------------------------------------**

26 Pp3s127_20/1-546 100.0% 58.2%  **ADFYETDPEASALQ------------------------------------------------------------------**

27 Sacu_v1.1_s0067.g016264/1-532 100.0% 65.4%  **ADYYETDAERAALL------------------------------------------------------------------**

28 Sacu_v1.1_s0095.g019244/1-550 100.0% 64.0%  **ADVYETEVEMAALL------------------------------------------------------------------**

29 Sacu_v1.1_s0157.g023711/1-526 100.0% 65.4%  **ADYYETDAERAAIL------------------------------------------------------------------**

30 Sacu_v1.1_s0001.g000380/1-536 98.7% 45.8%  **ADYYETDEQREIVT------------------------------------------------------------------**

31 Sacu_v1.1_s0076.g017538/1-573 98.7% 43.5%  **ADYYETDEQRDAVI------------------------------------------------------------------**

32 Azfi_s0120.g046768/1-488 100.0% 71.5%  **ADFYETDAERASLL------------------------------------------------------------------**

33 Azfi_s0003.g008048/1-504 98.7% 48.4%  **ADYYETDEQRQIVT------------------------------------------------------------------**

34 Azfi_s0106.g045016/1-528 98.7% 46.6%  **ADYYETDDQRSSVI------------------------------------------------------------------**

35 Azfi_s0012.g012923/1-564 98.7% 43.5%  **ADYYETKEQREAVI------------------------------------------------------------------**

36 CHBRA475g00200/1-614 99.8% 51.2%  **ADYYETDAERAALL------------------------------------------------------------------**

37 AaBONN_Sc2ySwM_340.122.1/1-513 98.7% 48.0%  **ADYYETDGQRQELL------------------------------------------------------------------**

38 Anthoceros/1-550 100.0% 66.0%  **ADVYETDVERAALL------------------------------------------------------------------**

cov pid **801**  **. . . . : . . .** **880**

1 Selmo_138695/1-447 100.0% 100.0%  **----AEGKV------------------------------------------PVGVGQNAKIRNCILDKNVRIGKNVVIAN**

2 Selmo_149205/1-533 99.3% 63.2%  **----AQGKV------------------------------------------PLGVGEHTTMRNCLVDKNARIGSHVIITN**

3 Selmo_169778/1-463 100.0% 95.2%  **----AEGKV------------------------------------------PVGVGQNAKIRNCILDKNVRIGKNVVIAN**

4 AT1G27680/1-518 100.0% 64.7%  **----AEGKV------------------------------------------PVGVGQNTKIKNCIIDKNAKIGKNVVIAN**

5 Bradi1g09537/1-504 100.0% 63.5%  **----AEGKV------------------------------------------PIGIGENTTIQNCIIDKNARIGKNVTIAN**

6 Bradi1g53500/1-512 100.0% 63.5%  **----SDGKV------------------------------------------PVGVGENTKIRNCIIDKNARIGKNVVIMN**

7 Bradi2g14970/1-522 99.6% 54.2%  **----SEGKV------------------------------------------PIGVGENAKISNCIIDMNARIGRDVIIAN**

8 Cre07.g331300/1-719 98.7% 23.6%  **HGRGSAGAVLLTAAQRAAGVYSMDPAEMERRGGGGGGGGGSETETDDPSVPPLGIGAGAVVEGALVDKNARIGTGCVVAN**

9 Cre13.g567950/1-512 99.6% 53.9%  **----KKGGV------------------------------------------PVGIGANSVITNAIIDKNARVGKNVKIVN**

10 Cre16.g683450/1-560 99.3% 33.2%  **----GPTFP------------------------------------------PMGIGEGSIVRNAILDLNVRVGKNVQLVN**

11 Mapoly0023s0119/1-534 100.0% 66.7%  **----AEGKV------------------------------------------PIGVGANCKISNCIIDKNARIGSNVVIAN**

12 Mapoly0033s0108/1-520 98.7% 47.7%  **----ATGGI------------------------------------------PMGIGRNSIIKRAIIDKNARIGENVKIVN**

13 Mapoly0101s0020/1-543 100.0% 67.6%  **----ASGKV------------------------------------------PLGVGTNSKIRNCIIDKNSRIGRNVIIMN**

14 Mapoly0132s0055/1-569 98.9% 47.0%  **----AIGKV------------------------------------------PMGVGEYSKIKRCIIDKNARIGKNVILTN**

15 Pp3c11_5720/1-539 99.8% 63.3%  **----KNGKI------------------------------------------PLGVGENSRISNCIIDKNARVGKNVIIAN**

16 Pp3c11_8270/1-524 98.7% 47.0%  **----ATGGI------------------------------------------PMGIGKNSVIKRAIVDKNARIGENVQIVN**

17 Pp3c1_3040/1-532 99.8% 64.5%  **----KAGKI------------------------------------------PLGVGENSRISNCIIDKNARIGKNVVIAN**

18 Pp3c1_31540/1-543 100.0% 59.3%  **----EEGKV------------------------------------------PLGIGTNTKIRNCIIDKNARIGNNVVIAN**

19 Pp3c14_12850/1-541 100.0% 59.9%  **----AEGKV------------------------------------------PLGVGENTKLRNCIVDKNARIGSNVVITN**

20 Pp3c17_13940/1-555 100.0% 58.7%  **----AEGKV------------------------------------------PLGVGENSKLRNCIVDKNARIGKDVVIAN**

21 Pp3c19_8410/1-532 100.0% 65.6%  **----AAGKV------------------------------------------PMGIGKNTTIKNCIIDKNAKIGKNVVIAN**

22 Pp3c2_35010/1-524 98.7% 46.6%  **----ATGGI------------------------------------------PMGIGKNSVVKRAIIDKNARIGENVKIVN**

23 Pp3c7_20320/1-526 98.7% 46.4%  **----ASGGI------------------------------------------PMGIGRNSVVKRAIIDKNARIGENVKIIN**

24 Pp3c7_20690/1-533 98.7% 45.8%  **----SSGGI------------------------------------------PMGIGRNSIVKRAIVDKNARIGENVQIVN**

25 Pp3c7_22280/1-536 99.8% 63.5%  **----RNGKI------------------------------------------PLGVGENSRISNCIIDKNARIGKNVVIAN**

26 Pp3s127_20/1-546 100.0% 58.2%  **----EEGKV------------------------------------------PLGIGANTKLRNCIVDKNARIGSNVVIAN**

27 Sacu_v1.1_s0067.g016264/1-532 100.0% 65.4%  **----ALGKV------------------------------------------PIGVGENSKIRNCIIDKNARIGKNVVLEN**

28 Sacu_v1.1_s0095.g019244/1-550 100.0% 64.0%  **----AEGKV------------------------------------------PVGVGENTIIKNCIIDQNVRIGRNVVITN**

29 Sacu_v1.1_s0157.g023711/1-526 100.0% 65.4%  **----AQGRV------------------------------------------PVGVGENSKIRNCIVDKNARIGKNVVLTN**

30 Sacu_v1.1_s0001.g000380/1-536 98.7% 45.8%  **----ANGGI------------------------------------------PVGIGRNCHIKRAIIDKNARIGENVKIIN**

31 Sacu_v1.1_s0076.g017538/1-573 98.7% 43.5%  **----ASGGV------------------------------------------PVGIGCNTHIKRAIIDKNAHIGDNVKIIN**

32 Azfi_s0120.g046768/1-488 100.0% 71.5%  **----AQGKV------------------------------------------PVGVGENSKIRNCIIDKNARIGKNVTLTN**

33 Azfi_s0003.g008048/1-504 98.7% 48.4%  **----SNGGI------------------------------------------AVGIGRNSHIKRAIVDKNARIGDNVKIIN**

34 Azfi_s0106.g045016/1-528 98.7% 46.6%  **----ANGGV------------------------------------------PVGIGCNTHIRRAIIDKNAHIGDNVKIIN**

35 Azfi_s0012.g012923/1-564 98.7% 43.5%  **----ASGGV------------------------------------------PVGIGRNTHIKRAIIDKNAHIGDNVKIIN**

36 CHBRA475g00200/1-614 99.8% 51.2%  **----ASGRV------------------------------------------PIGVGENTVLRNCIIDKNARIGRNCRITN**

37 AaBONN_Sc2ySwM_340.122.1/1-513 98.7% 48.0%  **----AAGGI------------------------------------------PIGIGRNSIIKRAIIDKNARIGENVKIVN**

38 Anthoceros/1-550 100.0% 66.0%  **----AEGKV------------------------------------------PIGIGENSKISNAIIDKNARIGRDVVIAN**

cov pid **881**  **. 9 . ]** **916**

1 Selmo_138695/1-447 100.0% 100.0%  **KDNVQEA-EKPSEGYYIRSGITVILKNATIADGTVI**

2 Selmo_149205/1-533 99.3% 63.2%  **TDGVQEA-ERPSEGIYIRSGITVVVKNSILKDGTVI**

3 Selmo_169778/1-463 100.0% 95.2%  **KDNVQEA-EKPSEGYYIRSGITVILKNATIADGTVI**

4 AT1G27680/1-518 100.0% 64.7%  **ADGVEEG-DRPEEGFHIRSGITVVLKNATIRDGLHI**

5 Bradi1g09537/1-504 100.0% 63.5%  **SEGVQEA-DRTSEGFHIRSGITVVLKNSVIADGLVI**

6 Bradi1g53500/1-512 100.0% 63.5%  **SENVQEA-DRPSEGYYIRSGITVVLKNAVIPDDTII**

7 Bradi2g14970/1-522 99.6% 54.2%  **SEGVEEA-DRAEEGYYIRSGIVVILKNATIKDGTVV**

8 Cre07.g331300/1-719 98.7% 23.6%  **RAGVWEAMDRVGVGLCVREGVPIVTKSAVLYEGTEI**

9 Cre13.g567950/1-512 99.6% 53.9%  **KEGVTEG-TREAEGIYIRSGIVVIDKGALVPDNTTI**

10 Cre16.g683450/1-560 99.3% 33.2%  **KEGVYESADRGVQGMYVRDGIIVLAREAVVPDGTIM**

11 Mapoly0023s0119/1-534 100.0% 66.7%  **ADNVQEA-ERASQGYYIRSGIVVILKNSTIAPGTVI**

12 Mapoly0033s0108/1-520 98.7% 47.7%  **RDDVQEA-ARETDGYFIKSGIVTIIKDAVIPNDTVI**

13 Mapoly0101s0020/1-543 100.0% 67.6%  **KDNVQEA-ERPSEGFYIRSGITVVLKNSTIKDGMVI**

14 Mapoly0132s0055/1-569 98.9% 47.0%  **TDGVREA-EKPSDGIYIRSGIIIVSENALIKDGMVI**

15 Pp3c11_5720/1-539 99.8% 63.3%  **TDNVQES-ARPELGFYIKTGVTVIEKNGIIRDGTVI**

16 Pp3c11_8270/1-524 98.7% 47.0%  **KDGVQEA-ARETDGYFIKSGIVTIIKDAIIPHGTII**

17 Pp3c1_3040/1-532 99.8% 64.5%  **TDNVQEA-SRPEEGFYIRTGVTVIEKNGIVKDGTVI**

18 Pp3c1_31540/1-543 100.0% 59.3%  **TDNVFEA-ARPSEGFYIRSGITVICKNAVIKHGTVI**

19 Pp3c14_12850/1-541 100.0% 59.9%  **ADNVFEA-ARPNEGFYIRSGIVVVCKNAVIKHGTVI**

20 Pp3c17_13940/1-555 100.0% 58.7%  **TDNVLEA-ERQSEGFYIRSGIVVVYKNAVIKHGTVI**

21 Pp3c19_8410/1-532 100.0% 65.6%  **TDTVFEA-DRAKEGFYIRSGIVVIAKNATIKDNTVI**

22 Pp3c2_35010/1-524 98.7% 46.6%  **KDSVQEA-ARETDGYFIKSGIVTIIKDAIIPHGSII**

23 Pp3c7_20320/1-526 98.7% 46.4%  **VGGVEEA-ARETDGYFIKSGIVTIIKDAIIPHGTVI**

24 Pp3c7_20690/1-533 98.7% 45.8%  **VDNVREA-EREADGFFIRSGLVTIFKDAIIPDGTII**

25 Pp3c7_22280/1-536 99.8% 63.5%  **TDNVQEA-TRPELGFYIKTGVTVIEKNGIIKDGTVI**

26 Pp3s127_20/1-546 100.0% 58.2%  **TDNVFEA-ARPDEGFYIRSGITVICKNAVIQNGTVI**

27 Sacu_v1.1_s0067.g016264/1-532 100.0% 65.4%  **TEGVEES-DRSEEGFYIRSGITIILKNSTIKDGTII**

28 Sacu_v1.1_s0095.g019244/1-550 100.0% 64.0%  **KEGVEEA-ERPAEGFYIRSGITVLVKNSIIKDGTVI**

29 Sacu_v1.1_s0157.g023711/1-526 100.0% 65.4%  **SENIEEC-DRTEDGFYIRSGIIVVLKNSTIADGTVI**

30 Sacu_v1.1_s0001.g000380/1-536 98.7% 45.8%  **RDDVQEA-ARETDGYFIKSGIVTVIKDAVIPSGIVI**

31 Sacu_v1.1_s0076.g017538/1-573 98.7% 43.5%  **RDDVQEA-ARESEGYFIKSGIVTVIKDAVIPSGMII**

32 Azfi_s0120.g046768/1-488 100.0% 71.5%  **SENVEDS-DRTDEGFYIRSGITVIPKNSTIKDGTVI**

33 Azfi_s0003.g008048/1-504 98.7% 48.4%  **RDNVQEA-ARETDGYFIKSGIVTVIKDAVIPAGIII**

34 Azfi_s0106.g045016/1-528 98.7% 46.6%  **RDDVQEA-AREIEGYFIKSGIVTVIKDAVIPSGMII**

35 Azfi_s0012.g012923/1-564 98.7% 43.5%  **RNEVQEA-ARESEGYFIKSGIVTVIKDAVIPSGMII**

36 CHBRA475g00200/1-614 99.8% 51.2%  **EKGVKEA-EMTAQGYYIRDGIVVILKNASIYHGTVI**

37 AaBONN_Sc2ySwM_340.122.1/1-513 98.7% 48.0%  **KDDVQEA-ARETDGYFIKGGIVTIIKDAIVPNNTII**

38 Anthoceros/1-550 100.0% 66.0%  **TDNVLEA-ERPSEGFYIRSGIAVILKNSVIKDGTVI**

>Selmo_138695/1-447

----------------------------------------------------------------APAIGK--

------------------------------------------------------------------------

--------------------------------------PRADPR-TVVSLILGGGAGT--RLFPLTNRRAKP

AVPIGGAYRLIDVPMSNCINSGINKIFILTQFNSASLNRHLARTYNFGNGVNF-GDGFVEVLAATQTPGEAG

MNWFQGTADAVRQFTWVFE-DTRSKEIENVLVLSGDHLYRMDYMEFIQKHQDTGADITIGCVP--MDDS--R

ASDFGLMKIDANGQ-ILYFSEKPKGAD-LK-AMQVD---------------------TTVL---GLTP----

---------------------------EEAIEKP----YIASMGIYVFKKDILLKLLRWRYPTANDFGSEIL

PASAKE--------------------------------------YNVQAYLFND-YWEDIG-TIKSFYEA--

NLALTCQP-PKFRFYDA-A---KPIYTSPRYLPPTKIEK-CRVLDSIVSHGCFLQ-----------------

--------------------------------------------ECSVTHSVIGIRSRVEAGAEIQDTMMLG

ADFYETEAEIASMV----------------------------------------------------------

------------AEGKV------------------------------------------PVGVGQNAKIRNC

ILDKNVRIGKNVVIANKDNVQEA-EKPSEGYYIRSGITVILKNATIADGTVI

>Selmo_149205/1-533

--------------------------------------MANAAISSSPMGSVSAGGRKDYLHACKSASSS--

-----------------LAKRDLFGAAL---------HLKKIP------------EKSKPRIAKCQAAQKGG

NAALSSAVLVDYPPREAKQVELPFSV---------FETPRVDPK-SVVSIILGGGVGT--RLFPLTKQRAKP

AVPIGGGYRLIDVPMSNCINSGINRVFVLTQFNSASLNRHLARTYNFINA----GEGFVEVLAATQTPGESG

MNWFQGTADAVRQFTWLFE-DVRNKDVDYVLVLSGDHLYRMDYMDFVQKHKDSGADITISCVP--VDES--R

ASDFGLVKTDARGR-IISFSEKPKGMD-LK-AMQVD---------------------TTAL---GLSR----

---------------------------EEAKKMP----YIASMGIYVFRKDVLLKLLRWRYPTSNDFGSEII

PAAANE--------------------------------------YNVQAYLFND-YWEDIG-TIKSFFDA--

NLALTAQP-PKFSFYDA-S---NPIFTSPRFLPPTKMEK-CRIIDSIVSHGCFLK-----------------

--------------------------------------------SCSVEHSLIGVRSRLESGVELKDTIIMG

ADSYETEAEIAALR----------------------------------------------------------

------------AQGKV------------------------------------------PLGVGEHTTMRNC

LVDKNARIGSHVIITNTDGVQEA-ERPSEGIYIRSGITVVVKNSILKDGTVI

>Selmo_169778/1-463

-------------------------------------------------------MQGFHRRGCLGRYCQ--

---------------------GNRGIGK--------------------------------------------

--------------------------------------PRADPR-TVVSLILGGGAGT--RLFPLTNRRAKP

AVPIGGAYRLIDVPMSNCINSGINKIFILTQFNSASLNRHLARTYNFGNGVNF-GDGFVEVLAATQTPGEAG

MNWFQGTADAVRQFTWVFE-DTRSKEIENVLVLSGDHLYRMDYMEFIQKHQDTGADITIGCVP--MDDS--R

ASDFGLMKIDANGQ-ILYFSEKPKGAD-LK-AMQVD---------------------TTVL---GLTP----

---------------------------EEAIEKP----YIASMGIYVFKKDILLKLLRWRYPTANDFGSEIL

PASAKE--------------------------------------YNVQAYLFND-YWEDIG-TIKSFYEA--

NLALTCQP-PKFRFYDA-A---KPIYTSPRYLPPTKIEK-CRVLDSIVSHGCFLQ-----------------

--------------------------------------------ECSVTHSVIGIRSRVEAGAEIQDTMMLG

ADFYETEAEIASMV----------------------------------------------------------

------------AEGKV------------------------------------------PVGVGQNAKIRNC

ILDKNVRIGKNVVIANKDNVQEA-EKPSEGYYIRSGITVILKNATIADGTVI

>AT1G27680/1-518

------------------------------------------MESCFPAMKLNQCTFGLNNEIVSERVSA--

-----------------FWGTQVVKPNH---------LRTTKL---------------------RSAPQKKI

QTNLIRSVLTPFVDQE---SHEPLLR-----------TQNADPK-NVASIILGGGAGT--RLFPLTSKRAKP

AVPIGGCYRLIDIPMSNCINSGIRKIFILTQFNSFSLNRHLSRTYNFGNGVNF-GDGFVEVLAATQTSGDAG

KKWFQGTADAVRQFIWVFE-DAKTKNVEHVLILSGDHLYRMDYMNFVQKHIESNADITVSCLP--MDES--R

ASDFGLLKIDQSGK-IIQFSEKPKGDD-LK-AMQVD---------------------TSIL---GLPP----

---------------------------KEAAESP----YIASMGVYVFRKEVLLKLLRSSYPTSNDFGSEII

PLAVGE--------------------------------------HNVQAFLFND-YWEDIG-TIGSFFDA--

NLALTEQP-PKFQFYDQ-K---TPFFTSPRFLPPTKVDK-CRILDSIVSHGCFLR-----------------

--------------------------------------------ECSVQHSIVGIRSRLESGVELQDTMMMG

ADFYQTEAEIASLL----------------------------------------------------------

------------AEGKV------------------------------------------PVGVGQNTKIKNC

IIDKNAKIGKNVVIANADGVEEG-DRPEEGFHIRSGITVVLKNATIRDGLHI

>Bradi1g09537/1-504

----------------------------------------MDLRVAAPASAAAAARRGVPGGGVVRLRAL--

----------------------------------------------------------------AAGGRQRR

LSVRVSVATTEAATAAVGASEDADT-------------ETRNAR-TVVAVILGGGAGT--RLFPLTKRRAKP

AVPIGGAYRLIDVPMSNCINSGINKVYVLTQFNSASLNRHLSRAYNFSNGVGF-GDGFVEVLAATQRPGLEG

KRWFQGTADAVRQFDWLFD-DAKAKDIEDVIILSGDHLYRMDYMDFVQSHRQRDAGISICCLP--IDDS--R

ASDFGLMKIDDTGR-VISFSEKPKGDD-LK-AMQVD---------------------TTVL---GLSK----

---------------------------EEAEEKP----YIASMGVYIFKKEILLNLLRWRFPTANDFGSEII

PAAAKE--------------------------------------INVKAYLFND-YWEDIG-TIKSFFEA--

NLALAEQP-PRFSFYDA-S---KPMYTSRRNLPPSMISS-SKITDSIISHGCFLD-----------------

--------------------------------------------NCRVEHSVVGVRSRVGSNVHLKDTVMLG

ADFYETDVERSDQL----------------------------------------------------------

------------AEGKV------------------------------------------PIGIGENTTIQNC

IIDKNARIGKNVTIANSEGVQEA-DRTSEGFHIRSGITVVLKNSVIADGLVI

>Bradi1g53500/1-512

-----------------------------------------MATSTGPMAPHAACPFSPVVLRRAPRLVR--

-----------------LAAPRRAAAAT---------TSCVLT-----------------------DAPQGI

KMEPSGAPSPESSTVVAPR-------------------RDVGPD-TVASIILGGGAGT--RLFPLTRTRAKP

AVPVGGCYRLIDIPMSNCINSKINKIYVLTQFNSQSLNRHIARTYNFGEGVGF-GNGFVEVLAATQTAGESG

KRWFQGTADAVRQFLWLFE-DARLKRIENILILSGDHLYRMDYMDFVQKHVDSGADISVACVP--MDES--R

ASDFGLMKTDRNGR-ITDFLEKPKGES-LK-SMVVD---------------------MEIF---GLSP----

---------------------------EVANVYN----YLASMGIYVFKTDVLLRLLRGHYPTANDFGSEVI

PMAAKD--------------------------------------YNVQAYLFDG-YWEDIG-TIKSFFEA--

NLALTDQS-PNFHFYEP-V---KPIFTSPRFLPPTKVED-CKVLNSIVSHGCFLT-----------------

--------------------------------------------ECSVEHSVIGIRSRLQPGVQLKDTMMMG

ADYYQTEAERFSEL----------------------------------------------------------

------------SDGKV------------------------------------------PVGVGENTKIRNC

IIDKNARIGKNVVIMNSENVQEA-DRPSEGYYIRSGITVVLKNAVIPDDTII

>Bradi2g14970/1-522

----------------------------------MSRMQFSSVLPLEGKACVSPLRRNVESSGSERIRIG--

-------------------------DSS---------TIKHER------------------ALRRMCFGARG

AASGAQCVLTSDASPDTLVVRTSFRR------------NYADPN-EVAAVILGGGTGT--QLFPLTSTRATP

AVPIGGCYRLIDIPMSNCFNSGINKIFVMTQFNSASLNRHIHRTY-LGGGINF-TDGSVEVLAATQMPGEA-

AGWFRGTADAVRKFIWVLEDYYKHKSIEHILILSGDQLYRMDYMELVQKHVDDNADITLSCAP--VGES--R

ASEYGLVKFDSSGR-VIQFSEKPKGVD-LE-AMKVD---------------------TSFL---NFAI----

---------------------------DDPAKFP----YIASMGVYVFKRDVLLNLLKSRYAELHDFGSEIL

PRALHE--------------------------------------HNVQAYVFTD-YWEDIG-TIRSFFDA--

NMALCEQP-PKFEFYDP-K---TPFFTSPRYLPPTKSDK-CRIKEAIISHGCFLR-----------------

--------------------------------------------ECTIEHSIVGVRSRLNSGCELKNAMMMG

ADLYETEDEISRLL----------------------------------------------------------

------------SEGKV------------------------------------------PIGVGENAKISNC

IIDMNARIGRDVIIANSEGVEEA-DRAEEGYYIRSGIVVILKNATIKDGTVV

>Cre07.g331300/1-719

-----------------------------MLLRHSAPGRGNAADRHRCTRLPTPPHAGTVAAGTGASAAR--

-----------------RSRGALRVLAF---------REQETRQQDKNDDQSKTHVKEKAGSAAAAAAAAAG

RRPQTRSHFPPVGRGGWEAADVSGRR------EDEGHGPPRISS-STLAVILGGGESD-RRLFPLTEKRALP

AVPVGGRYRLIDVPISNCLHACINKMFVLTQYNSQSLNKYINRAYGNREGVPNGGDGFVEVLATTQYPA--G

ARWSEGNADAVRMMAWLLD-QPRLRRIEDVLILPADQLYHTDLEALMEWHRGNEACLTVVAHG--VEGG--D

TEHVGLLKVRPSTRELLDYVEKPRTAR-EREPFRVP---------------------AEHV---G-------

---------------------------KLTNGRP----FLASCGIYVFDKHALLDVLS-AHPRAHDFGRGVI

PHAFDMAARSLKRRHQQARQRQERQERAAAAGVQLRPGYEYVHPYKVLTWTMAGRYWADVGNSIRTYKEATQ

HVLLGRGPGDAETPFDT-AYHHELIITGSALLPPAQLSPGCRLVRSALSPGCRVG-----------------

-------------------------------------------PGAVVENSVLGPRAVVGAGAVVRNSVVMG

ADYYDEDLRKGALRRRYGSGAAAGAGTGGTGSSHDSMEGPSLPPAPTPTPTTTTSSGGGGGSSSSSSGGGSS

GGGSSSSAHGRGSAGAVLLTAAQRAAGVYSMDPAEMERRGGGGGGGGGSETETDDPSVPPLGIGAGAVVEGA

LVDKNARIGTGCVVANRAGVWEAMDRVGVGLCVREGVPIVTKSAVLYEGTEI

>Cre13.g567950/1-512

------------------------------------------MATLQAKSRAAASSARSEKAVPASFSSGS-

-----------------FRGQAVARPAA---------TRAVR----------------------SVAARSRR

AQAVKAIIEAPL------LRYEP--------------ATKARTS-SVLSIILGGGAGT--RLFPLTKSRAKP

AVPIGGAYRLIDVPMSNCINSGISKIYILTQFNSTSLNRHLGRAYNMGSGVRFGGDGFVEVLAATQTPTD--

KEWFQGTADAVRQYSWLLE-DTKNRAIEDVLILSGDHLYRMDYMKFVNYHRETNADITIGCIA--YGSD--R

AKEFGLMKIDEKRR-VTSFAEKPKTQEALD-AMKVD---------------------TTVL---GLTP----

---------------------------EEAAEKP----YIASMGIYVFKKSVLLQLLNDSYAKANDFGGEII

PSAAKD--------------------------------------HNVVAYPFYG-YWEDIG-TIKSFFEE--

NLKLCRHP-ATFEFYDP-Q---SPIYTSPRVLPPATVRN-CKVTDAIIAQGSFVS-----------------

--------------------------------------------DCTINNAVIGIRSIIGQNCTIQDALVMG

ADYYESDDQRATLL----------------------------------------------------------

------------KKGGV------------------------------------------PVGIGANSVITNA

IIDKNARVGKNVKIVNKEGVTEG-TREAEGIYIRSGIVVIDKGALVPDNTTI

>Cre16.g683450/1-560

------------------------------------------------------------MKQLQQFQER--

-----------------WSGDMGGASGS---------SGLGSG---------------------GAATPGTG

LGEHSGSFGDPELSRS---------------------SLSIWPRGNLTAVVLGGGESDSRRLFPLTQYRTLP

AVPFGGAYRIIDLLMSNMLNSGINKIHILTAFNSYSLNRHLQRTYDMSGGVPYGGDGYIEVVANSMSPDS--

QNWVTGTAGCVRQFMSYFDSNSKNRFIEDIMILPGDHVYSADYTPIIAYHRSTGADLTIVCRP--VSGE--Q

ACRLGVVKLDAQNR-IKTFSEKPSASELPELAMSDDEMRPFMMPTEEALRQQARRRMQTTLASRGSGPLGRL

SAGAAKGGARMGGGGGGRVVLDTMTSVDETETRPGTTGYVGSCGIYIFKRSVLSEALKRHF-KMQDFGRQII

PELIREG-------------------------------------VKVHAYRLPG-YWADVGGSVGDFYAA--

NMSLLSDP-PSISFNAPIN---SPFFKFPLTIPASQMMH-SRVSGALVSAGCIVN-----------------

--------------------------------------------RSDIRNSVIGSRSIIGPNVTIEDSVVFG

ASHYDHEKPLPRPL----------------------------------------------------------

------------GPTFP------------------------------------------PMGIGEGSIVRNA

ILDLNVRVGKNVQLVNKEGVYESADRGVQGMYVRDGIIVLAREAVVPDGTIM

>Mapoly0023s0119/1-534

------------------------------------MAAAAVTSVGWHCAVKNVAVASTSGVAERSAEAS--

-----------------FGRSAFRGDAV---------SGFLKQ--------------GNRVLDSSRSGSSRQ

QSVFKRAPISSVITEVAKKTVQPVDE-----------SPRADPK-TVVSLILGGGAGT--RLFPLTKRRAKP

AVPIGGGYRLIDVPMSNCINSGINKVFILTQFNSASLNRHLARTYNFGNGINF-GDGFVEVLAATQTPGEGG

KEWFQGTADAVRQYLWLFE-DAKNKNLEDVLILSGDHLYRMDYMDFVEKHRNSGADITISCVP--MDDS--R

ASDYGLMKIDDTGR-VLYFSEKPKGAD-LK-AMEVD---------------------TSVL---GLSA----

---------------------------EDAKSKP----YIASMGIYVFKKEILQKLLRWRYPTANDFGSEII

PASAKE--------------------------------------FNVQAYLFDD-YWEDIG-TIKSFFDA--

NLGLTAQP-PKFSFYDA-V---KPIFTSPRYLPPSKIEK-CRVEDSIISHGCFLR-----------------

--------------------------------------------DCSIKHSIVGIRSQMASGSALKDTMMLG

ADYYETDAERAALI----------------------------------------------------------

------------AEGKV------------------------------------------PIGVGANCKISNC

IIDKNARIGSNVVIANADNVQEA-ERASQGYYIRSGIVVILKNSTIAPGTVI

>Mapoly0033s0108/1-520

-------------------------------------MAGVATAAVSCMSVHSVLAETKLGRGTKSVTAS--

-----------------SSDLRSASSSL---------RGARLS--------------------SQCTQRSHA

RMASKTLVAPRAVSDS--QNGESCL-------------TPEAGR-SVLGIILGGGAGT--RLYPLTKKRAKP

AVPLGANYRLIDIPVSNCINSNVQKIYVLTQFNSASLNRHLSRAYGSNMG-GYKNEGFVEVLAAQQSPEN--

PNWFQGTADAVRQYLWLFE----EQNVLEFLVLAGDHLYRMDYQNFIQAHRDTNADITVAALP--MDEA--R

AEAFGLMKINEKGR-IIEFAEKPKGDE-LK-AMAVD---------------------TTVL---GLDE----

---------------------------ARAKEMP----YIASMGIYVVSKDAMIKLLRDDFPEANDFGSEVI

PGATKLG-------------------------------------MSVQAYLYDG-YWEDIG-TIEAFYNA--

NLGLTKKPVPDFSFYDR-T---SPIYTQARFLPPSKMLD-ADVTDSVIGEGCVIK-----------------

--------------------------------------------NCKIYHSVVGLRSWIAEGAIVEDALLMG

ADYYETDEQRSELL----------------------------------------------------------

------------ATGGI------------------------------------------PMGIGRNSIIKRA

IIDKNARIGENVKIVNRDDVQEA-ARETDGYFIKSGIVTIIKDAVIPNDTVI

>Mapoly0101s0020/1-543

----------------------------------------MAVAASNVRSAVGLSQCSVSGSTAAAPVSS--

-----------------RAGSGLGRKAF---------CGESVT---------GSFGKGRVDFQVGESKSRRR

SGSLVQPVASSLLAEIAKEIAQAQAP---AQAPLTEFRPRVDPQ-TVASLILGGGAGT--RLFPLTRRRAKP

AVPIGGAYRLIDVPMSNCINSGINKVFILTQFNSASLNRHLARTYNFGNGVNF-GNGFVEVLAATQTPGESG

MNWFMGTADAVRQFTWLFE-DVKNKGVEHVLILSGDHLYRMDYMDFVQKHKDSGADITISCVP--MDDS--R

ASDYGLMKIDHKGQ-VLYFNEKPKGEQ-LK-SMQVD---------------------TTVL---GLSP----

---------------------------EEAKKMP----YIASMGIYVFKKEILLKLLRWRYPTANDFGSEII

PASAKE--------------------------------------FNVQAYLFND-YWEDIG-TIKSFFDA--

NLALTEQP-PKFKFYDV-A---KPIFTSPRYLPPTKVEK-CRIIDSIVSHGCFLR-----------------

--------------------------------------------DCSVEHSIVGIRSRIESGVELQDTMMMG

ADYYETDAEMASLL----------------------------------------------------------

------------ASGKV------------------------------------------PLGVGTNSKIRNC

IIDKNSRIGRNVIIMNKDNVQEA-ERPSEGFYIRSGITVVLKNSTIKDGMVI

>Mapoly0132s0055/1-569

-------------------------MAVATIVPFNLKLHSAQLNGTGPFRSSSNSLRNLFSSSSDRFFGRTD

QIWKLIKT---------CSATAYVSSTS---------KSCNCR------WTTSKSSNGSPVVAVAGKHNRRV

NKAATTSLLYDIAKE---IAAQASLSTDSEV------RSKVDPK-SVACMILGGEAGS--RLFPLTKRRAKS

AVPMGGAYRLIDIQMSNCINSGINKVYVLTQFNSASLNRHISRTYNSVTC----RDGFVEVLAATQTLGD--

KRWFMGTADAVRRFSWIFD-NVRSEAVEHVLVLSGDHLYRMNYMDLVQSHHNSGADITVSCVPLSMDDSSCQ

VSAGGLLRLDHKGR-VLSIHDKAMADNGYGLSMLLE---------------------SAAAAGSGLPE----

---------------------------GAAKNSP----FVASMGLYVFKKDVLIKLLKWIYPNSNDFASEII

PAAAKD--------------------------------------FYVQAYLFRN-YWQDVG-SIESYFEA--

NLALTEES-PKFEFYDV-S---NPIYTSPRYLPPTTVDH-CRIVDSIVSHGCFLR-----------------

--------------------------------------------SCSVQHSIIGIRSRIETGVELKDVVMIG

ADDYETEEERSRLL----------------------------------------------------------

------------AIGKV------------------------------------------PMGVGEYSKIKRC

IIDKNARIGKNVILTNTDGVREA-EKPSDGIYIRSGIIIVSENALIKDGMVI

>Pp3c11_5720/1-539

----------------------------------MAATMACVSTSCCSITSSRDSYGRQGQQQQQGGQGA--

-----------------APASPPFRSGF---------SGEKLA------ASLKAQAWLPVAPLKQRKDSKGP

LSVSSVLMEPRPLEDTIVNTGEA---------------TRVDPR-TVLSIILGGGAGT--RLYPLTKRRAKP

AVPIGGAYRLIDVPMSNCINSGINKVFILTQFNSTSLNRHLARTYNFGK-INF-GDGFVEVLAATQTPGDKG

AEWFQGTADAVRQYLWLLE-DAKNKVVEDVVILSGDHLYRMDYMDFVQKHRDSGADITISCVP--MDDS--R

ASDYGLMKIDDEGR-VLYFSEKPKGND-LK-NMQVD---------------------TTVL---GLSP----

---------------------------EEAVEKP----YIASMGIYVFKKDVLMKLLRWRYPTANDFGSEII

PASAKE--------------------------------------FNVQAYLFND-YWEDIG-TIKSFFDA--

NLALTAQP-PQFSFYDA-A---KPIFTSPRYLPPTSIEQ-CMIKDSIVSHGCFLK-----------------

--------------------------------------------NCSVEHSIVGVRSRLEFGSVLKDTMMMG

ADYYETEDEVAAML----------------------------------------------------------

------------KNGKI------------------------------------------PLGVGENSRISNC

IIDKNARVGKNVIIANTDNVQES-ARPELGFYIKTGVTVIEKNGIIRDGTVI

>Pp3c11_8270/1-524

------------------------------------------MAATLASTAAAVTSARLSNGSTTPRGGG--

-----------------FAKRGSALVGS---------NSSSLR------------GARLAVSSSYSSASARA

QRPRKGVVSPRNVSDS-PVVGEACL-------------DPDASR-SVLGIILGGGAGS--RLYPLTKKRAKP

AVPLGANYRLIDIPVSNCINSNINKIYVLTQFNSASLNRHLSRAYASNMG-GYKNEGFVEVLAAQQSPEN--

PNWFQGTADAVRQYLWLFE----EAQVLEYVILAGDHLYRMDYQKFIQVHRETNADITVAALP--MDET--R

ATAFGLMKINDQGR-IIEFAEKPKGDE-LR-AMQVD---------------------TTVL---GLDA----

---------------------------ARAKEMP----YIASMGIYVVSKEAMISLLRNDFPEANDFGSEVI

PGATEMG-------------------------------------MKVQAYLYDG-YWEDIG-TIEAFYNA--

NLGITKKPVPDFSFYDR-S---APIYTQARFLPPSKMLD-ADVTDSVIGEGCVIK-----------------

--------------------------------------------NARIYHSVVGLRSWVAEGAVIEDALLMG

ADYYETDDQRNELL----------------------------------------------------------

------------ATGGI------------------------------------------PMGIGKNSVIKRA

IVDKNARIGENVQIVNKDGVQEA-ARETDGYFIKSGIVTIIKDAIIPHGTII

>Pp3c1_3040/1-532

----------------------------------MAAATACVSTGCCSITSAREGYGRQQQGSPSNAAAK--

---------------------SLSRSSF---------CGDKVA-------------ASLKAQAWLPATPKRQ

NAAMGPIQVSSVLAELDEALEDGTEE-----------KARVDPR-TVLSIILGGGAGT--RLYPLTKRRAKP

AVPIGGAYRLIDVPMSNCINSRINKVFILTQFNSTSLNRHLARTYNFGK-INF-GDGFVEVLAATQTPGDRG

ADWFQGTADAVRQYLWLFE-DAKNKVVEDVVILSGDHLYRMDYMDFVQKHRDSGADITISCVP--MDDS--R

ASDYGLMKIDDEGR-VLYFNEKPKGDA-LK-SMQVD---------------------TTVL---GLTP----

---------------------------EEAAAKP----YIASMGIYVFKKDVLLKLLRWRYPTANDFGSEII

PASAKE--------------------------------------FNVQAYLFND-YWEDIG-TIKSFFDA--

NLALTSQP-PQFSFYDA-T---KPIFTSPRYLPPTKIEQ-CQVVDSIISHGCILK-----------------

--------------------------------------------GCSVNHSIVGIRSRLQEGIVLKDTMMMG

ADFYQTEEEVAQLL----------------------------------------------------------

------------KAGKI------------------------------------------PLGVGENSRISNC

IIDKNARIGKNVVIANTDNVQEA-SRPEEGFYIRTGVTVIEKNGIVKDGTVI

>Pp3c1_31540/1-543

------------------------------------MAAAGMFASTSYCTLAGPSCYEPILDGDSSLRSG--

-----------FKGDKKLSSAGFLAPGG---------DGLSFK---------------SNGCSKAAAIRSKS

STLNVSAVLAERPMETLQGQATRERD-------TKVPKTQVNMT-KVFSIILGGGAGT--RLNPLTLRRAKP

AVPLGGAYRLIDVPMSNCINSGINKIYVLTQFNSTSLNRHLARTYNFGNGCNF-GDGYVEVLAAAQRPGFGG

DKWFEGTADAVRQYLWLLE-DAKNKDVEDVIILSGDHLYRMDYEDFVQKHKDSGADVTVSCVP--MDDS--R

ASDYGLMKIDGKGR-INYFNEKPKGDD-LQ-AMQVD---------------------TSVL---GLSS----

---------------------------EEAKKKP----YIASMGIYVFKKSVLTKLLRWRYPLSNDFGSEII

PQAAKE--------------------------------------FNVHAYLYND-YWEDIG-TIKSFFDA--

NLALTSPN-SKFSFYDA-A---KPTYTSARYLPPTKIEK-CIVKDSIVSHGCFLR-----------------

--------------------------------------------DCSVENSIVGIRSRLESGCDVKRAMIMG

ADYYETDPEAAALL----------------------------------------------------------

------------EEGKV------------------------------------------PLGIGTNTKIRNC

IIDKNARIGNNVVIANTDNVFEA-ARPSEGFYIRSGITVICKNAVIKHGTVI

>Pp3c14_12850/1-541

------------------------------------MAAAGMFASTSYCTVARPAHCEALSGDVPFRSGF--

-----------------RGEQKMSSAGFM--------CGGDCA------------ALKSNVSPKPTAARTKS

SGLDISAILAERPLEALIEKTTRERV-------KKTPKQPVNMT-KVFSIILGGGAGT--RLNPLTLRRAKP

AVPLGGGYRLIDVPMSNCINSGINKIYVLTQFNSTSLNRHLARTYNFGNGCNF-GDGYVEVLAAAQRPGSGG

DKWFEGTADAVRQYLWLLE-DAKNKDVEDVIILSGDHLYRMDYEDFVQKHKDSGADVTVSCVP--IDDS--R

ASDYGLMKIDGKGQ-IRHFSEKPKGDD-LH-AMQVD---------------------TTVL---GLSG----

---------------------------EEAKKKP----YIASMGVYVFKKSVLAKLLRWRYPLANDFGSEII

PQAAKE--------------------------------------FNVQAHLFNG-YWEDIG-TIKSFFDA--

NLALTAEN-PKFSFFDA-A---KPIYTSARYLPPTKIEK-CRVKDSIVSHGCFLR-----------------

--------------------------------------------ECSLENSVIGVRSRLESGCDVKRSMVMG

ADFYETDPEAAALL----------------------------------------------------------

------------AEGKV------------------------------------------PLGVGENTKLRNC

IVDKNARIGSNVVITNADNVFEA-ARPNEGFYIRSGIVVVCKNAVIKHGTVI

>Pp3c17_13940/1-555

------------------------------------MAATGTFASTRYTALARPARCEAVGYDATMRSGF--

-----------------KGDLKPASSGFLA-------GGGRLALVTSVGPKRTVARAKTIGLEVSAVLAERP

MGALTRQVTREMEKEMEREREKEKSR------ESMSSKEQVNMT-KVFSIILGGGAGT--RLQPLTLRRAKP

AVPLGGGYRLIDVPMSNCINSGINKIYVLTQFNSTSLNRHLANTYNFGNGCNF-GDGYVEVLAAAQRPGFGG

DRWFEGTADAVRQYMWLLE-DAKNKDVEDVVILSGDHLYRMDYQDFVQKHKDSGADVTVSCIP--MDDS--R

ASDFGLMKIDGKGR-INHFSEKPKGKD-LQ-SMQVD---------------------TTVL---GLSA----

---------------------------EEAQKKP----YIASMGIYVFKKSVLAKLLRWRYPLANDFGSEII

PKAAKE--------------------------------------FNVNAYLFND-YWEDIG-TIKSFFDA--

NLALAAEN-PNFSFYDA-E---KPIYTSARYLPPTKIEK-CRVKDSIVSHGCFLR-----------------

--------------------------------------------ECSVEDSVIGIRSRLEAGCDVKRAMVMG

ADSYETDPEAAALL----------------------------------------------------------

------------AEGKV------------------------------------------PLGVGENSKLRNC

IVDKNARIGKDVVIANTDNVLEA-ERQSEGFYIRSGIVVVYKNAVIKHGTVI

>Pp3c19_8410/1-532

--------------------------------MAGAAILRSAQSAIVGTVACSSSQQKHSVNQLAAPPCS--

-----------------FSVSSFLHAGI---------CSPLRN--------------------DARSSTWCR

RVSKPNVVESLFVETTEESVESPPAQ-----------KPSVQAR-SVVSLILGGGAGT--RLFPLTHRRAKP

AVPIGGGYRLIDVPMSNCINSGINKIFILTQFNSASLNRHLARTYTFGNGVNF-GDGFVEVLAATQTPGEAG

MNWFQGTADAVRQFTWLFE-DAKNKQVEHVLILSGDHLYRMDYMDFIQKHKDSGADITISCVP--MDES--R

ASDYGLMKIDDKGR-VLYFNEKPRGVD-LE-SMQVD---------------------TSVL---GLSP----

---------------------------EEAKKMP----YIASMGIYVFRKDILLKLLRWRYPTSNDFGSEII

PAAAKE--------------------------------------YNVQAYLFND-YWEDIG-TIKSFFDA--

NLALAAQP-PKFKFYDA-T---KPIFTSPRYLPPTKVEQ-CRIIHSIVSHGCFLR-----------------

--------------------------------------------DCKVEHSLIGLRSRLESGVEVKNTMMLG

ADFYETDEERVALI----------------------------------------------------------

------------AAGKV------------------------------------------PMGIGKNTTIKNC

IIDKNAKIGKNVVIANTDTVFEA-DRAKEGFYIRSGIVVIAKNATIKDNTVI

>Pp3c2_35010/1-524

---------------------------------------MAARTAAAAAAALAVPSTSVRLSSGRAPRDS--

-----------------FRSGGCAQVGS---------RSSSLR---------------GAGVVCPSNVTKIT

QRTRKVVVSPRNVSDS-PVVGETCL-------------DPDASR-SVLGIILGGGAGS--RLYPLTKKRAKP

AVPLGANYRLIDIPVSNCINSNINKIYVLTQFNSASLNRHLSRAYASNMG-GYKNEGFVEVLAAQQSPEN--

PNWFQGTADAVRQYLWLFE----EAQVLEYVILAGDHLYRMDYQHFIQIHRETNADITVAALP--MDEA--R

ATAFGLMKINDQGR-IIEFAEKPKGDE-LR-AMQVD---------------------TTVL---GLDA----

---------------------------ERAKEMP----YIASMGIYVVSKEAMISLLRNDFPEANDFGSEVI

PGATKMG-------------------------------------MKVQAYLYDG-YWEDIG-TIEAFYNA--

NLGITKKPVPDFSFYDR-S---SPIYTQARFLPPSKMLD-ADVTDSVIGEGCVIK-----------------

--------------------------------------------NARIFHSVVGLRSWVSEGAVIEDALLMG

ADYYETDKQRNELL----------------------------------------------------------

------------ATGGI------------------------------------------PMGIGKNSVVKRA

IIDKNARIGENVKIVNKDSVQEA-ARETDGYFIKSGIVTIIKDAIIPHGSII

>Pp3c7_20320/1-526

--------------------------------------MAAALAAVAAAAASTAAASPARLSVERTTRGA--

-----------------FVSGGNAQLGS---------SSSSLR--------------GTGVALSSSNVTLRR

QRHRKVVVSPRNVSDS-PVSVEACL-------------DPDASR-SVLGIILGGGAGS--RLYPLTKKRAKP

AVPLGANYRLIDIPVSNCINSNINKIYVLTQFNSASLNRHLSRAYASNMG-GYKNEGFVEVLAAQQSPEN--

PNWFQGTADAVRQYLWLFE----EAQVLEYVILAGDHLYRMDYQHFIQVHRETNADITVAALP--MDEA--R

ATAFGLMKINDQGR-IIEFAEKPKGDE-LK-AMQVD---------------------TTVL---GLDA----

---------------------------ERAKEMP----YIASMGIYVVSKEAMITLLRNEFPEANDFGSEVI

PGATKMG-------------------------------------MKVQAYLYDG-YWEDIG-TIEAFYNA--

NLGITKKPVPDFSFYDR-S---APIYTQARFLPPSKMLD-ADVTDSVIGEGCVIK-----------------

--------------------------------------------NAKIFHSVVGLRSWVAEGAVVEDALLMG

ADYYETDEQRNELL----------------------------------------------------------

------------ASGGI------------------------------------------PMGIGRNSVVKRA

IIDKNARIGENVKIINVGGVEEA-ARETDGYFIKSGIVTIIKDAIIPHGTVI

>Pp3c7_20690/1-533

---------------------------------MMAVAYTAAVMPTAIINSETASLNVTSKLSVRSRICS--

-----------------FAKCSYSSLGS---------CESFLG--------------GTRMADSSWNLFSSG

KQSSRKMLLSPRAISDYPVGGDASLY------------SVDASR-SVLGIILGGGAGT--RLYPLTKKRAKP

AVPIGANYRLIDIPVSNCINSNINKIYVLTQYNSASLNKHVSRAYATNLG-SYRNEGFVEILAAQQSHDN--

PNWFQGTADAVRQYLWLFE----EAQVMDYVILGGDHLYRMDYQKFIQVHRDTGADITVAALP--MDEA--R

ASAFGLMKIDGKGR-IYEFAEKPKEDE-LR-AMQVD---------------------TTVL---GLDS----

---------------------------ERAKLKS----YIASMGIYVVRKEAMVSLLRKDFPEANDFGSEVI

PGATKSG-------------------------------------MKVQAYLFDG-YWEDIG-TIEAFYNA--

NLGITKKPVPDFSFYDR-I---SPIYTQPRFLPPSKMMD-ADIRDSVIGEGCIIR-----------------

--------------------------------------------NAAIYHSSLGLRSWVSEGAVVEDTLLMG

ADYYETDQQRRELL----------------------------------------------------------

------------SSGGI------------------------------------------PMGIGRNSIVKRA

IVDKNARIGENVQIVNVDNVREA-EREADGFFIRSGLVTIFKDAIIPDGTII

>Pp3c7_22280/1-536

----------------------------------MAAGTACVYTSCSSIKCVQEGCGRTWQQLNVATIPS--

----------------------LPRSAF---------CGKKLA------SSLKAQAWLPVAPLKRRNDSKHA

LKVSSVLMEPRPLENTIVNVGTGEE-------------KRVDPR-TVLSIILGGGAGT--RLYPLTKRRAKP

AVPIGGGYRLIDVPMSNCINSGINKVFILTQFNSTSLNRHLARTYNFGK-INF-GDGFVEVLAATQTPGDRG

ADWFQGTADAVRQYLWLFE-DAKNKVVEDVVILSGDHLYRMDYMDFVQKHRDSGADITISCVP--MDDS--R

ASDYGLMKIDGEGR-VMSFSEKPKGDD-LK-KMQVD---------------------TTIL---GLSP----

---------------------------EEAAEKP----YIASMGIYVFKKDVLMKLLRWRYPTANDFGSEII

PASAKE--------------------------------------FNVQAYLFNS-YWEDIG-TIKSFFDA--

NLALTAQP-PQFSFYDA-A---KPIFTSPRYLPPTSIEQ-CMVKDSIISHGCFLK-----------------

--------------------------------------------KCSVEHSIVGVRSRLESGSVLKDTMMMG

ADFYDTEKEVADML----------------------------------------------------------

------------RNGKI------------------------------------------PLGVGENSRISNC

IIDKNARIGKNVVIANTDNVQEA-TRPELGFYIKTGVTVIEKNGIIKDGTVI

>Pp3s127_20/1-546

------------------------------------MAATGIIASTSYCTLTRPSRQEAFWGVDSCLRSG--

-----------------FKGRG--KLSS---------AGFLAP------GVNSLRLRGNTCPRPATAARSKS

NTLSVSAVLAERPIETLFQQEQITREREVKTP-----KSQVNMK-KVFSIILGGGAGT--RLNPLTLRRAKP

AVPLGGAYRLIDVPMSNCINSGINKIYVLTQFNSTSLNRHLSRTYNLGNGSSF-GDGYVEVLAAAQRPGFGG

DKWFGGTADAVRQYLWLLE-DAKNKDVEEVVILSGDHLYRMDYEDFVQKHKDSGADITVSCVP--MDDS--R

ASDYGLMKINDKGR-IHYFNEKPKGDE-LQ-SMQVD---------------------TTVL---GLSP----

---------------------------DEAKKKP----YIASMGIYVFKKSVLSKLLKWRYPLANDFGSEII

PQAAKE--------------------------------------FYVHAYLFND-YWEDIG-TIKSFFDA--

NLALTSPD-SKFSFYDA-A---KPTYTSARYLPPTKIER-CRVKDSIVSHGCFLR-----------------

--------------------------------------------DCSVENSVIGIRSRLESGCDVKRAMIMG

ADFYETDPEASALQ----------------------------------------------------------

------------EEGKV------------------------------------------PLGIGANTKLRNC

IVDKNARIGSNVVIANTDNVFEA-ARPDEGFYIRSGITVICKNAVIQNGTVI

>Sacu_v1.1_s0067.g016264/1-532

------------------------------------------MASTAADSLQFLSRAPFFSTSSSSSTAS--

----------------SFLGTSLLPPSR---------HGLPSH---------PVHHHFNSGCQKPPLHRRSC

LVSASSATVLQNVSQHVVQTSETQK-------------SRVDPR-TVVSIILGGGAGT--RLYPLTKQRAKP

AVPIGGAYRLIDVPMSNCINSGINKVFILTQFNSASLNRHLARTYNFGNGVNF-GDGFVEVLAATQRPGETG

MNWFQGTADAVRQFTWLLE-DPKNADIEHVVILSGDHLYRMDYMDFVQKHKDTEADITISCVP--IDDS--R

ASDYGLMKIDDNGR-VLYFSEKPKGDD-LK-AMQVD---------------------TSVL---GLDP----

---------------------------VAAKESP----YIASMGVYVFRKDILLKLLRWRFPTANDFGSEII

PASAKE--------------------------------------YNMRAYLFDG-YWEDIG-TIRSFYEA--

NLALTAQP-PKFSFYDA-T---KPIYTSPRYLPPTKVDS-CRVVTSIISHGCFLQ-----------------

--------------------------------------------DCSVEHSIVGIRSRIENGVELKDTVMVG

ADYYETDAERAALL----------------------------------------------------------

------------ALGKV------------------------------------------PIGVGENSKIRNC

IIDKNARIGKNVVLENTEGVEES-DRSEEGFYIRSGITIILKNSTIKDGTII

>Sacu_v1.1_s0095.g019244/1-550

-------------------------------MASSSSAPPTSSSSSSISRTLLLRSPSSIASSRSPFLSP--

-----------------HHSLPFPSPPS---------LRLPSP------LLPISSHECSDRLSHPPPPRAST

SGNSAILQLERRLHMLDPNQWQANNG--------SIIGPRADPK-SVVSVILGGGAGT--RLFPLTSRRAKP

AVPIGGAYRLIDVPMSNCINSGINKIYILTQFNSFSLNRHLARTYSTGNGVHF-GDGFVEVLAATQTPGETG

MNWFQGTADAVRQFTWLFE-DAKNKNIEHVLILSGDHLYRMDYMDFIQKHQDTGADITISCVP--MDES--R

ASDYGLMKIDTRGR-VLYFSEKPTGTD-LK-SMQVD---------------------TTVL---GLSS----

---------------------------QEARKKP----YIASMGIYAFKMDVLLKLLRWRYPTSNDFGSEII

PASTKE--------------------------------------YNVQAYLFNG-YWEDIG-TIRSFFDA--

NLALTAES-PNFEFYDA-A---KPIYTSPRYLPPTKIER-CRVIDSIISHGCFLR-----------------

--------------------------------------------DCSVNHCIIGVRSRIDSGCELEDVMMMG

ADVYETEVEMAALL----------------------------------------------------------

------------AEGKV------------------------------------------PVGVGENTIIKNC

IIDQNVRIGRNVVITNKEGVEEA-ERPAEGFYIRSGITVLVKNSIIKDGTVI

>Sacu_v1.1_s0157.g023711/1-526

------------------------------------------MAYAASVPSPLPFSSPSTTTPPTFSSSF--

-----------------YGSCLFPFHAY---------SVASFP----------------RTIRHRQHRDRSS

LCIRSSASTVSLLSDTYQNAQTSETD-----------KSRVDPR-NVASIILGGGAGT--RLFPLTRRRAKP

AVPIGGAYRLIDVPMSNCINSRINKIYILTQFNSASLNRHIARTYNLGNGVNL-GDSFVEVLAATQRPGEAG

MQWFQGTADAVRQFAWLFE-DTKNSDIEHVIILSGDHLYRMDYMDFVQKHKDNNADITISCVP--IDNS--R

ASDYGLMKIDSNGR-VLYFSEKPKGED-LK-AMEVD---------------------TTVL---GLSP----

---------------------------AQAEKTP----YIASMGVYVFKKDILLKLLRWRYPTANDFGSEII

PASAKE--------------------------------------CNVQAYLFDD-YWEDIG-TIRSFYEA--

NLALTAQP-AKFSFYDA-A---KPIYTSPRYLPPTKVDR-CKVVGSIISHGCFLR-----------------

--------------------------------------------ECTVEHSIVGIRSRIEDGVELKDTVMLG

ADYYETDAERAAIL----------------------------------------------------------

------------AQGRV------------------------------------------PVGVGENSKIRNC

IVDKNARIGKNVVLTNSENIEEC-DRTEDGFYIRSGIIVVLKNSTIADGTVI

>Sacu_v1.1_s0001.g000380/1-536

-------------------------------------MANAAAIASSSRSTTHPPHLQFSSLLCTTTPTH--

-----------------YRPRCSRLLSS---------SSSSFL----LSSTSPFLLHLHRGSASSSASNSCT

TTLAPFIVAPRAVSDS--PTSEACL-------------DPPARE-SVLSIILGGGAGT--RLYPLTKKRAKP

AVPLGANYRLIDIPVSNCINSDIRKIYVLTQFNSASLNRHLSRAYASNMG-GYKHEGFVEVLAAQQSPEN--

PNWFQGTADAVRQYLWLFE----EQQVMEYLILAGDHLYRMDYQRFIQVHRETDADITVAALP--MDEK--K

ATAFGLMKIDSEGR-IIEFAEKPKGEF-LK-AMKVD---------------------TTIL---GLDE----

---------------------------ERAKEQP----FIASMGIYVINKDAMLRLLRDEFPQANDFGSEVI

PGATSLG-------------------------------------MKVQAYLYDG-YWEDIG-TIEAFYNA--

NLGITKKPFPDFSFYDQ-S---SPIYTQARFLPPSKMVD-ADVSESVIGDGCIIK-----------------

--------------------------------------------NCKIHHSVIGLRSWISEGAIIEDALLMG

ADYYETDEQREIVT----------------------------------------------------------

------------ANGGI------------------------------------------PVGIGRNCHIKRA

IIDKNARIGENVKIINRDDVQEA-ARETDGYFIKSGIVTVIKDAVIPSGIVI

>Sacu_v1.1_s0076.g017538/1-573

---------------------------------------MAAASASAPCFRHHHRSFPSLPRHCPSSANL--

-----------------ASLHAFSSPRL---------FASPLL--------------------------HRR

RAPSFPSFSPRAVSDS--RNDEVCL-------------DPDAGD-SVLGIILGGGAGT--RLYPLTKKRAKP

AVPLGANYRLIDIPVSNCINSNIRMIYVLTQFNSASLNRHLSRAYSSNMG-GYKHEGFVEVLAAQQSPEN--

PNWFQGTADAVRQYLWLFE----EQQVMEFLVLAGDHLYRMDYQKFIQVHRETNADITVAALP--MDEK--R

ATGFGLMKIDSEGR-IVEFAEKPKGDF-LK-AMKVD---------------------TTIL---GLDE----

---------------------------ERAKELP----YIASMGIYVVSKDVMIKLLRDEFPQANDFGSEVI

PGATSLG-------------------------------------MKVQAYLYDG-YWEDIG-TIEAFYNA--

NLGITKKPFPDFSFYDR-S---APIYTQPRFLPPSKVID-ADVTDSVIGEGCLIKVQELITIEVADVDLTTV

KRKIKICWNSWTRFRGLFLLKLFFGELILLPVRYITARIFMVLQNCKIHHSVIGLRSWISEGAIIEDALLMG

ADYYETDEQRDAVI----------------------------------------------------------

------------ASGGV------------------------------------------PVGIGCNTHIKRA

IIDKNAHIGDNVKIINRDDVQEA-ARESEGYFIKSGIVTVIKDAVIPSGMII

>Azfi_s0120.g046768/1-488

--------------------------------------------MTHMINNIGTNVVPIPRGVPESESQG--

------------------------------------------------------------------------

-----VELEVRAAPESEGLTSAAVQK------------TPVDPR-SVLSIILGGGAGT--RLFPLTKQRAKP

AVPIGGAYRLIDVPMSNCINSGISKVFILTQFNSASLNRHLARTYNFGNGVNF-GDGFVEVLAATQRPGETG

MNWFQGTADAVRQFTWLLE-DPKNTDLEHVVILSGDHLYRMDYMDFVQKHKDSAADITISCVP--IDDS--R

ASDYGLMKIDSNGR-VLNFAEKPKGDE-LK-AMQVD---------------------TTVL---GLDP----

---------------------------EAAKESP----YIASMGVYVFKKDILLKLLRWRYPTANDFGSEII

PASAKE--------------------------------------YNVKAYLFKG-YWEDIG-TIRSFYEA--

NLALTAQP-PKFSFYDA-A---KPIYTSARYLPPTKVDS-CRILGSILSHGCFLQ-----------------

--------------------------------------------SCSVEHSIVGIRSRIENGVELKDTVMLG

ADFYETDAERASLL----------------------------------------------------------

------------AQGKV------------------------------------------PVGVGENSKIRNC

IIDKNARIGKNVTLTNSENVEDS-DRTDEGFYIRSGITVIPKNSTIKDGTVI

>Azfi_s0003.g008048/1-504

----------------------------------------MAGTTISPVSCFCNPRRSKLTSSFIPPRPS--

-------------------------SSS---------HSPSSS-------------------------SLYF

SSRRVFLVSPRAVSDS--RNDDACL-------------DPAAGE-SVLSIILGGGAGT--RLYPLTKKRAKP

AVPLGANYRLIDIPVSNCINSDIRKIYVLTQFNSASLNRHLSRAYASNMG-GYKREGFVEVLAAQQSPEN--

PNWFQGTADAVRQYLWLLE----EQQVMEFLILAGDHLYRMDYQKFIQVHRESNADITVAALP--MDEK--R

ATAFGLMKIDSEGR-IVEFAEKPKGEF-LK-AMKVD---------------------TTIL---GLDE----

---------------------------DRAKELP----FIASMGIYVISKDAMIKLLRDEFPQANDFGSEVI

PGATSLG-------------------------------------MRVQAYLFDG-YWEDIG-TIEAFYNA--

NLGITKKPFPDFSFYDR-S---SPIYTQARFLPPSKMVD-ADITESVIGDGCIIK-----------------

--------------------------------------------NCKIHHSVIGLRSWISEGAIIEDTLLMG

ADYYETDEQRQIVT----------------------------------------------------------

------------SNGGI------------------------------------------AVGIGRNSHIKRA

IVDKNARIGDNVKIINRDNVQEA-ARETDGYFIKSGIVTVIKDAVIPAGIII

>Azfi_s0106.g045016/1-528

-----------------------------------MAEASMAIFSASSSSCIANLNVRWQPSSPSSLSVT--

-----------------FIRSNNLASSN---------SISDIS-------------SLTARPSVGSSLHRQG

RPNHFQLVSPRAVSDS--RNDEVCL-------------DPDAGD-SVLGIILGGGAGT--RLYPLTKKRAKP

AVPLGANYRLIDIPVSNCINSNIRMIYVLTQFNSASLNRHLSRAYSSNMG-GYKHEGFVEVLAAQQSPEN--

PNWFQGTADAVRQYLWLFE----EQQVMEFLILAGDHLYRMDYQKFIQVHRETNADITVAALP--MDEE--R

ATAFGLMKIDSEGR-IVEFAEKPKGDF-LR-GMKVD---------------------TTIL---GLDE----

---------------------------ERAKELP----YIASMGIYVVNKDAMIKLLKDDFPNANDFGSEVI

PGATALG-------------------------------------MKVQAYLYDG-YWEDIG-TIEAFYNA--

NLGITKKP-PDFSFYDR-S---APIYTQPRFLPPSKVVD-ADVTDSVIGEGCLIK-----------------

--------------------------------------------NCKIHHSVIGLRSWISEGAIIEDALLMG

ADYYETDDQRSSVI----------------------------------------------------------

------------ANGGV------------------------------------------PVGIGCNTHIRRA

IIDKNAHIGDNVKIINRDDVQEA-AREIEGYFIKSGIVTVIKDAVIPSGMII

>Azfi_s0012.g012923/1-564

--------------------------MSMAAETPSALAMVAPYSSCSSSSDSCICRTLSMATTTTMMTRSTR

RYQRYNASSLSSSSLLSPITHATIRPCF---------HGSAPP------KSLASCKAFSDTSFLSMRSTNGY

SRRHINTISPRAVSDS--RNDEVCL-------------DPDAGD-SVLGIILGGGAGT--RLYPLTKKRAKP

AVPLGANYRLIDIPVSNCINSNIRMIYVLTQFNSASLNRHLSRAYSSNMG-GYKHEGFVEVLAAQQSPEN--

PNWFQGTADAVRQYLWLFE----EQQVMEFLILAGDHLYRMDYQKFIQVHRETNADITVAALP--MDEK--R

ATGFGLMKIDSEGR-IVEFAEKPKGDF-LR-AMKVD---------------------TTIL---GLDE----

---------------------------ERAKDLP----YIASMGIYVVSKDAMIKLLRDDFPQANDFGSEVI

PGATSIG-------------------------------------MKVQAYLYDG-YWEDIG-TIEAFYNA--

NLGVTKKPFPDFSFYDR-S---APIYTQPRFLPPSKVVD-ADVTDSVIGEGCLIK-----------------

--------------------------------------------NCKIHHSVIGLRSWISEGAIIEDALLMG

ADYYETKEQREAVI----------------------------------------------------------

------------ASGGV------------------------------------------PVGIGRNTHIKRA

IIDKNAHIGDNVKIINRNEVQEA-ARESEGYFIKSGIVTVIKDAVIPSGMII

>CHBRA475g00200/1-614

MAAAMAHALPMPVVADGSTSFSLRPSGPVPTHAVTASAAMVKSAARAPSSAAGWSSALVPQSVSRASRDLIS

AREAFVAGGGSAEGAGFFRGQMFGAAGIPGAASFKSPSGEKLPLEMATVLCAAEPPARPGSDRGPPAARKGR

GKMEVNAVLADMAKRTTPEEELTHVI---------QLRERALPR-NVISVILGGGAGT--RLYPLTRHRAKP

AVPIGGAYRLIDVPMSNCINCGIKKIFILTQFNSTSLNRHLAKTYNSG-GITF-ADGFVEVLAATQTPGEGG

QEWFQGTADAVRQYLWLLEQDLSHRDVEDILILSGDHLYRMDYMDFVQRHKDTGADITISVLP--IDDS--R

ASDYGLMKTDDKGR-VVSFAEKPKGQE-LA-AMAVD---------------------TTIL---GLSA----

---------------------------AEAKEKP----YIASMGIYVFKKKTLIELLRSRYPHANDFGSEII

PSSAGE--------------------------------------FNVQAYLFND-YWEDIG-TMRSFFEA--

NLGLTRHP-ARFSFYDA-Q---KPIYTSPRNLPPSKIEK-CRVKDSIISHGCFLR-----------------

--------------------------------------------SCAVKHSIIGLRSRVEQGVDIADTMMMG

ADYYETDAERAALL----------------------------------------------------------

------------ASGRV------------------------------------------PIGVGENTVLRNC

IIDKNARIGRNCRITNEKGVKEA-EMTAQGYYIRDGIVVILKNASIYHGTVI

>AaBONN_Sc2ySwM_340.122.1/1-513

--------------------------------------MAAGGCFCNVAGKSCDTLRPVLRSSSRSLSST--

-----------------AAVGSFHALVA---------NIDSTP----------------------------W

RSSKLRNLHIRAVSDSQSASGDSCL-------------DPDAGR-SVLGIILGGGAGT--RLYPLTKKRAKP

AVPLGANYRLIDIPVSNCINSNIQKIYVLTQFNSASLNRHLSRAYASNMG-GYKNEGFVEVLAAQQSPEN--

PNWFQGTADAVRQYLWLFE----EQQVLEFLVLAGDHLYRMDYQKFIQVHRETNADITVAALP--MDEK--R

ATAFGLMKIDDQGR-IIEFAEKPKGDA-LS-AMKVD---------------------TTVL---GLDE----

---------------------------ERAKELP----YIASMGIYVVSKEAMIKLLRNDFPEANDFGSEVI

PGATRIG-------------------------------------MKVQAYLYDG-YWEDIG-TIEAFYNA--

NLGITKKPIPDFSFYDR-S---APIYTLARFLPPSKMLD-ADVTDSVIGEGCVIK-----------------

--------------------------------------------NCKIHHSVVGLRSWVGEGAVIEDALLMG

ADYYETDGQRQELL----------------------------------------------------------

------------AAGGI------------------------------------------PIGIGRNSIIKRA

IIDKNARIGENVKIVNKDDVQEA-ARETDGYFIKGGIVTIIKDAIVPNNTII

>Anthoceros/1-550

-------------------------------MAAAAAGGLATMSSTAPAACCSPSGSASSASSSSSSTSYSS

-----------------LPATRLGRAAF---------CGTPVS------GGFFSAALGGSRLCAGSGQPRRA

ERASKTVRATAVLADLVAKETVATSV----------DQSRVDPR-TVVSVILGGGAGT--RLFPLTRRRAKP

AVPIGGNYRLIDVPMSNCINSGINKVFILTQFNSASLNRHLARTYNFGNGVNF-GDGFVEVLAATQTPGEGG

MEWFQGTADAVRQYLWLFE-DAKNKQIENVLILSGDHLYRMDYMDFVQKHKDSGADITISCVP--MDDS--R

ASDFGLMKIDDKGQ-IVSFSEKPKGAD-LR-AMQVD---------------------TTVL---GLSA----

---------------------------EEAKKKP----YIASMGIYVFKKDILLKLLRWRYPLANDFGSEII

PASAKE--------------------------------------FNVQAYLFND-YWEDIG-TIKSFYDA--

NLALTAQP-PKFSFYDA-N---KPIFTSPRYLPPTKVDK-CRIQDSIVSHGCFLR-----------------

--------------------------------------------ECSVVHSIVGVRSRLENGVELKDTLMLG

ADVYETDVERAALL----------------------------------------------------------

------------AEGKV------------------------------------------PIGIGENSKISNA

IIDKNARIGRDVVIANTDNVLEA-ERPSEGFYIRSGIAVILKNSVIKDGTVI

**Alpha amylase, AMY3**

|  |
| --- |
| cov pid 1 [ . . . . : . . . 80  1 Cre08.g362450/1-706 100.0% 100.0% -----------MDRSL------LSRTSPRMQLGRPQQLPPPTVPLAQVPRLQR--------RCVV-GSRVCQPVVAVRPG  2 CHBRA206g00120/1-1039 99.2% 23.4% ---------MGNGSKRYGSNNLHFAAVCEKGSGGLVTLPHLGGRLRGVGGARSGGHSDDGGDHST-SPRAAASAASAADG  3 AagrBONN_Sc2ySwM_362.1200.1/1-412 58.1% 32.4% --------------------------------------------------------------------------------  4 Mapoly0024s0059/1-996 97.6% 25.0% ------------MEIFATMQATFTTGPPLNSTEVRKCRAPDAALHQSLLHLRK-------------SPKVCSVRHDD---  5 Pp3c21_19790/1-956 98.3% 26.4% -----------MDTILTRSLAGVSGHLLPIGRGEFQSTSPSIHLISSYHCAGS-----NNNNSTIKTPFLGERVLVSR--  6 Pp3c22_22380/1-950 98.7% 26.6% -------MDTVSARSLAGMSSNLLSSPARTWHGEFRLVSQQIPLISSYHVTGS--------NTNN-STSVRTSFFGERSP  7 Selmo_127605/1-400 56.7% 43.1% --------------------------------------------------------------------------------  8 Sacu_v1.1_s0008.g003998/1-993 98.6% 26.9% ------------MVSVVPMTFDAISGLPMFSKNKLDTIQPRNISSAGIHGSGS--------NSFS-GPSLRVETLSG-SG  9 Sacu_v1.1_s0102.g019864:1-1500/1-1500 98.6% 17.2% ------------MVSVALTQHDIISGLCSLSSNKVDSILQSRYACISTVAANL--------KPLR-APVVYIEGLAV-SG  10 Azfi_s0015.g013850/1-998 98.4% 25.7% MKPWDIDLLLMEKVSILPKQHDTFSCLCTIS---KSTIIQPRIASTGIHLSSS--------KSFS-GPLLCVETLAAVSG  11 Bradi2g48150/1-868 94.6% 28.2% ----------------MSAASWSIPAIPRAAPPARGGLPGDAFLVAARPGPGR--------RRAAPGRRLRLRG----GG  12 AT1G69830/1-887 97.0% 26.4% ---------MSTVPIESLLHHSYLRHNSKVNRGNRSFIPISLNLRSHFTSNKL--------LHSI-GKSVGVSSMNK-SP  cov pid 81 . 1 . . . . : . 160  1 Cre08.g362450/1-706 100.0% 100.0% R--------ASA------------------------------------------------------------------GR  2 CHBRA206g00120/1-1039 99.2% 23.4% LLIVLSALADTQPAGQSQIEWPGDDQVQTTLQKDYA---LTRRVKVRGKLWVKVALWEN-LHRIRISVECDVLHRALLHW  3 AagrBONN_Sc2ySwM_362.1200.1/1-412 58.1% 32.4% --------------------------------------------------------------------------------  4 Mapoly0024s0059/1-996 97.6% 25.0% ---------IFNSSGLSTPVKPFFGGKRKLYKWQRPSSELQKRENLPRLLVTASTAGIGSDNSSESSFGSNFP--DPVSL  5 Pp3c21_19790/1-956 98.3% 26.4% ---------NVNTTKALRRIWHGR---------------------------VFVSVLKG--ENNNGSRGFQIP-DDVESL  6 Pp3c22_22380/1-950 98.7% 26.6% N--------TSK---------------------------VAKGQRRSRPDHAVVSVLKG--DRITGSRDVQAP-DDVESL  7 Selmo_127605/1-400 56.7% 43.1% --------------------------------------------------------------------------------  8 Sacu_v1.1_s0008.g003998/1-993 98.6% 26.9% R--------YTQKLRLFRGI--KCSGNVVSRSGSTP---VEENDQGSSKAEQDAKVLVK--DFDANSIGQNLD--DIEGL  9 Sacu_v1.1_s0102.g019864:1-1500/1-1500 98.6% 17.2% R--------NPFQSGISRGI--RCS-NHVPSSGLTP---VEEGGKDGSQLEEAIQGMVK--DSRDGEADLNIDMNNVDSI  10 Azfi_s0015.g013850/1-998 98.4% 25.7% R--------HLPPSGVSR--WIRCSGNRFSRSGSSP---IEGGQDASQLEETIKRLVDE------GSIGQNIDLNDVEGL  11 Bradi2g48150/1-868 94.6% 28.2% V--------VVARAGAAEVPVTHPEESGVVFSEKFP---LRRCKTVQGKAWARVVAEPDGEGMCKIVIGCDVEGKWVLHW  12 AT1G69830/1-887 97.0% 26.4% V--------AIRATSSDTAVVETAQSDDVIFKEIFP---VQRIEKAEGKIYVRLKEVKE--KNWELSVGCSIPGKWILHW  cov pid 161 . . . 2 . . . . 240  1 Cre08.g362450/1-706 100.0% 100.0% GGRLVVSSVDMSNSPLSSMDAGEGLDIM----------------------------------------------------  2 CHBRA206g00120/1-1039 99.2% 23.4% GVTTREEAGRQWTQPPKKIRPPGTVKYKDYAARTPLRSCYGAVSDGGEEHGGGRVGVDLE--------------------  3 AagrBONN_Sc2ySwM_362.1200.1/1-412 58.1% 32.4% ------------MPPPSTPAPP----------------------------------------------------------  4 Mapoly0024s0059/1-996 97.6% 25.0% RAALKASQARVLEIENEKRDILEALRQSEAKVQEYAALMVQTTDEALSELEASKKLFKAELSKVLEEKSTLQKETLLAKQ  5 Pp3c21_19790/1-956 98.3% 26.4% KVALAAAEARADAAKKAEKQALEALTAMEGKSSDTVKTSRNMKQIKLKGGNDDADG------------------------  6 Pp3c22_22380/1-950 98.7% 26.6% KAALAAAEARTDAAKQAEKKALDALAAMKNKSRDMAQNDQNSQEVFEG--------------------------------  7 Selmo_127605/1-400 56.7% 43.1% --------------------------------------------------------------------------------  8 Sacu_v1.1_s0008.g003998/1-993 98.6% 26.9% KSALKAAHAKMLAAESEKADALRALAQAEARLREYATSAAETTESAVHEMEAAKESVSIELQNIMKEKLATESELVVARS  9 Sacu_v1.1_s0102.g019864:1-1500/1-1500 98.6% 17.2% KAALMAAHARIAAAENEKADALRSLEVAETRLEEYASTAVQVTESAVHEMQAAKESVNMELKNIMEQKLALESELVVVKK  10 Azfi_s0015.g013850/1-998 98.4% 25.7% KTILVAAHARIVAAESEKEDALKALAEAEAKLQEYTSTAVQATEEPVKKTKHSNKSEGVDLQSIIDQKLAVESELAVAKK  11 Bradi2g48150/1-868 94.6% 28.2% GVSYDGEQGREWDQPPSEVRPPGSVTIKDYAIETPLVGSPNSEGHMVHEVEIK---------------------------  12 AT1G69830/1-887 97.0% 26.4% GVSYVGDTGSEWDQPPEDMRPPGSIAIKDYAIETPLKKLSEGDSFFEVAIN-----------------------------  cov pid 241 : . . . . 3 . . 320  1 Cre08.g362450/1-706 100.0% 100.0% --FDNNSDAECTVVTVEGKDKAHLLMSLTGGFSSA---------------------------------------------  2 CHBRA206g00120/1-1039 99.2% 23.4% --IAEEGAPEVIAFVLKDEETGRWHDKAGGNFIINLADLLE---------------------QRHERKTPAAAAHAAGGL  3 AagrBONN_Sc2ySwM_362.1200.1/1-412 58.1% 32.4% --------------------------------------------------------------------------------  4 Mapoly0024s0059/1-996 97.6% 25.0% DAVNLAVKIEKIAESAIQEATQRFAEDLVLKDSAAETAAAEAAAGVEESIRLAASDAAALVVTEASTVMEEALAAASLAK  5 Pp3c21_19790/1-956 98.3% 26.4% --ISLAVQVEKISEAAIQKATARITEDATLKVAAAETAAAEAVLQLEERLQRAVDEAASAVAGETQVAIDEARAAAKVAK  6 Pp3c22_22380/1-950 98.7% 26.6% --ISLAVQVEKISESTIQKATLRITEDAELKIAAAETAAAEVILELEDQFRRAAEDAVQAASVEAQVTIDEARAAVSAAR  7 Selmo_127605/1-400 56.7% 43.1% --------------------------------------------------------------------------------  8 Sacu_v1.1_s0008.g003998/1-993 98.6% 26.9% DAIELAVSVEKVADTILREATAHLIEEAQLKIAAAKTSAAEAAANVEERIKSAVHDTANAMIRETKDAIEKSFAALEAAK  9 Sacu_v1.1_s0102.g019864:1-1500/1-1500 98.6% 17.2% DALELALSVDKVADSILGEATTHLAEEARLKVAAAKTSAAEAAANVEERIRSAILDTTDNLIKETRDAIEKSFAALEAAK  10 Azfi_s0015.g013850/1-998 98.4% 25.7% NAIELAVCVDKVADAIYEETTASLAEEAHLKIAEAKTSAAEAANSVEERVKSAVLDTANAMIRETRDAIEKSFSALEAAK  11 Bradi2g48150/1-868 94.6% 28.2% --FNQDTPIAIINFVLKEEETGAWFQHKGGDFRIPLSGSLEDGDPF------GAQQDTVHPGAKPEGSSAQPQETVPGDK  12 AT1G69830/1-887 97.0% 26.4% --LNLESSVAALNFVLKDEETGAWYQHKGRDFKVPLVDDVPDNGNL-----IGAKKGFGALGQLSNIPLKQDKSSAETDS  cov pid 321 . . : . . . . 4 400  1 Cre08.g362450/1-706 100.0% 100.0% -----------GLTVISASITSDDGRVLDVFRVQTADGKKV--PEEQFPSVREHILSVTATSS--RSSMPAIYGIVAAAE  2 CHBRA206g00120/1-1039 99.2% 23.4% RLLDGGAVGLLEETHDLENEVDIGNRLSAMVGLAEGGDVKVLLSTDLSGPVLLHWGLVKRGEEQSKWTVPAKRFLPSNST  3 AagrBONN_Sc2ySwM_362.1200.1/1-412 58.1% 32.4% --------------------------------------------------------------------------------  4 Mapoly0024s0059/1-996 97.6% 25.0% QQATKAQEALAKGMEIFEELSAAKLTTLSLQEKVSYLERELGISQGIVESLRLELKASQMRTE--AANARAAEA-EAAVQ  5 Pp3c21_19790/1-956 98.3% 26.4% AQAAKSEALLNEQVNVLNELAEIEAKMLVLEEALLAAGRQLQIANGETERVRIELDAVQSFIK--TATARAEAA-EKTII  6 Pp3c22_22380/1-950 98.7% 26.6% VQAEKSEAILNKQVKALNELAEAEAKVLMLEEALLDAGRKLQLANGETERIRIELDSAQRFIK--TATARAEAA-ERTAE  7 Selmo_127605/1-400 56.7% 43.1% --------------------------------------------------------------------------------  8 Sacu_v1.1_s0008.g003998/1-993 98.6% 26.9% EKAQKSEIALFQRMQILDDMVLKEASALGLQQAESDLQRKLLAAESEIQRLHGEVKAVLARAE--AAEVRASTA-DEALK  9 Sacu_v1.1_s0102.g019864:1-1500/1-1500 98.6% 17.2% EKAQKSEVALFQRMQILDDMVLKEASALGLQQTASELQRKLLASESEIQRLQGEVNAVLARAE--AAESRAAAA-YDALR  10 Azfi_s0015.g013850/1-998 98.4% 25.7% EKAQKSEIALFQRMQILDDMVLKEASALGLQKTASEIQRKLLAAESEIKRLQGEVTAVLARAE--AAESRASAA-DDALR  11 Bradi2g48150/1-868 94.6% 28.2% GPSVKRISEFYGEYPILKSEYVQNFVSVTVTENSETDKSLVEFDTDITGQVIIHWGVCKDNTM--TWEIPSEPH-PPKTK  12 AT1G69830/1-887 97.0% 26.4% IEERKGLQEFYEEMPISKRVADDNSVSVTARKCPETSKNIVSIETDLPGDVTVHWGVCKNGTK--KWEIPSEPY-PEETS  cov pid 401 . . . . : . . . 480  1 Cre08.g362450/1-706 100.0% 100.0% VERLKPLRSQSTQNDVDALELAAAEMTQAVAELVATERDIIR--------------------------------------  2 CHBRA206g00120/1-1039 99.2% 23.4% VYKKRAVQTVMKRDEGCDGDGEGSWIMVDVGGGFSELRFVLKEVDSNTWFDAEGEDFSMPLPAAKGPKDHGGGQEGAAAG  3 AagrBONN_Sc2ySwM_362.1200.1/1-412 58.1% 32.4% --------------------------------------------------------------------------------  4 Mapoly0024s0059/1-996 97.6% 25.0% EVQRAAAEDGRERDDRAKQTLEE--IKATLISKTEVASVVLQ-ADLEAL-----------KAAYHAAQEAGNVKEQANLR  5 Pp3c21_19790/1-956 98.3% 26.4% EVQKAASKAADEREASALTAIDA--VKKAAKARQVADKVAFE-AEADAL-----------RSANDASHKASEARRLVIKS  6 Pp3c22_22380/1-950 98.7% 26.6% ELQRAAAKEAEERADSAQSAINA--VKKATQVRLDADKIAFE-AELDAL-----------RSANDTSHKASEARRLVDKS  7 Selmo_127605/1-400 56.7% 43.1% ----------------------------------LDSSFCFK--------------------------------------  8 Sacu_v1.1_s0008.g003998/1-993 98.6% 26.9% QFQEAANISAHEQEGSAKKALEA--LKETGAARLEAARAAFK-ADVEVL-----------QSALETVQIAGKSKEQAYTR  9 Sacu_v1.1_s0102.g019864:1-1500/1-1500 98.6% 17.2% QYQEAAKRSAQEHEERAAKALEA--LKAAGAARLEAARSAFK-ADIEVL-----------QTALETVKIAEKSQEQAYAR  10 Azfi_s0015.g013850/1-998 98.4% 25.7% QFQERANQDALEHEERAKKALEA--LKLAGAARLEAARAAFK-SDIEVL-----------QAALDTVQIAGKSQEQAYAR  11 Bradi2g48150/1-868 94.6% 28.2% IFRQKALQTLLQQKTDGTGNT----ISFLLNADYSGLVFVLK-LDEYTW-----------LRNVD---------------  12 AT1G69830/1-887 97.0% 26.4% LFKNKALRTRLQRKDDGNGSFGL----FSLDGKLEGLCFVLK-LNENTW------------------------------L  cov pid 481 . 5 . . . . : . 560  1 Cre08.g362450/1-706 100.0% 100.0% -------------MRASN---ADARTLQTKEANRTEAAAGLE------------------RKMAAMQAVLAARRNL----  2 CHBRA206g00120/1-1039 99.2% 23.4% APGQTTSGVAASDDRDRD---RNRKKTDIARLEAKVAEPVLPIPIADMAEGGGIGVNGAPAAANVVSSSSATREDFAGTI  3 AagrBONN_Sc2ySwM_362.1200.1/1-412 58.1% 32.4% --------------------------------------------------------------------------------  4 Mapoly0024s0059/1-996 97.6% 25.0% MYEALERSLAAAEGSAEA---WKNRALSVEGLLRRVKEEGLEAVSSVVAEEMVAG-----GRMETLLGNDSRKRDL----  5 Pp3c21_19790/1-956 98.3% 26.4% RCESLEKSLVAAEGAAAA---WRNRALTAEELLRQSRINGVE---IDSSSPVPDLLPPNIGRLEMLPGSDAKIKDL----  6 Pp3c22_22380/1-950 98.7% 26.6% RFELLERSLLAVESATAA---WKNRALMAEKLLRLARINGAEIDTSSLPVEQAPSV----GRLEVLPGSDVRIKDL----  7 Selmo_127605/1-400 56.7% 43.1% --------------------------------------------------------------------------------  8 Sacu_v1.1_s0008.g003998/1-993 98.6% 26.9% KHQALERSLAAAETLAKA---WEERALAVESLLHKSRKEGAEQYAVELNGGVIDILTG--GRMETLLGNDSRKWEL----  9 Sacu_v1.1_s0102.g019864:1-1500/1-1500 98.6% 17.2% RSQALERSLAAAESLAKA---WEERALAVEYLLQKSGDECVD--ASERTRGFGVVLNG--GRMETLLGNDSRKWDL----  10 Azfi_s0015.g013850/1-998 98.4% 25.7% RYQALERSLSSAETLAKA---WEERALAVEALLQKSREEGAD--VAGFNVGLEGILTG--GRMETLLGNDSRKWDL----  11 Bradi2g48150/1-868 94.6% 28.2% --NGFDFYIPLKEPHKSDEQKVDDKSAQTDGLIGDIR--------------------------NLVVGLSSRR-------  12 AT1G69830/1-887 97.0% 26.4% NYRGEDFYVPFLTSSSSP---VETEAAQVSKPKRKTDKEVSASGFTK----------------EIITEIRNLAIDI----  cov pid 561 . . . 6 . . . . 640  1 Cre08.g362450/1-706 100.0% 100.0% ------------------ATEPEKPKSPTEK-----LLETLKPPTPMRA-------------------------------  2 CHBRA206g00120/1-1039 99.2% 23.4% APPAPPSPPPSPPSPSSPSPSPSTTEVAEEEVNLAAITRSAKGTGAVPAGTVSSNSRVVITSSGPGPEELLQEIDRLAAE  3 AagrBONN_Sc2ySwM_362.1200.1/1-412 58.1% 32.4% -----------------PKPKPAEPE------------------------------------------------------  4 Mapoly0024s0059/1-996 97.6% 25.0% ---------------LANGPRRETPEWMRRR-----IEVGFQGLPPRSSMPTNSEIEAQVPLHLPRPDEVW-SIFNAKVK  5 Pp3c21_19790/1-956 98.3% 26.4% ---------------LENGPRRETPDWMKRR-----LQTGQQNLPPMQPTSITADIDAAIPLELPTPEDVW-DVAKSKVK  6 Pp3c22_22380/1-950 98.7% 26.6% ---------------LENGPRRETPDWMKRR-----LQIGQQVLPPMQPIAINADVDALIPLQLPSSETVW-DVSKSKVK  7 Selmo_127605/1-400 56.7% 43.1% --------------------------------------------------------------------------------  8 Sacu_v1.1_s0008.g003998/1-993 98.6% 26.9% ---------------LSNGPRTDTPEWMERR-----IEVALQGLPARTG-STPVEQESTLSLQLPSPEEVW-CIATAEVK  9 Sacu_v1.1_s0102.g019864:1-1500/1-1500 98.6% 17.2% ---------------LSNGPRRETPEWMERS-----IETALQGLPPRS--LTQVQDEAGISLWLPSPEEVW-SIATAEVK  10 Azfi_s0015.g013850/1-998 98.4% 25.7% ---------------LANGPRRETPEWMERR-----IEVALQGLPPRKLGQI--EEEIGVSLKLPSPDEVW-SIATAEVK  11 Bradi2g48150/1-868 94.6% 28.2% -------------------GQRAKNKVLQED-----ILQEIERLAA----------------------------------  12 AT1G69830/1-887 97.0% 26.4% ---------------SSHKNQKTNVKEVQEN-----ILQEIEKLAA----------------------------------  cov pid 641 : . . . . 7 . . 720  1 Cre08.g362450/1-706 100.0% 100.0% -----------------------------------------------------AAGAGSGSGSEILLQAFNWESHRQ-KL  2 CHBRA206g00120/1-1039 99.2% 23.4% ASENFRRATVIAPAAPLSQQKLEQADSKPTTTLPLTSPQNVLPLPLPLSVPDRQPCPGTGSGKEILLQGFNWESHKTGRW  3 AagrBONN_Sc2ySwM_362.1200.1/1-412 58.1% 32.4% --------------------------------------------------------------TQILLQGFNWESHKSENW  4 Mapoly0024s0059/1-996 97.6% 25.0% EDDLYTKQAVEKEALDEQRRALERALQKKTVKRHPEDGEG-------------KLESGTGSGREIVFQGFNWESWRR-KW  5 Pp3c21_19790/1-956 98.3% 26.4% EDDKYTVRAAEKEALDLQRNALERALQTKSLRTLVRYPEE----------SESKTESGTGSGREIVFQGFNWESWRR-QW  6 Pp3c22_22380/1-950 98.7% 26.6% ENDKYAVRAAEKEALDLQRNAMERALQTKSIKTLVRYPEDAEE----------KSESGTGSGREIVFQGFNWESWRR-QW  7 Selmo_127605/1-400 56.7% 43.1% ------------------------------------------------------------------FQGFNWESCRK-RW  8 Sacu_v1.1_s0008.g003998/1-993 98.6% 26.9% E-DVLTREIAEKEAIDEQRRVLEKALKKKTVR---KTPQ--------------AMESGTGSGYEIVFQGFNWESWRK-KW  9 Sacu_v1.1_s0102.g019864:1-1500/1-1500 98.6% 17.2% E-DMYTREAAEKEAIDEQRRVLEKTLKKKAVR---KTPQ--------------ILESGTGSGREIVFQGFNWESWRK-QW  10 Azfi_s0015.g013850/1-998 98.4% 25.7% E-DVYTRQAAEKEAIDEQRRVLENTLKIKTVRKTAQ-----------------VLESGTGSGREIVFQGFNWESWRK-KW  11 Bradi2g48150/1-868 94.6% 28.2% --EAYSIFRSPTIDAVEDSVYIDDPATVK------------------------PACSGTGSGFEILCQGFNWESHKSGKW  12 AT1G69830/1-887 97.0% 26.4% --EAYSIFRSTTPAFSEEGVLEAEADKPDI-----------------------KISSGTGSGFEILCQGFNWESNKSGRW  cov pid 721 . . : . . . . 8 800  1 Cre08.g362450/1-706 100.0% 100.0% YKQLMGRVKDISDAGFTGVWMPPPSDSVSPQGYLPRDLYSLDSAYGSEAELRELIAAFHQNNIKVIADIVVNHRCANSQG  2 CHBRA206g00120/1-1039 99.2% 23.4% YNIIAEQAADIASGGFTAIWLPPPTDSISPEGYMPRDLYDLNSKYGDMEALKRVVKRLHEVGMVVLGDAVLNHRCAHFQG  3 AagrBONN_Sc2ySwM_362.1200.1/1-412 58.1% 32.4% YKTIASQADDLAGLGFTSIWLPPPSQSVAPQGYLPADLYNLDSKYGTLADLKEAIAKLHSVGIQVLADIVVNHRCAQSQN  4 Mapoly0024s0059/1-996 97.6% 25.0% YLELAPKAADLKKCGITTIWMPPPTESVAPQGYMPGDLYNLNSAYGTVDELKQCIEEMHNNDILVLGDAVLNHRCAQKQS  5 Pp3c21_19790/1-956 98.3% 26.4% WLEMSAKASDLAKCGITTIWLPPPTQSVAPQGYMPGDLYNLNSAYGGSEELKLCINEMHKHKILVLGDVVLNHRCAQKQS  6 Pp3c22_22380/1-950 98.7% 26.6% WLEMSAKASDLSKCGITTIWLPPPTHSVAPQGYMPGDLYNLNSAYGGSEELKQCIDEMHKHNILVLGDVVLNHRCAQKQS  7 Selmo_127605/1-400 56.7% 43.1% YQDLAPKAADLSQSGITTVWFPPPTESVAPQGYMPVDLYNLNSAYGSMDELKHCIQEMHKHDLLVLGDVVLNHRCAYKQN  8 Sacu_v1.1_s0008.g003998/1-993 98.6% 26.9% YLELGPKAADLSSCGITTIWFPPPTQSVSPQGYMPGDLYDLNSSYGTEEELKNCIEEMHNNELLVLGDAVLNHRCAQFKG  9 Sacu_v1.1_s0102.g019864:1-1500/1-1500 98.6% 17.2% YLELGPKAADLSSCGITTIWFPPPTQSVSPQGYMPGDLYNLNSSYGSVEELKNSIEEMHSNELLVLGDVVLNHRCAQFKG  10 Azfi_s0015.g013850/1-998 98.4% 25.7% YLELGPKAADLYSCGVTTIWFPPPTQSVSPQGYMPGDLYNLNSAYGTEEELKNCIEEMHNHELLVLGDAVLNHRCAQFKG  11 Bradi2g48150/1-868 94.6% 28.2% YVELGAKAKELASLGFTIVWSPPPTDSVSPEGYMPRDLYNLNSRYGTIEELKQLVNIFHEAGVKVLGDAVLNHRCAQFQN  12 AT1G69830/1-887 97.0% 26.4% YLELQEKADELASLGFTVLWLPPPTESVSPEGYMPKDLYNLNSRYGTIDELKDTVKKFHKVGIKVLGDAVLNHRCAHFKN  cov pid 801 . . . . : . . . 880  1 Cre08.g362450/1-706 100.0% 100.0% SDGKWNKFGGRLAWDASAICSNNPSFGGRGNPKQG----DDYAAAPNIDHSQERIRNDIVQWMKYLRNSIGFDGWRFDFV  2 CHBRA206g00120/1-1039 99.2% 23.4% PNGVWNRFGGKLAWDNRAIVCDDAHFDGAGNRSSG----DSFHAAPNIDHSQGFVRKDITEWLQWLRMEIGYDGWRLDFV  3 AagrBONN_Sc2ySwM_362.1200.1/1-412 58.1% 32.4% SQGIWNVYGGKMNWDARAIVSDDPNFQGQGNHSSG----ENFHAAPNIDHSQDFVRRDLCEWLQWLKSEVGFDGWRFDYV  4 Mapoly0024s0059/1-996 97.6% 25.0% PNGVWNIFGGKLAWGPEAIVKDDPNFQGRGNPSSG----DFFHAAPNIDHSQDFIRRDIKEWMKWLRSEIGFDGWRLDYV  5 Pp3c21_19790/1-956 98.3% 26.4% PNGVWNIFGGKLAWGPEAIVGDDPNFQGRGNPKSG----DFFHAAPNVDHSQKFVRKDIMEWMQWLRTEFGFDGWRLDFV  6 Pp3c22_22380/1-950 98.7% 26.6% PNGVWNRFGGKLNWGPEAIVRDDPNFQGQGNPKSG----DFFHAAPNIDHSQDFVRRDIIEWMKWLRSDFGFDGWRLDFV  7 Selmo_127605/1-400 56.7% 43.1% SNGVWNIFGGKLSWGPEAIVNDDPNFQGRGNPSSG----DIFHAAPNIDHSQAFVRKDIKEYLDWLKTEIGYDGWRLDFV  8 Sacu_v1.1_s0008.g003998/1-993 98.6% 26.9% PNGIWNVFGGKLAWGPDAIVRDDPNFQGRGNPSSG----DFFHAAPNIDHSQEFVRKDIKEWMKWLRTEIGFDGWRLDFV  9 Sacu_v1.1_s0102.g019864:1-1500/1-1500 98.6% 17.2% PNGIWNVFGGKLAWGPEAIVRDDPNFQGRGNPSSGNHLSDFFHAAPNIDHSQDFVRKDIKEWMKWLRTEIGFDGWRLDFV  10 Azfi_s0015.g013850/1-998 98.4% 25.7% PNGVWNVFGGKLAWGPEAIVRDDPNFQGRGNPSSG----DFFHAAPNIDHSQDFVQRDIKEWMKWLRTEIGFDGWRLDFV  11 Bradi2g48150/1-868 94.6% 28.2% QNGVWNIFGGRINWDDRAVVADDPHFQGRGNKSSG----DNFHAAPNIDHSQDFVRNDLKEWLCWMRKEVGYDGWRLDFV  12 AT1G69830/1-887 97.0% 26.4% QNGVWNLFGGRLNWDDRAVVADDPHFQGRGNKSSG----DNFHAAPNIDHSQDFVRKDIKEWLCWMMEEVGYDGWRLDFV  cov pid 881 . 9 . . . . : . 960  1 Cre08.g362450/1-706 100.0% 100.0% RGYLGSYCKQYIDETVPAMAFGEYWDSCEYTDGVLNYNQDAHRQRTVNWCDSTGGTSAAFDFTTKGILQEAVGRREYWRL  2 CHBRA206g00120/1-1039 99.2% 23.4% RGFWGGHVKEYIEGSNPWFAVGEYWDSLSYTYGEMDYNQDAHRQRIIDWMNATGGNAGAFDVTTKGILHTAIEKCEYWRL  3 AagrBONN_Sc2ySwM_362.1200.1/1-412 58.1% 32.4% RGFWGGHVKEYIDASQPSFSVGEYWDCMSYSNGQLDFNQNPHRQRIVNWINATGDKAAAFDFTTKGVLHAAIEKCEYWRL  4 Mapoly0024s0059/1-996 97.6% 25.0% RGFWGGYVKEYIEATDPAFSIGEYWDSLAYDGGQVSYNQDAHRQRIINWINATGGTSSAFDVTTKGILHSAL-HNEYWRL  5 Pp3c21_19790/1-956 98.3% 26.4% RGFWGGYVKEYIEATKPAFAIGEYWDSLSYEGGQVSYNQDAHRQRIVNWINATGGTSSAFDVTTKGILHSAL-HGEFWRL  6 Pp3c22_22380/1-950 98.7% 26.6% RGFWGGYVKEYIEATKPAFAIGEYWDSLAYEGGQVSYNQDAHRQRIVNWINAAGGTSSAFDVTTKGILHSAL-HGEFWRL  7 Selmo_127605/1-400 56.7% 43.1% RGFWGGYVKEYIEASEPAFAIGEYWDSLLYEGGNVAYNQDAHRQRIIDWINATGGTSSAFDVTTKGILHAAL-HNEYWRL  8 Sacu_v1.1_s0008.g003998/1-993 98.6% 26.9% RGFWGGYVKEYIEATDPAFAIGEYWDSLAYEGGNVCYNQDAHRQRIVNWINATGGTSSAFDVTTKGILHSAL-HNQYWRL  9 Sacu_v1.1_s0102.g019864:1-1500/1-1500 98.6% 17.2% RGFWGGYVKEYIEATEPAFAIGEYWDSLAYEGGNVCYNQDAHRQRIINWINATGGTSSAFDVTTKGILHSAL-HSQYWRL  10 Azfi_s0015.g013850/1-998 98.4% 25.7% RGFWGGYVKDYIEATDPAFAIGEYWDSLAYEGGNVCYNQDAHRQRIINWINATGGTSSAFDVTTKGILHSAL-HNQYWRL  11 Bradi2g48150/1-868 94.6% 28.2% RGFWGGYVKDYLEASEPYFAVGEYWDSLSYTYGEMDYNQDAHRQRIVDWINATSGTAGAFDVTTKGILHMALERSEYWRL  12 AT1G69830/1-887 97.0% 26.4% RGFWGGYVKDYMDASKPYFAVGEYWDSLSYTYGEMDYNQDAHRQRIVDWINATSGAAGAFDVTTKGILHTALQKCEYWRL  cov pid 961 . . . 0 . . . . 1040  1 Cre08.g362450/1-706 100.0% 100.0% VDSQGRPPGVMGMWPSRAITFIDNHDTGSTLNHWPFPSRNLPEGYAYILTHPGTPCVFYDHFYQEENNLRKIILDLLKVR  2 CHBRA206g00120/1-1039 99.2% 23.4% TDSERKPPGVVGWWPSRAVTFIENHDSGSTQGHWRFPQGREMLGYAYILTHPGTPTVFYDHWF--ANHLKEPIRILLALR  3 AagrBONN_Sc2ySwM_362.1200.1/1-412 58.1% 32.4% TDEKRKPPGVLGWWSSRAVTFIDNHDTGSTQGHWRFPAGKEVQGYAYILTHPGHVAVFYDHIK--DPKLREPIRKLIALR  4 Mapoly0024s0059/1-996 97.6% 25.0% IDPQGKPPGVMGWWPSRAVTFLENHDTGSTQGHWPFPRDKLMQGYAYILTHPGTPVIFYDHFY--DFGLHDQIAELIAAR  5 Pp3c21_19790/1-956 98.3% 26.4% IDPQGKPPGVMGWWPSRAVTFLENHDTGSTQGHWPFPRDKLMMGYAYILTHPGTPVIFHDHFY--DFGLHDQIAELIAVR  6 Pp3c22_22380/1-950 98.7% 26.6% IDPQGKPPGVMGWWPSRAVTFLENHDTGSTQGHWPFPRDKLMMGYAYILTHPGTPVIFHDHFY--DFGLHDQIADLIAVR  7 Selmo_127605/1-400 56.7% 43.1% IDPRQKPPGVMGWWPSRAVTFLENHDTGSTQGHWPFPRDKLLQGYAYILTHPGTPVIFYDHFY--DFGLRDPIVDLIAAR  8 Sacu_v1.1_s0008.g003998/1-993 98.6% 26.9% IDPNGKPPGVMGWWPSRAVTFLENHDTGSTQGHWPFPRDKLMQGYAYILTHPGTPVIFYDHFY--DFGLHDPIAELIAVR  9 Sacu_v1.1_s0102.g019864:1-1500/1-1500 98.6% 17.2% IDPNGKPPGVMGWWPSRAVTFLENHDTGSTQGHWPFPRDKLMQGYAYILTHPGTPVIFYDHFY--DFGLREPITELIAVR  10 Azfi_s0015.g013850/1-998 98.4% 25.7% IDPSGKPPGVMGWWPSRAVTFLENHDTGSTQGHWPFPRDKLMQGYAYILTHPGTPVIFYDHFY--DFGLRDAIAELIAVR  11 Bradi2g48150/1-868 94.6% 28.2% SDEKGKPPGVLGWWPSRAVTFIENHDTGSTQGHWRFPYGMEMQGYVYILTHPGTPAVFYDHVF--S-HLQQDIAKLISVR  12 AT1G69830/1-887 97.0% 26.4% SDPKGKPPGVVGWWPSRAVTFIENHDTGSTQGHWRFPEGKEMQGYAYILTHPGTPAVFFDHIF--S-DYHSEIAALLSLR  cov pid 1041 : . . . . 1 . . 1120  1 Cre08.g362450/1-706 100.0% 100.0% RRNGLNARSKVVMKKSAADVYAAMIDDKVAVKLGPGDWSPNQSGIKVNGKELKVAASGFQF-------------------  2 CHBRA206g00120/1-1039 99.2% 23.4% RRQGIHCRSVVRIQKAEKEVYGACIDEKVCMKIGPGHFDPPC-DET--KTW-VCVLEGQDF-------------------  3 AagrBONN_Sc2ySwM_362.1200.1/1-412 58.1% 32.4% KKANIHSKSNTNILEARKEFYAAIIDDRIVVKIGPGEYWPSG-HN-----WKLALE-GPDY-------------------  4 Mapoly0024s0059/1-996 97.6% 25.0% KRTAVHCRSPVKIFHANIEGYVAQVGENLVMKLGRLDWNPSK-ENNLAGSWERFLDRGSEY-------------------  5 Pp3c21_19790/1-956 98.3% 26.4% TRTGVHCRSPVKIFQANFEGYAAQIGENLVMKIGHLDWNPSK-QNNLPGSWDRCVDKG-EY-------------------  6 Pp3c22_22380/1-950 98.7% 26.6% TRTGVHCRSKVKIFQANFEGYAAQVGDNLVMKIGHLDWNPSK-QNNLAGSWNRCTDKG-EY-------------------  7 Selmo_127605/1-400 56.7% 43.1% NRTGINCRSPVKIFHANNDGYVAKVGEQLVVKLGRFDWNPSK-QNDLIGNWKRSVGQGSDY-------------------  8 Sacu_v1.1_s0008.g003998/1-993 98.6% 26.9% KRTGVNCRSPVKILQATNQGYAARIGDSLIVKLGFIDWNPSK-ENSLEGKWNRCLDKGADY-------------------  9 Sacu_v1.1_s0102.g019864:1-1500/1-1500 98.6% 17.2% KRTDVHCRSTVKIYQATNQGYAAQVGDNLVMKLGHLDWNPSK-ENNLEGKWSRCVDKDDSFSDITVRHRGNLEMLEQLYR  10 Azfi_s0015.g013850/1-998 98.4% 25.7% NRTGVNCRSPVKIYQATNQGYASQIGDNLVIKMGHLDWNPSK-ENNLEGKWNRCLDKGADY-------------------  11 Bradi2g48150/1-868 94.6% 28.2% RRLKIHCRSKIKILKAEQNLYAAEIDEKVTMKIGSGHFEPTG-PIN----WIVAVE-GQDY-------------------  12 AT1G69830/1-887 97.0% 26.4% NRQKLHCRSEVNIDKSERDVYAAIIDEKVAMKIGPGHYEPPN-GSQ---NWSVAVE-GRDY-------------------  cov pid 1121 . . : . . . . 2 1200  1 Cre08.g362450/1-706 100.0% 100.0% --------------------------------------------------------------------------------  2 CHBRA206g00120/1-1039 99.2% 23.4% --------------------------------------------------------------------------------  3 AagrBONN_Sc2ySwM_362.1200.1/1-412 58.1% 32.4% --------------------------------------------------------------------------------  4 Mapoly0024s0059/1-996 97.6% 25.0% --------------------------------------------------------------------------------  5 Pp3c21_19790/1-956 98.3% 26.4% --------------------------------------------------------------------------------  6 Pp3c22_22380/1-950 98.7% 26.6% --------------------------------------------------------------------------------  7 Selmo_127605/1-400 56.7% 43.1% --------------------------------------------------------------------------------  8 Sacu_v1.1_s0008.g003998/1-993 98.6% 26.9% --------------------------------------------------------------------------------  9 Sacu_v1.1_s0102.g019864:1-1500/1-1500 98.6% 17.2% GSECVYDDNSEIDAKLIAMAFACPPTRLLLFPDHLPPQHRLHPSSSPVPLFFHPPPFRLCFRILSISSSSSSLHKITELD  10 Azfi_s0015.g013850/1-998 98.4% 25.7% --------------------------------------------------------------------------------  11 Bradi2g48150/1-868 94.6% 28.2% --------------------------------------------------------------------------------  12 AT1G69830/1-887 97.0% 26.4% --------------------------------------------------------------------------------  cov pid 1201 . . . . : . . . 1280  1 Cre08.g362450/1-706 100.0% 100.0% --------------------------------------------------------------------------------  2 CHBRA206g00120/1-1039 99.2% 23.4% --------------------------------------------------------------------------------  3 AagrBONN_Sc2ySwM_362.1200.1/1-412 58.1% 32.4% --------------------------------------------------------------------------------  4 Mapoly0024s0059/1-996 97.6% 25.0% --------------------------------------------------------------------------------  5 Pp3c21_19790/1-956 98.3% 26.4% --------------------------------------------------------------------------------  6 Pp3c22_22380/1-950 98.7% 26.6% --------------------------------------------------------------------------------  7 Selmo_127605/1-400 56.7% 43.1% --------------------------------------------------------------------------------  8 Sacu_v1.1_s0008.g003998/1-993 98.6% 26.9% --------------------------------------------------------------------------------  9 Sacu_v1.1_s0102.g019864:1-1500/1-1500 98.6% 17.2% EGRPPPPPWQKFSTDAPPDDGSNPKPRAFRPAPWQQGSRDHRPQEPLRKQGAQTKRNFDGGAVEAEADKSALATIVEKLR  10 Azfi_s0015.g013850/1-998 98.4% 25.7% --------------------------------------------------------------------------------  11 Bradi2g48150/1-868 94.6% 28.2% --------------------------------------------------------------------------------  12 AT1G69830/1-887 97.0% 26.4% --------------------------------------------------------------------------------  cov pid 1281 . 3 . . . . : . 1360  1 Cre08.g362450/1-706 100.0% 100.0% --------------------------------------------------------------------------------  2 CHBRA206g00120/1-1039 99.2% 23.4% --------------------------------------------------------------------------------  3 AagrBONN_Sc2ySwM_362.1200.1/1-412 58.1% 32.4% --------------------------------------------------------------------------------  4 Mapoly0024s0059/1-996 97.6% 25.0% --------------------------------------------------------------------------------  5 Pp3c21_19790/1-956 98.3% 26.4% --------------------------------------------------------------------------------  6 Pp3c22_22380/1-950 98.7% 26.6% --------------------------------------------------------------------------------  7 Selmo_127605/1-400 56.7% 43.1% --------------------------------------------------------------------------------  8 Sacu_v1.1_s0008.g003998/1-993 98.6% 26.9% --------------------------------------------------------------------------------  9 Sacu_v1.1_s0102.g019864:1-1500/1-1500 98.6% 17.2% TIHDSLDASEPGTTGIAFNDSASSQSNADETHSSSSVSATEDKFPWEKPSESTEQETVVQVSQRRNPTRADLLIPPDELK  10 Azfi_s0015.g013850/1-998 98.4% 25.7% --------------------------------------------------------------------------------  11 Bradi2g48150/1-868 94.6% 28.2% --------------------------------------------------------------------------------  12 AT1G69830/1-887 97.0% 26.4% --------------------------------------------------------------------------------  cov pid 1361 . . . 4 . . . . 1440  1 Cre08.g362450/1-706 100.0% 100.0% --------------------------------------------------------------------------------  2 CHBRA206g00120/1-1039 99.2% 23.4% --------------------------------------------------------------------------------  3 AagrBONN_Sc2ySwM_362.1200.1/1-412 58.1% 32.4% --------------------------------------------------------------------------------  4 Mapoly0024s0059/1-996 97.6% 25.0% --------------------------------------------------------------------------------  5 Pp3c21_19790/1-956 98.3% 26.4% --------------------------------------------------------------------------------  6 Pp3c22_22380/1-950 98.7% 26.6% --------------------------------------------------------------------------------  7 Selmo_127605/1-400 56.7% 43.1% --------------------------------------------------------------------------------  8 Sacu_v1.1_s0008.g003998/1-993 98.6% 26.9% --------------------------------------------------------------------------------  9 Sacu_v1.1_s0102.g019864:1-1500/1-1500 98.6% 17.2% RLRMISPELQERLKIGKLGVTRSIVISLQQQWRTLELVKVRCQGPAANNIKKTLADLEENTGGLVIWRDKNAVVVYRGVG  10 Azfi_s0015.g013850/1-998 98.4% 25.7% --------------------------------------------------------------------------------  11 Bradi2g48150/1-868 94.6% 28.2% --------------------------------------------------------------------------------  12 AT1G69830/1-887 97.0% 26.4% --------------------------------------------------------------------------------  cov pid 1441 : . . . . 5 . . 1520  1 Cre08.g362450/1-706 100.0% 100.0% --------------------------------------------------------------------------------  2 CHBRA206g00120/1-1039 99.2% 23.4% --------------------------------------------------------------------------------  3 AagrBONN_Sc2ySwM_362.1200.1/1-412 58.1% 32.4% --------------------------------------------------------------------------------  4 Mapoly0024s0059/1-996 97.6% 25.0% --------------------------------------------------------------------------------  5 Pp3c21_19790/1-956 98.3% 26.4% --------------------------------------------------------------------------------  6 Pp3c22_22380/1-950 98.7% 26.6% --------------------------------------------------------------------------------  7 Selmo_127605/1-400 56.7% 43.1% --------------------------------------------------------------------------------  8 Sacu_v1.1_s0008.g003998/1-993 98.6% 26.9% --------------------------------------------------------------------------------  9 Sacu_v1.1_s0102.g019864:1-1500/1-1500 98.6% 17.2% FNPESDVGVKPEIETDISVAMGIEQNGATSIYGNHDRKNDETEMESLLDSLGPRYEKWTGLRPVPIDADLLPPEVPNYKP  10 Azfi_s0015.g013850/1-998 98.4% 25.7% --------------------------------------------------------------------------------  11 Bradi2g48150/1-868 94.6% 28.2% --------------------------------------------------------------------------------  12 AT1G69830/1-887 97.0% 26.4% --------------------------------------------------------------------------------  cov pid 1521 . . : . . . . 6 1600  1 Cre08.g362450/1-706 100.0% 100.0% ------------------------------------------------AVWEGQH-------------------------  2 CHBRA206g00120/1-1039 99.2% 23.4% ------------------------------------------------KVWEVSHN------------------------  3 AagrBONN_Sc2ySwM_362.1200.1/1-412 58.1% 32.4% ------------------------------------------------KVWERGS-------------------------  4 Mapoly0024s0059/1-996 97.6% 25.0% ------------------------------------------------QLWERK--------------------------  5 Pp3c21_19790/1-956 98.3% 26.4% ------------------------------------------------QLWERI--------------------------  6 Pp3c22_22380/1-950 98.7% 26.6% ------------------------------------------------QLWERK--------------------------  7 Selmo_127605/1-400 56.7% 43.1% ------------------------------------------------QVWEEE--------------------------  8 Sacu_v1.1_s0008.g003998/1-993 98.6% 26.9% ------------------------------------------------QIWERS--------------------------  9 Sacu_v1.1_s0102.g019864:1-1500/1-1500 98.6% 17.2% PFRLLPSGVWAGLTDAELTNLRRLARPLAPHFVLGRNKGQQGLAVAMLKLWEKTEIVKIAVKKRVQNTNNEMMAEQIRRL  10 Azfi_s0015.g013850/1-998 98.4% 25.7% ------------------------------------------------QIWERS--------------------------  11 Bradi2g48150/1-868 94.6% 28.2% ------------------------------------------------KIWEASS-------------------------  12 AT1G69830/1-887 97.0% 26.4% ------------------------------------------------KVWETS--------------------------  cov pid 1601 . ] 1612  1 Cre08.g362450/1-706 100.0% 100.0% ------------  2 CHBRA206g00120/1-1039 99.2% 23.4% ------------  3 AagrBONN_Sc2ySwM_362.1200.1/1-412 58.1% 32.4% ------------  4 Mapoly0024s0059/1-996 97.6% 25.0% ------------  5 Pp3c21_19790/1-956 98.3% 26.4% ------------  6 Pp3c22_22380/1-950 98.7% 26.6% ------------  7 Selmo_127605/1-400 56.7% 43.1% ------------  8 Sacu_v1.1_s0008.g003998/1-993 98.6% 26.9% ------------  9 Sacu_v1.1_s0102.g019864:1-1500/1-1500 98.6% 17.2% TGGVLLSRDKFF  10 Azfi_s0015.g013850/1-998 98.4% 25.7% ------------  11 Bradi2g48150/1-868 94.6% 28.2% ------------  12 AT1G69830/1-887 97.0% 26.4% ------------ |

>Cre08.g362450/1-706

-----------MDRSL------LSRTSPRMQLGRPQQLPPPTVPLAQVPRLQR--------RCVV-GSRVCQ

PVVAVRPGR--------ASA----------------------------------------------------

--------------GRGGRLVVSSVDMSNSPLSSMDAGEGLDIM----------------------------

--------------------------FDNNSDAECTVVTVEGKDKAHLLMSLTGGFSSA-------------

-------------------------------------------GLTVISASITSDDGRVLDVFRVQTADGKK

V--PEEQFPSVREHILSVTATSS--RSSMPAIYGIVAAAEVERLKPLRSQSTQNDVDALELAAAEMTQAVAE

LVATERDIIR---------------------------------------------------MRASN---ADA

RTLQTKEANRTEAAAGLE------------------RKMAAMQAVLAARRNL--------------------

--ATEPEKPKSPTEK-----LLETLKPPTPMRA---------------------------------------

---------------------------------------------AAGAGSGSGSEILLQAFNWESHRQ-KL

YKQLMGRVKDISDAGFTGVWMPPPSDSVSPQGYLPRDLYSLDSAYGSEAELRELIAAFHQNNIKVIADIVVN

HRCANSQGSDGKWNKFGGRLAWDASAICSNNPSFGGRGNPKQG----DDYAAAPNIDHSQERIRNDIVQWMK

YLRNSIGFDGWRFDFVRGYLGSYCKQYIDETVPAMAFGEYWDSCEYTDGVLNYNQDAHRQRTVNWCDSTGGT

SAAFDFTTKGILQEAVGRREYWRLVDSQGRPPGVMGMWPSRAITFIDNHDTGSTLNHWPFPSRNLPEGYAYI

LTHPGTPCVFYDHFYQEENNLRKIILDLLKVRRRNGLNARSKVVMKKSAADVYAAMIDDKVAVKLGPGDWSP

NQSGIKVNGKELKVAASGFQF---------------------------------------------------

------------------------------------------------------------------------

------------------------------------------------------------------------

------------------------------------------------------------------------

------------------------------------------------------------------------

------------------------------------------------------------------------

--------------------------------------------------------AVWEGQH---------

----------------------------

>CHBRA206g00120/1-1039

---------MGNGSKRYGSNNLHFAAVCEKGSGGLVTLPHLGGRLRGVGGARSGGHSDDGGDHST-SPRAAA

SAASAADGLLIVLSALADTQPAGQSQIEWPGDDQVQTTLQKDYA---LTRRVKVRGKLWVKVALWEN-LHRI

RISVECDVLHRALLHWGVTTREEAGRQWTQPPKKIRPPGTVKYKDYAARTPLRSCYGAVSDGGEEHGGGRVG

VDLE----------------------IAEEGAPEVIAFVLKDEETGRWHDKAGGNFIINLADLLE-------

--------------QRHERKTPAAAAHAAGGLRLLDGGAVGLLEETHDLENEVDIGNRLSAMVGLAEGGDVK

VLLSTDLSGPVLLHWGLVKRGEEQSKWTVPAKRFLPSNSTVYKKRAVQTVMKRDEGCDGDGEGSWIMVDVGG

GFSELRFVLKEVDSNTWFDAEGEDFSMPLPAAKGPKDHGGGQEGAAAGAPGQTTSGVAASDDRDRD---RNR

KKTDIARLEAKVAEPVLPIPIADMAEGGGIGVNGAPAAANVVSSSSATREDFAGTIAPPAPPSPPPSPPSPS

SPSPSPSTTEVAEEEVNLAAITRSAKGTGAVPAGTVSSNSRVVITSSGPGPEELLQEIDRLAAEASENFRRA

TVIAPAAPLSQQKLEQADSKPTTTLPLTSPQNVLPLPLPLSVPDRQPCPGTGSGKEILLQGFNWESHKTGRW

YNIIAEQAADIASGGFTAIWLPPPTDSISPEGYMPRDLYDLNSKYGDMEALKRVVKRLHEVGMVVLGDAVLN

HRCAHFQGPNGVWNRFGGKLAWDNRAIVCDDAHFDGAGNRSSG----DSFHAAPNIDHSQGFVRKDITEWLQ

WLRMEIGYDGWRLDFVRGFWGGHVKEYIEGSNPWFAVGEYWDSLSYTYGEMDYNQDAHRQRIIDWMNATGGN

AGAFDVTTKGILHTAIEKCEYWRLTDSERKPPGVVGWWPSRAVTFIENHDSGSTQGHWRFPQGREMLGYAYI

LTHPGTPTVFYDHWF--ANHLKEPIRILLALRRRQGIHCRSVVRIQKAEKEVYGACIDEKVCMKIGPGHFDP

PC-DET--KTW-VCVLEGQDF---------------------------------------------------

------------------------------------------------------------------------

------------------------------------------------------------------------

------------------------------------------------------------------------

------------------------------------------------------------------------

------------------------------------------------------------------------

--------------------------------------------------------KVWEVSHN--------

----------------------------

>AagrBONN_Sc2ySwM_362.1200.1/1-412

------------------------------------------------------------------------

------------------------------------------------------------------------

----------------------------MPPPSTPAPP----------------------------------

------------------------------------------------------------------------

------------------------------------------------------------------------

------------------------------------------------------------------------

------------------------------------------------------------------------

------------------------------------------------------------------------

-PKPKPAEPE--------------------------------------------------------------

------------------------------------------------------TQILLQGFNWESHKSENW

YKTIASQADDLAGLGFTSIWLPPPSQSVAPQGYLPADLYNLDSKYGTLADLKEAIAKLHSVGIQVLADIVVN

HRCAQSQNSQGIWNVYGGKMNWDARAIVSDDPNFQGQGNHSSG----ENFHAAPNIDHSQDFVRRDLCEWLQ

WLKSEVGFDGWRFDYVRGFWGGHVKEYIDASQPSFSVGEYWDCMSYSNGQLDFNQNPHRQRIVNWINATGDK

AAAFDFTTKGVLHAAIEKCEYWRLTDEKRKPPGVLGWWSSRAVTFIDNHDTGSTQGHWRFPAGKEVQGYAYI

LTHPGHVAVFYDHIK--DPKLREPIRKLIALRKKANIHSKSNTNILEARKEFYAAIIDDRIVVKIGPGEYWP

SG-HN-----WKLALE-GPDY---------------------------------------------------

------------------------------------------------------------------------

------------------------------------------------------------------------

------------------------------------------------------------------------

------------------------------------------------------------------------

------------------------------------------------------------------------

--------------------------------------------------------KVWERGS---------

----------------------------

>Mapoly0024s0059/1-996

------------MEIFATMQATFTTGPPLNSTEVRKCRAPDAALHQSLLHLRK-------------SPKVCS

VRHDD------------IFNSSGLSTPVKPFFGGKRKLYKWQRPSSELQKRENLPRLLVTASTAGIGSDNSS

ESSFGSNFP--DPVSLRAALKASQARVLEIENEKRDILEALRQSEAKVQEYAALMVQTTDEALSELEASKKL

FKAELSKVLEEKSTLQKETLLAKQDAVNLAVKIEKIAESAIQEATQRFAEDLVLKDSAAETAAAEAAAGVEE

SIRLAASDAAALVVTEASTVMEEALAAASLAKQQATKAQEALAKGMEIFEELSAAKLTTLSLQEKVSYLERE

LGISQGIVESLRLELKASQMRTE--AANARAAEA-EAAVQEVQRAAAEDGRERDDRAKQTLEE--IKATLIS

KTEVASVVLQ-ADLEAL-----------KAAYHAAQEAGNVKEQANLRMYEALERSLAAAEGSAEA---WKN

RALSVEGLLRRVKEEGLEAVSSVVAEEMVAG-----GRMETLLGNDSRKRDL-------------------L

ANGPRRETPEWMRRR-----IEVGFQGLPPRSSMPTNSEIEAQVPLHLPRPDEVW-SIFNAKVKEDDLYTKQ

AVEKEALDEQRRALERALQKKTVKRHPEDGEG-------------KLESGTGSGREIVFQGFNWESWRR-KW

YLELAPKAADLKKCGITTIWMPPPTESVAPQGYMPGDLYNLNSAYGTVDELKQCIEEMHNNDILVLGDAVLN

HRCAQKQSPNGVWNIFGGKLAWGPEAIVKDDPNFQGRGNPSSG----DFFHAAPNIDHSQDFIRRDIKEWMK

WLRSEIGFDGWRLDYVRGFWGGYVKEYIEATDPAFSIGEYWDSLAYDGGQVSYNQDAHRQRIINWINATGGT

SSAFDVTTKGILHSAL-HNEYWRLIDPQGKPPGVMGWWPSRAVTFLENHDTGSTQGHWPFPRDKLMQGYAYI

LTHPGTPVIFYDHFY--DFGLHDQIAELIAARKRTAVHCRSPVKIFHANIEGYVAQVGENLVMKLGRLDWNP

SK-ENNLAGSWERFLDRGSEY---------------------------------------------------

------------------------------------------------------------------------

------------------------------------------------------------------------

------------------------------------------------------------------------

------------------------------------------------------------------------

------------------------------------------------------------------------

--------------------------------------------------------QLWERK----------

----------------------------

>Pp3c21_19790/1-956

-----------MDTILTRSLAGVSGHLLPIGRGEFQSTSPSIHLISSYHCAGS-----NNNNSTIKTPFLGE

RVLVSR-----------NVNTTKALRRIWHGR---------------------------VFVSVLKG--ENN

NGSRGFQIP-DDVESLKVALAAAEARADAAKKAEKQALEALTAMEGKSSDTVKTSRNMKQIKLKGGNDDADG

--------------------------ISLAVQVEKISEAAIQKATARITEDATLKVAAAETAAAEAVLQLEE

RLQRAVDEAASAVAGETQVAIDEARAAAKVAKAQAAKSEALLNEQVNVLNELAEIEAKMLVLEEALLAAGRQ

LQIANGETERVRIELDAVQSFIK--TATARAEAA-EKTIIEVQKAASKAADEREASALTAIDA--VKKAAKA

RQVADKVAFE-AEADAL-----------RSANDASHKASEARRLVIKSRCESLEKSLVAAEGAAAA---WRN

RALTAEELLRQSRINGVE---IDSSSPVPDLLPPNIGRLEMLPGSDAKIKDL-------------------L

ENGPRRETPDWMKRR-----LQTGQQNLPPMQPTSITADIDAAIPLELPTPEDVW-DVAKSKVKEDDKYTVR

AAEKEALDLQRNALERALQTKSLRTLVRYPEE----------SESKTESGTGSGREIVFQGFNWESWRR-QW

WLEMSAKASDLAKCGITTIWLPPPTQSVAPQGYMPGDLYNLNSAYGGSEELKLCINEMHKHKILVLGDVVLN

HRCAQKQSPNGVWNIFGGKLAWGPEAIVGDDPNFQGRGNPKSG----DFFHAAPNVDHSQKFVRKDIMEWMQ

WLRTEFGFDGWRLDFVRGFWGGYVKEYIEATKPAFAIGEYWDSLSYEGGQVSYNQDAHRQRIVNWINATGGT

SSAFDVTTKGILHSAL-HGEFWRLIDPQGKPPGVMGWWPSRAVTFLENHDTGSTQGHWPFPRDKLMMGYAYI

LTHPGTPVIFHDHFY--DFGLHDQIAELIAVRTRTGVHCRSPVKIFQANFEGYAAQIGENLVMKIGHLDWNP

SK-QNNLPGSWDRCVDKG-EY---------------------------------------------------

------------------------------------------------------------------------

------------------------------------------------------------------------

------------------------------------------------------------------------

------------------------------------------------------------------------

------------------------------------------------------------------------

--------------------------------------------------------QLWERI----------

----------------------------

>Pp3c22_22380/1-950

-------MDTVSARSLAGMSSNLLSSPARTWHGEFRLVSQQIPLISSYHVTGS--------NTNN-STSVRT

SFFGERSPN--------TSK---------------------------VAKGQRRSRPDHAVVSVLKG--DRI

TGSRDVQAP-DDVESLKAALAAAEARTDAAKQAEKKALDALAAMKNKSRDMAQNDQNSQEVFEG--------

--------------------------ISLAVQVEKISESTIQKATLRITEDAELKIAAAETAAAEVILELED

QFRRAAEDAVQAASVEAQVTIDEARAAVSAARVQAEKSEAILNKQVKALNELAEAEAKVLMLEEALLDAGRK

LQLANGETERIRIELDSAQRFIK--TATARAEAA-ERTAEELQRAAAKEAEERADSAQSAINA--VKKATQV

RLDADKIAFE-AELDAL-----------RSANDTSHKASEARRLVDKSRFELLERSLLAVESATAA---WKN

RALMAEKLLRLARINGAEIDTSSLPVEQAPSV----GRLEVLPGSDVRIKDL-------------------L

ENGPRRETPDWMKRR-----LQIGQQVLPPMQPIAINADVDALIPLQLPSSETVW-DVSKSKVKENDKYAVR

AAEKEALDLQRNAMERALQTKSIKTLVRYPEDAEE----------KSESGTGSGREIVFQGFNWESWRR-QW

WLEMSAKASDLSKCGITTIWLPPPTHSVAPQGYMPGDLYNLNSAYGGSEELKQCIDEMHKHNILVLGDVVLN

HRCAQKQSPNGVWNRFGGKLNWGPEAIVRDDPNFQGQGNPKSG----DFFHAAPNIDHSQDFVRRDIIEWMK

WLRSDFGFDGWRLDFVRGFWGGYVKEYIEATKPAFAIGEYWDSLAYEGGQVSYNQDAHRQRIVNWINAAGGT

SSAFDVTTKGILHSAL-HGEFWRLIDPQGKPPGVMGWWPSRAVTFLENHDTGSTQGHWPFPRDKLMMGYAYI

LTHPGTPVIFHDHFY--DFGLHDQIADLIAVRTRTGVHCRSKVKIFQANFEGYAAQVGDNLVMKIGHLDWNP

SK-QNNLAGSWNRCTDKG-EY---------------------------------------------------

------------------------------------------------------------------------

------------------------------------------------------------------------

------------------------------------------------------------------------

------------------------------------------------------------------------

------------------------------------------------------------------------

--------------------------------------------------------QLWERK----------

----------------------------

>Selmo_127605/1-400 127605

------------------------------------------------------------------------

------------------------------------------------------------------------

------------------------------------------------------------------------

------------------------------------------------------------------------

------------------------------------------------------------------------

------------------------------------------------------------------------

--LDSSFCFK--------------------------------------------------------------

------------------------------------------------------------------------

------------------------------------------------------------------------

----------------------------------------------------------FQGFNWESCRK-RW

YQDLAPKAADLSQSGITTVWFPPPTESVAPQGYMPVDLYNLNSAYGSMDELKHCIQEMHKHDLLVLGDVVLN

HRCAYKQNSNGVWNIFGGKLSWGPEAIVNDDPNFQGRGNPSSG----DIFHAAPNIDHSQAFVRKDIKEYLD

WLKTEIGYDGWRLDFVRGFWGGYVKEYIEASEPAFAIGEYWDSLLYEGGNVAYNQDAHRQRIIDWINATGGT

SSAFDVTTKGILHAAL-HNEYWRLIDPRQKPPGVMGWWPSRAVTFLENHDTGSTQGHWPFPRDKLLQGYAYI

LTHPGTPVIFYDHFY--DFGLRDPIVDLIAARNRTGINCRSPVKIFHANNDGYVAKVGEQLVVKLGRFDWNP

SK-QNDLIGNWKRSVGQGSDY---------------------------------------------------

------------------------------------------------------------------------

------------------------------------------------------------------------

------------------------------------------------------------------------

------------------------------------------------------------------------

------------------------------------------------------------------------

--------------------------------------------------------QVWEEE----------

----------------------------

>Sacu_v1.1_s0008.g003998/1-993

------------MVSVVPMTFDAISGLPMFSKNKLDTIQPRNISSAGIHGSGS--------NSFS-GPSLRV

ETLSG-SGR--------YTQKLRLFRGI--KCSGNVVSRSGSTP---VEENDQGSSKAEQDAKVLVK--DFD

ANSIGQNLD--DIEGLKSALKAAHAKMLAAESEKADALRALAQAEARLREYATSAAETTESAVHEMEAAKES

VSIELQNIMKEKLATESELVVARSDAIELAVSVEKVADTILREATAHLIEEAQLKIAAAKTSAAEAAANVEE

RIKSAVHDTANAMIRETKDAIEKSFAALEAAKEKAQKSEIALFQRMQILDDMVLKEASALGLQQAESDLQRK

LLAAESEIQRLHGEVKAVLARAE--AAEVRASTA-DEALKQFQEAANISAHEQEGSAKKALEA--LKETGAA

RLEAARAAFK-ADVEVL-----------QSALETVQIAGKSKEQAYTRKHQALERSLAAAETLAKA---WEE

RALAVESLLHKSRKEGAEQYAVELNGGVIDILTG--GRMETLLGNDSRKWEL-------------------L

SNGPRTDTPEWMERR-----IEVALQGLPARTG-STPVEQESTLSLQLPSPEEVW-CIATAEVKE-DVLTRE

IAEKEAIDEQRRVLEKALKKKTVR---KTPQ--------------AMESGTGSGYEIVFQGFNWESWRK-KW

YLELGPKAADLSSCGITTIWFPPPTQSVSPQGYMPGDLYDLNSSYGTEEELKNCIEEMHNNELLVLGDAVLN

HRCAQFKGPNGIWNVFGGKLAWGPDAIVRDDPNFQGRGNPSSG----DFFHAAPNIDHSQEFVRKDIKEWMK

WLRTEIGFDGWRLDFVRGFWGGYVKEYIEATDPAFAIGEYWDSLAYEGGNVCYNQDAHRQRIVNWINATGGT

SSAFDVTTKGILHSAL-HNQYWRLIDPNGKPPGVMGWWPSRAVTFLENHDTGSTQGHWPFPRDKLMQGYAYI

LTHPGTPVIFYDHFY--DFGLHDPIAELIAVRKRTGVNCRSPVKILQATNQGYAARIGDSLIVKLGFIDWNP

SK-ENSLEGKWNRCLDKGADY---------------------------------------------------

------------------------------------------------------------------------

------------------------------------------------------------------------

------------------------------------------------------------------------

------------------------------------------------------------------------

------------------------------------------------------------------------

--------------------------------------------------------QIWERS----------

----------------------------

>Sacu_v1.1_s0102.g019864:1-1500/1-1500

------------MVSVALTQHDIISGLCSLSSNKVDSILQSRYACISTVAANL--------KPLR-APVVYI

EGLAV-SGR--------NPFQSGISRGI--RCS-NHVPSSGLTP---VEEGGKDGSQLEEAIQGMVK--DSR

DGEADLNIDMNNVDSIKAALMAAHARIAAAENEKADALRSLEVAETRLEEYASTAVQVTESAVHEMQAAKES

VNMELKNIMEQKLALESELVVVKKDALELALSVDKVADSILGEATTHLAEEARLKVAAAKTSAAEAAANVEE

RIRSAILDTTDNLIKETRDAIEKSFAALEAAKEKAQKSEVALFQRMQILDDMVLKEASALGLQQTASELQRK

LLASESEIQRLQGEVNAVLARAE--AAESRAAAA-YDALRQYQEAAKRSAQEHEERAAKALEA--LKAAGAA

RLEAARSAFK-ADIEVL-----------QTALETVKIAEKSQEQAYARRSQALERSLAAAESLAKA---WEE

RALAVEYLLQKSGDECVD--ASERTRGFGVVLNG--GRMETLLGNDSRKWDL-------------------L

SNGPRRETPEWMERS-----IETALQGLPPRS--LTQVQDEAGISLWLPSPEEVW-SIATAEVKE-DMYTRE

AAEKEAIDEQRRVLEKTLKKKAVR---KTPQ--------------ILESGTGSGREIVFQGFNWESWRK-QW

YLELGPKAADLSSCGITTIWFPPPTQSVSPQGYMPGDLYNLNSSYGSVEELKNSIEEMHSNELLVLGDVVLN

HRCAQFKGPNGIWNVFGGKLAWGPEAIVRDDPNFQGRGNPSSGNHLSDFFHAAPNIDHSQDFVRKDIKEWMK

WLRTEIGFDGWRLDFVRGFWGGYVKEYIEATEPAFAIGEYWDSLAYEGGNVCYNQDAHRQRIINWINATGGT

SSAFDVTTKGILHSAL-HSQYWRLIDPNGKPPGVMGWWPSRAVTFLENHDTGSTQGHWPFPRDKLMQGYAYI

LTHPGTPVIFYDHFY--DFGLREPITELIAVRKRTDVHCRSTVKIYQATNQGYAAQVGDNLVMKLGHLDWNP

SK-ENNLEGKWSRCVDKDDSFSDITVRHRGNLEMLEQLYRGSECVYDDNSEIDAKLIAMAFACPPTRLLLFP

DHLPPQHRLHPSSSPVPLFFHPPPFRLCFRILSISSSSSSLHKITELDEGRPPPPPWQKFSTDAPPDDGSNP

KPRAFRPAPWQQGSRDHRPQEPLRKQGAQTKRNFDGGAVEAEADKSALATIVEKLRTIHDSLDASEPGTTGI

AFNDSASSQSNADETHSSSSVSATEDKFPWEKPSESTEQETVVQVSQRRNPTRADLLIPPDELKRLRMISPE

LQERLKIGKLGVTRSIVISLQQQWRTLELVKVRCQGPAANNIKKTLADLEENTGGLVIWRDKNAVVVYRGVG

FNPESDVGVKPEIETDISVAMGIEQNGATSIYGNHDRKNDETEMESLLDSLGPRYEKWTGLRPVPIDADLLP

PEVPNYKPPFRLLPSGVWAGLTDAELTNLRRLARPLAPHFVLGRNKGQQGLAVAMLKLWEKTEIVKIAVKKR

VQNTNNEMMAEQIRRLTGGVLLSRDKFF

>Azfi_s0015.g013850/1-998

MKPWDIDLLLMEKVSILPKQHDTFSCLCTIS---KSTIIQPRIASTGIHLSSS--------KSFS-GPLLCV

ETLAAVSGR--------HLPPSGVSR--WIRCSGNRFSRSGSSP---IEGGQDASQLEETIKRLVDE-----

-GSIGQNIDLNDVEGLKTILVAAHARIVAAESEKEDALKALAEAEAKLQEYTSTAVQATEEPVKKTKHSNKS

EGVDLQSIIDQKLAVESELAVAKKNAIELAVCVDKVADAIYEETTASLAEEAHLKIAEAKTSAAEAANSVEE

RVKSAVLDTANAMIRETRDAIEKSFSALEAAKEKAQKSEIALFQRMQILDDMVLKEASALGLQKTASEIQRK

LLAAESEIKRLQGEVTAVLARAE--AAESRASAA-DDALRQFQERANQDALEHEERAKKALEA--LKLAGAA

RLEAARAAFK-SDIEVL-----------QAALDTVQIAGKSQEQAYARRYQALERSLSSAETLAKA---WEE

RALAVEALLQKSREEGAD--VAGFNVGLEGILTG--GRMETLLGNDSRKWDL-------------------L

ANGPRRETPEWMERR-----IEVALQGLPPRKLGQI--EEEIGVSLKLPSPDEVW-SIATAEVKE-DVYTRQ

AAEKEAIDEQRRVLENTLKIKTVRKTAQ-----------------VLESGTGSGREIVFQGFNWESWRK-KW

YLELGPKAADLYSCGVTTIWFPPPTQSVSPQGYMPGDLYNLNSAYGTEEELKNCIEEMHNHELLVLGDAVLN

HRCAQFKGPNGVWNVFGGKLAWGPEAIVRDDPNFQGRGNPSSG----DFFHAAPNIDHSQDFVQRDIKEWMK

WLRTEIGFDGWRLDFVRGFWGGYVKDYIEATDPAFAIGEYWDSLAYEGGNVCYNQDAHRQRIINWINATGGT

SSAFDVTTKGILHSAL-HNQYWRLIDPSGKPPGVMGWWPSRAVTFLENHDTGSTQGHWPFPRDKLMQGYAYI

LTHPGTPVIFYDHFY--DFGLRDAIAELIAVRNRTGVNCRSPVKIYQATNQGYASQIGDNLVIKMGHLDWNP

SK-ENNLEGKWNRCLDKGADY---------------------------------------------------

------------------------------------------------------------------------

------------------------------------------------------------------------

------------------------------------------------------------------------

------------------------------------------------------------------------

------------------------------------------------------------------------

--------------------------------------------------------QIWERS----------

----------------------------

>Bradi2g48150/1-868

----------------MSAASWSIPAIPRAAPPARGGLPGDAFLVAARPGPGR--------RRAAPGRRLRL

RG----GGV--------VVARAGAAEVPVTHPEESGVVFSEKFP---LRRCKTVQGKAWARVVAEPDGEGMC

KIVIGCDVEGKWVLHWGVSYDGEQGREWDQPPSEVRPPGSVTIKDYAIETPLVGSPNSEGHMVHEVEIK---

--------------------------FNQDTPIAIINFVLKEEETGAWFQHKGGDFRIPLSGSLEDGDPF--

----GAQQDTVHPGAKPEGSSAQPQETVPGDKGPSVKRISEFYGEYPILKSEYVQNFVSVTVTENSETDKSL

VEFDTDITGQVIIHWGVCKDNTM--TWEIPSEPH-PPKTKIFRQKALQTLLQQKTDGTGNT----ISFLLNA

DYSGLVFVLK-LDEYTW-----------LRNVD-----------------NGFDFYIPLKEPHKSDEQKVDD

KSAQTDGLIGDIR--------------------------NLVVGLSSRR-----------------------

---GQRAKNKVLQED-----ILQEIERLAA------------------------------------EAYSIF

RSPTIDAVEDSVYIDDPATVK------------------------PACSGTGSGFEILCQGFNWESHKSGKW

YVELGAKAKELASLGFTIVWSPPPTDSVSPEGYMPRDLYNLNSRYGTIEELKQLVNIFHEAGVKVLGDAVLN

HRCAQFQNQNGVWNIFGGRINWDDRAVVADDPHFQGRGNKSSG----DNFHAAPNIDHSQDFVRNDLKEWLC

WMRKEVGYDGWRLDFVRGFWGGYVKDYLEASEPYFAVGEYWDSLSYTYGEMDYNQDAHRQRIVDWINATSGT

AGAFDVTTKGILHMALERSEYWRLSDEKGKPPGVLGWWPSRAVTFIENHDTGSTQGHWRFPYGMEMQGYVYI

LTHPGTPAVFYDHVF--S-HLQQDIAKLISVRRRLKIHCRSKIKILKAEQNLYAAEIDEKVTMKIGSGHFEP

TG-PIN----WIVAVE-GQDY---------------------------------------------------

------------------------------------------------------------------------

------------------------------------------------------------------------

------------------------------------------------------------------------

------------------------------------------------------------------------

------------------------------------------------------------------------

--------------------------------------------------------KIWEASS---------

----------------------------

>AT1G69830/1-887

---------MSTVPIESLLHHSYLRHNSKVNRGNRSFIPISLNLRSHFTSNKL--------LHSI-GKSVGV

SSMNK-SPV--------AIRATSSDTAVVETAQSDDVIFKEIFP---VQRIEKAEGKIYVRLKEVKE--KNW

ELSVGCSIPGKWILHWGVSYVGDTGSEWDQPPEDMRPPGSIAIKDYAIETPLKKLSEGDSFFEVAIN-----

--------------------------LNLESSVAALNFVLKDEETGAWYQHKGRDFKVPLVDDVPDNGNL--

---IGAKKGFGALGQLSNIPLKQDKSSAETDSIEERKGLQEFYEEMPISKRVADDNSVSVTARKCPETSKNI

VSIETDLPGDVTVHWGVCKNGTK--KWEIPSEPY-PEETSLFKNKALRTRLQRKDDGNGSFGL----FSLDG

KLEGLCFVLK-LNENTW------------------------------LNYRGEDFYVPFLTSSSSP---VET

EAAQVSKPKRKTDKEVSASGFTK----------------EIITEIRNLAIDI-------------------S

SHKNQKTNVKEVQEN-----ILQEIEKLAA------------------------------------EAYSIF

RSTTPAFSEEGVLEAEADKPDI-----------------------KISSGTGSGFEILCQGFNWESNKSGRW

YLELQEKADELASLGFTVLWLPPPTESVSPEGYMPKDLYNLNSRYGTIDELKDTVKKFHKVGIKVLGDAVLN

HRCAHFKNQNGVWNLFGGRLNWDDRAVVADDPHFQGRGNKSSG----DNFHAAPNIDHSQDFVRKDIKEWLC

WMMEEVGYDGWRLDFVRGFWGGYVKDYMDASKPYFAVGEYWDSLSYTYGEMDYNQDAHRQRIVDWINATSGA

AGAFDVTTKGILHTALQKCEYWRLSDPKGKPPGVVGWWPSRAVTFIENHDTGSTQGHWRFPEGKEMQGYAYI

LTHPGTPAVFFDHIF--S-DYHSEIAALLSLRNRQKLHCRSEVNIDKSERDVYAAIIDEKVAMKIGPGHYEP

PN-GSQ---NWSVAVE-GRDY---------------------------------------------------

------------------------------------------------------------------------

------------------------------------------------------------------------

------------------------------------------------------------------------

------------------------------------------------------------------------

------------------------------------------------------------------------

--------------------------------------------------------KVWETS----------

----------------------------

**Apoferredoxin 1**

cov pid  **1** **[ . . . . : . . .** **80**

1 Selmo_166487/1-146 100.0% 100.0%  **---------------------------------------------------------------------------MAALA**

2 Selmo_236581/1-97 65.1% 51.5%  **--------------------------------------------------------------------------------**

3 Selmo_38551/1-101 68.5% 48.5%  **--------------------------------------------------------------------------------**

4 AT1G10960_Fd1/1-148 96.6% 34.5%  **----------------------------------------------------------------------------MAST**

5 AT1G60950_Fd2/1-148 96.6% 35.8%  **----------------------------------------------------------------------------MAST**

6 AT2G27510_Fd3/1-155 95.9% 32.9%  **-----------------------------------------------------------------------------MAT**

7 AT5G10000_fd4/1-148 95.9% 31.1%  **-----------------------------------------------------------------------------MDQ**

8 Bradi1g02590/1-152 96.6% 36.2%  **----------------------------------------------------------------------------MLTS**

9 Bradi1g53230/1-148 96.6% 37.8%  **----------------------------------------------------------------------------MSTC**

10 Bradi2g23931/1-268 97.3% 20.1%  **MRICQGRLGTKVWYPIGQWPPRPFAQAARRQPTAQRKEEACVPGQGQREPGRVAEAESDSPLLIRRRARASHLTSSHLAI**

11 Bradi2g55680/1-224 97.3% 24.1%  **--------------------------------------------------MAVSRRTPAAYGSRRGCYWCSSKHLTAINT**

12 Bradi3g13160/1-140 95.2% 36.6%  **-----------------------------------------------------------------------------MAT**

13 Bradi3g13170/1-144 97.3% 37.9%  **--------------------------------------------------------------------------MATALS**

14 Bradi5g09320/1-159 95.9% 31.4%  **-----------------------------------------------------------------------------MTT**

15 Cre14.g626700/1-126 85.6% 36.9%  **-----------------------------------------------------------------------------MAM**

16 Cre16.g658400/1-121 82.2% 37.9%  **--------------------------------------------------------------------------------**

17 Cre17.g700950/1-130 85.6% 24.7%  **----------------------------------------------------------------------------MLCA**

18 Mapoly0082s0023/1-142 96.6% 36.6%  **----------------------------------------------------------------------------MAMA**

19 Mapoly3477s0001/1-156 97.3% 35.3%  **---------------------------------------------------------------------MASAVSTTVAA**

20 Pp3c10_16880/1-149 97.3% 32.9%  **--------------------------------------------------------------------------MAAAVA**

21 Pp3c17_18910/1-148 95.2% 32.2%  **-----------------------------------------------------------------------------MAA**

22 Pp3c3_22119/1-152 96.6% 33.6%  **----------------------------------------------------------------------------MAAA**

23 Pp3c3_22140/1-152 96.6% 33.6%  **----------------------------------------------------------------------------MAAA**

24 Pp3c4_8159/1-145 95.2% 30.3%  **-----------------------------------------------------------------------------MAA**

25 Pp3c4_8171/1-145 95.2% 30.3%  **-----------------------------------------------------------------------------MAA**

26 Pp3c4_8330/1-145 95.2% 30.3%  **-----------------------------------------------------------------------------MAA**

27 Sacu_v1.1_s0069.g016503/1-155 96.6% 32.3%  **--------------------------------------------------------------------------MAASTI**

28 Sacu_v1.1_s0059.g015062/1-131 84.9% 33.6%  **---------------------------------------------------------------------------MAAAA**

29 CHBRA25g00470/1-207 97.9% 26.1%  **---------------------------------------------------------------------------MSTSA**

30 CHBRA37g00180/1-155 96.6% 32.3%  **----------------------------------------------------------------------------MAST**

31 CHBRA52g00290/1-235 97.3% 24.7%  **------------------------------------------MEVMEAMAAVPSWCVGNAAGARGQEFNTPEKCLGKSLS**

32 Azfi_s0092.g043125/1-166 95.9% 30.7%  **----------------------------------------------------------------------------MAAP**

33 Azfi_s0003.g007828/1-154 97.3% 33.8%  **----------------------------------------------------------------------------MATC**

34 Azfi_s0393.g067829/1-234 95.9% 21.8%  **----------------------------------------------------------------------------MAAA**

35 Azfi_s0006.g009850/1-141 95.9% 36.8%  **----------------------------------------------------------------------------MASA**

cov pid  **81**  **. 1 . . . . : .** **160**

1 Selmo_166487/1-146 100.0% 100.0%  **LGDHPRTVFSNLGLDLRRRAAIGIELQSRR--------------------------------------------------**

2 Selmo_236581/1-97 65.1% 51.5%  **--------------------------------------------------------------------------------**

3 Selmo_38551/1-101 68.5% 48.5%  **--------------------------------------------------------------------------------**

4 AT1G10960_Fd1/1-148 96.6% 34.5%  **ALSSAIVSTSFLRRQQTPISLRSLPFANTQSLFGLK--------------------------------------------**

5 AT1G60950_Fd2/1-148 96.6% 35.8%  **ALSSAIVGTSFIRRSPAPISLRSLPSANTQSLFGLK--------------------------------------------**

6 AT2G27510_Fd3/1-155 95.9% 32.9%  **VRISSTSMTKAVLRSQTTNKLITNKSYNLSVGST----------------------------------------------**

7 AT5G10000_fd4/1-148 95.9% 31.1%  **VLYSSYIIKIPVISRISPSQAQLTTRLNNTTYFGLS--------------------------------------------**

8 Bradi1g02590/1-152 96.6% 36.2%  **TFATSCTLFSNVTTEASQKVVKSPSSLSFFSQGVLQVLS-----------------------------------------**

9 Bradi1g53230/1-148 96.6% 37.8%  **TVVASCAPASNFGIKASMAAVKVNSVGFAGHRAVI---------------------------------------------**

10 Bradi2g23931/1-268 97.3% 20.1%  **QSPNRLFAFPRLGDQRAPQPTRPPPISSGRRRRRISKMSIATAPCVRIARSGAGCRAVSPAIRSPSFVGYTKQTSNLSG-**

11 Bradi2g55680/1-224 97.3% 24.1%  **TTCVLCPSSCDILRSRVPSRLLDLPASFRALNSNLEFSCSSRPMPTAISLPTGCAPANRGCTTLAAPPMRSPASLAQQP-**

12 Bradi3g13160/1-140 95.2% 36.6%  **TALSSLSLFTAAPPPSSAVSSRVVLPLT----------------------------------------------------**

13 Bradi3g13170/1-144 97.3% 37.9%  **SLRAPFSLFTSAAPAPSPIVAPSRVALPL---------------------------------------------------**

14 Bradi5g09320/1-159 95.9% 31.4%  **TFTTPIFCNFRANPHLQQLPLRSHSSTRQAQHLHLSPVRIMP--------------------------------------**

15 Cre14.g626700/1-126 85.6% 36.9%  **AMRSTFAARVGAKPAV----------------------------------------------------------------**

16 Cre16.g658400/1-121 82.2% 37.9%  **-----MIVRRPMLTARAPARVAVAPL------------------------------------------------------**

17 Cre17.g700950/1-130 85.6% 24.7%  **RSQLVCKPVKAARASRATVKVQ----------------------------------------------------------**

18 Mapoly0082s0023/1-142 96.6% 36.6%  **VCASAVVALAAPVSSLKAVSMKNTNVSKAF--------------------------------------------------**

19 Mapoly3477s0001/1-156 97.3% 35.3%  **ATAFGGLATSSPAAAGAGRNLRTTSVSFRARSVGHAF-------------------------------------------**

20 Pp3c10_16880/1-149 97.3% 32.9%  **GTMSTLASVATFVPTCRVTAVRSSSVSIAKAFG-----------------------------------------------**

21 Pp3c17_18910/1-148 95.2% 32.2%  **VALGPISMPSRTAASASVLSFPDAMSSSQ---------------------------------------------------**

22 Pp3c3_22119/1-152 96.6% 33.6%  **VSRSVVPVGSAAVAAASPCSQASSRNHVSM--------------------------------------------------**

23 Pp3c3_22140/1-152 96.6% 33.6%  **VSRSVVPVGSAAVAAASPCSQASSRNHVSM--------------------------------------------------**

24 Pp3c4_8159/1-145 95.2% 30.3%  **AAMTSIVPVASIAPVSKVANVRPSSVSVAKAFG-----------------------------------------------**

25 Pp3c4_8171/1-145 95.2% 30.3%  **AAMTSIVPVASIAPVSKVANVRPSSVSVAKAFG-----------------------------------------------**

26 Pp3c4_8330/1-145 95.2% 30.3%  **AAMTSIVPVASIAPVSKVANVRPSSVSVAKAFG-----------------------------------------------**

27 Sacu_v1.1_s0069.g016503/1-155 96.6% 32.3%  **ILPSSFCSPCSLRPSSSLKASSPLPSTSTSPHP-----------------------------------------------**

28 Sacu_v1.1_s0059.g015062/1-131 84.9% 33.6%  **ATAQTVCASTAIGAGLKRGVRLEKAGGAGELWGKVA--------------------------------------------**

29 CHBRA25g00470/1-207 97.9% 26.1%  **SVAAAATAAAPASSAGTILGGAGSAATSATQRRRDTSCRLGPGQPGHLVWVPVKCSAFANVGQQRAAVRRELGPRQPGRL**

30 CHBRA37g00180/1-155 96.6% 32.3%  **IAAAATTGAIAAASLGAIAKVSASPRSSVSLKMT----------------------------------------------**

31 CHBRA52g00290/1-235 97.3% 24.7%  **TARTSVSPPSPSVGGTRFPPFSLSRASSPTLFPRFCLARASSLSTM--SSCFPPTTESKNWRCPVLARRRMYGSVGSSGL**

32 Azfi_s0092.g043125/1-166 95.9% 30.7%  **LLLLSTSSPSLSLRSHSPASFSPAGLHNNASSSLLCLDRLP--ARS----------------------------------**

33 Azfi_s0003.g007828/1-154 97.3% 33.8%  **TTSTSMVMGSLANPSSAPVLLGGLSSQRVSL-------------------------------------------------**

34 Azfi_s0393.g067829/1-234 95.9% 21.8%  **SSSSILCSINHTMPAPVSTPLLVPSPSSSSSFVSLACPSFLLHRAAHSHGANFAYCKVPRISLSCNVTMRQYPAMGDVDM**

35 Azfi_s0006.g009850/1-141 95.9% 36.8%  **SAMSSILSHPPHQQQQRFHTNLTISNA-----------------------------------------------------**

cov pid **161**  **. . . 2 . . . .** **240**

1 Selmo_166487/1-146 100.0% 100.0%  **----ERLKPSFAIRA----------T------------------------AYRVLLKMPL-G-EKMI-SCPDDKSILDAA**

2 Selmo_236581/1-97 65.1% 51.5%  **------------------------MA------------------------KHKVTLKLED-GSEKTF-QCPDDVYILDEA**

3 Selmo_38551/1-101 68.5% 48.5%  **----------SRLRV---------WA------------------------AYKVTLVTPE-G-EKVL-SVPEDSYILDAA**

4 AT1G10960_Fd1/1-148 96.6% 34.5%  **----SSTARGGRVTA---------MA------------------------TYKVKFITPE-G-EQEV-ECEEDVYVLDAA**

5 AT1G60950_Fd2/1-148 96.6% 35.8%  **----SGTARGGRVTA---------MA------------------------TYKVKFITPE-G-ELEV-ECDDDVYVLDAA**

6 AT2G27510_Fd3/1-155 95.9% 32.9%  **----KRVSRSFGLKCSANSGGATMSA------------------------VYKVKLLGPD-GQEDEF-EVQDDQYILDAA**

7 AT5G10000_fd4/1-148 95.9% 31.1%  **----SSRGNFGKVFA---------KE------------------------SRKVKLISPE-GEEQEI-EGNEDCCILESA**

8 Bradi1g02590/1-152 96.6% 36.2%  **----LKSSKKLDVSA---------MA------------------------VYKVKLVTPE-GQEHEF-EAPDDTYILDAA**

9 Bradi1g53230/1-148 96.6% 37.8%  **----PSLKTSRNLAA---------MA------------------------VYKVKLVTPE-GDEHEF-EAPDDTYILDAA**

10 Bradi2g23931/1-268 97.3% 20.1%  **----LRISNKFRVSA---------TA------------------------VHKVKLVGPD-GEEHEF-EAPEDTYILEAA**

11 Bradi2g55680/1-224 97.3% 24.1%  **----RPNRRSGRARL---SARFGPAA------------------------AHKVRLVGPD-GAETEL-EVGEDAYILDAA**

12 Bradi3g13160/1-140 95.2% 36.6%  **----APSARGMRLRA---------QA------------------------TYKVKLVTPE-G-EVDL-EVPDDVYILDHA**

13 Bradi3g13170/1-144 97.3% 37.9%  **----TPSARAIRLRA---------QA------------------------TYKVKLVTPE-G-EVEM-EVPDDVYILDHF**

14 Bradi5g09320/1-159 95.9% 31.4%  **----IPTSPGSRTSNDFRAA----AA------------------------AYKVKLIGPE-GKESVL-DVAEDSYILDAA**

15 Cre14.g626700/1-126 85.6% 36.9%  **----RGARPASRMSC---------MA-------------------------YKVTLKTPS-G-DKTI-ECPADTYILDAA**

16 Cre16.g658400/1-121 82.2% 37.9%  **-----------RTRV---------IS------------------------HFKVTFKTPK-G-EKTI-DVEADKYLLDAA**

17 Cre17.g700950/1-130 85.6% 24.7%  **--------------------------------------------------AFQVTLRMPS-GKTKTM-EVGPDEALFDAV**

18 Mapoly0082s0023/1-142 96.6% 36.6%  **----GLTSSRSRVTC---------MA------------------------TYKVTLNTPT-G-QSVI-DVDDDVYILDAA**

19 Mapoly3477s0001/1-156 97.3% 35.3%  **----GLKAAKSRLTC---------ST------------------------AYTITLKTPS-G-EKVF-ECEEDTYILDAA**

20 Pp3c10_16880/1-149 97.3% 32.9%  **----LKTQSMGRVTC---------MA------------------------SYQVTIKNGETGEVSSF-ECPDDEYILDAA**

21 Pp3c17_18910/1-148 95.2% 32.2%  **----LILRRSFRINALHVSGRVI-AA------------------------AYQVTLITPE-A-ERVV-HVSKDECILDAA**

22 Pp3c3_22119/1-152 96.6% 33.6%  **----GWSNASSRFAGLQASSRGSVKA------------------------GYTVTLKTKDAG-DVTF-EVDGSTYILDAA**

23 Pp3c3_22140/1-152 96.6% 33.6%  **----GWSNASSRFAGLQASSRGSVKA------------------------GYTVTLKTKDAG-DVTF-EVDGSTYILDAA**

24 Pp3c4_8159/1-145 95.2% 30.3%  **----LKSRSMGRLTC---------MA------------------------TYKVTFLDGETGAENVV-ECSDEEYVLDAA**

25 Pp3c4_8171/1-145 95.2% 30.3%  **----LKSRSMGRLTC---------MA------------------------TYKVTFLDGETGAENVV-ECSDEEYVLDAA**

26 Pp3c4_8330/1-145 95.2% 30.3%  **----LKSRSMGRLTC---------MA------------------------TYKVTFLDGETGAENVV-ECSDEEYVLDAA**

27 Sacu_v1.1_s0069.g016503/1-155 96.6% 32.3%  **----RPRRMLPVAASKGGPGKGDHHK------------------------GFKVKFLTPD-G-EKVV-NIPGDKYILDGG**

28 Sacu_v1.1_s0059.g015062/1-131 84.9% 33.6%  **----GGKGVGGRVRM---------EA------------------------VYKVTFQTQE-G-DMTI-DVPASTYLLDAA**

29 CHBRA25g00470/1-207 97.9% 26.1%  **VVRPSLRPDTGRSFGLSRSPSVTQCA------------------------VYQVTLKTPD-G-DKII-DCMDDEYILDKA**

30 CHBRA37g00180/1-155 96.6% 32.3%  **----PKIGRLNAARVFGVQPSLGRCQ------------------------AYKVTFITGK-D-EVVV-DCPEDVYVLDAA**

31 CHBRA52g00290/1-235 97.3% 24.7%  **GLGRVCTWKSFGLRAADVSGG---AT------------------------CYQVTFKIPD-GETTTI-EVEEDEFILDKA**

32 Azfi_s0092.g043125/1-166 95.9% 30.7%  **----SVAITGIRASSDPKKGKPPQPR------------------------VYKVKFLLPE-GAEPVLVDVPPNTYILDAA**

33 Azfi_s0003.g007828/1-154 97.3% 33.8%  **----QKKSDSNSLKFGLSGSRMGRIQ------------------------AFKVTLKVKDVG-ETVL-EVPADQYILDAA**

34 Azfi_s0393.g067829/1-234 95.9% 21.8%  **----LAATSGCRVSAIGKGSYGHVAAKRDSYRASAVGKGSSHEDLKKKKNTYTVKFITPE-GEETVV-EVPGDKYLLDAG**

35 Azfi_s0006.g009850/1-141 95.9% 36.8%  **----RKSMVVRAMSG---------TQ------------------------TFKVTLKTPQ-G-EKIL-NVPEDVYISDAA**

cov pid **241**  **: . . . . 3 . .** **320**

1 Selmo_166487/1-146 100.0% 100.0%  **EDAN----LELPFF--CRAGSCPACAGKLEM---------GSVDQGGNAFMGYEHIDQGFVLT------CIAYPTSDLVV**

2 Selmo_236581/1-97 65.1% 51.5%  **EEQS----IDLPSS--CRAGSCSSCAGKVVS---------GSVDQTDQNFLDDDQIGNGFVLT------CVARPTSDVVI**

3 Selmo_38551/1-101 68.5% 48.5%  **EEQG----VELPYS--CKSGACSSCAGMVKL---------GEVDQRDQTFLTDLQVKQGYVLT------CVAYPVSDLVI**

4 AT1G10960_Fd1/1-148 96.6% 34.5%  **EEAG----LDLPYS--CRAGSCSSCAGKVVS---------GSIDQSDQSFLDDEQMSEGYVLT------CVAYPTSDVVI**

5 AT1G60950_Fd2/1-148 96.6% 35.8%  **EEAG----IDLPYS--CRAGSCSSCAGKVVS---------GSVDQSDQSFLDDEQIGEGFVLT------CAAYPTSDVTI**

6 AT2G27510_Fd3/1-155 95.9% 32.9%  **EEAG----VDLPYS--CRAGACSTCAGQIVS---------GNVDQSDGSFLEDSHLEKGYVLT------CVAYPQSDCVI**

7 AT5G10000_fd4/1-148 95.9% 31.1%  **ENAG----LELPYS--CRSGTCGTCCGKLVS---------GKVDQSLGSFLEEEQIQKGYILT------CIALPLEDCVV**

8 Bradi1g02590/1-152 96.6% 36.2%  **ETAG----VELPYS--CRAGACSTCAGKIEA---------GSVDQSDGSFLDDGQQEEGYVLT------CVSYPKSDCVI**

9 Bradi1g53230/1-148 96.6% 37.8%  **ETAG----VELPYS--CRAGACSTCAGKIEA---------GTIDQSDGSFLDDDQQSEGYVLT------CVSYPKSDCVI**

10 Bradi2g23931/1-268 97.3% 20.1%  **ENAG----VELPFS--CRAGSCSTCAGKMTT---------GEVDQSEGSFLDENQMGEGYLLT------CISYPKADCVI**

11 Bradi2g55680/1-224 97.3% 24.1%  **EEAG----VELPFS--CRAGSCSTCAGKLAS---------GEVDQSEGSFLDDAQLAQGYVLT------CVAYPKADCVI**

12 Bradi3g13160/1-140 95.2% 36.6%  **EEEG----IDLPYS--CRAGSCSSCAGKVVS---------GEVDQSDQSFLDDDQVAAGWVLT------CAAYPQSDLVI**

13 Bradi3g13170/1-144 97.3% 37.9%  **EEEG----IDLPYS--CRAGSCSSCAGKVIS---------GEVDQSDQSFLDDDQMEAGWVLT------CHAYPKSDLVI**

14 Bradi5g09320/1-159 95.9% 31.4%  **EEAG----VELPYS--CRAGACSTCAGKVLE---------GAVDQSDQSFLDEAQVGAGYALT------CVAYPTSDCVI**

15 Cre14.g626700/1-126 85.6% 36.9%  **EEAG----LDLPYS--CRAGACSSCAGKVAA---------GTVDQSDQSFLDDAQMGNGFVLT------CVAYPTSDCTI**

16 Cre16.g658400/1-121 82.2% 37.9%  **EEAG----MDLPYS--CRSGGCSTCCGKLES---------GTVDQSDQNMLDEDQLKQGFVLT------CVAYPTSDIVI**

17 Cre17.g700950/1-130 85.6% 24.7%  **ERYD----VDLPYL--CRTGTCGTCAGRVQE---------GQVELKGQHILDPDQVKAGFILM------CSAYPRSDCTI**

18 Mapoly0082s0023/1-142 96.6% 36.6%  **EEAG----LSLPYS--CRAGACSSCAGKVTA---------GEVDQADQSFLDDDQMDEGFVLT------CIAYPTSDVTI**

19 Mapoly3477s0001/1-156 97.3% 35.3%  **ESAG----AELPYS--CRAGACCSCAGKVLS---------GEVDQSDGSFLDDEQIAKGYLLT------CISYPKSDCVI**

20 Pp3c10_16880/1-149 97.3% 32.9%  **EELG----ITLPCS--CRSGACSSCAGLLQQ---------GSVDQSEQSFLDDSQVGAGYVLT------CVAYPTSDCII**

21 Pp3c17_18910/1-148 95.2% 32.2%  **EREG----VLLPHS--CRSGACSTCVGLLKS---------GSLNQDEQSFLDEAQLQAGFALM------CASYPTSDCVI**

22 Pp3c3_22119/1-152 96.6% 33.6%  **EEAG----VDLPYS--CRAGACSTCAGQIKE---------GTVDQSDGSFLDDEQMEKGFVLT------CVAYPTSDLVI**

23 Pp3c3_22140/1-152 96.6% 33.6%  **EEAG----VDLPYS--CRAGACSTCAGQIKE---------GTVDQSDGSFLDDEQMEKGFVLT------CVAYPTSDLVI**

24 Pp3c4_8159/1-145 95.2% 30.3%  **ERAG----MDLPYS--CRAGACSSCAGIIKA---------GEVDQSDQSFLDDSQIDDGFVLT------CVAYPASDCII**

25 Pp3c4_8171/1-145 95.2% 30.3%  **ERAG----MDLPYS--CRAGACSSCAGIIKA---------GEVDQSDQSFLDDSQIDDGFVLT------CVAYPVSDCTI**

26 Pp3c4_8330/1-145 95.2% 30.3%  **ERAG----MDLPYS--CRAGACSSCAGIIKA---------GEVDQSDQSFLDDSQIDDGFVLT------CVAYPASDCII**

27 Sacu_v1.1_s0069.g016503/1-155 96.6% 32.3%  **EEAG----LDLPYS--CRAGACSSCCAQIVE---------GTVDQSEQSFLDDDQIKEGFVLT------CVSYPTSDLVL**

28 Sacu_v1.1_s0059.g015062/1-131 84.9% 33.6%  **EEEG----LNLPYS--CRAGACSSCAGKLSS---------GTVDQSQQSFLDDAQISDGFVLT------CI---------**

29 CHBRA25g00470/1-207 97.9% 26.1%  **EELG----LILPSS--CRSGACSACAGKILA---------GTVDQGDQSFLTDSQLGSGYVLT------CVAYPTSDVVI**

30 CHBRA37g00180/1-155 96.6% 32.3%  **EEAG----LDLPYS--CRAGACSTCAGKLEK---------GTIDQSDQSFLDDDQVEAGFVLT------CVTYPTSDCTI**

31 CHBRA52g00290/1-235 97.3% 24.7%  **EEMG----LDLPYS--CRAGSCSTCAAVLES---------GSVDQSNQSFLDDEQIEKGFVLI------CQAYPKSDIVL**

32 Azfi_s0092.g043125/1-166 95.9% 30.7%  **EEAG----LDLPYS--CRAGACSSCAGKVIK---------GSVDQADQSFLDDDQIGKGFLLT------CVSYATSDLTI**

33 Azfi_s0003.g007828/1-154 97.3% 33.8%  **EEAG----LDLPYS--CRAGACSSCTGKVVS---------GTVDQSEQSFLDDDQVAEGFVLT------CCAFVTSDVVI**

34 Azfi_s0393.g067829/1-234 95.9% 21.8%  **EEAG----VDLPYS--CRAGACSSCAVRILNLKNANGEEEGTVDQSDQSFLDEEQIHKGFVLA------CVAYPTSDLVL**

35 Azfi_s0006.g009850/1-141 95.9% 36.8%  **EEAG----LTLPIS--CRSGNCSSCAAILKS---------GEVDQSYQTFLDESRVAQGFVLT------CVAYPLSDVVI**

cov pid **321**  **. ]** **337**

1 Selmo_166487/1-146 100.0% 100.0%  **ITHAEDELVASLL----**

2 Selmo_236581/1-97 65.1% 51.5%  **LTHQEDNI---------**

3 Selmo_38551/1-101 68.5% 48.5%  **QTHQEEKL---------**

4 AT1G10960_Fd1/1-148 96.6% 34.5%  **ETHKEEAIM--------**

5 AT1G60950_Fd2/1-148 96.6% 35.8%  **ETHKEEDIV--------**

6 AT2G27510_Fd3/1-155 95.9% 32.9%  **HTHKETELF--------**

7 AT5G10000_fd4/1-148 95.9% 31.1%  **YTHKQSDLI--------**

8 Bradi1g02590/1-152 96.6% 36.2%  **HTHKEGDLY--------**

9 Bradi1g53230/1-148 96.6% 37.8%  **HTHKEGELY--------**

10 Bradi2g23931/1-268 97.3% 20.1%  **HTHQEEELY--------**

11 Bradi2g55680/1-224 97.3% 24.1%  **YTHKEDEVH--------**

12 Bradi3g13160/1-140 95.2% 36.6%  **ETHKEEELTA-------**

13 Bradi3g13170/1-144 97.3% 37.9%  **ETHKEEELTA-------**

14 Bradi5g09320/1-159 95.9% 31.4%  **QTHRESDLY--------**

15 Cre14.g626700/1-126 85.6% 36.9%  **QTHQEEALY--------**

16 Cre16.g658400/1-121 82.2% 37.9%  **LTDQESKL---------**

17 Cre17.g700950/1-130 85.6% 24.7%  **LTHQEERLHTCEYGKHQ**

18 Mapoly0082s0023/1-142 96.6% 36.6%  **DTHQEEAIM--------**

19 Mapoly3477s0001/1-156 97.3% 35.3%  **ETHQEEHVV--------**

20 Pp3c10_16880/1-149 97.3% 32.9%  **VSHQEQALA--------**

21 Pp3c17_18910/1-148 95.2% 32.2%  **ETHQEEKFY--------**

22 Pp3c3_22119/1-152 96.6% 33.6%  **ETHKEEDLQ--------**

23 Pp3c3_22140/1-152 96.6% 33.6%  **ETHKEEDLQ--------**

24 Pp3c4_8159/1-145 95.2% 30.3%  **LTHQEENM---------**

25 Pp3c4_8171/1-145 95.2% 30.3%  **LTHQEENM---------**

26 Pp3c4_8330/1-145 95.2% 30.3%  **LTHQEENM---------**

27 Sacu_v1.1_s0069.g016503/1-155 96.6% 32.3%  **TTHCEDKL---------**

28 Sacu_v1.1_s0059.g015062/1-131 84.9% 33.6%  **-----------------**

29 CHBRA25g00470/1-207 97.9% 26.1%  **ETGKESEVGS-------**

30 CHBRA37g00180/1-155 96.6% 32.3%  **VTNQEDKIY--------**

31 CHBRA52g00290/1-235 97.3% 24.7%  **TTHKEEELY--------**

32 Azfi_s0092.g043125/1-166 95.9% 30.7%  **KTHCEDEI---------**

33 Azfi_s0003.g007828/1-154 97.3% 33.8%  **ETHQEDNLTL-------**

34 Azfi_s0393.g067829/1-234 95.9% 21.8%  **QTHCEDQL---------**

35 Azfi_s0006.g009850/1-141 95.9% 36.8%  **ETHQESLLYDS------**

>Selmo_166487/1-146

------------------------------------------------------------------------

---MAALALGDHPRTVFSNLGLDLRRRAAIGIELQSRR----------------------------------

--------------------ERLKPSFAIRA----------T------------------------AYRVLL

KMPL-G-EKMI-SCPDDKSILDAAEDAN----LELPFF--CRAGSCPACAGKLEM---------GSVDQGGN

AFMGYEHIDQGFVLT------CIAYPTSDLVVITHAEDELVASLL----

>Selmo_236581/1-97

------------------------------------------------------------------------

------------------------------------------------------------------------

----------------------------------------MA------------------------KHKVTL

KLED-GSEKTF-QCPDDVYILDEAEEQS----IDLPSS--CRAGSCSSCAGKVVS---------GSVDQTDQ

NFLDDDQIGNGFVLT------CVARPTSDVVILTHQEDNI---------

>Selmo_38551/1-101

------------------------------------------------------------------------

------------------------------------------------------------------------

--------------------------SRLRV---------WA------------------------AYKVTL

VTPE-G-EKVL-SVPEDSYILDAAEEQG----VELPYS--CKSGACSSCAGMVKL---------GEVDQRDQ

TFLTDLQVKQGYVLT------CVAYPVSDLVIQTHQEEKL---------

>AT1G10960_Fd1/1-148

------------------------------------------------------------------------

----MASTALSSAIVSTSFLRRQQTPISLRSLPFANTQSLFGLK----------------------------

--------------------SSTARGGRVTA---------MA------------------------TYKVKF

ITPE-G-EQEV-ECEEDVYVLDAAEEAG----LDLPYS--CRAGSCSSCAGKVVS---------GSIDQSDQ

SFLDDEQMSEGYVLT------CVAYPTSDVVIETHKEEAIM--------

>AT1G60950_Fd2/1-148

------------------------------------------------------------------------

----MASTALSSAIVGTSFIRRSPAPISLRSLPSANTQSLFGLK----------------------------

--------------------SGTARGGRVTA---------MA------------------------TYKVKF

ITPE-G-ELEV-ECDDDVYVLDAAEEAG----IDLPYS--CRAGSCSSCAGKVVS---------GSVDQSDQ

SFLDDEQIGEGFVLT------CAAYPTSDVTIETHKEEDIV--------

>AT2G27510_Fd3/1-155

------------------------------------------------------------------------

-----MATVRISSTSMTKAVLRSQTTNKLITNKSYNLSVGST------------------------------

--------------------KRVSRSFGLKCSANSGGATMSA------------------------VYKVKL

LGPD-GQEDEF-EVQDDQYILDAAEEAG----VDLPYS--CRAGACSTCAGQIVS---------GNVDQSDG

SFLEDSHLEKGYVLT------CVAYPQSDCVIHTHKETELF--------

>AT5G10000_fd4/1-148

------------------------------------------------------------------------

-----MDQVLYSSYIIKIPVISRISPSQAQLTTRLNNTTYFGLS----------------------------

--------------------SSRGNFGKVFA---------KE------------------------SRKVKL

ISPE-GEEQEI-EGNEDCCILESAENAG----LELPYS--CRSGTCGTCCGKLVS---------GKVDQSLG

SFLEEEQIQKGYILT------CIALPLEDCVVYTHKQSDLI--------

>Bradi1g02590/1-152

------------------------------------------------------------------------

----MLTSTFATSCTLFSNVTTEASQKVVKSPSSLSFFSQGVLQVLS-------------------------

--------------------LKSSKKLDVSA---------MA------------------------VYKVKL

VTPE-GQEHEF-EAPDDTYILDAAETAG----VELPYS--CRAGACSTCAGKIEA---------GSVDQSDG

SFLDDGQQEEGYVLT------CVSYPKSDCVIHTHKEGDLY--------

>Bradi1g53230/1-148

------------------------------------------------------------------------

----MSTCTVVASCAPASNFGIKASMAAVKVNSVGFAGHRAVI-----------------------------

--------------------PSLKTSRNLAA---------MA------------------------VYKVKL

VTPE-GDEHEF-EAPDDTYILDAAETAG----VELPYS--CRAGACSTCAGKIEA---------GTIDQSDG

SFLDDDQQSEGYVLT------CVSYPKSDCVIHTHKEGELY--------

>Bradi2g23931/1-268

MRICQGRLGTKVWYPIGQWPPRPFAQAARRQPTAQRKEEACVPGQGQREPGRVAEAESDSPLLIRRRARASH

LTSSHLAIQSPNRLFAFPRLGDQRAPQPTRPPPISSGRRRRRISKMSIATAPCVRIARSGAGCRAVSPAIRS

PSFVGYTKQTSNLSG-----LRISNKFRVSA---------TA------------------------VHKVKL

VGPD-GEEHEF-EAPEDTYILEAAENAG----VELPFS--CRAGSCSTCAGKMTT---------GEVDQSEG

SFLDENQMGEGYLLT------CISYPKADCVIHTHQEEELY--------

>Bradi2g55680/1-224

--------------------------------------------------MAVSRRTPAAYGSRRGCYWCSS

KHLTAINTTTCVLCPSSCDILRSRVPSRLLDLPASFRALNSNLEFSCSSRPMPTAISLPTGCAPANRGCTTL

AAPPMRSPASLAQQP-----RPNRRSGRARL---SARFGPAA------------------------AHKVRL

VGPD-GAETEL-EVGEDAYILDAAEEAG----VELPFS--CRAGSCSTCAGKLAS---------GEVDQSEG

SFLDDAQLAQGYVLT------CVAYPKADCVIYTHKEDEVH--------

>Bradi3g13160/1-140

------------------------------------------------------------------------

-----MATTALSSLSLFTAAPPPSSAVSSRVVLPLT------------------------------------

--------------------APSARGMRLRA---------QA------------------------TYKVKL

VTPE-G-EVDL-EVPDDVYILDHAEEEG----IDLPYS--CRAGSCSSCAGKVVS---------GEVDQSDQ

SFLDDDQVAAGWVLT------CAAYPQSDLVIETHKEEELTA-------

>Bradi3g13170/1-144

------------------------------------------------------------------------

--MATALSSLRAPFSLFTSAAPAPSPIVAPSRVALPL-----------------------------------

--------------------TPSARAIRLRA---------QA------------------------TYKVKL

VTPE-G-EVEM-EVPDDVYILDHFEEEG----IDLPYS--CRAGSCSSCAGKVIS---------GEVDQSDQ

SFLDDDQMEAGWVLT------CHAYPKSDLVIETHKEEELTA-------

>Bradi5g09320/1-159

------------------------------------------------------------------------

-----MTTTFTTPIFCNFRANPHLQQLPLRSHSSTRQAQHLHLSPVRIMP----------------------

--------------------IPTSPGSRTSNDFRAA----AA------------------------AYKVKL

IGPE-GKESVL-DVAEDSYILDAAEEAG----VELPYS--CRAGACSTCAGKVLE---------GAVDQSDQ

SFLDEAQVGAGYALT------CVAYPTSDCVIQTHRESDLY--------

>Cre14.g626700/1-126

------------------------------------------------------------------------

-----MAMAMRSTFAARVGAKPAV------------------------------------------------

--------------------RGARPASRMSC---------MA-------------------------YKVTL

KTPS-G-DKTI-ECPADTYILDAAEEAG----LDLPYS--CRAGACSSCAGKVAA---------GTVDQSDQ

SFLDDAQMGNGFVLT------CVAYPTSDCTIQTHQEEALY--------

>Cre16.g658400/1-121

------------------------------------------------------------------------

-------------MIVRRPMLTARAPARVAVAPL--------------------------------------

---------------------------RTRV---------IS------------------------HFKVTF

KTPK-G-EKTI-DVEADKYLLDAAEEAG----MDLPYS--CRSGGCSTCCGKLES---------GTVDQSDQ

NMLDEDQLKQGFVLT------CVAYPTSDIVILTDQESKL---------

>Cre17.g700950/1-130

------------------------------------------------------------------------

----MLCARSQLVCKPVKAARASRATVKVQ------------------------------------------

------------------------------------------------------------------AFQVTL

RMPS-GKTKTM-EVGPDEALFDAVERYD----VDLPYL--CRTGTCGTCAGRVQE---------GQVELKGQ

HILDPDQVKAGFILM------CSAYPRSDCTILTHQEERLHTCEYGKHQ

>Mapoly0082s0023/1-142

------------------------------------------------------------------------

----MAMAVCASAVVALAAPVSSLKAVSMKNTNVSKAF----------------------------------

--------------------GLTSSRSRVTC---------MA------------------------TYKVTL

NTPT-G-QSVI-DVDDDVYILDAAEEAG----LSLPYS--CRAGACSSCAGKVTA---------GEVDQADQ

SFLDDDQMDEGFVLT------CIAYPTSDVTIDTHQEEAIM--------

>Mapoly3477s0001/1-156

---------------------------------------------------------------------MAS

AVSTTVAAATAFGGLATSSPAAAGAGRNLRTTSVSFRARSVGHAF---------------------------

--------------------GLKAAKSRLTC---------ST------------------------AYTITL

KTPS-G-EKVF-ECEEDTYILDAAESAG----AELPYS--CRAGACCSCAGKVLS---------GEVDQSDG

SFLDDEQIAKGYLLT------CISYPKSDCVIETHQEEHVV--------

>Pp3c10_16880/1-149

------------------------------------------------------------------------

--MAAAVAGTMSTLASVATFVPTCRVTAVRSSSVSIAKAFG-------------------------------

--------------------LKTQSMGRVTC---------MA------------------------SYQVTI

KNGETGEVSSF-ECPDDEYILDAAEELG----ITLPCS--CRSGACSSCAGLLQQ---------GSVDQSEQ

SFLDDSQVGAGYVLT------CVAYPTSDCIIVSHQEQALA--------

>Pp3c17_18910/1-148

------------------------------------------------------------------------

-----MAAVALGPISMPSRTAASASVLSFPDAMSSSQ-----------------------------------

--------------------LILRRSFRINALHVSGRVI-AA------------------------AYQVTL

ITPE-A-ERVV-HVSKDECILDAAEREG----VLLPHS--CRSGACSTCVGLLKS---------GSLNQDEQ

SFLDEAQLQAGFALM------CASYPTSDCVIETHQEEKFY--------

>Pp3c3_22119/1-152

------------------------------------------------------------------------

----MAAAVSRSVVPVGSAAVAAASPCSQASSRNHVSM----------------------------------

--------------------GWSNASSRFAGLQASSRGSVKA------------------------GYTVTL

KTKDAG-DVTF-EVDGSTYILDAAEEAG----VDLPYS--CRAGACSTCAGQIKE---------GTVDQSDG

SFLDDEQMEKGFVLT------CVAYPTSDLVIETHKEEDLQ--------

>Pp3c3_22140/1-152

------------------------------------------------------------------------

----MAAAVSRSVVPVGSAAVAAASPCSQASSRNHVSM----------------------------------

--------------------GWSNASSRFAGLQASSRGSVKA------------------------GYTVTL

KTKDAG-DVTF-EVDGSTYILDAAEEAG----VDLPYS--CRAGACSTCAGQIKE---------GTVDQSDG

SFLDDEQMEKGFVLT------CVAYPTSDLVIETHKEEDLQ--------

>Pp3c4_8159/1-145

------------------------------------------------------------------------

-----MAAAAMTSIVPVASIAPVSKVANVRPSSVSVAKAFG-------------------------------

--------------------LKSRSMGRLTC---------MA------------------------TYKVTF

LDGETGAENVV-ECSDEEYVLDAAERAG----MDLPYS--CRAGACSSCAGIIKA---------GEVDQSDQ

SFLDDSQIDDGFVLT------CVAYPASDCIILTHQEENM---------

>Pp3c4_8171/1-145

------------------------------------------------------------------------

-----MAAAAMTSIVPVASIAPVSKVANVRPSSVSVAKAFG-------------------------------

--------------------LKSRSMGRLTC---------MA------------------------TYKVTF

LDGETGAENVV-ECSDEEYVLDAAERAG----MDLPYS--CRAGACSSCAGIIKA---------GEVDQSDQ

SFLDDSQIDDGFVLT------CVAYPVSDCTILTHQEENM---------

>Pp3c4_8330/1-145

------------------------------------------------------------------------

-----MAAAAMTSIVPVASIAPVSKVANVRPSSVSVAKAFG-------------------------------

--------------------LKSRSMGRLTC---------MA------------------------TYKVTF

LDGETGAENVV-ECSDEEYVLDAAERAG----MDLPYS--CRAGACSSCAGIIKA---------GEVDQSDQ

SFLDDSQIDDGFVLT------CVAYPASDCIILTHQEENM---------

>Sacu_v1.1_s0069.g016503/1-155

------------------------------------------------------------------------

--MAASTIILPSSFCSPCSLRPSSSLKASSPLPSTSTSPHP-------------------------------

--------------------RPRRMLPVAASKGGPGKGDHHK------------------------GFKVKF

LTPD-G-EKVV-NIPGDKYILDGGEEAG----LDLPYS--CRAGACSSCCAQIVE---------GTVDQSEQ

SFLDDDQIKEGFVLT------CVSYPTSDLVLTTHCEDKL---------

>Sacu_v1.1_s0059.g015062/1-131

------------------------------------------------------------------------

---MAAAAATAQTVCASTAIGAGLKRGVRLEKAGGAGELWGKVA----------------------------

--------------------GGKGVGGRVRM---------EA------------------------VYKVTF

QTQE-G-DMTI-DVPASTYLLDAAEEEG----LNLPYS--CRAGACSSCAGKLSS---------GTVDQSQQ

SFLDDAQISDGFVLT------CI--------------------------

>CHBRA25g00470/1-207

------------------------------------------------------------------------

---MSTSASVAAAATAAAPASSAGTILGGAGSAATSATQRRRDTSCRLGPGQPGHLVWVPVKCSAFANVGQQ

RAAVRRELGPRQPGRLVVRPSLRPDTGRSFGLSRSPSVTQCA------------------------VYQVTL

KTPD-G-DKII-DCMDDEYILDKAEELG----LILPSS--CRSGACSACAGKILA---------GTVDQGDQ

SFLTDSQLGSGYVLT------CVAYPTSDVVIETGKESEVGS-------

>CHBRA37g00180/1-155

------------------------------------------------------------------------

----MASTIAAAATTGAIAAASLGAIAKVSASPRSSVSLKMT------------------------------

--------------------PKIGRLNAARVFGVQPSLGRCQ------------------------AYKVTF

ITGK-D-EVVV-DCPEDVYVLDAAEEAG----LDLPYS--CRAGACSTCAGKLEK---------GTIDQSDQ

SFLDDDQVEAGFVLT------CVTYPTSDCTIVTNQEDKIY--------

>CHBRA52g00290/1-235

------------------------------------------MEVMEAMAAVPSWCVGNAAGARGQEFNTPE

KCLGKSLSTARTSVSPPSPSVGGTRFPPFSLSRASSPTLFPRFCLARASSLSTM--SSCFPPTTESKNWRCP

VLARRRMYGSVGSSGLGLGRVCTWKSFGLRAADVSGG---AT------------------------CYQVTF

KIPD-GETTTI-EVEEDEFILDKAEEMG----LDLPYS--CRAGSCSTCAAVLES---------GSVDQSNQ

SFLDDEQIEKGFVLI------CQAYPKSDIVLTTHKEEELY--------

>Azfi_s0092.g043125/1-166

------------------------------------------------------------------------

----MAAPLLLLSTSSPSLSLRSHSPASFSPAGLHNNASSSLLCLDRLP--ARS------------------

--------------------SVAITGIRASSDPKKGKPPQPR------------------------VYKVKF

LLPE-GAEPVLVDVPPNTYILDAAEEAG----LDLPYS--CRAGACSSCAGKVIK---------GSVDQADQ

SFLDDDQIGKGFLLT------CVSYATSDLTIKTHCEDEI---------

>Azfi_s0003.g007828/1-154

------------------------------------------------------------------------

----MATCTTSTSMVMGSLANPSSAPVLLGGLSSQRVSL---------------------------------

--------------------QKKSDSNSLKFGLSGSRMGRIQ------------------------AFKVTL

KVKDVG-ETVL-EVPADQYILDAAEEAG----LDLPYS--CRAGACSSCTGKVVS---------GTVDQSEQ

SFLDDDQVAEGFVLT------CCAFVTSDVVIETHQEDNLTL-------

>Azfi_s0393.g067829/1-234

------------------------------------------------------------------------

----MAAASSSSILCSINHTMPAPVSTPLLVPSPSSSSSFVSLACPSFLLHRAAHSHGANFAYCKVPRISLS

CNVTMRQYPAMGDVDM----LAATSGCRVSAIGKGSYGHVAAKRDSYRASAVGKGSSHEDLKKKKNTYTVKF

ITPE-GEETVV-EVPGDKYLLDAGEEAG----VDLPYS--CRAGACSSCAVRILNLKNANGEEEGTVDQSDQ

SFLDEEQIHKGFVLA------CVAYPTSDLVLQTHCEDQL---------

>Azfi_s0006.g009850/1-141

------------------------------------------------------------------------

----MASASAMSSILSHPPHQQQQRFHTNLTISNA-------------------------------------

--------------------RKSMVVRAMSG---------TQ------------------------TFKVTL

KTPQ-G-EKIL-NVPEDVYISDAAEEAG----LTLPIS--CRSGNCSSCAAILKS---------GEVDQSYQ

TFLDESRVAQGFVLT------CVAYPLSDVVIETHQESLLYDS------

**Beta amylase 3**

cov pid  **1** **[ . . . . : . . .** **80**

1 Selmo_145994/1-464 100.0% 100.0%  **--------------------------------------------------------------------------------**

2 Selmo_232533/1-472 98.9% 63.5%  **--------------------------------------------------------------------------------**

3 AT4G17090_BAM3/1-548 97.4% 50.4%  **------------------------------------------------------------------------------ME**

4 Bradi3g33730/1-548 97.8% 48.9%  **--------------------------------------------------------------------------------**

5 Cre01.g044100/1-1337 99.1% 16.2%  **--------------------------------------------------------------------------------**

6 Cre06.g270350/1-653 97.4% 27.4%  **--------------------------------------------------------------------------------**

7 Cre06.g307150/1-594 91.4% 36.2%  **--------------------------------------------------------------------------------**

8 Mapoly0043s0083/1-611 99.4% 50.7%  **--------------------------------------------------------------------------------**

9 Mapoly0043s0084/1-622 99.8% 50.9%  **--------------------------------------------------------------------------------**

10 Pp3c16_11260/1-612 99.8% 49.3%  **--------------------------------------------------------------------------------**

11 Pp3c25_2760/1-609 99.6% 49.1%  **--------------------------------------------------------------------------------**

12 Pp3c6_10130/1-611 99.6% 49.6%  **--------------------------------------------------------------------------------**

13 CHBRA1096g00040/1-868 98.9% 32.1%  **-----------------------------MDLADKKSDRVVSKLPTSRTPSSLRRSDCSATSIVYPESVLTGADKHHWER**

14 Azfi_s0206.g057798/1-543 93.3% 45.6%  **----------------------------------------------------------------------------MAFP**

15 Azfi_s0159.g053957/1-547 71.1% 24.5%  **-------------------------------MYICICIYICICIYVFMYVYIHMWYVCIYVLPCSSPQEKDPLVPLVFLP**

16 Sacu_v1.1_s0011.g005198/1-632 99.4% 48.0%  **--------------------------------------------------------------------------------**

17 Mapoly0008s0172/7-441 92.5% 50.5%  **--------------------------------------------------------------------------------**

18 AaBONN_Sc2ySwM_344.3169.1/1-621 99.8% 50.2%  **--------------------------------------------------------------------------------**

19 AaBONN_Sc2ySwM_362.2644.1/1-479 95.9% 51.7%  **--------------------------------------------------------------------------------**

cov pid  **81**  **. 1 . . . . : .** **160**

1 Selmo_145994/1-464 100.0% 100.0%  **--------------------------------------------------------------------------------**

2 Selmo_232533/1-472 98.9% 63.5%  **--------------------------------------------------------------------------------**

3 AT4G17090_BAM3/1-548 97.4% 50.4%  **LTLNSSSSLIKRKDAKSSRN---QESSSNNMTFAKMKPPTYQFQAKNSVK-----------EMKFTHEKTFTPEGETLEK**

4 Bradi3g33730/1-548 97.8% 48.9%  **-----MALTLRSSTSFLSPL---EPSSKLHKAEDAPPSCVAVPAAPSRLR-----------VLRAAAQAPLSPMEAPAPE**

5 Cre01.g044100/1-1337 99.1% 16.2%  **--MSSENILLDRALSVQGPG---YGYLHLGGVYGDCVATTALGYRAAAPS-----------MPASASVPSFGQSGVLRPH**

6 Cre06.g270350/1-653 97.4% 27.4%  **-MRASIDRQCCAAASSPASA---RCPILGLLSRGQKRLGWRLGGHIASSP-----------HAELTSPTPVAAGATARAA**

7 Cre06.g307150/1-594 91.4% 36.2%  **--MPTSLPQCPSAPRSVQPL---RSALLAAQRLSQCRGSRSQRGSGACHS-----------QGRQVTHPTAHPRTSRTLD**

8 Mapoly0043s0083/1-611 99.4% 50.7%  **---MAAACSLVGPSMFSSHS---RRVLVTEFSAVEVSSSSSVVDVPVRAR-----------CTFRSSSVGSGHGPCIQRA**

9 Mapoly0043s0084/1-622 99.8% 50.9%  **---MAAVYWLVGPSTFLGSS---RSVVAPESSAVEIPSSSNVVDGPVRVR-----------CVIQGSSLGSGPGQSTQRS**

10 Pp3c16_11260/1-612 99.8% 49.3%  **---MVVFTISHSVAWKSSFG---GCSVLKNASEPEVSSSTRDDHVRQPVT-----------------KLSPLVWGLGRQQ**

11 Pp3c25_2760/1-609 99.6% 49.1%  **----MSSAMCRSATLANRFV---CGNVLTDRLQSEASSSGRVELARFSAR-----------------RAVPGVSCSQRNQ**

12 Pp3c6_10130/1-611 99.6% 49.6%  **----MAHAMCGNYILTNSFI---GRSVLTDNSQSESSPSDSVEVMRTRVR-----------------KPASVVLCLQRQH**

13 CHBRA1096g00040/1-868 98.9% 32.1%  **LTSSSPCYSSSSSSSSSSYS---SYSAFSPSSSLSSPRSSSPLPARPPVR-----------STASSTLPPSCSSASPSSS**

14 Azfi_s0206.g057798/1-543 93.3% 45.6%  **SSTLPGSLHGSSPETVRNML---HTSTATSSSSSSSTTSTTNISSSSSSS-----------DQKHHRKSSSSPHSLIHNS**

15 Azfi_s0159.g053957/1-547 71.1% 24.5%  **FLFEKSENQTSIPSFLFLFL---FCFVYPDLRLKTTQNSPMASLSCSLSGSDALRGFLPDIVEKKHPKVVIDCAHFPNPN**

16 Sacu_v1.1_s0011.g005198/1-632 99.4% 48.0%  **MEKGAKSYAETGKNISPPLL---LSPCSVSFIHFLFLCQTSMAFPSSTLS-----------GSLHATHPDAFRGIIHGIP**

17 Mapoly0008s0172/7-441 92.5% 50.5%  **--------------------------------------------------------------------------------**

18 AaBONN_Sc2ySwM_344.3169.1/1-621 99.8% 50.2%  **---MAFACMSSSGSLFVRLS---GSGRSFPAELEPSSSIRDEACSPRCLA--------------AHFRPVPIQSLPRLPK**

19 AaBONN_Sc2ySwM_362.2644.1/1-479 95.9% 51.7%  **----------------------------------------------------------------MADFPDPAQQEEQQQS**

cov pid  **161**  **. . . 2 . . . .** **240**

1 Selmo_145994/1-464 100.0% 100.0%  **--------------------------------------------------------------------------------**

2 Selmo_232533/1-472 98.9% 63.5%  **--------------------------------------------------------------------------------**

3 AT4G17090_BAM3/1-548 97.4% 50.4%  **WEKLHVLSYPHSK-------------------------------------------------------------------**

4 Bradi3g33730/1-548 97.8% 48.9%  **LLHGQAQ-------------------------------------------------------------------------**

5 Cre01.g044100/1-1337 99.1% 16.2%  **PRRYSQNAFGTSL-------------------------------------------------------------------**

6 Cre06.g270350/1-653 97.4% 27.4%  **APTWRSTVDEEAV-------------------------------------------------------------------**

7 Cre06.g307150/1-594 91.4% 36.2%  **DDSGFASFAPEPIVTTPGASAFSE---V---------EDDLQIHRLLRETGQHIKIPMIDEDRFD---------------**

8 Mapoly0043s0083/1-611 99.4% 50.7%  **AKELDTCLSYSPFSSS-----------------------SPSADERWRSQGSRGSHLEALDMGMFGTAAEGERMVP----**

9 Mapoly0043s0084/1-622 99.8% 50.9%  **SKDFDTCLTASSFPSTSLSTSPPS---S-----------SPSADEWWRSQGSRGSHPDALDMRMFGIATEGQSKVA----**

10 Pp3c16_11260/1-612 99.8% 49.3%  **FDGYAAQSSAGSLSSASA---------------------SSFPEEWWRKNSNNVADSMGGLRATHHTQTVSMFPERRSIL**

11 Pp3c25_2760/1-609 99.6% 49.1%  **SDGHVAHTSSGSVSSFG----------------------SLSSEEWWRKNTGSAGDSTGGLRATHHTQMDTMFPEHKSLL**

12 Pp3c6_10130/1-611 99.6% 49.6%  **FDGHAAQSTSGPLSASSA---------------------SPFPDEWWRKHSSTSGVSMAELRVSQFAEMETMFPEHRSIL**

13 CHBRA1096g00040/1-868 98.9% 32.1%  **SSSSSSSSAPSPLFLSSPLVSPPS---TEEELWCRFQKMGYWPEVWMVDPSTKYSSKIRESSSPLPRMPPSRGPSSSSPS**

14 Azfi_s0206.g057798/1-543 93.3% 45.6%  **SSSSSQSPPPPSF-------------------------------------------------------------------**

15 Azfi_s0159.g053957/1-547 71.1% 24.5%  **ANPEPKNNNSEPNPKHNPIPSSSI---G---------SSPLLAQEWFKKPSAVLWPVSVETNHLFSFEESCVEKEEK---**

16 Sacu_v1.1_s0011.g005198/1-632 99.4% 48.0%  **EKQHAHHRRTSPFSHHGCIALNNS---S-----------GINVESFCSSPPSVHTTEDHTWRIPSYQPPSALFSPY----**

17 Mapoly0008s0172/7-441 92.5% 50.5%  **--------------------------------------------------------------------------------**

18 AaBONN_Sc2ySwM_344.3169.1/1-621 99.8% 50.2%  **EPDFSSSLTTSSISSSC----------------------SPFPEDWWRSPPQLQTARLTGSRVDVSELRVSRMSDTEGMI**

19 AaBONN_Sc2ySwM_362.2644.1/1-479 95.9% 51.7%  **KPG------------------------------------------WIRASGM----------------------------**

cov pid  **241**  **: . . . . 3 . .** **320**

1 Selmo_145994/1-464 100.0% 100.0%  **--------------------------------------------------------------------------------**

2 Selmo_232533/1-472 98.9% 63.5%  **--------------------------------------------------------------------------------**

3 AT4G17090_BAM3/1-548 97.4% 50.4%  **--------------------------------------------------------------------------------**

4 Bradi3g33730/1-548 97.8% 48.9%  **--------------------------------------------------------------------------------**

5 Cre01.g044100/1-1337 99.1% 16.2%  **--------------------------------------------------------------------------------**

6 Cre06.g270350/1-653 97.4% 27.4%  **--------------------------------------------------------------------------------**

7 Cre06.g307150/1-594 91.4% 36.2%  **--------------------------------------------------------------------------------**

8 Mapoly0043s0083/1-611 99.4% 50.7%  **--------------------------------------------------------------------------------**

9 Mapoly0043s0084/1-622 99.8% 50.9%  **--------------------------------------------------------------------------------**

10 Pp3c16_11260/1-612 99.8% 49.3%  **--------------------------------------------------------------------------------**

11 Pp3c25_2760/1-609 99.6% 49.1%  **--------------------------------------------------------------------------------**

12 Pp3c6_10130/1-611 99.6% 49.6%  **--------------------------------------------------------------------------------**

13 CHBRA1096g00040/1-868 98.9% 32.1%  **SSPPSSPSSSPPSSPLPSAPSSPASNVSLSPPSSRRPSSLSACTGNSKRRTCPAAPASAMSVGIAARPMEERTEDDRQQM**

14 Azfi_s0206.g057798/1-543 93.3% 45.6%  **--------------------------------------------------------------------------------**

15 Azfi_s0159.g053957/1-547 71.1% 24.5%  **--------------------------------------------------------------------------------**

16 Sacu_v1.1_s0011.g005198/1-632 99.4% 48.0%  **-SHKLF--------------------------------------------------------------------------**

17 Mapoly0008s0172/7-441 92.5% 50.5%  **--------------------------------------------------------------------------------**

18 AaBONN_Sc2ySwM_344.3169.1/1-621 99.8% 50.2%  **SGHTAS--------------------------------------------------------------------------**

19 AaBONN_Sc2ySwM_362.2644.1/1-479 95.9% 51.7%  **--------------------------------------------------------------------------------**

cov pid  **321**  **. . : . . . . 4** **400**

1 Selmo_145994/1-464 100.0% 100.0%  **--------------------------------------------------------------------------------**

2 Selmo_232533/1-472 98.9% 63.5%  **--------------------------------------------------------------------------------**

3 AT4G17090_BAM3/1-548 97.4% 50.4%  **--------------------------------------------------------------------------------**

4 Bradi3g33730/1-548 97.8% 48.9%  **--------------------------------------------------------------------------------**

5 Cre01.g044100/1-1337 99.1% 16.2%  **--------------------------------------------------------------------------------**

6 Cre06.g270350/1-653 97.4% 27.4%  **--------------------------------------------------------------------------------**

7 Cre06.g307150/1-594 91.4% 36.2%  **--------------------------------------------------------------------------------**

8 Mapoly0043s0083/1-611 99.4% 50.7%  **--------------------------------------------------------------------------------**

9 Mapoly0043s0084/1-622 99.8% 50.9%  **--------------------------------------------------------------------------------**

10 Pp3c16_11260/1-612 99.8% 49.3%  **--------------------------------------------------------------------------------**

11 Pp3c25_2760/1-609 99.6% 49.1%  **--------------------------------------------------------------------------------**

12 Pp3c6_10130/1-611 99.6% 49.6%  **--------------------------------------------------------------------------------**

13 CHBRA1096g00040/1-868 98.9% 32.1%  **GDDVTAEELLSHVLPAGSRDTCCGGGAAAAAEVDGVLGSRSRQKSLPRSSSSSSSRAGRRTGIVMLPVLTEERQQERREQ**

14 Azfi_s0206.g057798/1-543 93.3% 45.6%  **--------------------------------------------------------------------------------**

15 Azfi_s0159.g053957/1-547 71.1% 24.5%  **--------------------------------------------------------------------------------**

16 Sacu_v1.1_s0011.g005198/1-632 99.4% 48.0%  **--------------------------------------------------------------------------------**

17 Mapoly0008s0172/7-441 92.5% 50.5%  **--------------------------------------------------------------------------------**

18 AaBONN_Sc2ySwM_344.3169.1/1-621 99.8% 50.2%  **--------------------------------------------------------------------------------**

19 AaBONN_Sc2ySwM_362.2644.1/1-479 95.9% 51.7%  **--------------------------------------------------------------------------------**

cov pid  **401**  **. . . . : . . .** **480**

1 Selmo_145994/1-464 100.0% 100.0%  **------------------------------------------------MLPLDS---INSGNN---QVNRARAMNASFQA**

2 Selmo_232533/1-472 98.9% 63.5%  **------------------------------------------------MLPLDS---VNV-NN---TLNRRRALNAGLIA**

3 AT4G17090_BAM3/1-548 97.4% 50.4%  **---------------------------------------NDASVPVFVMLPLDT---VTM-SG---HLNKPRAMNASLMA**

4 Bradi3g33730/1-548 97.8% 48.9%  **--------------------------------QAHSGGQKRGGVPVYVMLPLDT---VGP-GG---QLLRARALAASLMA**

5 Cre01.g044100/1-1337 99.1% 16.2%  **-------------------------------SPVHGPSDGGTGANVYCMLPLDT---VNS-EGVF-RYAGSAWFAQALQL**

6 Cre06.g270350/1-653 97.4% 27.4%  **-------------------------------------------LPLYTCLDGDI---LTP-AN---KFAYYEALRSGLKA**

7 Cre06.g307150/1-594 91.4% 36.2%  **--------------------------TQYPESQAEEPSSSGSGCPVYVMLPLDTVWVVERDGKRISVLKKERSLDIALHT**

8 Mapoly0043s0083/1-611 99.4% 50.7%  **--------------GLEVGAGSW---LEHHSEETSSSRGTSGGVPVFVMLPLDS---VKM-SN---VVNRKRAMNASLLA**

9 Mapoly0043s0084/1-622 99.8% 50.9%  **--------------DWELGSTPW---LEHHLEETSTSRGTSGGVPVFVMLPLDS---VNM-NN---TVNRKRAMNAGLLA**

10 Pp3c16_11260/1-612 99.8% 49.3%  **--------------DWDNTSDKW---KEHAFHETATSRGVHGRVPVFVMLPLDS---VNM-NN---TLNRRRALNASLMA**

11 Pp3c25_2760/1-609 99.6% 49.1%  **--------------DWDNTADEW---KEHAFHETPTSRGVHGGVPVFVMLPLDS---VNI-NN---TLKRRRALNASLLA**

12 Pp3c6_10130/1-611 99.6% 49.6%  **--------------DWQNTAEEW---IEHSTHETPTSRGVCGGVPVFVMLPLDS---VNM-NN---TLNRRRAMNASLLA**

13 CHBRA1096g00040/1-868 98.9% 32.1%  **RSPGEVGAMRERERERGRGRDAWRI-HDVWDHASPGGRGAGNGVPVYIMLPLDT---VNM-NN---TLTRPRALRASLTA**

14 Azfi_s0206.g057798/1-543 93.3% 45.6%  **-------------------------LSSITNSYNNIQSSAVLWGPINLV-EEKL---FDFHDS---PVFSKNSISESLVD**

15 Azfi_s0159.g053957/1-547 71.1% 24.5%  **--------------------------HMYIEHGLKGYDDGDGVIPVYVMLPLDS---INPKSG---TVKRPKAMSASMRA**

16 Sacu_v1.1_s0011.g005198/1-632 99.4% 48.0%  **--------------DFHESSDHAPLEREHVYVDTPTSIGPNGGVPVYVMMPLDT---VNPHNN---TLNRRRAMDASLRA**

17 Mapoly0008s0172/7-441 92.5% 50.5%  **------------------------------------------GVPVYVMMPLDS---AEL-VK---SESGQWMLSLCLKR**

18 AaBONN_Sc2ySwM_344.3169.1/1-621 99.8% 50.2%  **--------------EWQRTSHDW---KEFAFHETPTSRGTTGGVPVYVMLPLDS---VNM-NN---TLNRRRALNASLLA**

19 AaBONN_Sc2ySwM_362.2644.1/1-479 95.9% 51.7%  **-----------------------------------------AGVPVFVMLPLDS---VQM-LE---SGSGRWALSASLVA**

cov pid  **481**  **. 5 . . . . : .** **560**

1 Selmo_145994/1-464 100.0% 100.0%  **LK-SAGVEGVMVDVWWGIVEKDGPCNYNWSGYRELLEMAKKHGLKVQAVMSFHQCGGN-VGDSAFIPLPWWVVEEAKNNP**

2 Selmo_232533/1-472 98.9% 63.5%  **LK-SAGVEGVMVDVWWGIVEREKPHHYKWSAYKELVSLIQKNGLKIQVVMSFHQCGGN-VGDSCYIPLPLWVLEEVQNNP**

3 AT4G17090_BAM3/1-548 97.4% 50.4%  **LK-GAGVEGVMVDAWWGLVEKDGPMNYNWEGYAELIQMVQKHGLKLQVVMSFHQCGGN-VGDSCSIPLPPWVLEEISKNP**

4 Bradi3g33730/1-548 97.8% 48.9%  **LR-SAGVEGVMVDVWWGVVEREGPGRYDWEGYAELVRMVERAGLRLQMVMSFHQCGGN-VGDSCNIPLPSWVLEEVSANP**

5 Cre01.g044100/1-1337 99.1% 16.2%  **LV-ASGAHGVAMDFWWGAVER-SPGQYNWSGYKQALEVIKQTGLKVQVVLSFHACGGN-VGDTVQIPLPDWVVQCAEADP**

6 Cre06.g270350/1-653 97.4% 27.4%  **LR-ALGINGISVDVYWGIVEGAAPMEYDWSSYKQLFALIRDEGFMAQVCLCFH--GTE------AVPLPAWVLAAGAANP**

7 Cre06.g307150/1-594 91.4% 36.2%  **LK-QAGVEGVMVDVWWGIVERAGPRQYDFSAYKRLFYKVAAAGLKVQAVMSFHAAGGN-VGDTCKIPLPKWVLEIGERNP**

8 Mapoly0043s0083/1-611 99.4% 50.7%  **LK-SAGVEGIMMDVWWGIVEKDGPMKYNWSAYLELITMARKHGLKVQCVMSFHQCGGN-VGDTCTIPLPPWVVEEIKSNN**

9 Mapoly0043s0084/1-622 99.8% 50.9%  **LK-SAGVEGIMMDVWWGIVEKDGPMKYNWSGYIELINMARKHGLKVQCVMSFHQCGGN-VGDSCNIPLPPWVVEEIKSNN**

10 Pp3c16_11260/1-612 99.8% 49.3%  **LK-SAGIEGIMMDVWWGIVEKDAPLNYNWSAYRELIEMARKHGLKVQAVMSFHQCGGN-VGDSCNIPLPPWVLEEIQKNP**

11 Pp3c25_2760/1-609 99.6% 49.1%  **LK-SAGVEGVMMDVWWGIVEKEGPRNYNWSAYRELIDMVRKHGLKVQAVMSFHQCGGN-VGDSCNIPLPPWVLEEVQKNP**

12 Pp3c6_10130/1-611 99.6% 49.6%  **LK-SAGVEGIMMDVWWGIVEKDGPHQYNWSAYRELIDMVRNHGLKVQAVMSFHQCGGN-VGDSCNVPLPPWVLEEVRKNP**

13 CHBRA1096g00040/1-868 98.9% 32.1%  **LK-SAGVEGVMVDVWWGIVEGQEPHKYRWTAYQDLVKMVHDAGLKMQVVMSFHQCGGN-VGDSCLIPLPQWVLQEIEKNP**

14 Azfi_s0206.g057798/1-543 93.3% 45.6%  **LN-LTPL------------------------HKELIIML------IIVAKEDIQCGSI-RCAWCRIPLPPWVKEEIDQNP**

15 Azfi_s0159.g053957/1-547 71.1% 24.5%  **VQ-MAGVKGIVMEVWWGIVEKEGPQNYNWCGYKELLDMAKQWGLKVQVILCFHQCWNIAGGDNCLIPLPPWVREEIEHNP**

16 Sacu_v1.1_s0011.g005198/1-632 99.4% 48.0%  **LK-SAGVEGIMVDVWWGAVEKN-PQMYNWSGYRELIAMAKAYGLKMQTVMSFHQCGGN-VGDNCWIPLPPWVKEEIDHNP**

17 Mapoly0008s0172/7-441 92.5% 50.5%  **LK-DAGVEGVMMDVWWGLVEKTAPGVYDWSLYDRLVKVIQDNDLKLQAVMSFHQCGGN-IGDGVYIPLPDWVLEESHRNP**

18 AaBONN_Sc2ySwM_344.3169.1/1-621 99.8% 50.2%  **LK-SAGVEGIMMDVWWGIVEKDVPGQYNWSAYKELVDLVRKYGLKVQAVMSFHQCGGN-VGDSCFIPLPQWVTDEIHQNP**

19 AaBONN_Sc2ySwM_362.2644.1/1-479 95.9% 51.7%  **LQ-GAGVKGVMMDVWWGIVEKDVPGDYDWSGYAKLIELVRFCGLKIQAVMSFHQCGSN-VGDSCSIPLPSWVLEEVDRNA**

cov pid  **561**  **. . . 6 . . . .** **640**

1 Selmo_145994/1-464 100.0% 100.0%  **DMVYTDRY-----GNRNFEYLSLGCDHLP-VLKGRTPVQAYSDFMRSFKESFSDML-GDVIVEIQVGMGPAGELRYPGYP**

2 Selmo_232533/1-472 98.9% 63.5%  **NIVYTDKS-----GNRNHEYLSLGCDFLP-VLRGRTPIQAYSDFMRSFKHVFKDVL-GETIVEVQVGLGPAGELRYPAYP**

3 AT4G17090_BAM3/1-548 97.4% 50.4%  **DLVYTDKS-----GRRNPEYISLGCDSVP-VLRGRTPIQVYSDFMRSFRERFEGYI-GGVIAEIQVGMGPCGELRYPSYP**

4 Bradi3g33730/1-548 97.8% 48.9%  **DIVYTDRS-----GRRNPEYISLGCDTLP-VLKGRTPVQVYSDFMRSFRDRFSGYL-GTVIAEIQVGLGPCGELRYPSYP**

5 Cre01.g044100/1-1337 99.1% 16.2%  **DLFFADRPRNGGLGNRNREYLSIWADDAPGVLRGRSPMQCYEEYMVSLRENFSQEL-GTVIDEVVVGAGPCGELRLPSYV**

6 Cre06.g270350/1-653 97.4% 27.4%  **DIYFTDRA-----GVRNTHCISLGVDEVP-ALDGRTALACYRDLMTSFRVELEPLL-GSTIVDVCVGLGPDGELKYPAHP**

7 Cre06.g307150/1-594 91.4% 36.2%  **DIFYTDKA-----GHRNRECLSLGCDEVP-LFWGRTPVLMYRDFINAFADKFQHLF-GTVITEVTVGLGPAGELRYPSYP**

8 Mapoly0043s0083/1-611 99.4% 50.7%  **DLVYTDKN-----GNRNFEYLSLGCDSLP-VLKGRTQVQVYSDFMRSFRETFKDLL-GNTVIEIQVGMGPAGELRYPGYP**

9 Mapoly0043s0084/1-622 99.8% 50.9%  **DLVYTDKY-----GNRNYEYLSLGCDSLP-VLKGRTPVQVYSDFMRSFRDTFKDLL-GDTVIEIQVGMGPAGELRYPGYP**

10 Pp3c16_11260/1-612 99.8% 49.3%  **DLAYTDKS-----GRRNAEYICLGADNVP-ALKGRTPVQCYADFMRSFRDNFEDLL-GDVIIEIQCGMGPAGELRYPSYP**

11 Pp3c25_2760/1-609 99.6% 49.1%  **DLAYTDKA-----GKRNAEYISLGADNVP-ALKGRTPVQCYADFMRSFRDNFKDLL-GDVIIEIQCGMGPAGELRYPSYP**

12 Pp3c6_10130/1-611 99.6% 49.6%  **DLAYTDRV-----GRRNAEYISLGADNVP-ALQGRTPVQCYADFMRSFRDNFKDLL-GDVIIEIQCGMGPAGELRYPSYP**

13 CHBRA1096g00040/1-868 98.9% 32.1%  **DIVYTDKA-----GKRNLEYLSLGCDSLP-VLRGRSPVQAYADFMRSFRNVFASYL-GNVISEIQVGLGPAGELRYPSYP**

14 Azfi_s0206.g057798/1-543 93.3% 45.6%  **DLVYTDKD-----GRRNYEYLSLGCDSLP-VLKGRSPIQVYSDFMRDFRDEFSEYL-GEVIVEIQVGMGPAGELRYPAYP**

15 Azfi_s0159.g053957/1-547 71.1% 24.5%  **DILFTNKR-----GMKSYDYISFGCDTLP-ILQGRSPVQVYADFMRGFRDEFKDYI-GEVLVEIQVGLGPGGELQYPSCL**

16 Sacu_v1.1_s0011.g005198/1-632 99.4% 48.0%  **DLVYTDKD-----GRRNYEYLSLGCDMLP-VLKGRSPVQVYSDFMRSFRDEFNDYL-GKVIVEIQVGLGPAGELRYPAYP**

17 Mapoly0008s0172/7-441 92.5% 50.5%  **DIFYTDQ------GWRNPEYISLGCDTLP-VIRGRTPVQCYSDYMTSFRENYKSVL-GDVIVEIQVGMGPAGELRYPAYP**

18 AaBONN_Sc2ySwM_344.3169.1/1-621 99.8% 50.2%  **DIVYTDKS-----SRRNFEYLSLGCDVLP-VLRGRSPVQAYADFMRSFRDTFEPLL-GEVINEIQVGMGPAGELRYPAYP**

19 AaBONN_Sc2ySwM_362.2644.1/1-479 95.9% 51.7%  **EIVFTDKS-----QNRNYEYISLGCDLLP-VLRGRSPVEVYADYMTSFRDTFVELI-GDVIVEIQVGMGPAGELRYPSYP**

cov pid  **641**  **: . . . . 7 . .** **720**

1 Selmo_145994/1-464 100.0% 100.0%  **ERDGIWKFPGVGEFQCHDNYMLASLKASAESIGKPDWG-CAPSDAGHYNQWPEDSIFFKRD-GGWNTDYGRFFLEWYSGK**

2 Selmo_232533/1-472 98.9% 63.5%  **EYNGKWRFPGIGEFQCYDKYMLASLRACATACGTKHWGQGGPHDAGHYNQWPDDTGFFNRD-GSWNSPYGQFFLEWYSGM**

3 AT4G17090_BAM3/1-548 97.4% 50.4%  **ESNGTWRFPGIGEFQCYDKYMKSSLQAYAESIGKTNWGTSGPHDAGEYKNLPEDTEFFRRD-GTWNSEYGKFFMEWYSGK**

4 Bradi3g33730/1-548 97.8% 48.9%  **EANGTWSFPGIGEFQCYDKYMRASLQAAAAAAGHENWGTNGPHDAGEYKQFPEETGFFRWD-GTWSTEYGSFFLEWYSGM**

5 Cre01.g044100/1-1337 99.1% 16.2%  **EANG-WRFPGAGEFQCYDRRALASLAQAAREAGHPEWGYTGPHDAGEYNSTPEHTGFFSHN-GSWNTPYGRFFLEWYSGC**

6 Cre06.g270350/1-653 97.4% 27.4%  **-RDRRWNFPGIGEFQCYDKYMLAGLRACSHQVSQPSWGLGGPHDAGAYTVWPQQTGFFNQY-GNWSSPYGKFFLQWYSDM**

7 Cre06.g307150/1-594 91.4% 36.2%  **EGDGRWRFPGVGEFQCYDKFMLESLRRTAEAAGHAEWGLSGPHDAGHYNSSSWETGFFVSQNGSWNTAYGHFFLSWYSNM**

8 Mapoly0043s0083/1-611 99.4% 50.7%  **EQNGRWRFPGIGEFQCYDKYMIASLKSAAEAIGMPAWGHAGPNDAGDYNRWPEEVAFFSRD-GGWKSAYGEFFLDWYSKM**

9 Mapoly0043s0084/1-622 99.8% 50.9%  **EQNGRWRFPGIGEFQCYDKYMIASLKSAAEAIGKPAWGHGGPSDAGDYNRWPDETAFFSRD-GGWKSAYGEFFLDWYSKM**

10 Pp3c16_11260/1-612 99.8% 49.3%  **ESEGRWRFPGIGEFQCYDKYMLAGLKASAEAVGMPAWGTSGPHDAGNYNQWPDDTGFFRKD-GTWSTDYGQFFMEWYSEM**

11 Pp3c25_2760/1-609 99.6% 49.1%  **ESEGRWRFPGIGEFQCYDKYMLASLKANAQALGKPAWGHGGPCDAGNYNQWPDETGFFHRD-GSWCSEYGQFFMEWYSEM**

12 Pp3c6_10130/1-611 99.6% 49.6%  **ESEGRWRFPGIGEFQSYDKYMIASLKASAHAVGKPAWGSGGPHDSGSYNQWPEETGFFKKD-GTWSTEYGQFFMEWYSEM**

13 CHBRA1096g00040/1-868 98.9% 32.1%  **ESYSRWRFPGIGEFQCYDKHMLANLKAAAEAIGKPRWGLGGPHDAGSYNQWPEETGFFQRD-GSWKSQYGHFFLQWYSEM**

14 Azfi_s0206.g057798/1-543 93.3% 45.6%  **ESNNTWQFPGIGEFQCYDKYMLANLQASADSISQPGWGAGGPHDAGHYKQYPEETGFFQRE-GAWDTPYGKFFLEWYSGM**

15 Azfi_s0159.g053957/1-547 71.1% 24.5%  **DTSHISEFPSFEEFMCYDK----------------------------------------------------FFLEWYSEQ**

16 Sacu_v1.1_s0011.g005198/1-632 99.4% 48.0%  **ERNNTWQFPGIGEFECYDKYMLANLQAYAVARGKSEWGNGGPHDAGHYKQYPEETGFFQNG-GGWKSPYGVFFLEWYSGM**

17 Mapoly0008s0172/7-441 92.5% 50.5%  **QSENQWTFPGIGELQCYDKYMVTSLQAKATSVGKPQYG-KIPTDAGTYNQLPQDTGFWKDN-GTWKTAYGQFFLSWYSDQ**

18 AaBONN_Sc2ySwM_344.3169.1/1-621 99.8% 50.2%  **ESNGRWRFPGIGEFQCYDKYMMASLRACAEAVGRSHWGLGGPHDAGNYNQWPDETGFFHRD-GSWNSPYGQFFLEWYSEM**

19 AaBONN_Sc2ySwM_362.2644.1/1-479 95.9% 51.7%  **ECNGLWRFPGIGEFQCYDKYMLEDLKTRAISIGKDDWGVGGPAGTGAYNQLPSETEFFQKN-GTWSTPYGHFFLEWYSDL**

cov pid  **721**  **. . : . . . . 8** **800**

1 Selmo_145994/1-464 100.0% 100.0%  **LIEHGESVLTAAEGIF------------------R-----------------G---------------------------**

2 Selmo_232533/1-472 98.9% 63.5%  **LISHGERVLSAAEAVF------------------R-----------------G---------------------------**

3 AT4G17090_BAM3/1-548 97.4% 50.4%  **LLEHGDQLLSSAKGIF------------------Q-----------------G---------------------------**

4 Bradi3g33730/1-548 97.8% 48.9%  **LLEHGDRVLAAAEAVF------------------G-----------------G---------------------------**

5 Cre01.g044100/1-1337 99.1% 16.2%  **LLKHGDRLLTVANAVF------------------ASKLNPPPQPPQSLLQTLGAAGSALAAQLQQVAQQTVGSLGHGSSG**

6 Cre06.g270350/1-653 97.4% 27.4%  **LMQHADSVLGIARDVLLPADGSLSFSGSSAGFGSSSSSSSVRPGFYGNGYGNG---------------------------**

7 Cre06.g307150/1-594 91.4% 36.2%  **LLEHADRVLSSAAEVL------------NKHGRPRVFNSMRDASNGHVIYEFT---------------------------**

8 Mapoly0043s0083/1-611 99.4% 50.7%  **LINHGEKILSAAAGIF------------------R-----------------G---------------------------**

9 Mapoly0043s0084/1-622 99.8% 50.9%  **LINHGEKILSAAAGIF------------------R-----------------G---------------------------**

10 Pp3c16_11260/1-612 99.8% 49.3%  **LLAHGERILSVATGIF------------------R-----------------D---------------------------**

11 Pp3c25_2760/1-609 99.6% 49.1%  **ILAHGERLLASASGIF------------------K-----------------G---------------------------**

12 Pp3c6_10130/1-611 99.6% 49.6%  **LLAHGERILSEATGIF------------------R-----------------G---------------------------**

13 CHBRA1096g00040/1-868 98.9% 32.1%  **LLRHGDLILSSAAGIF------------------R-----------------G---------------------------**

14 Azfi_s0206.g057798/1-543 93.3% 45.6%  **LIEHGEKIVSSAEAIF------------------R-----------------G---------------------------**

15 Azfi_s0159.g053957/1-547 71.1% 24.5%  **LLKHGERVLELAEAIF------------------R-----------------G---------------------------**

16 Sacu_v1.1_s0011.g005198/1-632 99.4% 48.0%  **LLEHGERVLRSAEAIF------------------R-----------------G---------------------------**

17 Mapoly0008s0172/7-441 92.5% 50.5%  **LLAHGERILTVANRVF----------------------------------------------------------------**

18 AaBONN_Sc2ySwM_344.3169.1/1-621 99.8% 50.2%  **LIAHGERILAAAEGIF------------------R-----------------G---------------------------**

19 AaBONN_Sc2ySwM_362.2644.1/1-479 95.9% 51.7%  **LLAHGERLLSAAHKIF------------------Q-----------------S---------------------------**

cov pid  **801**  **. . . . : . . .** **880**

1 Selmo_145994/1-464 100.0% 100.0%  **--------------------------------------------------------------------------------**

2 Selmo_232533/1-472 98.9% 63.5%  **--------------------------------------------------------------------------------**

3 AT4G17090_BAM3/1-548 97.4% 50.4%  **--------------------------------------------------------------------------------**

4 Bradi3g33730/1-548 97.8% 48.9%  **--------------------------------------------------------------------------------**

5 Cre01.g044100/1-1337 99.1% 16.2%  **GNAMGNGAGSGPSSSHGVQAFLSPMAPGGMNSYVTNTPFQPMSFMGLHTSQTSSGGPRMSTHSLNRILETGGSCGSRPGS**

6 Cre06.g270350/1-653 97.4% 27.4%  **--------------------------------------------------------------------------------**

7 Cre06.g307150/1-594 91.4% 36.2%  **--------------------------------------------------------------------------------**

8 Mapoly0043s0083/1-611 99.4% 50.7%  **--------------------------------------------------------------------------------**

9 Mapoly0043s0084/1-622 99.8% 50.9%  **--------------------------------------------------------------------------------**

10 Pp3c16_11260/1-612 99.8% 49.3%  **--------------------------------------------------------------------------------**

11 Pp3c25_2760/1-609 99.6% 49.1%  **--------------------------------------------------------------------------------**

12 Pp3c6_10130/1-611 99.6% 49.6%  **--------------------------------------------------------------------------------**

13 CHBRA1096g00040/1-868 98.9% 32.1%  **--------------------------------------------------------------------------------**

14 Azfi_s0206.g057798/1-543 93.3% 45.6%  **--------------------------------------------------------------------------------**

15 Azfi_s0159.g053957/1-547 71.1% 24.5%  **--------------------------------------------------------------------------------**

16 Sacu_v1.1_s0011.g005198/1-632 99.4% 48.0%  **--------------------------------------------------------------------------------**

17 Mapoly0008s0172/7-441 92.5% 50.5%  **--------------------------------------------------------------------------------**

18 AaBONN_Sc2ySwM_344.3169.1/1-621 99.8% 50.2%  **--------------------------------------------------------------------------------**

19 AaBONN_Sc2ySwM_362.2644.1/1-479 95.9% 51.7%  **--------------------------------------------------------------------------------**

cov pid  **881**  **. 9 . . . . : .** **960**

1 Selmo_145994/1-464 100.0% 100.0%  **--------------------------------------------------------------------------------**

2 Selmo_232533/1-472 98.9% 63.5%  **--------------------------------------------------------------------------------**

3 AT4G17090_BAM3/1-548 97.4% 50.4%  **--------------------------------------------------------------------------------**

4 Bradi3g33730/1-548 97.8% 48.9%  **--------------------------------------------------------------------------------**

5 Cre01.g044100/1-1337 99.1% 16.2%  **GHASGNELTGSASADGGGMPPPPAPPLPRRQATEGMLSSCGVPSSISPCCTYSSMSTYTGTSSGGASGSTANMIMATAGS**

6 Cre06.g270350/1-653 97.4% 27.4%  **--------------------------------------------------------------------------------**

7 Cre06.g307150/1-594 91.4% 36.2%  **--------------------------------------------------------------------------------**

8 Mapoly0043s0083/1-611 99.4% 50.7%  **--------------------------------------------------------------------------------**

9 Mapoly0043s0084/1-622 99.8% 50.9%  **--------------------------------------------------------------------------------**

10 Pp3c16_11260/1-612 99.8% 49.3%  **--------------------------------------------------------------------------------**

11 Pp3c25_2760/1-609 99.6% 49.1%  **--------------------------------------------------------------------------------**

12 Pp3c6_10130/1-611 99.6% 49.6%  **--------------------------------------------------------------------------------**

13 CHBRA1096g00040/1-868 98.9% 32.1%  **--------------------------------------------------------------------------------**

14 Azfi_s0206.g057798/1-543 93.3% 45.6%  **--------------------------------------------------------------------------------**

15 Azfi_s0159.g053957/1-547 71.1% 24.5%  **--------------------------------------------------------------------------------**

16 Sacu_v1.1_s0011.g005198/1-632 99.4% 48.0%  **--------------------------------------------------------------------------------**

17 Mapoly0008s0172/7-441 92.5% 50.5%  **--------------------------------------------------------------------------------**

18 AaBONN_Sc2ySwM_344.3169.1/1-621 99.8% 50.2%  **--------------------------------------------------------------------------------**

19 AaBONN_Sc2ySwM_362.2644.1/1-479 95.9% 51.7%  **--------------------------------------------------------------------------------**

cov pid  **961**  **. . . 0 . . . .** **1040**

1 Selmo_145994/1-464 100.0% 100.0%  **--------------------------------------------------------------------------------**

2 Selmo_232533/1-472 98.9% 63.5%  **--------------------------------------------------------------------------------**

3 AT4G17090_BAM3/1-548 97.4% 50.4%  **--------------------------------------------------------------------------------**

4 Bradi3g33730/1-548 97.8% 48.9%  **--------------------------------------------------------------------------------**

5 Cre01.g044100/1-1337 99.1% 16.2%  **SGSLMSPRSMSVFHGTACGAPHSEAHGAVGAAAAAGTVLGGRRSSGGGAAGSPQNASGVFSAAMNAMVTRGVATAAALMQ**

6 Cre06.g270350/1-653 97.4% 27.4%  **--------------------------------------------------------------------------------**

7 Cre06.g307150/1-594 91.4% 36.2%  **--------------------------------------------------------------------------------**

8 Mapoly0043s0083/1-611 99.4% 50.7%  **--------------------------------------------------------------------------------**

9 Mapoly0043s0084/1-622 99.8% 50.9%  **--------------------------------------------------------------------------------**

10 Pp3c16_11260/1-612 99.8% 49.3%  **--------------------------------------------------------------------------------**

11 Pp3c25_2760/1-609 99.6% 49.1%  **--------------------------------------------------------------------------------**

12 Pp3c6_10130/1-611 99.6% 49.6%  **--------------------------------------------------------------------------------**

13 CHBRA1096g00040/1-868 98.9% 32.1%  **--------------------------------------------------------------------------------**

14 Azfi_s0206.g057798/1-543 93.3% 45.6%  **--------------------------------------------------------------------------------**

15 Azfi_s0159.g053957/1-547 71.1% 24.5%  **--------------------------------------------------------------------------------**

16 Sacu_v1.1_s0011.g005198/1-632 99.4% 48.0%  **--------------------------------------------------------------------------------**

17 Mapoly0008s0172/7-441 92.5% 50.5%  **--------------------------------------------------------------------------------**

18 AaBONN_Sc2ySwM_344.3169.1/1-621 99.8% 50.2%  **--------------------------------------------------------------------------------**

19 AaBONN_Sc2ySwM_362.2644.1/1-479 95.9% 51.7%  **--------------------------------------------------------------------------------**

cov pid **1041**  **: . . . . 1 . .** **1120**

1 Selmo_145994/1-464 100.0% 100.0%  **--------------------------------------------------------------------------------**

2 Selmo_232533/1-472 98.9% 63.5%  **--------------------------------------------------------------------------------**

3 AT4G17090_BAM3/1-548 97.4% 50.4%  **--------------------------------------------------------------------------------**

4 Bradi3g33730/1-548 97.8% 48.9%  **--------------------------------------------------------------------------------**

5 Cre01.g044100/1-1337 99.1% 16.2%  **AAAAQGGAATSMAATNARAVAAACGSSSRTSSDSAAATNVGTTGAAALASGDATMIDVEPSDNSHTSTVAELMNAQGLSG**

6 Cre06.g270350/1-653 97.4% 27.4%  **--------------------------------------------------------------------------------**

7 Cre06.g307150/1-594 91.4% 36.2%  **--------------------------------------------------------------------------------**

8 Mapoly0043s0083/1-611 99.4% 50.7%  **--------------------------------------------------------------------------------**

9 Mapoly0043s0084/1-622 99.8% 50.9%  **--------------------------------------------------------------------------------**

10 Pp3c16_11260/1-612 99.8% 49.3%  **--------------------------------------------------------------------------------**

11 Pp3c25_2760/1-609 99.6% 49.1%  **--------------------------------------------------------------------------------**

12 Pp3c6_10130/1-611 99.6% 49.6%  **--------------------------------------------------------------------------------**

13 CHBRA1096g00040/1-868 98.9% 32.1%  **--------------------------------------------------------------------------------**

14 Azfi_s0206.g057798/1-543 93.3% 45.6%  **--------------------------------------------------------------------------------**

15 Azfi_s0159.g053957/1-547 71.1% 24.5%  **--------------------------------------------------------------------------------**

16 Sacu_v1.1_s0011.g005198/1-632 99.4% 48.0%  **--------------------------------------------------------------------------------**

17 Mapoly0008s0172/7-441 92.5% 50.5%  **--------------------------------------------------------------------------------**

18 AaBONN_Sc2ySwM_344.3169.1/1-621 99.8% 50.2%  **--------------------------------------------------------------------------------**

19 AaBONN_Sc2ySwM_362.2644.1/1-479 95.9% 51.7%  **--------------------------------------------------------------------------------**

cov pid **1121**  **. . : . . . . 2** **1200**

1 Selmo_145994/1-464 100.0% 100.0%  **--------------------------------------------------------------------------------**

2 Selmo_232533/1-472 98.9% 63.5%  **--------------------------------------------------------------------------------**

3 AT4G17090_BAM3/1-548 97.4% 50.4%  **--------------------------------------------------------------------------------**

4 Bradi3g33730/1-548 97.8% 48.9%  **--------------------------------------------------------------------------------**

5 Cre01.g044100/1-1337 99.1% 16.2%  **SSNASGPLAHPLNAAAQPGTLAPTAVTAAASGAIVAAPGAGHASCHPSPLSGVRFRSSASTLADAEPMFGSGQGTAFAAV**

6 Cre06.g270350/1-653 97.4% 27.4%  **--------------------------------------------------------------------------------**

7 Cre06.g307150/1-594 91.4% 36.2%  **--------------------------------------------------------------------------------**

8 Mapoly0043s0083/1-611 99.4% 50.7%  **--------------------------------------------------------------------------------**

9 Mapoly0043s0084/1-622 99.8% 50.9%  **--------------------------------------------------------------------------------**

10 Pp3c16_11260/1-612 99.8% 49.3%  **--------------------------------------------------------------------------------**

11 Pp3c25_2760/1-609 99.6% 49.1%  **--------------------------------------------------------------------------------**

12 Pp3c6_10130/1-611 99.6% 49.6%  **--------------------------------------------------------------------------------**

13 CHBRA1096g00040/1-868 98.9% 32.1%  **--------------------------------------------------------------------------------**

14 Azfi_s0206.g057798/1-543 93.3% 45.6%  **--------------------------------------------------------------------------------**

15 Azfi_s0159.g053957/1-547 71.1% 24.5%  **--------------------------------------------------------------------------------**

16 Sacu_v1.1_s0011.g005198/1-632 99.4% 48.0%  **--------------------------------------------------------------------------------**

17 Mapoly0008s0172/7-441 92.5% 50.5%  **--------------------------------------------------------------------------------**

18 AaBONN_Sc2ySwM_344.3169.1/1-621 99.8% 50.2%  **--------------------------------------------------------------------------------**

19 AaBONN_Sc2ySwM_362.2644.1/1-479 95.9% 51.7%  **--------------------------------------------------------------------------------**

cov pid **1201**  **. . . . : . . .** **1280**

1 Selmo_145994/1-464 100.0% 100.0%  **--------------------------------------------------------------------------------**

2 Selmo_232533/1-472 98.9% 63.5%  **--------------------------------------------------------------------------------**

3 AT4G17090_BAM3/1-548 97.4% 50.4%  **--------------------------------------------------------------------------------**

4 Bradi3g33730/1-548 97.8% 48.9%  **--------------------------------------------------------------------------------**

5 Cre01.g044100/1-1337 99.1% 16.2%  **AAAGVIIASGHTARMDLCNSLSSLSTEFTTTGATNGAGNIASMHYLDVAASLVADGDDESDVGTSCCTAGAAEEADELFL**

6 Cre06.g270350/1-653 97.4% 27.4%  **--------------------------------------------------------------------------------**

7 Cre06.g307150/1-594 91.4% 36.2%  **--------------------------------------------------------------------------------**

8 Mapoly0043s0083/1-611 99.4% 50.7%  **--------------------------------------------------------------------------------**

9 Mapoly0043s0084/1-622 99.8% 50.9%  **--------------------------------------------------------------------------------**

10 Pp3c16_11260/1-612 99.8% 49.3%  **--------------------------------------------------------------------------------**

11 Pp3c25_2760/1-609 99.6% 49.1%  **--------------------------------------------------------------------------------**

12 Pp3c6_10130/1-611 99.6% 49.6%  **--------------------------------------------------------------------------------**

13 CHBRA1096g00040/1-868 98.9% 32.1%  **--------------------------------------------------------------------------------**

14 Azfi_s0206.g057798/1-543 93.3% 45.6%  **--------------------------------------------------------------------------------**

15 Azfi_s0159.g053957/1-547 71.1% 24.5%  **--------------------------------------------------------------------------------**

16 Sacu_v1.1_s0011.g005198/1-632 99.4% 48.0%  **--------------------------------------------------------------------------------**

17 Mapoly0008s0172/7-441 92.5% 50.5%  **--------------------------------------------------------------------------------**

18 AaBONN_Sc2ySwM_344.3169.1/1-621 99.8% 50.2%  **--------------------------------------------------------------------------------**

19 AaBONN_Sc2ySwM_362.2644.1/1-479 95.9% 51.7%  **--------------------------------------------------------------------------------**

cov pid **1281**  **. 3 . . . . : .** **1360**

1 Selmo_145994/1-464 100.0% 100.0%  **--------------------------------------------------------------------------------**

2 Selmo_232533/1-472 98.9% 63.5%  **--------------------------------------------------------------------------------**

3 AT4G17090_BAM3/1-548 97.4% 50.4%  **--------------------------------------------------------------------------------**

4 Bradi3g33730/1-548 97.8% 48.9%  **--------------------------------------------------------------------------------**

5 Cre01.g044100/1-1337 99.1% 16.2%  **GAGNSAGRTPHPSISEHAAALSLVPVAGRPLHHGQSAGTSSGSSGGGLQAQLQQLQPPHGARMAGIYSDDGRRSSAELSG**

6 Cre06.g270350/1-653 97.4% 27.4%  **--------------------------------------------------------------------------------**

7 Cre06.g307150/1-594 91.4% 36.2%  **--------------------------------------------------------------------------------**

8 Mapoly0043s0083/1-611 99.4% 50.7%  **--------------------------------------------------------------------------------**

9 Mapoly0043s0084/1-622 99.8% 50.9%  **--------------------------------------------------------------------------------**

10 Pp3c16_11260/1-612 99.8% 49.3%  **--------------------------------------------------------------------------------**

11 Pp3c25_2760/1-609 99.6% 49.1%  **--------------------------------------------------------------------------------**

12 Pp3c6_10130/1-611 99.6% 49.6%  **--------------------------------------------------------------------------------**

13 CHBRA1096g00040/1-868 98.9% 32.1%  **--------------------------------------------------------------------------------**

14 Azfi_s0206.g057798/1-543 93.3% 45.6%  **--------------------------------------------------------------------------------**

15 Azfi_s0159.g053957/1-547 71.1% 24.5%  **--------------------------------------------------------------------------------**

16 Sacu_v1.1_s0011.g005198/1-632 99.4% 48.0%  **--------------------------------------------------------------------------------**

17 Mapoly0008s0172/7-441 92.5% 50.5%  **--------------------------------------------------------------------------------**

18 AaBONN_Sc2ySwM_344.3169.1/1-621 99.8% 50.2%  **--------------------------------------------------------------------------------**

19 AaBONN_Sc2ySwM_362.2644.1/1-479 95.9% 51.7%  **--------------------------------------------------------------------------------**

cov pid **1361**  **. . . 4 . . . .** **1440**

1 Selmo_145994/1-464 100.0% 100.0%  **-------------------------------------------------------------------------SPV----**

2 Selmo_232533/1-472 98.9% 63.5%  **-------------------------------------------------------------------------AGI----**

3 AT4G17090_BAM3/1-548 97.4% 50.4%  **-------------------------------------------------------------------------SGA----**

4 Bradi3g33730/1-548 97.8% 48.9%  **-------------------------------------------------------------------------TGA----**

5 Cre01.g044100/1-1337 99.1% 16.2%  **MGLGVATLGVGMSTASAGTAVISGVGGVGGVAAAATNPVTALLSPTTSYHTAGTAPALATSSGGGAGAALYGATGM----**

6 Cre06.g270350/1-653 97.4% 27.4%  **-------------------------------------------------------------------------NGIWRGS**

7 Cre06.g307150/1-594 91.4% 36.2%  **-------------------------------------------------------------------------PAC----**

8 Mapoly0043s0083/1-611 99.4% 50.7%  **-------------------------------------------------------------------------TGA----**

9 Mapoly0043s0084/1-622 99.8% 50.9%  **-------------------------------------------------------------------------TGA----**

10 Pp3c16_11260/1-612 99.8% 49.3%  **-------------------------------------------------------------------------TEA----**

11 Pp3c25_2760/1-609 99.6% 49.1%  **-------------------------------------------------------------------------TGA----**

12 Pp3c6_10130/1-611 99.6% 49.6%  **-------------------------------------------------------------------------TGA----**

13 CHBRA1096g00040/1-868 98.9% 32.1%  **-------------------------------------------------------------------------TGA----**

14 Azfi_s0206.g057798/1-543 93.3% 45.6%  **-------------------------------------------------------------------------TGA----**

15 Azfi_s0159.g053957/1-547 71.1% 24.5%  **-------------------------------------------------------------------------TGS----**

16 Sacu_v1.1_s0011.g005198/1-632 99.4% 48.0%  **-------------------------------------------------------------------------SGA----**

17 Mapoly0008s0172/7-441 92.5% 50.5%  **---------------------------------------------------------------------------V----**

18 AaBONN_Sc2ySwM_344.3169.1/1-621 99.8% 50.2%  **-------------------------------------------------------------------------TGA----**

19 AaBONN_Sc2ySwM_362.2644.1/1-479 95.9% 51.7%  **-------------------------------------------------------------------------TGA----**

cov pid **1441**  **: . . . . 5 . .** **1520**

1 Selmo_145994/1-464 100.0% 100.0%  **----------RLSAKVAGIHWHYGT--RSHAPELTAGYYNTRFRDGYLPLARMFGRHGVTFNFTCFEMR--DVEQ-PAAA**

2 Selmo_232533/1-472 98.9% 63.5%  **----------KLAGKVAGVHWHYGT--KPHPAELTAGYYNTRLRDGYTGLARMFGRHGAVMIFTCLEMR--DLEQ-PPHA**

3 AT4G17090_BAM3/1-548 97.4% 50.4%  **----------KLSGKVAGIHWHYNT--RSHAAELTAGYYNTRNHDGYLPIAKMFNKHGVVLNFTCMEMK--DGEQ-PEHA**

4 Bradi3g33730/1-548 97.8% 48.9%  **----------MLSAKVAGIHWHYRT--RSHAAELTAGYYNTRNHDGYAPIAGMLAKRGVVLNFTCMEMK--DEQQ-PGHA**

5 Cre01.g044100/1-1337 99.1% 16.2%  **----------HLALKIAGIHWWYRS--RSHAAELTAGYYNVDGHDGYEAIVNLCARHRANLVLTCVEMC--DSQH-PAQA**

6 Cre06.g270350/1-653 97.4% 27.4%  **PAAAAEPPRLRLHAKLPGVHWWYNT--ASRAPELTAGFYNTTSRDGYLPIMEVLSRHGISVRLRSAEMR--SSEIAPQQA**

7 Cre06.g307150/1-594 91.4% 36.2%  **----------KMGIKLAGVHWWFKS--RAHAAELTAGYYNTRDRDGYLPFMAMLRRHDASLSFTCVEMR--DCEH-PPEG**

8 Mapoly0043s0083/1-611 99.4% 50.7%  **----------VLSGKVAGIHWHYGT--RSHAAELTAGYYNTRYRDGYAPIARMFGRHGVTLNFTCIEMK--DEEQ-PPQA**

9 Mapoly0043s0084/1-622 99.8% 50.9%  **----------VLSGKVAGIHWHYGT--RSHAAELTAGYYNTRYRDGYAPIARMFGRHGVTLNFTCIEMK--DEEQ-PPQA**

10 Pp3c16_11260/1-612 99.8% 49.3%  **----------VISGKVAGIHWHYGT--RSHAAELTAGYYNTRTRDGYAPIAQLFAKYGVTLNFTCFEMR--DLEQ-PSHA**

11 Pp3c25_2760/1-609 99.6% 49.1%  **----------VISGKVAGIHWHYGT--RSHAAELTAGYYNTRTRDGYATIAQMFAKYGVTLNFTCIEMR--DYEQ-PSQA**

12 Pp3c6_10130/1-611 99.6% 49.6%  **----------VISGKVAGIHWHYGT--RSHAAELTAGYYNTRSRDGYLPIAQMFAKYGVTLNFTCIEMR--DFEQ-PAHA**

13 CHBRA1096g00040/1-868 98.9% 32.1%  **----------MISAKIAGIHWHYYS--RSHAAELTAGYYNTWNRDGYLPIARMLARHGAILNFTCFEMR--DSEQ-MPQA**

14 Azfi_s0206.g057798/1-543 93.3% 45.6%  **----------KLSGKVAGIHWHYGT--RSHSAELTAGYYNTRTRDGYVPIARMFGRHGVTLNFTCFEMK--DEEQYPSSA**

15 Azfi_s0159.g053957/1-547 71.1% 24.5%  **----------KLSAKVPKALHHHGKEISSYFTHMVAGYL---YDDGYLRVMKMFGRHGVALNFTCLEMN--EIKQQDP--**

16 Sacu_v1.1_s0011.g005198/1-632 99.4% 48.0%  **----------KLSGKVAGIYWHYGT--QSHPAELTAGYYNTRTRDGYIPIAQMFGRHGVTLNFTCFEMR--DEEQYPPSA**

17 Mapoly0008s0172/7-441 92.5% 50.5%  **----------VLSAKISGIHWHYKT--PSHAAECGVGYYNTEKRNGYLPFARLFARQNTVFNFTCLEMK--DSQQ--DVA**

18 AaBONN_Sc2ySwM_344.3169.1/1-621 99.8% 50.2%  **----------HISGKVAGIHWHYGT--RSHAAELTAGYYNTRIRDGYLPIAHMFARHGVTLNFTCIEMR--DTEQ-PPHA**

19 AaBONN_Sc2ySwM_362.2644.1/1-479 95.9% 51.7%  **----------HISGKVSGIHWQHRT--SSHAAEVTAGYYNTETRDGYLPIASLFAKFGAILNFTCIEMK--DAEQ-PAEA**

cov pid **1521**  **. . : . . . . 6** **1600**

1 Selmo_145994/1-464 100.0% 100.0%  **QCSPEGLLKQVVAAAKSAGVPLAGENALPRY-----DEGAYHQIVMKSR-----------------------LEVEGE--**

2 Selmo_232533/1-472 98.9% 63.5%  **LSSPESLLHQVVSACKQAGISLAGENALPRF-----DEAAYEQVVKKSR-----------------------MQESEEED**

3 AT4G17090_BAM3/1-548 97.4% 50.4%  **NCSPEGLVKQVQNATRQAGTELAGENALERY-----DSSAFGQVVATNR-------------------------------**

4 Bradi3g33730/1-548 97.8% 48.9%  **GCSPEQLVRQVRAAARAANVELAGENALERY-----DESAFAQVAATAA-------------------------------**

5 Cre01.g044100/1-1337 99.1% 16.2%  **QCGPEGLLRQLRQLAARAGVQLSGENALPIFSSGGVDNDALDRIVNNMRSWPMPNNNVNSRVGISPAPSMVMPSGNGNGN**

6 Cre06.g270350/1-653 97.4% 27.4%  **CCDPERQVAQQRTVAAALLVPVGLENAHERF-----DESALARLEASL------------------------FDTSVH--**

7 Cre06.g307150/1-594 91.4% 36.2%  **RCSPQALLQQVIEAAEKYGVPLSGENALQRY-----DDYAFERIAESAF-------------------------------**

8 Mapoly0043s0083/1-611 99.4% 50.7%  **SCSPESLLKQVTVASRTAGVRLAGENALPRF-----DQTAYNQVVKKSR-----------------------LQFNLP--**

9 Mapoly0043s0084/1-622 99.8% 50.9%  **ACSPESLLRQVTVASRTAGVRLAGENALPRF-----DQTAYNQVVKKSR-----------------------LQFNLH--**

10 Pp3c16_11260/1-612 99.8% 49.3%  **LCSPEGLVKQVAFATRTAGTPMAGENALPRF-----DSSAHEQIITSSR-----------------------LRMPVE--**

11 Pp3c25_2760/1-609 99.6% 49.1%  **SCSPEGLVRQVALATRRAGIPMAGENALPRF-----DSSAHEQIVRKSR-----------------------LRMNEH--**

12 Pp3c6_10130/1-611 99.6% 49.6%  **LCSPEGLVRQVALATRKTGIPMAGENALPRF-----DSSAHEQIVRKSR-----------------------LQMNEK--**

13 CHBRA1096g00040/1-868 98.9% 32.1%  **ACSPESLLRQVVRASKIAGVRVSGENALQRF-----DLDAYEQIVRKSR-----------------------LRLDGL--**

14 Azfi_s0206.g057798/1-543 93.3% 45.6%  **LCSPEGLLKQVTLAARKASVPLAGENALLRF-----DEGAHHQIITKSR-----------------------LTVDGHD-**

15 Azfi_s0159.g053957/1-547 71.1% 24.5%  **--------------------------------------------------------------------------------**

16 Sacu_v1.1_s0011.g005198/1-632 99.4% 48.0%  **RCSPEGLLKQVTLAARKASVPLAGENALLRF-----DEGAHRQIIAKSR-----------------------LSDQ----**

17 Mapoly0008s0172/7-441 92.5% 50.5%  **NSSPETLIKQVAASCRKADIRMSGENALAVF-----DDASYDQISETAS-----------------------LQFDGD--**

18 AaBONN_Sc2ySwM_344.3169.1/1-621 99.8% 50.2%  **LCSPEGLLRQIAVAARTAGIRLSGENALPRF-----DESAHDQVVRKSR-----------------------LQVHED--**

19 AaBONN_Sc2ySwM_362.2644.1/1-479 95.9% 51.7%  **QSSPEKLIEQVAAAAGSAGIALSGENALSRF-----DEVAFDQVLKQAR-----------------------LPSAGD--**

cov pid **1601**  **. . . . : . . .** **1680**

1 Selmo_145994/1-464 100.0% 100.0%  **--------ESMERAYEPMCCFTFLRMNERLFHPENWRRFVQFVKEIGDGK---------GSSS-S---------------**

2 Selmo_232533/1-472 98.9% 63.5%  **DWISASSGGCSSTACEPMCSFTFLRMSEKLFYSENWHNFVPFVRRMAGGR---------AF-------------------**

3 AT4G17090_BAM3/1-548 97.4% 50.4%  **-----------SDSGNGLTAFTYLRMNKRLFEGQNWQQLVEFVKNMKEGG---------HGRRLS---------------**

4 Bradi3g33730/1-548 97.8% 48.9%  **----------AGDAGAGLSAFTYLRMNRNLFDGDNWRRFVAFVKTMADGG------GARTGLP-----------------**

5 Cre01.g044100/1-1337 99.1% 16.2%  **GNGNGNGNGAHASSGGNIVCVGY-RMSESGRMVPVGTAASAVVNANGNGSGNGNGYGLNGASGSQSAA------------**

6 Cre06.g270350/1-653 97.4% 27.4%  **----------EGIELPQVQSLVFNRMCDSMFEPGNWSRFKEFVRRVRNRA-------DTLVVPAWRWRGGPGSSMDGMAL**

7 Cre06.g307150/1-594 91.4% 36.2%  **---------GRNARAGRLTQVTFLRMGDLMF--DNWDAFSRFLNRMRNKA------------------------------**

8 Mapoly0043s0083/1-611 99.4% 50.7%  **--------VDSHEEQEPMCAFTYLRMSESLFQAENWRLFVPFVRHMQEGR---------TFQT-W---------------**

9 Mapoly0043s0084/1-622 99.8% 50.9%  **--------GDSQEEQEPMCAFTYLRMSESLFQADNWRLFVPFVRHMQEGR---------TFQP-W---------------**

10 Pp3c16_11260/1-612 99.8% 49.3%  **--------GDCHQDYEPMAAFTFLRMSESMFHSENWRLFVPFVRHMEEGR---------TFQP-W---------------**

11 Pp3c25_2760/1-609 99.6% 49.1%  **--------GDCHEEYEPMAAFTFLRMCESLFHSENWKLFVPFVRHMEEGR---------TFQP-W---------------**

12 Pp3c6_10130/1-611 99.6% 49.6%  **--------GDCQEHYEPMSAFTFLRMCESLFHSENWRLFVPFVRHMEEGR---------TFQP-W---------------**

13 CHBRA1096g00040/1-868 98.9% 32.1%  **------------ATDEPMMSFTFLRTSEMLFRPNNWSNFVAFVRNMSKGT--------EEVMPHWVKE------------**

14 Azfi_s0206.g057798/1-543 93.3% 45.6%  **--------TADETIYEPMCSFTFLRMSEQLFRAENWRRFVQFVHQMDEGR---------TFQP-W---------------**

15 Azfi_s0159.g053957/1-547 71.1% 24.5%  **--------------------------SNQLFLSDAWRTFVKFVEDKSKRA------------------------------**

16 Sacu_v1.1_s0011.g005198/1-632 99.4% 48.0%  **--------NGGQVIYEPMCSFTFLRMTEQLFQSDNWREFVRFVHQMAEGR---------TSQS-W---------------**

17 Mapoly0008s0172/7-441 92.5% 50.5%  **----------------NIVSFTYLRLSEDLFTGENWSNFEEFVAKMVSSA------------------------------**

18 AaBONN_Sc2ySwM_344.3169.1/1-621 99.8% 50.2%  **--------GSSLTAFEPMCSFTFLRMCERLFHNDNWRVFVPFVMHMEEGR---------TFQP-W---------------**

19 AaBONN_Sc2ySwM_362.2644.1/1-479 95.9% 51.7%  **--------KGVLEKPDRLCSFTYLRMCEDLFEPENWNIFVSFVKNMHQ--------------------------------**

cov pid **1681**  **. 7 . . . . : ]** **1755**

1 Selmo_145994/1-464 100.0% 100.0%  **---REHEHRASELLVATKP----LIQEAAAALV------------------------------------------**

2 Selmo_232533/1-472 98.9% 63.5%  **---QEEHHDTESHMHATRP-----VQEAAAALMCH----------------------------------------**

3 AT4G17090_BAM3/1-548 97.4% 50.4%  **---KEDTTGSDLYVGFVKGKIAENVEEAALV--------------------------------------------**

4 Bradi3g33730/1-548 97.8% 48.9%  **---SCDTGHSDLYVGFLEAANERRAPEAEAAAAAL----------------------------------------**

5 Cre01.g044100/1-1337 99.1% 16.2%  **---ESGSQINGQPTSAVTPHGSTDLNGAYSTCLSPAPPEVLPSLRAFTFLRLVPEMLLPGYQSLWMRFMGKLLAS**

6 Cre06.g270350/1-653 97.4% 27.4%  **PLTVELPMGGEPQQVSAGSGSNVAMAAAAAAAAAAAAVAAGNGHGSGATGAAGGGGDAGMTAAAPTGSAGALQLV**

7 Cre06.g307150/1-594 91.4% 36.2%  **---------------------------------------------------------------------------**

8 Mapoly0043s0083/1-611 99.4% 50.7%  **---EQGHQDSESHVHATRP----MVQEAV--LMYH----------------------------------------**

9 Mapoly0043s0084/1-622 99.8% 50.9%  **---EQGHQDSESHVHATRP----LVQEAAQALMYH----------------------------------------**

10 Pp3c16_11260/1-612 99.8% 49.3%  **---EEEHQRTETHVKATGP----LVQEAASLMLH-----------------------------------------**

11 Pp3c25_2760/1-609 99.6% 49.1%  **---EEEHHRTETHVHATRP----LVQEAA-SLMY-----------------------------------------**

12 Pp3c6_10130/1-611 99.6% 49.6%  **---EEESHRTQNDMHATQP----LVQEAA-SLMYH----------------------------------------**

13 CHBRA1096g00040/1-868 98.9% 32.1%  **---KENHHAVSPPIWANVH----NSSKHLGQSIAV----------------------------------------**

14 Azfi_s0206.g057798/1-543 93.3% 45.6%  **---EDRHSTADSHVYATTP----LIQEAEAAMLC-----------------------------------------**

15 Azfi_s0159.g053957/1-547 71.1% 24.5%  **------FSHRLSSCHVSTP----FLQQPSS---------------------------------------------**

16 Sacu_v1.1_s0011.g005198/1-632 99.4% 48.0%  **---ENRHSDTESHVYLMAP----LIQEAEVAMLCN----------------------------------------**

17 Mapoly0008s0172/7-441 92.5% 50.5%  **---SVNQSGFRSKI-------------------------------------------------------------**

18 AaBONN_Sc2ySwM_344.3169.1/1-621 99.8% 50.2%  **---EEEHQNTVSHVHATNP----LIQEAAAALMYH----------------------------------------**

19 AaBONN_Sc2ySwM_362.2644.1/1-479 95.9% 51.7%  **---VEGAQNQMLPTKSTL---------------------------------------------------------**

>Selmo_145994/1-464

------------------------------------------------------------------------

------------------------------------------------------------------------

------------------------------------------------------------------------

------------------------------------------------------------------------

------------------------------------------------------------------------

------------------------------------------------------------------------

----------------MLPLDS---INSGNN---QVNRARAMNASFQALK-SAGVEGVMVDVWWGIVEKDGP

CNYNWSGYRELLEMAKKHGLKVQAVMSFHQCGGN-VGDSAFIPLPWWVVEEAKNNPDMVYTDRY-----GNR

NFEYLSLGCDHLP-VLKGRTPVQAYSDFMRSFKESFSDML-GDVIVEIQVGMGPAGELRYPGYPERDGIWKF

PGVGEFQCHDNYMLASLKASAESIGKPDWG-CAPSDAGHYNQWPEDSIFFKRD-GGWNTDYGRFFLEWYSGK

LIEHGESVLTAAEGIF------------------R-----------------G-------------------

------------------------------------------------------------------------

------------------------------------------------------------------------

------------------------------------------------------------------------

------------------------------------------------------------------------

------------------------------------------------------------------------

------------------------------------------------------------------------

------------------------------------------------------------------------

------------------------------------------------------------------------

-----------------------------------------------------------------SPV----

----------RLSAKVAGIHWHYGT--RSHAPELTAGYYNTRFRDGYLPLARMFGRHGVTFNFTCFEMR--D

VEQ-PAAAQCSPEGLLKQVVAAAKSAGVPLAGENALPRY-----DEGAYHQIVMKSR---------------

--------LEVEGE----------ESMERAYEPMCCFTFLRMNERLFHPENWRRFVQFVKEIGDGK------

---GSSS-S------------------REHEHRASELLVATKP----LIQEAAAALV---------------

---------------------------

>Selmo_232533/1-472

------------------------------------------------------------------------

------------------------------------------------------------------------

------------------------------------------------------------------------

------------------------------------------------------------------------

------------------------------------------------------------------------

------------------------------------------------------------------------

----------------MLPLDS---VNV-NN---TLNRRRALNAGLIALK-SAGVEGVMVDVWWGIVEREKP

HHYKWSAYKELVSLIQKNGLKIQVVMSFHQCGGN-VGDSCYIPLPLWVLEEVQNNPNIVYTDKS-----GNR

NHEYLSLGCDFLP-VLRGRTPIQAYSDFMRSFKHVFKDVL-GETIVEVQVGLGPAGELRYPAYPEYNGKWRF

PGIGEFQCYDKYMLASLRACATACGTKHWGQGGPHDAGHYNQWPDDTGFFNRD-GSWNSPYGQFFLEWYSGM

LISHGERVLSAAEAVF------------------R-----------------G-------------------

------------------------------------------------------------------------

------------------------------------------------------------------------

------------------------------------------------------------------------

------------------------------------------------------------------------

------------------------------------------------------------------------

------------------------------------------------------------------------

------------------------------------------------------------------------

------------------------------------------------------------------------

-----------------------------------------------------------------AGI----

----------KLAGKVAGVHWHYGT--KPHPAELTAGYYNTRLRDGYTGLARMFGRHGAVMIFTCLEMR--D

LEQ-PPHALSSPESLLHQVVSACKQAGISLAGENALPRF-----DEAAYEQVVKKSR---------------

--------MQESEEEDDWISASSGGCSSTACEPMCSFTFLRMSEKLFYSENWHNFVPFVRRMAGGR------

---AF----------------------QEEHHDTESHMHATRP-----VQEAAAALMCH-------------

---------------------------

>AT4G17090_BAM3/1-548

------------------------------------------------------------------------

------MELTLNSSSSLIKRKDAKSSRN---QESSSNNMTFAKMKPPTYQFQAKNSVK-----------EMK

FTHEKTFTPEGETLEKWEKLHVLSYPHSK-------------------------------------------

------------------------------------------------------------------------

------------------------------------------------------------------------

------------------------------------------------------------------------

-------NDASVPVFVMLPLDT---VTM-SG---HLNKPRAMNASLMALK-GAGVEGVMVDAWWGLVEKDGP

MNYNWEGYAELIQMVQKHGLKLQVVMSFHQCGGN-VGDSCSIPLPPWVLEEISKNPDLVYTDKS-----GRR

NPEYISLGCDSVP-VLRGRTPIQVYSDFMRSFRERFEGYI-GGVIAEIQVGMGPCGELRYPSYPESNGTWRF

PGIGEFQCYDKYMKSSLQAYAESIGKTNWGTSGPHDAGEYKNLPEDTEFFRRD-GTWNSEYGKFFMEWYSGK

LLEHGDQLLSSAKGIF------------------Q-----------------G-------------------

------------------------------------------------------------------------

------------------------------------------------------------------------

------------------------------------------------------------------------

------------------------------------------------------------------------

------------------------------------------------------------------------

------------------------------------------------------------------------

------------------------------------------------------------------------

------------------------------------------------------------------------

-----------------------------------------------------------------SGA----

----------KLSGKVAGIHWHYNT--RSHAAELTAGYYNTRNHDGYLPIAKMFNKHGVVLNFTCMEMK--D

GEQ-PEHANCSPEGLVKQVQNATRQAGTELAGENALERY-----DSSAFGQVVATNR---------------

---------------------------SDSGNGLTAFTYLRMNKRLFEGQNWQQLVEFVKNMKEGG------

---HGRRLS------------------KEDTTGSDLYVGFVKGKIAENVEEAALV-----------------

---------------------------

>Bradi3g33730/1-548

------------------------------------------------------------------------

-------------MALTLRSSTSFLSPL---EPSSKLHKAEDAPPSCVAVPAAPSRLR-----------VLR

AAAQAPLSPMEAPAPELLHGQAQ-------------------------------------------------

------------------------------------------------------------------------

------------------------------------------------------------------------

------------------------------------------------------------------------

QAHSGGQKRGGVPVYVMLPLDT---VGP-GG---QLLRARALAASLMALR-SAGVEGVMVDVWWGVVEREGP

GRYDWEGYAELVRMVERAGLRLQMVMSFHQCGGN-VGDSCNIPLPSWVLEEVSANPDIVYTDRS-----GRR

NPEYISLGCDTLP-VLKGRTPVQVYSDFMRSFRDRFSGYL-GTVIAEIQVGLGPCGELRYPSYPEANGTWSF

PGIGEFQCYDKYMRASLQAAAAAAGHENWGTNGPHDAGEYKQFPEETGFFRWD-GTWSTEYGSFFLEWYSGM

LLEHGDRVLAAAEAVF------------------G-----------------G-------------------

------------------------------------------------------------------------

------------------------------------------------------------------------

------------------------------------------------------------------------

------------------------------------------------------------------------

------------------------------------------------------------------------

------------------------------------------------------------------------

------------------------------------------------------------------------

------------------------------------------------------------------------

-----------------------------------------------------------------TGA----

----------MLSAKVAGIHWHYRT--RSHAAELTAGYYNTRNHDGYAPIAGMLAKRGVVLNFTCMEMK--D

EQQ-PGHAGCSPEQLVRQVRAAARAANVELAGENALERY-----DESAFAQVAATAA---------------

--------------------------AGDAGAGLSAFTYLRMNRNLFDGDNWRRFVAFVKTMADGG------

GARTGLP--------------------SCDTGHSDLYVGFLEAANERRAPEAEAAAAAL-------------

---------------------------

>Cre01.g044100/1-1337

------------------------------------------------------------------------

----------MSSENILLDRALSVQGPG---YGYLHLGGVYGDCVATTALGYRAAAPS-----------MPA

SASVPSFGQSGVLRPHPRRYSQNAFGTSL-------------------------------------------

------------------------------------------------------------------------

------------------------------------------------------------------------

-----------------------------------------------------------------------S

PVHGPSDGGTGANVYCMLPLDT---VNS-EGVF-RYAGSAWFAQALQLLV-ASGAHGVAMDFWWGAVER-SP

GQYNWSGYKQALEVIKQTGLKVQVVLSFHACGGN-VGDTVQIPLPDWVVQCAEADPDLFFADRPRNGGLGNR

NREYLSIWADDAPGVLRGRSPMQCYEEYMVSLRENFSQEL-GTVIDEVVVGAGPCGELRLPSYVEANG-WRF

PGAGEFQCYDRRALASLAQAAREAGHPEWGYTGPHDAGEYNSTPEHTGFFSHN-GSWNTPYGRFFLEWYSGC

LLKHGDRLLTVANAVF------------------ASKLNPPPQPPQSLLQTLGAAGSALAAQLQQVAQQTVG

SLGHGSSGGNAMGNGAGSGPSSSHGVQAFLSPMAPGGMNSYVTNTPFQPMSFMGLHTSQTSSGGPRMSTHSL

NRILETGGSCGSRPGSGHASGNELTGSASADGGGMPPPPAPPLPRRQATEGMLSSCGVPSSISPCCTYSSMS

TYTGTSSGGASGSTANMIMATAGSSGSLMSPRSMSVFHGTACGAPHSEAHGAVGAAAAAGTVLGGRRSSGGG

AAGSPQNASGVFSAAMNAMVTRGVATAAALMQAAAAQGGAATSMAATNARAVAAACGSSSRTSSDSAAATNV

GTTGAAALASGDATMIDVEPSDNSHTSTVAELMNAQGLSGSSNASGPLAHPLNAAAQPGTLAPTAVTAAASG

AIVAAPGAGHASCHPSPLSGVRFRSSASTLADAEPMFGSGQGTAFAAVAAAGVIIASGHTARMDLCNSLSSL

STEFTTTGATNGAGNIASMHYLDVAASLVADGDDESDVGTSCCTAGAAEEADELFLGAGNSAGRTPHPSISE

HAAALSLVPVAGRPLHHGQSAGTSSGSSGGGLQAQLQQLQPPHGARMAGIYSDDGRRSSAELSGMGLGVATL

GVGMSTASAGTAVISGVGGVGGVAAAATNPVTALLSPTTSYHTAGTAPALATSSGGGAGAALYGATGM----

----------HLALKIAGIHWWYRS--RSHAAELTAGYYNVDGHDGYEAIVNLCARHRANLVLTCVEMC--D

SQH-PAQAQCGPEGLLRQLRQLAARAGVQLSGENALPIFSSGGVDNDALDRIVNNMRSWPMPNNNVNSRVGI

SPAPSMVMPSGNGNGNGNGNGNGNGAHASSGGNIVCVGY-RMSESGRMVPVGTAASAVVNANGNGSGNGNGY

GLNGASGSQSAA---------------ESGSQINGQPTSAVTPHGSTDLNGAYSTCLSPAPPEVLPSLRAFT

FLRLVPEMLLPGYQSLWMRFMGKLLAS

>Cre06.g270350/1-653

------------------------------------------------------------------------

---------MRASIDRQCCAAASSPASA---RCPILGLLSRGQKRLGWRLGGHIASSP-----------HAE

LTSPTPVAAGATARAAAPTWRSTVDEEAV-------------------------------------------

------------------------------------------------------------------------

------------------------------------------------------------------------

------------------------------------------------------------------------

-----------LPLYTCLDGDI---LTP-AN---KFAYYEALRSGLKALR-ALGINGISVDVYWGIVEGAAP

MEYDWSSYKQLFALIRDEGFMAQVCLCFH--GTE------AVPLPAWVLAAGAANPDIYFTDRA-----GVR

NTHCISLGVDEVP-ALDGRTALACYRDLMTSFRVELEPLL-GSTIVDVCVGLGPDGELKYPAHP-RDRRWNF

PGIGEFQCYDKYMLAGLRACSHQVSQPSWGLGGPHDAGAYTVWPQQTGFFNQY-GNWSSPYGKFFLQWYSDM

LMQHADSVLGIARDVLLPADGSLSFSGSSAGFGSSSSSSSVRPGFYGNGYGNG-------------------

------------------------------------------------------------------------

------------------------------------------------------------------------

------------------------------------------------------------------------

------------------------------------------------------------------------

------------------------------------------------------------------------

------------------------------------------------------------------------

------------------------------------------------------------------------

------------------------------------------------------------------------

-----------------------------------------------------------------NGIWRGS

PAAAAEPPRLRLHAKLPGVHWWYNT--ASRAPELTAGFYNTTSRDGYLPIMEVLSRHGISVRLRSAEMR--S

SEIAPQQACCDPERQVAQQRTVAAALLVPVGLENAHERF-----DESALARLEASL----------------

--------FDTSVH------------EGIELPQVQSLVFNRMCDSMFEPGNWSRFKEFVRRVRNRA------

-DTLVVPAWRWRGGPGSSMDGMALPLTVELPMGGEPQQVSAGSGSNVAMAAAAAAAAAAAAVAAGNGHGSGA

TGAAGGGGDAGMTAAAPTGSAGALQLV

>Cre06.g307150/1-594

------------------------------------------------------------------------

----------MPTSLPQCPSAPRSVQPL---RSALLAAQRLSQCRGSRSQRGSGACHS-----------QGR

QVTHPTAHPRTSRTLDDDSGFASFAPEPIVTTPGASAFSE---V---------EDDLQIHRLLRETGQHIKI

PMIDEDRFD---------------------------------------------------------------

------------------------------------------------------------------------

------------------------------------------------------------------TQYPES

QAEEPSSSGSGCPVYVMLPLDTVWVVERDGKRISVLKKERSLDIALHTLK-QAGVEGVMVDVWWGIVERAGP

RQYDFSAYKRLFYKVAAAGLKVQAVMSFHAAGGN-VGDTCKIPLPKWVLEIGERNPDIFYTDKA-----GHR

NRECLSLGCDEVP-LFWGRTPVLMYRDFINAFADKFQHLF-GTVITEVTVGLGPAGELRYPSYPEGDGRWRF

PGVGEFQCYDKFMLESLRRTAEAAGHAEWGLSGPHDAGHYNSSSWETGFFVSQNGSWNTAYGHFFLSWYSNM

LLEHADRVLSSAAEVL------------NKHGRPRVFNSMRDASNGHVIYEFT-------------------

------------------------------------------------------------------------

------------------------------------------------------------------------

------------------------------------------------------------------------

------------------------------------------------------------------------

------------------------------------------------------------------------

------------------------------------------------------------------------

------------------------------------------------------------------------

------------------------------------------------------------------------

-----------------------------------------------------------------PAC----

----------KMGIKLAGVHWWFKS--RAHAAELTAGYYNTRDRDGYLPFMAMLRRHDASLSFTCVEMR--D

CEH-PPEGRCSPQALLQQVIEAAEKYGVPLSGENALQRY-----DDYAFERIAESAF---------------

-------------------------GRNARAGRLTQVTFLRMGDLMF--DNWDAFSRFLNRMRNKA------

------------------------------------------------------------------------

---------------------------

>Mapoly0043s0083/1-611

------------------------------------------------------------------------

-----------MAAACSLVGPSMFSSHS---RRVLVTEFSAVEVSSSSSVVDVPVRAR-----------CTF

RSSSVGSGHGPCIQRAAKELDTCLSYSPFSSS-----------------------SPSADERWRSQGSRGSH

LEALDMGMFGTAAEGERMVP----------------------------------------------------

------------------------------------------------------------------------

------------------------------------------------------GLEVGAGSW---LEHHSE

ETSSSRGTSGGVPVFVMLPLDS---VKM-SN---VVNRKRAMNASLLALK-SAGVEGIMMDVWWGIVEKDGP

MKYNWSAYLELITMARKHGLKVQCVMSFHQCGGN-VGDTCTIPLPPWVVEEIKSNNDLVYTDKN-----GNR

NFEYLSLGCDSLP-VLKGRTQVQVYSDFMRSFRETFKDLL-GNTVIEIQVGMGPAGELRYPGYPEQNGRWRF

PGIGEFQCYDKYMIASLKSAAEAIGMPAWGHAGPNDAGDYNRWPEEVAFFSRD-GGWKSAYGEFFLDWYSKM

LINHGEKILSAAAGIF------------------R-----------------G-------------------

------------------------------------------------------------------------

------------------------------------------------------------------------

------------------------------------------------------------------------

------------------------------------------------------------------------

------------------------------------------------------------------------

------------------------------------------------------------------------

------------------------------------------------------------------------

------------------------------------------------------------------------

-----------------------------------------------------------------TGA----

----------VLSGKVAGIHWHYGT--RSHAAELTAGYYNTRYRDGYAPIARMFGRHGVTLNFTCIEMK--D

EEQ-PPQASCSPESLLKQVTVASRTAGVRLAGENALPRF-----DQTAYNQVVKKSR---------------

--------LQFNLP----------VDSHEEQEPMCAFTYLRMSESLFQAENWRLFVPFVRHMQEGR------

---TFQT-W------------------EQGHQDSESHVHATRP----MVQEAV--LMYH-------------

---------------------------

>Mapoly0043s0084/1-622

------------------------------------------------------------------------

-----------MAAVYWLVGPSTFLGSS---RSVVAPESSAVEIPSSSNVVDGPVRVR-----------CVI

QGSSLGSGPGQSTQRSSKDFDTCLTASSFPSTSLSTSPPS---S-----------SPSADEWWRSQGSRGSH

PDALDMRMFGIATEGQSKVA----------------------------------------------------

------------------------------------------------------------------------

------------------------------------------------------DWELGSTPW---LEHHLE

ETSTSRGTSGGVPVFVMLPLDS---VNM-NN---TVNRKRAMNAGLLALK-SAGVEGIMMDVWWGIVEKDGP

MKYNWSGYIELINMARKHGLKVQCVMSFHQCGGN-VGDSCNIPLPPWVVEEIKSNNDLVYTDKY-----GNR

NYEYLSLGCDSLP-VLKGRTPVQVYSDFMRSFRDTFKDLL-GDTVIEIQVGMGPAGELRYPGYPEQNGRWRF

PGIGEFQCYDKYMIASLKSAAEAIGKPAWGHGGPSDAGDYNRWPDETAFFSRD-GGWKSAYGEFFLDWYSKM

LINHGEKILSAAAGIF------------------R-----------------G-------------------

------------------------------------------------------------------------

------------------------------------------------------------------------

------------------------------------------------------------------------

------------------------------------------------------------------------

------------------------------------------------------------------------

------------------------------------------------------------------------

------------------------------------------------------------------------

------------------------------------------------------------------------

-----------------------------------------------------------------TGA----

----------VLSGKVAGIHWHYGT--RSHAAELTAGYYNTRYRDGYAPIARMFGRHGVTLNFTCIEMK--D

EEQ-PPQAACSPESLLRQVTVASRTAGVRLAGENALPRF-----DQTAYNQVVKKSR---------------

--------LQFNLH----------GDSQEEQEPMCAFTYLRMSESLFQADNWRLFVPFVRHMQEGR------

---TFQP-W------------------EQGHQDSESHVHATRP----LVQEAAQALMYH-------------

---------------------------

>Pp3c16_11260/1-612

------------------------------------------------------------------------

-----------MVVFTISHSVAWKSSFG---GCSVLKNASEPEVSSSTRDDHVRQPVT--------------

---KLSPLVWGLGRQQFDGYAAQSSAGSLSSASA---------------------SSFPEEWWRKNSNNVAD

SMGGLRATHHTQTVSMFPERRSIL------------------------------------------------

------------------------------------------------------------------------

------------------------------------------------------DWDNTSDKW---KEHAFH

ETATSRGVHGRVPVFVMLPLDS---VNM-NN---TLNRRRALNASLMALK-SAGIEGIMMDVWWGIVEKDAP

LNYNWSAYRELIEMARKHGLKVQAVMSFHQCGGN-VGDSCNIPLPPWVLEEIQKNPDLAYTDKS-----GRR

NAEYICLGADNVP-ALKGRTPVQCYADFMRSFRDNFEDLL-GDVIIEIQCGMGPAGELRYPSYPESEGRWRF

PGIGEFQCYDKYMLAGLKASAEAVGMPAWGTSGPHDAGNYNQWPDDTGFFRKD-GTWSTDYGQFFMEWYSEM

LLAHGERILSVATGIF------------------R-----------------D-------------------

------------------------------------------------------------------------

------------------------------------------------------------------------

------------------------------------------------------------------------

------------------------------------------------------------------------

------------------------------------------------------------------------

------------------------------------------------------------------------

------------------------------------------------------------------------

------------------------------------------------------------------------

-----------------------------------------------------------------TEA----

----------VISGKVAGIHWHYGT--RSHAAELTAGYYNTRTRDGYAPIAQLFAKYGVTLNFTCFEMR--D

LEQ-PSHALCSPEGLVKQVAFATRTAGTPMAGENALPRF-----DSSAHEQIITSSR---------------

--------LRMPVE----------GDCHQDYEPMAAFTFLRMSESMFHSENWRLFVPFVRHMEEGR------

---TFQP-W------------------EEEHQRTETHVKATGP----LVQEAASLMLH--------------

---------------------------

>Pp3c25_2760/1-609

------------------------------------------------------------------------

------------MSSAMCRSATLANRFV---CGNVLTDRLQSEASSSGRVELARFSAR--------------

---RAVPGVSCSQRNQSDGHVAHTSSGSVSSFG----------------------SLSSEEWWRKNTGSAGD

STGGLRATHHTQMDTMFPEHKSLL------------------------------------------------

------------------------------------------------------------------------

------------------------------------------------------DWDNTADEW---KEHAFH

ETPTSRGVHGGVPVFVMLPLDS---VNI-NN---TLKRRRALNASLLALK-SAGVEGVMMDVWWGIVEKEGP

RNYNWSAYRELIDMVRKHGLKVQAVMSFHQCGGN-VGDSCNIPLPPWVLEEVQKNPDLAYTDKA-----GKR

NAEYISLGADNVP-ALKGRTPVQCYADFMRSFRDNFKDLL-GDVIIEIQCGMGPAGELRYPSYPESEGRWRF

PGIGEFQCYDKYMLASLKANAQALGKPAWGHGGPCDAGNYNQWPDETGFFHRD-GSWCSEYGQFFMEWYSEM

ILAHGERLLASASGIF------------------K-----------------G-------------------

------------------------------------------------------------------------

------------------------------------------------------------------------

------------------------------------------------------------------------

------------------------------------------------------------------------

------------------------------------------------------------------------

------------------------------------------------------------------------

------------------------------------------------------------------------

------------------------------------------------------------------------

-----------------------------------------------------------------TGA----

----------VISGKVAGIHWHYGT--RSHAAELTAGYYNTRTRDGYATIAQMFAKYGVTLNFTCIEMR--D

YEQ-PSQASCSPEGLVRQVALATRRAGIPMAGENALPRF-----DSSAHEQIVRKSR---------------

--------LRMNEH----------GDCHEEYEPMAAFTFLRMCESLFHSENWKLFVPFVRHMEEGR------

---TFQP-W------------------EEEHHRTETHVHATRP----LVQEAA-SLMY--------------

---------------------------

>Pp3c6_10130/1-611

------------------------------------------------------------------------

------------MAHAMCGNYILTNSFI---GRSVLTDNSQSESSPSDSVEVMRTRVR--------------

---KPASVVLCLQRQHFDGHAAQSTSGPLSASSA---------------------SPFPDEWWRKHSSTSGV

SMAELRVSQFAEMETMFPEHRSIL------------------------------------------------

------------------------------------------------------------------------

------------------------------------------------------DWQNTAEEW---IEHSTH

ETPTSRGVCGGVPVFVMLPLDS---VNM-NN---TLNRRRAMNASLLALK-SAGVEGIMMDVWWGIVEKDGP

HQYNWSAYRELIDMVRNHGLKVQAVMSFHQCGGN-VGDSCNVPLPPWVLEEVRKNPDLAYTDRV-----GRR

NAEYISLGADNVP-ALQGRTPVQCYADFMRSFRDNFKDLL-GDVIIEIQCGMGPAGELRYPSYPESEGRWRF

PGIGEFQSYDKYMIASLKASAHAVGKPAWGSGGPHDSGSYNQWPEETGFFKKD-GTWSTEYGQFFMEWYSEM

LLAHGERILSEATGIF------------------R-----------------G-------------------

------------------------------------------------------------------------

------------------------------------------------------------------------

------------------------------------------------------------------------

------------------------------------------------------------------------

------------------------------------------------------------------------

------------------------------------------------------------------------

------------------------------------------------------------------------

------------------------------------------------------------------------

-----------------------------------------------------------------TGA----

----------VISGKVAGIHWHYGT--RSHAAELTAGYYNTRSRDGYLPIAQMFAKYGVTLNFTCIEMR--D

FEQ-PAHALCSPEGLVRQVALATRKTGIPMAGENALPRF-----DSSAHEQIVRKSR---------------

--------LQMNEK----------GDCQEHYEPMSAFTFLRMCESLFHSENWRLFVPFVRHMEEGR------

---TFQP-W------------------EEESHRTQNDMHATQP----LVQEAA-SLMYH-------------

---------------------------

>CHBRA1096g00040/1-868

-----------------------------MDLADKKSDRVVSKLPTSRTPSSLRRSDCSATSIVYPESVLTG

ADKHHWERLTSSSPCYSSSSSSSSSSYS---SYSAFSPSSSLSSPRSSSPLPARPPVR-----------STA

SSTLPPSCSSASPSSSSSSSSSSSAPSPLFLSSPLVSPPS---TEEELWCRFQKMGYWPEVWMVDPSTKYSS

KIRESSSPLPRMPPSRGPSSSSPSSSPPSSPSSSPPSSPLPSAPSSPASNVSLSPPSSRRPSSLSACTGNSK

RRTCPAAPASAMSVGIAARPMEERTEDDRQQMGDDVTAEELLSHVLPAGSRDTCCGGGAAAAAEVDGVLGSR

SRQKSLPRSSSSSSSRAGRRTGIVMLPVLTEERQQERREQRSPGEVGAMRERERERGRGRDAWRI-HDVWDH

ASPGGRGAGNGVPVYIMLPLDT---VNM-NN---TLTRPRALRASLTALK-SAGVEGVMVDVWWGIVEGQEP

HKYRWTAYQDLVKMVHDAGLKMQVVMSFHQCGGN-VGDSCLIPLPQWVLQEIEKNPDIVYTDKA-----GKR

NLEYLSLGCDSLP-VLRGRSPVQAYADFMRSFRNVFASYL-GNVISEIQVGLGPAGELRYPSYPESYSRWRF

PGIGEFQCYDKHMLANLKAAAEAIGKPRWGLGGPHDAGSYNQWPEETGFFQRD-GSWKSQYGHFFLQWYSEM

LLRHGDLILSSAAGIF------------------R-----------------G-------------------

------------------------------------------------------------------------

------------------------------------------------------------------------

------------------------------------------------------------------------

------------------------------------------------------------------------

------------------------------------------------------------------------

------------------------------------------------------------------------

------------------------------------------------------------------------

------------------------------------------------------------------------

-----------------------------------------------------------------TGA----

----------MISAKIAGIHWHYYS--RSHAAELTAGYYNTWNRDGYLPIARMLARHGAILNFTCFEMR--D

SEQ-MPQAACSPESLLRQVVRASKIAGVRVSGENALQRF-----DLDAYEQIVRKSR---------------

--------LRLDGL--------------ATDEPMMSFTFLRTSEMLFRPNNWSNFVAFVRNMSKGT------

--EEVMPHWVKE---------------KENHHAVSPPIWANVH----NSSKHLGQSIAV-------------

---------------------------

>Azfi_s0206.g057798/1-543

------------------------------------------------------------------------

----MAFPSSTLPGSLHGSSPETVRNML---HTSTATSSSSSSSTTSTTNISSSSSSS-----------DQK

HHRKSSSSPHSLIHNSSSSSSQSPPPPSF-------------------------------------------

------------------------------------------------------------------------

------------------------------------------------------------------------

-----------------------------------------------------------------LSSITNS

YNNIQSSAVLWGPINLV-EEKL---FDFHDS---PVFSKNSISESLVDLN-LTPL-----------------

-------HKELIIML------IIVAKEDIQCGSI-RCAWCRIPLPPWVKEEIDQNPDLVYTDKD-----GRR

NYEYLSLGCDSLP-VLKGRSPIQVYSDFMRDFRDEFSEYL-GEVIVEIQVGMGPAGELRYPAYPESNNTWQF

PGIGEFQCYDKYMLANLQASADSISQPGWGAGGPHDAGHYKQYPEETGFFQRE-GAWDTPYGKFFLEWYSGM

LIEHGEKIVSSAEAIF------------------R-----------------G-------------------

------------------------------------------------------------------------

------------------------------------------------------------------------

------------------------------------------------------------------------

------------------------------------------------------------------------

------------------------------------------------------------------------

------------------------------------------------------------------------

------------------------------------------------------------------------

------------------------------------------------------------------------

-----------------------------------------------------------------TGA----

----------KLSGKVAGIHWHYGT--RSHSAELTAGYYNTRTRDGYVPIARMFGRHGVTLNFTCFEMK--D

EEQYPSSALCSPEGLLKQVTLAARKASVPLAGENALLRF-----DEGAHHQIITKSR---------------

--------LTVDGHD---------TADETIYEPMCSFTFLRMSEQLFRAENWRRFVQFVHQMDEGR------

---TFQP-W------------------EDRHSTADSHVYATTP----LIQEAEAAMLC--------------

---------------------------

>Azfi_s0159.g053957/1-547

-------------------------------MYICICIYICICIYVFMYVYIHMWYVCIYVLPCSSPQEKDP

LVPLVFLPFLFEKSENQTSIPSFLFLFL---FCFVYPDLRLKTTQNSPMASLSCSLSGSDALRGFLPDIVEK

KHPKVVIDCAHFPNPNANPEPKNNNSEPNPKHNPIPSSSI---G---------SSPLLAQEWFKKPSAVLWP

VSVETNHLFSFEESCVEKEEK---------------------------------------------------

------------------------------------------------------------------------

------------------------------------------------------------------HMYIEH

GLKGYDDGDGVIPVYVMLPLDS---INPKSG---TVKRPKAMSASMRAVQ-MAGVKGIVMEVWWGIVEKEGP

QNYNWCGYKELLDMAKQWGLKVQVILCFHQCWNIAGGDNCLIPLPPWVREEIEHNPDILFTNKR-----GMK

SYDYISFGCDTLP-ILQGRSPVQVYADFMRGFRDEFKDYI-GEVLVEIQVGLGPGGELQYPSCLDTSHISEF

PSFEEFMCYDK----------------------------------------------------FFLEWYSEQ

LLKHGERVLELAEAIF------------------R-----------------G-------------------

------------------------------------------------------------------------

------------------------------------------------------------------------

------------------------------------------------------------------------

------------------------------------------------------------------------

------------------------------------------------------------------------

------------------------------------------------------------------------

------------------------------------------------------------------------

------------------------------------------------------------------------

-----------------------------------------------------------------TGS----

----------KLSAKVPKALHHHGKEISSYFTHMVAGYL---YDDGYLRVMKMFGRHGVALNFTCLEMN--E

IKQQDP------------------------------------------------------------------

------------------------------------------SNQLFLSDAWRTFVKFVEDKSKRA------

------------------------------FSHRLSSCHVSTP----FLQQPSS------------------

---------------------------

>Sacu_v1.1_s0011.g005198/1-632

------------------------------------------------------------------------

--------MEKGAKSYAETGKNISPPLL---LSPCSVSFIHFLFLCQTSMAFPSSTLS-----------GSL

HATHPDAFRGIIHGIPEKQHAHHRRTSPFSHHGCIALNNS---S-----------GINVESFCSSPPSVHTT

EDHTWRIPSYQPPSALFSPY-----SHKLF------------------------------------------

------------------------------------------------------------------------

------------------------------------------------------DFHESSDHAPLEREHVYV

DTPTSIGPNGGVPVYVMMPLDT---VNPHNN---TLNRRRAMDASLRALK-SAGVEGIMVDVWWGAVEKN-P

QMYNWSGYRELIAMAKAYGLKMQTVMSFHQCGGN-VGDNCWIPLPPWVKEEIDHNPDLVYTDKD-----GRR

NYEYLSLGCDMLP-VLKGRSPVQVYSDFMRSFRDEFNDYL-GKVIVEIQVGLGPAGELRYPAYPERNNTWQF

PGIGEFECYDKYMLANLQAYAVARGKSEWGNGGPHDAGHYKQYPEETGFFQNG-GGWKSPYGVFFLEWYSGM

LLEHGERVLRSAEAIF------------------R-----------------G-------------------

------------------------------------------------------------------------

------------------------------------------------------------------------

------------------------------------------------------------------------

------------------------------------------------------------------------

------------------------------------------------------------------------

------------------------------------------------------------------------

------------------------------------------------------------------------

------------------------------------------------------------------------

-----------------------------------------------------------------SGA----

----------KLSGKVAGIYWHYGT--QSHPAELTAGYYNTRTRDGYIPIAQMFGRHGVTLNFTCFEMR--D

EEQYPPSARCSPEGLLKQVTLAARKASVPLAGENALLRF-----DEGAHRQIIAKSR---------------

--------LSDQ------------NGGQVIYEPMCSFTFLRMTEQLFQSDNWREFVRFVHQMAEGR------

---TSQS-W------------------ENRHSDTESHVYLMAP----LIQEAEVAMLCN-------------

---------------------------

>Mapoly0008s0172/7-441

------------------------------------------------------------------------

------------------------------------------------------------------------

------------------------------------------------------------------------

------------------------------------------------------------------------

------------------------------------------------------------------------

------------------------------------------------------------------------

----------GVPVYVMMPLDS---AEL-VK---SESGQWMLSLCLKRLK-DAGVEGVMMDVWWGLVEKTAP

GVYDWSLYDRLVKVIQDNDLKLQAVMSFHQCGGN-IGDGVYIPLPDWVLEESHRNPDIFYTDQ------GWR

NPEYISLGCDTLP-VIRGRTPVQCYSDYMTSFRENYKSVL-GDVIVEIQVGMGPAGELRYPAYPQSENQWTF

PGIGELQCYDKYMVTSLQAKATSVGKPQYG-KIPTDAGTYNQLPQDTGFWKDN-GTWKTAYGQFFLSWYSDQ

LLAHGERILTVANRVF--------------------------------------------------------

------------------------------------------------------------------------

------------------------------------------------------------------------

------------------------------------------------------------------------

------------------------------------------------------------------------

------------------------------------------------------------------------

------------------------------------------------------------------------

------------------------------------------------------------------------

------------------------------------------------------------------------

-------------------------------------------------------------------V----

----------VLSAKISGIHWHYKT--PSHAAECGVGYYNTEKRNGYLPFARLFARQNTVFNFTCLEMK--D

SQQ--DVANSSPETLIKQVAASCRKADIRMSGENALAVF-----DDASYDQISETAS---------------

--------LQFDGD------------------NIVSFTYLRLSEDLFTGENWSNFEEFVAKMVSSA------

---------------------------SVNQSGFRSKI----------------------------------

---------------------------

>AaBONN_Sc2ySwM_344.3169.1/1-621

------------------------------------------------------------------------

-----------MAFACMSSSGSLFVRLS---GSGRSFPAELEPSSSIRDEACSPRCLA--------------

AHFRPVPIQSLPRLPKEPDFSSSLTTSSISSSC----------------------SPFPEDWWRSPPQLQTA

RLTGSRVDVSELRVSRMSDTEGMISGHTAS------------------------------------------

------------------------------------------------------------------------

------------------------------------------------------EWQRTSHDW---KEFAFH

ETPTSRGTTGGVPVYVMLPLDS---VNM-NN---TLNRRRALNASLLALK-SAGVEGIMMDVWWGIVEKDVP

GQYNWSAYKELVDLVRKYGLKVQAVMSFHQCGGN-VGDSCFIPLPQWVTDEIHQNPDIVYTDKS-----SRR

NFEYLSLGCDVLP-VLRGRSPVQAYADFMRSFRDTFEPLL-GEVINEIQVGMGPAGELRYPAYPESNGRWRF

PGIGEFQCYDKYMMASLRACAEAVGRSHWGLGGPHDAGNYNQWPDETGFFHRD-GSWNSPYGQFFLEWYSEM

LIAHGERILAAAEGIF------------------R-----------------G-------------------

------------------------------------------------------------------------

------------------------------------------------------------------------

------------------------------------------------------------------------

------------------------------------------------------------------------

------------------------------------------------------------------------

------------------------------------------------------------------------

------------------------------------------------------------------------

------------------------------------------------------------------------

-----------------------------------------------------------------TGA----

----------HISGKVAGIHWHYGT--RSHAAELTAGYYNTRIRDGYLPIAHMFARHGVTLNFTCIEMR--D

TEQ-PPHALCSPEGLLRQIAVAARTAGIRLSGENALPRF-----DESAHDQVVRKSR---------------

--------LQVHED----------GSSLTAFEPMCSFTFLRMCERLFHNDNWRVFVPFVMHMEEGR------

---TFQP-W------------------EEEHQNTVSHVHATNP----LIQEAAAALMYH-------------

---------------------------

>AaBONN_Sc2ySwM_362.2644.1/1-479

------------------------------------------------------------------------

------------------------------------------------------------------------

MADFPDPAQQEEQQQSKPG------------------------------------------WIRASGM----

------------------------------------------------------------------------

------------------------------------------------------------------------

------------------------------------------------------------------------

---------AGVPVFVMLPLDS---VQM-LE---SGSGRWALSASLVALQ-GAGVKGVMMDVWWGIVEKDVP

GDYDWSGYAKLIELVRFCGLKIQAVMSFHQCGSN-VGDSCSIPLPSWVLEEVDRNAEIVFTDKS-----QNR

NYEYISLGCDLLP-VLRGRSPVEVYADYMTSFRDTFVELI-GDVIVEIQVGMGPAGELRYPSYPECNGLWRF

PGIGEFQCYDKYMLEDLKTRAISIGKDDWGVGGPAGTGAYNQLPSETEFFQKN-GTWSTPYGHFFLEWYSDL

LLAHGERLLSAAHKIF------------------Q-----------------S-------------------

------------------------------------------------------------------------

------------------------------------------------------------------------

------------------------------------------------------------------------

------------------------------------------------------------------------

------------------------------------------------------------------------

------------------------------------------------------------------------

------------------------------------------------------------------------

------------------------------------------------------------------------

-----------------------------------------------------------------TGA----

----------HISGKVSGIHWQHRT--SSHAAEVTAGYYNTETRDGYLPIASLFAKFGAILNFTCIEMK--D

AEQ-PAEAQSSPEKLIEQVAAAAGSAGIALSGENALSRF-----DEVAFDQVLKQAR---------------

--------LPSAGD----------KGVLEKPDRLCSFTYLRMCEDLFEPENWNIFVSFVKNMHQ--------

---------------------------VEGAQNQMLPTKSTL------------------------------

---------------------------

**Chaperonin 60B2**

cov pid  **1** **[ . . . . : . . .** **80**

1 Selmo_165043/1-607 100.0% 100.0%  **---MAASHAAAASLNS-----------------------------PSTALFSSSFAQSTAF-GRVSFQDFRQRSKSGGLR**

2 Selmo_168153/1-550 90.1% 79.6%  **--------------------------------------------------------------------------------**

3 AT3G13470/1-596 97.5% 74.8%  **---MASTFTATSSLGSLLA--------------------------PNAIKLSSATSISSSSFGRRHNVCVR---------**

4 Bradi3g00480/1-598 97.4% 74.8%  **---MASPFGGTSTCGLKA---------------------------AAPAGFATKKQLSLVLPPCVSLPQ-----------**

5 Bradi3g28070/1-574 93.9% 42.4%  **MYRAAVSLASKARQAG-----------------------------SSARQVGSRLAWSRNY-------------------**

6 Bradi5g11240/1-586 94.7% 55.2%  **---MSRMPPPPPPLSSK----------------------------PPTLPFSP---------------------------**

7 Cre07.g339150/1-577 94.7% 62.5%  **---MAGTQMRSFQAKS-----------------------------AASARGAVR--------------------------**

8 Cre17.g741450/1-581 95.1% 59.7%  **---MSSTILGRVTGKT-----------------------------AAKGGVSRS--------------------------**

9 Mapoly0001s0499/1-613 99.0% 75.3%  **---MASAVGSMAAVNAAASLKPVEISA------------------SKSVKNAQSLASSSAF-SRLSFNDVHRRQSRSAV-**

10 Pp3c16_4660/1-604 97.9% 73.9%  **---MAAASMAIAGSAATASLRV-----------------------SSESRAPSSLSSSSAI-GRVGFSGGLN--------**

11 Pp3c27_5110/1-604 97.9% 74.4%  **---MAATTMAMAGSAATSSLRV-----------------------AAENKAASSLSSSAAF-GRVNFGGVAK--------**

12 Pp3c6_20400/1-601 97.7% 73.7%  **---MAATNMLMAGSAATASL-------------------------RIESPAASSLSSSAAV-GRVGFVLPA---------**

13 Azfi_s0217.g058536/1-592 97.2% 75.0%  **---MASLSFSSSSFTS-----------------------------FPQRQLLLSNNSPSSFPSSSQL-------------**

14 Azfi_s0001.g000356/1-608 98.0% 75.0%  **---MAAMIAAKLSSLSATAPLSERRCL-----------------APSQKALSSSASISSAF-PQPKLSKSTK--------**

15 Azfi_s0029.g024060/1-637 99.3% 72.7%  **---MASSSSAAALCTPSSSSSPSFSSALHLLRASSGRRTRRAASLPPTTSISISLSFSSSFSSSLSLREGHGNSSSSCCR**

16 Sacu_v1.1_s0182.g024885/1-614 98.8% 74.2%  **---MASTYRLSLASSAALSDPRSPSS-------------------ACCPRRSPALSFSSSF---ASLAIAGTRSGNSSRT**

17 Sacu_v1.1_s0110.g020651/1-614 99.8% 76.1%  **---MATIVAAKGLSLSPSPSP------------------------SSPLPIQKSLSSSASF-ASFHRRQLLLSNRSPGML**

18 Sacu_v1.1_s0065.g016064/1-624 98.7% 74.2%  **---MAAASISAVGASAPGNAATIT---------------------ECSRHGISSAASIVSFSQKDFFSSNAANL------**

19 CHBRA163g00060/1-608 94.2% 66.4%  **---MASTTAAAAAVAPIAGVASSSKTSRKEQRRAARQATS--SSWICQRTGLRVTSTASSFLSSPTFSSSSLAVRDNGHH**

20 CHBRA278g00260/1-644 99.3% 66.4%  **MASTTAAAGAAAAIAPTAGIAASPSARRDHHRVSSRQSASPCSSCICQRTGLRVSSTASSFLASPTFSSSSSLSLRGAQQ**

21 AaBONN_Sc2ySwM_228.3832.1/1-560 90.8% 44.8%  **---MAANAMLANGAALSPVA-------------------------APSCSSSPSLKQGRRLSGCWQGRNQLAQKASGLKA**

22 AaBONN_Sc2ySwM_117.2749.4/1-539 87.5% 64.1%  **---MAAAVGSVAALSAPAAAPAKA---------------------SGSGASAQSYLSSSAF-SRLSFKDLNSRKAGRN--**

23 AaBONN_Sc2ySwM_368.227.1/1-581 94.7% 43.2%  **---MNRAAAAIASQLSRRGA-------------------------RARLRDASRQQWSRNY-------------------**

cov pid  **81**  **. 1 . . . . : .** **160**

1 Selmo_165043/1-607 100.0% 100.0%  **---CRAPVVRTR----AAKQLFFNKDGSAMKKLQAGANKVADLVGVTLGPKGRNVVLESKYGSPKIVNDGVTVAKEVELE**

2 Selmo_168153/1-550 90.1% 79.6%  **----------------MAKELVFNKDGQATKRLQAGVNKLADLVGVTLGPKGRNVVLESKYGSPKIINDGVTVTKEIELE**

3 AT3G13470/1-596 97.5% 74.8%  **---RSRPAIVC-----AAKELHFNKDGTTIRKLQTGVNKLADLVGVTLGPKGRNVVLESKYGSPRIVNDGVTVAREVELE**

4 Bradi3g00480/1-598 97.4% 74.8%  **---KFRPRRKCSFRVNAAKELYFNKDGSAIKKLQNGVNKLADLVGVTLGPKGRNVVLESKYGSPKIVNDGVTVAKEVELE**

5 Bradi3g28070/1-574 93.9% 42.4%  **----------------AAKDIKFGVEARAL-MLR-GVEELADAVKVTMGPKGRTVIIEQSFGAPKVTKDGVTVAKSIEFK**

6 Bradi5g11240/1-586 94.7% 55.2%  **---KKPPPMP------VYKDLHFNHDLSATKKLQAGVDLVARLVGVTLGPKGRNVVLSNKYGPPKIVNDGETVLKEIELE**

7 Cre07.g339150/1-577 94.7% 62.5%  **---GRRSVVVQ-----AAKELHFNRNMEALKKMQAGVDKLATVVGVTIGPKGRNVVLESKFGSPKIVNDGVTIAREVELE**

8 Cre17.g741450/1-581 95.1% 59.7%  **---RRSVVVR------AAKELHFNKDMQALKRMQAGVDKLATVVGVTIGPKGRNVVLESKFGAPKIVNDGVTIAREVELS**

9 Mapoly0001s0499/1-613 99.0% 75.3%  **---ARKGAVRTNA---MAKELYFNKDGSATKKMQAGVNKLADLVGVTLGPKGRNVVLESKYGSPKIVNDGVTVAKEVELE**

10 Pp3c16_4660/1-604 97.9% 73.9%  **---QRRQRVAHRTTVKAAKELHFNKDGSAIKKMQAGVDKLADLVGVTLGPKGRNVVLESKYGSPKIVNDGVTVAKEVELE**

11 Pp3c27_5110/1-604 97.9% 74.4%  **---LQRQRVAHRLPVKAAKELHFNKDGSAIKRMQAGVDKLADLVGVTLGPKGRNVVLESKYGSPKIVNDGVTVAKEVELE**

12 Pp3c6_20400/1-601 97.7% 73.7%  **---RQQRRVAQRMTVKAAKQLHFNKDGSAIKKMQAGVDKLADLVGVTLGPKGRNVVLESKYGSPKIVNDGVTVAKEVELE**

13 Azfi_s0217.g058536/1-592 97.2% 75.0%  **---QRSSALHTRS---MAKELHFNNDGSTMKKLQAGVNKLSDLVGVTLGPKGRNVVLESKYGAPKIVNDGVTVAKEVELE**

14 Azfi_s0001.g000356/1-608 98.0% 75.0%  **---KHNCAVSTRA---MAKELHFNKDGATMKKLQAGVDKLADLVGVTLGPKGRNVVLESKYGAPKIVNDGVTVAKEVELE**

15 Azfi_s0029.g024060/1-637 99.3% 72.7%  **VQRRRAARLQTWA---MAKELCFNTDGSTIKRLQAGVDKLADLVGVTLGPKGRNVVLESKFGAPKIVNDGVTVAKEVELE**

16 Sacu_v1.1_s0182.g024885/1-614 98.8% 74.2%  **THCRSRRAVQTWA---AAKELCFNTDGFTMKRLQAGVDKLADLVGVTLGPKGRNVVLESKYGAPKIVNDGVTVAKEVELE**

17 Sacu_v1.1_s0110.g020651/1-614 99.8% 76.1%  **CRRRHTPVSTRA----MAKELYFNKDGSTMKKLQAGVNKLSDLVGVTLGPKGRNVVLESKYGAPKIVNDGVTVAKEVELE**

18 Sacu_v1.1_s0065.g016064/1-624 98.7% 74.2%  **---RRKSIVQTRA---MAKELYFNKDGSTMKKLQAGVNKLSDLVGVTLGPKGRNVVLESKYGAPKIVNDGVTVAKEVELE**

19 CHBRA163g00060/1-608 94.2% 66.4%  **SHGAAPAGRRLTVTKSMAKELYFNKDGSAMKKMQAGVDKLANLVGVTLGPKGRNVVLESKYGAPKIVNDGVTVAKEVELE**

20 CHBRA278g00260/1-644 99.3% 66.4%  **RHGGASTGRGMPVTRSMAKELYFNKDGSAMKKMQVGADKLAELVGVTLGPKGRNVVLESKYGPPRIVNDGVTVAKEVELE**

21 AaBONN_Sc2ySwM_228.3832.1/1-560 90.8% 44.8%  **QAGSRRSVAARA----QAKDISF--DGKSRAALQAGIDKLADAVAVTLGPRGRNVVLD-EFGTPKVINDGVTIARAIELP**

22 AaBONN_Sc2ySwM_117.2749.4/1-539 87.5% 64.1%  **---LRAGAVQTR----AAKELYFNKDGSATKKLQAGVDKLANLVGVTLGPKGRNVVLESKYGSPKIVNDGVTVAKEVELE**

23 AaBONN_Sc2ySwM_368.227.1/1-581 94.7% 43.2%  **----------------AAKDIRFGVAARAL-MLQ-GVEELADAVKVTMGPKGRNVVIEQSFGAPKITKDGVTVAKNIEFK**

cov pid **161**  **. . . 2 . . . .** **240**

1 Selmo_165043/1-607 100.0% 100.0%  **DAVENIGARLVRQAASKTNDLAGDGTTTSVVLAQGLINEGVKLVAAGANPIQIARGIDKVIAGLVKELKTLSKEVE-DSE**

2 Selmo_168153/1-550 90.1% 79.6%  **DPVENIGVKLVRQAASKTNDLAGDGTTTSLILAQGLIAEGVKVVAAGSNPIQITRGIDKTIAALVKELKEISKEVE-DSE**

3 AT3G13470/1-596 97.5% 74.8%  **DPVENIGAKLVRQAAAKTNDLAGDGTTTSVVLAQGFIAEGVKVVAAGANPVLITRGIEKTAKALVNELKLMSKEVE-DSE**

4 Bradi3g00480/1-598 97.4% 74.8%  **DPVENIGAKLVRQAAAKTNDLAGDGTTTSVILAQGMITEGVKIVAAGANPVQIARGIEKTAKALVSELRKMSKEVE-DSE**

5 Bradi3g28070/1-574 93.9% 42.4%  **DRVKNVGASLVKQVANATNDTAGDGTTCATVLTKAIFSEGCKSVAAGMNAMDLRRGISMAVDDVVTNLKGMARMISTPEE**

6 Bradi5g11240/1-586 94.7% 55.2%  **DPLENLGVKLVRQAGARTNDIAGDGCTTSIILAQGLIAEGMKVLAAGINPVQIARGIGKTSDALVSELRSMSREIE-DHE**

7 Cre07.g339150/1-577 94.7% 62.5%  **DPVENIGAKLVRQAAARTNDTAGDGTTTATVLSAAFIAEGMKIVAAGTNPVQLTRGMEKTVNALVKELKAASSQVHSDKD**

8 Cre17.g741450/1-581 95.1% 59.7%  **DPVENIGATLVRQAAARTNDTAGDGTTTATVLSAAFIAEGMKIVSAGTNPVQLVRGMEKTVQELVKELRKMSSVVQTDKD**

9 Mapoly0001s0499/1-613 99.0% 75.3%  **DPVENIGAKLVRQAAAKTNDLAGDGTTTSVVLAQGLIAEGVKVVAAGANPIQIIRGIDKTIVALVHELKLLSKEVE-DSE**

10 Pp3c16_4660/1-604 97.9% 73.9%  **DPVENIGAKLVRQASAKTNDLAGDGTTTSVVLAQGLIAEGVKVVAAGANPVQITRGIDRTVIALVKELKNMSKEVE-DSE**

11 Pp3c27_5110/1-604 97.9% 74.4%  **DPVENIGAKLVRQASAKTNDLAGDGTTTSVVLAQGLIAEGVKVVAAGANPVQITRGIDKTVVALVKELKKMSKEVE-DSE**

12 Pp3c6_20400/1-601 97.7% 73.7%  **DPVENIGAKLVRQAAAKTNDLAGDGTTTSVVLAQGLIAEGVKVVAAGANPVQITRGIEKTVAALVKELKKLSKEVE-DSE**

13 Azfi_s0217.g058536/1-592 97.2% 75.0%  **DPVENIGAKLVRQAAAKTNDLAGDGTTTSVVLAQGLIAEGVKVVAAGANPIQITRGIEKTVKALVEELKKISKDVE-DSE**

14 Azfi_s0001.g000356/1-608 98.0% 75.0%  **DSVENIGAKLVRQAAAKTNDLAGDGTTTSVVLAQGLIAEGVKVIAAGANPIQITRGIEKTVKALVEELKKMSKDVE-DSE**

15 Azfi_s0029.g024060/1-637 99.3% 72.7%  **DPVENIGARLVRQAAAKTNDLAGDGTTTSVVLAQGLIAEGVKVVAAGANPIEIARGIEKTVKALVEELKKLSKEVE-DSE**

16 Sacu_v1.1_s0182.g024885/1-614 98.8% 74.2%  **DPVENIGAKLVRQAAAKTNDLAGDGTTTSVVLAQGLITEGVKVVAAGANPIQISRGIEKTVKALVTELKALSKEVE-DSE**

17 Sacu_v1.1_s0110.g020651/1-614 99.8% 76.1%  **DPVENIGAKLVRQAAAKTNDLAGDGTTTSVVLAQGLIAEGVKVVAAGANPIQITRGIDKTVKALVEELKKISKDVE-DSE**

18 Sacu_v1.1_s0065.g016064/1-624 98.7% 74.2%  **DPVENIGAKLVRQAAAKTNDLAGDGTTTSVVLAQGLIAEGVKVVAAGANPIQITRGIEKTVKHLVEELKALSKDVE-DSE**

19 CHBRA163g00060/1-608 94.2% 66.4%  **DPVENIGAKLVRQAASKTNDLAGDGTTTSVVLAQGLIAEGMKVVAAGANPVQITRGIDKTVAALVKELKAMSKEVE-DKE**

20 CHBRA278g00260/1-644 99.3% 66.4%  **DPVENIGAKLVRQAASKTNDLAGDGTTTSVILAQGLIAEGMKVVAAGANPVQITRGIDKTIAALVNELKAMSKEVE-DKE**

21 AaBONN_Sc2ySwM_228.3832.1/1-560 90.8% 44.8%  **DAMENAGAALIREVASKTNDSAGDGTTTACVLARELIRFGLLNVTSGASPVAIKKGIDKTVTALVKKLAEKARPVQGRED**

22 AaBONN_Sc2ySwM_117.2749.4/1-539 87.5% 64.1%  **DPVENIGAKLVRQAAAKTNDLAGDGTTTSVVLAQGLIAEGVKVVAAGANPIQITRGIEKTVAALVQELKNIAKDVE-DSE**

23 AaBONN_Sc2ySwM_368.227.1/1-581 94.7% 43.2%  **DKIKNLGASLVKQVANATNDVAGDGTTCATVLARAIYSEGCKSVAAGMNAMDLRRGINLAVDAVVQNLKSRAKMISTSEE**

cov pid **241**  **: . . . . 3 . .** **320**

1 Selmo_165043/1-607 100.0% 100.0%  **LADVAAVSAGNNYEIGDMIAEAMKRVGRKGVVTLEEGRSAESTLQFVEGMQFDRGYISPYFVTDTEKMAVEYQNCKLLLV**

2 Selmo_168153/1-550 90.1% 79.6%  **LADVATVSAGNNDEVGKMIAEAMNKVGRKGVVTLEEGKSAENTLHVVEGMQFERGYMSPYFVTDTDKMAVEYDNCKLLLV**

3 AT3G13470/1-596 97.5% 74.8%  **LADVAAVSAGNNHEVGSMIAEAMSKVGRKGVVTLEEGKSAENNLYVVEGMQFDRGYISPYFVTDSEKMSVEYDNCKLLLV**

4 Bradi3g00480/1-598 97.4% 74.8%  **LADVAAVSAGNNYEVGNMIAEAMSKVGRQGVVTLEEGKSAENNLYVVEGMQFDRGYISPYFVTDSEKMTVEYENCKLLLV**

5 Bradi3g28070/1-574 93.9% 42.4%  **IAQVGTISANGEREIGELIAKAMEKVGKEGVITIADGNTLYNELEVVEGMKIDRGYISPYFITNPKTQKCEMEDPMILIH**

6 Bradi5g11240/1-586 94.7% 55.2%  **IAHVAAVSAGNDYAVGNMISDAFKRVGRKGMVRIENGRGTENSLEIVEGMQFERGYLSPYFVTNHANMSVEFTDCKILLV**

7 Cre07.g339150/1-577 94.7% 62.5%  **LSNVASVSAGGNPDVGKLISDAMAKVGRQGVVTMEESKTAEDALIFVEGMQFDRGYYSPYFVTDPERMLAEYENCRILLV**

8 Cre17.g741450/1-581 95.1% 59.7%  **LANVACVSAGGNTDIGSLISDAMAKVGRTGVVTMEEGKTAEDQLVFVEGMQFERGYTSPYFVTDPERMICEYENCKILLV**

9 Mapoly0001s0499/1-613 99.0% 75.3%  **LADVAAVSAGNNEEVGAMIADAMSKVGRKGVVTLEEGKSAENSLYVVEGMQFERGYISPYFVTDPEKMTVEYDNCKLLLV**

10 Pp3c16_4660/1-604 97.9% 73.9%  **LADVAAVSAGNNPEIGQMIAEAMGKVGRKGVVTMEEGRSAENHLYVVEGMQFDRGYISPYFVTDPEKMSVEYDNCRLLLV**

11 Pp3c27_5110/1-604 97.9% 74.4%  **LADVAAVSAGNNPEIGQMIAEAMSKVGRKGVVTLEEGKSAENNLYVVEGMQFDRGYISPYFVTDPEKMDVEYDNCRLLLV**

12 Pp3c6_20400/1-601 97.7% 73.7%  **LADVAAVSAGNNPEVGQMIAEAMSKVGRRGVVTLEEGKSSENSLYVVEGMQFDRGYISPYFVTDPEKMSVEYDNCRLLLV**

13 Azfi_s0217.g058536/1-592 97.2% 75.0%  **LADVAAVSAGNNYEVGNMIAEAMSKVGRKGVVTLEEGKSAENNLVVVEGMQFDRGYISPYFVTDTEKMIIEYANCKLLLV**

14 Azfi_s0001.g000356/1-608 98.0% 75.0%  **LADVAAVSAGNNYEVGNMIAEAMSKVGRKGVVTLEEGRSAENSLIVVEGMQFDRGYISPYFVTDTEKMIIEYANCKLFLV**

15 Azfi_s0029.g024060/1-637 99.3% 72.7%  **LVDVAAVSAGNNYEIGTMIAEAMQKVGRKGVVTLEEGKSAENRLHVVEGMQFDRGYISPYFVTDNEKMIVEYTTCKLLLV**

16 Sacu_v1.1_s0182.g024885/1-614 98.8% 74.2%  **LADVAAVSAGNNYEVGNMIADAMSKVGRKGVVTLEEGRSAENNLYVVEGMQFDRGYISPYFVTDNEKMVAEYTNCKLLLV**

17 Sacu_v1.1_s0110.g020651/1-614 99.8% 76.1%  **LADVAAVSAGNNYEVGNMIAEAMNKVGRKGVVTLEEGRSAENILTVVEGMQFDRGYISPYFVTDTEKMVVEYTNCKLLLV**

18 Sacu_v1.1_s0065.g016064/1-624 98.7% 74.2%  **LADVAAVSAGNNYEVGNMIAEAMTRVGRKGVVTLEEGRSAENSLHVVEGMQFDRGYISPYFVTDTEKMIVEYANCKLLLV**

19 CHBRA163g00060/1-608 94.2% 66.4%  **LADVAAVSAGNNYEVGNMIAEAMAKVGRSGVITLEEAKSVENNLYVVEGMQFDRGYISPYFVTDPERMCAEYENCKLLLV**

20 CHBRA278g00260/1-644 99.3% 66.4%  **LADVAAVSAGNNYEVGNMIAEAMAKVGRAGVITLEEARSVENNLYVVEGMQFDRGYISPYFVTDPERMIVEYENCKLLLV**

21 AaBONN_Sc2ySwM_228.3832.1/1-560 90.8% 44.8%  **IKAIAAISAGNDDFVGTMIADAIDKVGSDGVLSIESSSSFETTVEVEEGMEIDRGYISPQFVTNPEKLLVEFENARVLVT**

22 AaBONN_Sc2ySwM_117.2749.4/1-539 87.5% 64.1%  **LADVAAVSAGNNEEVGLMIAEAMSKVGRKGVVTLEEGKSAENNLYVVEGMQFDRGYISPYFVTDSEKMSVEYDNCKLLIV**

23 AaBONN_Sc2ySwM_368.227.1/1-581 94.7% 43.2%  **IAQVATISANGEREIGDMIARAMEKVGKDGIITVADGKTLHNEIEVVEGMKLDRGYISPYFVTNPKTQKVELENPLILIF**

cov pid **321**  **. . : . . . . 4** **400**

1 Selmo_165043/1-607 100.0% 100.0%  **DKKITNARDIVSILEDAIRAGSPLLIIAEDIEQEALATLVVNRIRGNLKVAAIKAPGFGERKSQYLEDIATLTGGT----**

2 Selmo_168153/1-550 90.1% 79.6%  **DRKIATARDVIGILEDAIRGGYPLMIMAEEIEQEALATLVVNRLRGSLKIVAIKAPGFGERKTQYLEDIAILTGGT----**

3 AT3G13470/1-596 97.5% 74.8%  **DKKVTNARDLVGVLEDAIRGGYPILIIAEDIEQEALATLVVNKLRGTLKIAALKAPGFGERKSQYLDDIAILTGAT----**

4 Bradi3g00480/1-598 97.4% 74.8%  **DKKINNARDLITILEDAIKSGYPILIIAEDIEQEALATLVVNRLRGALKIAAIKAPGFGERKSQYLDDIATLTGGT----**

5 Bradi3g28070/1-574 93.9% 42.4%  **EKKISNLPAFVKVLELAHAKQRPLLVVAEDLESEALGTLILNKLRVGLKLCAIKAPGFGENRKANLQDLAILTGGE----**

6 Bradi5g11240/1-586 94.7% 55.2%  **DKIITDPRELLKVCFSAVKEDFPLVIIAEDVEEEALATLTRNKLSGMIKVAAIEALSFGEQKTQCLEDIAIMTGGT----**

7 Cre07.g339150/1-577 94.7% 62.5%  **DKKISTARDIIGILEAAIRGNYPLLIMAEDVEQEALATLVVNKLRGTLKVVAVKAPGFGERKSSYLEDIAILTGGT----**

8 Cre17.g741450/1-581 95.1% 59.7%  **DKKISTARDIITILESAIRGNYPLLIMAEEVEQEALATLVVNKLRGTLKVVAIKAPGFGERRSSYLEDIAILTGGT----**

9 Mapoly0001s0499/1-613 99.0% 75.3%  **DKKITTARDMIGILEDAIRGGYPLLIIAEDIEQEALATLVVNKLRGSLKVAALKAPGFGERKSQYLDDIACLTAGT----**

10 Pp3c16_4660/1-604 97.9% 73.9%  **DKKITTARDMIGILEETIRGGFPLLIIAEDIEQEALSTLVVNKLRGSLKVAALKAPGFGERKSQYLDDIAILTSGT----**

11 Pp3c27_5110/1-604 97.9% 74.4%  **DKKITTARDMIGILEETIRGGFPLLIIAEDIEQEALSTLVVNKLRGSLKVAALKAPGFGERKSQYLDDIAILTNGT----**

12 Pp3c6_20400/1-601 97.7% 73.7%  **DKKIVTARDMIGILEESIRGGFPLLIIAEDIEQEALSTLVVNKLRGSLKVAALKAPGFGERKSQYLDDIAILTNAT----**

13 Azfi_s0217.g058536/1-592 97.2% 75.0%  **DKKITTARDIIGILEDAIKGGYPLLIIAEDIEQEALATLVVNKLRGSLKIAALKAPGFGERKSQYLDDIAILTGGT----**

14 Azfi_s0001.g000356/1-608 98.0% 75.0%  **DKKITTARDIIGILEDAIRGGYPLLIIAEDIEQEALATLVVNKLRGNLKIAALKAPGFGERKSQYLDDIAILTGGT----**

15 Azfi_s0029.g024060/1-637 99.3% 72.7%  **DKKITNARDIVGALEDAIRGGYPLLIIAEDIEQEALATLVVNKLRGALKIAALKAPGFGERKSQYLDDIAILTGGT----**

16 Sacu_v1.1_s0182.g024885/1-614 98.8% 74.2%  **DKKITNARDIIGILEDAIRGGHPILIIAEDIEQEALATLVVNKLRGALKVAALKAPGFGDRKSQYLDDIAILTGGT----**

17 Sacu_v1.1_s0110.g020651/1-614 99.8% 76.1%  **DKKITTARDIIGILEDVIRGGHPLLIIAEDIEQEALATLVVNKLRGSLKIAALKAPGFGERRSQYLDDIAILTAGT----**

18 Sacu_v1.1_s0065.g016064/1-624 98.7% 74.2%  **DKKITNARDIVNILEDAIRTGYPLLVIAEDIEQEALATLVVNKLRGSLKIAALKAPGFGERKSQYLDDIAILTGGKNVFV**

19 CHBRA163g00060/1-608 94.2% 66.4%  **DKKITTARDMIGILEDAIRNGYPLVIIAEDIEQEALATLVVNKLRGSLKVAALKAPGFGERKSQYLDDIAILTGGT----**

20 CHBRA278g00260/1-644 99.3% 66.4%  **DKKITTARDMIGILEDAIRNSYPLVIIAEDIEQEALSTLVVNKLRGTFKVAALKAPGFGERKSQYLDDIAILTGAT----**

21 AaBONN_Sc2ySwM_228.3832.1/1-560 90.8% 44.8%  **DQKITTIKDIIPVLEKTTQLSVPLLIIAEDVSGEALATLVVNKLRGVLQVAAIKAPGFGERRKALLQDIAIVTGAE----**

22 AaBONN_Sc2ySwM_117.2749.4/1-539 87.5% 64.1%  **DKKISTARDMIGILEDAIRGGYPLLIIAEDIEQEALATLVVNKLRGALKVAALKAPGFGERKSQYLDDIACLTGGT----**

23 AaBONN_Sc2ySwM_368.227.1/1-581 94.7% 43.2%  **EKKISGLQSVIPVLELSLKEQRPLLIIAEDVENEALATLIVNKIRAGVKVGAVKAPGFGENRKANLQDLAILVGGQ----**

cov pid **401**  **. . . . : . . .** **480**

1 Selmo_165043/1-607 100.0% 100.0%  **--VVKDEVGLQLDKVGSEVLGTASKVVITKDYSTIVGDGST-QEAVSKRVNQIKNQLEATEQDYEREKLNERIAKLSGGV**

2 Selmo_168153/1-550 90.1% 79.6%  **--VVREEVGLSLDKVGREVLGNAAKLVVTKDSTTIVGDGST-QEAVTKRVTQIRNLMEVAEQSYEKEKLNERIAKLSGGV**

3 AT3G13470/1-596 97.5% 74.8%  **--VIREEVGLSLDKAGKEVLGNASKVVLTKEMTTIVGDGTT-QEAVNKRVVQIRNLIEQAEQDYEKEKLNERIAKLSGGV**

4 Bradi3g00480/1-598 97.4% 74.8%  **--VIREEIGLSLDKADSEVLGTAAKVLVTKDTTTIVGDGST-QEEVSKRVTQIKNQIEAAEQEYEKEKLNERIAKLSGGV**

5 Bradi3g28070/1-574 93.9% 42.4%  **--VISEELGMSLENFDPQMLGTCKKVTVSKDDTVILDGAGD-KKNMEERAEQIRSAIEESTSDYDKEKLQERLAKLSGGI**

6 Bradi5g11240/1-586 94.7% 55.2%  **--VVRDDMGYTLEEAGKEVLGSASKVVIKKDSTLIVTDGST-LHAVEKRVAQIKGQIENSKERYQKKILGERIARLCGAI**

7 Cre07.g339150/1-577 94.7% 62.5%  **--VVKDELGITLEKATEEVLGLAAKVSISKEATTIVGDGRT-QQQVEGRVKQIRNLAAETEQEYEKEKLNERIARLSGGV**

8 Cre17.g741450/1-581 95.1% 59.7%  **--VVRDEMGVSLEQATDAVLGTAAKITITKERTTVVGDGST-AADVAARVKQIRNLQMQTDQDYEREKLQERIARLSGGV**

9 Mapoly0001s0499/1-613 99.0% 75.3%  **--VVRDEIGLSLDKVGKEVLGNAARVELTKDYTTIVGDGTT-QDAVSKRVVQIRNLIDNAEQEYEKEKLNERIAKLSGGV**

10 Pp3c16_4660/1-604 97.9% 73.9%  **--VIRDEIGLALDKVGTEVLGTAAKVVLTKDSTTIVGDGST-QDAVEKRVAQIRNLIEIAEQEYEKEKLNERIAKLSGGV**

11 Pp3c27_5110/1-604 97.9% 74.4%  **--VVRDELGLSLDKVGTEVLGTAAKVVLTKDSTTIVGDGST-QDAVEKRVAQIRNLIEIAEQEYEKEKLNERIAKLSGGV**

12 Pp3c6_20400/1-601 97.7% 73.7%  **--VIRDETGLALDKVGTEVLGTAAKVVLTKDATTIVGDGST-QDAVSRRVNQIRNLIEVAEQEYEKEKLNERIAKLSGGV**

13 Azfi_s0217.g058536/1-592 97.2% 75.0%  **--VIRDEIGLSLDKVGREVLGTAAKVVLTKDTTTIVGDGST-QDEVTKRVAQIKTLIEIAEQDYEREKLNERIAKLSGGV**

14 Azfi_s0001.g000356/1-608 98.0% 75.0%  **--VVRDEVGLALDKVGREVLGTAAKVVLTKDATTIVGDGST-QEEVTKRVAQIKSLIEVAEQEYEKEKLNERIAKLSGGV**

15 Azfi_s0029.g024060/1-637 99.3% 72.7%  **--VVRDAVGLSLDQVGGEVLGTAARVVLTKDTTTIVGDGST-QDVVSKRVAQIKTLLEDAEQDYEKEKLSERIAKLSGGV**

16 Sacu_v1.1_s0182.g024885/1-614 98.8% 74.2%  **--VVRDEVGLSLDKVGGEVLGTAARVVLTKETTTIVGDGST-QDAVNKRVTQIRSLVEAAEQDYEREKLNERIAKLSGGV**

17 Sacu_v1.1_s0110.g020651/1-614 99.8% 76.1%  **--VVRDELGLSLDKVGTEVLGTAAKVVLTKDATTIVGDGST-QEEVNKRVIQIKSLIEVAEQEYEREKLNERIAKLSGGV**

18 Sacu_v1.1_s0065.g016064/1-624 98.7% 74.2%  **GCVVRDEMGLSLDKVGREVLGTASKVVLTKDATTIVGDGST-QEEVNKRVAQIKNLIEVAEQDYEKEKLNERIAKLSGGV**

19 CHBRA163g00060/1-608 94.2% 66.4%  **--VVREEIGLSLDKVGREVLGTAAKIELTKDHTTIVGDGST-QDAVSARVKQIKALIEETEQDYEKEKLNERIAKLSGGV**

20 CHBRA278g00260/1-644 99.3% 66.4%  **--VVREETGLSLDRIGREVLGTAAKVELTKDHTTIVGDGST-QDAVNARVKQIKSLIEGTDVDYEKEKLNERVAKLSGGV**

21 AaBONN_Sc2ySwM_228.3832.1/1-560 90.8% 44.8%  **--FLASDLGMKVENTSSDQLGIARKIVISNGSTTIIADAAT-KDEIQARVAQIKKELAETDSVYDSEKLSERIAKLSGGV**

22 AaBONN_Sc2ySwM_117.2749.4/1-539 87.5% 64.1%  **--VVRDEVGLSLDKVGREVLGTAAKVEITKDATTIVGDGST-QDVVEKRVAQIRNLIENAEQEYEKEKLNERIAKLSGGV**

23 AaBONN_Sc2ySwM_368.227.1/1-581 94.7% 43.2%  **--LISEDLGLKLEKVTREQLGTAKKVTISKDDTIILDGGGD-KKAIEERCEQIVEATKDATSDYDKEKLQERLAKLSGGV**

cov pid **481**  **. 5 . . . . : .** **560**

1 Selmo_165043/1-607 100.0% 100.0%  **AVIQVGAQTETELKEKKLRVEDALNATKAAVEEGIVVGGGIALLRLSSKVDAIKDTLENDEQKIGAEIVRKALSYPVKLI**

2 Selmo_168153/1-550 90.1% 79.6%  **AVVTVGAQTETELKEKKLRVEDALNATKAAVEEGIVVGGGTALLRLSKKVDAIKETLENQEQKIGADIVKRALGYPIKLI**

3 AT3G13470/1-596 97.5% 74.8%  **AVIQVGAQTETELKEKKLRVEDALNATKAAVEEGIVVGGGCTLLRLASKVDAIKDTLENDEEKVGAEIVKRALSYPLKLI**

4 Bradi3g00480/1-598 97.4% 74.8%  **AVIQVGAQTETELKEKKLRVEDALNATKAAVEEGIVVGGGCTLLRLAAKVDAIKETLENDEQKIGAEIVRKSLSYPLKLI**

5 Bradi3g28070/1-574 93.9% 42.4%  **AVLKIGGASEAEVSEKKDRVTDALNATKAAVEEGIVPGGGVALLYASKALDKLQTA--NFDQKIGVQIIQNALKTPVYTI**

6 Bradi5g11240/1-586 94.7% 55.2%  **AIIQVGAQTVIEMKDKKLRIEDALNATMAAIDEGVVVGGGCSLLRLSKKIDIIKESLDNIEQKIGADIFKHALSYPTILI**

7 Cre07.g339150/1-577 94.7% 62.5%  **AIIQVGAQTETELKEKKLRVEDALNATKAAVEEGIVIGGGCTLLRLSQKVDSIKETLSNEEQKMGADIIKRALSYPIKLI**

8 Cre17.g741450/1-581 95.1% 59.7%  **AIIQVGAQTETELKEKKLRVEDALNATRAAVEEGVVPGGGCTLLRLSEKVDVIKRRMTDPEQQMGADIIKRALCYPIKLI**

9 Mapoly0001s0499/1-613 99.0% 75.3%  **AIIQVGAQTETELKEKKLRVEDALNATKAAVEEGIVVGGGSTLLRLAAKVDAIKATLENDEQKVGADIVKRALSYPLKLI**

10 Pp3c16_4660/1-604 97.9% 73.9%  **AIIQVGAQTETELKEKKLRVEDALNATKAAVEEGIVVGGGCTLLRLASKVDAIKESLDNDEQKVGADIVKRALCYPLKLI**

11 Pp3c27_5110/1-604 97.9% 74.4%  **AIIQVGAQTETELKEKKLRVEDALNATKAAVEEGIVVGGGCTLLRLASQVDAIKDTLDNDEQKVGSEIVRRALSYPLKLI**

12 Pp3c6_20400/1-601 97.7% 73.7%  **AIIQVGAQTETELKEKKLRVEDALNATKAAVEEGIVVGGGCTLLRLASKVSAIVSTLDNDEQRVGAEIVRRALCYPLKLI**

13 Azfi_s0217.g058536/1-592 97.2% 75.0%  **AILQVGAQTETELKEKKLRVEDALNATKAAVEEGIVVGGGSALLRLASKVDAIKETLDNYEQQVGADIVKRALSYPLKLI**

14 Azfi_s0001.g000356/1-608 98.0% 75.0%  **AILQVGAQTETELKEKKLRVEDALNATKAAVEEGIVVGGGSALLRLASKVDAIKETLDNYEQQVGADIVKRALSYPLKLI**

15 Azfi_s0029.g024060/1-637 99.3% 72.7%  **AIIQVGAQTETELKEKKLRVEDALNATKAAVEEGIVVGGGCALLRLAVKVDAIKETLEDREQQIGAEIVKRALRYPLKLI**

16 Sacu_v1.1_s0182.g024885/1-614 98.8% 74.2%  **AIIQVGAQTETELKEKKLRVEDALNATKAAVEEGIVVGGGCTLLRLAAKVDSIKETLDDSEQQIGADIVKRALSYPLKLI**

17 Sacu_v1.1_s0110.g020651/1-614 99.8% 76.1%  **AILQVGAQTETELKEKKLRVEDALNATKAAVEEGIVVGGGSALLRLSSKVDAIKATLENYEQQVGADIVKRALSYPLKLI**

18 Sacu_v1.1_s0065.g016064/1-624 98.7% 74.2%  **AIIQVGAQTETELKEKKLRVEDALNATKAAVEEGIVVGGGSALLRLAAKVDSIRDTLDNYEQQVGADIVKRALSYPLKLI**

19 CHBRA163g00060/1-608 94.2% 66.4%  **AIVQVGAQTETELKEKKLRVEDALNATKAAVEEGIVVGGGCTLLRLSSKVDAIQSTLENQEQKVRQS-------------**

20 CHBRA278g00260/1-644 99.3% 66.4%  **AIVQIGAQTETELKEKKLRVEDALNATKAAVEEGIVVGGGCTLLRLSAKVDAIESTLDNQEQKIGAGIVRRALTYPLKLI**

21 AaBONN_Sc2ySwM_228.3832.1/1-560 90.8% 44.8%  **AVIKVGAATETELEDRKLRIEDAKNATFAAIEEGIVPGGGAAMVHLSAFVPEIKAGIEDPEEKLGSDIVQKALLSPASII**

22 AaBONN_Sc2ySwM_117.2749.4/1-539 87.5% 64.1%  **AIIQVGAQTETELKEKKLRVEDALNATKAAVEEGIVVGGGCTLLRLAAKVEGIKSSLDNDEQRVGFFCLLSGLF------**

23 AaBONN_Sc2ySwM_368.227.1/1-581 94.7% 43.2%  **AVLKIGGASEVEVAEKKDRVTDALNATKAAVEEGIVPGGGVALLYASRVLEKLQTA--NFDQKVGVQIIANALKMPTYTI**

cov pid **561**  **. . . 6 . . . .** **640**

1 Selmo_165043/1-607 100.0% 100.0%  **AKNAGVNGSVIVEKVLAD-DNFNHG-YNAATGAFENLMAAGIIDPTKVVRCCLEHAGSVARTFLTSDAVVVSIPE---PE**

2 Selmo_168153/1-550 90.1% 79.6%  **AKNAGVNGSVVVEKVLAN-DNFNFG-YNAATGTYEDLMAAGIIDPTKVVRCCLEHAGSVAKTFLTSDVVVVDIKEEFIPT**

3 AT3G13470/1-596 97.5% 74.8%  **AKNAGVNGSVVSEKVLAN-DNVKFG-YNAATGKYEDLMAAGIIDPTKVVRCCLEHAASVAKTFLMSDCVVVEIPE---PE**

4 Bradi3g00480/1-598 97.4% 74.8%  **AKNAGVNGSVVTEKVLAN-DNFRYG-YNAATGKYEDLMAAGIIDPTKVVRCCLEHAASVAKTFITSDAVVVDIKE---AE**

5 Bradi3g28070/1-574 93.9% 42.4%  **ASNAGVEGAVVVGKLLEQ-DNTDLG-YDAAKGEYVDMVKVGIIDPLKVIRTALVDAASVSSLMTTTESIIVEIPK---EE**

6 Bradi5g11240/1-586 94.7% 55.2%  **ANNAGMSGKFVIQKVLSN-ENTNYG-YNAANDCYEDLMAAGILDPSKVVRCCIEHAAVVARSFLTSDVVVVEAKE---SK**

7 Cre07.g339150/1-577 94.7% 62.5%  **ANNAGTNGSVVMQRVMDNIDQPYYG-YNAATDTFEDLMEAGIIDPTKVVRCSLENAVSVAKTFLLADVVVTEIPE---KE**

8 Cre17.g741450/1-581 95.1% 59.7%  **AQNAGVNGSVVMNEVMKNLDRPHYG-YNAATDSFENLMETGIIDPSKVVRCSMENAVSVAKTFLLADVVVTELKE--IEA**

9 Mapoly0001s0499/1-613 99.0% 75.3%  **AKNAGVNGSVVVEKVLSN-DNPNWG-YNAATGVYEDLMAAGIIDPSKVVRCCLENASSVAKTFLTSDVVVVEIKE---PE**

10 Pp3c16_4660/1-604 97.9% 73.9%  **AKNAGVNGSVVVEKVLAN-ENSSFG-YNAATGVYEDLMAAGIIDPAKVVRCCLEHAASVAKIFLTSDVVVTEIKE---PE**

11 Pp3c27_5110/1-604 97.9% 74.4%  **AKNAGVNGSVVVEKVLAN-ENPAFG-YNAATGEYQDLMAAGIIDPAKVVRCCLEHAASVAKIFLTSDVVVTEIKE---PE**

12 Pp3c6_20400/1-601 97.7% 73.7%  **AKNAGVNGSVVVEKVLAN-SNPSFG-YNAATGVYEDLMAAGIIDPTKVVRCCLEHAASVAKIFLTSDVVVTEIKE---AE**

13 Azfi_s0217.g058536/1-592 97.2% 75.0%  **AKNAGVNGSVVVERVLSN-DNLKYG-YNAATGAYEDLMAAGIIDPTKVVRCCLEHAASVAKTFLTSDVVVVEIKE---PE**

14 Azfi_s0001.g000356/1-608 98.0% 75.0%  **AKNAGVNGSVVVEKVLSN-DNPKYG-YNAATGKYEDLVAAGIIDPTKVVRCCLEHAASVAKTFLTCDVVVVEIKE---PE**

15 Azfi_s0029.g024060/1-637 99.3% 72.7%  **AKNAGVNGSVVVEKVLAN-ENFKFG-YNAATGAYEDLMAAGIIDPTKVVRCCLEHAGSVAKTFLTSDAVVYEIKE---PE**

16 Sacu_v1.1_s0182.g024885/1-614 98.8% 74.2%  **AKNAGVNGSVVVEKVLSN-DNFRFG-YNAATGVYEDLMSAGIIDPTKVVRCCLEHAASVAKTFLTSDAVVVEIKE---PE**

17 Sacu_v1.1_s0110.g020651/1-614 99.8% 76.1%  **AKNAGVNGSVVVEKVLSN-ENPKYG-YNAATGKYEDLMAAGIIDPTKVVRCCLEHAASVAKTFLTSDVVVVEIKE---PE**

18 Sacu_v1.1_s0065.g016064/1-624 98.7% 74.2%  **AKNAGVNGSVVVEKVLAN-DNPNFG-YNAATGTYENLITAGIIDPTKVVRCCLEHAASVAKTFLTSDVVVVEIKE---PE**

19 CHBRA163g00060/1-608 94.2% 66.4%  **-------------------DNINYG-YNAATGVYEDLMAAGIIDPTKVVRCCLEHAGSVAKTFLTSDCVVVDIKE---PE**

20 CHBRA278g00260/1-644 99.3% 66.4%  **AKNAGVNGGVVVQKVKQN-DSVNFG-FNAATGVYEDLMAAGIIDPTKVVRCCLEHAASVAKTFLMSDCVVVEIKE---PE**

21 AaBONN_Sc2ySwM_228.3832.1/1-560 90.8% 44.8%  **ASNSGVEGAVVIEKILSS--DWEFG-YNAMT---DNSTSPG-------------------------------------DE**

22 AaBONN_Sc2ySwM_117.2749.4/1-539 87.5% 64.1%  **-------------RTLYS-DFSRFP-W-----------------------------------FITC------------PG**

23 AaBONN_Sc2ySwM_368.227.1/1-581 94.7% 43.2%  **ASNAGVEGAVVVGKLLEQ-ENPDIG-YDAAKAEYVDMIKAGIVDPVKVIRTALVDAASVSSLMTTTEAIIVESLK---ED**

cov pid **641**  **: . ]** **670**

1 Selmo_165043/1-607 100.0% 100.0%  **PAVPA-GNPMDNSGTKSLSST---------**

2 Selmo_168153/1-550 90.1% 79.6%  **GA-AQ-GMAMDNS---GYPPY---------**

3 AT3G13470/1-596 97.5% 74.8%  **PV-PA-GNPMDNS---GY-GY---------**

4 Bradi3g00480/1-598 97.4% 74.8%  **QA-PA-ANPMAGS---GY-GF---------**

5 Bradi3g28070/1-574 93.9% 42.4%  **KEAPA----MGGMGGMDY------------**

6 Bradi5g11240/1-586 94.7% 55.2%  **PVRIRPPMPPRNL----IPPIPASVSSIRV**

7 Cre07.g339150/1-577 94.7% 62.5%  **KA-PA---PAAGGGDYDY------------**

8 Cre17.g741450/1-581 95.1% 59.7%  **GAKPNPVAPGAAGFGGGL------------**

9 Mapoly0001s0499/1-613 99.0% 75.3%  **PAQPA--NPMDNS---GY-GY---------**

10 Pp3c16_4660/1-604 97.9% 73.9%  **PV-QA-GNPMDAS---GY-GY---------**

11 Pp3c27_5110/1-604 97.9% 74.4%  **PV-PA-GNPMDAS---GY-GY---------**

12 Pp3c6_20400/1-601 97.7% 73.7%  **VA-VA-GNPMDAS---GY-GY---------**

13 Azfi_s0217.g058536/1-592 97.2% 75.0%  **PSVPA-GNPMDNS---GY-GY---------**

14 Azfi_s0001.g000356/1-608 98.0% 75.0%  **GAAPA-GNPMDNS---GY-GY---------**

15 Azfi_s0029.g024060/1-637 99.3% 72.7%  **SPAVP-SNPMDNS---GY-GY---------**

16 Sacu_v1.1_s0182.g024885/1-614 98.8% 74.2%  **VPAVA-GNPMDNSGKQ--------------**

17 Sacu_v1.1_s0110.g020651/1-614 99.8% 76.1%  **PAVPA-GNPMDNSGTTGY-GY---------**

18 Sacu_v1.1_s0065.g016064/1-624 98.7% 74.2%  **PA-PA-ANPMDNSERSNV-NINFTVFLYIK**

19 CHBRA163g00060/1-608 94.2% 66.4%  **PAGAAMGNPMDAS---GY-GY---------**

20 CHBRA278g00260/1-644 99.3% 66.4%  **APAAAMPNSMDPS---GY-GY---------**

21 AaBONN_Sc2ySwM_228.3832.1/1-560 90.8% 44.8%  **HVEVP-------------------------**

22 AaBONN_Sc2ySwM_117.2749.4/1-539 87.5% 64.1%  **WC----GHREESS-----------------**

23 AaBONN_Sc2ySwM_368.227.1/1-581 94.7% 43.2%  **KELAAGRGGMGGMGGMDY-DY---------**

>Selmo_165043/1-607

---MAASHAAAASLNS-----------------------------PSTALFSSSFAQSTAF-GRVSFQDFRQ

RSKSGGLR---CRAPVVRTR----AAKQLFFNKDGSAMKKLQAGANKVADLVGVTLGPKGRNVVLESKYGSP

KIVNDGVTVAKEVELEDAVENIGARLVRQAASKTNDLAGDGTTTSVVLAQGLINEGVKLVAAGANPIQIARG

IDKVIAGLVKELKTLSKEVE-DSELADVAAVSAGNNYEIGDMIAEAMKRVGRKGVVTLEEGRSAESTLQFVE

GMQFDRGYISPYFVTDTEKMAVEYQNCKLLLVDKKITNARDIVSILEDAIRAGSPLLIIAEDIEQEALATLV

VNRIRGNLKVAAIKAPGFGERKSQYLEDIATLTGGT------VVKDEVGLQLDKVGSEVLGTASKVVITKDY

STIVGDGST-QEAVSKRVNQIKNQLEATEQDYEREKLNERIAKLSGGVAVIQVGAQTETELKEKKLRVEDAL

NATKAAVEEGIVVGGGIALLRLSSKVDAIKDTLENDEQKIGAEIVRKALSYPVKLIAKNAGVNGSVIVEKVL

AD-DNFNHG-YNAATGAFENLMAAGIIDPTKVVRCCLEHAGSVARTFLTSDAVVVSIPE---PEPAVPA-GN

PMDNSGTKSLSST---------

>Selmo_168153/1-550

------------------------------------------------------------------------

------------------------MAKELVFNKDGQATKRLQAGVNKLADLVGVTLGPKGRNVVLESKYGSP

KIINDGVTVTKEIELEDPVENIGVKLVRQAASKTNDLAGDGTTTSLILAQGLIAEGVKVVAAGSNPIQITRG

IDKTIAALVKELKEISKEVE-DSELADVATVSAGNNDEVGKMIAEAMNKVGRKGVVTLEEGKSAENTLHVVE

GMQFERGYMSPYFVTDTDKMAVEYDNCKLLLVDRKIATARDVIGILEDAIRGGYPLMIMAEEIEQEALATLV

VNRLRGSLKIVAIKAPGFGERKTQYLEDIAILTGGT------VVREEVGLSLDKVGREVLGNAAKLVVTKDS

TTIVGDGST-QEAVTKRVTQIRNLMEVAEQSYEKEKLNERIAKLSGGVAVVTVGAQTETELKEKKLRVEDAL

NATKAAVEEGIVVGGGTALLRLSKKVDAIKETLENQEQKIGADIVKRALGYPIKLIAKNAGVNGSVVVEKVL

AN-DNFNFG-YNAATGTYEDLMAAGIIDPTKVVRCCLEHAGSVAKTFLTSDVVVVDIKEEFIPTGA-AQ-GM

AMDNS---GYPPY---------

>AT3G13470/1-596

---MASTFTATSSLGSLLA--------------------------PNAIKLSSATSISSSSFGRRHNVCVR-

-----------RSRPAIVC-----AAKELHFNKDGTTIRKLQTGVNKLADLVGVTLGPKGRNVVLESKYGSP

RIVNDGVTVAREVELEDPVENIGAKLVRQAAAKTNDLAGDGTTTSVVLAQGFIAEGVKVVAAGANPVLITRG

IEKTAKALVNELKLMSKEVE-DSELADVAAVSAGNNHEVGSMIAEAMSKVGRKGVVTLEEGKSAENNLYVVE

GMQFDRGYISPYFVTDSEKMSVEYDNCKLLLVDKKVTNARDLVGVLEDAIRGGYPILIIAEDIEQEALATLV

VNKLRGTLKIAALKAPGFGERKSQYLDDIAILTGAT------VIREEVGLSLDKAGKEVLGNASKVVLTKEM

TTIVGDGTT-QEAVNKRVVQIRNLIEQAEQDYEKEKLNERIAKLSGGVAVIQVGAQTETELKEKKLRVEDAL

NATKAAVEEGIVVGGGCTLLRLASKVDAIKDTLENDEEKVGAEIVKRALSYPLKLIAKNAGVNGSVVSEKVL

AN-DNVKFG-YNAATGKYEDLMAAGIIDPTKVVRCCLEHAASVAKTFLMSDCVVVEIPE---PEPV-PA-GN

PMDNS---GY-GY---------

>Bradi3g00480/1-598

---MASPFGGTSTCGLKA---------------------------AAPAGFATKKQLSLVLPPCVSLPQ---

-----------KFRPRRKCSFRVNAAKELYFNKDGSAIKKLQNGVNKLADLVGVTLGPKGRNVVLESKYGSP

KIVNDGVTVAKEVELEDPVENIGAKLVRQAAAKTNDLAGDGTTTSVILAQGMITEGVKIVAAGANPVQIARG

IEKTAKALVSELRKMSKEVE-DSELADVAAVSAGNNYEVGNMIAEAMSKVGRQGVVTLEEGKSAENNLYVVE

GMQFDRGYISPYFVTDSEKMTVEYENCKLLLVDKKINNARDLITILEDAIKSGYPILIIAEDIEQEALATLV

VNRLRGALKIAAIKAPGFGERKSQYLDDIATLTGGT------VIREEIGLSLDKADSEVLGTAAKVLVTKDT

TTIVGDGST-QEEVSKRVTQIKNQIEAAEQEYEKEKLNERIAKLSGGVAVIQVGAQTETELKEKKLRVEDAL

NATKAAVEEGIVVGGGCTLLRLAAKVDAIKETLENDEQKIGAEIVRKSLSYPLKLIAKNAGVNGSVVTEKVL

AN-DNFRYG-YNAATGKYEDLMAAGIIDPTKVVRCCLEHAASVAKTFITSDAVVVDIKE---AEQA-PA-AN

PMAGS---GY-GF---------

>Bradi3g28070/1-574

MYRAAVSLASKARQAG-----------------------------SSARQVGSRLAWSRNY-----------

------------------------AAKDIKFGVEARAL-MLR-GVEELADAVKVTMGPKGRTVIIEQSFGAP

KVTKDGVTVAKSIEFKDRVKNVGASLVKQVANATNDTAGDGTTCATVLTKAIFSEGCKSVAAGMNAMDLRRG

ISMAVDDVVTNLKGMARMISTPEEIAQVGTISANGEREIGELIAKAMEKVGKEGVITIADGNTLYNELEVVE

GMKIDRGYISPYFITNPKTQKCEMEDPMILIHEKKISNLPAFVKVLELAHAKQRPLLVVAEDLESEALGTLI

LNKLRVGLKLCAIKAPGFGENRKANLQDLAILTGGE------VISEELGMSLENFDPQMLGTCKKVTVSKDD

TVILDGAGD-KKNMEERAEQIRSAIEESTSDYDKEKLQERLAKLSGGIAVLKIGGASEAEVSEKKDRVTDAL

NATKAAVEEGIVPGGGVALLYASKALDKLQTA--NFDQKIGVQIIQNALKTPVYTIASNAGVEGAVVVGKLL

EQ-DNTDLG-YDAAKGEYVDMVKVGIIDPLKVIRTALVDAASVSSLMTTTESIIVEIPK---EEKEAPA---

-MGGMGGMDY------------

>Bradi5g11240/1-586

---MSRMPPPPPPLSSK----------------------------PPTLPFSP-------------------

-----------KKPPPMP------VYKDLHFNHDLSATKKLQAGVDLVARLVGVTLGPKGRNVVLSNKYGPP

KIVNDGETVLKEIELEDPLENLGVKLVRQAGARTNDIAGDGCTTSIILAQGLIAEGMKVLAAGINPVQIARG

IGKTSDALVSELRSMSREIE-DHEIAHVAAVSAGNDYAVGNMISDAFKRVGRKGMVRIENGRGTENSLEIVE

GMQFERGYLSPYFVTNHANMSVEFTDCKILLVDKIITDPRELLKVCFSAVKEDFPLVIIAEDVEEEALATLT

RNKLSGMIKVAAIEALSFGEQKTQCLEDIAIMTGGT------VVRDDMGYTLEEAGKEVLGSASKVVIKKDS

TLIVTDGST-LHAVEKRVAQIKGQIENSKERYQKKILGERIARLCGAIAIIQVGAQTVIEMKDKKLRIEDAL

NATMAAIDEGVVVGGGCSLLRLSKKIDIIKESLDNIEQKIGADIFKHALSYPTILIANNAGMSGKFVIQKVL

SN-ENTNYG-YNAANDCYEDLMAAGILDPSKVVRCCIEHAAVVARSFLTSDVVVVEAKE---SKPVRIRPPM

PPRNL----IPPIPASVSSIRV

>Cre07.g339150/1-577

---MAGTQMRSFQAKS-----------------------------AASARGAVR------------------

-----------GRRSVVVQ-----AAKELHFNRNMEALKKMQAGVDKLATVVGVTIGPKGRNVVLESKFGSP

KIVNDGVTIAREVELEDPVENIGAKLVRQAAARTNDTAGDGTTTATVLSAAFIAEGMKIVAAGTNPVQLTRG

MEKTVNALVKELKAASSQVHSDKDLSNVASVSAGGNPDVGKLISDAMAKVGRQGVVTMEESKTAEDALIFVE

GMQFDRGYYSPYFVTDPERMLAEYENCRILLVDKKISTARDIIGILEAAIRGNYPLLIMAEDVEQEALATLV

VNKLRGTLKVVAVKAPGFGERKSSYLEDIAILTGGT------VVKDELGITLEKATEEVLGLAAKVSISKEA

TTIVGDGRT-QQQVEGRVKQIRNLAAETEQEYEKEKLNERIARLSGGVAIIQVGAQTETELKEKKLRVEDAL

NATKAAVEEGIVIGGGCTLLRLSQKVDSIKETLSNEEQKMGADIIKRALSYPIKLIANNAGTNGSVVMQRVM

DNIDQPYYG-YNAATDTFEDLMEAGIIDPTKVVRCSLENAVSVAKTFLLADVVVTEIPE---KEKA-PA---

PAAGGGDYDY------------

>Cre17.g741450/1-581

---MSSTILGRVTGKT-----------------------------AAKGGVSRS------------------

-----------RRSVVVR------AAKELHFNKDMQALKRMQAGVDKLATVVGVTIGPKGRNVVLESKFGAP

KIVNDGVTIAREVELSDPVENIGATLVRQAAARTNDTAGDGTTTATVLSAAFIAEGMKIVSAGTNPVQLVRG

MEKTVQELVKELRKMSSVVQTDKDLANVACVSAGGNTDIGSLISDAMAKVGRTGVVTMEEGKTAEDQLVFVE

GMQFERGYTSPYFVTDPERMICEYENCKILLVDKKISTARDIITILESAIRGNYPLLIMAEEVEQEALATLV

VNKLRGTLKVVAIKAPGFGERRSSYLEDIAILTGGT------VVRDEMGVSLEQATDAVLGTAAKITITKER

TTVVGDGST-AADVAARVKQIRNLQMQTDQDYEREKLQERIARLSGGVAIIQVGAQTETELKEKKLRVEDAL

NATRAAVEEGVVPGGGCTLLRLSEKVDVIKRRMTDPEQQMGADIIKRALCYPIKLIAQNAGVNGSVVMNEVM

KNLDRPHYG-YNAATDSFENLMETGIIDPSKVVRCSMENAVSVAKTFLLADVVVTELKE--IEAGAKPNPVA

PGAAGFGGGL------------

>Mapoly0001s0499/1-613

---MASAVGSMAAVNAAASLKPVEISA------------------SKSVKNAQSLASSSAF-SRLSFNDVHR

RQSRSAV----ARKGAVRTNA---MAKELYFNKDGSATKKMQAGVNKLADLVGVTLGPKGRNVVLESKYGSP

KIVNDGVTVAKEVELEDPVENIGAKLVRQAAAKTNDLAGDGTTTSVVLAQGLIAEGVKVVAAGANPIQIIRG

IDKTIVALVHELKLLSKEVE-DSELADVAAVSAGNNEEVGAMIADAMSKVGRKGVVTLEEGKSAENSLYVVE

GMQFERGYISPYFVTDPEKMTVEYDNCKLLLVDKKITTARDMIGILEDAIRGGYPLLIIAEDIEQEALATLV

VNKLRGSLKVAALKAPGFGERKSQYLDDIACLTAGT------VVRDEIGLSLDKVGKEVLGNAARVELTKDY

TTIVGDGTT-QDAVSKRVVQIRNLIDNAEQEYEKEKLNERIAKLSGGVAIIQVGAQTETELKEKKLRVEDAL

NATKAAVEEGIVVGGGSTLLRLAAKVDAIKATLENDEQKVGADIVKRALSYPLKLIAKNAGVNGSVVVEKVL

SN-DNPNWG-YNAATGVYEDLMAAGIIDPSKVVRCCLENASSVAKTFLTSDVVVVEIKE---PEPAQPA--N

PMDNS---GY-GY---------

>Pp3c16_4660/1-604

---MAAASMAIAGSAATASLRV-----------------------SSESRAPSSLSSSSAI-GRVGFSGGLN

-----------QRRQRVAHRTTVKAAKELHFNKDGSAIKKMQAGVDKLADLVGVTLGPKGRNVVLESKYGSP

KIVNDGVTVAKEVELEDPVENIGAKLVRQASAKTNDLAGDGTTTSVVLAQGLIAEGVKVVAAGANPVQITRG

IDRTVIALVKELKNMSKEVE-DSELADVAAVSAGNNPEIGQMIAEAMGKVGRKGVVTMEEGRSAENHLYVVE

GMQFDRGYISPYFVTDPEKMSVEYDNCRLLLVDKKITTARDMIGILEETIRGGFPLLIIAEDIEQEALSTLV

VNKLRGSLKVAALKAPGFGERKSQYLDDIAILTSGT------VIRDEIGLALDKVGTEVLGTAAKVVLTKDS

TTIVGDGST-QDAVEKRVAQIRNLIEIAEQEYEKEKLNERIAKLSGGVAIIQVGAQTETELKEKKLRVEDAL

NATKAAVEEGIVVGGGCTLLRLASKVDAIKESLDNDEQKVGADIVKRALCYPLKLIAKNAGVNGSVVVEKVL

AN-ENSSFG-YNAATGVYEDLMAAGIIDPAKVVRCCLEHAASVAKIFLTSDVVVTEIKE---PEPV-QA-GN

PMDAS---GY-GY---------

>Pp3c27_5110/1-604

---MAATTMAMAGSAATSSLRV-----------------------AAENKAASSLSSSAAF-GRVNFGGVAK

-----------LQRQRVAHRLPVKAAKELHFNKDGSAIKRMQAGVDKLADLVGVTLGPKGRNVVLESKYGSP

KIVNDGVTVAKEVELEDPVENIGAKLVRQASAKTNDLAGDGTTTSVVLAQGLIAEGVKVVAAGANPVQITRG

IDKTVVALVKELKKMSKEVE-DSELADVAAVSAGNNPEIGQMIAEAMSKVGRKGVVTLEEGKSAENNLYVVE

GMQFDRGYISPYFVTDPEKMDVEYDNCRLLLVDKKITTARDMIGILEETIRGGFPLLIIAEDIEQEALSTLV

VNKLRGSLKVAALKAPGFGERKSQYLDDIAILTNGT------VVRDELGLSLDKVGTEVLGTAAKVVLTKDS

TTIVGDGST-QDAVEKRVAQIRNLIEIAEQEYEKEKLNERIAKLSGGVAIIQVGAQTETELKEKKLRVEDAL

NATKAAVEEGIVVGGGCTLLRLASQVDAIKDTLDNDEQKVGSEIVRRALSYPLKLIAKNAGVNGSVVVEKVL

AN-ENPAFG-YNAATGEYQDLMAAGIIDPAKVVRCCLEHAASVAKIFLTSDVVVTEIKE---PEPV-PA-GN

PMDAS---GY-GY---------

>Pp3c6_20400/1-601

---MAATNMLMAGSAATASL-------------------------RIESPAASSLSSSAAV-GRVGFVLPA-

-----------RQQRRVAQRMTVKAAKQLHFNKDGSAIKKMQAGVDKLADLVGVTLGPKGRNVVLESKYGSP

KIVNDGVTVAKEVELEDPVENIGAKLVRQAAAKTNDLAGDGTTTSVVLAQGLIAEGVKVVAAGANPVQITRG

IEKTVAALVKELKKLSKEVE-DSELADVAAVSAGNNPEVGQMIAEAMSKVGRRGVVTLEEGKSSENSLYVVE

GMQFDRGYISPYFVTDPEKMSVEYDNCRLLLVDKKIVTARDMIGILEESIRGGFPLLIIAEDIEQEALSTLV

VNKLRGSLKVAALKAPGFGERKSQYLDDIAILTNAT------VIRDETGLALDKVGTEVLGTAAKVVLTKDA

TTIVGDGST-QDAVSRRVNQIRNLIEVAEQEYEKEKLNERIAKLSGGVAIIQVGAQTETELKEKKLRVEDAL

NATKAAVEEGIVVGGGCTLLRLASKVSAIVSTLDNDEQRVGAEIVRRALCYPLKLIAKNAGVNGSVVVEKVL

AN-SNPSFG-YNAATGVYEDLMAAGIIDPTKVVRCCLEHAASVAKIFLTSDVVVTEIKE---AEVA-VA-GN

PMDAS---GY-GY---------

>Azfi_s0217.g058536/1-592

---MASLSFSSSSFTS-----------------------------FPQRQLLLSNNSPSSFPSSSQL-----

-----------QRSSALHTRS---MAKELHFNNDGSTMKKLQAGVNKLSDLVGVTLGPKGRNVVLESKYGAP

KIVNDGVTVAKEVELEDPVENIGAKLVRQAAAKTNDLAGDGTTTSVVLAQGLIAEGVKVVAAGANPIQITRG

IEKTVKALVEELKKISKDVE-DSELADVAAVSAGNNYEVGNMIAEAMSKVGRKGVVTLEEGKSAENNLVVVE

GMQFDRGYISPYFVTDTEKMIIEYANCKLLLVDKKITTARDIIGILEDAIKGGYPLLIIAEDIEQEALATLV

VNKLRGSLKIAALKAPGFGERKSQYLDDIAILTGGT------VIRDEIGLSLDKVGREVLGTAAKVVLTKDT

TTIVGDGST-QDEVTKRVAQIKTLIEIAEQDYEREKLNERIAKLSGGVAILQVGAQTETELKEKKLRVEDAL

NATKAAVEEGIVVGGGSALLRLASKVDAIKETLDNYEQQVGADIVKRALSYPLKLIAKNAGVNGSVVVERVL

SN-DNLKYG-YNAATGAYEDLMAAGIIDPTKVVRCCLEHAASVAKTFLTSDVVVVEIKE---PEPSVPA-GN

PMDNS---GY-GY---------

>Azfi_s0001.g000356/1-608

---MAAMIAAKLSSLSATAPLSERRCL-----------------APSQKALSSSASISSAF-PQPKLSKSTK

-----------KHNCAVSTRA---MAKELHFNKDGATMKKLQAGVDKLADLVGVTLGPKGRNVVLESKYGAP

KIVNDGVTVAKEVELEDSVENIGAKLVRQAAAKTNDLAGDGTTTSVVLAQGLIAEGVKVIAAGANPIQITRG

IEKTVKALVEELKKMSKDVE-DSELADVAAVSAGNNYEVGNMIAEAMSKVGRKGVVTLEEGRSAENSLIVVE

GMQFDRGYISPYFVTDTEKMIIEYANCKLFLVDKKITTARDIIGILEDAIRGGYPLLIIAEDIEQEALATLV

VNKLRGNLKIAALKAPGFGERKSQYLDDIAILTGGT------VVRDEVGLALDKVGREVLGTAAKVVLTKDA

TTIVGDGST-QEEVTKRVAQIKSLIEVAEQEYEKEKLNERIAKLSGGVAILQVGAQTETELKEKKLRVEDAL

NATKAAVEEGIVVGGGSALLRLASKVDAIKETLDNYEQQVGADIVKRALSYPLKLIAKNAGVNGSVVVEKVL

SN-DNPKYG-YNAATGKYEDLVAAGIIDPTKVVRCCLEHAASVAKTFLTCDVVVVEIKE---PEGAAPA-GN

PMDNS---GY-GY---------

>Azfi_s0029.g024060/1-637

---MASSSSAAALCTPSSSSSPSFSSALHLLRASSGRRTRRAASLPPTTSISISLSFSSSFSSSLSLREGHG

NSSSSCCRVQRRRAARLQTWA---MAKELCFNTDGSTIKRLQAGVDKLADLVGVTLGPKGRNVVLESKFGAP

KIVNDGVTVAKEVELEDPVENIGARLVRQAAAKTNDLAGDGTTTSVVLAQGLIAEGVKVVAAGANPIEIARG

IEKTVKALVEELKKLSKEVE-DSELVDVAAVSAGNNYEIGTMIAEAMQKVGRKGVVTLEEGKSAENRLHVVE

GMQFDRGYISPYFVTDNEKMIVEYTTCKLLLVDKKITNARDIVGALEDAIRGGYPLLIIAEDIEQEALATLV

VNKLRGALKIAALKAPGFGERKSQYLDDIAILTGGT------VVRDAVGLSLDQVGGEVLGTAARVVLTKDT

TTIVGDGST-QDVVSKRVAQIKTLLEDAEQDYEKEKLSERIAKLSGGVAIIQVGAQTETELKEKKLRVEDAL

NATKAAVEEGIVVGGGCALLRLAVKVDAIKETLEDREQQIGAEIVKRALRYPLKLIAKNAGVNGSVVVEKVL

AN-ENFKFG-YNAATGAYEDLMAAGIIDPTKVVRCCLEHAGSVAKTFLTSDAVVYEIKE---PESPAVP-SN

PMDNS---GY-GY---------

>Sacu_v1.1_s0182.g024885/1-614
[truncated: 731,694 more chars]
