# Supplementary material for: Plasticity in plastid redox networks: evolution of glutathione-dependent redox cascades and glutathionylation sites
Source: BMC Plant Biol. 2021 Jul 5;21:322. doi: 10.1186/s12870-021-03087-2 (PMC8256493; doi:10.1186/s12870-021-03087-2)
Supplement: Supplementary file 11 — Additional file 11. Word-file containing AMY3 alignment with additional angiosperm sequences. [file 12870_2021_3087_MOESM11_ESM.docx]

**Additional file 11: Alignment of AtAMY3 with increased phylogenetic coverage**

AMY angiosperm alignment using Jalview and Muscle to generate the alignment and Mview (<https://www.ebi.ac.uk>) to display it.

Species abbreviation in gene identifiers:

*Aco = Ananas comosus; Atricho = Amborella trichopoda; Macum = Musa acuminata; Spoyrhiza = Spirodela polyrhiza; Brara = Brassica rapa; Lus = Linum usitatissimum; Mesculenta = Manihot esculenta; Potri = Populus trichocarpa; Sapur = Salix purpurea; Zmays = Zea mays; Os = Oryzia sativa; Zosma = Zostera marina Cb = Chara braunii; Aa = Anthoceros agrestis; Mp = Marchantia polymorpha; Pp = Physcomitrium patens; Sm = Selaginella moellendorffii; Sc = Salvinia cucullata; Af = Azolla filiculoides; Bd = Brachypodium distachyon; At = Arabidopsis thaliana*

AMY3


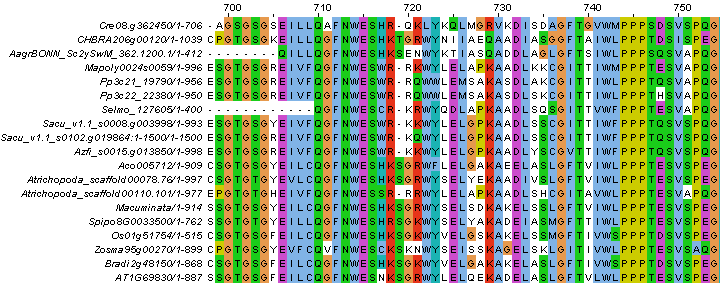


Figure 2: Alignment position of AT1G69830 Cys119 at position 709

Mview alignment file:

cov pid  **1** **[ . . . . : . . .** **80**

1 Cre08.g362450/1-706 100.0% 100.0% **-----------------------MDRSLLSRTSPRMQLGR----------------------------------------**

2 CHBRA206g00120/1-1039 94.3% 23.1% **MGNGSKRYGSNNLHFAAVCEKGSGGLVTLPHLGGRLRGVGGARSGGHSDDGGDHSTSPRAAASAASAADGLLIVLSALAD**

3 AagrBONN_Sc2ySwM_362.1200.1/1-412 57.8% 29.9% **--------------------------------------------------------------------------------**

4 Mapoly0024s0059/1-996 98.9% 25.0% **------MEIFATMQATFTTGPPLNSTEVRKCRAPDAALHQSLLHLR---KSPKVCSVRHDDIFNSSGLSTPVKPFFGGK-**

5 Pp3c21_19790/1-956 98.9% 25.6% **----------------------MDTILTRSLAGVSGHLLPIGRGEFQSTSPSIHLISSYHCAGSNNNNSTIKTPFLGER-**

6 Pp3c22_22380/1-950 98.9% 26.0% **----------------------MDTVSARSLAGMSSNLLSSPARTWHGEFRLVSQQIPLISSYHVTGSNTNNSTSVRTS-**

7 Selmo_127605/1-400 56.7% 45.3% **--------------------------------------------------------------------------------**

8 Sacu_v1.1_s0008.g003998/1-993 98.7% 26.1% **-----------MVSVVPMTFDAISGLPMFSKNKLDTIQPRNISSAGIHGSGSNSFSGPSLRVETLSG-SGRYTQKLRLF-**

9 Sacu_v1.1_s0102.g019864:1-1500/1-1500 98.7% 16.9% **-----------MVSVALTQHDIISGLCSLSSNKVDSILQSRYACISTVAANLKPLRAPVVYIEGLAV-SGRNPFQSGIS-**

10 Azfi_s0015.g013850/1-998 98.6% 25.3% **--MKPWDIDLLLMEKVSILPKQHDTFSCLCTISKSTIIQPRIASTGIHLSSSKSFSGPLLCVETLAAVSGRHLPPSGVS-**

11 Aco005712/1-909 95.9% 28.3% **-------MSIVRLKPILHHLPPIENPRLSPRELRRSELPGSIRC------CSKPRVSVSRGLRRSDPLRSLPIIRAGVA-**

12 Atrichopoda_scaffold00078.76/1-997 96.3% 24.0% **-------MATLRLKPSLHHHTKWNPRSNQKLRNYSNWNPRLNHKLRNSTFSGLNCIYKRFDIRSFSKIKPGVVVRASST-**

13 Atrichopoda_scaffold00110.101/1-977 99.0% 25.7% **------------MALLAWPGVPSRSFSHHSILPRNTKLHNPNLCIWHHSFAFRNASNHRRKIHEREKGLDGYQPLLRAS-**

14 Macuminata/1-914 95.9% 27.2% **-------MLLVRWKPVLHCPPQGHRRRGFAWPRGPRRSLLLRRPIRS--AAPAFLSISSSKFSQAGRARVRPVVRAGLA-**

15 Spipo8G0033500/1-762 93.2% 31.1% **--------------------------------------------------------------------------------**

16 Os01g51754/1-515 65.2% 36.0% **--------------------------------------------------------------------------------**

17 Zosma95g00270/1-899 95.6% 25.4% **-----------MLSLIRFGHPVVRPRIHEHTLFPSFSQWRLSGRLRLSVPTTRRRSGAVSCFFRPWRNSELANMEEGGK-**

18 Bradi2g48150/1-868 94.9% 27.5% **------------------MSAASWSIPAIPRAAPPARGGLPGDAFLVAARPGPGRRRAAPGRRLRLRGGGVVVARAGAA-**

19 AT1G69830/1-887 94.3% 25.6% **-------MSTVPIESLLHHS---YLRHNSKVNRGNRSFIPISLNLRSHFTSNKLLHSIGKSVGVSSMNKSPVAIRATSS-**

cov pid  **81**  **. 1 . . . . : .** **160**

1 Cre08.g362450/1-706 100.0% 100.0% **--------------------------------------------------------------------------------**

2 CHBRA206g00120/1-1039 94.3% 23.1% **TQPAGQSQIEWPGDDQVQTTLQKDYALTRRVKVRGKLWVKVAL---WENLHRIRISVECDVL-HRALLHWGVTTREEA--**

3 AagrBONN_Sc2ySwM_362.1200.1/1-412 57.8% 29.9% **--------------------------------------------------------------------------------**

4 Mapoly0024s0059/1-996 98.9% 25.0% **-----RKLYKWQRPSSELQKRENLPRLLVTASTAG-----------IGSDNSSESSFGSNFP-DPVSLRAALKASQARVL**

5 Pp3c21_19790/1-956 98.9% 25.6% **----------------VLVSRNVNTTKALRRIWHGRVFVS------VLKGENNNGSRGFQIPDDVESLKVALAAAEARAD**

6 Pp3c22_22380/1-950 98.9% 26.0% **-------------------FFGERSPNTSKVAKGQRRSRPDHAVVSVLKGDRITGSRDVQAPDDVESLKAALAAAEARTD**

7 Selmo_127605/1-400 56.7% 45.3% **--------------------------------------------------------------------------------**

8 Sacu_v1.1_s0008.g003998/1-993 98.7% 26.1% **-----RGI------KCSGNVVSRSGSTPVEENDQGSSKAEQDAKVLVKDFDANSIGQNLD---DIEGLKSALKAAHAKML**

9 Sacu_v1.1_s0102.g019864:1-1500/1-1500 98.7% 16.9% **-----RGI------RCSNHVPS-SGLTPVEEGGKDGSQLEEAIQGMVKDSRDGEADLNIDMN-NVDSIKAALMAAHARIA**

10 Azfi_s0015.g013850/1-998 98.6% 25.3% **-----RWI------RCSGNRFSRSGSSP----IEGGQDASQLEETIKRLVDEGSIGQNIDLN-DVEGLKTILVAAHARIV**

11 Aco005712/1-909 95.9% 28.3% **-----PTPSLAEDDQATEVVYSETFLLKRSQAVEGKVSVRLDAA--EEDGSRWRLVIGCNLP-GKWILHWGVTYHDER--**

12 Atrichopoda_scaffold00078.76/1-997 96.3% 24.0% **-------NTSVEEAVASDVLFTETFQLKRSEKVEGKISVRVDH---QKDDDKSQVAIGCNLP-GKWVLHWGVTYYDDV--**

13 Atrichopoda_scaffold00110.101/1-977 99.0% 25.7% **--------------------MGDSKDILTDTVFEGDGVSSGSGNGEVLQITREEFIATND----------ALEEARLRQE**

14 Macuminata/1-914 95.9% 27.2% **-----QTP-SLADVENTEILFSETLSLKRSQTVEGKITVRLDPAVAEEEVSKWRLTIGCNLE-GKWTLHWGVSYCDDLGR**

15 Spipo8G0033500/1-762 93.2% 31.1% **--------------------------------------------------------------------------------**

16 Os01g51754/1-515 65.2% 36.0% **--------------------------------------------------------------------------------**

17 Zosma95g00270/1-899 95.6% 25.4% **-----TLEEGQNLIRSAVVVMEEKFEVQRTQMVEGKLTVRLERS--EDGVNKGRFVVGCDIE-GNWVLHWGVTYFDQ-LE**

18 Bradi2g48150/1-868 94.9% 27.5% **-------EVPVTHPEESGVVFSEKFPLRRCKTVQGKAWARVVAE--PDGEGMCKIVIGCDVE-GKWVLHWGVSYDGEQ--**

19 AT1G69830/1-887 94.3% 25.6% **-----DTA-VVETAQSDDVIFKEIFPVQRIEKAEGKIYVRLK----EVKEKNWELSVGCSIP-GKWILHWGVSYV---GD**

cov pid  **161**  **. . . 2 . . . .** **240**

1 Cre08.g362450/1-706 100.0% 100.0% **---------PQQLPPPTVPLAQVPRLQRR--CV-----------------------------------VGSRVCQPVVAV**

2 CHBRA206g00120/1-1039 94.3% 23.1% **-GRQWTQPPKKIRPPGTVKYKDYAARTPLRSCYGAVSDGGEEHGGGRVGVDLEIAEEG----------APEVIAFVLKDE**

3 AagrBONN_Sc2ySwM_362.1200.1/1-412 57.8% 29.9% **------MPPPSTPAP-----------------------------------------------------------------**

4 Mapoly0024s0059/1-996 98.9% 25.0% **EIENEKRDILEALRQSEAKVQEYAALMVQ--TTDEALSELEASKKLFKAELSKVLEEKSTLQKETLLAKQDAVNLAVKIE**

5 Pp3c21_19790/1-956 98.9% 25.6% **AAKKAEKQALEALTAMEGKSSDTVKTSRN--MKQIKLKGGND--------------------------DADGISLAVQVE**

6 Pp3c22_22380/1-950 98.9% 26.0% **AAKQAEKKALDALAAMKNKSRDMAQNDQN--SQE----------------------------------VFEGISLAVQVE**

7 Selmo_127605/1-400 56.7% 45.3% **--------------------------------------------------------------------------------**

8 Sacu_v1.1_s0008.g003998/1-993 98.7% 26.1% **AAESEKADALRALAQAEARLREYATSAAE--TTESAVHEMEAAKESVSIELQNIMKEKLATESELVVARSDAIELAVSVE**

9 Sacu_v1.1_s0102.g019864:1-1500/1-1500 98.7% 16.9% **AAENEKADALRSLEVAETRLEEYASTAVQ--VTESAVHEMQAAKESVNMELKNIMEQKLALESELVVVKKDALELALSVD**

10 Azfi_s0015.g013850/1-998 98.6% 25.3% **AAESEKEDALKALAEAEAKLQEYTSTAVQ--ATEEPVKKTKHSNKSEGVDLQSIIDQKLAVESELAVAKKNAIELAVCVD**

11 Aco005712/1-909 95.9% 28.3% **-GSEWDQPPPEMWPPESVPIKDYAIETPL--KTSSSNPEEQVLHEVQIDFDSSV--------------QIGAIHFVLKEE**

12 Atrichopoda_scaffold00078.76/1-997 96.3% 24.0% **-SSEWDQPPPDMRPPDSIAIKDYAIETPL--KKSPLAVEGNSLYEVQIDIKVNH--------------SVGALHFVLKDE**

13 Atrichopoda_scaffold00110.101/1-977 99.0% 25.7% **AAEKERDRLTQDLALSEAKLQEYAATIDG--NRELAVAELEAAKSLFHDKLQDSLNEKFALETRLVLAKQDAVELAVQVE**

14 Macuminata/1-914 95.9% 27.2% **QAFEWDQPPPEMRPPESVLIKDYAIETPL--KRLSSQSERQALHELQIEFDSNT--------------PIAAIHFVLKEE**

15 Spipo8G0033500/1-762 93.2% 31.1% **RGGEWDQPPLDMRPPGSIPIKDYAIETPL--KKSSSASEGEILHELQIDLKPSF--------------SISAIHFVLKDE**

16 Os01g51754/1-515 65.2% 36.0% **--------------------------------------------------------------------------------**

17 Zosma95g00270/1-899 95.6% 25.4% **FGSEWEQPPNEIRPPGSIPIKDYAVETPL--KKSSSNIMGETFHEVKIDFNFDS--------------SIAAIHFVLKEE**

18 Bradi2g48150/1-868 94.9% 27.5% **-GREWDQPPSEVRPPGSVTIKDYAIETPL---VGSPNSEGHMVHEVEIKFNQDT--------------PIAIINFVLKEE**

19 AT1G69830/1-887 94.3% 25.6% **TGSEWDQPPEDMRPPGSIAIKDYAIETPL--KK---LSEGDSFFEVAINLNLES--------------SVAALNFVLKDE**

cov pid  **241**  **: . . . . 3 . .** **320**

1 Cre08.g362450/1-706 100.0% 100.0% **RPGRASAGRGGRLVVSSVDMSNSPLSSMDAGEGLDIMFDNNSDAECTVVTVEGKDKAHLLMSLTGG-----------FSS**

2 CHBRA206g00120/1-1039 94.3% 23.1% **ETGRWHDKAGGNFIINLADLLEQRHERKTPAAAAHAAGGLRLLDGGAV------------------------------GL**

3 AagrBONN_Sc2ySwM_362.1200.1/1-412 57.8% 29.9% **--------------------------------------------------------------------------------**

4 Mapoly0024s0059/1-996 98.9% 25.0% **KIAESAIQEATQRFAEDLVLKDSAAETAAAEAAAGVEESIRLAASDAAALVVTEASTVMEEALAAASLAKQQATKAQEAL**

5 Pp3c21_19790/1-956 98.9% 25.6% **KISEAAIQKATARITEDATLKVAAAETAAAEAVLQLEERLQRAVDEAASAVAGETQVAIDEARAAAKVAKAQAAKSEALL**

6 Pp3c22_22380/1-950 98.9% 26.0% **KISESTIQKATLRITEDAELKIAAAETAAAEVILELEDQFRRAAEDAVQAASVEAQVTIDEARAAVSAARVQAEKSEAIL**

7 Selmo_127605/1-400 56.7% 45.3% **--------------------------------------------------------------------------------**

8 Sacu_v1.1_s0008.g003998/1-993 98.7% 26.1% **KVADTILREATAHLIEEAQLKIAAAKTSAAEAAANVEERIKSAVHDTANAMIRETKDAIEKSFAALEAAKEKAQKSEIAL**

9 Sacu_v1.1_s0102.g019864:1-1500/1-1500 98.7% 16.9% **KVADSILGEATTHLAEEARLKVAAAKTSAAEAAANVEERIRSAILDTTDNLIKETRDAIEKSFAALEAAKEKAQKSEVAL**

10 Azfi_s0015.g013850/1-998 98.6% 25.3% **KVADAIYEETTASLAEEAHLKIAEAKTSAAEAANSVEERVKSAVLDTANAMIRETRDAIEKSFSALEAAKEKAQKSEIAL**

11 Aco005712/1-909 95.9% 28.3% **ETGAWFQHKGRDFRITLRDTF-KEESSLGGTQGFSIWPGALEQISTLLK--PEGSSPMTQETLRGGREAKQWNSR-IAGL**

12 Atrichopoda_scaffold00078.76/1-997 96.3% 24.0% **ETGAWYQHRGRDFRVCLLEDLQDENDKVGDKKSFSLWPGDFVKMPEVLLTAIKREANGQEPNGDGKDARKK--AKLIEEF**

13 Atrichopoda_scaffold00110.101/1-977 99.0% 25.7% **KLAEIAFQQSTSHILEDAQMRVSAAGTSAAEAAYHIEEQLRTTTENTLSSIVEQSNDTLGKVLMAAQQASDHAKRAMESL**

14 Macuminata/1-914 95.9% 27.2% **ETGAWFQHKGRDFRISFTDYF-EVANSVGGNQGLSIWPGGFDQISSLLLKAEESTSKKEDPDDEDGNVVKQ--NRCIAPI**

15 Spipo8G0033500/1-762 93.2% 31.1% **ETGAWYQYRGRDFKIPL--LIWEDEGIISGEKGFSLWPGALSQISNILLKTDGAATNVDDREKSEAKPE----IRLVEKY**

16 Os01g51754/1-515 65.2% 36.0% **--------------------------------------------------------------------------------**

17 Zosma95g00270/1-899 95.6% 25.4% **VSGAQYQHKGRDFKIPLIDNVQEDD--IGIDGSKDPFNSSGLKVDGIRINAIENSSSCKDVNYKNG---------LLEGY**

18 Bradi2g48150/1-868 94.9% 27.5% **ETGAWFQHKGGDFRIPLSGSLEDGDPFGAQQDTVHPGAKPEGSSAQPQETVPGDKGPSVKR---------------ISEF**

19 AT1G69830/1-887 94.3% 25.6% **ETGAWYQHKGRDFKVPLVDDVPDNGNLIGAKKGFGALGQL--------------SNIPLKQDKSSAETDSIEERKGLQEF**

cov pid  **321**  **. . : . . . . 4** **400**

1 Cre08.g362450/1-706 100.0% 100.0% **AGLTVISASITSDDGRVLDVFRVQTADGKKV--PEEQFPSVREHILSVT--ATSSRSSMPAIYGIVAAAEVERLKPLRSQ**

2 CHBRA206g00120/1-1039 94.3% 23.1% **LEETHDLENEVDIGNRLSAMVGLAEGGDVKVLLSTDLSGPVLLHWGLVKRGEEQSKWTVPAKRFLPSNSTVYKKRAVQTV**

3 AagrBONN_Sc2ySwM_362.1200.1/1-412 57.8% 29.9% **-----------------------------------------------------------PPKPK-PAEPET---------**

4 Mapoly0024s0059/1-996 98.9% 25.0% **AKGMEIFEELSAAKLTTLSLQEKVSYLERELGISQGIVESLRLELKASQ--MRTEAANARAAEA-EAAVQEVQRAAAEDG**

5 Pp3c21_19790/1-956 98.9% 25.6% **NEQVNVLNELAEIEAKMLVLEEALLAAGRQLQIANGETERVRIELDAVQ--SFIKTATARAEAA-EKTIIEVQKAASKAA**

6 Pp3c22_22380/1-950 98.9% 26.0% **NKQVKALNELAEAEAKVLMLEEALLDAGRKLQLANGETERIRIELDSAQ--RFIKTATARAEAA-ERTAEELQRAAAKEA**

7 Selmo_127605/1-400 56.7% 45.3% **--------------------------------------------------------------------------------**

8 Sacu_v1.1_s0008.g003998/1-993 98.7% 26.1% **FQRMQILDDMVLKEASALGLQQAESDLQRKLLAAESEIQRLHGEVKAVL--ARAEAAEVRASTA-DEALKQFQEAANISA**

9 Sacu_v1.1_s0102.g019864:1-1500/1-1500 98.7% 16.9% **FQRMQILDDMVLKEASALGLQQTASELQRKLLASESEIQRLQGEVNAVL--ARAEAAESRAAAA-YDALRQYQEAAKRSA**

10 Azfi_s0015.g013850/1-998 98.6% 25.3% **FQRMQILDDMVLKEASALGLQKTASEIQRKLLAAESEIKRLQGEVTAVL--ARAEAAESRASAA-DDALRQFQERANQDA**

11 Aco005712/1-909 95.9% 28.3% **YEEFSISKEEPVQNLMTVTVRKSNDTEKKLVQFDTDIPGEVTVHWGVCK--DDSKKWEIPPTPH-PPATKLFRSKALQTS**

12 Atrichopoda_scaffold00078.76/1-997 96.3% 24.0% **YDEYIFMKEKMVGNYLTVSVQENEEKNKALVLFDTDLPGNVIIHWGVCR--DNGKKWEIPQASH-PPSTNLFRKKALQTS**

13 Atrichopoda_scaffold00110.101/1-977 99.0% 25.7% **TDGLQVVDEMVSVHSMNVGLQSAMSELERQLTFKQNEVDRLSSELELVQ--ARANSLEARANSL-ENTLAEVQESTKRKL**

14 Macuminata/1-914 95.9% 27.2% **YKEFPILKEEFVPNHMTVSVRSSDKTDKNIVQFDTDLPGDVVIHWGVCK--DDGRKWVIPSTPH-PPATKIFRHKALQTL**

15 Spipo8G0033500/1-762 93.2% 31.1% **SEEFPIVQEETVQNYLTVSVQRSDELDRNVVHFDTDIPGNVVVHWGVCR--DENRNWEIPTPPH-PAGSRVFRGKALQTL**

16 Os01g51754/1-515 65.2% 36.0% **------------------------------------------------------MTWEIPPEPH-PPATKIFRQKALQTM**

17 Zosma95g00270/1-899 95.6% 25.4% **HKEYSICKEEQVTNSVTVAIMRNEGENTNHLLFDTDIPGEVIVHWGVCK--GDDKSWHIPKTPH-PPKSRVFRKKAVQTL**

18 Bradi2g48150/1-868 94.9% 27.5% **YGEYPILKSEYVQNFVSVTVTENSETDKSLVEFDTDITGQVIIHWGVCK--DNTMTWEIPSEPH-PPKTKIFRQKALQTL**

19 AT1G69830/1-887 94.3% 25.6% **YEEMPISKRVADDNSVSVTARKCPETSKNIVSIETDLPGDVTVHWGVCK--NGTKKWEIPSEPY-PEETSLFKNKALRTR**

cov pid  **401**  **. . . . : . . .** **480**

1 Cre08.g362450/1-706 100.0% 100.0% **STQND---------VDALELAAAEMTQAVAELVATERDIIRMRASNADARTLQTKEANRTEAAAGL--------------**

2 CHBRA206g00120/1-1039 94.3% 23.1% **MKRDEGCDGDGEGSWIMV-------------DVGGGFSELRFVLKEVDSNTWFDA--EGEDFSMPLPAAKGPKDHGGGQE**

3 AagrBONN_Sc2ySwM_362.1200.1/1-412 57.8% 29.9% **--------------------------------------------------------------------------------**

4 Mapoly0024s0059/1-996 98.9% 25.0% **RERDD----RAKQTLEEIKATLISKTEVASVVLQADLEALKAAYH-AAQEAGNVKEQANLRMYEAL--------------**

5 Pp3c21_19790/1-956 98.9% 25.6% **DEREA----SALTAIDAVKKAAKARQVADKVAFEAEADALRSAND-ASHKASEARRLVIKSRCESL--------------**

6 Pp3c22_22380/1-950 98.9% 26.0% **EERAD----SAQSAINAVKKATQVRLDADKIAFEAELDALRSAND-TSHKASEARRLVDKSRFELL--------------**

7 Selmo_127605/1-400 56.7% 45.3% **---------------------------------------------------------LDSSFCFKF--------------**

8 Sacu_v1.1_s0008.g003998/1-993 98.7% 26.1% **HEQEG----SAKKALEALKETGAARLEAARAAFKADVEVLQSALE-TVQIAGKSKEQAYTRKHQAL--------------**

9 Sacu_v1.1_s0102.g019864:1-1500/1-1500 98.7% 16.9% **QEHEE----RAAKALEALKAAGAARLEAARSAFKADIEVLQTALE-TVKIAEKSQEQAYARRSQAL--------------**

10 Azfi_s0015.g013850/1-998 98.6% 25.3% **LEHEE----RAKKALEALKLAGAARLEAARAAFKSDIEVLQAALD-TVQIAGKSQEQAYARRYQAL--------------**

11 Aco005712/1-909 95.9% 28.3% **LQPKE----NGRGSWGIF-------------PVAQESLGLLFVLK-LDKYTWLKN--DGTDFYIPL--------------**

12 Atrichopoda_scaffold00078.76/1-997 96.3% 24.0% **LQFKE----NGGGSWGLF-------------TLDKELAGLLFVLK-LDGYTWLNN--NGSDFYIPL--------------**

13 Atrichopoda_scaffold00110.101/1-977 99.0% 25.7% **LEQEE----ATKSLLKKFKEEAAKSEASATMALKVELEGIRSTVD-AAKKTMELKDRAYMQRCLAL--------------**

14 Macuminata/1-914 95.9% 27.2% **LQPKP----DGLGSWGLF-------------LVDQGTSGVVFVLK-LNEYTWLNN--NGTDFFIPI--------------**

15 Spipo8G0033500/1-762 93.2% 31.1% **LQPKA----DGSGNWGDF-------------PVDEDFSCLHFVLK-LSEYTWLND--VGDDFYIPL--------------**

16 Os01g51754/1-515 65.2% 36.0% **LQQKA----DGTGNSLSF-------------LLDGEYSGLIFVVK-LDEYTWLRNVENGFDFYIPL--------------**

17 Zosma95g00270/1-899 95.6% 25.4% **LQKKS----EGVGSWGLF-------------PMEKDISGVPFVLK-LNKDTWLDN--LGIDFYVPL--------------**

18 Bradi2g48150/1-868 94.9% 27.5% **LQQKT----DGTGNTISF-------------LLNADYSGLVFVLK-LDEYTWLRNVDNGFDFYIPL--------------**

19 AT1G69830/1-887 94.3% 25.6% **LQRKD----DGNGSFGLF-------------SLDGKLEGLCFVLK-LNENTWLNY--RGEDFYVPF--------------**

cov pid  **481**  **. 5 . . . . : .** **560**

1 Cre08.g362450/1-706 100.0% 100.0% **----------ER-------------------------------------------------KMAA-MQAVLAARRNLATE**

2 CHBRA206g00120/1-1039 94.3% 23.1% **GAAAGAPGQTTSGVAASDDRDRDRNRKKTDIARLEAKVAEPVLPIPIADMAEGGGIGVNGAPAAANVVSSSSATREDFAG**

3 AagrBONN_Sc2ySwM_362.1200.1/1-412 57.8% 29.9% **--------------------------------------------------------------------------------**

4 Mapoly0024s0059/1-996 98.9% 25.0% **----------ERSLAAAEGSAEAWKNRALSVEGLLRRVKEEGLE---AVSSVVAEEMVAGGRMET-LLGNDSRKRDLLAN**

5 Pp3c21_19790/1-956 98.9% 25.6% **----------EKSLVAAEGAAAAWRNRALTAEELLRQSRINGVE-IDSSSPVPDLLPPNIGRLEM-LPGSDAKIKDLLEN**

6 Pp3c22_22380/1-950 98.9% 26.0% **----------ERSLLAVESATAAWKNRALMAEKLLRLARINGAE--IDTSSLPVEQAPSVGRLEV-LPGSDVRIKDLLEN**

7 Selmo_127605/1-400 56.7% 45.3% **--------------------------------------------------------------------------------**

8 Sacu_v1.1_s0008.g003998/1-993 98.7% 26.1% **----------ERSLAAAETLAKAWEERALAVESLLHKSRKEGAEQYAVELNGGVIDILTGGRMET-LLGNDSRKWELLSN**

9 Sacu_v1.1_s0102.g019864:1-1500/1-1500 98.7% 16.9% **----------ERSLAAAESLAKAWEERALAVEYLLQKSGDECVD--ASERTRGFGVVLNGGRMET-LLGNDSRKWDLLSN**

10 Azfi_s0015.g013850/1-998 98.6% 25.3% **----------ERSLSSAETLAKAWEERALAVEALLQKSREEGAD--VAGFNVGLEGILTGGRMET-LLGNDSRKWDLLAN**

11 Aco005712/1-909 95.9% 28.3% **----------INVSGSSTSNSQELGNKQMDSSQGSVSTE----------------------EAKP-VVKNIEYTHEIISE**

12 Atrichopoda_scaffold00078.76/1-997 96.3% 24.0% **----------SAEIGTSSVRPTEKINAPEGHKEEDISNDVKNDTWTIEESGSSQLEKSQSGANSP--VSRVSYTDEIINE**

13 Atrichopoda_scaffold00110.101/1-977 99.0% 25.7% **----------ERSLKASEAATNVWRQRAEMAESLLQEGRLVGEE------DQDATVVVNGGRLDI-LTEDDSQRWRLLAD**

14 Macuminata/1-914 95.9% 27.2% **-------------GSVSSTTAEIGTSDPKNINSLPMKPQ----------------------GPEE-LIEAVAYTDEIIKE**

15 Spipo8G0033500/1-762 93.2% 31.1% **-----------------------ISKKELLKEAVLSESQPTDV------------------EMTQ-DVEAVSYTDDIISE**

16 Os01g51754/1-515 65.2% 36.0% **----------TRADAEADKQK---------------------------------------------ADDKSSQDDGLISD**

17 Zosma95g00270/1-899 95.6% 25.4% **----------------TGDMSLEYKTKDQIEAELISTDV----------------------KEAQ-EIESSTNTDEIITE**

18 Bradi2g48150/1-868 94.9% 27.5% **----------KEPHKSDEQK----------------------------------------------VDDKSAQTDGLIGD**

19 AT1G69830/1-887 94.3% 25.6% **----------LTSSSSPVETEAAQVSKPKR-------------------------------KTDK-EVSASGFTKEIITE**

cov pid  **561**  **. . . 6 . . . .** **640**

1 Cre08.g362450/1-706 100.0% 100.0% **----------------------PEKPKSPTEKLLE---------------------------------------------**

2 CHBRA206g00120/1-1039 94.3% 23.1% **TIAPPAPPSPPPSPPSPSSPSPSPSTTEVAEEEVNLAAITRSAKGTGAVPAGTVSSNSRVVITSSGPGPEELLQEIDRLA**

3 AagrBONN_Sc2ySwM_362.1200.1/1-412 57.8% 29.9% **--------------------------------------------------------------------------------**

4 Mapoly0024s0059/1-996 98.9% 25.0% **GPRRETPEWMRRRIEVGFQGLPPRS-SMPTNSEIE----------------------------------AQVPLHLPR-P**

5 Pp3c21_19790/1-956 98.9% 25.6% **GPRRETPDWMKRRLQTGQQNLPPMQPTSITADIDA-----------------------------------AIPLELPT-P**

6 Pp3c22_22380/1-950 98.9% 26.0% **GPRRETPDWMKRRLQIGQQVLPPMQPIAINADVDA-----------------------------------LIPLQLPS-S**

7 Selmo_127605/1-400 56.7% 45.3% **--------------------------------------------------------------------------------**

8 Sacu_v1.1_s0008.g003998/1-993 98.7% 26.1% **GPRTDTPEWMERRIEVALQGLPARTGSTPVEQ--E----------------------------------STLSLQLPS-P**

9 Sacu_v1.1_s0102.g019864:1-1500/1-1500 98.7% 16.9% **GPRRETPEWMERSIETALQGLPPRS-LTQVQD--E----------------------------------AGISLWLPS-P**

10 Azfi_s0015.g013850/1-998 98.6% 25.3% **GPRRETPEWMERRIEVALQGLPPRKLGQIEEE-------------------------------------IGVSLKLPS-P**

11 Aco005712/1-909 95.9% 28.3% **-------------IRNLVTDISSKNGKGANTKEAQ----------------------------------ESILEEIEKLA**

12 Atrichopoda_scaffold00078.76/1-997 96.3% 24.0% **-------------IRSLVSDISSERSANMKSKDAR----------------------------------ESILQEIEKLA**

13 Atrichopoda_scaffold00110.101/1-977 99.0% 25.7% **GPRRDIPEWMARRIRSICPKFPPRKTTIPEELTVS-----------------------------------SSSLTLPK-P**

14 Macuminata/1-914 95.9% 27.2% **-------------IRHLVTDISSEKGKRAKSKEAQ----------------------------------ENILQEIEKLA**

15 Spipo8G0033500/1-762 93.2% 31.1% **-------------IRSLVTDISAEKGNITKSKEAQ----------------------------------ENILQEIEKLA**

16 Os01g51754/1-515 65.2% 36.0% **-------------IRNLVVGLSSRRGQRAKNKVLQ----------------------------------EDILQEIERLA**

17 Zosma95g00270/1-899 95.6% 25.4% **-------------IRNLVTDISAEKSLTTTTKEAQ----------------------------------ESILEEIEKLA**

18 Bradi2g48150/1-868 94.9% 27.5% **-------------IRNLVVGLSSRRGQRAKNKVLQ----------------------------------EDILQEIERLA**

19 AT1G69830/1-887 94.3% 25.6% **-------------IRNLAIDISSHKNQKTNVKEVQ----------------------------------ENILQEIEKLA**

cov pid  **641**  **: . . . . 7 . .** **720**

1 Cre08.g362450/1-706 100.0% 100.0% **-------------------------------TLKPPTPMRAAAG--------------AGSGSGSEILLQAFNWESHR-Q**

2 CHBRA206g00120/1-1039 94.3% 23.1% **AEASENFRRATVIAPAAPLSQQKLEQADSKPTTTLPLTSPQNVLPLPLPLSVPDRQPCPGTGSGKEILLQGFNWESHKTG**

3 AagrBONN_Sc2ySwM_362.1200.1/1-412 57.8% 29.9% **-----------------------------------------------------------------QILLQGFNWESHKSE**

4 Mapoly0024s0059/1-996 98.9% 25.0% **DEVWSIFNAKVKEDDLYTKQAVEKEALDEQRRALERALQKKTVK---RHPEDGEGKLESGTGSGREIVFQGFNWESWR-R**

5 Pp3c21_19790/1-956 98.9% 25.6% **EDVWDVAKSKVKEDDKYTVRAAEKEALDLQRNALERALQTKSLRTLVRYPEESESKTESGTGSGREIVFQGFNWESWR-R**

6 Pp3c22_22380/1-950 98.9% 26.0% **ETVWDVSKSKVKENDKYAVRAAEKEALDLQRNAMERALQTKSIKTLVRYPEDAEEKSESGTGSGREIVFQGFNWESWR-R**

7 Selmo_127605/1-400 56.7% 45.3% **---------------------------------------------------------------------QGFNWESCR-K**

8 Sacu_v1.1_s0008.g003998/1-993 98.7% 26.1% **EEVWCIATAEVKE-DVLTREIAEKEAIDEQRRVLEKALKKKTVR---KTPQ----AMESGTGSGYEIVFQGFNWESWR-K**

9 Sacu_v1.1_s0102.g019864:1-1500/1-1500 98.7% 16.9% **EEVWSIATAEVKE-DMYTREAAEKEAIDEQRRVLEKTLKKKAVR---KTPQ----ILESGTGSGREIVFQGFNWESWR-K**

10 Azfi_s0015.g013850/1-998 98.6% 25.3% **DEVWSIATAEVKE-DVYTRQAAEKEAIDEQRRVLENTLKIKTVR-------KTAQVLESGTGSGREIVFQGFNWESWR-K**

11 Aco005712/1-909 95.9% 28.3% **AEAYKIFRSSALG------------FVEE--TVSHAEPSKPAVQ------------ICSGTGSGYEILCQGFNWESHKSG**

12 Atrichopoda_scaffold00078.76/1-997 96.3% 24.0% **AEAYSIFRSSIPT------------FLKE--LVSEPEIEKPQPK------------ICSGTGTGYEVLCQGFNWESHKSG**

13 Atrichopoda_scaffold00110.101/1-977 99.0% 25.7% **EEVWSIAQEKPKQGDTFIKQVIEKEAIGKQRKALERALQRKTIQ---RQRIPEPTKLEPGTGTGHEIVFQGFNWESSR-R**

14 Macuminata/1-914 95.9% 27.2% **AEAYSIFRISIPG------------FVE---LASDTELLKPAVK------------LSSGTGSGYEILCQGFNWESHKSG**

15 Spipo8G0033500/1-762 93.2% 31.1% **AEAYGIFRSSIPV------------YVEE--PVSDEELLKPPVQ------------ISSGTGTGYEILLQGFNWESHRSG**

16 Os01g51754/1-515 65.2% 36.0% **AEAYSIFRSPTID------------TVEESVYIDDSSIVKPA---------------CSGTGSGFEILCQGFNWESHKSG**

17 Zosma95g00270/1-899 95.6% 25.4% **AEAYSVFRSITPI------------YVEE--PISDATAIKPPIG------------KCPGTGSGYEVFCQVFNWESCKSK**

18 Bradi2g48150/1-868 94.9% 27.5% **AEAYSIFRSPTID------------AVEDSVYIDDPATVKPA---------------CSGTGSGFEILCQGFNWESHKSG**

19 AT1G69830/1-887 94.3% 25.6% **AEAYSIFRSTTPA------------FSEE--GVLEAEADKPDIK------------ISSGTGSGFEILCQGFNWESNKSG**

cov pid  **721**  **. . : . . . . 8** **800**

1 Cre08.g362450/1-706 100.0% 100.0% **KLYKQLMGRVKDISDAGFTGVWMPPPSDSVSPQGYLPRDLYSLDSAYGSEAELRELIAAFHQNNIKVIADIVVNHRCANS**

2 CHBRA206g00120/1-1039 94.3% 23.1% **RWYNIIAEQAADIASGGFTAIWLPPPTDSISPEGYMPRDLYDLNSKYGDMEALKRVVKRLHEVGMVVLGDAVLNHRCAHF**

3 AagrBONN_Sc2ySwM_362.1200.1/1-412 57.8% 29.9% **NWYKTIASQADDLAGLGFTSIWLPPPSQSVAPQGYLPADLYNLDSKYGTLADLKEAIAKLHSVGIQVLADIVVNHRCAQS**

4 Mapoly0024s0059/1-996 98.9% 25.0% **KWYLELAPKAADLKKCGITTIWMPPPTESVAPQGYMPGDLYNLNSAYGTVDELKQCIEEMHNNDILVLGDAVLNHRCAQK**

5 Pp3c21_19790/1-956 98.9% 25.6% **QWWLEMSAKASDLAKCGITTIWLPPPTQSVAPQGYMPGDLYNLNSAYGGSEELKLCINEMHKHKILVLGDVVLNHRCAQK**

6 Pp3c22_22380/1-950 98.9% 26.0% **QWWLEMSAKASDLSKCGITTIWLPPPTHSVAPQGYMPGDLYNLNSAYGGSEELKQCIDEMHKHNILVLGDVVLNHRCAQK**

7 Selmo_127605/1-400 56.7% 45.3% **RWYQDLAPKAADLSQSGITTVWFPPPTESVAPQGYMPVDLYNLNSAYGSMDELKHCIQEMHKHDLLVLGDVVLNHRCAYK**

8 Sacu_v1.1_s0008.g003998/1-993 98.7% 26.1% **KWYLELGPKAADLSSCGITTIWFPPPTQSVSPQGYMPGDLYDLNSSYGTEEELKNCIEEMHNNELLVLGDAVLNHRCAQF**

9 Sacu_v1.1_s0102.g019864:1-1500/1-1500 98.7% 16.9% **QWYLELGPKAADLSSCGITTIWFPPPTQSVSPQGYMPGDLYNLNSSYGSVEELKNSIEEMHSNELLVLGDVVLNHRCAQF**

10 Azfi_s0015.g013850/1-998 98.6% 25.3% **KWYLELGPKAADLYSCGVTTIWFPPPTQSVSPQGYMPGDLYNLNSAYGTEEELKNCIEEMHNHELLVLGDAVLNHRCAQF**

11 Aco005712/1-909 95.9% 28.3% **RWFLELGAKAEELASLGFTVIWLPPPTESVSPEGYMPKDLYNLNSRYGSMEELRDLVKRFHEVGIKVLGDVVLNHRCAHY**

12 Atrichopoda_scaffold00078.76/1-997 96.3% 24.0% **RWYSELYEKAADIVSLGFTVIWLPPPTESVSPEGYMPKDLYNLNSRYGTIEELKTLVRRFHEVGIKVLGDAVLNHRCAHY**

13 Atrichopoda_scaffold00110.101/1-977 99.0% 25.7% **RWYLELAPKAADLSHCGITAVWLPPPTESVAPQGYMPSDLYNLNSAYGTVDELKQCIEEFHSQDLLALGDVVLNHRCAQK**

14 Macuminata/1-914 95.9% 27.2% **RWYSELSDKAKELSSLGFTVIWLPPPTESVSPEGYMPKDLYNLNSRYGSLEELKDLVNSFHEVGIKVLGDAVLNHRCAHF**

15 Spipo8G0033500/1-762 93.2% 31.1% **KWYSELYAKADEIASMGFTTIWLPPPTESVSPEGYMPKDLYNLNSRYGNIEELKSLVKKFHEVGIRVLGDVVLNHRCAHA**

16 Os01g51754/1-515 65.2% 36.0% **KWYVELGSKAKELSSMGFTIVWSPPPTDSVSPEGYMPRDLYNLNSRYGTMEELKEAVKRFHEAGMKVLGDAVLNHRCAQF**

17 Zosma95g00270/1-899 95.6% 25.4% **NWYSEISSKAGELSKLGITIVWLPPPTESVSAQGYMPSDLYNLNSSYGSMEDLKNAINTFHSFGIKVVGDVVLNHRCAQH**

18 Bradi2g48150/1-868 94.9% 27.5% **KWYVELGAKAKELASLGFTIVWSPPPTDSVSPEGYMPRDLYNLNSRYGTIEELKQLVNIFHEAGVKVLGDAVLNHRCAQF**

19 AT1G69830/1-887 94.3% 25.6% **RWYLELQEKADELASLGFTVLWLPPPTESVSPEGYMPKDLYNLNSRYGTIDELKDTVKKFHKVGIKVLGDAVLNHRCAHF**

cov pid  **801**  **. . . . : . . .** **880**

1 Cre08.g362450/1-706 100.0% 100.0% **QGSDGKWNKFGGRLAWDASAICSNNPSFGGRGNPKQG----DDYAAAPNIDHSQERIRNDIVQWMKYLRNSIGFDGWRFD**

2 CHBRA206g00120/1-1039 94.3% 23.1% **QGPNGVWNRFGGKLAWDNRAIVCDDAHFDGAGNRSSG----DSFHAAPNIDHSQGFVRKDITEWLQWLRMEIGYDGWRLD**

3 AagrBONN_Sc2ySwM_362.1200.1/1-412 57.8% 29.9% **QNSQGIWNVYGGKMNWDARAIVSDDPNFQGQGNHSSG----ENFHAAPNIDHSQDFVRRDLCEWLQWLKSEVGFDGWRFD**

4 Mapoly0024s0059/1-996 98.9% 25.0% **QSPNGVWNIFGGKLAWGPEAIVKDDPNFQGRGNPSSG----DFFHAAPNIDHSQDFIRRDIKEWMKWLRSEIGFDGWRLD**

5 Pp3c21_19790/1-956 98.9% 25.6% **QSPNGVWNIFGGKLAWGPEAIVGDDPNFQGRGNPKSG----DFFHAAPNVDHSQKFVRKDIMEWMQWLRTEFGFDGWRLD**

6 Pp3c22_22380/1-950 98.9% 26.0% **QSPNGVWNRFGGKLNWGPEAIVRDDPNFQGQGNPKSG----DFFHAAPNIDHSQDFVRRDIIEWMKWLRSDFGFDGWRLD**

7 Selmo_127605/1-400 56.7% 45.3% **QNSNGVWNIFGGKLSWGPEAIVNDDPNFQGRGNPSSG----DIFHAAPNIDHSQAFVRKDIKEYLDWLKTEIGYDGWRLD**

8 Sacu_v1.1_s0008.g003998/1-993 98.7% 26.1% **KGPNGIWNVFGGKLAWGPDAIVRDDPNFQGRGNPSSG----DFFHAAPNIDHSQEFVRKDIKEWMKWLRTEIGFDGWRLD**

9 Sacu_v1.1_s0102.g019864:1-1500/1-1500 98.7% 16.9% **KGPNGIWNVFGGKLAWGPEAIVRDDPNFQGRGNPSSGNHLSDFFHAAPNIDHSQDFVRKDIKEWMKWLRTEIGFDGWRLD**

10 Azfi_s0015.g013850/1-998 98.6% 25.3% **KGPNGVWNVFGGKLAWGPEAIVRDDPNFQGRGNPSSG----DFFHAAPNIDHSQDFVQRDIKEWMKWLRTEIGFDGWRLD**

11 Aco005712/1-909 95.9% 28.3% **KNQNGIWNIFGGRLNWDDRAIVADDPHFHGRGNKSSG----DHFHAAPNIDHSQEFVRRDLKEWLCWLREEIGYDGWRLD**

12 Atrichopoda_scaffold00078.76/1-997 96.3% 24.0% **KNQNGVWNIFGGRLNWDDRAIVADDPHFQGRGNKSSG----DNFHAAPNIDHSQDFVRNDLKEWLNWLRNEIGYDGWRLD**

13 Atrichopoda_scaffold00110.101/1-977 99.0% 25.7% **QSPNGVWNIFGGKLAWGPEAIVCDDPNFQGLGNPSSG----DIFHAAPNVDHSQEFVRRDIKEWLNWLRSEIGFDGWRLD**

14 Macuminata/1-914 95.9% 27.2% **QNKNGIWNVFGGRLNWDDRAIVADDPHFQGRGNKSSG----DNFHAAPNIDHSQDFVRRDLKEWLCWLRKEVGYDGWRLD**

15 Spipo8G0033500/1-762 93.2% 31.1% **KNQNGVWNIFGGRLAWDDRAVVSDDPHFQGRGNKSSG----DNFHAAPNIDHSQEFVRKDLKEWLLWLREEIGYDGWRLD**

16 Os01g51754/1-515 65.2% 36.0% **QNQNGVWNIFGGRLNWDDRAVVADDPHFQGRGNKSSG----DNFHAAPNIDHSQEFVRSDLKEWLCWMRKEVGYDGWRLD**

17 Zosma95g00270/1-899 95.6% 25.4% **QNKNGIWNIFGGRLNWDDRAIVADDPHYQGRGNKSSG----ENFHAAPNIDHSQEFVRRDLKEWLCWLRKEIGFDGWRLD**

18 Bradi2g48150/1-868 94.9% 27.5% **QNQNGVWNIFGGRINWDDRAVVADDPHFQGRGNKSSG----DNFHAAPNIDHSQDFVRNDLKEWLCWMRKEVGYDGWRLD**

19 AT1G69830/1-887 94.3% 25.6% **KNQNGVWNLFGGRLNWDDRAVVADDPHFQGRGNKSSG----DNFHAAPNIDHSQDFVRKDIKEWLCWMMEEVGYDGWRLD**

cov pid  **881**  **. 9 . . . . : .** **960**

1 Cre08.g362450/1-706 100.0% 100.0% **FVRGYLGSYCKQYIDETVPAMAFGEYWDSCEYTDGVLNYNQDAHRQRTVNWCDSTGGTSAAFDFTTKGILQEAVGRREYW**

2 CHBRA206g00120/1-1039 94.3% 23.1% **FVRGFWGGHVKEYIEGSNPWFAVGEYWDSLSYTYGEMDYNQDAHRQRIIDWMNATGGNAGAFDVTTKGILHTAIEKCEYW**

3 AagrBONN_Sc2ySwM_362.1200.1/1-412 57.8% 29.9% **YVRGFWGGHVKEYIDASQPSFSVGEYWDCMSYSNGQLDFNQNPHRQRIVNWINATGDKAAAFDFTTKGVLHAAIEKCEYW**

4 Mapoly0024s0059/1-996 98.9% 25.0% **YVRGFWGGYVKEYIEATDPAFSIGEYWDSLAYDGGQVSYNQDAHRQRIINWINATGGTSSAFDVTTKGILHSAL-HNEYW**

5 Pp3c21_19790/1-956 98.9% 25.6% **FVRGFWGGYVKEYIEATKPAFAIGEYWDSLSYEGGQVSYNQDAHRQRIVNWINATGGTSSAFDVTTKGILHSAL-HGEFW**

6 Pp3c22_22380/1-950 98.9% 26.0% **FVRGFWGGYVKEYIEATKPAFAIGEYWDSLAYEGGQVSYNQDAHRQRIVNWINAAGGTSSAFDVTTKGILHSAL-HGEFW**

7 Selmo_127605/1-400 56.7% 45.3% **FVRGFWGGYVKEYIEASEPAFAIGEYWDSLLYEGGNVAYNQDAHRQRIIDWINATGGTSSAFDVTTKGILHAAL-HNEYW**

8 Sacu_v1.1_s0008.g003998/1-993 98.7% 26.1% **FVRGFWGGYVKEYIEATDPAFAIGEYWDSLAYEGGNVCYNQDAHRQRIVNWINATGGTSSAFDVTTKGILHSAL-HNQYW**

9 Sacu_v1.1_s0102.g019864:1-1500/1-1500 98.7% 16.9% **FVRGFWGGYVKEYIEATEPAFAIGEYWDSLAYEGGNVCYNQDAHRQRIINWINATGGTSSAFDVTTKGILHSAL-HSQYW**

10 Azfi_s0015.g013850/1-998 98.6% 25.3% **FVRGFWGGYVKDYIEATDPAFAIGEYWDSLAYEGGNVCYNQDAHRQRIINWINATGGTSSAFDVTTKGILHSAL-HNQYW**

11 Aco005712/1-909 95.9% 28.3% **FVRGFWGGYVKDYLEATEPFFAVGEYWDSLSYTYSEMDYNQDAHRQRIIDWINATNGTAGAFDVTTKGILHSALGRCEYW**

12 Atrichopoda_scaffold00078.76/1-997 96.3% 24.0% **FVRGFWGGYVKDYLDATEPYFAVGEYWDSLSYTYGEMDHNQDAHRQRIIDWINATNGTAGAFDVTTKGILHSALGKCEYW**

13 Atrichopoda_scaffold00110.101/1-977 99.0% 25.7% **FVRGFSGGYVKEYIEASNPAFAIGEYWDSLAYEGGNLCYNQDAHRQRIVNWINATSGTSSAFDVTSKGILHSAL-HNQYW**

14 Macuminata/1-914 95.9% 27.2% **FVRGFWGGYVKDYMEATEPYFAVGEYWDSLSYTYGDMDHNQDAHRQRIVDWINATNGTAGAFDVTTKGILHSALEKCEYW**

15 Spipo8G0033500/1-762 93.2% 31.1% **FVRGFWGGYVKDYLEASEPYFAVGEYWDSLSYTYGQMDYSQDAHRQRIIDWINATGGNASAFDVTTKGILHAALEKCEYW**

16 Os01g51754/1-515 65.2% 36.0% **FVRGFWGGYVHDYLEASEPYFAVGEYWDSLSYTYGEMDYNQDAHRQRIVDWINATNGTAGAFDVTTKGILHSALERSEYW**

17 Zosma95g00270/1-899 95.6% 25.4% **FARGFWGGYMKDYMEASEPYFSVGEFWDSLSYTYGEMDHNQDAHRQRCIDWINATNGTSAAFDVTLKGILHTTLEKFEYW**

18 Bradi2g48150/1-868 94.9% 27.5% **FVRGFWGGYVKDYLEASEPYFAVGEYWDSLSYTYGEMDYNQDAHRQRIVDWINATSGTAGAFDVTTKGILHMALERSEYW**

19 AT1G69830/1-887 94.3% 25.6% **FVRGFWGGYVKDYMDASKPYFAVGEYWDSLSYTYGEMDYNQDAHRQRIVDWINATSGAAGAFDVTTKGILHTALQKCEYW**

cov pid  **961**  **. . . 0 . . . .** **1040**

1 Cre08.g362450/1-706 100.0% 100.0% **RLVDSQGRPPGVMGMWPSRAITFIDNHDTGSTLNHWPFPSRNLPEGYAYILTHPGTPCVFYDHFYQEENNLRKIILDLLK**

2 CHBRA206g00120/1-1039 94.3% 23.1% **RLTDSERKPPGVVGWWPSRAVTFIENHDSGSTQGHWRFPQGREMLGYAYILTHPGTPTVFYDHWF--ANHLKEPIRILLA**

3 AagrBONN_Sc2ySwM_362.1200.1/1-412 57.8% 29.9% **RLTDEKRKPPGVLGWWSSRAVTFIDNHDTGSTQGHWRFPAGKEVQGYAYILTHPGHVAVFYDHIK--DPKLREPIRKLIA**

4 Mapoly0024s0059/1-996 98.9% 25.0% **RLIDPQGKPPGVMGWWPSRAVTFLENHDTGSTQGHWPFPRDKLMQGYAYILTHPGTPVIFYDHFY--DFGLHDQIAELIA**

5 Pp3c21_19790/1-956 98.9% 25.6% **RLIDPQGKPPGVMGWWPSRAVTFLENHDTGSTQGHWPFPRDKLMMGYAYILTHPGTPVIFHDHFY--DFGLHDQIAELIA**

6 Pp3c22_22380/1-950 98.9% 26.0% **RLIDPQGKPPGVMGWWPSRAVTFLENHDTGSTQGHWPFPRDKLMMGYAYILTHPGTPVIFHDHFY--DFGLHDQIADLIA**

7 Selmo_127605/1-400 56.7% 45.3% **RLIDPRQKPPGVMGWWPSRAVTFLENHDTGSTQGHWPFPRDKLLQGYAYILTHPGTPVIFYDHFY--DFGLRDPIVDLIA**

8 Sacu_v1.1_s0008.g003998/1-993 98.7% 26.1% **RLIDPNGKPPGVMGWWPSRAVTFLENHDTGSTQGHWPFPRDKLMQGYAYILTHPGTPVIFYDHFY--DFGLHDPIAELIA**

9 Sacu_v1.1_s0102.g019864:1-1500/1-1500 98.7% 16.9% **RLIDPNGKPPGVMGWWPSRAVTFLENHDTGSTQGHWPFPRDKLMQGYAYILTHPGTPVIFYDHFY--DFGLREPITELIA**

10 Azfi_s0015.g013850/1-998 98.6% 25.3% **RLIDPSGKPPGVMGWWPSRAVTFLENHDTGSTQGHWPFPRDKLMQGYAYILTHPGTPVIFYDHFY--DFGLRDAIAELIA**

11 Aco005712/1-909 95.9% 28.3% **RLSDQKGKPPGVIGWWPSRAVTFIENHDTGSTQGHWRFPSGTEMQGYAYTLTHPGTPAVFYDHIF---SHYQPEISRLIS**

12 Atrichopoda_scaffold00078.76/1-997 96.3% 24.0% **RLSDQKGKPPGVVGWWPSRAVTFIENHDTGSTQGHWRFPSGKEMQGYAYILTHPGTPAVFYDHIF---SHYRDEISALIG**

13 Atrichopoda_scaffold00110.101/1-977 99.0% 25.7% **RLIDPQGKPTGVMGWWPSRAVTFLENHDTGSTQGHWPFPREKLTQGYAYILTHPGTPVIFYDHFY--DFGLRDVITELIE**

14 Macuminata/1-914 95.9% 27.2% **RLSDQNGKPPGVVGWWASRAVTFIENHDTGSTQGHWRFPSGKEMQGYAYILTHPGTPAVFYDHIF---SHYQQEISRLIS**

15 Spipo8G0033500/1-762 93.2% 31.1% **RMSDEKGKPPGVMGWWPSRAVTFIENHDTGSTQGHWRFPSGKEMQGYAYILTHSGTPTVFYDHIV---SHYQREVAALIS**

16 Os01g51754/1-515 65.2% 36.0% **RLSDEKGKPPGVLGWWPSRAVTFIENHDTGSTQGHWRFPFGMELQGYVYILTHPGTPAIFYDHIF---SHLQPEIAKLIS**

17 Zosma95g00270/1-899 95.6% 25.4% **RLSDERGKPPGVAGWWPSRAVTFIENHDTGSTQGHWRFPAGKEMQGYTYILTHAGTPTVFYDHLC---SHYNQEIGKLIA**

18 Bradi2g48150/1-868 94.9% 27.5% **RLSDEKGKPPGVLGWWPSRAVTFIENHDTGSTQGHWRFPYGMEMQGYVYILTHPGTPAVFYDHVF---SHLQQDIAKLIS**

19 AT1G69830/1-887 94.3% 25.6% **RLSDPKGKPPGVVGWWPSRAVTFIENHDTGSTQGHWRFPEGKEMQGYAYILTHPGTPAVFFDHIF---SDYHSEIAALLS**

cov pid **1041**  **: . . . . 1 . .** **1120**

1 Cre08.g362450/1-706 100.0% 100.0% **VRRRNGLNARSKVVMKKSAADVYAAMIDDKVAVKLGPGDWSPNQSGIKVNGKELKVAASG--------------------**

2 CHBRA206g00120/1-1039 94.3% 23.1% **LRRRQGIHCRSVVRIQKAEKEVYGACIDEKVCMKIGPGHFDPPC-DET--KTWVCVLE-G--------------------**

3 AagrBONN_Sc2ySwM_362.1200.1/1-412 57.8% 29.9% **LRKKANIHSKSNTNILEARKEFYAAIIDDRIVVKIGPGEYWPSG------HNWKLALE-G--------------------**

4 Mapoly0024s0059/1-996 98.9% 25.0% **ARKRTAVHCRSPVKIFHANIEGYVAQVGENLVMKLGRLDWNPSK-ENNLAGSWERFLDRG--------------------**

5 Pp3c21_19790/1-956 98.9% 25.6% **VRTRTGVHCRSPVKIFQANFEGYAAQIGENLVMKIGHLDWNPSK-QNNLPGSWDRCVDKG--------------------**

6 Pp3c22_22380/1-950 98.9% 26.0% **VRTRTGVHCRSKVKIFQANFEGYAAQVGDNLVMKIGHLDWNPSK-QNNLAGSWNRCTDKG--------------------**

7 Selmo_127605/1-400 56.7% 45.3% **ARNRTGINCRSPVKIFHANNDGYVAKVGEQLVVKLGRFDWNPSK-QNDLIGNWKRSVGQG--------------------**

8 Sacu_v1.1_s0008.g003998/1-993 98.7% 26.1% **VRKRTGVNCRSPVKILQATNQGYAARIGDSLIVKLGFIDWNPSK-ENSLEGKWNRCLDKG--------------------**

9 Sacu_v1.1_s0102.g019864:1-1500/1-1500 98.7% 16.9% **VRKRTDVHCRSTVKIYQATNQGYAAQVGDNLVMKLGHLDWNPSK-ENNLEGKWSRCVDKDDSFSDITVRHRGNLEMLEQL**

10 Azfi_s0015.g013850/1-998 98.6% 25.3% **VRNRTGVNCRSPVKIYQATNQGYASQIGDNLVIKMGHLDWNPSK-ENNLEGKWNRCLDKG--------------------**

11 Aco005712/1-909 95.9% 28.3% **VRRRQEIHCRSKIKIIKAERDVYAAEIDEKVAVKIGPGHYEPSN-GPK---NWVLAAE-G--------------------**

12 Atrichopoda_scaffold00078.76/1-997 96.3% 24.0% **LRHRKKINCRSTVEIRKAERDVYAATIDDRVTVKIGPGHYEPPS-GSQ---NWSLIAQ-G--------------------**

13 Atrichopoda_scaffold00110.101/1-977 99.0% 25.7% **ARSRAGIHCRSSVKIYHANNEGYVAQIGDTLLMKIGHLDWNPSK-ENQLEGSWQKFVDKG--------------------**

14 Macuminata/1-914 95.9% 27.2% **VRNENKIHCRSTVKIVKAERDVYAAEIDGKLAVKIGPGHYEPPD-GPT---KWVVAAE-G--------------------**

15 Spipo8G0033500/1-762 93.2% 31.1% **LRNRNKIHCRSTVKIKKAERDVYAAEIDERVVMKIGPGYYEPE--GPR---SWSVAAEGG--------------------**

16 Os01g51754/1-515 65.2% 36.0% **IRNRQKIHCRS---------------------------------------------------------------------**

17 Zosma95g00270/1-899 95.6% 25.4% **LRHRKKIHCRSKVKITKAEKEVYAAIIDDKVAMKIGPGHYEPGD-----HNKWKVAAE-G--------------------**

18 Bradi2g48150/1-868 94.9% 27.5% **VRRRLKIHCRSKIKILKAEQNLYAAEIDEKVTMKIGSGHFEPT--GPI---NWIVAVE-G--------------------**

19 AT1G69830/1-887 94.3% 25.6% **LRNRQKLHCRSEVNIDKSERDVYAAIIDEKVAMKIGPGHYEPPN-GSQ---NWSVAVE-G--------------------**

cov pid **1121**  **. . : . . . . 2** **1200**

1 Cre08.g362450/1-706 100.0% 100.0% **--------------------------------------------------------------------------------**

2 CHBRA206g00120/1-1039 94.3% 23.1% **--------------------------------------------------------------------------------**

3 AagrBONN_Sc2ySwM_362.1200.1/1-412 57.8% 29.9% **--------------------------------------------------------------------------------**

4 Mapoly0024s0059/1-996 98.9% 25.0% **--------------------------------------------------------------------------------**

5 Pp3c21_19790/1-956 98.9% 25.6% **--------------------------------------------------------------------------------**

6 Pp3c22_22380/1-950 98.9% 26.0% **--------------------------------------------------------------------------------**

7 Selmo_127605/1-400 56.7% 45.3% **--------------------------------------------------------------------------------**

8 Sacu_v1.1_s0008.g003998/1-993 98.7% 26.1% **--------------------------------------------------------------------------------**

9 Sacu_v1.1_s0102.g019864:1-1500/1-1500 98.7% 16.9% **YRGSECVYDDNSEIDAKLIAMAFACPPTRLLLFPDHLPPQHRLHPSSSPVPLFFHPPPFRLCFRILSISSSSSSLHKITE**

10 Azfi_s0015.g013850/1-998 98.6% 25.3% **--------------------------------------------------------------------------------**

11 Aco005712/1-909 95.9% 28.3% **--------------------------------------------------------------------------------**

12 Atrichopoda_scaffold00078.76/1-997 96.3% 24.0% **--------------------------------------------------------------------------------**

13 Atrichopoda_scaffold00110.101/1-977 99.0% 25.7% **--------------------------------------------------------------------------------**

14 Macuminata/1-914 95.9% 27.2% **--------------------------------------------------------------------------------**

15 Spipo8G0033500/1-762 93.2% 31.1% **--------------------------------------------------------------------------------**

16 Os01g51754/1-515 65.2% 36.0% **--------------------------------------------------------------------------------**

17 Zosma95g00270/1-899 95.6% 25.4% **--------------------------------------------------------------------------------**

18 Bradi2g48150/1-868 94.9% 27.5% **--------------------------------------------------------------------------------**

19 AT1G69830/1-887 94.3% 25.6% **--------------------------------------------------------------------------------**

cov pid **1201**  **. . . . : . . .** **1280**

1 Cre08.g362450/1-706 100.0% 100.0% **--------------------------------------------------------------------------------**

2 CHBRA206g00120/1-1039 94.3% 23.1% **--------------------------------------------------------------------------------**

3 AagrBONN_Sc2ySwM_362.1200.1/1-412 57.8% 29.9% **--------------------------------------------------------------------------------**

4 Mapoly0024s0059/1-996 98.9% 25.0% **--------------------------------------------------------------------------------**

5 Pp3c21_19790/1-956 98.9% 25.6% **--------------------------------------------------------------------------------**

6 Pp3c22_22380/1-950 98.9% 26.0% **--------------------------------------------------------------------------------**

7 Selmo_127605/1-400 56.7% 45.3% **--------------------------------------------------------------------------------**

8 Sacu_v1.1_s0008.g003998/1-993 98.7% 26.1% **--------------------------------------------------------------------------------**

9 Sacu_v1.1_s0102.g019864:1-1500/1-1500 98.7% 16.9% **LDEGRPPPPPWQKFSTDAPPDDGSNPKPRAFRPAPWQQGSRDHRPQEPLRKQGAQTKRNFDGGAVEAEADKSALATIVEK**

10 Azfi_s0015.g013850/1-998 98.6% 25.3% **--------------------------------------------------------------------------------**

11 Aco005712/1-909 95.9% 28.3% **--------------------------------------------------------------------------------**

12 Atrichopoda_scaffold00078.76/1-997 96.3% 24.0% **--------------------------------------------------------------------------------**

13 Atrichopoda_scaffold00110.101/1-977 99.0% 25.7% **--------------------------------------------------------------------------------**

14 Macuminata/1-914 95.9% 27.2% **--------------------------------------------------------------------------------**

15 Spipo8G0033500/1-762 93.2% 31.1% **--------------------------------------------------------------------------------**

16 Os01g51754/1-515 65.2% 36.0% **--------------------------------------------------------------------------------**

17 Zosma95g00270/1-899 95.6% 25.4% **--------------------------------------------------------------------------------**

18 Bradi2g48150/1-868 94.9% 27.5% **--------------------------------------------------------------------------------**

19 AT1G69830/1-887 94.3% 25.6% **--------------------------------------------------------------------------------**

cov pid **1281**  **. 3 . . . . : .** **1360**

1 Cre08.g362450/1-706 100.0% 100.0% **--------------------------------------------------------------------------------**

2 CHBRA206g00120/1-1039 94.3% 23.1% **--------------------------------------------------------------------------------**

3 AagrBONN_Sc2ySwM_362.1200.1/1-412 57.8% 29.9% **--------------------------------------------------------------------------------**

4 Mapoly0024s0059/1-996 98.9% 25.0% **--------------------------------------------------------------------------------**

5 Pp3c21_19790/1-956 98.9% 25.6% **--------------------------------------------------------------------------------**

6 Pp3c22_22380/1-950 98.9% 26.0% **--------------------------------------------------------------------------------**

7 Selmo_127605/1-400 56.7% 45.3% **--------------------------------------------------------------------------------**

8 Sacu_v1.1_s0008.g003998/1-993 98.7% 26.1% **--------------------------------------------------------------------------------**

9 Sacu_v1.1_s0102.g019864:1-1500/1-1500 98.7% 16.9% **LRTIHDSLDASEPGTTGIAFNDSASSQSNADETHSSSSVSATEDKFPWEKPSESTEQETVVQVSQRRNPTRADLLIPPDE**

10 Azfi_s0015.g013850/1-998 98.6% 25.3% **--------------------------------------------------------------------------------**

11 Aco005712/1-909 95.9% 28.3% **--------------------------------------------------------------------------------**

12 Atrichopoda_scaffold00078.76/1-997 96.3% 24.0% **--------------------------------------------------------------------------------**

13 Atrichopoda_scaffold00110.101/1-977 99.0% 25.7% **--------------------------------------------------------------------------------**

14 Macuminata/1-914 95.9% 27.2% **--------------------------------------------------------------------------------**

15 Spipo8G0033500/1-762 93.2% 31.1% **--------------------------------------------------------------------------------**

16 Os01g51754/1-515 65.2% 36.0% **--------------------------------------------------------------------------------**

17 Zosma95g00270/1-899 95.6% 25.4% **--------------------------------------------------------------------------------**

18 Bradi2g48150/1-868 94.9% 27.5% **--------------------------------------------------------------------------------**

19 AT1G69830/1-887 94.3% 25.6% **--------------------------------------------------------------------------------**

cov pid **1361**  **. . . 4 . . . .** **1440**

1 Cre08.g362450/1-706 100.0% 100.0% **--------------------------------------------------------------------------------**

2 CHBRA206g00120/1-1039 94.3% 23.1% **--------------------------------------------------------------------------------**

3 AagrBONN_Sc2ySwM_362.1200.1/1-412 57.8% 29.9% **--------------------------------------------------------------------------------**

4 Mapoly0024s0059/1-996 98.9% 25.0% **--------------------------------------------------------------------------------**

5 Pp3c21_19790/1-956 98.9% 25.6% **--------------------------------------------------------------------------------**

6 Pp3c22_22380/1-950 98.9% 26.0% **--------------------------------------------------------------------------------**

7 Selmo_127605/1-400 56.7% 45.3% **--------------------------------------------------------------------------------**

8 Sacu_v1.1_s0008.g003998/1-993 98.7% 26.1% **--------------------------------------------------------------------------------**

9 Sacu_v1.1_s0102.g019864:1-1500/1-1500 98.7% 16.9% **LKRLRMISPELQERLKIGKLGVTRSIVISLQQQWRTLELVKVRCQGPAANNIKKTLADLEENTGGLVIWRDKNAVVVYRG**

10 Azfi_s0015.g013850/1-998 98.6% 25.3% **--------------------------------------------------------------------------------**

11 Aco005712/1-909 95.9% 28.3% **--------------------------------------------------------------------------------**

12 Atrichopoda_scaffold00078.76/1-997 96.3% 24.0% **--------------------------------------------------------------------------------**

13 Atrichopoda_scaffold00110.101/1-977 99.0% 25.7% **--------------------------------------------------------------------------------**

14 Macuminata/1-914 95.9% 27.2% **--------------------------------------------------------------------------------**

15 Spipo8G0033500/1-762 93.2% 31.1% **--------------------------------------------------------------------------------**

16 Os01g51754/1-515 65.2% 36.0% **--------------------------------------------------------------------------------**

17 Zosma95g00270/1-899 95.6% 25.4% **--------------------------------------------------------------------------------**

18 Bradi2g48150/1-868 94.9% 27.5% **--------------------------------------------------------------------------------**

19 AT1G69830/1-887 94.3% 25.6% **--------------------------------------------------------------------------------**

cov pid **1441**  **: . . . . 5 . .** **1520**

1 Cre08.g362450/1-706 100.0% 100.0% **--------------------------------------------------------------------------------**

2 CHBRA206g00120/1-1039 94.3% 23.1% **--------------------------------------------------------------------------------**

3 AagrBONN_Sc2ySwM_362.1200.1/1-412 57.8% 29.9% **--------------------------------------------------------------------------------**

4 Mapoly0024s0059/1-996 98.9% 25.0% **--------------------------------------------------------------------------------**

5 Pp3c21_19790/1-956 98.9% 25.6% **--------------------------------------------------------------------------------**

6 Pp3c22_22380/1-950 98.9% 26.0% **--------------------------------------------------------------------------------**

7 Selmo_127605/1-400 56.7% 45.3% **--------------------------------------------------------------------------------**

8 Sacu_v1.1_s0008.g003998/1-993 98.7% 26.1% **--------------------------------------------------------------------------------**

9 Sacu_v1.1_s0102.g019864:1-1500/1-1500 98.7% 16.9% **VGFNPESDVGVKPEIETDISVAMGIEQNGATSIYGNHDRKNDETEMESLLDSLGPRYEKWTGLRPVPIDADLLPPEVPNY**

10 Azfi_s0015.g013850/1-998 98.6% 25.3% **--------------------------------------------------------------------------------**

11 Aco005712/1-909 95.9% 28.3% **--------------------------------------------------------------------------------**

12 Atrichopoda_scaffold00078.76/1-997 96.3% 24.0% **--------------------------------------------------------------------------------**

13 Atrichopoda_scaffold00110.101/1-977 99.0% 25.7% **--------------------------------------------------------------------------------**

14 Macuminata/1-914 95.9% 27.2% **--------------------------------------------------------------------------------**

15 Spipo8G0033500/1-762 93.2% 31.1% **--------------------------------------------------------------------------------**

16 Os01g51754/1-515 65.2% 36.0% **--------------------------------------------------------------------------------**

17 Zosma95g00270/1-899 95.6% 25.4% **--------------------------------------------------------------------------------**

18 Bradi2g48150/1-868 94.9% 27.5% **--------------------------------------------------------------------------------**

19 AT1G69830/1-887 94.3% 25.6% **--------------------------------------------------------------------------------**

cov pid **1521**  **. . : . . . . 6** **1600**

1 Cre08.g362450/1-706 100.0% 100.0% **-FQFA----------------------------------------------VWEGQH-----------------------**

2 CHBRA206g00120/1-1039 94.3% 23.1% **-QDFK----------------------------------------------VWEVSHN----------------------**

3 AagrBONN_Sc2ySwM_362.1200.1/1-412 57.8% 29.9% **-PDYK----------------------------------------------VWERGS-----------------------**

4 Mapoly0024s0059/1-996 98.9% 25.0% **-SEYQ----------------------------------------------LWERK------------------------**

5 Pp3c21_19790/1-956 98.9% 25.6% **--EYQ----------------------------------------------LWERI------------------------**

6 Pp3c22_22380/1-950 98.9% 26.0% **--EYQ----------------------------------------------LWERK------------------------**

7 Selmo_127605/1-400 56.7% 45.3% **-SDYQ----------------------------------------------VWEEE------------------------**

8 Sacu_v1.1_s0008.g003998/1-993 98.7% 26.1% **-ADYQ----------------------------------------------IWERS------------------------**

9 Sacu_v1.1_s0102.g019864:1-1500/1-1500 98.7% 16.9% **KPPFRLLPSGVWAGLTDAELTNLRRLARPLAPHFVLGRNKGQQGLAVAMLKLWEKTEIVKIAVKKRVQNTNNEMMAEQIR**

10 Azfi_s0015.g013850/1-998 98.6% 25.3% **-ADYQ----------------------------------------------IWERS------------------------**

11 Aco005712/1-909 95.9% 28.3% **-KDYK----------------------------------------------VWETS------------------------**

12 Atrichopoda_scaffold00078.76/1-997 96.3% 24.0% **-QDYKRMPWSNLALICSKFESPGTVKTCLKRANVLPLICLSPLVVMFPKLSSWTLTSSFLKPFIARNRAHVR--------**

13 Atrichopoda_scaffold00110.101/1-977 99.0% 25.7% **-GDYQ----------------------------------------------LWLRP------------------------**

14 Macuminata/1-914 95.9% 27.2% **-RDYK----------------------------------------------VWETS------------------------**

15 Spipo8G0033500/1-762 93.2% 31.1% **--EYK----------------------------------------------VWEAI------------------------**

16 Os01g51754/1-515 65.2% 36.0% **----K----------------------------------------------VWEVSS-----------------------**

17 Zosma95g00270/1-899 95.6% 25.4% **-RDYK----------------------------------------------VWETS------------------------**

18 Bradi2g48150/1-868 94.9% 27.5% **-QDYK----------------------------------------------IWEASS-----------------------**

19 AT1G69830/1-887 94.3% 25.6% **-RDYK----------------------------------------------VWETS------------------------**

cov pid **1601**  **. ]** **1614**

1 Cre08.g362450/1-706 100.0% 100.0% **--------------**

2 CHBRA206g00120/1-1039 94.3% 23.1% **--------------**

3 AagrBONN_Sc2ySwM_362.1200.1/1-412 57.8% 29.9% **--------------**

4 Mapoly0024s0059/1-996 98.9% 25.0% **--------------**

5 Pp3c21_19790/1-956 98.9% 25.6% **--------------**

6 Pp3c22_22380/1-950 98.9% 26.0% **--------------**

7 Selmo_127605/1-400 56.7% 45.3% **--------------**

8 Sacu_v1.1_s0008.g003998/1-993 98.7% 26.1% **--------------**

9 Sacu_v1.1_s0102.g019864:1-1500/1-1500 98.7% 16.9% **RLTGGVLLSRDKFF**

10 Azfi_s0015.g013850/1-998 98.6% 25.3% **--------------**

11 Aco005712/1-909 95.9% 28.3% **--------------**

12 Atrichopoda_scaffold00078.76/1-997 96.3% 24.0% **--------------**

13 Atrichopoda_scaffold00110.101/1-977 99.0% 25.7% **--------------**

14 Macuminata/1-914 95.9% 27.2% **--------------**

15 Spipo8G0033500/1-762 93.2% 31.1% **--------------**

16 Os01g51754/1-515 65.2% 36.0% **--------------**

17 Zosma95g00270/1-899 95.6% 25.4% **--------------**

18 Bradi2g48150/1-868 94.9% 27.5% **--------------**

19 AT1G69830/1-887 94.3% 25.6% **-------------**

Alignment as multi-fasta:

>Cre08.g362450/1-706

-----------------------MDRSLLSRTSPRMQLGR--------------------------------

------------------------------------------------------------------------

-------------------------PQQLPPPTVPLAQVPRLQRR--CV-----------------------

------------VGSRVCQPVVAVRPGRASAGRGGRLVVSSVDMSNSPLSSMDAGEGLDIMFDNNSDAECTV

VTVEGKDKAHLLMSLTGG-----------FSSAGLTVISASITSDDGRVLDVFRVQTADGKKV--PEEQFPS

VREHILSVT--ATSSRSSMPAIYGIVAAAEVERLKPLRSQSTQND---------VDALELAAAEMTQAVAEL

VATERDIIRMRASNADARTLQTKEANRTEAAAGL------------------------ER------------

-------------------------------------KMAA-MQAVLAARRNLATE----------------

------PEKPKSPTEKLLE-----------------------------------------------------

-----------------------TLKPPTPMRAAAG--------------AGSGSGSEILLQAFNWESHR-Q

KLYKQLMGRVKDISDAGFTGVWMPPPSDSVSPQGYLPRDLYSLDSAYGSEAELRELIAAFHQNNIKVIADIV

VNHRCANSQGSDGKWNKFGGRLAWDASAICSNNPSFGGRGNPKQG----DDYAAAPNIDHSQERIRNDIVQW

MKYLRNSIGFDGWRFDFVRGYLGSYCKQYIDETVPAMAFGEYWDSCEYTDGVLNYNQDAHRQRTVNWCDSTG

GTSAAFDFTTKGILQEAVGRREYWRLVDSQGRPPGVMGMWPSRAITFIDNHDTGSTLNHWPFPSRNLPEGYA

YILTHPGTPCVFYDHFYQEENNLRKIILDLLKVRRRNGLNARSKVVMKKSAADVYAAMIDDKVAVKLGPGDW

SPNQSGIKVNGKELKVAASG----------------------------------------------------

------------------------------------------------------------------------

------------------------------------------------------------------------

------------------------------------------------------------------------

------------------------------------------------------------------------

------------------------------------------------------------------------

---------FQFA----------------------------------------------VWEGQH-------

------------------------------

>CHBRA206g00120/1-1039

MGNGSKRYGSNNLHFAAVCEKGSGGLVTLPHLGGRLRGVGGARSGGHSDDGGDHSTSPRAAASAASAADGLL

IVLSALADTQPAGQSQIEWPGDDQVQTTLQKDYALTRRVKVRGKLWVKVAL---WENLHRIRISVECDVL-H

RALLHWGVTTREEA---GRQWTQPPKKIRPPGTVKYKDYAARTPLRSCYGAVSDGGEEHGGGRVGVDLEIAE

EG----------APEVIAFVLKDEETGRWHDKAGGNFIINLADLLEQRHERKTPAAAAHAAGGLRLLDGGAV

------------------------------GLLEETHDLENEVDIGNRLSAMVGLAEGGDVKVLLSTDLSGP

VLLHWGLVKRGEEQSKWTVPAKRFLPSNSTVYKKRAVQTVMKRDEGCDGDGEGSWIMV-------------D

VGGGFSELRFVLKEVDSNTWFDA--EGEDFSMPLPAAKGPKDHGGGQEGAAAGAPGQTTSGVAASDDRDRDR

NRKKTDIARLEAKVAEPVLPIPIADMAEGGGIGVNGAPAAANVVSSSSATREDFAGTIAPPAPPSPPPSPPS

PSSPSPSPSTTEVAEEEVNLAAITRSAKGTGAVPAGTVSSNSRVVITSSGPGPEELLQEIDRLAAEASENFR

RATVIAPAAPLSQQKLEQADSKPTTTLPLTSPQNVLPLPLPLSVPDRQPCPGTGSGKEILLQGFNWESHKTG

RWYNIIAEQAADIASGGFTAIWLPPPTDSISPEGYMPRDLYDLNSKYGDMEALKRVVKRLHEVGMVVLGDAV

LNHRCAHFQGPNGVWNRFGGKLAWDNRAIVCDDAHFDGAGNRSSG----DSFHAAPNIDHSQGFVRKDITEW

LQWLRMEIGYDGWRLDFVRGFWGGHVKEYIEGSNPWFAVGEYWDSLSYTYGEMDYNQDAHRQRIIDWMNATG

GNAGAFDVTTKGILHTAIEKCEYWRLTDSERKPPGVVGWWPSRAVTFIENHDSGSTQGHWRFPQGREMLGYA

YILTHPGTPTVFYDHWF--ANHLKEPIRILLALRRRQGIHCRSVVRIQKAEKEVYGACIDEKVCMKIGPGHF

DPPC-DET--KTWVCVLE-G----------------------------------------------------

------------------------------------------------------------------------

------------------------------------------------------------------------

------------------------------------------------------------------------

------------------------------------------------------------------------

------------------------------------------------------------------------

---------QDFK----------------------------------------------VWEVSHN------

------------------------------

>AagrBONN_Sc2ySwM_362.1200.1/1-412

------------------------------------------------------------------------

------------------------------------------------------------------------

----------------------MPPPSTPAP-----------------------------------------

------------------------------------------------------------------------

------------------------------------------------------------------------

-------------------PPKPK-PAEPET-----------------------------------------

------------------------------------------------------------------------

------------------------------------------------------------------------

------------------------------------------------------------------------

---------------------------------------------------------QILLQGFNWESHKSE

NWYKTIASQADDLAGLGFTSIWLPPPSQSVAPQGYLPADLYNLDSKYGTLADLKEAIAKLHSVGIQVLADIV

VNHRCAQSQNSQGIWNVYGGKMNWDARAIVSDDPNFQGQGNHSSG----ENFHAAPNIDHSQDFVRRDLCEW

LQWLKSEVGFDGWRFDYVRGFWGGHVKEYIDASQPSFSVGEYWDCMSYSNGQLDFNQNPHRQRIVNWINATG

DKAAAFDFTTKGVLHAAIEKCEYWRLTDEKRKPPGVLGWWSSRAVTFIDNHDTGSTQGHWRFPAGKEVQGYA

YILTHPGHVAVFYDHIK--DPKLREPIRKLIALRKKANIHSKSNTNILEARKEFYAAIIDDRIVVKIGPGEY

WPSG------HNWKLALE-G----------------------------------------------------

------------------------------------------------------------------------

------------------------------------------------------------------------

------------------------------------------------------------------------

------------------------------------------------------------------------

------------------------------------------------------------------------

---------PDYK----------------------------------------------VWERGS-------

------------------------------

>Mapoly0024s0059/1-996

------MEIFATMQATFTTGPPLNSTEVRKCRAPDAALHQSLLHLR---KSPKVCSVRHDDIFNSSGLSTPV

KPFFGGK------RKLYKWQRPSSELQKRENLPRLLVTASTAG-----------IGSDNSSESSFGSNFP-D

PVSLRAALKASQARVLEIENEKRDILEALRQSEAKVQEYAALMVQ--TTDEALSELEASKKLFKAELSKVLE

EKSTLQKETLLAKQDAVNLAVKIEKIAESAIQEATQRFAEDLVLKDSAAETAAAEAAAGVEESIRLAASDAA

ALVVTEASTVMEEALAAASLAKQQATKAQEALAKGMEIFEELSAAKLTTLSLQEKVSYLERELGISQGIVES

LRLELKASQ--MRTEAANARAAEA-EAAVQEVQRAAAEDGRERDD----RAKQTLEEIKATLISKTEVASVV

LQADLEALKAAYH-AAQEAGNVKEQANLRMYEAL------------------------ERSLAAAEGSAEAW

KNRALSVEGLLRRVKEEGLE---AVSSVVAEEMVAGGRMET-LLGNDSRKRDLLANGPRRETPEWMRRRIEV

GFQGLPPRS-SMPTNSEIE----------------------------------AQVPLHLPR-PDEVWSIFN

AKVKEDDLYTKQAVEKEALDEQRRALERALQKKTVK---RHPEDGEGKLESGTGSGREIVFQGFNWESWR-R

KWYLELAPKAADLKKCGITTIWMPPPTESVAPQGYMPGDLYNLNSAYGTVDELKQCIEEMHNNDILVLGDAV

LNHRCAQKQSPNGVWNIFGGKLAWGPEAIVKDDPNFQGRGNPSSG----DFFHAAPNIDHSQDFIRRDIKEW

MKWLRSEIGFDGWRLDYVRGFWGGYVKEYIEATDPAFSIGEYWDSLAYDGGQVSYNQDAHRQRIINWINATG

GTSSAFDVTTKGILHSAL-HNEYWRLIDPQGKPPGVMGWWPSRAVTFLENHDTGSTQGHWPFPRDKLMQGYA

YILTHPGTPVIFYDHFY--DFGLHDQIAELIAARKRTAVHCRSPVKIFHANIEGYVAQVGENLVMKLGRLDW

NPSK-ENNLAGSWERFLDRG----------------------------------------------------

------------------------------------------------------------------------

------------------------------------------------------------------------

------------------------------------------------------------------------

------------------------------------------------------------------------

------------------------------------------------------------------------

---------SEYQ----------------------------------------------LWERK--------

------------------------------

>Pp3c21_19790/1-956

----------------------MDTILTRSLAGVSGHLLPIGRGEFQSTSPSIHLISSYHCAGSNNNNSTIK

TPFLGER-----------------VLVSRNVNTTKALRRIWHGRVFVS------VLKGENNNGSRGFQIPDD

VESLKVALAAAEARADAAKKAEKQALEALTAMEGKSSDTVKTSRN--MKQIKLKGGND--------------

------------DADGISLAVQVEKISEAAIQKATARITEDATLKVAAAETAAAEAVLQLEERLQRAVDEAA

SAVAGETQVAIDEARAAAKVAKAQAAKSEALLNEQVNVLNELAEIEAKMLVLEEALLAAGRQLQIANGETER

VRIELDAVQ--SFIKTATARAEAA-EKTIIEVQKAASKAADEREA----SALTAIDAVKKAAKARQVADKVA

FEAEADALRSAND-ASHKASEARRLVIKSRCESL------------------------EKSLVAAEGAAAAW

RNRALTAEELLRQSRINGVE-IDSSSPVPDLLPPNIGRLEM-LPGSDAKIKDLLENGPRRETPDWMKRRLQT

GQQNLPPMQPTSITADIDA-----------------------------------AIPLELPT-PEDVWDVAK

SKVKEDDKYTVRAAEKEALDLQRNALERALQTKSLRTLVRYPEESESKTESGTGSGREIVFQGFNWESWR-R

QWWLEMSAKASDLAKCGITTIWLPPPTQSVAPQGYMPGDLYNLNSAYGGSEELKLCINEMHKHKILVLGDVV

LNHRCAQKQSPNGVWNIFGGKLAWGPEAIVGDDPNFQGRGNPKSG----DFFHAAPNVDHSQKFVRKDIMEW

MQWLRTEFGFDGWRLDFVRGFWGGYVKEYIEATKPAFAIGEYWDSLSYEGGQVSYNQDAHRQRIVNWINATG

GTSSAFDVTTKGILHSAL-HGEFWRLIDPQGKPPGVMGWWPSRAVTFLENHDTGSTQGHWPFPRDKLMMGYA

YILTHPGTPVIFHDHFY--DFGLHDQIAELIAVRTRTGVHCRSPVKIFQANFEGYAAQIGENLVMKIGHLDW

NPSK-QNNLPGSWDRCVDKG----------------------------------------------------

------------------------------------------------------------------------

------------------------------------------------------------------------

------------------------------------------------------------------------

------------------------------------------------------------------------

------------------------------------------------------------------------

----------EYQ----------------------------------------------LWERI--------

------------------------------

>Pp3c22_22380/1-950

----------------------MDTVSARSLAGMSSNLLSSPARTWHGEFRLVSQQIPLISSYHVTGSNTNN

STSVRTS--------------------FFGERSPNTSKVAKGQRRSRPDHAVVSVLKGDRITGSRDVQAPDD

VESLKAALAAAEARTDAAKQAEKKALDALAAMKNKSRDMAQNDQN--SQE----------------------

------------VFEGISLAVQVEKISESTIQKATLRITEDAELKIAAAETAAAEVILELEDQFRRAAEDAV

QAASVEAQVTIDEARAAVSAARVQAEKSEAILNKQVKALNELAEAEAKVLMLEEALLDAGRKLQLANGETER

IRIELDSAQ--RFIKTATARAEAA-ERTAEELQRAAAKEAEERAD----SAQSAINAVKKATQVRLDADKIA

FEAELDALRSAND-TSHKASEARRLVDKSRFELL------------------------ERSLLAVESATAAW

KNRALMAEKLLRLARINGAE--IDTSSLPVEQAPSVGRLEV-LPGSDVRIKDLLENGPRRETPDWMKRRLQI

GQQVLPPMQPIAINADVDA-----------------------------------LIPLQLPS-SETVWDVSK

SKVKENDKYAVRAAEKEALDLQRNAMERALQTKSIKTLVRYPEDAEEKSESGTGSGREIVFQGFNWESWR-R

QWWLEMSAKASDLSKCGITTIWLPPPTHSVAPQGYMPGDLYNLNSAYGGSEELKQCIDEMHKHNILVLGDVV

LNHRCAQKQSPNGVWNRFGGKLNWGPEAIVRDDPNFQGQGNPKSG----DFFHAAPNIDHSQDFVRRDIIEW

MKWLRSDFGFDGWRLDFVRGFWGGYVKEYIEATKPAFAIGEYWDSLAYEGGQVSYNQDAHRQRIVNWINAAG

GTSSAFDVTTKGILHSAL-HGEFWRLIDPQGKPPGVMGWWPSRAVTFLENHDTGSTQGHWPFPRDKLMMGYA

YILTHPGTPVIFHDHFY--DFGLHDQIADLIAVRTRTGVHCRSKVKIFQANFEGYAAQVGDNLVMKIGHLDW

NPSK-QNNLAGSWNRCTDKG----------------------------------------------------

------------------------------------------------------------------------

------------------------------------------------------------------------

------------------------------------------------------------------------

------------------------------------------------------------------------

------------------------------------------------------------------------

----------EYQ----------------------------------------------LWERK--------

------------------------------

>Selmo_127605/1-400 127605

------------------------------------------------------------------------

------------------------------------------------------------------------

------------------------------------------------------------------------

------------------------------------------------------------------------

------------------------------------------------------------------------

------------------------------------------------------------------------

-------------------------LDSSFCFKF--------------------------------------

------------------------------------------------------------------------

------------------------------------------------------------------------

-------------------------------------------------------------QGFNWESCR-K

RWYQDLAPKAADLSQSGITTVWFPPPTESVAPQGYMPVDLYNLNSAYGSMDELKHCIQEMHKHDLLVLGDVV

LNHRCAYKQNSNGVWNIFGGKLSWGPEAIVNDDPNFQGRGNPSSG----DIFHAAPNIDHSQAFVRKDIKEY

LDWLKTEIGYDGWRLDFVRGFWGGYVKEYIEASEPAFAIGEYWDSLLYEGGNVAYNQDAHRQRIIDWINATG

GTSSAFDVTTKGILHAAL-HNEYWRLIDPRQKPPGVMGWWPSRAVTFLENHDTGSTQGHWPFPRDKLLQGYA

YILTHPGTPVIFYDHFY--DFGLRDPIVDLIAARNRTGINCRSPVKIFHANNDGYVAKVGEQLVVKLGRFDW

NPSK-QNDLIGNWKRSVGQG----------------------------------------------------

------------------------------------------------------------------------

------------------------------------------------------------------------

------------------------------------------------------------------------

------------------------------------------------------------------------

------------------------------------------------------------------------

---------SDYQ----------------------------------------------VWEEE--------

------------------------------

>Sacu_v1.1_s0008.g003998/1-993

-----------MVSVVPMTFDAISGLPMFSKNKLDTIQPRNISSAGIHGSGSNSFSGPSLRVETLSG-SGRY

TQKLRLF------RGI------KCSGNVVSRSGSTPVEENDQGSSKAEQDAKVLVKDFDANSIGQNLD---D

IEGLKSALKAAHAKMLAAESEKADALRALAQAEARLREYATSAAE--TTESAVHEMEAAKESVSIELQNIMK

EKLATESELVVARSDAIELAVSVEKVADTILREATAHLIEEAQLKIAAAKTSAAEAAANVEERIKSAVHDTA

NAMIRETKDAIEKSFAALEAAKEKAQKSEIALFQRMQILDDMVLKEASALGLQQAESDLQRKLLAAESEIQR

LHGEVKAVL--ARAEAAEVRASTA-DEALKQFQEAANISAHEQEG----SAKKALEALKETGAARLEAARAA

FKADVEVLQSALE-TVQIAGKSKEQAYTRKHQAL------------------------ERSLAAAETLAKAW

EERALAVESLLHKSRKEGAEQYAVELNGGVIDILTGGRMET-LLGNDSRKWELLSNGPRTDTPEWMERRIEV

ALQGLPARTGSTPVEQ--E----------------------------------STLSLQLPS-PEEVWCIAT

AEVKE-DVLTREIAEKEAIDEQRRVLEKALKKKTVR---KTPQ----AMESGTGSGYEIVFQGFNWESWR-K

KWYLELGPKAADLSSCGITTIWFPPPTQSVSPQGYMPGDLYDLNSSYGTEEELKNCIEEMHNNELLVLGDAV

LNHRCAQFKGPNGIWNVFGGKLAWGPDAIVRDDPNFQGRGNPSSG----DFFHAAPNIDHSQEFVRKDIKEW

MKWLRTEIGFDGWRLDFVRGFWGGYVKEYIEATDPAFAIGEYWDSLAYEGGNVCYNQDAHRQRIVNWINATG

GTSSAFDVTTKGILHSAL-HNQYWRLIDPNGKPPGVMGWWPSRAVTFLENHDTGSTQGHWPFPRDKLMQGYA

YILTHPGTPVIFYDHFY--DFGLHDPIAELIAVRKRTGVNCRSPVKILQATNQGYAARIGDSLIVKLGFIDW

NPSK-ENSLEGKWNRCLDKG----------------------------------------------------

------------------------------------------------------------------------

------------------------------------------------------------------------

------------------------------------------------------------------------

------------------------------------------------------------------------

------------------------------------------------------------------------

---------ADYQ----------------------------------------------IWERS--------

------------------------------

>Sacu_v1.1_s0102.g019864:1-1500/1-1500

-----------MVSVALTQHDIISGLCSLSSNKVDSILQSRYACISTVAANLKPLRAPVVYIEGLAV-SGRN

PFQSGIS------RGI------RCSNHVPS-SGLTPVEEGGKDGSQLEEAIQGMVKDSRDGEADLNIDMN-N

VDSIKAALMAAHARIAAAENEKADALRSLEVAETRLEEYASTAVQ--VTESAVHEMQAAKESVNMELKNIME

QKLALESELVVVKKDALELALSVDKVADSILGEATTHLAEEARLKVAAAKTSAAEAAANVEERIRSAILDTT

DNLIKETRDAIEKSFAALEAAKEKAQKSEVALFQRMQILDDMVLKEASALGLQQTASELQRKLLASESEIQR

LQGEVNAVL--ARAEAAESRAAAA-YDALRQYQEAAKRSAQEHEE----RAAKALEALKAAGAARLEAARSA

FKADIEVLQTALE-TVKIAEKSQEQAYARRSQAL------------------------ERSLAAAESLAKAW

EERALAVEYLLQKSGDECVD--ASERTRGFGVVLNGGRMET-LLGNDSRKWDLLSNGPRRETPEWMERSIET

ALQGLPPRS-LTQVQD--E----------------------------------AGISLWLPS-PEEVWSIAT

AEVKE-DMYTREAAEKEAIDEQRRVLEKTLKKKAVR---KTPQ----ILESGTGSGREIVFQGFNWESWR-K

QWYLELGPKAADLSSCGITTIWFPPPTQSVSPQGYMPGDLYNLNSSYGSVEELKNSIEEMHSNELLVLGDVV

LNHRCAQFKGPNGIWNVFGGKLAWGPEAIVRDDPNFQGRGNPSSGNHLSDFFHAAPNIDHSQDFVRKDIKEW

MKWLRTEIGFDGWRLDFVRGFWGGYVKEYIEATEPAFAIGEYWDSLAYEGGNVCYNQDAHRQRIINWINATG

GTSSAFDVTTKGILHSAL-HSQYWRLIDPNGKPPGVMGWWPSRAVTFLENHDTGSTQGHWPFPRDKLMQGYA

YILTHPGTPVIFYDHFY--DFGLREPITELIAVRKRTDVHCRSTVKIYQATNQGYAAQVGDNLVMKLGHLDW

NPSK-ENNLEGKWSRCVDKDDSFSDITVRHRGNLEMLEQLYRGSECVYDDNSEIDAKLIAMAFACPPTRLLL

FPDHLPPQHRLHPSSSPVPLFFHPPPFRLCFRILSISSSSSSLHKITELDEGRPPPPPWQKFSTDAPPDDGS

NPKPRAFRPAPWQQGSRDHRPQEPLRKQGAQTKRNFDGGAVEAEADKSALATIVEKLRTIHDSLDASEPGTT

GIAFNDSASSQSNADETHSSSSVSATEDKFPWEKPSESTEQETVVQVSQRRNPTRADLLIPPDELKRLRMIS

PELQERLKIGKLGVTRSIVISLQQQWRTLELVKVRCQGPAANNIKKTLADLEENTGGLVIWRDKNAVVVYRG

VGFNPESDVGVKPEIETDISVAMGIEQNGATSIYGNHDRKNDETEMESLLDSLGPRYEKWTGLRPVPIDADL

LPPEVPNYKPPFRLLPSGVWAGLTDAELTNLRRLARPLAPHFVLGRNKGQQGLAVAMLKLWEKTEIVKIAVK

KRVQNTNNEMMAEQIRRLTGGVLLSRDKFF

>Azfi_s0015.g013850/1-998

--MKPWDIDLLLMEKVSILPKQHDTFSCLCTISKSTIIQPRIASTGIHLSSSKSFSGPLLCVETLAAVSGRH

LPPSGVS------RWI------RCSGNRFSRSGSSP----IEGGQDASQLEETIKRLVDEGSIGQNIDLN-D

VEGLKTILVAAHARIVAAESEKEDALKALAEAEAKLQEYTSTAVQ--ATEEPVKKTKHSNKSEGVDLQSIID

QKLAVESELAVAKKNAIELAVCVDKVADAIYEETTASLAEEAHLKIAEAKTSAAEAANSVEERVKSAVLDTA

NAMIRETRDAIEKSFSALEAAKEKAQKSEIALFQRMQILDDMVLKEASALGLQKTASEIQRKLLAAESEIKR

LQGEVTAVL--ARAEAAESRASAA-DDALRQFQERANQDALEHEE----RAKKALEALKLAGAARLEAARAA

FKSDIEVLQAALD-TVQIAGKSQEQAYARRYQAL------------------------ERSLSSAETLAKAW

EERALAVEALLQKSREEGAD--VAGFNVGLEGILTGGRMET-LLGNDSRKWDLLANGPRRETPEWMERRIEV

ALQGLPPRKLGQIEEE-------------------------------------IGVSLKLPS-PDEVWSIAT

AEVKE-DVYTRQAAEKEAIDEQRRVLENTLKIKTVR-------KTAQVLESGTGSGREIVFQGFNWESWR-K

KWYLELGPKAADLYSCGVTTIWFPPPTQSVSPQGYMPGDLYNLNSAYGTEEELKNCIEEMHNHELLVLGDAV

LNHRCAQFKGPNGVWNVFGGKLAWGPEAIVRDDPNFQGRGNPSSG----DFFHAAPNIDHSQDFVQRDIKEW

MKWLRTEIGFDGWRLDFVRGFWGGYVKDYIEATDPAFAIGEYWDSLAYEGGNVCYNQDAHRQRIINWINATG

GTSSAFDVTTKGILHSAL-HNQYWRLIDPSGKPPGVMGWWPSRAVTFLENHDTGSTQGHWPFPRDKLMQGYA

YILTHPGTPVIFYDHFY--DFGLRDAIAELIAVRNRTGVNCRSPVKIYQATNQGYASQIGDNLVIKMGHLDW

NPSK-ENNLEGKWNRCLDKG----------------------------------------------------

------------------------------------------------------------------------

------------------------------------------------------------------------

------------------------------------------------------------------------

------------------------------------------------------------------------

------------------------------------------------------------------------

---------ADYQ----------------------------------------------IWERS--------

------------------------------

>Aco005712/1-909

-------MSIVRLKPILHHLPPIENPRLSPRELRRSELPGSIRC------CSKPRVSVSRGLRRSDPLRSLP

IIRAGVA------PTPSLAEDDQATEVVYSETFLLKRSQAVEGKVSVRLDAA--EEDGSRWRLVIGCNLP-G

KWILHWGVTYHDER---GSEWDQPPPEMWPPESVPIKDYAIETPL--KTSSSNPEEQVLHEVQIDFDSSV--

------------QIGAIHFVLKEEETGAWFQHKGRDFRITLRDTF-KEESSLGGTQGFSIWPGALEQISTLL

K--PEGSSPMTQETLRGGREAKQWNSR-IAGLYEEFSISKEEPVQNLMTVTVRKSNDTEKKLVQFDTDIPGE

VTVHWGVCK--DDSKKWEIPPTPH-PPATKLFRSKALQTSLQPKE----NGRGSWGIF-------------P

VAQESLGLLFVLK-LDKYTWLKN--DGTDFYIPL------------------------INVSGSSTSNSQEL

GNKQMDSSQGSVSTE----------------------EAKP-VVKNIEYTHEIISE-------------IRN

LVTDISSKNGKGANTKEAQ----------------------------------ESILEEIEKLAAEAYKIFR

SSALG------------FVEE--TVSHAEPSKPAVQ------------ICSGTGSGYEILCQGFNWESHKSG

RWFLELGAKAEELASLGFTVIWLPPPTESVSPEGYMPKDLYNLNSRYGSMEELRDLVKRFHEVGIKVLGDVV

LNHRCAHYKNQNGIWNIFGGRLNWDDRAIVADDPHFHGRGNKSSG----DHFHAAPNIDHSQEFVRRDLKEW

LCWLREEIGYDGWRLDFVRGFWGGYVKDYLEATEPFFAVGEYWDSLSYTYSEMDYNQDAHRQRIIDWINATN

GTAGAFDVTTKGILHSALGRCEYWRLSDQKGKPPGVIGWWPSRAVTFIENHDTGSTQGHWRFPSGTEMQGYA

YTLTHPGTPAVFYDHIF---SHYQPEISRLISVRRRQEIHCRSKIKIIKAERDVYAAEIDEKVAVKIGPGHY

EPSN-GPK---NWVLAAE-G----------------------------------------------------

------------------------------------------------------------------------

------------------------------------------------------------------------

------------------------------------------------------------------------

------------------------------------------------------------------------

------------------------------------------------------------------------

---------KDYK----------------------------------------------VWETS--------

------------------------------

>Atrichopoda_scaffold00078.76/1-997 scaffold00078.76

-------MATLRLKPSLHHHTKWNPRSNQKLRNYSNWNPRLNHKLRNSTFSGLNCIYKRFDIRSFSKIKPGV

VVRASST--------NTSVEEAVASDVLFTETFQLKRSEKVEGKISVRVDH---QKDDDKSQVAIGCNLP-G

KWVLHWGVTYYDDV---SSEWDQPPPDMRPPDSIAIKDYAIETPL--KKSPLAVEGNSLYEVQIDIKVNH--

------------SVGALHFVLKDEETGAWYQHRGRDFRVCLLEDLQDENDKVGDKKSFSLWPGDFVKMPEVL

LTAIKREANGQEPNGDGKDARKK--AKLIEEFYDEYIFMKEKMVGNYLTVSVQENEEKNKALVLFDTDLPGN

VIIHWGVCR--DNGKKWEIPQASH-PPSTNLFRKKALQTSLQFKE----NGGGSWGLF-------------T

LDKELAGLLFVLK-LDGYTWLNN--NGSDFYIPL------------------------SAEIGTSSVRPTEK

INAPEGHKEEDISNDVKNDTWTIEESGSSQLEKSQSGANSP--VSRVSYTDEIINE-------------IRS

LVSDISSERSANMKSKDAR----------------------------------ESILQEIEKLAAEAYSIFR

SSIPT------------FLKE--LVSEPEIEKPQPK------------ICSGTGTGYEVLCQGFNWESHKSG

RWYSELYEKAADIVSLGFTVIWLPPPTESVSPEGYMPKDLYNLNSRYGTIEELKTLVRRFHEVGIKVLGDAV

LNHRCAHYKNQNGVWNIFGGRLNWDDRAIVADDPHFQGRGNKSSG----DNFHAAPNIDHSQDFVRNDLKEW

LNWLRNEIGYDGWRLDFVRGFWGGYVKDYLDATEPYFAVGEYWDSLSYTYGEMDHNQDAHRQRIIDWINATN

GTAGAFDVTTKGILHSALGKCEYWRLSDQKGKPPGVVGWWPSRAVTFIENHDTGSTQGHWRFPSGKEMQGYA

YILTHPGTPAVFYDHIF---SHYRDEISALIGLRHRKKINCRSTVEIRKAERDVYAATIDDRVTVKIGPGHY

EPPS-GSQ---NWSLIAQ-G----------------------------------------------------

------------------------------------------------------------------------

------------------------------------------------------------------------

------------------------------------------------------------------------

------------------------------------------------------------------------

------------------------------------------------------------------------

---------QDYKRMPWSNLALICSKFESPGTVKTCLKRANVLPLICLSPLVVMFPKLSSWTLTSSFLKPFI

ARNRAHVR----------------------

>Atrichopoda_scaffold00110.101/1-977

------------MALLAWPGVPSRSFSHHSILPRNTKLHNPNLCIWHHSFAFRNASNHRRKIHEREKGLDGY

QPLLRAS---------------------MGDSKDILTDTVFEGDGVSSGSGNGEVLQITREEFIATND----

------ALEEARLRQEAAEKERDRLTQDLALSEAKLQEYAATIDG--NRELAVAELEAAKSLFHDKLQDSLN

EKFALETRLVLAKQDAVELAVQVEKLAEIAFQQSTSHILEDAQMRVSAAGTSAAEAAYHIEEQLRTTTENTL

SSIVEQSNDTLGKVLMAAQQASDHAKRAMESLTDGLQVVDEMVSVHSMNVGLQSAMSELERQLTFKQNEVDR

LSSELELVQ--ARANSLEARANSL-ENTLAEVQESTKRKLLEQEE----ATKSLLKKFKEEAAKSEASATMA

LKVELEGIRSTVD-AAKKTMELKDRAYMQRCLAL------------------------ERSLKASEAATNVW

RQRAEMAESLLQEGRLVGEE------DQDATVVVNGGRLDI-LTEDDSQRWRLLADGPRRDIPEWMARRIRS

ICPKFPPRKTTIPEELTVS-----------------------------------SSSLTLPK-PEEVWSIAQ

EKPKQGDTFIKQVIEKEAIGKQRKALERALQRKTIQ---RQRIPEPTKLEPGTGTGHEIVFQGFNWESSR-R

RWYLELAPKAADLSHCGITAVWLPPPTESVAPQGYMPSDLYNLNSAYGTVDELKQCIEEFHSQDLLALGDVV

LNHRCAQKQSPNGVWNIFGGKLAWGPEAIVCDDPNFQGLGNPSSG----DIFHAAPNVDHSQEFVRRDIKEW

LNWLRSEIGFDGWRLDFVRGFSGGYVKEYIEASNPAFAIGEYWDSLAYEGGNLCYNQDAHRQRIVNWINATS

GTSSAFDVTSKGILHSAL-HNQYWRLIDPQGKPTGVMGWWPSRAVTFLENHDTGSTQGHWPFPREKLTQGYA

YILTHPGTPVIFYDHFY--DFGLRDVITELIEARSRAGIHCRSSVKIYHANNEGYVAQIGDTLLMKIGHLDW

NPSK-ENQLEGSWQKFVDKG----------------------------------------------------

------------------------------------------------------------------------

------------------------------------------------------------------------

------------------------------------------------------------------------

------------------------------------------------------------------------

------------------------------------------------------------------------

---------GDYQ----------------------------------------------LWLRP--------

------------------------------

>Macuminata/1-914 _Achr8G04140_001

-------MLLVRWKPVLHCPPQGHRRRGFAWPRGPRRSLLLRRPIRS--AAPAFLSISSSKFSQAGRARVRP

VVRAGLA------QTP-SLADVENTEILFSETLSLKRSQTVEGKITVRLDPAVAEEEVSKWRLTIGCNLE-G

KWTLHWGVSYCDDLGRQAFEWDQPPPEMRPPESVLIKDYAIETPL--KRLSSQSERQALHELQIEFDSNT--

------------PIAAIHFVLKEEETGAWFQHKGRDFRISFTDYF-EVANSVGGNQGLSIWPGGFDQISSLL

LKAEESTSKKEDPDDEDGNVVKQ--NRCIAPIYKEFPILKEEFVPNHMTVSVRSSDKTDKNIVQFDTDLPGD

VVIHWGVCK--DDGRKWVIPSTPH-PPATKIFRHKALQTLLQPKP----DGLGSWGLF-------------L

VDQGTSGVVFVLK-LNEYTWLNN--NGTDFFIPI---------------------------GSVSSTTAEIG

TSDPKNINSLPMKPQ----------------------GPEE-LIEAVAYTDEIIKE-------------IRH

LVTDISSEKGKRAKSKEAQ----------------------------------ENILQEIEKLAAEAYSIFR

ISIPG------------FVE---LASDTELLKPAVK------------LSSGTGSGYEILCQGFNWESHKSG

RWYSELSDKAKELSSLGFTVIWLPPPTESVSPEGYMPKDLYNLNSRYGSLEELKDLVNSFHEVGIKVLGDAV

LNHRCAHFQNKNGIWNVFGGRLNWDDRAIVADDPHFQGRGNKSSG----DNFHAAPNIDHSQDFVRRDLKEW

LCWLRKEVGYDGWRLDFVRGFWGGYVKDYMEATEPYFAVGEYWDSLSYTYGDMDHNQDAHRQRIVDWINATN

GTAGAFDVTTKGILHSALEKCEYWRLSDQNGKPPGVVGWWASRAVTFIENHDTGSTQGHWRFPSGKEMQGYA

YILTHPGTPAVFYDHIF---SHYQQEISRLISVRNENKIHCRSTVKIVKAERDVYAAEIDGKLAVKIGPGHY

EPPD-GPT---KWVVAAE-G----------------------------------------------------

------------------------------------------------------------------------

------------------------------------------------------------------------

------------------------------------------------------------------------

------------------------------------------------------------------------

------------------------------------------------------------------------

---------RDYK----------------------------------------------VWETS--------

------------------------------

>Spipo8G0033500/1-762

------------------------------------------------------------------------

------------------------------------------------------------------------

----------------RGGEWDQPPLDMRPPGSIPIKDYAIETPL--KKSSSASEGEILHELQIDLKPSF--

------------SISAIHFVLKDEETGAWYQYRGRDFKIPL--LIWEDEGIISGEKGFSLWPGALSQISNIL

LKTDGAATNVDDREKSEAKPE----IRLVEKYSEEFPIVQEETVQNYLTVSVQRSDELDRNVVHFDTDIPGN

VVVHWGVCR--DENRNWEIPTPPH-PAGSRVFRGKALQTLLQPKA----DGSGNWGDF-------------P

VDEDFSCLHFVLK-LSEYTWLND--VGDDFYIPL-------------------------------------I

SKKELLKEAVLSESQPTDV------------------EMTQ-DVEAVSYTDDIISE-------------IRS

LVTDISAEKGNITKSKEAQ----------------------------------ENILQEIEKLAAEAYGIFR

SSIPV------------YVEE--PVSDEELLKPPVQ------------ISSGTGTGYEILLQGFNWESHRSG

KWYSELYAKADEIASMGFTTIWLPPPTESVSPEGYMPKDLYNLNSRYGNIEELKSLVKKFHEVGIRVLGDVV

LNHRCAHAKNQNGVWNIFGGRLAWDDRAVVSDDPHFQGRGNKSSG----DNFHAAPNIDHSQEFVRKDLKEW

LLWLREEIGYDGWRLDFVRGFWGGYVKDYLEASEPYFAVGEYWDSLSYTYGQMDYSQDAHRQRIIDWINATG

GNASAFDVTTKGILHAALEKCEYWRMSDEKGKPPGVMGWWPSRAVTFIENHDTGSTQGHWRFPSGKEMQGYA

YILTHSGTPTVFYDHIV---SHYQREVAALISLRNRNKIHCRSTVKIKKAERDVYAAEIDERVVMKIGPGYY

EPE--GPR---SWSVAAEGG----------------------------------------------------

------------------------------------------------------------------------

------------------------------------------------------------------------

------------------------------------------------------------------------

------------------------------------------------------------------------

------------------------------------------------------------------------

----------EYK----------------------------------------------VWEAI--------

------------------------------

>Os01g51754/1-515

------------------------------------------------------------------------

------------------------------------------------------------------------

------------------------------------------------------------------------

------------------------------------------------------------------------

------------------------------------------------------------------------

--------------MTWEIPPEPH-PPATKIFRQKALQTMLQQKA----DGTGNSLSF-------------L

LDGEYSGLIFVVK-LDEYTWLRNVENGFDFYIPL------------------------TRADAEADKQK---

------------------------------------------ADDKSSQDDGLISD-------------IRN

LVVGLSSRRGQRAKNKVLQ----------------------------------EDILQEIERLAAEAYSIFR

SPTID------------TVEESVYIDDSSIVKPA---------------CSGTGSGFEILCQGFNWESHKSG

KWYVELGSKAKELSSMGFTIVWSPPPTDSVSPEGYMPRDLYNLNSRYGTMEELKEAVKRFHEAGMKVLGDAV

LNHRCAQFQNQNGVWNIFGGRLNWDDRAVVADDPHFQGRGNKSSG----DNFHAAPNIDHSQEFVRSDLKEW

LCWMRKEVGYDGWRLDFVRGFWGGYVHDYLEASEPYFAVGEYWDSLSYTYGEMDYNQDAHRQRIVDWINATN

GTAGAFDVTTKGILHSALERSEYWRLSDEKGKPPGVLGWWPSRAVTFIENHDTGSTQGHWRFPFGMELQGYV

YILTHPGTPAIFYDHIF---SHLQPEIAKLISIRNRQKIHCRS-----------------------------

------------------------------------------------------------------------

------------------------------------------------------------------------

------------------------------------------------------------------------

------------------------------------------------------------------------

------------------------------------------------------------------------

------------------------------------------------------------------------

------------K----------------------------------------------VWEVSS-------

------------------------------

>Zosma95g00270/1-899

-----------MLSLIRFGHPVVRPRIHEHTLFPSFSQWRLSGRLRLSVPTTRRRSGAVSCFFRPWRNSELA

NMEEGGK------TLEEGQNLIRSAVVVMEEKFEVQRTQMVEGKLTVRLERS--EDGVNKGRFVVGCDIE-G

NWVLHWGVTYFDQ-LEFGSEWEQPPNEIRPPGSIPIKDYAVETPL--KKSSSNIMGETFHEVKIDFNFDS--

------------SIAAIHFVLKEEVSGAQYQHKGRDFKIPLIDNVQEDD--IGIDGSKDPFNSSGLKVDGIR

INAIENSSSCKDVNYKNG---------LLEGYHKEYSICKEEQVTNSVTVAIMRNEGENTNHLLFDTDIPGE

VIVHWGVCK--GDDKSWHIPKTPH-PPKSRVFRKKAVQTLLQKKS----EGVGSWGLF-------------P

MEKDISGVPFVLK-LNKDTWLDN--LGIDFYVPL------------------------------TGDMSLEY

KTKDQIEAELISTDV----------------------KEAQ-EIESSTNTDEIITE-------------IRN

LVTDISAEKSLTTTTKEAQ----------------------------------ESILEEIEKLAAEAYSVFR

SITPI------------YVEE--PISDATAIKPPIG------------KCPGTGSGYEVFCQVFNWESCKSK

NWYSEISSKAGELSKLGITIVWLPPPTESVSAQGYMPSDLYNLNSSYGSMEDLKNAINTFHSFGIKVVGDVV

LNHRCAQHQNKNGIWNIFGGRLNWDDRAIVADDPHYQGRGNKSSG----ENFHAAPNIDHSQEFVRRDLKEW

LCWLRKEIGFDGWRLDFARGFWGGYMKDYMEASEPYFSVGEFWDSLSYTYGEMDHNQDAHRQRCIDWINATN

GTSAAFDVTLKGILHTTLEKFEYWRLSDERGKPPGVAGWWPSRAVTFIENHDTGSTQGHWRFPAGKEMQGYT

YILTHAGTPTVFYDHLC---SHYNQEIGKLIALRHRKKIHCRSKVKITKAEKEVYAAIIDDKVAMKIGPGHY

EPGD-----HNKWKVAAE-G----------------------------------------------------

------------------------------------------------------------------------

------------------------------------------------------------------------

------------------------------------------------------------------------

------------------------------------------------------------------------

------------------------------------------------------------------------

---------RDYK----------------------------------------------VWETS--------

------------------------------

>Bradi2g48150/1-868

------------------MSAASWSIPAIPRAAPPARGGLPGDAFLVAARPGPGRRRAAPGRRLRLRGGGVV

VARAGAA--------EVPVTHPEESGVVFSEKFPLRRCKTVQGKAWARVVAE--PDGEGMCKIVIGCDVE-G

KWVLHWGVSYDGEQ---GREWDQPPSEVRPPGSVTIKDYAIETPL---VGSPNSEGHMVHEVEIKFNQDT--

------------PIAIINFVLKEEETGAWFQHKGGDFRIPLSGSLEDGDPFGAQQDTVHPGAKPEGSSAQPQ

ETVPGDKGPSVKR---------------ISEFYGEYPILKSEYVQNFVSVTVTENSETDKSLVEFDTDITGQ

VIIHWGVCK--DNTMTWEIPSEPH-PPKTKIFRQKALQTLLQQKT----DGTGNTISF-------------L

LNADYSGLVFVLK-LDEYTWLRNVDNGFDFYIPL------------------------KEPHKSDEQK----

------------------------------------------VDDKSAQTDGLIGD-------------IRN

LVVGLSSRRGQRAKNKVLQ----------------------------------EDILQEIERLAAEAYSIFR

SPTID------------AVEDSVYIDDPATVKPA---------------CSGTGSGFEILCQGFNWESHKSG

KWYVELGAKAKELASLGFTIVWSPPPTDSVSPEGYMPRDLYNLNSRYGTIEELKQLVNIFHEAGVKVLGDAV

LNHRCAQFQNQNGVWNIFGGRINWDDRAVVADDPHFQGRGNKSSG----DNFHAAPNIDHSQDFVRNDLKEW

LCWMRKEVGYDGWRLDFVRGFWGGYVKDYLEASEPYFAVGEYWDSLSYTYGEMDYNQDAHRQRIVDWINATS

GTAGAFDVTTKGILHMALERSEYWRLSDEKGKPPGVLGWWPSRAVTFIENHDTGSTQGHWRFPYGMEMQGYV

YILTHPGTPAVFYDHVF---SHLQQDIAKLISVRRRLKIHCRSKIKILKAEQNLYAAEIDEKVTMKIGSGHF

EPT--GPI---NWIVAVE-G----------------------------------------------------

------------------------------------------------------------------------

------------------------------------------------------------------------

------------------------------------------------------------------------

------------------------------------------------------------------------

------------------------------------------------------------------------

---------QDYK----------------------------------------------IWEASS-------

------------------------------

>AT1G69830/1-887

-------MSTVPIESLLHHS---YLRHNSKVNRGNRSFIPISLNLRSHFTSNKLLHSIGKSVGVSSMNKSPV

AIRATSS------DTA-VVETAQSDDVIFKEIFPVQRIEKAEGKIYVRLK----EVKEKNWELSVGCSIP-G

KWILHWGVSYV---GDTGSEWDQPPEDMRPPGSIAIKDYAIETPL--KK---LSEGDSFFEVAINLNLES--

------------SVAALNFVLKDEETGAWYQHKGRDFKVPLVDDVPDNGNLIGAKKGFGALGQL--------

------SNIPLKQDKSSAETDSIEERKGLQEFYEEMPISKRVADDNSVSVTARKCPETSKNIVSIETDLPGD

VTVHWGVCK--NGTKKWEIPSEPY-PEETSLFKNKALRTRLQRKD----DGNGSFGLF-------------S

LDGKLEGLCFVLK-LNENTWLNY--RGEDFYVPF------------------------LTSSSSPVETEAAQ

VSKPKR-------------------------------KTDK-EVSASGFTKEIITE-------------IRN

LAIDISSHKNQKTNVKEVQ----------------------------------ENILQEIEKLAAEAYSIFR

STTPA------------FSEE--GVLEAEADKPDIK------------ISSGTGSGFEILCQGFNWESNKSG

RWYLELQEKADELASLGFTVLWLPPPTESVSPEGYMPKDLYNLNSRYGTIDELKDTVKKFHKVGIKVLGDAV

LNHRCAHFKNQNGVWNLFGGRLNWDDRAVVADDPHFQGRGNKSSG----DNFHAAPNIDHSQDFVRKDIKEW

LCWMMEEVGYDGWRLDFVRGFWGGYVKDYMDASKPYFAVGEYWDSLSYTYGEMDYNQDAHRQRIVDWINATS

GAAGAFDVTTKGILHTALQKCEYWRLSDPKGKPPGVVGWWPSRAVTFIENHDTGSTQGHWRFPEGKEMQGYA

YILTHPGTPAVFFDHIF---SDYHSEIAALLSLRNRQKLHCRSEVNIDKSERDVYAAIIDEKVAMKIGPGHY

EPPN-GSQ---NWSVAVE-G----------------------------------------------------

------------------------------------------------------------------------

------------------------------------------------------------------------

------------------------------------------------------------------------

------------------------------------------------------------------------

------------------------------------------------------------------------

---------RDYK----------------------------------------------VWETS--------

------------------------------
