# Supplementary material for: Plasticity in plastid redox networks: evolution of glutathione-dependent redox cascades and glutathionylation sites
Source: BMC Plant Biol. 2021 Jul 5;21:322. doi: 10.1186/s12870-021-03087-2 (PMC8256493; doi:10.1186/s12870-021-03087-2)
Supplement: Supplementary file 12 — Additional file 12. Word-file containing all alignments used to build phylogenetic trees in FASTA format. [file 12870_2021_3087_MOESM12_ESM.docx]

**Additional File 12**

**Protein sequence alignments used to build phylogenetic trees in fasta format**

Alignments were generated in Jalview 2.11.1.0 (using the Muscle algorithm) and manually curated.

Alignments were exported to word using Mview (EMBL-EBI search and sequence analysis tools APIs) <https://www.ebi.ac.uk/Tools/msa/mview/>

A multi-fasta format is additionally provided for each alignment.

**DHAR**

cov pid  **1** **[ . . . . : . . .** **80**

1 139875_Selmo 100.0% 100.0% **MASQDVDLEVFGKAATGTSNQRGDCPFSQRVYMVLEEKHLPYKATYVEEGPNKPDWFMQHNPSGLMPVLRD-AADWIQDS**

2 233824_Selmo 85.3% 68.6% **--------------------------------MELEEKRLPYKATYIQEGPDKPAWFMEKNPSGLMPVLRD-GSEWIQDS**

3 271409_Selmo 99.5% 42.4% **AVATTEAVEVLVKAANGDPSRLGDCPFSQRVLLTLEEKGIPYNSKFVDME-NKPAWFLEANPEGKVPVIKD-DGKWVADS**

4 AT1G19570_AtDHAR1 97.2% 44.3% **-----MALEICVKAAVGAPDHLGDCPFSQRALLTLEEKSLTYKIHLINLS-DKPQWFLDISPQGKVPVLKI-DDKWVTDS**

5 AT1G75270_AtDHAR2 97.2% 45.3% **-----MALDICVKVAVGAPDVLGDCPFSQRVLLTLEEKKLPYKTHLINVS-DKPQWFLDISPEGKVPVVKL-DGKWVADS**

6 AT5G16710_AtDHAR3 99.5% 40.6% **MATAASPLEICVKASITTPNKLGDCPFCQKVLLTMEEKNVPYDMKMVDLS-NKPEWFLKISPEGKVPVVKF-DEKWVPDS**

7 AaBonn_Sc2ySwM_117_162_1 99.5% 41.9% **MAAVAEPLEVLVKAASGQPDQLGDCPFSQRVLLTLEEKGIPYNARYVDTE-NKPEWFLEANPEGKVPVIKH-EGSWVADS**

8 Azfi_s0061g034873 98.2% 44.4% **---MAEPVEVLVKAAVGEPDKIGDCPFSQRVLLTLEEKNIPYTAKYVDLT-NKPEWFLELNPEGKVPVIKH-EGKWVPDS**

9 Azfi_s0081g038618 99.5% 47.5% **LANADDSVEVLVKAAVGEPEKIGDCPFSQRVLLTLEEKGIPYNAKYVDLT-NKPDWFLEINPEGKVPVIKH-EGKWVPDS**

10 Azfi_s0390g067700 100.0% 48.8% **NVRELPALQAFAVAFPGSSESKGPCPFSQKVLIALEERRLPYVATYIDESIERPKWFLDKNSSGLLPVLRD-GETWIQDS**

11 Bradi1g45010 99.5% 40.1% **ARASAEPLEVCAKASITVPDRLGDCPFTQRVLLTIEEKHLAYDLKLVDLA-NKPDWFLKINPEGKVPIVKL-EEKWVADS**

12 Bradi2g37480 97.2% 46.0% **-----MTVEVCVKAAVGHPDTLGDCPFSQRVLLTLEEKKVPYEMKLIDVS-NKPEWFLKINPEGKVPVFNSGDGKWIADS**

13 G22068_CHBRA263g00260 81.1% 35.5% **ATVDGNTLDVAIKAATGRPDLVGDCPFSQRVTMTLEEKGIKYNPILVDTS-NKPEWFLEANPSGKVPVIKY-QDKWIADS**

14 G52001_CHBRA74g00660_Outgroup 98.2% 25.8% **MTSGESTVEREGGEGEGEVVLYSSCPYAQRARISLEEKRIAYRQVEVDLS-NKPPELLAANPRGLVPAIIH-NGKSLFES**

15 Mapoly0082s0046 99.5% 43.3% **VKSGVDSMELLAKAAVGKPGQLGDCPFSQRVMLTLEEKHIKYDATYIDVS-NKPQWFLEANPEGKVPVIKH-EGKWIADS**

16 Pp3c15_21480V3_PpDHAR2 100.0% 62.2% **AANAGLDLEAFGKAKSGSSKERGDCPFSQRIYIELEEKKLPYTATYIEEGENKPDWFMEKNPKGLMPVLRD-GDEWIQDS**

17 Pp3c20_23200V3_PpDHAR3 99.5% 35.9% **MASRCQRLKVYVKAATGNPCKLGDCPFSQRVLITCELKNIAYDVKFVDLD-RKPEWFLRINPEGRVPVIKI-NGDYIPDS**

18 Pp3c22_5470V3_PpDHAR1 99.5% 45.6% **AAAEAAPTEVFVKAAVGHPDKFGDCPFSHRVVLTLAEKKVPYDMKLIDVS-NKPQWFLDINPEGKVPVIKD-EGKFVADS**

19 Sacu_s0041g012552 99.1% 44.2% **MAVSQEPVEILVKSAVGDAEKIGDCPFSQRVLLTLEEKGLPYNAKFVDLN-NKPDWFLDINPEGKVPIVKF-DGKWVPDS**

20 Sacu_s0074g017273 99.5% 45.2% **MADVSEPVEILVKAAVGEPDKIGDCPFSQRVLLTLGEKGIPYTAKYVDLT-NKPDWFLELNPDGKVPVIKH-EGKWVPDS**

cov pid  **81**  **. 1 . . . . : .** **160**

1 139875_Selmo 100.0% 100.0% **DKIFEHVENKFKEPSLKTPDEFKSVGAGIFPAFTNWLKSKDRNAPAKQEFINELTALEEHLKKHGPYIAGKNPTDSDFAL**

2 233824_Selmo 85.3% 68.6% **ERIFEHLEAKFPNPALKTPDEFKDVGSGIFPRFVEWLKSKDQAHPAKQDLIKELLSFNQHLQKHGPYIAGEKPTDSDFTV**

3 271409_Selmo 99.5% 42.4% **DVITQLIDTKFPSPSLVTPPEKSSVGSKIFSSFVKFLKSKDPSDGSEAALLEELKALDEYLAKNGPFVNGSNISAVDLSL**

4 AT1G19570_AtDHAR1 97.2% 44.3% **DVIVGILEEKYPDPPLKTPAEFASVGSNIFGTFGTFLKSKDSNDGSEHALLVELEALENHLKSHGPFIAGERVSAVDLSL**

5 AT1G75270_AtDHAR2 97.2% 45.3% **DVIVGLLEEKYPEPSLKTPPEFASVGSKIFGAFVTFLKSKDANDGSEKALVDELEALENHLKTHGPFVAGEKITAVDLSL**

6 AT5G16710_AtDHAR3 99.5% 40.6% **DVITQALEEKYPEPPLATPPEKASVGSKIFSTFVGFLKSKDSGDGTEQVLLDELTTFNDYIKDNGPFINGEKISAADLSL**

7 AaBonn_Sc2ySwM_117_162_1 99.5% 41.9% **DVITALLEEKYPEPSLAVSEDKAAVGSKLFGAFVRFLKSKDPADGTEAALVDELKALNDFLKENGPYVGGDKISAVDLSL**

8 Azfi_s0061g034873 98.2% 44.4% **DVITQILEEKHPEPSLQIPAEKASVGSKIFPSFVKFLKSKDNTDGSEEALVAELATLDEYLKENGPFINGEKISAPDLGL**

9 Azfi_s0081g038618 99.5% 47.5% **DVITGIIEEKFPEPSLKTPEEKASVGSKIFPSFVKFLKSKDPSDGSEEALVSELIAFNDYLKDNGPFVHGGEITASDLSL**

10 Azfi_s0390g067700 100.0% 48.8% **EAIIDHLCSKYVEEPFKTPQNIKEMIQKQFGSLNDWLKSKNKDDPAGKAYEHALQEVNEHLKKNGPYMAGDKPTDIDFDF**

11 Bradi1g45010 99.5% 40.1% **DVITQALEEKYPQPSLAIPPERASTGSKIFSTFIGFLKSKDPNDGTEQAILSELTSFNSYLEDNGPFINGGTVSAADLSL**

12 Bradi2g37480 97.2% 46.0% **DVITQIIEEKYPTPSLVTPPEYSSVGSKIFSTFIAFLKSKDASDGTEKALLDELQALEEHLKAHGPYINGENVSAADLSL**

13 G22068_CHBRA263g00260 81.1% 35.5% **DVIVGILEKDFPEPSLAVPDDKVV----------------------------------------GPFVNGDKISSADMSL**

14 G52001_CHBRA74g00660_Outgroup 98.2% 25.8% **MVVAEYLDEAFPRGSIGVPYDRAIVGKKITATFLKVLQTQDP-EGQEKAHIKELSSAMEGISPDGPFFMGEEFGIVDIAL**

15 Mapoly0082s0046 99.5% 43.3% **DVITALLEDLYPEPSLKVPDEKKSVGSSIFGAFIGHLKSKDSSDGTEAALLKVLVEFNEYLQQSGPFVNGEKICSVDLAL**

16 Pp3c15_21480V3_PpDHAR2 100.0% 62.2% **DKIAEHLEKKYPEVSLATPKEYKQIGLNIFQAFTTYLKSKNADDQSKQELLKELAALDQHLQTKGPYIAGENPTDSDYAL**

17 Pp3c20_23200V3_PpDHAR3 99.5% 35.9% **DIIVDVLEKSYPYPPLSTCRNITCRGQNIFPAGMAFFKSKNPRDGTESQFVCELDHMNHHLCNEGPYIAGQYVTSADIAL**

18 Pp3c22_5470V3_PpDHAR1 99.5% 45.6% **DVITQLLEEKYPEPCLKTPEDKASAGARIFPNFAAFLKSKDPNDGTEAALLAELKSLDEHLKSNKPFIAGEAVTAADLAL**

19 Sacu_s0041g012552 99.1% 44.2% **DVITGILEEKFPEPSLKTPEDKKSIGSKIFPSFVKFLKSKEP-DGSEEVLVAELTTFNDYLKDNGPFINGEKITAADLSL**

20 Sacu_s0074g017273 99.5% 45.2% **DVITQILEEKYPEPSLKLPSEKASVGSKIFPSFVKFLKSKDSSDGSEEALVAELTALNDYLKDNGPFINGDKISAADLSL**

cov pid **161**  **. . . 2 . ]** **218**

1 139875_Selmo 100.0% 100.0% **APKLRHARVALKHFIDFVFPSNLQHVAKYIELMETRPSFKKTDSPDEMIIAGWQTKFD**

2 233824_Selmo 85.3% 68.6% **APKLRHARVALGQIMGFAFPEKLEALHKYIELMEARPSFIHTDSPDEMIICGWRKKFS**

3 271409_Selmo 99.5% 42.4% **APKLYHLKIALGHYKQWSVPENLTNLNSYMEALFKRESFQKTMAPAEVVVKGWAKHLS**

4 AT1G19570_AtDHAR1 97.2% 44.3% **APKLYHLQVALGHFKSWSVPESFPHVHNYMKTLFSLDSFEKTKTEEKYVISGWAPKVN**

5 AT1G75270_AtDHAR2 97.2% 45.3% **APKLYHLEVALGHYKNWSVPESLTSVRNYAKALFSRESFENTKAKKEIVVAGWESKVN**

6 AT5G16710_AtDHAR3 99.5% 40.6% **APKLYHMKIALGHYKNWSVPDSLPFVKSYMENVFSRESFTNTRAETEDVIAGWRPKVM**

7 AaBonn_Sc2ySwM_117_162_1 99.5% 41.9% **APKLYHLKITLDHFKKWAIPGDLTYLLDYIKDLHSRESFEKTAAPAPVVVKGWSKHLG**

8 Azfi_s0061g034873 98.2% 44.4% **APKLFHLEIALGQYKKWSIPGNLTYLKAYIEALHARESFLKTKPAHEHVIAGWQKHVS**

9 Azfi_s0081g038618 99.5% 47.5% **APKLFHLEVALGHYKKWALPSNLTYLKSYIEALHSRESFVKTKPASEHVVAGWQKHVS**

10 Azfi_s0390g067700 100.0% 48.8% **APKLRHARTLLLHFMDFIIPTELTSVLQYIDLTENRPSFKRTDSPDNDIIFSWQKKLS**

11 Bradi1g45010 99.5% 40.1% **GPKLYHMEIALGHYKTWSVPDALAHVTTYMKTIFSRDSFVKTRALPEDVIAGWRPKVM**

12 Bradi2g37480 97.2% 46.0% **GPKLFHLQVSLEHFKGWKIPETLTGVHAYTEALFSRESFAKTKPAKEHLIAGWAPKVN**

13 G22068_CHBRA263g00260 81.1% 35.5% **VPKLYHLRIALKHYRGWSIPEDLKNVHAYIKATESRDSFQKTAAAEEYVIKGWEKHVV**

14 G52001_CHBRA74g00660_Outgroup 98.2% 25.8% **VP--FAMRHVLKHFRELSVPDEFSRFRVWFAAAVNWPSVKATSFDEKDILPILSKYAN**

15 Mapoly0082s0046 99.5% 43.3% **APKLFHMQVALGHYKQWTVPKEVPEVSSYMNALFSRESFLKTKAKEEDVIKGWEAKVL**

16 Pp3c15_21480V3_PpDHAR2 100.0% 62.2% **IPKLHHMRVSLAHYMGFKIPSEHKALHKYIKLLESRPSFQKTNSPDDMIIEGWQKKFG**

17 Pp3c20_23200V3_PpDHAR3 99.5% 35.9% **APQLYVLQTALAYYKNWTNFEQFPALNLFMKNMYALPAFMQTAPAPEVVIQGWAKHWS**

18 Pp3c22_5470V3_PpDHAR1 99.5% 45.6% **APKLHHLTVALGHYKKWSIPEDLTNVLSYVEAVHSLESFKKTKPADEFIIAGWAKFFV**

19 Sacu_s0041g012552 99.1% 44.2% **SPKLFHMEVALGHYKKWKIPDNLSYLKDYIQALHSRESFLKTKPHQEAVIAGWQKHVL**

20 Sacu_s0074g017273 99.5% 45.2% **APKLFHLKIALGHYKKWSIPENLTYLNSYIETLHARESFVKTKAAHEHVVAGWQKHVS**

>139875_Selmo

MASQDVDLEVFGKAATGTSNQRGDCPFSQRVYMVLEEKHLPYKATYVEEGPNKPDWFMQHNPSGLMPVLRD-

AADWIQDSDKIFEHVENKFKEPSLKTPDEFKSVGAGIFPAFTNWLKSKDRNAPAKQEFINELTALEEHLKKH

GPYIAGKNPTDSDFALAPKLRHARVALKHFIDFVFPSNLQHVAKYIELMETRPSFKKTDSPDEMIIAGWQTK

FD

>233824_Selmo

--------------------------------MELEEKRLPYKATYIQEGPDKPAWFMEKNPSGLMPVLRD-

GSEWIQDSERIFEHLEAKFPNPALKTPDEFKDVGSGIFPRFVEWLKSKDQAHPAKQDLIKELLSFNQHLQKH

GPYIAGEKPTDSDFTVAPKLRHARVALGQIMGFAFPEKLEALHKYIELMEARPSFIHTDSPDEMIICGWRKK

FS

>271409_Selmo

AVATTEAVEVLVKAANGDPSRLGDCPFSQRVLLTLEEKGIPYNSKFVDME-NKPAWFLEANPEGKVPVIKD-

DGKWVADSDVITQLIDTKFPSPSLVTPPEKSSVGSKIFSSFVKFLKSKDPSDGSEAALLEELKALDEYLAKN

GPFVNGSNISAVDLSLAPKLYHLKIALGHYKQWSVPENLTNLNSYMEALFKRESFQKTMAPAEVVVKGWAKH

LS

>AT1G19570_AtDHAR1

-----MALEICVKAAVGAPDHLGDCPFSQRALLTLEEKSLTYKIHLINLS-DKPQWFLDISPQGKVPVLKI-

DDKWVTDSDVIVGILEEKYPDPPLKTPAEFASVGSNIFGTFGTFLKSKDSNDGSEHALLVELEALENHLKSH

GPFIAGERVSAVDLSLAPKLYHLQVALGHFKSWSVPESFPHVHNYMKTLFSLDSFEKTKTEEKYVISGWAPK

VN

>AT1G75270_AtDHAR2

-----MALDICVKVAVGAPDVLGDCPFSQRVLLTLEEKKLPYKTHLINVS-DKPQWFLDISPEGKVPVVKL-

DGKWVADSDVIVGLLEEKYPEPSLKTPPEFASVGSKIFGAFVTFLKSKDANDGSEKALVDELEALENHLKTH

GPFVAGEKITAVDLSLAPKLYHLEVALGHYKNWSVPESLTSVRNYAKALFSRESFENTKAKKEIVVAGWESK

VN

>AT5G16710_AtDHAR3

MATAASPLEICVKASITTPNKLGDCPFCQKVLLTMEEKNVPYDMKMVDLS-NKPEWFLKISPEGKVPVVKF-

DEKWVPDSDVITQALEEKYPEPPLATPPEKASVGSKIFSTFVGFLKSKDSGDGTEQVLLDELTTFNDYIKDN

GPFINGEKISAADLSLAPKLYHMKIALGHYKNWSVPDSLPFVKSYMENVFSRESFTNTRAETEDVIAGWRPK

VM

>AaBonn_Sc2ySwM_117_162_1

MAAVAEPLEVLVKAASGQPDQLGDCPFSQRVLLTLEEKGIPYNARYVDTE-NKPEWFLEANPEGKVPVIKH-

EGSWVADSDVITALLEEKYPEPSLAVSEDKAAVGSKLFGAFVRFLKSKDPADGTEAALVDELKALNDFLKEN

GPYVGGDKISAVDLSLAPKLYHLKITLDHFKKWAIPGDLTYLLDYIKDLHSRESFEKTAAPAPVVVKGWSKH

LG

>Azfi_s0061g034873

---MAEPVEVLVKAAVGEPDKIGDCPFSQRVLLTLEEKNIPYTAKYVDLT-NKPEWFLELNPEGKVPVIKH-

EGKWVPDSDVITQILEEKHPEPSLQIPAEKASVGSKIFPSFVKFLKSKDNTDGSEEALVAELATLDEYLKEN

GPFINGEKISAPDLGLAPKLFHLEIALGQYKKWSIPGNLTYLKAYIEALHARESFLKTKPAHEHVIAGWQKH

VS

>Azfi_s0081g038618

LANADDSVEVLVKAAVGEPEKIGDCPFSQRVLLTLEEKGIPYNAKYVDLT-NKPDWFLEINPEGKVPVIKH-

EGKWVPDSDVITGIIEEKFPEPSLKTPEEKASVGSKIFPSFVKFLKSKDPSDGSEEALVSELIAFNDYLKDN

GPFVHGGEITASDLSLAPKLFHLEVALGHYKKWALPSNLTYLKSYIEALHSRESFVKTKPASEHVVAGWQKH

VS

>Azfi_s0390g067700

NVRELPALQAFAVAFPGSSESKGPCPFSQKVLIALEERRLPYVATYIDESIERPKWFLDKNSSGLLPVLRD-

GETWIQDSEAIIDHLCSKYVEEPFKTPQNIKEMIQKQFGSLNDWLKSKNKDDPAGKAYEHALQEVNEHLKKN

GPYMAGDKPTDIDFDFAPKLRHARTLLLHFMDFIIPTELTSVLQYIDLTENRPSFKRTDSPDNDIIFSWQKK

LS

>Bradi1g45010

ARASAEPLEVCAKASITVPDRLGDCPFTQRVLLTIEEKHLAYDLKLVDLA-NKPDWFLKINPEGKVPIVKL-

EEKWVADSDVITQALEEKYPQPSLAIPPERASTGSKIFSTFIGFLKSKDPNDGTEQAILSELTSFNSYLEDN

GPFINGGTVSAADLSLGPKLYHMEIALGHYKTWSVPDALAHVTTYMKTIFSRDSFVKTRALPEDVIAGWRPK

VM

>Bradi2g37480

-----MTVEVCVKAAVGHPDTLGDCPFSQRVLLTLEEKKVPYEMKLIDVS-NKPEWFLKINPEGKVPVFNSG

DGKWIADSDVITQIIEEKYPTPSLVTPPEYSSVGSKIFSTFIAFLKSKDASDGTEKALLDELQALEEHLKAH

GPYINGENVSAADLSLGPKLFHLQVSLEHFKGWKIPETLTGVHAYTEALFSRESFAKTKPAKEHLIAGWAPK

VN

>G22068_CHBRA263g00260

ATVDGNTLDVAIKAATGRPDLVGDCPFSQRVTMTLEEKGIKYNPILVDTS-NKPEWFLEANPSGKVPVIKY-

QDKWIADSDVIVGILEKDFPEPSLAVPDDKVV----------------------------------------

GPFVNGDKISSADMSLVPKLYHLRIALKHYRGWSIPEDLKNVHAYIKATESRDSFQKTAAAEEYVIKGWEKH

VV

>G52001_CHBRA74g00660_Outgroup

MTSGESTVEREGGEGEGEVVLYSSCPYAQRARISLEEKRIAYRQVEVDLS-NKPPELLAANPRGLVPAIIH-

NGKSLFESMVVAEYLDEAFPRGSIGVPYDRAIVGKKITATFLKVLQTQDP-EGQEKAHIKELSSAMEGISPD

GPFFMGEEFGIVDIALVP--FAMRHVLKHFRELSVPDEFSRFRVWFAAAVNWPSVKATSFDEKDILPILSKY

AN

>Mapoly0082s0046

VKSGVDSMELLAKAAVGKPGQLGDCPFSQRVMLTLEEKHIKYDATYIDVS-NKPQWFLEANPEGKVPVIKH-

EGKWIADSDVITALLEDLYPEPSLKVPDEKKSVGSSIFGAFIGHLKSKDSSDGTEAALLKVLVEFNEYLQQS

GPFVNGEKICSVDLALAPKLFHMQVALGHYKQWTVPKEVPEVSSYMNALFSRESFLKTKAKEEDVIKGWEAK

VL

>Pp3c15_21480V3_PpDHAR2

AANAGLDLEAFGKAKSGSSKERGDCPFSQRIYIELEEKKLPYTATYIEEGENKPDWFMEKNPKGLMPVLRD-

GDEWIQDSDKIAEHLEKKYPEVSLATPKEYKQIGLNIFQAFTTYLKSKNADDQSKQELLKELAALDQHLQTK

GPYIAGENPTDSDYALIPKLHHMRVSLAHYMGFKIPSEHKALHKYIKLLESRPSFQKTNSPDDMIIEGWQKK

FG

>Pp3c20_23200V3_PpDHAR3

MASRCQRLKVYVKAATGNPCKLGDCPFSQRVLITCELKNIAYDVKFVDLD-RKPEWFLRINPEGRVPVIKI-

NGDYIPDSDIIVDVLEKSYPYPPLSTCRNITCRGQNIFPAGMAFFKSKNPRDGTESQFVCELDHMNHHLCNE

GPYIAGQYVTSADIALAPQLYVLQTALAYYKNWTNFEQFPALNLFMKNMYALPAFMQTAPAPEVVIQGWAKH

WS

>Pp3c22_5470V3_PpDHAR1

AAAEAAPTEVFVKAAVGHPDKFGDCPFSHRVVLTLAEKKVPYDMKLIDVS-NKPQWFLDINPEGKVPVIKD-

EGKFVADSDVITQLLEEKYPEPCLKTPEDKASAGARIFPNFAAFLKSKDPNDGTEAALLAELKSLDEHLKSN

KPFIAGEAVTAADLALAPKLHHLTVALGHYKKWSIPEDLTNVLSYVEAVHSLESFKKTKPADEFIIAGWAKF

FV

>Sacu_s0041g012552

MAVSQEPVEILVKSAVGDAEKIGDCPFSQRVLLTLEEKGLPYNAKFVDLN-NKPDWFLDINPEGKVPIVKF-

DGKWVPDSDVITGILEEKFPEPSLKTPEDKKSIGSKIFPSFVKFLKSKEP-DGSEEVLVAELTTFNDYLKDN

GPFINGEKITAADLSLSPKLFHMEVALGHYKKWKIPDNLSYLKDYIQALHSRESFLKTKPHQEAVIAGWQKH

VL

>Sacu_s0074g017273

MADVSEPVEILVKAAVGEPDKIGDCPFSQRVLLTLGEKGIPYTAKYVDLT-NKPDWFLELNPDGKVPVIKH-

EGKWVPDSDVITQILEEKYPEPSLKLPSEKASVGSKIFPSFVKFLKSKDSSDGSEEALVAELTALNDYLKDN

GPFINGDKISAADLSLAPKLFHLKIALGHYKKWSIPENLTYLNSYIETLHARESFVKTKAAHEHVVAGWQKH

VS

**GSTI and GSTL**

cov pid  **1** **[ . . . . : . . .** **80**

1 AT1G19570_DHAR1_outgroup 100.0% 100.0% **EICVKAAVGAPDHLGD---------CPFSQRALLTLEEKSLTYKIHLIN---LSDKPQWFLD-ISPQGKVPVLKIDDKWV**

2 G22068_CHBRA263g00260_DHAR_outgroup 83.7% 44.0% **DVAIKAATGRPDLVGD---------CPFSQRVTMTLEEKGIKYNPILVD---TSNKPEWFLE-ANPSGKVPVIKYQDKWI**

3 AT5G02780_GSTL1 99.0% 22.8% **QVPLDATSDPPALFDGTTRLYISYTCPFAQRVWITRNLKGLQDEIKLVPID-LPNRPAWLKEKVNPANKVPALEHNGKIT**

4 AT3G55040_GSTL2 100.0% 18.5% **VPELDSSSEPVQVFDGSTRLYISYTCPFAQRAWIARNYKGLQNKIELVPID-LKNRPAWYKEKVYSANKVPALEHNNRVL**

5 AT5G02790_GSTL3 99.5% 23.7% **PAPLDATSDPPSLFDGTTRLYTSYVCPFAQRVWITRNFKGLQEKIKLVPLD-LGNRPAWYKEKVYPENKVPALEHNGKII**

6 Pp3c10_15380 99.5% 20.4% **REVLDSKSASPAIFDGTTRLYFSSRCPYAQRVWVAVKYKGL-DEIECVEIS-LSDKPTWYKEKVYPVGKVPALEHNGTVT**

7 Azfi_s0042g026899 99.5% 24.5% **IPVYDSSSSPPNLFDGTTRLYINVTCPYAQRTWIARNFKGL-NQIELLGIN-LSDKPKWYLEKVYPAGKVPSLEHNGKVT**

8 Azfi_s0121g046868 75.5% 15.6% **LLPLNSKSPPPPLFNGTPRLYISLTCPYSQRVWIARNFK------------------------------VPALEHNGRVK**

9 Sacu_s0033g011070 99.5% 25.9% **LPILDSSSAPPNLFDGTTRLYISLTCPFAQRPWIAKNYKGL-DNIELIAIN-LADKPKWYLEKVYPVGKVPSLEHDGKVK**

10 Bradi1g66030 100.0% 21.3% **PPSLTSTSEPPPLFDGTTRLYVAYHCPYAQRAWITRNCKGLQDKIKIVAID-LADRPVWYKEKVYPENKVPSLEHDNQVK**

11 Bradi1g66020 100.0% 22.2% **PPALGAVSEPPPLFDGTTRLYICYFCPFAQRAWVTRNCKGLREEIKLVGID-LQDKPAWYKEKVYPRGTVPSLEHDGKVT**

12 Bradi1g42890 70.9% 12.4% **HASLASTSEPPPLFDGTTRLYVAYHCPYTRRAWINRNYK---DKIKVVVIG-LADRPSRYKENVYPETK-----HDNQVK**

13 Sacu_s0025g009288 90.3% 23.3% **-------------------------MLLLMSLWAELYVRGL-ENIEVIPIN-IVDKPQWYIKKISASGKLPAFEHKTTIK**

14 Bradi1g66035 75.5% 18.3% **---------------------MSYTCPYAQRAWVTRNCKGLQKKIELVPLE-MADRPAWYKE-IYPKN------------**

15 Pp3c4_26900_GSTI 99.0% 23.0% **LTAMEASVESSRLPNMKRRVFEDGQCPYCQRVWLQLEEKKIPYQVEKINMRCYGDKPAWFTKMV-PSGLLPVIELDGRII**

16 Mapoly0019s0024 99.5% 23.5% **VGCASAAMVETRVAHATERIFGPGNCPYCERLWLLLEEKQLNYTVEKINMWCYGQKPEWYTRMV-PSGLLPAVVLDGKLL**

17 90536_Smoellendorffii 99.5% 24.0% **LPGYNAAAAAARLPNVQQRDFGDTSCPYCQRVWLQLEEKRIPYKVEKINMNCYGPKPAWYLEKV-PSGLLPALELKGRLL**

18 Cre01g044700 99.5% 24.0% **PTVLSFSDNAPLHPMATRRTFGSSACPYCHKVWMQLEEKRIPYEIEKINMRCYGDKPASFMAKV-PNGLLPVIELDGRVV**

19 AaBonn_Sc2ySwM_228_2049_1_GSTI 99.5% 24.0% **MAAVEAPAGLPRLAHATERLFGSDTCPYCERVWLQLEEKQVSYAVEKINMRCYGPKPDWFTRMV-PSGLLPVVKLDGNVV**

cov pid  **81**  **. 1 . . . . : .** **160**

1 AT1G19570_DHAR1_outgroup 100.0% 100.0% **TDSDVIVGILEEKYPDPPL--------KTPAEFASVGS-NIFGTFGTFLLVELEALENHLKSHDGPFIAGEVSAVDLSLA**

2 G22068_CHBRA263g00260_DHAR_outgroup 83.7% 44.0% **ADSDVIVGILEKDFPEPSLAVPD------------------------------DKV-------VGPFVNGDISSADMSLV**

3 AT5G02780_GSTL1 99.0% 22.8% **GESLDLIKYVDSNFDGPSLYPEDSAKREFGEELLKYVDETFVKTVFGSFASAFDHVENALKKFDGPFFLGELSLVDIAYI**

4 AT3G55040_GSTL2 100.0% 18.5% **GESLDLIKYIDTNFEGPSLTPDGLEKQVVADELLSYTD-SFSKAVRSTLDVAFDYIEQALSKFEGPFFLGQFSLVDVAYA**

5 AT5G02790_GSTL3 99.5% 23.7% **GESLDLIKYLDNTFEGPSLYPEDHAKREFGDELLKYTD-TFVKTMYVSLAPVLDYLENALYKFDGPFFLGQLSLVDIAYI**

6 Pp3c10_15380 99.5% 20.4% **GESMDLLTYLDDHFGGPKLAPTEESKKQAAAELLQYAD-TFNKLGFTGLAPAFDFLENALAKFEGPLFLGNFGLVDIVYA**

7 Azfi_s0042g026899 99.5% 24.5% **GESLDLLEYIDQNFGEPRLFPEGTAKEEARKELWTFSD-KFNGEFFKALGPLLDKLENDLGKFEGPYFLGEISAVDLAYA**

8 Azfi_s0121g046868 75.5% 15.6% **VESLEILQYMENNFNGPKLFPQVSPKW----------------------RPLFDQLELALARFEGPYLLGSISMVDFVYV**

9 Sacu_s0033g011070 99.5% 25.9% **GESLDLLEYLDHCFPGPDLFPKEHAKVEAKKELWNFSD-EFNAQFFKAFGPLLDKLEDALGKFEGPFFLGEISAVDFAYA**

10 Bradi1g66030 100.0% 21.3% **GESLDLVKYIDSNFEGPALLPEDSAKKQSAEELLAYTD-EFNKALYSSIVAALDKIEAALGKFDGPFLLGQFSSVDIAYL**

11 Bradi1g66020 100.0% 22.2% **GESLDLIKYIDSNFEGPALLPQDPAKRQFADELIAYAG-AFTKALYSPLVAALDKIEAALSKFDGPFFLGQFSLADIAYV**

12 Bradi1g42890 70.9% 12.4% **GESLDLIKYIDRNFEGPSLLPEDSAQ-QSAEELLVYTD-EFNEALHSSI--------------------GDSEETGTAYL**

13 Sacu_s0025g009288 90.3% 23.3% **VESIEILGYMDNNFNGPKLFPTVSPKKEATNELLQNVE-LMNQRVFTYLGPLFEQLELALARFEGPFFLGHITVVDFVYI**

14 Bradi1g66035 75.5% 18.3% **---------------------TDSEKRRFADELLAYSD-TFNLAMLSGLSPVTAEAEFSLSKFDGPFFLGQFSLVDIAYA**

15 Pp3c4_26900_GSTI 99.0% 23.0% **TESMDIMILIEKRFPEP-LLPAGGPELAAVNSLLGLER-RLAGAWMNRLENTMDKVNSALQTFGGPYFLGSFSLVDAVYA**

16 Mapoly0019s0024 99.5% 23.5% **TESLDIMLLLESKFSDPMLPRPGSPQWSSLDGLLQLER-RLFGAWLSRLDSTMDTVNAALNKFGGPYFLGPFSLIDAVYA**

17 90536_Smoellendorffii 99.5% 24.0% **TESLDIMLILEEAFPEPLLPPKGSPKANAVDGLLRLER-LLAGAWLSRLERAMDDVDGALAKFGGPFFLGDFSLVDAVYA**

18 Cre01g044700 99.5% 24.0% **TESAVIMNLLEQAFPDPLMPPQGTPERARADQLMRLER-RFFSDWLGWLEATVDAVAAELERAGGPYFLGSISLVDITFA**

19 AaBonn_Sc2ySwM_228_2049_1_GSTI 99.5% 24.0% **TESLDIMLLLEKRFPDPLLPAKGTPENDALPGLLRLER-VLMGGWLSCISNSLDKVDAALRTFGGPYFLGSFSLVDAVYA**

cov pid **161**  **. . . 2 . ]** **218**

1 AT1G19570_DHAR1_outgroup 100.0% 100.0% **PKLYHLQVALGHFKSWSVPESFPHVHNYMKTLFSLDSFEKTKTEEKYVISGWAPKVNP**

2 G22068_CHBRA263g00260_DHAR_outgroup 83.7% 44.0% **PKLYHLRIALKHYRGWSIPEDLKNVHAYIKATESRDSFQKTAAAEEYVIKGWEKHVVG**

3 AT5G02780_GSTL1 99.0% 22.8% **PFIERFQVFLDEVFKYEIIIGRPNLAAWIEQMNKMVAYTQTKTDSEYVVNYFKRFM--**

4 AT3G55040_GSTL2 100.0% 18.5% **PFIERFRLILSDVMNVDITSGRPNLALWIQEMNKIEAYTETRQDPQELVERYKRRVQA**

5 AT5G02790_GSTL3 99.5% 23.7% **PFIERFQTVLNELFKCDITAERPKLSAWIEEINKSDGYAQTKMDPKEIVEVFKKKFM-**

6 Pp3c10_15380 99.5% 20.4% **PFIERFEIAFGGIRNYDIRAGRPRLAKWIEAMDNVEAYSSTKVPRATLLELYKKMLEN**

7 Azfi_s0042g026899 99.5% 24.5% **PFIERFDLLSLDLLDYNIFEGRPKLSKWFEAFNTVDAYTSTKQEPKGLVESLKKHLGR**

8 Azfi_s0121g046868 75.5% 15.6% **PFIERFNILLQELFHYNIFERRPRLFQWLAALNELGAYTSTKVDNSLIIEDMRKSLV-**

9 Sacu_s0033g011070 99.5% 25.9% **PFIERFELLSLDLLDYKIFEGRPKLTKWFETLNTLDAYTSTRHEPKSLTENLKKHLGR**

10 Bradi1g66030 100.0% 21.3% **PFIERFQIFYSGIKNYDITKGRPNFQKYIEEANKIDAYTQTKLEPQFLLDQTKKRLGI**

11 Bradi1g66020 100.0% 22.2% **TILERVQIYYSHLRNYEIAKGRPNLEKYIEEMNKIEAYTQTKNEPLNLLDMAKRHLKI**

12 Bradi1g42890 70.9% 12.4% **AFVVRFQIFFSGIKNCDITKGRLNVQKYI-----------------------------**

13 Sacu_s0025g009288 90.3% 23.3% **PFVERLNILLQEIFNYNIFERRPRLYKWLRALNELDAYTITKVNPKVIINDFKKSM--**

14 Bradi1g66035 75.5% 18.3% **PFVDGFQIFFADIKNYDTTAERSNTRRFIEEMNDIAAYAHTKYDAQELVALTKKKLG-**

15 Pp3c4_26900_GSTI 99.0% 23.0% **PFLERTAASMPYWPGVKVRDRWNAVNLWFDAMDSRPSYQAMKSDDFTHTHDLEPQIGP**

16 Mapoly0019s0024 99.5% 23.5% **PFLERIAASMPYWPGLVVRDRWPAVNRWYDAMDSRPAYQAIKSDDFYIVHNLEPQIGA**

17 90536_Smoellendorffii 99.5% 24.0% **PFLERIAASMPYWQGVMIRTRWPHLQAWFDAMDAKPSYQAVKSDDYTITHTLEPQIGA**

18 Cre01g044700 99.5% 24.0% **PMLERAAASLTYYKGFHLRGRWPAVDRWFAAMESRPTYLGTRSDYYTHAHDLPPQLGG**

19 AaBonn_Sc2ySwM_228_2049_1_GSTI 99.5% 24.0% **PFLERIAASVPYWAGLTVRSRWPALNAWYDAMDTRPIYQAMKSDDFTITHTLEPQIGP**

>AT1G19570_DHAR1_outgroup

EICVKAAVGAPDHLGD---------CPFSQRALLTLEEKSLTYKIHLIN---LSDKPQWFLD-ISPQGKVPV

LKIDDKWVTDSDVIVGILEEKYPDPPL--------KTPAEFASVGS-NIFGTFGTFLLVELEALENHLKSHD

GPFIAGEVSAVDLSLAPKLYHLQVALGHFKSWSVPESFPHVHNYMKTLFSLDSFEKTKTEEKYVISGWAPKV

NP

>G22068_CHBRA263g00260_DHAR_outgroup

DVAIKAATGRPDLVGD---------CPFSQRVTMTLEEKGIKYNPILVD---TSNKPEWFLE-ANPSGKVPV

IKYQDKWIADSDVIVGILEKDFPEPSLAVPD------------------------------DKV-------V

GPFVNGDISSADMSLVPKLYHLRIALKHYRGWSIPEDLKNVHAYIKATESRDSFQKTAAAEEYVIKGWEKHV

VG

>AT5G02780_GSTL1

QVPLDATSDPPALFDGTTRLYISYTCPFAQRVWITRNLKGLQDEIKLVPID-LPNRPAWLKEKVNPANKVPA

LEHNGKITGESLDLIKYVDSNFDGPSLYPEDSAKREFGEELLKYVDETFVKTVFGSFASAFDHVENALKKFD

GPFFLGELSLVDIAYIPFIERFQVFLDEVFKYEIIIGRPNLAAWIEQMNKMVAYTQTKTDSEYVVNYFKRFM

--

>AT3G55040_GSTL2

VPELDSSSEPVQVFDGSTRLYISYTCPFAQRAWIARNYKGLQNKIELVPID-LKNRPAWYKEKVYSANKVPA

LEHNNRVLGESLDLIKYIDTNFEGPSLTPDGLEKQVVADELLSYTD-SFSKAVRSTLDVAFDYIEQALSKFE

GPFFLGQFSLVDVAYAPFIERFRLILSDVMNVDITSGRPNLALWIQEMNKIEAYTETRQDPQELVERYKRRV

QA

>AT5G02790_GSTL3

PAPLDATSDPPSLFDGTTRLYTSYVCPFAQRVWITRNFKGLQEKIKLVPLD-LGNRPAWYKEKVYPENKVPA

LEHNGKIIGESLDLIKYLDNTFEGPSLYPEDHAKREFGDELLKYTD-TFVKTMYVSLAPVLDYLENALYKFD

GPFFLGQLSLVDIAYIPFIERFQTVLNELFKCDITAERPKLSAWIEEINKSDGYAQTKMDPKEIVEVFKKKF

M-

>Pp3c10_15380

REVLDSKSASPAIFDGTTRLYFSSRCPYAQRVWVAVKYKGL-DEIECVEIS-LSDKPTWYKEKVYPVGKVPA

LEHNGTVTGESMDLLTYLDDHFGGPKLAPTEESKKQAAAELLQYAD-TFNKLGFTGLAPAFDFLENALAKFE

GPLFLGNFGLVDIVYAPFIERFEIAFGGIRNYDIRAGRPRLAKWIEAMDNVEAYSSTKVPRATLLELYKKML

EN

>Azfi_s0042g026899

IPVYDSSSSPPNLFDGTTRLYINVTCPYAQRTWIARNFKGL-NQIELLGIN-LSDKPKWYLEKVYPAGKVPS

LEHNGKVTGESLDLLEYIDQNFGEPRLFPEGTAKEEARKELWTFSD-KFNGEFFKALGPLLDKLENDLGKFE

GPYFLGEISAVDLAYAPFIERFDLLSLDLLDYNIFEGRPKLSKWFEAFNTVDAYTSTKQEPKGLVESLKKHL

GR

>Azfi_s0121g046868

LLPLNSKSPPPPLFNGTPRLYISLTCPYSQRVWIARNFK------------------------------VPA

LEHNGRVKVESLEILQYMENNFNGPKLFPQVSPKW----------------------RPLFDQLELALARFE

GPYLLGSISMVDFVYVPFIERFNILLQELFHYNIFERRPRLFQWLAALNELGAYTSTKVDNSLIIEDMRKSL

V-

>Sacu_s0033g011070

LPILDSSSAPPNLFDGTTRLYISLTCPFAQRPWIAKNYKGL-DNIELIAIN-LADKPKWYLEKVYPVGKVPS

LEHDGKVKGESLDLLEYLDHCFPGPDLFPKEHAKVEAKKELWNFSD-EFNAQFFKAFGPLLDKLEDALGKFE

GPFFLGEISAVDFAYAPFIERFELLSLDLLDYKIFEGRPKLTKWFETLNTLDAYTSTRHEPKSLTENLKKHL

GR

>Bradi1g66030

PPSLTSTSEPPPLFDGTTRLYVAYHCPYAQRAWITRNCKGLQDKIKIVAID-LADRPVWYKEKVYPENKVPS

LEHDNQVKGESLDLVKYIDSNFEGPALLPEDSAKKQSAEELLAYTD-EFNKALYSSIVAALDKIEAALGKFD

GPFLLGQFSSVDIAYLPFIERFQIFYSGIKNYDITKGRPNFQKYIEEANKIDAYTQTKLEPQFLLDQTKKRL

GI

>Bradi1g66020

PPALGAVSEPPPLFDGTTRLYICYFCPFAQRAWVTRNCKGLREEIKLVGID-LQDKPAWYKEKVYPRGTVPS

LEHDGKVTGESLDLIKYIDSNFEGPALLPQDPAKRQFADELIAYAG-AFTKALYSPLVAALDKIEAALSKFD

GPFFLGQFSLADIAYVTILERVQIYYSHLRNYEIAKGRPNLEKYIEEMNKIEAYTQTKNEPLNLLDMAKRHL

KI

>Bradi1g42890

HASLASTSEPPPLFDGTTRLYVAYHCPYTRRAWINRNYK---DKIKVVVIG-LADRPSRYKENVYPETK---

--HDNQVKGESLDLIKYIDRNFEGPSLLPEDSAQ-QSAEELLVYTD-EFNEALHSSI---------------

-----GDSEETGTAYLAFVVRFQIFFSGIKNCDITKGRLNVQKYI---------------------------

--

>Sacu_s0025g009288

-------------------------MLLLMSLWAELYVRGL-ENIEVIPIN-IVDKPQWYIKKISASGKLPA

FEHKTTIKVESIEILGYMDNNFNGPKLFPTVSPKKEATNELLQNVE-LMNQRVFTYLGPLFEQLELALARFE

GPFFLGHITVVDFVYIPFVERLNILLQEIFNYNIFERRPRLYKWLRALNELDAYTITKVNPKVIINDFKKSM

--

>Bradi1g66035

---------------------MSYTCPYAQRAWVTRNCKGLQKKIELVPLE-MADRPAWYKE-IYPKN----

-----------------------------TDSEKRRFADELLAYSD-TFNLAMLSGLSPVTAEAEFSLSKFD

GPFFLGQFSLVDIAYAPFVDGFQIFFADIKNYDTTAERSNTRRFIEEMNDIAAYAHTKYDAQELVALTKKKL

G-

>Pp3c4_26900_GSTI

LTAMEASVESSRLPNMKRRVFEDGQCPYCQRVWLQLEEKKIPYQVEKINMRCYGDKPAWFTKMV-PSGLLPV

IELDGRIITESMDIMILIEKRFPEP-LLPAGGPELAAVNSLLGLER-RLAGAWMNRLENTMDKVNSALQTFG

GPYFLGSFSLVDAVYAPFLERTAASMPYWPGVKVRDRWNAVNLWFDAMDSRPSYQAMKSDDFTHTHDLEPQI

GP

>Mapoly0019s0024

VGCASAAMVETRVAHATERIFGPGNCPYCERLWLLLEEKQLNYTVEKINMWCYGQKPEWYTRMV-PSGLLPA

VVLDGKLLTESLDIMLLLESKFSDPMLPRPGSPQWSSLDGLLQLER-RLFGAWLSRLDSTMDTVNAALNKFG

GPYFLGPFSLIDAVYAPFLERIAASMPYWPGLVVRDRWPAVNRWYDAMDSRPAYQAIKSDDFYIVHNLEPQI

GA

>90536_Smoellendorffii

LPGYNAAAAAARLPNVQQRDFGDTSCPYCQRVWLQLEEKRIPYKVEKINMNCYGPKPAWYLEKV-PSGLLPA

LELKGRLLTESLDIMLILEEAFPEPLLPPKGSPKANAVDGLLRLER-LLAGAWLSRLERAMDDVDGALAKFG

GPFFLGDFSLVDAVYAPFLERIAASMPYWQGVMIRTRWPHLQAWFDAMDAKPSYQAVKSDDYTITHTLEPQI

GA

>Cre01g044700

PTVLSFSDNAPLHPMATRRTFGSSACPYCHKVWMQLEEKRIPYEIEKINMRCYGDKPASFMAKV-PNGLLPV

IELDGRVVTESAVIMNLLEQAFPDPLMPPQGTPERARADQLMRLER-RFFSDWLGWLEATVDAVAAELERAG

GPYFLGSISLVDITFAPMLERAAASLTYYKGFHLRGRWPAVDRWFAAMESRPTYLGTRSDYYTHAHDLPPQL

GG

>AaBonn_Sc2ySwM_228_2049_1_GSTI

MAAVEAPAGLPRLAHATERLFGSDTCPYCERVWLQLEEKQVSYAVEKINMRCYGPKPDWFTRMV-PSGLLPV

VKLDGNVVTESLDIMLLLEKRFPDPLLPAKGTPENDALPGLLRLER-VLMGGWLSCISNSLDKVDAALRTFG

GPYFLGSFSLVDAVYAPFLERIAASVPYWAGLTVRSRWPALNAWYDAMDTRPIYQAMKSDDFTITHTLEPQI

GP

**MSRB**

cov pid 1 [ . . . . : . . . 80

1 AT1G53670.1/71-197 100.0% 100.0% ASLSENEWKKRLTPEQYYITRQKGTERAFTGEYWNSKTPGVYNCVCCDTPLFDSSTKFDSGTGWPSYYQPIGNNVKTKLD

2 AT4G21860.1/73-198 100.0% 44.4% VNKPEEEWRAILSPEQFRILRQKGTEYPGTGEYNKVFDDGIYCCAGCGTPLYKSTTKFDSGCGWPAFFDGLPGAITRTPD

3 AT4G04800.1/47-172 100.0% 43.7% VQKGDEEWRAILSPEQFRILRQKGTEYPGTGEYVNFDKEGVYGCVGCNAPLYKSTTKFNAGCGWPAFFEGIPGAITRTTD

4 AT4G04810.1/8-133 100.0% 40.5% VKKTEEEWRAVLSPEQFRILRQKGTETPGTEEYDKFFEEGIFSCIGCKTPLYKSTTKFDAGCGWPAFFEGLPGAINRAPD

5 AT4G04830.1/8-133 100.0% 42.9% VQKTEEEWRAVLSPEQFRILRQKGTEKPGTGEYDKFFEEGIFDCVGCKTPLYKSTTKFDSGCGWPAFFEGLPGAINRTPD

6 AT4G04840.1/24-149 100.0% 39.7% IKKSNEEWRTVLSPEQFKILREKSIEKRGSGEYVKLFEEGIYCCVGCGNPVYKSTTKFDSGCGWPAFFDAIPGAINRTEE

7 AT4G21830.1/15-140 100.0% 39.7% FQKQDEEWRAVLSPEQFRVLRLKGTDKRGKGEFTKKFEEGTYSCAGCGTALYKSTTKFDSGCGWPAFFDAIPGAIKQTPE

8 AT4G21840.1/14-139 100.0% 39.7% FQKQDEEWRAVLSPEQFRVLRLKGTDKRGKGEFTKKFEEGTYSCAGCGTALYKSTTKFDSGCGWPAFFDAIPGAIKQTPE

9 AT4G21850.1/15-140 100.0% 40.5% VQKKDQDWRAILSPEQFRVLREKGTENRGKGEYTKLFDDGIYSCAGCATPLYKSTTKFDSGCGWPSFFDAIPGAIKQTPE

10 Pp3c6_10330V3.1/75-201 100.0% 72.6% KTFSDEEWKKRLSQQQFYVARKKGTERPFTGEYWNTKTAGTYLCVCCKTPLFSSKTKFDSGTGWPSYYDTIGDNVKSHMD

11 Pp3c27_120V3.1/76-201 100.0% 47.6% VQKSEEEWRAILSPEQFRILRQKGTEYPGTGEYNKNKAEGVYNCAGCGTPLYKSTTKFDSGCGWPAFFEGLPGAINETTD

12 Pp3c16_290V3.1/11-136 100.0% 44.4% VRKSDEEWRAILSPEQFRILRKKGTEYPNSGEYNKTYNDGVYNCAGCEAPLYKSTTKFDSGCGWPAFFEGIPGAINETRD

13 Mapoly0057s0060.1/97-223 100.0% 73.4% KSISDSEWQKRLTREQFNVARQKGTERAFTGEYWNTKTSGTYLCVCCQTPLFDSSTKFDSGTGWPSYWEKIGDNVKSETD

14 Mapoly0057s0057.1/28-154 100.0% 73.4% KSMSDSEWQNRLTKEQFYVARKKGTERAFTGEYWNTKTPGTYTCACCDTPLFDSSTKFDSGTGWPSYWQPIGNSVKSARD

15 g3238/164-289 100.0% 45.2% VQKSEDEWRAVLSPEQFRILRKKGTEYPGTGKYNKFYEEGVYECAGCGTPLYKSTTKFDSGCGWPAFYEGLPGAINRTVD

16 G41095/29-155 100.0% 64.5% ANMTDVDWKKILTPEQYRVCRRKGTEMAFSGQYWNTKTKGMYLCVCCKTALFRSQTKFDSGTGWPSFYDKVDLNVKMEMD

17 Azfi_s0301.g063683/77-219 100.0% 70.2% PALSDEEWKKRLTPEQYVITRKKGTERAFSGAYWKTKSPGVYECICCGTPLFDSKTKFDSGTGWPSYFEPIENNVKSEMD

18 Azfi_s0114.g046037/104-229 100.0% 45.2% VQRSEQEWRAILAPEQFRILRQKGTEYPGTGKYNKVYDEGIYECAGCGTPLYKSITKFDSGCGWPAFYEGLPGAINETVD

19 Azfi_s0383.g067445/2-133 100.0% 34.1% TEDMLHEMRCLMFMIQYTMIHKMQVLCLGVAEDISHEMEGVYLCAGCETPLYKSTTKFDSGCGWPAFFDGLPGAINRTVD

20 Sacu_v1.1_s0042.g012768/49-171 96.8% 71.0% PALTDEEWKKRLTPEQYTITRKKGTERAFTGEYWNTKTPGLYECRCCGTPLFDSRTKFDSGTGWPSYYEPIADNVKSEMD

21 Sacu_v1.1_s1799.g028179/352-477 100.0% 53.2% SMTDDMDWKDKLTPEQYRVLREKGTERPFTGEYVDYHGDGSFKCVGCGNMLFSSDTKFDSGTGWPSFEEAIPGSVAYHVD

22 Sacu_v1.1_s0062.g015489/51-211 100.0% 45.2% VQKSDEEWRAVLSPEQFRILRQKGTEWAGTGQYDKFFEEGVYGCAGCGTPLYKSTTKFNSGCGWPAFYEGLPGAINENVD

23 92187_Selmo/3-121 93.5% 71.6% KSVSDTEWKEKLTDEQFYVTRQKGTERAFSGKYWNTKTAGIYECICCGTPLFNSITKFDSGTGWPSYYEAIGNNVKSESD

24 170312_Selmo/6-131 100.0% 44.4% GNKSEEEWRAILSPEQFRVLRRKGTEFAGTGIYNKHFEGGVYECAGCGTPLYKSDTKFDSGCGWPAFFQGLPGAINRNAD

25 98474_Selmo/7-132 100.0% 42.9% VNKTEEEWRAVLNSEQFRILRMKGTEMPGSGEYNKFYKDGVYNCGGCGTPLYKSTAKFDSGCGWPAFFEGLPGAINRTVD

26 18637_Selmo/3-98 75.8% 36.4% -QRREEEWRAVLNPEQ--TLRIK---RTGTGKHN---EDGVHNCAGCGTPLYKSTT--DSDC----IYEGLPGAINRTVD

27 18636_Selmo/3-98 75.8% 36.4% -QRREEEWRAVLNPEQ--TLRIK---RTGTGKHN---EDGVHNCAGCGTPLYKSTT--DSDC----IYEGLPGAINRTVD

28 AaBonn_Sc2ySwM_344.1824.2/25-143 93.5% 70.7% KQISDEEWKKKLSKEQYNVARQKGTERAFTGQYWNTKTPGTYHCVCCDTPLFQSSTKFDSGTGWPSYWEYVSDNVKSESD

29 AaBonn_Sc2ySwM_117.2635.1_manually_corrected/1-124 98.4% 46.8% --MSDSEWQRCLSPMQYKVLRQKVTERPFTGEYYSHYESGTYKCVGCNNSLYSSSAKFDSGCGWPVFYEALDGAIETQTD

30 AaBonn_Sc2ySwM_344.851.2/130-255 100.0% 42.9% VQKSDEEWRAVLSPEQFRILRQKGTEYPGTGKYNKHKEDGVYECAGCGTPLYKSTTKFDSGCGWPAFFEGLPGAITETRD

31 Bradi1g41830.3/64-190 100.0% 78.2% ASMSDEDLKERLTKEQYYVTRQKGTERAFTGEYWNTKTPGIYHCICCDTPLFESSTKFDSGTGWPSYYRPVGDNVKNKLD

32 Bradi2g25820.1/92-217 100.0% 46.0% VQKSEEEWEAVLTPEQFRILRRKGTEYPGTGEYDKFFDEGIYGCAGCGTPLYKSSTKFNSGCGWPAFYEGFPGAIKRTAD

33 Bradi1g61800.1/7-132 100.0% 42.1% KPKTEEEWRAVLSPEQFRILRLKDTELPGTGEYNKFYGSGVYNCAGCGTPLYKSTTKFDSGCGWPAFFEGLPGAIQRTPD

cov pid 81 . 1 . . ] 126

1 AT1G53670.1/71-197 100.0% 100.0% IFMPRQEVVCAVCNAHLGHVFDDG--PRPTGKRYCLNSAALKLNAL

2 AT4G21860.1/73-198 100.0% 44.4% PDGRRIEITCAACGGHLGHVFKGEGFPTPTDERHCVNSISLKFTPE

3 AT4G04800.1/47-172 100.0% 43.7% PDGRRIEINCATCGGHLGHVFKGEGFATPTDERHCVNSVSLKFTPA

4 AT4G04810.1/8-133 100.0% 40.5% PDGRRTEITCAVCDGHLGHVHKGEGYSTPTDERLCVNSVSINFNPA

5 AT4G04830.1/8-133 100.0% 42.9% PDGRRTEITCAACDGHLGHVFKGEGYGNPTDERHCVNSVSISFNPA

6 AT4G04840.1/24-149 100.0% 39.7% RAGLRYEITCTKCDGHLGHVLKNEGFPTPTDERHCVNSVALKFSSA

7 AT4G21830.1/15-140 100.0% 39.7% AGGRRMEITCAVCDGHLGHVFKGEGYSTPTDQRHCVNSVSLKFSSA

8 AT4G21840.1/14-139 100.0% 39.7% AGGRRMEITCAVCDGHLGHVFKGEGYSTPTDQRHCVNSVSLKFASA

9 AT4G21850.1/15-140 100.0% 40.5% AGGRRMEITCAACDGHLGHVVKGEGFPTATDERHCVNSVSLKFSEI

10 Pp3c6_10330V3.1/75-201 100.0% 72.6% PFMPRTEVVCAVCDAHLGHVFDDG--PRPTGKRYCINSAAIDLKAE

11 Pp3c27_120V3.1/76-201 100.0% 47.6% ADGRRVEITCAACGGHLGHVFRGEGFPTPTDARHCVNSVSLKFTPA

12 Pp3c16_290V3.1/11-136 100.0% 44.4% ADGRRVEITCAACGGHLGHVFKGEGFPTPTDARHCVNSVSLKFTPA

13 Mapoly0057s0060.1/97-223 100.0% 73.4% PFMPRTEVLCAKCDAHLGHVFDDG--PRPTGKRYCINSASIKLKPE

14 Mapoly0057s0057.1/28-154 100.0% 73.4% PFMPRTEVLCIKCDAHLGHVFDDG--PHPTGERYCINSACLKLKPE

15 g3238/164-289 100.0% 45.2% ADGYRIEITCAACGGHLGHVFKGEGFPTPTNERHCVNSISLTFKAP

16 G41095/29-155 100.0% 64.5% PFMPRTEVSCAKCNAHLGHVFSDG--PPPTLKRYCINSVALVLKPD

17 Azfi_s0301.g063683/77-219 100.0% 70.2% PFMPRTEVVCSKCNAHLGHVFNDG--PPPTGKRYCINRLLVLYLEI

18 Azfi_s0114.g046037/104-229 100.0% 45.2% ADGYRIEITCAACGGHLGHVFKGEGFPTPTNERHCVNSISLKFIPA

19 Azfi_s0383.g067445/2-133 100.0% 34.1% ADGRRIEITCAACGGHLGHVFKGELFPTPTNERHCVNSVSLKFTPA

20 Sacu_v1.1_s0042.g012768/49-171 96.8% 71.0% PFMPRTEVLCAKCDAHLGHVFNDG--PPPTGKRYCINR----YRVA

21 Sacu_v1.1_s1799.g028179/352-477 100.0% 53.2% HGMVRTEVTCAKCGSHLGHVFEDG--PGASGKRYCINSVCLKLEKP

22 Sacu_v1.1_s0062.g015489/51-211 100.0% 45.2% ADGYRIEITCAACGGHLGHVFKGEGFPTPTNERHCVNSVSLKFTPA

23 92187_Selmo/3-121 93.5% 71.6% PFMPRTEVKCSKCDAHLGHVFDDG--PPPTGKRYCINR--------

24 170312_Selmo/6-131 100.0% 44.4% ADGRRVEITCAACGGHLGHVFKGEGYRTPTDERHCVNSVSLKFTPG

25 98474_Selmo/7-132 100.0% 42.9% ADGYRTEITCAACGGHLGHVFKGEGFPTPTNERHCVNSISLKFAPG

26 18637_Selmo/3-98 75.8% 36.4% AEGHRIEITCAACGRHLGHVFNGEGFPTPTD---------------

27 18636_Selmo/3-98 75.8% 36.4% AEGHRIEITCAACGRHLGHVFNGEGFPTPTD---------------

28 AaBonn_Sc2ySwM_344.1824.2/25-143 93.5% 70.7% PFMPRTEVMCAVCDAHLGHVFNDG--PPPTKKRYCINR--------

29 AaBonn_Sc2ySwM_117.2635.1_manually_corrected/1-124 98.4% 46.8% SDGRRTEIVCANCNGHLGHLFTGEGFPTPTDERHCVNSICLKFCPE

30 AaBonn_Sc2ySwM_344.851.2/130-255 100.0% 42.9% PDGRRIEITCTACGGHLGHVFKGEGFKTPTDARHCVNSVSLTFTPA

31 Bradi1g41830.3/64-190 100.0% 78.2% IFMPRTEALCAVCDAHLGHVFDDG--PPPTGKRYCINSASLKLKPQ

32 Bradi2g25820.1/92-217 100.0% 46.0% PDGRRVEITCAACDGHLGHVFKGEGFNTPTDERHCVNSISLKFIPA

33 Bradi1g61800.1/7-132 100.0% 42.1% PDGRRVEITCTACGGHLGHVFKGEGFKTPTDERHCVNSVSMKFTPA

>AT1G53670.1/71-197 AtMSRB1

ASLSENEWKKRLTPEQYYITRQKGTERAFTGEYWNSKTPGVYNCVCCDTPLFDSSTKFDSGTGWPSYYQPIG

NNVKTKLDIFMPRQEVVCAVCNAHLGHVFDDG--PRPTGKRYCLNSAALKLNAL

>AT4G21860.1/73-198 AtMSRB2

VNKPEEEWRAILSPEQFRILRQKGTEYPGTGEYNKVFDDGIYCCAGCGTPLYKSTTKFDSGCGWPAFFDGLP

GAITRTPDPDGRRIEITCAACGGHLGHVFKGEGFPTPTDERHCVNSISLKFTPE

>AT4G04800.1/47-172 AtMSRB3

VQKGDEEWRAILSPEQFRILRQKGTEYPGTGEYVNFDKEGVYGCVGCNAPLYKSTTKFNAGCGWPAFFEGIP

GAITRTTDPDGRRIEINCATCGGHLGHVFKGEGFATPTDERHCVNSVSLKFTPA

>AT4G04810.1/8-133 AtMSRB4

VKKTEEEWRAVLSPEQFRILRQKGTETPGTEEYDKFFEEGIFSCIGCKTPLYKSTTKFDAGCGWPAFFEGLP

GAINRAPDPDGRRTEITCAVCDGHLGHVHKGEGYSTPTDERLCVNSVSINFNPA

>AT4G04830.1/8-133 AtMSRB5

VQKTEEEWRAVLSPEQFRILRQKGTEKPGTGEYDKFFEEGIFDCVGCKTPLYKSTTKFDSGCGWPAFFEGLP

GAINRTPDPDGRRTEITCAACDGHLGHVFKGEGYGNPTDERHCVNSVSISFNPA

>AT4G04840.1/24-149 AtMSRB6

IKKSNEEWRTVLSPEQFKILREKSIEKRGSGEYVKLFEEGIYCCVGCGNPVYKSTTKFDSGCGWPAFFDAIP

GAINRTEERAGLRYEITCTKCDGHLGHVLKNEGFPTPTDERHCVNSVALKFSSA

>AT4G21830.1/15-140 AtMSRB7

FQKQDEEWRAVLSPEQFRVLRLKGTDKRGKGEFTKKFEEGTYSCAGCGTALYKSTTKFDSGCGWPAFFDAIP

GAIKQTPEAGGRRMEITCAVCDGHLGHVFKGEGYSTPTDQRHCVNSVSLKFSSA

>AT4G21840.1/14-139 AtMSRB8

FQKQDEEWRAVLSPEQFRVLRLKGTDKRGKGEFTKKFEEGTYSCAGCGTALYKSTTKFDSGCGWPAFFDAIP

GAIKQTPEAGGRRMEITCAVCDGHLGHVFKGEGYSTPTDQRHCVNSVSLKFASA

>AT4G21850.1/15-140 AtMSRB9

VQKKDQDWRAILSPEQFRVLREKGTENRGKGEYTKLFDDGIYSCAGCATPLYKSTTKFDSGCGWPSFFDAIP

GAIKQTPEAGGRRMEITCAACDGHLGHVVKGEGFPTATDERHCVNSVSLKFSEI

>Pp3c6_10330V3.1/75-201

KTFSDEEWKKRLSQQQFYVARKKGTERPFTGEYWNTKTAGTYLCVCCKTPLFSSKTKFDSGTGWPSYYDTIG

DNVKSHMDPFMPRTEVVCAVCDAHLGHVFDDG--PRPTGKRYCINSAAIDLKAE

>Pp3c27_120V3.1/76-201

VQKSEEEWRAILSPEQFRILRQKGTEYPGTGEYNKNKAEGVYNCAGCGTPLYKSTTKFDSGCGWPAFFEGLP

GAINETTDADGRRVEITCAACGGHLGHVFRGEGFPTPTDARHCVNSVSLKFTPA

>Pp3c16_290V3.1/11-136

VRKSDEEWRAILSPEQFRILRKKGTEYPNSGEYNKTYNDGVYNCAGCEAPLYKSTTKFDSGCGWPAFFEGIP

GAINETRDADGRRVEITCAACGGHLGHVFKGEGFPTPTDARHCVNSVSLKFTPA

>Mapoly0057s0060.1/97-223

KSISDSEWQKRLTREQFNVARQKGTERAFTGEYWNTKTSGTYLCVCCQTPLFDSSTKFDSGTGWPSYWEKIG

DNVKSETDPFMPRTEVLCAKCDAHLGHVFDDG--PRPTGKRYCINSASIKLKPE

>Mapoly0057s0057.1/28-154

KSMSDSEWQNRLTKEQFYVARKKGTERAFTGEYWNTKTPGTYTCACCDTPLFDSSTKFDSGTGWPSYWQPIG

NSVKSARDPFMPRTEVLCIKCDAHLGHVFDDG--PHPTGERYCINSACLKLKPE

>g3238/164-289 CHBRA117g00330

VQKSEDEWRAVLSPEQFRILRKKGTEYPGTGKYNKFYEEGVYECAGCGTPLYKSTTKFDSGCGWPAFYEGLP

GAINRTVDADGYRIEITCAACGGHLGHVFKGEGFPTPTNERHCVNSISLTFKAP

>G41095/29-155 CHBRA576g00180

ANMTDVDWKKILTPEQYRVCRRKGTEMAFSGQYWNTKTKGMYLCVCCKTALFRSQTKFDSGTGWPSFYDKVD

LNVKMEMDPFMPRTEVSCAKCNAHLGHVFSDG--PPPTLKRYCINSVALVLKPD

>Azfi_s0301.g063683/77-219

PALSDEEWKKRLTPEQYVITRKKGTERAFSGAYWKTKSPGVYECICCGTPLFDSKTKFDSGTGWPSYFEPIE

NNVKSEMDPFMPRTEVVCSKCNAHLGHVFNDG--PPPTGKRYCINRLLVLYLEI

>Azfi_s0114.g046037/104-229

VQRSEQEWRAILAPEQFRILRQKGTEYPGTGKYNKVYDEGIYECAGCGTPLYKSITKFDSGCGWPAFYEGLP

GAINETVDADGYRIEITCAACGGHLGHVFKGEGFPTPTNERHCVNSISLKFIPA

>Azfi_s0383.g067445/2-133

TEDMLHEMRCLMFMIQYTMIHKMQVLCLGVAEDISHEMEGVYLCAGCETPLYKSTTKFDSGCGWPAFFDGLP

GAINRTVDADGRRIEITCAACGGHLGHVFKGELFPTPTNERHCVNSVSLKFTPA

>Sacu_v1.1_s0042.g012768/49-171

PALTDEEWKKRLTPEQYTITRKKGTERAFTGEYWNTKTPGLYECRCCGTPLFDSRTKFDSGTGWPSYYEPIA

DNVKSEMDPFMPRTEVLCAKCDAHLGHVFNDG--PPPTGKRYCINR----YRVA

>Sacu_v1.1_s1799.g028179/352-477

SMTDDMDWKDKLTPEQYRVLREKGTERPFTGEYVDYHGDGSFKCVGCGNMLFSSDTKFDSGTGWPSFEEAIP

GSVAYHVDHGMVRTEVTCAKCGSHLGHVFEDG--PGASGKRYCINSVCLKLEKP

>Sacu_v1.1_s0062.g015489/51-211

VQKSDEEWRAVLSPEQFRILRQKGTEWAGTGQYDKFFEEGVYGCAGCGTPLYKSTTKFNSGCGWPAFYEGLP

GAINENVDADGYRIEITCAACGGHLGHVFKGEGFPTPTNERHCVNSVSLKFTPA

>92187_Selmo/3-121

KSVSDTEWKEKLTDEQFYVTRQKGTERAFSGKYWNTKTAGIYECICCGTPLFNSITKFDSGTGWPSYYEAIG

NNVKSESDPFMPRTEVKCSKCDAHLGHVFDDG--PPPTGKRYCINR--------

>170312_Selmo/6-131

GNKSEEEWRAILSPEQFRVLRRKGTEFAGTGIYNKHFEGGVYECAGCGTPLYKSDTKFDSGCGWPAFFQGLP

GAINRNADADGRRVEITCAACGGHLGHVFKGEGYRTPTDERHCVNSVSLKFTPG

>98474_Selmo/7-132

VNKTEEEWRAVLNSEQFRILRMKGTEMPGSGEYNKFYKDGVYNCGGCGTPLYKSTAKFDSGCGWPAFFEGLP

GAINRTVDADGYRTEITCAACGGHLGHVFKGEGFPTPTNERHCVNSISLKFAPG

>18637_Selmo/3-98

-QRREEEWRAVLNPEQ--TLRIK---RTGTGKHN---EDGVHNCAGCGTPLYKSTT--DSDC----IYEGLP

GAINRTVDAEGHRIEITCAACGRHLGHVFNGEGFPTPTD---------------

>18636_Selmo/3-98

-QRREEEWRAVLNPEQ--TLRIK---RTGTGKHN---EDGVHNCAGCGTPLYKSTT--DSDC----IYEGLP

GAINRTVDAEGHRIEITCAACGRHLGHVFNGEGFPTPTD---------------

>AaBonn_Sc2ySwM_344.1824.2/25-143

KQISDEEWKKKLSKEQYNVARQKGTERAFTGQYWNTKTPGTYHCVCCDTPLFQSSTKFDSGTGWPSYWEYVS

DNVKSESDPFMPRTEVMCAVCDAHLGHVFNDG--PPPTKKRYCINR--------

>AaBonn_Sc2ySwM_117.2635.1_manually_corrected/1-124

--MSDSEWQRCLSPMQYKVLRQKVTERPFTGEYYSHYESGTYKCVGCNNSLYSSSAKFDSGCGWPVFYEALD

GAIETQTDSDGRRTEIVCANCNGHLGHLFTGEGFPTPTDERHCVNSICLKFCPE

>AaBonn_Sc2ySwM_344.851.2/130-255

VQKSDEEWRAVLSPEQFRILRQKGTEYPGTGKYNKHKEDGVYECAGCGTPLYKSTTKFDSGCGWPAFFEGLP

GAITETRDPDGRRIEITCTACGGHLGHVFKGEGFKTPTDARHCVNSVSLTFTPA

>Bradi1g41830.3/64-190 MSRB1

ASMSDEDLKERLTKEQYYVTRQKGTERAFTGEYWNTKTPGIYHCICCDTPLFESSTKFDSGTGWPSYYRPVG

DNVKNKLDIFMPRTEALCAVCDAHLGHVFDDG--PPPTGKRYCINSASLKLKPQ

>Bradi2g25820.1/92-217 MSRB2

VQKSEEEWEAVLTPEQFRILRRKGTEYPGTGEYDKFFDEGIYGCAGCGTPLYKSSTKFNSGCGWPAFYEGFP

GAIKRTADPDGRRVEITCAACDGHLGHVFKGEGFNTPTDERHCVNSISLKFIPA

>Bradi1g61800.1/7-132 MSRB3

KPKTEEEWRAVLSPEQFRILRLKDTELPGTGEYNKFYGSGVYNCAGCGTPLYKSTTKFDSGCGWPAFFEGLP

GAIQRTPDPDGRRVEITCTACGGHLGHVFKGEGFKTPTDERHCVNSVSMKFTPA

**PRXIIE**

cov pid  **1** **[ . . . . : . . .** **80**

1 28066_Selmo 100.0% 100.0% **-----------IAVGERIPDGELSYFDE-SGAIQSIKVSDLTSKKKVVIFAVPGAFTPTCSSKHVPGFIDKADELKSKGV**

2 AT3G52960_PrxIIE 100.0% 60.1% **RSFATTPVTASISVGDKLPDSTLSYLDPSTGDVKTVTVSSLTAGKKTILFAVPGAFTPTCSQKHVPGFVSKAGELRSKGI**

3 AaBonn_Sc2ySwM_228_3611_1 100.0% 66.3% **SVPATAAPSRSISVGEKLPEAELSYFDQ-EGNIQVVKVSELTKGKKVVLFAVPGAFTPTCSQKHLPGFLEKADELRAKGV**

4 Azfi_s0233_g059382 100.0% 62.2% **VSTETSVESKTISVGDKLPEATFAYLDK-EGQVQTVTVSDLTKGKKAVFFAVPGAFTPTCSQKHLPGFVEKADELRSKGV**

5 Bradi1g35660 100.0% 64.2% **AAASPTTAAATIAVGDRLPDATLSYFDSPDGELKTVTVSDLTAGKKVVLFAVPGAFTPTCTQKHLPGFVAKAGELRAKGV**

6 Bradi3g06750 100.0% 61.8% **AAATASPVVATIAVGDKLPDATLSYFDPADGELKTVTVGELTAGKKAVLFAVPGAFTPTCSQKHLPGFVAAAGDLRAKGV**

7 G23695_CHBRA288g00020 98.8% 55.8% **SSAPSATKAKTIAVGDSIPNVGLQYFDE-EGVMQTVMTGDLAKGKKIVLFAVPGAFTPTCSQKHLPAFVEKADELNAKGV**

8 Mapoly0147s0023 100.0% 56.6% **SFSLSTSIKATIQVGEKLPEAELSYLDQ-ENNVQTVKISELTKGKKIVLFAVPGAFTPTCSQKHVPGFVDKSDELKSKGV**

9 Pp3c13_7010V3_PpPrxIIA 100.0% 64.5% **SRSGAGQVVATISVGDKLPEAQLSYFDK-DGNVQSVSVSELTKGKKVVLFAVPGAFTPTCSSKHLPGFVAKADELRKAGV**

10 Pp3c3_490V3_PpPrxIIB 100.0% 63.4% **SRSGVTQVMATISVGDKLPEANLSYFDT-EGNVQSVSVSELTRGKKVVLFAVPGAFTPTCSSKHLPGFVANAEELRKAGV**

11 Pp3c3_500V3 100.0% 59.9% **LPTFGSRAATTIAEGSKLPDAELSYFDK-EGNVNIVKVSDLMRAKKVVLFAVPGAFTPTCSTQHLPGFVAKADKLRKAGA**

12 Sacu_s0011_g005025 100.0% 60.5% **TASLSTEASSPISLGDKLPESTFAYLDK-EGQVQTVSVSDLTKGKKAVLFAVPGAFTPTCSQKHLPGFVEKAEELRAKGV**

13 AT1G65980_PrxIIB_outgroup 98.8% 58.5% **--------MAPIAVGDVVPDGTISFFDE-NDQLQTASVHSLAAGKKVILFGVPGAFTPTCSMKHVPGFIEKAEELKSKGV**

cov pid  **81**  **. 1 . . . . : .** **160**

1 28066_Selmo 100.0% 100.0% **DTIACVSVNDAFVMKSWGEALGVNGKILMLSDGNGKFTRDLGVTVDLSDKVEGLGVRSRRYSLLAEDGIVKVLNLEEGGA**

2 AT3G52960_PrxIIE 100.0% 60.1% **DVIACISVNDAFVMEAWRKDLGINDEVMLLSDGNGEFTGKLGVELDLRDKPVGLGVRSRRYAILADDGVVKVLNLEEGGA**

3 AaBonn_Sc2ySwM_228_3611_1 100.0% 66.3% **DTIACVSVNDTFVLRAWGESVGVGDKVLLLSDGNAVFTKALGVSVDLSDKPALLGIRSRRYALLADDGVVKVLNLEEGGA**

4 Azfi_s0233_g059382 100.0% 62.2% **DTIACISVNDVFVMRAWGENLSTGDKVLLLSDGNLNFTKAIGATLDLSDKPVGLGVRSRRYSLLAEDGVVRVLHLEEGGS**

5 Bradi1g35660 100.0% 64.2% **DTVACVSVNDAFVMRAWKDSLGVGDEVLLLSDGNGELTRAMGVELDLSDKPVGLGVRSRRYALLAEDGVVKVLNLEEGGS**

6 Bradi3g06750 100.0% 61.8% **DTVACVSVNDAFVMKAWKESLGLGDDVMMLSDGNLELTRALGVEMDLSDKPMGLGVRSRRYALLADDGVVKVLNLEEGGA**

7 G23695_CHBRA288g00020 98.8% 55.8% **SVIACVSVNDPFVMQAWGKNVGVSDKVMLLADGSGQFTQAMGVELDLIDR--GLGVRSRRYAMLVDDGVVKICNLEEGGA**

8 Mapoly0147s0023 100.0% 56.6% **DTIACVSVNDVFVMKAWGEGLGVGDKVLLLSDGNGHFTKALGVELDLSDKPVGLGVRSRRYALLADDGVVKQLNLEEGGA**

9 Pp3c13_7010V3_PpPrxIIA 100.0% 64.5% **DTLACVSVNDAFVMQAWGKSAGVGDSVLMLSDGLAKFTQALGTAVDLTDKVEGLGIRSRRYSMLVEDGVVKVLNLEVGGA**

10 Pp3c3_490V3_PpPrxIIB 100.0% 63.4% **DTLACVSVNDAFVMQAWAKSVGAGDKVLMLSDGLAKFTQALGTTVDLTDKVEGLGIRSRRYSMLVDDGVVKVLNLEEGGA**

11 Pp3c3_500V3 100.0% 59.9% **DLLACVSVNDAFVMRAWGENQNVGESVLLLSDGLGKFTHAMGASVDLSDKPVGLGVRSRRYAMLVDDGVVKTLHMEEGGA**

12 Sacu_s0011_g005025 100.0% 60.5% **DTIACISVNDVFVMRAWGESIGVGDKVLLLSDGNLNFTKAIGVTLDLTDKPVGLGVRSRRYSILAEDGVIKVLHLEEGGS**

13 AT1G65980_PrxIIB_outgroup 98.8% 58.5% **DEIICFSVNDPFVMKAWGKTYPENKHVKFVADGSGEYTHLLGLELDLKDK--GLGVRSRRFALLLDDLKVTVANVESGGE**

cov pid **161**  **. . . 2 ]** **206**

1 28066_Selmo 100.0% 100.0% **YTVSSADEILKAL---------------------------------**

2 AT3G52960_PrxIIE 100.0% 60.1% **FTNSSAEDMLKAL---------------------------------**

3 AaBonn_Sc2ySwM_228_3611_1 100.0% 66.3% **FTISGPEEILKAL---------------------------------**

4 Azfi_s0233_g059382 100.0% 62.2% **FTTSGADDILKAL---------------------------------**

5 Bradi1g35660 100.0% 64.2% **FTNSSAEDMLKAL---------------------------------**

6 Bradi3g06750 100.0% 61.8% **FTTSSAEEMLKVL---------------------------------**

7 G23695_CHBRA288g00020 98.8% 55.8% **FTNSGPESILEAL---------------------------------**

8 Mapoly0147s0023 100.0% 56.6% **FSISGPDEILKALLKCSYAYGAAGFPEWGIQPNSTLLFEIEVLKIV**

9 Pp3c13_7010V3_PpPrxIIA 100.0% 64.5% **FTNSSAEEILSSL---------------------------------**

10 Pp3c3_490V3_PpPrxIIB 100.0% 63.4% **FTSSSAEEILSSL---------------------------------**

11 Pp3c3_500V3 100.0% 59.9% **FTSSGADDILKAL---------------------------------**

12 Sacu_s0011_g005025 100.0% 60.5% **FTTSGADDILKAL---------------------------------**

13 AT1G65980_PrxIIB_outgroup 98.8% 58.5% **FTVSSADDILKAL---------------------------------**

>28066_Selmo

-----------IAVGERIPDGELSYFDE-SGAIQSIKVSDLTSKKKVVIFAVPGAFTPTCSSKHVPGFIDKA

DELKSKGVDTIACVSVNDAFVMKSWGEALGVNGKILMLSDGNGKFTRDLGVTVDLSDKVEGLGVRSRRYSLL

AEDGIVKVLNLEEGGAYTVSSADEILKAL---------------------------------

>AT3G52960_PrxIIE

RSFATTPVTASISVGDKLPDSTLSYLDPSTGDVKTVTVSSLTAGKKTILFAVPGAFTPTCSQKHVPGFVSKA

GELRSKGIDVIACISVNDAFVMEAWRKDLGINDEVMLLSDGNGEFTGKLGVELDLRDKPVGLGVRSRRYAIL

ADDGVVKVLNLEEGGAFTNSSAEDMLKAL---------------------------------

>AaBonn_Sc2ySwM_228_3611_1

SVPATAAPSRSISVGEKLPEAELSYFDQ-EGNIQVVKVSELTKGKKVVLFAVPGAFTPTCSQKHLPGFLEKA

DELRAKGVDTIACVSVNDTFVLRAWGESVGVGDKVLLLSDGNAVFTKALGVSVDLSDKPALLGIRSRRYALL

ADDGVVKVLNLEEGGAFTISGPEEILKAL---------------------------------

>Azfi_s0233_g059382

VSTETSVESKTISVGDKLPEATFAYLDK-EGQVQTVTVSDLTKGKKAVFFAVPGAFTPTCSQKHLPGFVEKA

DELRSKGVDTIACISVNDVFVMRAWGENLSTGDKVLLLSDGNLNFTKAIGATLDLSDKPVGLGVRSRRYSLL

AEDGVVRVLHLEEGGSFTTSGADDILKAL---------------------------------

>Bradi1g35660

AAASPTTAAATIAVGDRLPDATLSYFDSPDGELKTVTVSDLTAGKKVVLFAVPGAFTPTCTQKHLPGFVAKA

GELRAKGVDTVACVSVNDAFVMRAWKDSLGVGDEVLLLSDGNGELTRAMGVELDLSDKPVGLGVRSRRYALL

AEDGVVKVLNLEEGGSFTNSSAEDMLKAL---------------------------------

>Bradi3g06750

AAATASPVVATIAVGDKLPDATLSYFDPADGELKTVTVGELTAGKKAVLFAVPGAFTPTCSQKHLPGFVAAA

GDLRAKGVDTVACVSVNDAFVMKAWKESLGLGDDVMMLSDGNLELTRALGVEMDLSDKPMGLGVRSRRYALL

ADDGVVKVLNLEEGGAFTTSSAEEMLKVL---------------------------------

>G23695_CHBRA288g00020

SSAPSATKAKTIAVGDSIPNVGLQYFDE-EGVMQTVMTGDLAKGKKIVLFAVPGAFTPTCSQKHLPAFVEKA

DELNAKGVSVIACVSVNDPFVMQAWGKNVGVSDKVMLLADGSGQFTQAMGVELDLIDR--GLGVRSRRYAML

VDDGVVKICNLEEGGAFTNSGPESILEAL---------------------------------

>Mapoly0147s0023

SFSLSTSIKATIQVGEKLPEAELSYLDQ-ENNVQTVKISELTKGKKIVLFAVPGAFTPTCSQKHVPGFVDKS

DELKSKGVDTIACVSVNDVFVMKAWGEGLGVGDKVLLLSDGNGHFTKALGVELDLSDKPVGLGVRSRRYALL

ADDGVVKQLNLEEGGAFSISGPDEILKALLKCSYAYGAAGFPEWGIQPNSTLLFEIEVLKIV

>Pp3c13_7010V3_PpPrxIIA

SRSGAGQVVATISVGDKLPEAQLSYFDK-DGNVQSVSVSELTKGKKVVLFAVPGAFTPTCSSKHLPGFVAKA

DELRKAGVDTLACVSVNDAFVMQAWGKSAGVGDSVLMLSDGLAKFTQALGTAVDLTDKVEGLGIRSRRYSML

VEDGVVKVLNLEVGGAFTNSSAEEILSSL---------------------------------

>Pp3c3_490V3_PpPrxIIB

SRSGVTQVMATISVGDKLPEANLSYFDT-EGNVQSVSVSELTRGKKVVLFAVPGAFTPTCSSKHLPGFVANA

EELRKAGVDTLACVSVNDAFVMQAWAKSVGAGDKVLMLSDGLAKFTQALGTTVDLTDKVEGLGIRSRRYSML

VDDGVVKVLNLEEGGAFTSSSAEEILSSL---------------------------------

>Pp3c3_500V3

LPTFGSRAATTIAEGSKLPDAELSYFDK-EGNVNIVKVSDLMRAKKVVLFAVPGAFTPTCSTQHLPGFVAKA

DKLRKAGADLLACVSVNDAFVMRAWGENQNVGESVLLLSDGLGKFTHAMGASVDLSDKPVGLGVRSRRYAML

VDDGVVKTLHMEEGGAFTSSGADDILKAL---------------------------------

>Sacu_s0011_g005025

TASLSTEASSPISLGDKLPESTFAYLDK-EGQVQTVSVSDLTKGKKAVLFAVPGAFTPTCSQKHLPGFVEKA

EELRAKGVDTIACISVNDVFVMRAWGESIGVGDKVLLLSDGNLNFTKAIGVTLDLTDKPVGLGVRSRRYSIL

AEDGVIKVLHLEEGGSFTTSGADDILKAL---------------------------------

>AT1G65980_PrxIIB_outgroup

--------MAPIAVGDVVPDGTISFFDE-NDQLQTASVHSLAAGKKVILFGVPGAFTPTCSMKHVPGFIEKA

EELKSKGVDEIICFSVNDPFVMKAWGKTYPENKHVKFVADGSGEYTHLLGLELDLKDK--GLGVRSRRFALL

LDDLKVTVANVESGGEFTVSSADDILKAL---------------------------------

**GRX “CPYC” (Class 1)**

cov pid  **1** **[ . . . . : . . .** **80**

1 Sacu_v1.1_s0167.g024192/93-192 100.0% 100.0% **HLKENISSNPVVVYSKSYCPYCMRVKDLFKELGVKPFVIELDELSDGNEVQTALQKLTGQYTVPNVFIGGKHIGGCDDTV**

2 Sacu_v1.1_s0073.g017170b_manually_corrected/41-141 100.0% 51.1% **FVQRVVHDNKIAIFSKSYCPYCKRAKGVFNELKEKPFVVELDLRDDGADIQKAVSDLVGRRTVPQVFVHGKHLGGSDDTV**

3 Sacu_v1.1_s0009.g004405/5-102 100.0% 68.5% **KAKDLVSSNPVVVFSKSYCPYCINVKQLLSSLGVKSKVIELDQQDDGPEMQAALAEWTGQRTVPNVFIGGKHIGGCDTTV**

4 PpGrxC5_Pp3c3_7440V1.1/130-219 97.8% 65.9% **WIKKKNSSEPVVVYSKTYCPYCMRVKKLFSTLGYDFEVIELDAGGQLG-LQDALERVSGQYTVPNVFIGGKHIGGCDDTV**

5 PpGrxC3_Pp3c18_5670V1.1/65-172 100.0% 54.3% **FVKKTLAEHPLVIFSKSYCPYCKRAKSVFESMSVKPFVLELDEREDGDDIQQALGKFVGRRTVPQVFINGVHLGGSDDTV**

6 PpGrxC2_Pp3c9_24380V3.2/108-209 100.0% 62.0% **KAQALISQNAVVVFSKSYCPFCLRVKSLLKSIGAEMKVVELDEESDGSDIQAALAKLSGQRTVPNVFIGGQHIGGRDDTT**

7 PpGrxC1_Pp3c7_9000V3.1/5-139 100.0% 52.2% **KVQELIEQNPLIVFSKSKCPFCKTVKELFKSLEVEPRVVEIDLEKDGGAIQKALFQTSKQLTVPNVFIGGEHIGGNDAVK**

8 Pp3c2_23170V1.1_no_UTRs,no_ESTs/5-128 72.8% 38.0% **KVQELILQNPLIIFSKSYCPYCRNVKELLKGLGAEAKVVELDRESE-------------------------HIGGNDATK**

9 Mapoly0182s0020.1/1-102 100.0% 65.2% **MICSKNSENAVVVYSKSWCPYCGRVKSLFRELGVEFLLIELDNLVEEQEVQEALRRLTRQSTVPNIFIGGKHIGGCDDTM**

10 Mapoly0078s0047.1/5-102 100.0% 59.8% **KAQTLVSENAVVVFSKSYCPYCIKVKQLLSSLGAKMKVVELDDEKDGDEIQSALAKWTKQRTVPNVFVGGQHIGGCDDTV**

11 Mapoly0078s0046.1/5-102 100.0% 57.6% **SAQNLVSQNAVVVFSKSYCPYCKKVKQLLSSLGAQVKVVELDLEKDGDEIQSALTNWTKQRTVPNVFVGGQHIGGCDDTM**

12 Mapoly0004s0084.1/46-150 100.0% 50.0% **FVKKTIAAHPIVIFSKSYCPYCKRAKSVFKQMDTTPYVVELDLREDGGRIQAALSELVGRRTVPQVFVDGNHIGGSDDTL**

13 CHBRA70g00580/177-262 93.5% 57.0% **LIAEKNAQNPVVVYSKTWCPYCAAVKGLFTKLGVEFKLVELDELVGEEDWQYALSQLTGQRTVPSVFVGGEHIGGCDSTM**

14 CHBRA403g00260/6-108 100.0% 57.6% **KVKELVESNGLVVFSKTWCSYCIRVRDLFNEIGAKGKFVQLDEEEDGEDMQFALLEWTGQRTVPNVFIGGEHVGGCDDTV**

15 CHBRA222g00290/30-136 100.0% 53.3% **FVKTTIASNPLVIFSKSYCPYCKRAKNVFKELKETPYVVELDLRDDGGSIQEEIGKMHGVWTVPQVFIGGQRLGGSDDTV**

16 Bradi5g15220.1/20-128 100.0% 64.1% **KAKEIVASAPVVVFSKSYCPFCVKVKQLFTQLGASFKAIELDKESDGAEMQSALAEWTGQRTVPNVFINGKHIGGCDDTV**

17 Bradi3g50172.1/29-128 100.0% 47.8% **DVQNAIYSNRITIFSKSYCPYCMRAKRIFRDLKENPYVVELDLREDGQEIQSVLLDLVGRNTVPQVFVNGHHVGGSDDTK**

18 Bradi3g12720.1/66-168 100.0% 59.8% **SVKKTLADNPVVIYSKSWCSYSMEVKGLFKRIGVQPHVIELDHLAQGPQLQKVLERLTGQSTVPNVFIGGKHIGGCTDTV**

19 Bradi1g31450.1/36-137 100.0% 52.2% **FVKSTVKAHDVVIFSKSYCPYCRRAKAVFKELQLKPYVVELDQREDGGEIQDALSDMVGRRTVPQVFVRGKHLGGSDDTV**

20 Azfi_s0270.g061184/38-136 100.0% 57.6% **FVKRVIQENKIVVFSKSYCPYCKRAKAVFNELKEKPFVIELDERDDGSAIQRAVSDLVGRRTVPQVFIHGKHLGGSDDTV**

21 Azfi_s0158.g053892/24-121 100.0% 63.0% **KAQDLVSSNPVMVFSKSYCPYCTRVKQLLASIGAKSKVIELDQESDGSDLQSALAQWTGLRTVPNVFIGGTHIGGCDTTV**

22 Azfi_s0074.g037496_manually_corrected/6-103 100.0% 64.1% **KVKDLVSSNPLIVFSKSYCPYCARVKQLFSSLGAKSKVIELDQENDGSELQSALAEWTGQRSVPNVFIGGNHIGGCDTTV**

23 Azfi_s0004.g008860/50-149 100.0% 52.2% **VVKEAIEQHKIVIFSKSYCPYCKRAKAIFSELNEKPFVIELDQREDGYDIQKAVINLVARRTVPQVFIHKQHLGGADDTL**

24 AtGrxS12_At2g20270/77-206 100.0% 59.8% **TVKTTVAENPVVVYSKTWCSYSSQVKSLFKSLQVEPLVVELDQLSEGSQLQNVLEKITGQYTVPNVFIGGKHIGGCSDTL**

25 AtGrxC5_AT4G28730/72-174 100.0% 54.3% **SIRKTVTENTVVIYSKTWCSYCTEVKTLFKRLGVQPLVVELDQLPQGPQLQKVLERLTGQHTVPNVFVCGKHIGGCTDTV**

26 AtGrxC4_AT5G20500/34-135 100.0% 53.3% **FVKKTISSHKIVIFSKSYCPYCKKAKSVFRELDQVPYVVELDEREDGWSIQTALGEIVGRRTVPQVFINGKHLGGSDDTV**

27 AtGrxC3_AT1G77370/36-130 100.0% 48.9% **FVQNAILSNKIVIFSKSYCPYCLRSKRIFSQLKEEPFVVELDQREDGDQIQYELLEFVGRRTVPQVFVNGKHIGGSDDLG**

28 AtGrxC2_AT5G40370/21-136 100.0% 55.4% **YLDAFVYQWMILSYSKTYCPYCVRVKELLQQLGAKFKAVELDTESDGSQIQSGLAEWTGQRTVPNVFIGGNHIGGCDATS**

29 AtGrxC1_AT5G63030/21-125 100.0% 58.7% **KAKEIVSAYPVVVFSKTYCGYCQRVKQLLTQLGATFKVLELDEMSDGGEIQSALSEWTGQTTVPNVFIKGNHIGGCDRVM**

30 AaBonn_Sc2ySwM_344_5317_1/84-189 100.0% 57.6% **KVQSIVEKDPLVVFSKSYCPFCKRVKKLFSDLGATGSVVELDEEPDGDAIQNALASWTKQRTVPNVFIGGKHVGGCDDTV**

31 AaBonn_Sc2ySwM_228_4790_1/123-213 98.9% 62.6% **LIKSKNELNPVVVYSKTWCPYCGQVKSLFKKLNVSFVLVELDELVEERDVQAALRRISGQSTVPNVFIGGKHIGGCDDTM**

32 89111_Selmo/5-103 100.0% 52.2% **KAKDIVAHNPLVVFSKTYCPFCVKVKELFSSIGAQPKVVELDSEADGADLQAALAEWTGQRSVPSVFVGGKHVGGCDDTT**

33 229902_Selmo/3-103 100.0% 59.8% **KAKDLVASNPVMVFSKSYCPYCVSVKKLLASLGAKFTALELNAEKDGAEIQAALAEWTGQRTVPSVFIGGKHIGGCDDTT**

34 19369_Selmo/1-89 91.3% 70.2% **--------NPVVIYSKSWCPYCSKVKGLFKKLGVKVVVVELDELVEEADVQAALKRMTGQSTVPNVFIGGKHVGGCDDTH**

35 174566_Selmo/31-131 100.0% 53.3% **FVKSTIDNHDIVIFSKSYCPYCRRAKSVFKSLNETPHVVELDLREDGDEIQEALQGLVGRRTVPQVFVGGKHIGGSDDTV**

36 CHBRA15g00780_corrected/7-240 100.0% 54.3% **QVKAMVQTEPVLLFSKTYCPYCGRVKKLLRDLGVKATVVELDERDDGETLQRALEELTGQSTVPSLFVGGKHIGGCEETM**

37 AT3G54900_GRXS14/74-173 96.7% 23.9% **TLEKLVNSEKVVLFTRDFCGFSNTVVQILKNLNVPFEDVNI---LENEMLRQGLKEYSNWPTFPQLYIGGEFFGGCDITL**

cov pid **81**  **. ]** **92**

1 Sacu_v1.1_s0167.g024192/93-192 100.0% 100.0% **ALHQRGKLIPLL**

2 Sacu_v1.1_s0073.g017170b_manually_corrected/41-141 100.0% 51.1% **EAYQSGKLAELL**

3 Sacu_v1.1_s0009.g004405/5-102 100.0% 68.5% **AKHREGKLLPLL**

4 PpGrxC5_Pp3c3_7440V1.1/130-219 97.8% 65.9% **ALHSKGQLEPL-**

5 PpGrxC3_Pp3c18_5670V1.1/65-172 100.0% 54.3% **AAQQSGRLKKLL**

6 PpGrxC2_Pp3c9_24380V3.2/108-209 100.0% 62.0% **AMHKKGQLLPLL**

7 PpGrxC1_Pp3c7_9000V3.1/5-139 100.0% 52.2% **ALHSKGELVVKL**

8 Pp3c2_23170V1.1_no_UTRs,no_ESTs/5-128 72.8% 38.0% **AAHKKGTLHPKL**

9 Mapoly0182s0020.1/1-102 100.0% 65.2% **DLHRRGQLMPLL**

10 Mapoly0078s0047.1/5-102 100.0% 59.8% **SKHNGGKLLPML**

11 Mapoly0078s0046.1/5-102 100.0% 57.6% **SKHNGGKLLPLL**

12 Mapoly0004s0084.1/46-150 100.0% 50.0% **EAFNNGKLKKLL**

13 CHBRA70g00580/177-262 93.5% 57.0% **ELHESG------**

14 CHBRA403g00260/6-108 100.0% 57.6% **RKHRREELIPML**

15 CHBRA222g00290/30-136 100.0% 53.3% **AAYSSGKLQKML**

16 Bradi5g15220.1/20-128 100.0% 64.1% **ALNNGGKLVALL**

17 Bradi3g50172.1/29-128 100.0% 47.8% **EALSNGQLHKLL**

18 Bradi3g12720.1/66-168 100.0% 59.8% **KLYRKGELATML**

19 Bradi1g31450.1/36-137 100.0% 52.2% **DAYESGELAKLL**

20 Azfi_s0270.g061184/38-136 100.0% 57.6% **EAYQSGKLVELI**

21 Azfi_s0158.g053892/24-121 100.0% 63.0% **AKHNAGKLVPLL**

22 Azfi_s0074.g037496_manually_corrected/6-103 100.0% 64.1% **AKHEGGQLLPLL**

23 Azfi_s0004.g008860/50-149 100.0% 52.2% **EAYQSGKLAKLL**

24 AtGrxS12_At2g20270/77-206 100.0% 59.8% **QLHNKGELEAIL**

25 AtGrxC5_AT4G28730/72-174 100.0% 54.3% **KLNRKGDLELML**

26 AtGrxC4_AT5G20500/34-135 100.0% 53.3% **DAYESGELAKLL**

27 AtGrxC3_AT1G77370/36-130 100.0% 48.9% **AALESGQLQKLL**

28 AtGrxC2_AT5G40370/21-136 100.0% 55.4% **NLHKDGKLVPLL**

29 AtGrxC1_AT5G63030/21-125 100.0% 58.7% **ETNKQGKLVPLL**

30 AaBonn_Sc2ySwM_344_5317_1/84-189 100.0% 57.6% **LLQKQGKLVPML**

31 AaBonn_Sc2ySwM_228_4790_1/123-213 98.9% 62.6% **ALHSKGQLIPL-**

32 89111_Selmo/5-103 100.0% 52.2% **KKHNSGQLVPML**

33 229902_Selmo/3-103 100.0% 59.8% **ATHRKGQLVPLL**

34 19369_Selmo/1-89 91.3% 70.2% **RLHSQGKLIPML**

35 174566_Selmo/31-131 100.0% 53.3% **EAHESGRLETII**

36 CHBRA15g00780_corrected/7-240 100.0% 54.3% **AAHENGYLTRAL**

37 AT3G54900_GRXS14/74-173 96.7% 23.9% **EAFKTGELQEEV**

>Sacu_v1.1_s0167.g024192/93-192

HLKENISSNPVVVYSKSYCPYCMRVKDLFKELGVKPFVIELDELSDGNEVQTALQKLTGQYTVPNVFIGGKH

IGGCDDTVALHQRGKLIPLL

>Sacu_v1.1_s0073.g017170b_manually_corrected/41-141

FVQRVVHDNKIAIFSKSYCPYCKRAKGVFNELKEKPFVVELDLRDDGADIQKAVSDLVGRRTVPQVFVHGKH

LGGSDDTVEAYQSGKLAELL

>Sacu_v1.1_s0009.g004405/5-102

KAKDLVSSNPVVVFSKSYCPYCINVKQLLSSLGVKSKVIELDQQDDGPEMQAALAEWTGQRTVPNVFIGGKH

IGGCDTTVAKHREGKLLPLL

>PpGrxC5_Pp3c3_7440V1.1/130-219

WIKKKNSSEPVVVYSKTYCPYCMRVKKLFSTLGYDFEVIELDAGGQLG-LQDALERVSGQYTVPNVFIGGKH

IGGCDDTVALHSKGQLEPL-

>PpGrxC3_Pp3c18_5670V1.1/65-172

FVKKTLAEHPLVIFSKSYCPYCKRAKSVFESMSVKPFVLELDEREDGDDIQQALGKFVGRRTVPQVFINGVH

LGGSDDTVAAQQSGRLKKLL

>PpGrxC2_Pp3c9_24380V3.2/108-209

KAQALISQNAVVVFSKSYCPFCLRVKSLLKSIGAEMKVVELDEESDGSDIQAALAKLSGQRTVPNVFIGGQH

IGGRDDTTAMHKKGQLLPLL

>PpGrxC1_Pp3c7_9000V3.1/5-139

KVQELIEQNPLIVFSKSKCPFCKTVKELFKSLEVEPRVVEIDLEKDGGAIQKALFQTSKQLTVPNVFIGGEH

IGGNDAVKALHSKGELVVKL

>Pp3c2_23170V1.1_no_UTRs,no_ESTs/5-128

KVQELILQNPLIIFSKSYCPYCRNVKELLKGLGAEAKVVELDRESE-------------------------H

IGGNDATKAAHKKGTLHPKL

>Mapoly0182s0020.1/1-102

MICSKNSENAVVVYSKSWCPYCGRVKSLFRELGVEFLLIELDNLVEEQEVQEALRRLTRQSTVPNIFIGGKH

IGGCDDTMDLHRRGQLMPLL

>Mapoly0078s0047.1/5-102

KAQTLVSENAVVVFSKSYCPYCIKVKQLLSSLGAKMKVVELDDEKDGDEIQSALAKWTKQRTVPNVFVGGQH

IGGCDDTVSKHNGGKLLPML

>Mapoly0078s0046.1/5-102

SAQNLVSQNAVVVFSKSYCPYCKKVKQLLSSLGAQVKVVELDLEKDGDEIQSALTNWTKQRTVPNVFVGGQH

IGGCDDTMSKHNGGKLLPLL

>Mapoly0004s0084.1/46-150

FVKKTIAAHPIVIFSKSYCPYCKRAKSVFKQMDTTPYVVELDLREDGGRIQAALSELVGRRTVPQVFVDGNH

IGGSDDTLEAFNNGKLKKLL

>CHBRA70g00580/177-262

LIAEKNAQNPVVVYSKTWCPYCAAVKGLFTKLGVEFKLVELDELVGEEDWQYALSQLTGQRTVPSVFVGGEH

IGGCDSTMELHESG------

>CHBRA403g00260/6-108

KVKELVESNGLVVFSKTWCSYCIRVRDLFNEIGAKGKFVQLDEEEDGEDMQFALLEWTGQRTVPNVFIGGEH

VGGCDDTVRKHRREELIPML

>CHBRA222g00290/30-136

FVKTTIASNPLVIFSKSYCPYCKRAKNVFKELKETPYVVELDLRDDGGSIQEEIGKMHGVWTVPQVFIGGQR

LGGSDDTVAAYSSGKLQKML

>Bradi5g15220.1/20-128

KAKEIVASAPVVVFSKSYCPFCVKVKQLFTQLGASFKAIELDKESDGAEMQSALAEWTGQRTVPNVFINGKH

IGGCDDTVALNNGGKLVALL

>Bradi3g50172.1/29-128

DVQNAIYSNRITIFSKSYCPYCMRAKRIFRDLKENPYVVELDLREDGQEIQSVLLDLVGRNTVPQVFVNGHH

VGGSDDTKEALSNGQLHKLL

>Bradi3g12720.1/66-168

SVKKTLADNPVVIYSKSWCSYSMEVKGLFKRIGVQPHVIELDHLAQGPQLQKVLERLTGQSTVPNVFIGGKH

IGGCTDTVKLYRKGELATML

>Bradi1g31450.1/36-137

FVKSTVKAHDVVIFSKSYCPYCRRAKAVFKELQLKPYVVELDQREDGGEIQDALSDMVGRRTVPQVFVRGKH

LGGSDDTVDAYESGELAKLL

>Azfi_s0270.g061184/38-136

FVKRVIQENKIVVFSKSYCPYCKRAKAVFNELKEKPFVIELDERDDGSAIQRAVSDLVGRRTVPQVFIHGKH

LGGSDDTVEAYQSGKLVELI

>Azfi_s0158.g053892/24-121

KAQDLVSSNPVMVFSKSYCPYCTRVKQLLASIGAKSKVIELDQESDGSDLQSALAQWTGLRTVPNVFIGGTH

IGGCDTTVAKHNAGKLVPLL

>Azfi_s0074.g037496_manually_corrected/6-103

KVKDLVSSNPLIVFSKSYCPYCARVKQLFSSLGAKSKVIELDQENDGSELQSALAEWTGQRSVPNVFIGGNH

IGGCDTTVAKHEGGQLLPLL

>Azfi_s0004.g008860/50-149

VVKEAIEQHKIVIFSKSYCPYCKRAKAIFSELNEKPFVIELDQREDGYDIQKAVINLVARRTVPQVFIHKQH

LGGADDTLEAYQSGKLAKLL

>AtGrxS12_At2g20270/77-206

TVKTTVAENPVVVYSKTWCSYSSQVKSLFKSLQVEPLVVELDQLSEGSQLQNVLEKITGQYTVPNVFIGGKH

IGGCSDTLQLHNKGELEAIL

>AtGrxC5_AT4G28730/72-174

SIRKTVTENTVVIYSKTWCSYCTEVKTLFKRLGVQPLVVELDQLPQGPQLQKVLERLTGQHTVPNVFVCGKH

IGGCTDTVKLNRKGDLELML

>AtGrxC4_AT5G20500/34-135

FVKKTISSHKIVIFSKSYCPYCKKAKSVFRELDQVPYVVELDEREDGWSIQTALGEIVGRRTVPQVFINGKH

LGGSDDTVDAYESGELAKLL

>AtGrxC3_AT1G77370/36-130

FVQNAILSNKIVIFSKSYCPYCLRSKRIFSQLKEEPFVVELDQREDGDQIQYELLEFVGRRTVPQVFVNGKH

IGGSDDLGAALESGQLQKLL

>AtGrxC2_AT5G40370/21-136

YLDAFVYQWMILSYSKTYCPYCVRVKELLQQLGAKFKAVELDTESDGSQIQSGLAEWTGQRTVPNVFIGGNH

IGGCDATSNLHKDGKLVPLL

>AtGrxC1_AT5G63030/21-125

KAKEIVSAYPVVVFSKTYCGYCQRVKQLLTQLGATFKVLELDEMSDGGEIQSALSEWTGQTTVPNVFIKGNH

IGGCDRVMETNKQGKLVPLL

>AaBonn_Sc2ySwM_344_5317_1/84-189

KVQSIVEKDPLVVFSKSYCPFCKRVKKLFSDLGATGSVVELDEEPDGDAIQNALASWTKQRTVPNVFIGGKH

VGGCDDTVLLQKQGKLVPML

>AaBonn_Sc2ySwM_228_4790_1/123-213

LIKSKNELNPVVVYSKTWCPYCGQVKSLFKKLNVSFVLVELDELVEERDVQAALRRISGQSTVPNVFIGGKH

IGGCDDTMALHSKGQLIPL-

>89111_Selmo/5-103

KAKDIVAHNPLVVFSKTYCPFCVKVKELFSSIGAQPKVVELDSEADGADLQAALAEWTGQRSVPSVFVGGKH

VGGCDDTTKKHNSGQLVPML

>229902_Selmo/3-103

KAKDLVASNPVMVFSKSYCPYCVSVKKLLASLGAKFTALELNAEKDGAEIQAALAEWTGQRTVPSVFIGGKH

IGGCDDTTATHRKGQLVPLL

>19369_Selmo/1-89

--------NPVVIYSKSWCPYCSKVKGLFKKLGVKVVVVELDELVEEADVQAALKRMTGQSTVPNVFIGGKH

VGGCDDTHRLHSQGKLIPML

>174566_Selmo/31-131

FVKSTIDNHDIVIFSKSYCPYCRRAKSVFKSLNETPHVVELDLREDGDEIQEALQGLVGRRTVPQVFVGGKH

IGGSDDTVEAHESGRLETII

>CHBRA15g00780_corrected/7-240

QVKAMVQTEPVLLFSKTYCPYCGRVKKLLRDLGVKATVVELDERDDGETLQRALEELTGQSTVPSLFVGGKH

IGGCEETMAAHENGYLTRAL

>AT3G54900_GRXS14/74-173

TLEKLVNSEKVVLFTRDFCGFSNTVVQILKNLNVPFEDVNI---LENEMLRQGLKEYSNWPTFPQLYIGGEF

FGGCDITLEAFKTGELQEEV

**GRX S14 and S16 (Class 2)**

cov pid  **1** **[ . . . . : . . .** **80**

1 G41800_CHBRA601g00040 100.0% 100.0% **NKVVLFMKGSKLFPQCGFSNTCVQILNVLNVPYETVNILEDE---DLRQGMKEYSKWPTFPQLYVDKEFIGGCDIMIELY**

2 102863_Selmo 100.0% 75.0% **HKVVLFMKGTKLFPQCGFSNTVVQILNNLSVPYETVNILEDD---GLRQGLKAYSNWPTFPQLYIDGEFFGGCDITLEAF**

3 Bradi1g01570 100.0% 68.5% **HKVVLFMKGTKDFPQCGFSHTVVQILRSLDVPFETLDVLANE---ALRQGLKEYSSWPTFPQLYIDGEFFGGCDITVEAY**

4 Pp3c24_14100 100.0% 77.2% **NKVVLFMKGNKQFPQCGFSNTCVQILNTLNVPYETVNILEDD---NLRQGMKEYSAWPTFPQLYIDGEFFGGCDITYESY**

5 Pp3c8_18520 100.0% 77.2% **NKVVLFMKGNKMFPQCGFSNTCVRILNSLNVPYETVNILEDD---RLRQGMKEYSDWPTFPQLYIDGEFFGGCDITYAAY**

6 Azfi_s0173g055803 100.0% 73.9% **NKVVLFMKGNKLFPQCGFSNTVVQILNSLNVPYETVDILANE---QMRSAMKIYSSWPTFPQLYIDGEFFGGCDITMEAF**

7 Sacu_s0022g008645 100.0% 67.4% **NKVVLFMKGTKLFPQCGFSNTVVQILNSLNVPYETVNILENE---QLRSAMKIYSSWPTFPQLYIDGEFFGGCDITLSLY**

8 AtGrxS14_AT3G54900 100.0% 70.7% **EKVVLFMKGTRDFPMCGFSNTVVQILKNLNVPFEDVNILENE---MLRQGLKEYSNWPTFPQLYIGGEFFGGCDITLEAF**

9 AaBonn_Sc2ySwM_228_376_1 100.0% 75.0% **NKVVVFMKGTKLFPQCGFSNTVVQILNSLDAKYETVNILEDN---GLRQAMKEYSNWPTFPQLYIDGEFFGGCDIAIEAY**

10 Mapoly0006s0309 100.0% 75.0% **NKVVLFMKGNKNFPQCGFSNTCVQILNQFGVPYETVNILEDD---SLRQGVKEYSNWPTFPQLYIDGEFFGGCDITIEAF**

11 AtGrxS16_AT2G38270 94.6% 53.3% **SKVVAFIKGSRSAPQCGFSQRVVGILESQGVDYETVDVLDDEYNHGLRETLKNYSNWPTFPQIFVKGELVGGCDILTSMY**

12 G22937_CHBRA273g00240 98.9% 40.4% **TPVIAFIKGTRSSPQCGFSHRVLMALNKEGVDYETVNVLDEEFNPGVREAIKAYSKWPTIPQVFINGELIGGADLLEESV**

13 57444_Selmo 92.4% 46.6% **HNVVAFIKGSRTAPQCGFSHRVLTILEQQGVDFETVNVLDEEHNSGVREAIKSYSQWPTIPQVFVKGEFVGGADVMSELA**

14 Mapoly0001s0103 100.0% 46.3% **CTVVAFVKGTRTAPQCGFSHRVLTILNENGADYEVVNVLDDHHNPGLREAIKEYSQWPTIPQVYVKGEFVGGADILDEMV**

15 Sacu_s0028g009899 97.8% 49.5% **NKVVAFIKGSRTAPQCGFSHKVLTILNEHGIEYESVNVLDDEHNSGLREAIKVYSQWPTIPQIYAYGEFVGGADILEELA**

16 Azfi_s0224g058869 97.8% 45.2% **NKVVAFIKGSRTSPQCGFSHRVLTILNEHGIDYESVNVLDEEHNSGLREAIKVYSQWPTIPQVFAYGEFVGGADILEELV**

17 Pp3c14_14790 95.7% 46.2% **LKVVAFIKGTRTSPQCGFSHRVLTILNEQGVDYEVLNVLDEDHNPGLREVIKKYSQWPTIPQLYVKGEFVGGADVLDEMV**

18 Bradi4g45030 95.7% 49.5% **NKVVAFIKGSRSAPQCGFSQRVVGILEAHGVDFASVDILDEEHNHGLRETLKTYSNWPTFPQVFVGGELVGGCDIISSMA**

19 AaBonn_Sc2ySwM_362_2023_1 95.7% 49.5% **HSVVAFIKGPRTAPQCGFSHKVLTILNEQGVDYETVNVLDEEHNRGVRDAIKTYSQWPTIPQLYVKGEFVGGADVLEELV**

20 AtGrxS15_AT3G15660 100.0% 43.5% **NPVMIYMKGVPESPQCGFSSLAVRVLQQYNVPISSRNILEDQ---ELKNAVKSFSHWPTFPQIFIKGEFIGGSDIILNMH**

cov pid **81**  **. ]** **95**

1 G41800_CHBRA601g00040 100.0% 100.0% **QNGELQEIVEKALVS**

2 102863_Selmo 100.0% 75.0% **QSGQLKEVVEKAMCS**

3 Bradi1g01570 100.0% 68.5% **KSGELQETLEKAMCS**

4 Pp3c24_14100 100.0% 77.2% **NSGELKELLDRAMLS**

5 Pp3c8_18520 100.0% 77.2% **SSGELKEVLEKAMLS**

6 Azfi_s0173g055803 100.0% 73.9% **KSGELQEVVEKAMCS**

7 Sacu_s0022g008645 100.0% 67.4% **LPEQQPHVIFSPMRG**

8 AtGrxS14_AT3G54900 100.0% 70.7% **KTGELQEEVEKAMCS**

9 AaBonn_Sc2ySwM_228_376_1 100.0% 75.0% **QSGELKELLEKVSLS**

10 Mapoly0006s0309 100.0% 75.0% **KSGELKETLDKAMLS**

11 AtGrxS16_AT2G38270 94.6% 53.3% **ENGELANILN-----**

12 G22937_CHBRA273g00240 98.9% 40.4% **EKGEFKQLLAAIKK-**

13 57444_Selmo 92.4% 46.6% **ESGEISKL-------**

14 Mapoly0001s0103 100.0% 46.3% **QSGEIKSLFQKSGPK**

15 Sacu_s0028g009899 97.8% 49.5% **TIGKIKEVFQKSS--**

16 Azfi_s0224g058869 97.8% 45.2% **TDGKIKAVFQQST--**

17 Pp3c14_14790 95.7% 46.2% **QSGEIKDLFKN----**

18 Bradi4g45030 95.7% 49.5% **ENGELAALFQK----**

19 AaBonn_Sc2ySwM_362_2023_1 95.7% 49.5% **SSGEIKNIFKK----**

20 AtGrxS15_AT3G15660 100.0% 43.5% **KEGELEQKLKDVSGN**

>G41800_CHBRA601g00040

NKVVLFMKGSKLFPQCGFSNTCVQILNVLNVPYETVNILEDE---DLRQGMKEYSKWPTFPQLYVDKEFIGG

CDIMIELYQNGELQEIVEKALVS

>102863_Selmo

HKVVLFMKGTKLFPQCGFSNTVVQILNNLSVPYETVNILEDD---GLRQGLKAYSNWPTFPQLYIDGEFFGG

CDITLEAFQSGQLKEVVEKAMCS

>Bradi1g01570

HKVVLFMKGTKDFPQCGFSHTVVQILRSLDVPFETLDVLANE---ALRQGLKEYSSWPTFPQLYIDGEFFGG

CDITVEAYKSGELQETLEKAMCS

>Pp3c24_14100

NKVVLFMKGNKQFPQCGFSNTCVQILNTLNVPYETVNILEDD---NLRQGMKEYSAWPTFPQLYIDGEFFGG

CDITYESYNSGELKELLDRAMLS

>Pp3c8_18520

NKVVLFMKGNKMFPQCGFSNTCVRILNSLNVPYETVNILEDD---RLRQGMKEYSDWPTFPQLYIDGEFFGG

CDITYAAYSSGELKEVLEKAMLS

>Azfi_s0173g055803

NKVVLFMKGNKLFPQCGFSNTVVQILNSLNVPYETVDILANE---QMRSAMKIYSSWPTFPQLYIDGEFFGG

CDITMEAFKSGELQEVVEKAMCS

>Sacu_s0022g008645

NKVVLFMKGTKLFPQCGFSNTVVQILNSLNVPYETVNILENE---QLRSAMKIYSSWPTFPQLYIDGEFFGG

CDITLSLYLPEQQPHVIFSPMRG

>AtGrxS14_AT3G54900

EKVVLFMKGTRDFPMCGFSNTVVQILKNLNVPFEDVNILENE---MLRQGLKEYSNWPTFPQLYIGGEFFGG

CDITLEAFKTGELQEEVEKAMCS

>AaBonn_Sc2ySwM_228_376_1

NKVVVFMKGTKLFPQCGFSNTVVQILNSLDAKYETVNILEDN---GLRQAMKEYSNWPTFPQLYIDGEFFGG

CDIAIEAYQSGELKELLEKVSLS

>Mapoly0006s0309

NKVVLFMKGNKNFPQCGFSNTCVQILNQFGVPYETVNILEDD---SLRQGVKEYSNWPTFPQLYIDGEFFGG

CDITIEAFKSGELKETLDKAMLS

>AtGrxS16_AT2G38270

SKVVAFIKGSRSAPQCGFSQRVVGILESQGVDYETVDVLDDEYNHGLRETLKNYSNWPTFPQIFVKGELVGG

CDILTSMYENGELANILN-----

>G22937_CHBRA273g00240

TPVIAFIKGTRSSPQCGFSHRVLMALNKEGVDYETVNVLDEEFNPGVREAIKAYSKWPTIPQVFINGELIGG

ADLLEESVEKGEFKQLLAAIKK-

>57444_Selmo

HNVVAFIKGSRTAPQCGFSHRVLTILEQQGVDFETVNVLDEEHNSGVREAIKSYSQWPTIPQVFVKGEFVGG

ADVMSELAESGEISKL-------

>Mapoly0001s0103

CTVVAFVKGTRTAPQCGFSHRVLTILNENGADYEVVNVLDDHHNPGLREAIKEYSQWPTIPQVYVKGEFVGG

ADILDEMVQSGEIKSLFQKSGPK

>Sacu_s0028g009899

NKVVAFIKGSRTAPQCGFSHKVLTILNEHGIEYESVNVLDDEHNSGLREAIKVYSQWPTIPQIYAYGEFVGG

ADILEELATIGKIKEVFQKSS--

>Azfi_s0224g058869

NKVVAFIKGSRTSPQCGFSHRVLTILNEHGIDYESVNVLDEEHNSGLREAIKVYSQWPTIPQVFAYGEFVGG

ADILEELVTDGKIKAVFQQST--

>Pp3c14_14790

LKVVAFIKGTRTSPQCGFSHRVLTILNEQGVDYEVLNVLDEDHNPGLREVIKKYSQWPTIPQLYVKGEFVGG

ADVLDEMVQSGEIKDLFKN----

>Bradi4g45030

NKVVAFIKGSRSAPQCGFSQRVVGILEAHGVDFASVDILDEEHNHGLRETLKTYSNWPTFPQVFVGGELVGG

CDIISSMAENGELAALFQK----

>AaBonn_Sc2ySwM_362_2023_1

HSVVAFIKGPRTAPQCGFSHKVLTILNEQGVDYETVNVLDEEHNRGVRDAIKTYSQWPTIPQLYVKGEFVGG

ADVLEELVSSGEIKNIFKK----

>AtGrxS15_AT3G15660

NPVMIYMKGVPESPQCGFSSLAVRVLQQYNVPISSRNILEDQ---ELKNAVKSFSHWPTFPQIFIKGEFIGG

SDIILNMHKEGELEQKLKDVSGN

**GR**

cov pid  **1** **[ . . . . : . . .** **80**

1 G36558_CHBRA44g00300 100.0% 100.0% **YDYDVLTIGAGSGGVRASRFVAG-FGAKVAVCELPFALTSSDSKGGVGGTCVIRGCVPKKLLVYGSQFAHEFLASKGFGW**

2 AaOxford_utg000033l_212_1 99.3% 43.7% **YDYDLLAIGAGSGGVRASRFAAS-MGARVAICELPFAAVSSDAAGGVGGTCVLRGCVPKKLLVIGANFSHDFHASEGFGW**

3 Ap_utg000041l_218_1 99.3% 43.5% **YDYDLLAIGAGSGGVRASRFAAS-MGARVAICELPFAAVSSDAAGGVGGTCVLRGCVPKKLLVIGANFSHDFHASEGFGW**

4 Mapoly0001s0404 99.3% 43.5% **YDYDLVTIGAGSGGVRASRFAAN-LGAKVAVCELPFSTISSDSAGGVGGTCVLRGCVPKKILVYGSQFAHSFEDSKGFGW**

5 Pp3c4_17890V3 99.3% 42.9% **YDYDLIAIGAGSGGVRAARFASQ-FGANVAVCEMPFAPISSDEAGGVGGTCVLRGCVPKKLLVYGANFPHDFESSRGFGW**

6 438142_Selmo 99.3% 41.5% **FDYDLITIGAGSGGVRASRFAAN-FGARVAVVELPFATISSDEAGGVGGTCVLRGCVPKKLLVYGSSFAHEFDESKGFGW**

7 Azfi_s0059g034599 99.7% 41.3% **FDFELITIGGGSGGVRASRFASN-FGAKVALIELPFATISSDSAGGLGGTCVLRGCVPKKLLVYGSKFSHEFEESRGFGW**

8 Bradi1g74180 99.7% 40.0% **YDYDLFTIGAGSGGVRASRFASTLYGARAAICEMPFATIATDDLGGLGGTCVLRGCVPKKLLVYASKFSHEFEESHGFGW**

9 Bradi3g26737 99.7% 38.0% **YEYDLFTIGAGSGGMRASRFASTLYGARAAVCEMPFATVASDALGGVGGTCVLRGCVPKKLLVYASKYSHEFDESHGFGW**

10 AT3G54660 99.7% 40.5% **YDFDLFTIGAGSGGVRASRFATS-FGASAAVCELPFSTISSDTAGGVGGTCVLRGCVPKKLLVYASKYSHEFEDSHGFGW**

11 G21301_CHBRA256g00370 89.4% 29.8% **LEWE-------AEGLEA-------RGWQQAMEP----------------RCVLRGCVPKKILVYGSQFGSEFEDAEGYGW**

12 AaOxford_utg000125l_130_2 97.0% 35.6% **FDYDLFVIGAGSGGVRASRIAAG-FGAKVAVVELAFHPVSSEALGGIGGTCVLRGCVPKKILVYGSEFHDQFEDARNYGW**

13 AaBonn_Sc2ySwM_368_3221_corrected 99.3% 33.2% **FDYDLFVIGAGSGGVRASRIAAG-FGAKVAVVELAFHPVSSEALGGIGGTCVLRGCVPKKILVYGSEFHDQFEDARNYGW**

14 Ap_utg000045l_632_1 63.2% 25.4% **--------------------------------------------------------------------------------**

15 Mapoly0002s0333 99.3% 33.6% **FDYDLFVIGGGSGGVRASRMSAS-HGAKVALVELPYHPISSETHGGLGGTCVLRGCVPKKILVYGSGFSHEFQDAKGFGW**

16 Pp3c5_16850V3 99.3% 36.6% **YDYDLFVIGAGSGGVRASRTAAG-FGAKVAICELPYHPISSESAGGIGGTCVLRGCVPKKILVYGSAFGGEFQDAREFGW**

17 164085_Selmo 99.3% 35.8% **FDFDLFTIGAGSGGVRASRTAAN-FGAKVAVVELPFAHVSSESAGGVGGTCVIRGCVPKKILVYASMFSAEFQDSKNFGW**

18 Sacu_s0076g017562 99.3% 35.3% **FDFDLFTIGAGSGGVRASRMSSG-YGAKVAICELPFHPISSEGLGGCGGTCVIRGCVPKKILVYGASFKGEFEDSKNFGW**

19 Azfi_s0090g042652 90.7% 32.0% **---------------------------MVAICELPFHPISSEGLGGCGGTCVIRGCVPKKILVYGAAFKGEFEDSKNFGW**

20 Bradi3g55030 99.3% 34.7% **YEYDLFVIGAGSGGVRGSRTSAG-FGAKVAICELPFHPISSEWQGGHGGTCVIRGCVPKKILVYGAAFRGEFEDAKNFGW**

21 AT3G24170 99.3% 31.7% **YDFDLFVIGAGSGGVRAARFSAN-HGAKVGICELPFHPISSEEIGGVGGTCVIRGCVPKKILVYGATYGGELEDAKNYGW**

22 P48638_GSHR_NOSS1 96.0% 28.5% **FDYDLFVIGAGSGGLAASKRAAS-YGAKVAIAE----------NDLVGGTCVIRGCVPKKLMVYGSHFPALFEDAAGYGW**

23 AaOxford_utg000003l_24_1_extN 96.4% 21.5% **YKYHLLVVGGGPGGLAAAKRVAR-RGYRVALAE----------QDTVGGTCVMRGCVPEKLMTFAAKFPEACRDAVSYGW**

24 AaBonn_Sc2ySwM_228_5258_1_extN 96.4% 21.5% **YKYHLLVVGGGPGGLAAAKRVAR-RGYRVALAE----------QDTVGGTCVMRGCVPEKLMTFAAKFPEACRDAVSYGW**

25 Ap_utg000048l_105_1 96.4% 21.3% **YKYHLLVVGGGPGGLAAAKRVAR-QGYRVALAE----------QDTVGGTCVMRGCVPEKLMTFAAKFPEACRDAVSYGW**

26 P06715_GSHR_ECOLIK12 95.0% 23.6% **KHYDYIAIGGGSGGIASINRAAM-YGQKCALIE----------AKELGGTCVNVGCVPKKVMWHAAQIR---EAIHMYGP**

cov pid  **81**  **. 1 . . . . : .** **160**

1 G36558_CHBRA44g00300 100.0% 100.0% **SFDKEPSHDWVTLIDNKNKEIQRLTGVYRKLLDGSKVDLLEGRAKLVGPHTVEV----GS-KRYTAKNILLAVGGRPFVP**

2 AaOxford_utg000033l_212_1 99.3% 43.7% **KLESLPVHDWSALVARKNQEINRLTGVYKSLLAGSGVKLIEGRGKITGAHAVEV----GG-KEYTAKHILISVGGRATVP**

3 Ap_utg000041l_218_1 99.3% 43.5% **KLESLPVHDWSALVARKNQEINRLTGVYKSLLAGSGVKLIEGRGKITGAHAVEV----GG-KEYTAKHILISVGGRATVP**

4 Mapoly0001s0404 99.3% 43.5% **SYDAKPKHDWNMLIDNKNKELNRLLGVYKNILKNANVALIEGRGKIVDPHTVDI----SG-ERIRARHILVAVGGRPYVP**

5 Pp3c4_17890V3 99.3% 42.9% **SFETEPKHDWKTLITNKNAELNRLTGVYKSLLQKSEVDLIEGRGKIVDAHTVEV----KG-KQYTTQHILVSVGGRATVP**

6 438142_Selmo 99.3% 41.5% **SYDSPPRHDWKTLMKNKNTELQRLIGVYKSILSSAGVTLVEGRGKILDAHTVQV----SGKEKYTAKYILVAVGGRSTVP**

7 Azfi_s0059g034599 99.7% 41.3% **NFESEPKHDWTTLIAQKNAELRRLLEVYKNILTNAGVSVIEGRGKIVDNHTVEV----NG-KHYTAKHILVAVGGRAFVP**

8 Bradi1g74180 99.7% 40.0% **TYETDPKHDWSTLIANKNTELQRLVGIYKNILNNAGVDLIEGRGKVVDPHTVSV----DG-KLYTAKNILIAVGGRPSMP**

9 Bradi3g26737 99.7% 38.0% **KYETDPKHDWSTLMTRKNLELQRLVDFQTDMLKNSGVTLIEGRGKIVDPHTVSV----DG-KLYTARNILIAVGARPSIP**

10 AT3G54660 99.7% 40.5% **KYETEPSHDWTTLIANKNAELQRLTGIYKNILSKANVKLIEGRGKVIDPHTVDV----DG-KIYTTRNILIAVGGRPFIP**

11 G21301_CHBRA256g00370 89.4% 29.8% **KL-EKPEFDWKKLIVGKTKEIERLNGIYKRILANAGVTLIEGEGKLLDAHTVQVTPPAGEVKYITAKHILIATGSRAAVL**

12 AaOxford_utg000125l_130_2 97.0% 35.6% **NLDETITFDWKKLIASKHREITRLNGIYKKMLAGAGVTVYEGEGKFLDRHTVQITEPSGEVKTYRSKHILIATGARAVTL**

13 AaBonn_Sc2ySwM_368_3221_corrected 99.3% 33.2% **NLDETITFDWKKLIASKHREITRLNGIYKKMLAGAGVTVYEGEGKFLDRHTVQITEPSGEVKTYRSKHILIATGARAVTL**

14 Ap_utg000045l_632_1 63.2% 25.4% **------------------------------MLAGAGVTVYEGEGKFLDRHTVQVTEPSGEVKTYRSKHILIATGARAVTL**

15 Mapoly0002s0333 99.3% 33.6% **EIDGEIRFNWKKLIQNKTKEIERLNGVYKRMLVNAGVTLFEGFGRIVDKHTVEVKDVDGQTKTFTTNHILIATGGKAVKL**

16 Pp3c5_16850V3 99.3% 36.6% **NINGDITFDWKRLIANKTKEIIRLNGVYKRLLAGSKVDMYEGGGKIVDPHTVDVEQTGGEVKRFTAKKILVATGGRAVPL**

17 164085_Selmo 99.3% 35.8% **NVPDGITFEWKRLIANKDREIERLNGIYKRLLTGSGVTILEGRASLVDDHTVEVSHSDGSSKRYRAKHILVATGSRAVRL**

18 Sacu_s0076g017562 99.3% 35.3% **DIDGEITFDWKRLIENKTKEITRLNGVYKRILSGAGVTFIEGAGKVIDPHTVEVQLPDGQLKRFTTKHILIATGSRAVLI**

19 Azfi_s0090g042652 90.7% 32.0% **DINGEITFDWKRLIENKTKEITRLNGIYKRILTGAGVTLIEGAGRVIDPHTVEVQYPNGQSKRFTTRHILIATGGRAVLL**

20 Bradi3g55030 99.3% 34.7% **EINGDINYNWKKLLENKTQEIVRLNGVYKRILGNSGVTMIEGAGSVVDAHTVEVTQPDGSKQRHTAKHILIATGSRAHLV**

21 AT3G24170 99.3% 31.7% **EINEKVDFTWKKLLQKKTDEILRLNNIYKRLLANAAVKLYEGEGRVVGPNEVEVRQIDGTKISYTAKHILIATGSRAQKP**

22 P48638_GSHR_NOSS1 96.0% 28.5% **QV-GKAELNWEHFITSIDKEVRRLSQLHISFLEKAGVELISGRATLVDNHTVEV----GE-RKFTADKILIAVGGRPIKP**

23 AaOxford_utg000003l_24_1_extN 96.4% 21.5% **SQECSQNFDWPRFIEAKEQETRRLSQVHRQALEKAGVELLRGRATFINAHTVDV----GG-AEVTADTILIAVGAIPVPH**

24 AaBonn_Sc2ySwM_228_5258_1_extN 96.4% 21.5% **SQECSQNFDWPRFIEAKEQETRRLSQVHRQALEKAGVELLRGRATFINAHTVDV----GG-AEVTADTILIAVGAIPVPH**

25 Ap_utg000048l_105_1 96.4% 21.3% **SQECSQNFDWPRFIEAKEQETRRLSQVHRQALEKAGVELLRGRATFINAHTVDV----GG-AEVTADTILIAVGAIPVPH**

26 P06715_GSHR_ECOLIK12 95.0% 23.6% **DYTTINKFNWETLIASRTAYIDRIHTSYENVLGKNNVDVIKGFARFVDAKTLEV----NG-ETITADHILIATGGRPSHP**

cov pid **161**  **. . . 2 . . . .** **240**

1 G36558_CHBRA44g00300 100.0% 100.0% **DIPGKEYGITSDEALELPERPQSICIIGGGYIALEFASIFNALGTETHVLIRQPKVLRGFDEEIRDFLGEQLQTRGIKIH**

2 AaOxford_utg000033l_212_1 99.3% 43.7% **DIPGKEYVITSDEALELPELPKSICIVGGGYIALEFAGIFNGLGSDVHVYIRGDKVLRGFDEEIRDFLADQLKQKGINIH**

3 Ap_utg000041l_218_1 99.3% 43.5% **DIPGKEYVITSDEALELPELPKSICIVGGGYIALEFAGIFNGLGSDVHVYIRGDKVLRGFDEEIRDFLADQLKQKGINIH**

4 Mapoly0001s0404 99.3% 43.5% **DIPGKEFAITSDEALDLPSRPEKICIVGGGYIALEFAGIFNGLGSEVHVFIRQDKVLRGFDEEVRDFVAEQMSLKGIKFH**

5 Pp3c4_17890V3 99.3% 42.9% **NIPGKENAITSDEALNLSERPNKICIVGGGYIALEFAGIFAGLGTEVHVFVRQPKVLRGFDEEIRDFIAAQLQAQGIVFH**

6 438142_Selmo 99.3% 41.5% **DIPGKEFVITSDHALDLPARPEKICIVGAGYIALEFASIFNGFGSEVHVFLRGPKVLRGFDDEIRDFVADQMAAKGVKFH**

7 Azfi_s0059g034599 99.7% 41.3% **DIVGKEYAITSDEALDLPSRPEKIGIIGGGYISLEFAGIFNSLGSEVHVFIRQNKVLRGFDEEIRDFIADQMTLRGINFH**

8 Bradi1g74180 99.7% 40.0% **TIPGIDHVIDSDAALDLPSKPEKIAIVGGGYIALEFAGIFNGLKSDVHVFIRQPKVLRGFDEEVRDFLAEQMSLRGITFH**

9 Bradi3g26737 99.7% 38.0% **DIPGIEHVIDSDAALDLPSRPEKIAIVGGGYIALEFAGIFNGLKSEVHVYIRQKKVLRGFDEEVRDFVTQQMSLRGITFH**

10 AT3G54660 99.7% 40.5% **DIPGKEFAIDSDAALDLPSKPKKIAIVGGGYIALEFAGIFNGLNCEVHVFIRQKKVLRGFDEDVRDFVGEQMSLRGIEFH**

11 G21301_CHBRA256g00370 89.4% 29.8% **NIPGKEYGITSDEALSLEQFPKRVVVVGGGYIAVEFAGIFKGLGSEVHIMYRKELPLRGFDQEMRVIVAENLKARGIKVH**

12 AaOxford_utg000125l_130_2 97.0% 35.6% **NIPGKELAITSDEALSLEKFPKRAVIVGAGYIAVEFSGIFSGLGSKVDLFFRSDAPLRGFDDEIRGAVATHLQNRGVQLH**

13 AaBonn_Sc2ySwM_368_3221_corrected 99.3% 33.2% **NIPGKELAITSDEALSLEKFPKRAVIVGAGYIAVEFSGIFSGLGSKVDLFFRSDAPLRGFDDEIRGAVATHLQNRGVQLH**

14 Ap_utg000045l_632_1 63.2% 25.4% **NIPGKELAITSDEALSLEKFPKRAVIVGAGYIAVEFSGIFSGLGSKVDLFFRSDAPLRGFDDEIRGAVATHLQNRGVQLH**

15 Mapoly0002s0333 99.3% 33.6% **NIPGQELGITSDEGLSLEEFPKKVLIIGGGYIAVEFAGIYSGMGADVHLCYRKSLPLAGFDTEMAEVVARNLEARGIKCH**

16 Pp3c5_16850V3 99.3% 36.6% **NIPGKELAITSDEGLSLEEFPKRVVIAGGGYIAVEFAGIYSGMGAKVDLFYRKPLPLTGFDEEMREVVARNLENRGIKCH**

17 164085_Selmo 99.3% 35.8% **NVPGKELAITSDEGLNLDELPRRCVIVGGGYIAVEFAGIYSGMGSKVELLYRKKTPLRGFDDEMRAVVARNLENRGVHLR**

18 Sacu_s0076g017562 99.3% 35.3% **DIPGKELAITSDEALSLEEFPKRSVIVGGGYIAVEFAGIYNAMGSKVDLFYRKDAPLRGFDDEMRITVAKNLEGRGVNLH**

19 Azfi_s0090g042652 90.7% 32.0% **DIPGKELAITSDEALSLEEFPKRSVIVGGGYIAVEFAGIYNGMGSKVNLFYRKDAPLRGFDDEMRVAVAKNLENRGVVLH**

20 Bradi3g55030 99.3% 34.7% **DIPGKELAITSDEALSLEELPKRAVILGGGYIAVEFASIWKGLGAEVDLFYRKELPLRGFDDEMRTVVASNLEGRGIRLH**

21 AT3G24170 99.3% 31.7% **NIPGHELAITSDEALSLEEFPKRAIVLGGGYIAVEFASIWRGMGATVDLFFRKELPLRGFDDEMRALVARNLEGRGVNLH**

22 P48638_GSHR_NOSS1 96.0% 28.5% **ELPGMEYGITSNEIFHLKTQPKHIAIIGSGYIGTEFAGIMRGLGSQVTQITRGDKILKGFDEDIRTEIQEGMTNHGIRII**

23 AaOxford_utg000003l_24_1_extN 96.4% 21.5% **DVPGIQHAVTNSELFVLPEQPREMAIMGGDYIAVKLGGIMHNLGSRLTHVVPEESVLATFDQDLCEGVTDGITKRGITVL**

24 AaBonn_Sc2ySwM_228_5258_1_extN 96.4% 21.5% **DVPGIQHAVTNSELFVLPEQPREMAIMGGDYIAVKLGGIMHNLGSRLTHVVPEESVLATFDQDLCEGVTDGITKRGITVL**

25 Ap_utg000048l_105_1 96.4% 21.3% **DVPGVQHAVTNSELFVLPEQPREMAIMGGDYIAVKLGGIMHNLGSRVTHVVPEESVLATFDQDLCEGVTDGITKRGITVL**

26 P06715_GSHR_ECOLIK12 95.0% 23.6% **DIPGVEYGIDSDGFFALPALPERVAVVGAGYIAVELAGVINGLGAKTHLFVRKHAPLRSFDPMISETLVEVMNAEGPQLH**

cov pid **241**  **: . . . . 3 . .** **320**

1 G36558_CHBRA44g00300 100.0% 100.0% **NRASPVSVEKDADTGKLVL--------------TTDKGEKITTEVVMFATGRKPRTERLGAEEVGVELDSSGAIK-----**

2 AaOxford_utg000033l_212_1 99.3% 43.7% **FRETPVAVEKNSN-GSLKL--------------VTNKGSKDA-SAVMFATGRAPNTKNLGLEDVGVKLDKKGAIEVDEYS**

3 Ap_utg000041l_218_1 99.3% 43.5% **FGETPVAVEKNSN-GSLKL--------------VTNKGSKDA-SAVMFATGRAPNTKNLGLEDVGVKLDKKGAIEVDEYS**

4 Mapoly0001s0404 99.3% 43.5% **FGESPTAVEKGSD-GKFTL--------------VTTKGSEVA-DSIMFATGRKPNTKNLGLEDVGVNINSKGAIEVDEYS**

5 Pp3c4_17890V3 99.3% 42.9% **FGESPTAIEKRND-GTFCL--------------VTDSGKEVS-DLVMFATGRAPNTKNLGLEEVGVKLDKRGAIEVDSFS**

6 438142_Selmo 99.3% 41.5% **FEESPEAVEKCPD-GSLLL--------------RTNKSTEKT-KCVMFATGRAPNTKNLGLEDIGVRLGKNGAIMVDEYS**

7 Azfi_s0059g034599 99.7% 41.3% **FEESPVAIEKDST-GQLSL--------------KTNKGSTHSFSHIMFATGRRANTKNLGLEAAGVALDQKGAIQVDEYS**

8 Bradi1g74180 99.7% 40.0% **TEQSPQAVTKSSD-GLLSL--------------KTNKETIGGFSHVMFATGRKPNTKNLGLEEVGVKMDRNGAIVVDEYS**

9 Bradi3g26737 99.7% 38.0% **TEQTPQAITKSDD-GLLSL--------------KTNKGTVNGFSHIMFATGRKPNTKNLGLEDVGVKMDKHGSVMVDEYS**

10 AT3G54660 99.7% 40.5% **TEESPEAIIKAGD-GSFSL--------------KTSKGTVEGFSHVMFATGRKPNTKNLGLENVGVKMAKNGAIEVDEYS**

11 G21301_CHBRA256g00370 89.4% 29.8% **AGLNPKRIEKTAD-G-LVV--------------TTDKGEKLVGDVVMFATGRKPNVRRLNLKAASIATDEVGAIVVNEYS**

12 AaOxford_utg000125l_130_2 97.0% 35.6% **PGTNITKIEKVED-G-LKV--------------TTDKGEEFVTDEVLFATGRKPNSHRLNLEAVGVALDQQGGIKVNEFS**

13 AaBonn_Sc2ySwM_368_3221_corrected 99.3% 33.2% **PGTNITKIEKVED-G-LKV--------------TTDKGEEFVTDEVLFATGRKPNSHRLNLEAVGVALDQQGGIKVNEFS**

14 Ap_utg000045l_632_1 63.2% 25.4% **PGTNITKIEKVED-G-LKV--------------TTDKGEEFVTDEVLFATGRKPNSHRLNLEAVGVALDQQGGIKVNEFS**

15 Mapoly0002s0333 99.3% 33.6% **KHTNIVKLESHYL-G-IKA--------------TTDTNEEYHVDQVMFATGREPKTANLNLEGVGVELDEAGAIKVNEYS**

16 Pp3c5_16850V3 99.3% 36.6% **PETNLTKLEKVAG-G-IKV--------------TVDNGEEHEVDAVMFATGRKPSTKNIGLEDVGVELDKTGAIKVNEYS**

17 164085_Selmo 99.3% 35.8% **PDTNVTKIEKVG--GELKV--------------SIDNGGEIMTDAVLFAVGRKPKTSGLNLEELGVELDKSGAIKVDEYS**

18 Sacu_s0076g017562 99.3% 35.3% **PKANIIKIEKTEG-G-LKA--------------YTDKGDVFETDVVLMAVGRKPNIKRLNLESVGVELHTDDAIKVNEYS**

19 Azfi_s0090g042652 90.7% 32.0% **PSTNIVKIEKVES-G-LKV--------------YTDKGDVLETDVVLMAVGRAPNVNKLNLDVVGVELHGDGAIKVNEYS**

20 Bradi3g55030 99.3% 34.7% **PATNLTELSKTAD-G-IKV--------------VTDKGDELMADVVLFATGRTPNTNKLNLEAVGVEVDQIGAIKVDEFS**

21 AT3G24170 99.3% 31.7% **PQTSLTQLTKTDQ-G-IKV--------------ISSHGEEFVADVVLFATGRSPNTKRLNLEAVGVELDQAGAVKVDEYS**

22 P48638_GSHR_NOSS1 96.0% 28.5% **PKNVVTAIEQVPEGLKISL--------------SGEDQEPIIADVFLVATGRVPNVDGLGLENAGVDVSTMNAIAVNEYS**

23 AaOxford_utg000003l_24_1_extN 96.4% 21.5% **NRCKVEEIRKVEEEGGGKLLEVVVGPVGDGEIQEKEKCRTLRVDTVLDGMLRLPNIEDLGLEVAGVRVTARRCIAVDEHC**

24 AaBonn_Sc2ySwM_228_5258_1_extN 96.4% 21.5% **NRCKVEEIRKVEEEGGGKLLEVVVGPVGDGEIQEKEKCRTLRVDTVLDGMLRLPNIEDLGLEVAGVRVTARRCIAVDEHC**

25 Ap_utg000048l_105_1 96.4% 21.3% **SRCKVEEIRKVEEEGGGKLLEVVVGPASDGEIQEKEKCRTLRVDTVLDGMLRLPNIEDLGLEAAGVRVAARRCIAVDEHC**

26 P06715_GSHR_ECOLIK12 95.0% 23.6% **TNAIPKAVVKNTD-GSLTL--------------ELEDGRSETVDCLIWAIGREPANDNINLEAAGVKTNEKGYIVVDKYQ**

cov pid **321**  **. . : . . . . 4** **400**

1 G36558_CHBRA44g00300 100.0% 100.0% **--------------------------------------------------------------------------------**

2 AaOxford_utg000033l_212_1 99.3% 43.7% **RTSVDSIWAVGDVTNRVNLTPVALMEAMAFSKTVFGDNPTKP-DHRNIASAVFTNPPIGTVGLTESQAVELYEDVDVFTA**

3 Ap_utg000041l_218_1 99.3% 43.5% **RTSVDSIWAVGDVTNRVNLTPVALMEAMAFSKTVFGDNPTKP-DHRNIASAVFTNPPIGTVGLTESQAVELYEDVDVFTA**

4 Mapoly0001s0404 99.3% 43.5% **RTSVASIWAVGDVTDRMNLTPVALMEGMAFAKTAFGDEPSKP-DHRYVASAVFTQPPIGTVGLTEAEALETYGDIDVYTS**

5 Pp3c4_17890V3 99.3% 42.9% **RTNVDSIWAIGDVTNRINLTPVALMEGMAMAKTAFGNEPTKP-DYRFIASAVFTQPPIGTVGYTEEQAVEKFGDVDVYTS**

6 438142_Selmo 99.3% 41.5% **KSNVDSIWAVGDVTNRTNLTPVALMEGMAFSKTVFGDRPTKP-DYNNIPSAVFTQPPIGTVGLTEEQAIKELRNIDVYTS**

7 Azfi_s0059g034599 99.7% 41.3% **RTSVESIWAVGDVTDRINLTPVALMEGGAFARTVFGGQAVKP-DYRNVASAVFTQPPIGTVGLTEEQAIAKYGDVDVYTA**

8 Bradi1g74180 99.7% 40.0% **RTSVDSIWAVGDVTDRINLTPVALMEGGAFAKTLFGDEPTKP-DYRAVPAAVFSQPPIGQVGLTEEQAIEEYGDVDVFLS**

9 Bradi3g26737 99.7% 38.0% **RTSVDSIWAVGDVTNRVNLTPVALMEGGALTHTIFGNDPIKP-DHSAVPSAVFSQPPIGQVGLTEEQATEKYGDVDVYTS**

10 AT3G54660 99.7% 40.5% **QTSVPSIWAVGDVTDRINLTPVALMEGGALAKTLFQNEPTKP-DYRAVPCAVFSQPPIGTVGLTEEQAIEQYGDVDVYTS**

11 G21301_CHBRA256g00370 89.4% 29.8% **QTNVPNIWAVGDVTNRINLTPVALMEGMAFAKTVFGRQPTKP-DYQFVPSAVFCQPPMAVVGYTEDEAVAEGGDIVVYTS**

12 AaOxford_utg000125l_130_2 97.0% 35.6% **QTNVPNIWAVGDVTNRIQLTPVALMEGHCFAKTVFGGQPSKP-DYRDVACAVFCQPPVSVVGLTEKDALEQSGDILVFTS**

13 AaBonn_Sc2ySwM_368_3221_corrected 99.3% 33.2% **QTNVPNIWAVGDVTNRIQLTPVALMEGHCFAKTVFGGQPSKP-DYRDVACAVFCQPPVSVVGLTEKDALEQSGDILVFTS**

14 Ap_utg000045l_632_1 63.2% 25.4% **QTNVPNIWAVGDVTNRIQLTPVALMEGHCFAKTVFGGQPSKP-DYRDVACAVFCQPPVSVVGLTEKDALEQSGDILVFTS**

15 Mapoly0002s0333 99.3% 33.6% **QTSIPSIWAVGDVTNRINLTPVALMEGTCFAKTVFLKEPTKP-DYENVAHAVFCQPPLSSVGLTEDQVKANHGEFKIFTS**

16 Pp3c5_16850V3 99.3% 36.6% **QTNVPSIWAIGDVTNRINLTPVALMEGTCFAKTEFGGKPMKP-DYENVASAVFCQPPLSVVGLTEDKAVKQANDILVFTS**

17 164085_Selmo 99.3% 35.8% **RSSVPSVWAIGDVTNRINLTPVALMEGTCFAKTAFGGQATKP-DHENVARAVFCQPPLAVVGLSEEEAVAAAGEVAVYSS**

18 Sacu_s0076g017562 99.3% 35.3% **QTNIPSIWAVGDVTNRINLTPVALMEGTCLAKYLFAGQDVKP-DYENVPCAVFSIPPLSVVGLSEQEAVAKANDIIVYTS**

19 Azfi_s0090g042652 90.7% 32.0% **QTNIPSIWAVGDVTNRINLTPVALMEGTCLAKTLFTEQKVKP-DYENVPCAVFSIPPMSSVGLTEQEAIAQANDIIVYTS**

20 Bradi3g55030 99.3% 34.7% **HTSVPSIWAVGDVTNRINLTPVALMEATCFAKTVFGGQPVKP-DYRDVPCAVFSIPPLSVVGLSEQEALEEANDILVYTS**

21 AT3G24170 99.3% 31.7% **RTNIPSIWAVGDATNRINLTPVALMEATCFANTAFGGKPTKA-EYSNVACAVFCIPPLAVVGLSEEEAVEQAGDILVFTS**

22 P48638_GSHR_NOSS1 96.0% 28.5% **QTSQPNIYAVGDVTDRLNLTPVAIGEGRAFADSEFGNNRREF-SHETIATAVFSNPQASTVGLTEAEARAKLGDVTIYRT**

23 AaOxford_utg000003l_24_1_extN 96.4% 21.5% **RTSQSNIFAVGDCVQPMQFTPLAVAQGRAFADYQFGGQPVVP-GVRVIPAAVSWYPEAATVGLSDAEARARFKDVRCYTN**

24 AaBonn_Sc2ySwM_228_5258_1_extN 96.4% 21.5% **RTSQSNIFAVGDCVQPMQFTPLAVAQGRAFADYQFGGQPVVP-GVRVIPAAVSWYPEAATVGLSDAEARARFKDVRCYTN**

25 Ap_utg000048l_105_1 96.4% 21.3% **RTSQSNIFAVGDCVQPMQFTPLAVAQGRAFADYQFGGQPVVP-GVRVIPAAVSWYPEAATVGLSDAKARARLKDVRCYTN**

26 P06715_GSHR_ECOLIK12 95.0% 23.6% **NTNIEGIYAVGDNTGAVELTPVAVAAGRRLSERLFNNKPDEHLDYSNIPTVVFSHPPIGTVGLTEPQAREQYGDVKVYKS**

cov pid **401**  **. . . . : . . .** **480**

1 G36558_CHBRA44g00300 100.0% 100.0% **----------------------------------C-------------------------------------LYVIRL--**

2 AaOxford_utg000033l_212_1 99.3% 43.7% **NFRPMKATLSGLPDRTFMKVLVDVASDRVVGIHMCGDESPEILQGFAVAVKAGLTKAQFDSTVGIHPTSAEELVTMRTAS**

3 Ap_utg000041l_218_1 99.3% 43.5% **NFRPMKATLSGLPDRTFMKVLVDVASDRVVGIHMCGDESPEILQGFAVAVKAGLTKAQFDSTVGIHPTSAEELVTMRTAS**

4 Mapoly0001s0404 99.3% 43.5% **NFRPMKATISGLGNRTFMKIIVDAASNVVVGVHMCGDDAPEVLQGFGVAVKAGLTKAQFDATVGIHPTAAEELVTMRSPT**

5 Pp3c4_17890V3 99.3% 42.9% **TFRPMKATLSGLPEKTFMKIIVDASSDKVVGIHMCGDETPEILQGFAVAVKAGLTKKMFDSTVGIHPTAAEELVTMRTPT**

6 438142_Selmo 99.3% 41.5% **SFRPMKATLSGLSDRTFIKMIVDCATGKVVGVHMCGEDAGEILQGVGIAVKAGLTKDHFDATVGIHPTSAEEIVTMRSPT**

7 Azfi_s0059g034599 99.7% 41.3% **NFRPMKATLSGLPDRTLMKIIVDVATDKVVGVHMCGEETPEIAQGVAIAVKAELTKAQFDSTVGIHPTAAEELVTMRTPT**

8 Bradi1g74180 99.7% 40.0% **NFRPLRATLSGLPDRVLMKVIVSAATNKVVGVHMCGDDAPEIIQGIAIAVKAGLTKQDFDATVGVHPTSAEEFVTMRNAT**

9 Bradi3g26737 99.7% 38.0% **NFRPLKATLSGLPDRIYTKLIVCADTNKVLGVHMCGEDAPEIIQGIAIAVKAGLTKQNFDATVGVHPTAAEELVTMRNPT**

10 AT3G54660 99.7% 40.5% **NFRPLKATLSGLPDRVFMKLIVCANTNKVLGVHMCGEDSPEIIQGFGVAVKAGLTKADFDATVGVHPTAAEEFVTMRAPT**

11 G21301_CHBRA256g00370 89.4% 29.8% **SFTPMKNTISGRSEKTLMKLIVDSATDKVLGAAMCGPDAAEIMQGIAIAMKCGATKAQFDSTVGIHPSAAEEFVTMRTPT**

12 AaOxford_utg000125l_130_2 97.0% 35.6% **TFNPLKNTVSG-----YLPVCCGQTFSCCCVSHI--NNVLQIFSSFLYSSKAGTS-------------------------**

13 AaBonn_Sc2ySwM_368_3221_corrected 99.3% 33.2% **TFNPLKNTVSGRVEKTFMKLIVDAETDVVLGVHMAGHDAPEIVQGLAIALKCRATKAQFDATVGIHPSAAEEFVTMRTAT**

14 Ap_utg000045l_632_1 63.2% 25.4% **TFNPLKNTVSGRVEKTFMKLIVDAETDVVLGVHMAGHDAPEIVQGLAIALKCRATKAQFDATVGIHPSAAEEFVTMRTAT**

15 Mapoly0002s0333 99.3% 33.6% **SFNPMKNTVSGRQEKTMMKLIVHGATDKVMGAMMCGPDAPEIIQGLAVALKCGATKAQFDATVGIHPTAAEEFVTMRTMT**

16 Pp3c5_16850V3 99.3% 36.6% **SFNPMKNTISGRVEKTFMKLIVDAVTDKVLGAGMVGPDAAEIMQGVAIALKCGATKAQFDATVGIHPTAAEELVTMRTAT**

17 164085_Selmo 99.3% 35.8% **SFTPMKNTISGRQEKSVMKLLVDTGDDRVLGVAMCGPDAPEIMQGISVALKGGATKAQFDSTVGIHPTAAEEFVTMRSVA**

18 Sacu_s0076g017562 99.3% 35.3% **SFNPMKNTISGRQEKTLMKLVVDSLTDKVLGAAMCGPDAPEIIQGIAVALKCGATKAQLDSTVGIHPSAAEEFVTMRSAT**

19 Azfi_s0090g042652 90.7% 32.0% **TFNPMKNTVSGRQEKSLMKLIVDSATDIVLGASMCGPDAPEIIQGIAVALKCRATKAQLDSTVGIHPSAAEEFVTMRTAT**

20 Bradi3g55030 99.3% 34.7% **SFNPMKNSISKRQEKSIMKLVVDSETDRVLGAAMCGPDAAEIMQGIAVALKSGATKATFDSTVGIHPSAAEEFVTMRTLT**

21 AT3G24170 99.3% 31.7% **GFNPMKNTISGRQEKTLMKLIVDEKSDKVIGASMCGPDAAEIMQGIAIALKCGATKAQFDSTVGIHPSSAEEFVTMRSVT**

22 P48638_GSHR_NOSS1 96.0% 28.5% **RFRPMYHSFTGKQERIMMKLVVDTKTDKVLGAHMVGENAAEIIQGVAIAVKMGATKKDFDATVGIHPSSAEEFVTMR---**

23 AaOxford_utg000003l_24_1_extN 96.4% 21.5% **RVTPLEHSLLDRDDKAMIKLVVEGSSDRVVGAHMVGDRAVDIVQCLALAIRLGARKSDFDHTVGIHPSIAEEFFSVS---**

24 AaBonn_Sc2ySwM_228_5258_1_extN 96.4% 21.5% **RVTPLEHSLLDRDDKAMIKLVVEGSSDRVVGAHMVGDRAVDIVQCLALAIRLGARKSDFDHTVGIHPSIAEEFFSVS---**

25 Ap_utg000048l_105_1 96.4% 21.3% **RVTPLEHSLSDRDEKAMIKLVVEGSSDRVVGAHMVGDRAVDIVQCLALAIRLGARKSDFDHTVGIHPSIAEEFFSLY---**

26 P06715_GSHR_ECOLIK12 95.0% 23.6% **SFTAMYTAVTTHRQPCRMKLVCVGSEEKIVGIHGIGFGMDEMLQGFAVALKMGATKKDFDNTVAIHPTAAEEFVTMR---**

cov pid **481**  **]** **483**

1 G36558_CHBRA44g00300 100.0% 100.0% **---**

2 AaOxford_utg000033l_212_1 99.3% 43.7% **RKI**

3 Ap_utg000041l_218_1 99.3% 43.5% **RKI**

4 Mapoly0001s0404 99.3% 43.5% **RKL**

5 Pp3c4_17890V3 99.3% 42.9% **RKI**

6 438142_Selmo 99.3% 41.5% **RKI**

7 Azfi_s0059g034599 99.7% 41.3% **RKI**

8 Bradi1g74180 99.7% 40.0% **RKI**

9 Bradi3g26737 99.7% 38.0% **RKV**

10 AT3G54660 99.7% 40.5% **RKF**

11 G21301_CHBRA256g00370 89.4% 29.8% **RRV**

12 AaOxford_utg000125l_130_2 97.0% 35.6% **---**

13 AaBonn_Sc2ySwM_368_3221_corrected 99.3% 33.2% **RRV**

14 Ap_utg000045l_632_1 63.2% 25.4% **RRV**

15 Mapoly0002s0333 99.3% 33.6% **RQI**

16 Pp3c5_16850V3 99.3% 36.6% **RRV**

17 164085_Selmo 99.3% 35.8% **RFV**

18 Sacu_s0076g017562 99.3% 35.3% **RRV**

19 Azfi_s0090g042652 90.7% 32.0% **RRV**

20 Bradi3g55030 99.3% 34.7% **RRV**

21 AT3G24170 99.3% 31.7% **RRI**

22 P48638_GSHR_NOSS1 96.0% 28.5% **---**

23 AaOxford_utg000003l_24_1_extN 96.4% 21.5% **---**

24 AaBonn_Sc2ySwM_228_5258_1_extN 96.4% 21.5% **---**

25 Ap_utg000048l_105_1 96.4% 21.3% **---**

26 P06715_GSHR_ECOLIK12 95.0% 23.6% **---**

>G36558_CHBRA44g00300

YDYDVLTIGAGSGGVRASRFVAG-FGAKVAVCELPFALTSSDSKGGVGGTCVIRGCVPKKLLVYGSQFAHEF

LASKGFGWSFDKEPSHDWVTLIDNKNKEIQRLTGVYRKLLDGSKVDLLEGRAKLVGPHTVEV----GS-KRY

TAKNILLAVGGRPFVPDIPGKEYGITSDEALELPERPQSICIIGGGYIALEFASIFNALGTETHVLIRQPKV

LRGFDEEIRDFLGEQLQTRGIKIHNRASPVSVEKDADTGKLVL--------------TTDKGEKITTEVVMF

ATGRKPRTERLGAEEVGVELDSSGAIK---------------------------------------------

------------------------------------------------------------------------

--C-------------------------------------LYVIRL-----

>AaOxford_utg000033l_212_1

YDYDLLAIGAGSGGVRASRFAAS-MGARVAICELPFAAVSSDAAGGVGGTCVLRGCVPKKLLVIGANFSHDF

HASEGFGWKLESLPVHDWSALVARKNQEINRLTGVYKSLLAGSGVKLIEGRGKITGAHAVEV----GG-KEY

TAKHILISVGGRATVPDIPGKEYVITSDEALELPELPKSICIVGGGYIALEFAGIFNGLGSDVHVYIRGDKV

LRGFDEEIRDFLADQLKQKGINIHFRETPVAVEKNSN-GSLKL--------------VTNKGSKDA-SAVMF

ATGRAPNTKNLGLEDVGVKLDKKGAIEVDEYSRTSVDSIWAVGDVTNRVNLTPVALMEAMAFSKTVFGDNPT

KP-DHRNIASAVFTNPPIGTVGLTESQAVELYEDVDVFTANFRPMKATLSGLPDRTFMKVLVDVASDRVVGI

HMCGDESPEILQGFAVAVKAGLTKAQFDSTVGIHPTSAEELVTMRTASRKI

>Ap_utg000041l_218_1

YDYDLLAIGAGSGGVRASRFAAS-MGARVAICELPFAAVSSDAAGGVGGTCVLRGCVPKKLLVIGANFSHDF

HASEGFGWKLESLPVHDWSALVARKNQEINRLTGVYKSLLAGSGVKLIEGRGKITGAHAVEV----GG-KEY

TAKHILISVGGRATVPDIPGKEYVITSDEALELPELPKSICIVGGGYIALEFAGIFNGLGSDVHVYIRGDKV

LRGFDEEIRDFLADQLKQKGINIHFGETPVAVEKNSN-GSLKL--------------VTNKGSKDA-SAVMF

ATGRAPNTKNLGLEDVGVKLDKKGAIEVDEYSRTSVDSIWAVGDVTNRVNLTPVALMEAMAFSKTVFGDNPT

KP-DHRNIASAVFTNPPIGTVGLTESQAVELYEDVDVFTANFRPMKATLSGLPDRTFMKVLVDVASDRVVGI

HMCGDESPEILQGFAVAVKAGLTKAQFDSTVGIHPTSAEELVTMRTASRKI

>Mapoly0001s0404

YDYDLVTIGAGSGGVRASRFAAN-LGAKVAVCELPFSTISSDSAGGVGGTCVLRGCVPKKILVYGSQFAHSF

EDSKGFGWSYDAKPKHDWNMLIDNKNKELNRLLGVYKNILKNANVALIEGRGKIVDPHTVDI----SG-ERI

RARHILVAVGGRPYVPDIPGKEFAITSDEALDLPSRPEKICIVGGGYIALEFAGIFNGLGSEVHVFIRQDKV

LRGFDEEVRDFVAEQMSLKGIKFHFGESPTAVEKGSD-GKFTL--------------VTTKGSEVA-DSIMF

ATGRKPNTKNLGLEDVGVNINSKGAIEVDEYSRTSVASIWAVGDVTDRMNLTPVALMEGMAFAKTAFGDEPS

KP-DHRYVASAVFTQPPIGTVGLTEAEALETYGDIDVYTSNFRPMKATISGLGNRTFMKIIVDAASNVVVGV

HMCGDDAPEVLQGFGVAVKAGLTKAQFDATVGIHPTAAEELVTMRSPTRKL

>Pp3c4_17890V3

YDYDLIAIGAGSGGVRAARFASQ-FGANVAVCEMPFAPISSDEAGGVGGTCVLRGCVPKKLLVYGANFPHDF

ESSRGFGWSFETEPKHDWKTLITNKNAELNRLTGVYKSLLQKSEVDLIEGRGKIVDAHTVEV----KG-KQY

TTQHILVSVGGRATVPNIPGKENAITSDEALNLSERPNKICIVGGGYIALEFAGIFAGLGTEVHVFVRQPKV

LRGFDEEIRDFIAAQLQAQGIVFHFGESPTAIEKRND-GTFCL--------------VTDSGKEVS-DLVMF

ATGRAPNTKNLGLEEVGVKLDKRGAIEVDSFSRTNVDSIWAIGDVTNRINLTPVALMEGMAMAKTAFGNEPT

KP-DYRFIASAVFTQPPIGTVGYTEEQAVEKFGDVDVYTSTFRPMKATLSGLPEKTFMKIIVDASSDKVVGI

HMCGDETPEILQGFAVAVKAGLTKKMFDSTVGIHPTAAEELVTMRTPTRKI

>438142_Selmo

FDYDLITIGAGSGGVRASRFAAN-FGARVAVVELPFATISSDEAGGVGGTCVLRGCVPKKLLVYGSSFAHEF

DESKGFGWSYDSPPRHDWKTLMKNKNTELQRLIGVYKSILSSAGVTLVEGRGKILDAHTVQV----SGKEKY

TAKYILVAVGGRSTVPDIPGKEFVITSDHALDLPARPEKICIVGAGYIALEFASIFNGFGSEVHVFLRGPKV

LRGFDDEIRDFVADQMAAKGVKFHFEESPEAVEKCPD-GSLLL--------------RTNKSTEKT-KCVMF

ATGRAPNTKNLGLEDIGVRLGKNGAIMVDEYSKSNVDSIWAVGDVTNRTNLTPVALMEGMAFSKTVFGDRPT

KP-DYNNIPSAVFTQPPIGTVGLTEEQAIKELRNIDVYTSSFRPMKATLSGLSDRTFIKMIVDCATGKVVGV

HMCGEDAGEILQGVGIAVKAGLTKDHFDATVGIHPTSAEEIVTMRSPTRKI

>Azfi_s0059g034599

FDFELITIGGGSGGVRASRFASN-FGAKVALIELPFATISSDSAGGLGGTCVLRGCVPKKLLVYGSKFSHEF

EESRGFGWNFESEPKHDWTTLIAQKNAELRRLLEVYKNILTNAGVSVIEGRGKIVDNHTVEV----NG-KHY

TAKHILVAVGGRAFVPDIVGKEYAITSDEALDLPSRPEKIGIIGGGYISLEFAGIFNSLGSEVHVFIRQNKV

LRGFDEEIRDFIADQMTLRGINFHFEESPVAIEKDST-GQLSL--------------KTNKGSTHSFSHIMF

ATGRRANTKNLGLEAAGVALDQKGAIQVDEYSRTSVESIWAVGDVTDRINLTPVALMEGGAFARTVFGGQAV

KP-DYRNVASAVFTQPPIGTVGLTEEQAIAKYGDVDVYTANFRPMKATLSGLPDRTLMKIIVDVATDKVVGV

HMCGEETPEIAQGVAIAVKAELTKAQFDSTVGIHPTAAEELVTMRTPTRKI

>Bradi1g74180

YDYDLFTIGAGSGGVRASRFASTLYGARAAICEMPFATIATDDLGGLGGTCVLRGCVPKKLLVYASKFSHEF

EESHGFGWTYETDPKHDWSTLIANKNTELQRLVGIYKNILNNAGVDLIEGRGKVVDPHTVSV----DG-KLY

TAKNILIAVGGRPSMPTIPGIDHVIDSDAALDLPSKPEKIAIVGGGYIALEFAGIFNGLKSDVHVFIRQPKV

LRGFDEEVRDFLAEQMSLRGITFHTEQSPQAVTKSSD-GLLSL--------------KTNKETIGGFSHVMF

ATGRKPNTKNLGLEEVGVKMDRNGAIVVDEYSRTSVDSIWAVGDVTDRINLTPVALMEGGAFAKTLFGDEPT

KP-DYRAVPAAVFSQPPIGQVGLTEEQAIEEYGDVDVFLSNFRPLRATLSGLPDRVLMKVIVSAATNKVVGV

HMCGDDAPEIIQGIAIAVKAGLTKQDFDATVGVHPTSAEEFVTMRNATRKI

>Bradi3g26737

YEYDLFTIGAGSGGMRASRFASTLYGARAAVCEMPFATVASDALGGVGGTCVLRGCVPKKLLVYASKYSHEF

DESHGFGWKYETDPKHDWSTLMTRKNLELQRLVDFQTDMLKNSGVTLIEGRGKIVDPHTVSV----DG-KLY

TARNILIAVGARPSIPDIPGIEHVIDSDAALDLPSRPEKIAIVGGGYIALEFAGIFNGLKSEVHVYIRQKKV

LRGFDEEVRDFVTQQMSLRGITFHTEQTPQAITKSDD-GLLSL--------------KTNKGTVNGFSHIMF

ATGRKPNTKNLGLEDVGVKMDKHGSVMVDEYSRTSVDSIWAVGDVTNRVNLTPVALMEGGALTHTIFGNDPI

KP-DHSAVPSAVFSQPPIGQVGLTEEQATEKYGDVDVYTSNFRPLKATLSGLPDRIYTKLIVCADTNKVLGV

HMCGEDAPEIIQGIAIAVKAGLTKQNFDATVGVHPTAAEELVTMRNPTRKV

>AT3G54660

YDFDLFTIGAGSGGVRASRFATS-FGASAAVCELPFSTISSDTAGGVGGTCVLRGCVPKKLLVYASKYSHEF

EDSHGFGWKYETEPSHDWTTLIANKNAELQRLTGIYKNILSKANVKLIEGRGKVIDPHTVDV----DG-KIY

TTRNILIAVGGRPFIPDIPGKEFAIDSDAALDLPSKPKKIAIVGGGYIALEFAGIFNGLNCEVHVFIRQKKV

LRGFDEDVRDFVGEQMSLRGIEFHTEESPEAIIKAGD-GSFSL--------------KTSKGTVEGFSHVMF

ATGRKPNTKNLGLENVGVKMAKNGAIEVDEYSQTSVPSIWAVGDVTDRINLTPVALMEGGALAKTLFQNEPT

KP-DYRAVPCAVFSQPPIGTVGLTEEQAIEQYGDVDVYTSNFRPLKATLSGLPDRVFMKLIVCANTNKVLGV

HMCGEDSPEIIQGFGVAVKAGLTKADFDATVGVHPTAAEEFVTMRAPTRKF

>G21301_CHBRA256g00370

LEWE-------AEGLEA-------RGWQQAMEP----------------RCVLRGCVPKKILVYGSQFGSEF

EDAEGYGWKL-EKPEFDWKKLIVGKTKEIERLNGIYKRILANAGVTLIEGEGKLLDAHTVQVTPPAGEVKYI

TAKHILIATGSRAAVLNIPGKEYGITSDEALSLEQFPKRVVVVGGGYIAVEFAGIFKGLGSEVHIMYRKELP

LRGFDQEMRVIVAENLKARGIKVHAGLNPKRIEKTAD-G-LVV--------------TTDKGEKLVGDVVMF

ATGRKPNVRRLNLKAASIATDEVGAIVVNEYSQTNVPNIWAVGDVTNRINLTPVALMEGMAFAKTVFGRQPT

KP-DYQFVPSAVFCQPPMAVVGYTEDEAVAEGGDIVVYTSSFTPMKNTISGRSEKTLMKLIVDSATDKVLGA

AMCGPDAAEIMQGIAIAMKCGATKAQFDSTVGIHPSAAEEFVTMRTPTRRV

>AaOxford_utg000125l_130_2

FDYDLFVIGAGSGGVRASRIAAG-FGAKVAVVELAFHPVSSEALGGIGGTCVLRGCVPKKILVYGSEFHDQF

EDARNYGWNLDETITFDWKKLIASKHREITRLNGIYKKMLAGAGVTVYEGEGKFLDRHTVQITEPSGEVKTY

RSKHILIATGARAVTLNIPGKELAITSDEALSLEKFPKRAVIVGAGYIAVEFSGIFSGLGSKVDLFFRSDAP

LRGFDDEIRGAVATHLQNRGVQLHPGTNITKIEKVED-G-LKV--------------TTDKGEEFVTDEVLF

ATGRKPNSHRLNLEAVGVALDQQGGIKVNEFSQTNVPNIWAVGDVTNRIQLTPVALMEGHCFAKTVFGGQPS

KP-DYRDVACAVFCQPPVSVVGLTEKDALEQSGDILVFTSTFNPLKNTVSG-----YLPVCCGQTFSCCCVS

HI--NNVLQIFSSFLYSSKAGTS----------------------------

>AaBonn_Sc2ySwM_368_3221_corrected

FDYDLFVIGAGSGGVRASRIAAG-FGAKVAVVELAFHPVSSEALGGIGGTCVLRGCVPKKILVYGSEFHDQF

EDARNYGWNLDETITFDWKKLIASKHREITRLNGIYKKMLAGAGVTVYEGEGKFLDRHTVQITEPSGEVKTY

RSKHILIATGARAVTLNIPGKELAITSDEALSLEKFPKRAVIVGAGYIAVEFSGIFSGLGSKVDLFFRSDAP

LRGFDDEIRGAVATHLQNRGVQLHPGTNITKIEKVED-G-LKV--------------TTDKGEEFVTDEVLF

ATGRKPNSHRLNLEAVGVALDQQGGIKVNEFSQTNVPNIWAVGDVTNRIQLTPVALMEGHCFAKTVFGGQPS

KP-DYRDVACAVFCQPPVSVVGLTEKDALEQSGDILVFTSTFNPLKNTVSGRVEKTFMKLIVDAETDVVLGV

HMAGHDAPEIVQGLAIALKCRATKAQFDATVGIHPSAAEEFVTMRTATRRV

>Ap_utg000045l_632_1

------------------------------------------------------------------------

--------------------------------------MLAGAGVTVYEGEGKFLDRHTVQVTEPSGEVKTY

RSKHILIATGARAVTLNIPGKELAITSDEALSLEKFPKRAVIVGAGYIAVEFSGIFSGLGSKVDLFFRSDAP

LRGFDDEIRGAVATHLQNRGVQLHPGTNITKIEKVED-G-LKV--------------TTDKGEEFVTDEVLF

ATGRKPNSHRLNLEAVGVALDQQGGIKVNEFSQTNVPNIWAVGDVTNRIQLTPVALMEGHCFAKTVFGGQPS

KP-DYRDVACAVFCQPPVSVVGLTEKDALEQSGDILVFTSTFNPLKNTVSGRVEKTFMKLIVDAETDVVLGV

HMAGHDAPEIVQGLAIALKCRATKAQFDATVGIHPSAAEEFVTMRTATRRV

>Mapoly0002s0333

FDYDLFVIGGGSGGVRASRMSAS-HGAKVALVELPYHPISSETHGGLGGTCVLRGCVPKKILVYGSGFSHEF

QDAKGFGWEIDGEIRFNWKKLIQNKTKEIERLNGVYKRMLVNAGVTLFEGFGRIVDKHTVEVKDVDGQTKTF

TTNHILIATGGKAVKLNIPGQELGITSDEGLSLEEFPKKVLIIGGGYIAVEFAGIYSGMGADVHLCYRKSLP

LAGFDTEMAEVVARNLEARGIKCHKHTNIVKLESHYL-G-IKA--------------TTDTNEEYHVDQVMF

ATGREPKTANLNLEGVGVELDEAGAIKVNEYSQTSIPSIWAVGDVTNRINLTPVALMEGTCFAKTVFLKEPT

KP-DYENVAHAVFCQPPLSSVGLTEDQVKANHGEFKIFTSSFNPMKNTVSGRQEKTMMKLIVHGATDKVMGA

MMCGPDAPEIIQGLAVALKCGATKAQFDATVGIHPTAAEEFVTMRTMTRQI

>Pp3c5_16850V3

YDYDLFVIGAGSGGVRASRTAAG-FGAKVAICELPYHPISSESAGGIGGTCVLRGCVPKKILVYGSAFGGEF

QDAREFGWNINGDITFDWKRLIANKTKEIIRLNGVYKRLLAGSKVDMYEGGGKIVDPHTVDVEQTGGEVKRF

TAKKILVATGGRAVPLNIPGKELAITSDEGLSLEEFPKRVVIAGGGYIAVEFAGIYSGMGAKVDLFYRKPLP

LTGFDEEMREVVARNLENRGIKCHPETNLTKLEKVAG-G-IKV--------------TVDNGEEHEVDAVMF

ATGRKPSTKNIGLEDVGVELDKTGAIKVNEYSQTNVPSIWAIGDVTNRINLTPVALMEGTCFAKTEFGGKPM

KP-DYENVASAVFCQPPLSVVGLTEDKAVKQANDILVFTSSFNPMKNTISGRVEKTFMKLIVDAVTDKVLGA

GMVGPDAAEIMQGVAIALKCGATKAQFDATVGIHPTAAEELVTMRTATRRV

>164085_Selmo

FDFDLFTIGAGSGGVRASRTAAN-FGAKVAVVELPFAHVSSESAGGVGGTCVIRGCVPKKILVYASMFSAEF

QDSKNFGWNVPDGITFEWKRLIANKDREIERLNGIYKRLLTGSGVTILEGRASLVDDHTVEVSHSDGSSKRY

RAKHILVATGSRAVRLNVPGKELAITSDEGLNLDELPRRCVIVGGGYIAVEFAGIYSGMGSKVELLYRKKTP

LRGFDDEMRAVVARNLENRGVHLRPDTNVTKIEKVG--GELKV--------------SIDNGGEIMTDAVLF

AVGRKPKTSGLNLEELGVELDKSGAIKVDEYSRSSVPSVWAIGDVTNRINLTPVALMEGTCFAKTAFGGQAT

KP-DHENVARAVFCQPPLAVVGLSEEEAVAAAGEVAVYSSSFTPMKNTISGRQEKSVMKLLVDTGDDRVLGV

AMCGPDAPEIMQGISVALKGGATKAQFDSTVGIHPTAAEEFVTMRSVARFV

>Sacu_s0076g017562

FDFDLFTIGAGSGGVRASRMSSG-YGAKVAICELPFHPISSEGLGGCGGTCVIRGCVPKKILVYGASFKGEF

EDSKNFGWDIDGEITFDWKRLIENKTKEITRLNGVYKRILSGAGVTFIEGAGKVIDPHTVEVQLPDGQLKRF

TTKHILIATGSRAVLIDIPGKELAITSDEALSLEEFPKRSVIVGGGYIAVEFAGIYNAMGSKVDLFYRKDAP

LRGFDDEMRITVAKNLEGRGVNLHPKANIIKIEKTEG-G-LKA--------------YTDKGDVFETDVVLM

AVGRKPNIKRLNLESVGVELHTDDAIKVNEYSQTNIPSIWAVGDVTNRINLTPVALMEGTCLAKYLFAGQDV

KP-DYENVPCAVFSIPPLSVVGLSEQEAVAKANDIIVYTSSFNPMKNTISGRQEKTLMKLVVDSLTDKVLGA

AMCGPDAPEIIQGIAVALKCGATKAQLDSTVGIHPSAAEEFVTMRSATRRV

>Azfi_s0090g042652

---------------------------MVAICELPFHPISSEGLGGCGGTCVIRGCVPKKILVYGAAFKGEF

EDSKNFGWDINGEITFDWKRLIENKTKEITRLNGIYKRILTGAGVTLIEGAGRVIDPHTVEVQYPNGQSKRF

TTRHILIATGGRAVLLDIPGKELAITSDEALSLEEFPKRSVIVGGGYIAVEFAGIYNGMGSKVNLFYRKDAP

LRGFDDEMRVAVAKNLENRGVVLHPSTNIVKIEKVES-G-LKV--------------YTDKGDVLETDVVLM

AVGRAPNVNKLNLDVVGVELHGDGAIKVNEYSQTNIPSIWAVGDVTNRINLTPVALMEGTCLAKTLFTEQKV

KP-DYENVPCAVFSIPPMSSVGLTEQEAIAQANDIIVYTSTFNPMKNTVSGRQEKSLMKLIVDSATDIVLGA

SMCGPDAPEIIQGIAVALKCRATKAQLDSTVGIHPSAAEEFVTMRTATRRV

>Bradi3g55030

YEYDLFVIGAGSGGVRGSRTSAG-FGAKVAICELPFHPISSEWQGGHGGTCVIRGCVPKKILVYGAAFRGEF

EDAKNFGWEINGDINYNWKKLLENKTQEIVRLNGVYKRILGNSGVTMIEGAGSVVDAHTVEVTQPDGSKQRH

TAKHILIATGSRAHLVDIPGKELAITSDEALSLEELPKRAVILGGGYIAVEFASIWKGLGAEVDLFYRKELP

LRGFDDEMRTVVASNLEGRGIRLHPATNLTELSKTAD-G-IKV--------------VTDKGDELMADVVLF

ATGRTPNTNKLNLEAVGVEVDQIGAIKVDEFSHTSVPSIWAVGDVTNRINLTPVALMEATCFAKTVFGGQPV

KP-DYRDVPCAVFSIPPLSVVGLSEQEALEEANDILVYTSSFNPMKNSISKRQEKSIMKLVVDSETDRVLGA

AMCGPDAAEIMQGIAVALKSGATKATFDSTVGIHPSAAEEFVTMRTLTRRV

>AT3G24170

YDFDLFVIGAGSGGVRAARFSAN-HGAKVGICELPFHPISSEEIGGVGGTCVIRGCVPKKILVYGATYGGEL

EDAKNYGWEINEKVDFTWKKLLQKKTDEILRLNNIYKRLLANAAVKLYEGEGRVVGPNEVEVRQIDGTKISY

TAKHILIATGSRAQKPNIPGHELAITSDEALSLEEFPKRAIVLGGGYIAVEFASIWRGMGATVDLFFRKELP

LRGFDDEMRALVARNLEGRGVNLHPQTSLTQLTKTDQ-G-IKV--------------ISSHGEEFVADVVLF

ATGRSPNTKRLNLEAVGVELDQAGAVKVDEYSRTNIPSIWAVGDATNRINLTPVALMEATCFANTAFGGKPT

KA-EYSNVACAVFCIPPLAVVGLSEEEAVEQAGDILVFTSGFNPMKNTISGRQEKTLMKLIVDEKSDKVIGA

SMCGPDAAEIMQGIAIALKCGATKAQFDSTVGIHPSSAEEFVTMRSVTRRI

>P48638_GSHR_NOSS1

FDYDLFVIGAGSGGLAASKRAAS-YGAKVAIAE----------NDLVGGTCVIRGCVPKKLMVYGSHFPALF

EDAAGYGWQV-GKAELNWEHFITSIDKEVRRLSQLHISFLEKAGVELISGRATLVDNHTVEV----GE-RKF

TADKILIAVGGRPIKPELPGMEYGITSNEIFHLKTQPKHIAIIGSGYIGTEFAGIMRGLGSQVTQITRGDKI

LKGFDEDIRTEIQEGMTNHGIRIIPKNVVTAIEQVPEGLKISL--------------SGEDQEPIIADVFLV

ATGRVPNVDGLGLENAGVDVSTMNAIAVNEYSQTSQPNIYAVGDVTDRLNLTPVAIGEGRAFADSEFGNNRR

EF-SHETIATAVFSNPQASTVGLTEAEARAKLGDVTIYRTRFRPMYHSFTGKQERIMMKLVVDTKTDKVLGA

HMVGENAAEIIQGVAIAVKMGATKKDFDATVGIHPSSAEEFVTMR------

>AaOxford_utg000003l_24_1_extN

YKYHLLVVGGGPGGLAAAKRVAR-RGYRVALAE----------QDTVGGTCVMRGCVPEKLMTFAAKFPEAC

RDAVSYGWSQECSQNFDWPRFIEAKEQETRRLSQVHRQALEKAGVELLRGRATFINAHTVDV----GG-AEV

TADTILIAVGAIPVPHDVPGIQHAVTNSELFVLPEQPREMAIMGGDYIAVKLGGIMHNLGSRLTHVVPEESV

LATFDQDLCEGVTDGITKRGITVLNRCKVEEIRKVEEEGGGKLLEVVVGPVGDGEIQEKEKCRTLRVDTVLD

GMLRLPNIEDLGLEVAGVRVTARRCIAVDEHCRTSQSNIFAVGDCVQPMQFTPLAVAQGRAFADYQFGGQPV

VP-GVRVIPAAVSWYPEAATVGLSDAEARARFKDVRCYTNRVTPLEHSLLDRDDKAMIKLVVEGSSDRVVGA

HMVGDRAVDIVQCLALAIRLGARKSDFDHTVGIHPSIAEEFFSVS------

>AaBonn_Sc2ySwM_228_5258_1_extN

YKYHLLVVGGGPGGLAAAKRVAR-RGYRVALAE----------QDTVGGTCVMRGCVPEKLMTFAAKFPEAC

RDAVSYGWSQECSQNFDWPRFIEAKEQETRRLSQVHRQALEKAGVELLRGRATFINAHTVDV----GG-AEV

TADTILIAVGAIPVPHDVPGIQHAVTNSELFVLPEQPREMAIMGGDYIAVKLGGIMHNLGSRLTHVVPEESV

LATFDQDLCEGVTDGITKRGITVLNRCKVEEIRKVEEEGGGKLLEVVVGPVGDGEIQEKEKCRTLRVDTVLD

GMLRLPNIEDLGLEVAGVRVTARRCIAVDEHCRTSQSNIFAVGDCVQPMQFTPLAVAQGRAFADYQFGGQPV

VP-GVRVIPAAVSWYPEAATVGLSDAEARARFKDVRCYTNRVTPLEHSLLDRDDKAMIKLVVEGSSDRVVGA

HMVGDRAVDIVQCLALAIRLGARKSDFDHTVGIHPSIAEEFFSVS------

>Ap_utg000048l_105_1

YKYHLLVVGGGPGGLAAAKRVAR-QGYRVALAE----------QDTVGGTCVMRGCVPEKLMTFAAKFPEAC

RDAVSYGWSQECSQNFDWPRFIEAKEQETRRLSQVHRQALEKAGVELLRGRATFINAHTVDV----GG-AEV

TADTILIAVGAIPVPHDVPGVQHAVTNSELFVLPEQPREMAIMGGDYIAVKLGGIMHNLGSRVTHVVPEESV

LATFDQDLCEGVTDGITKRGITVLSRCKVEEIRKVEEEGGGKLLEVVVGPASDGEIQEKEKCRTLRVDTVLD

GMLRLPNIEDLGLEAAGVRVAARRCIAVDEHCRTSQSNIFAVGDCVQPMQFTPLAVAQGRAFADYQFGGQPV

VP-GVRVIPAAVSWYPEAATVGLSDAKARARLKDVRCYTNRVTPLEHSLSDRDEKAMIKLVVEGSSDRVVGA

HMVGDRAVDIVQCLALAIRLGARKSDFDHTVGIHPSIAEEFFSLY------

>P06715_GSHR_ECOLIK12

KHYDYIAIGGGSGGIASINRAAM-YGQKCALIE----------AKELGGTCVNVGCVPKKVMWHAAQIR---

EAIHMYGPDYTTINKFNWETLIASRTAYIDRIHTSYENVLGKNNVDVIKGFARFVDAKTLEV----NG-ETI

TADHILIATGGRPSHPDIPGVEYGIDSDGFFALPALPERVAVVGAGYIAVELAGVINGLGAKTHLFVRKHAP

LRSFDPMISETLVEVMNAEGPQLHTNAIPKAVVKNTD-GSLTL--------------ELEDGRSETVDCLIW

AIGREPANDNINLEAAGVKTNEKGYIVVDKYQNTNIEGIYAVGDNTGAVELTPVAVAAGRRLSERLFNNKPD

EHLDYSNIPTVVFSHPPIGTVGLTEPQAREQYGDVKVYKSSFTAMYTAVTTHRQPCRMKLVCVGSEEKIVGI

HGIGFGMDEMLQGFAVALKMGATKKDFDNTVAIHPTAAEEFVTMR------
