# Supplementary material for: Plasticity in plastid redox networks: evolution of glutathione-dependent redox cascades and glutathionylation sites
Source: BMC Plant Biol. 2021 Jul 5;21:322. doi: 10.1186/s12870-021-03087-2 (PMC8256493; doi:10.1186/s12870-021-03087-2)
Supplement: Supplementary file 13 — Additional file 13. Word-file containing phylogenetic trees generated with alternative method (Maximum Likelihood). [file 12870_2021_3087_MOESM13_ESM.docx]

**Additional File 13**

**Phylogenetic trees generated with alternative method (Maximum Likelihood)**


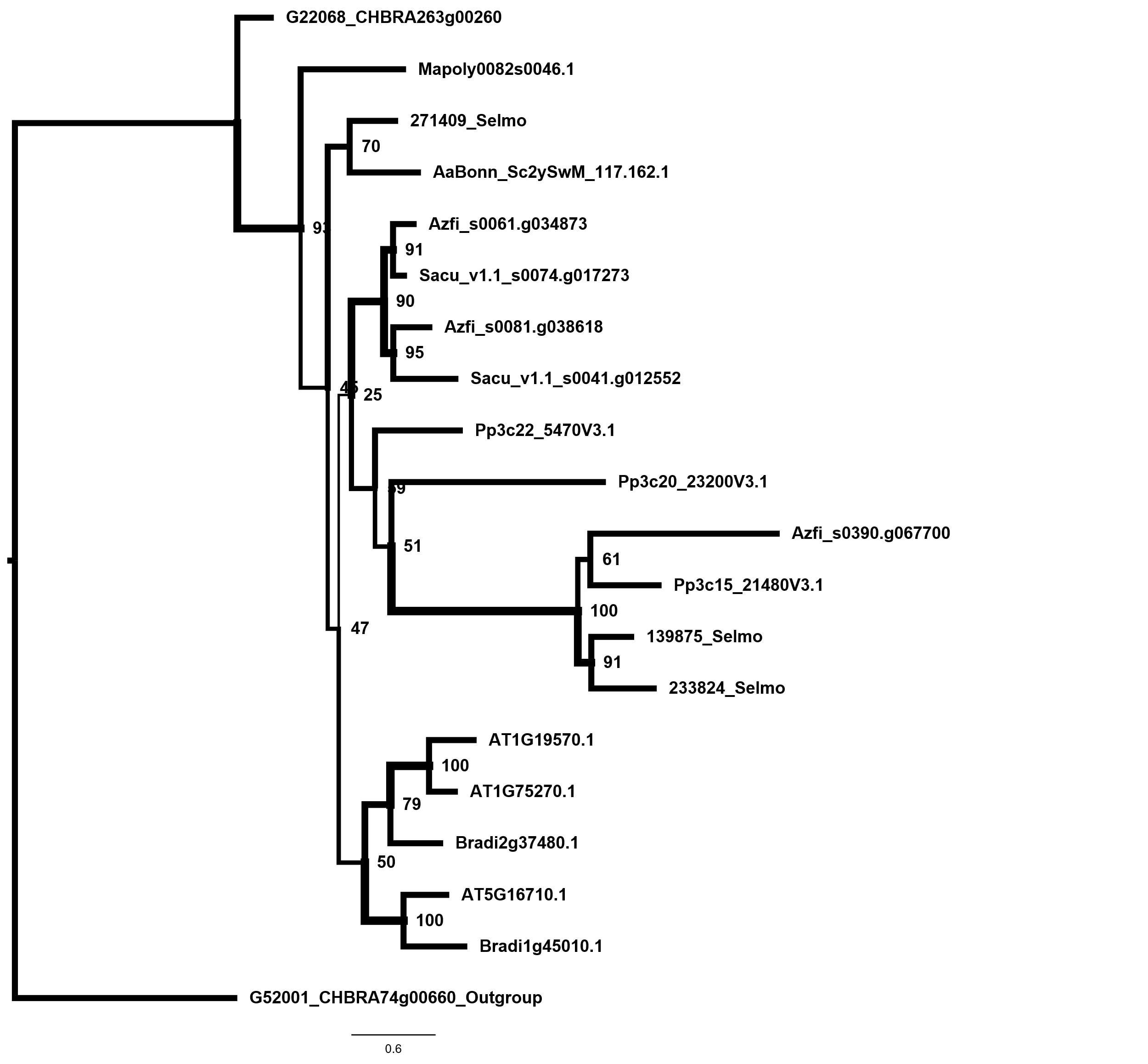
**Phylogenetic tree of DHAR (Dehydroascorbate reductase)**

Phylogenetic tree of DHAR isoforms (P. patens nomenclature according to Liu et al., 2013) constructed with Maximum Likelihood using iQtree, node values and line weights depict bootstrap values (1000) The model WAG+G4 was used. P. patens DHAR1 was identified and quantified in mitochondrial and plastid proteomes (Mueller et al., 2014) and is putatively dual targeted. The used gene models are Chara braunii (CHBRA), Marchantia polymorpha (Mapoly), Physcomitrella patens (Pp), Selaginella moellendorfii (Selmo), Salvinia cucullata (Sacu), Azolla filiculoides (Azfi), Brachipodium distachyon (Bradi) and Arabidopsis thaliana (At)


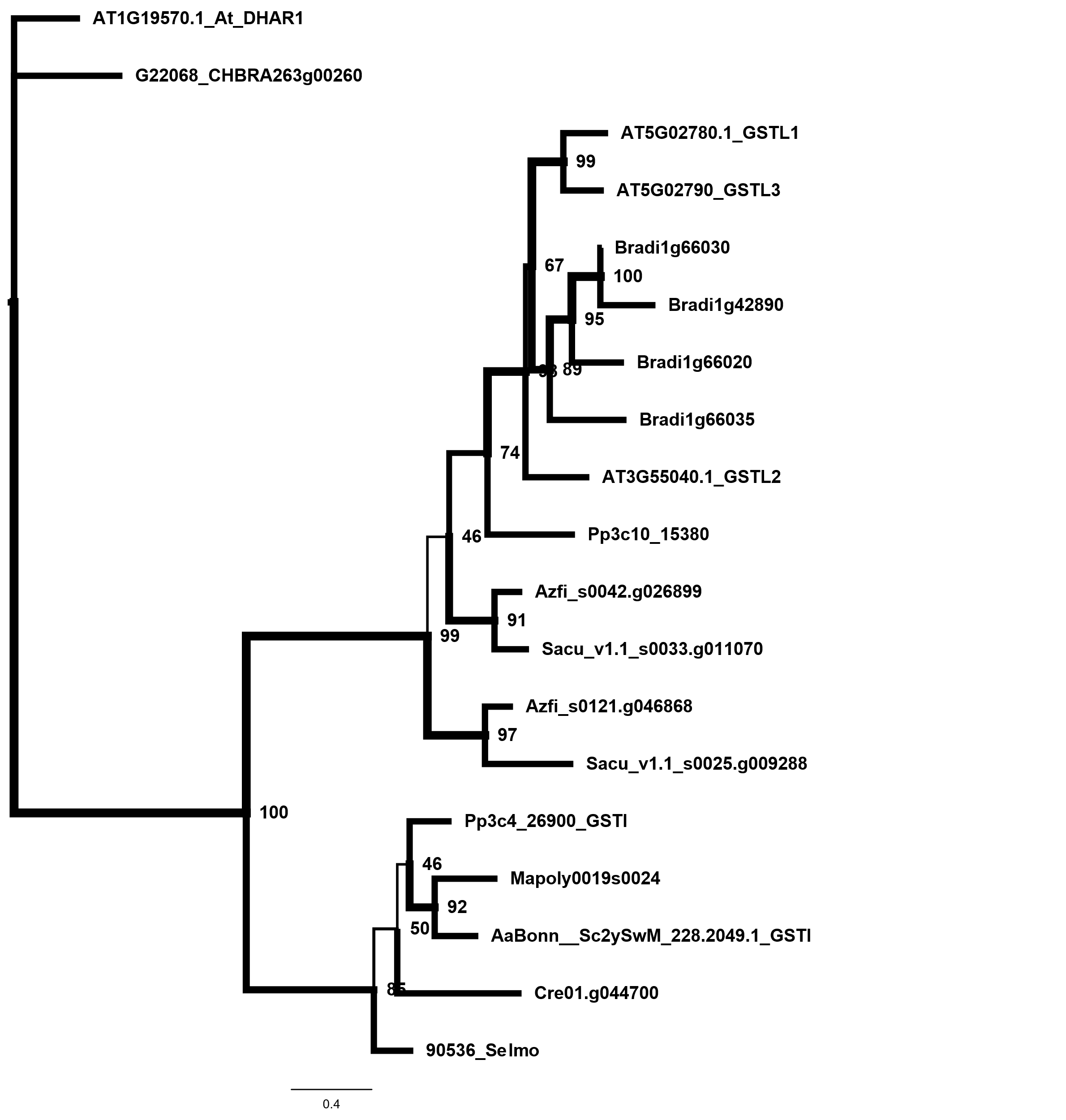


**Phylogenetic tree of GSTI/GSTL** **(Glutathione S-transferase I and L)**

Phylogenetic tree of GSTI/GSTL isoforms constructed with Maximum Likelihood using iQtree, node values and line weights depict bootstrap values (1000) The model LG+G4 was used. The gene models shown are Chara braunii (CHBRA), Marchantia polymorpha (Mapoly), Physcomitrella patens (Pp), Selaginella moellendorfii (Selmo), Salvinia cucullata (Sacu), Azolla filiculoides (Azfi), Brachipodium distachyon (Bradi) and Arabidopsis thaliana (At)


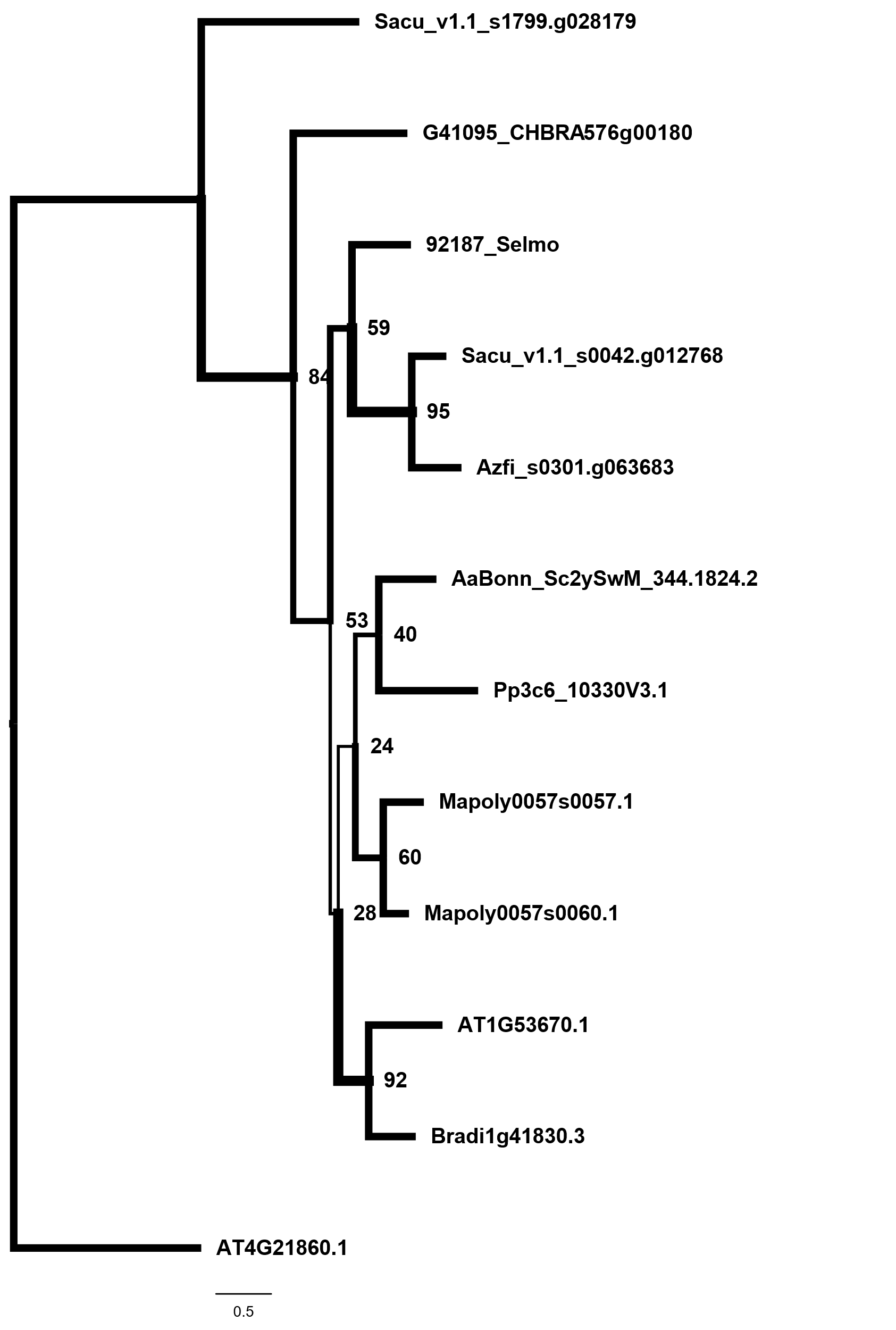


**Phylogenetic tree of MSRB1 (Methionine sulfoxide reductase B)**

Phylogenetic tree of MSRB1 isoforms constructed with Maximum Likelihood using iQtree, node values and line weights depict bootstrap values (1000) The model WAG+G4 was used. The gene models shown are Chara braunii (CHBRA), Marchantia polymorpha (Mapoly), Physcomitrella patens (Pp), Selaginella moellendorfii (Selmo), Salvinia cucullata (Sacu), Azolla filiculoides (Azfi), Brachipodium distachyon (Bradi) and Arabidopsis thaliana (At)


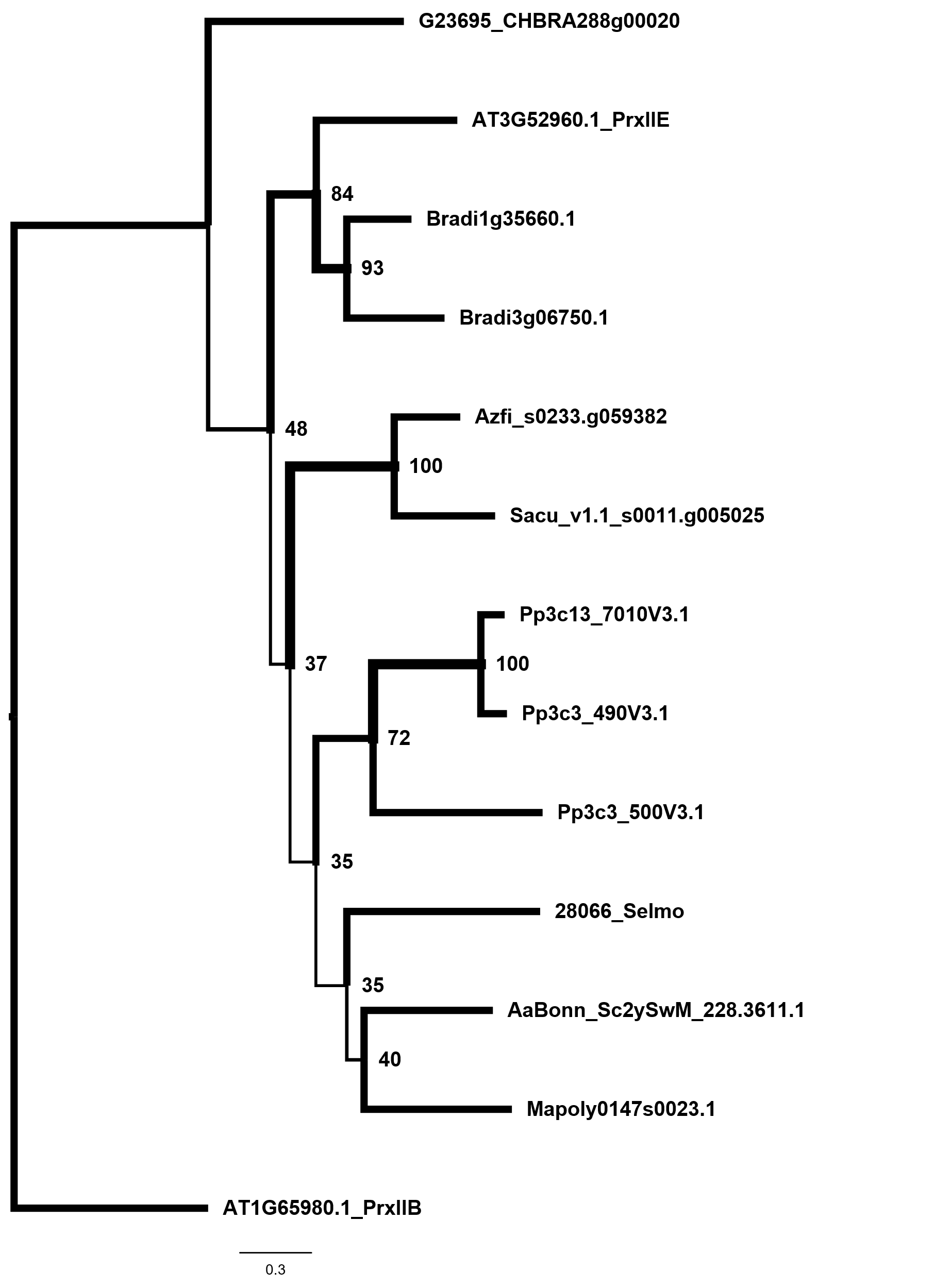


**Phylogenetic tree of PrxIIE (Peroxiredoxin IIE)**

Phylogenetic tree of PrxIIE isoforms constructed with Maximum Likelihood using iQtree, node values and line weights depict bootstrap values (1000) The model WAG+RF+G4 was used. The gene models shown are Chara braunii (CHBRA), Marchantia polymorpha (Mapoly), Physcomitrella patens (Pp), Selaginella moellendorfii (Selmo), Salvinia cucullata (Sacu), Azolla filiculoides (Azfi), Brachipodium distachyon (Bradi) and Arabidopsis thaliana (At)


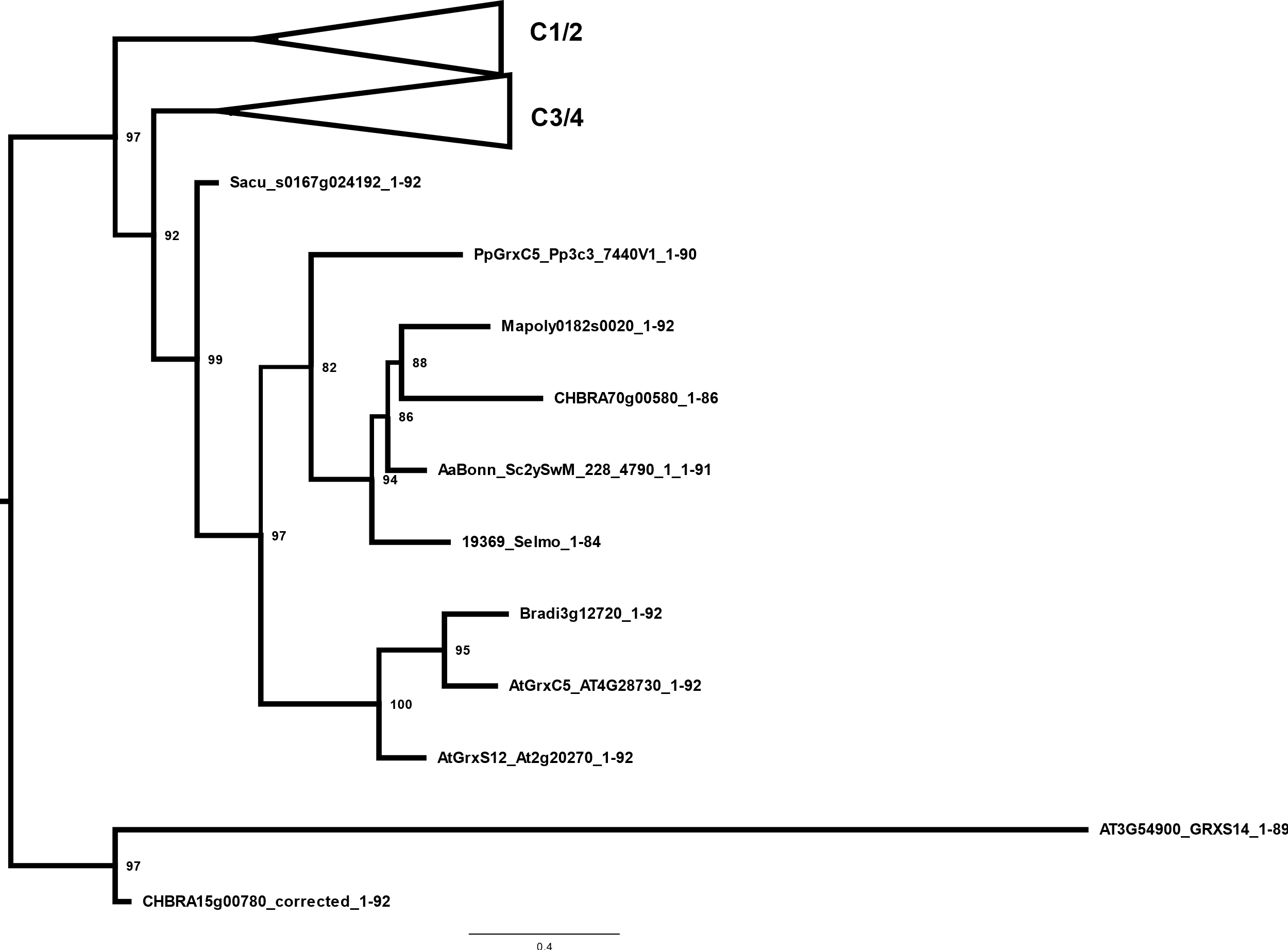


**Phylogenetic tree of GRX Class I (Glutaredoxins class I)**

Phylogenetic tree of GRX Class I isoforms constructed with Maximum Likelihood using iQtree, node values and line weights depict bootstrap values (1000) The model LG+G4 was used. The gene models shown are Chara braunii (CHBRA), Marchantia polymorpha (Mapoly), Physcomitrella patens (Pp), Selaginella moellendorfii (Selmo), Salvinia cucullata (Sacu), Azolla filiculoides (Azfi), Brachipodium distachyon (Bradi) and Arabidopsis thaliana (At)


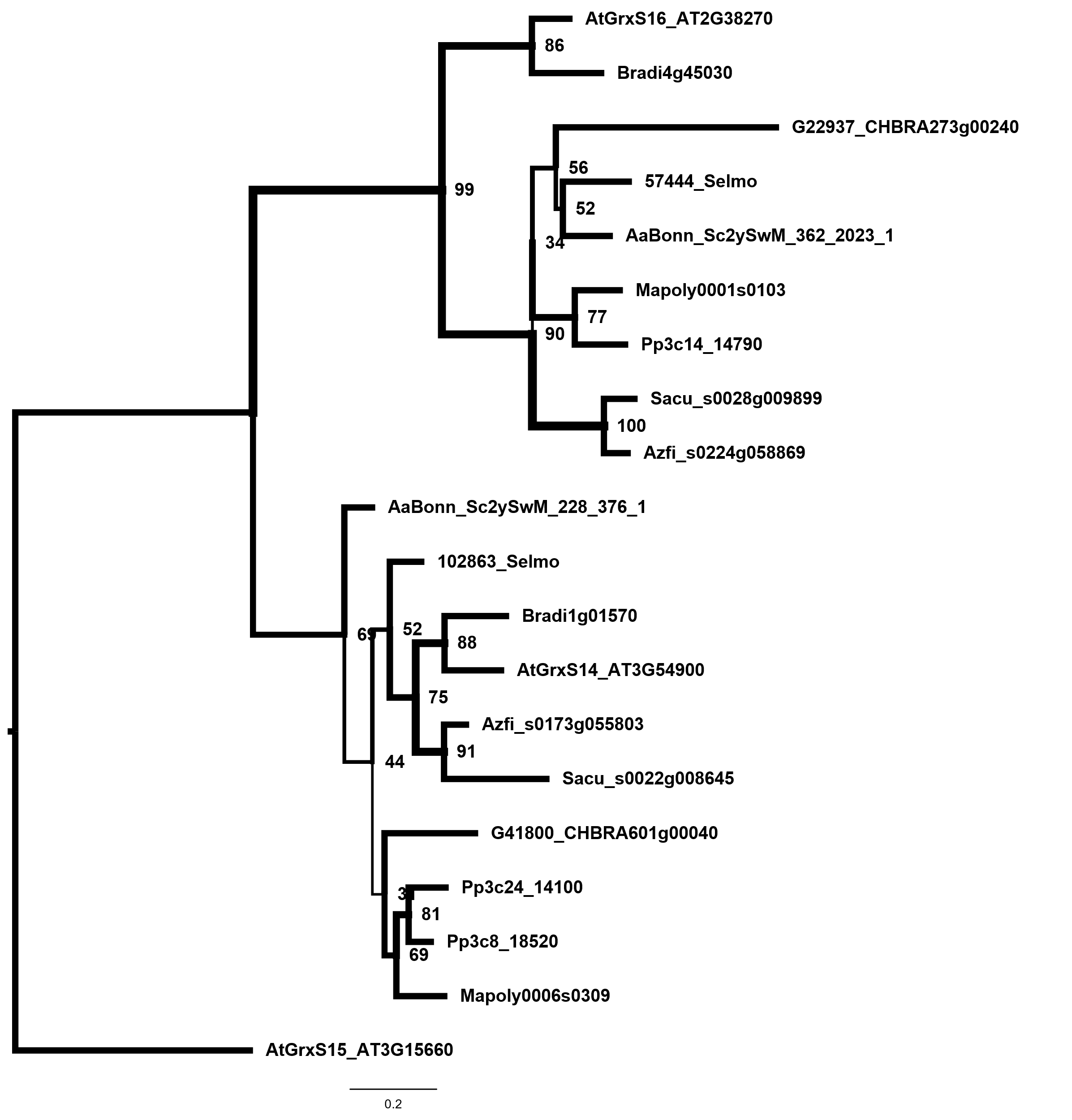


**Phylogenetic tree of GRX14 and GRXS16 (Glutaredoxin S14/16)**

Phylogenetic tree of GRXS14/S16 isoforms constructed with Maximum Likelihood using iQtree, node values and line weights depict bootstrap values (1000) The model LG+I+G4 was used. The gene models shown are Chara braunii (CHBRA), Marchantia polymorpha (Mapoly), Physcomitrella patens (Pp), Selaginella moellendorfii (Selmo), Salvinia cucullata (Sacu), Azolla filiculoides (Azfi), Brachipodium distachyon (Bradi) and Arabidopsis thaliana (At)

**
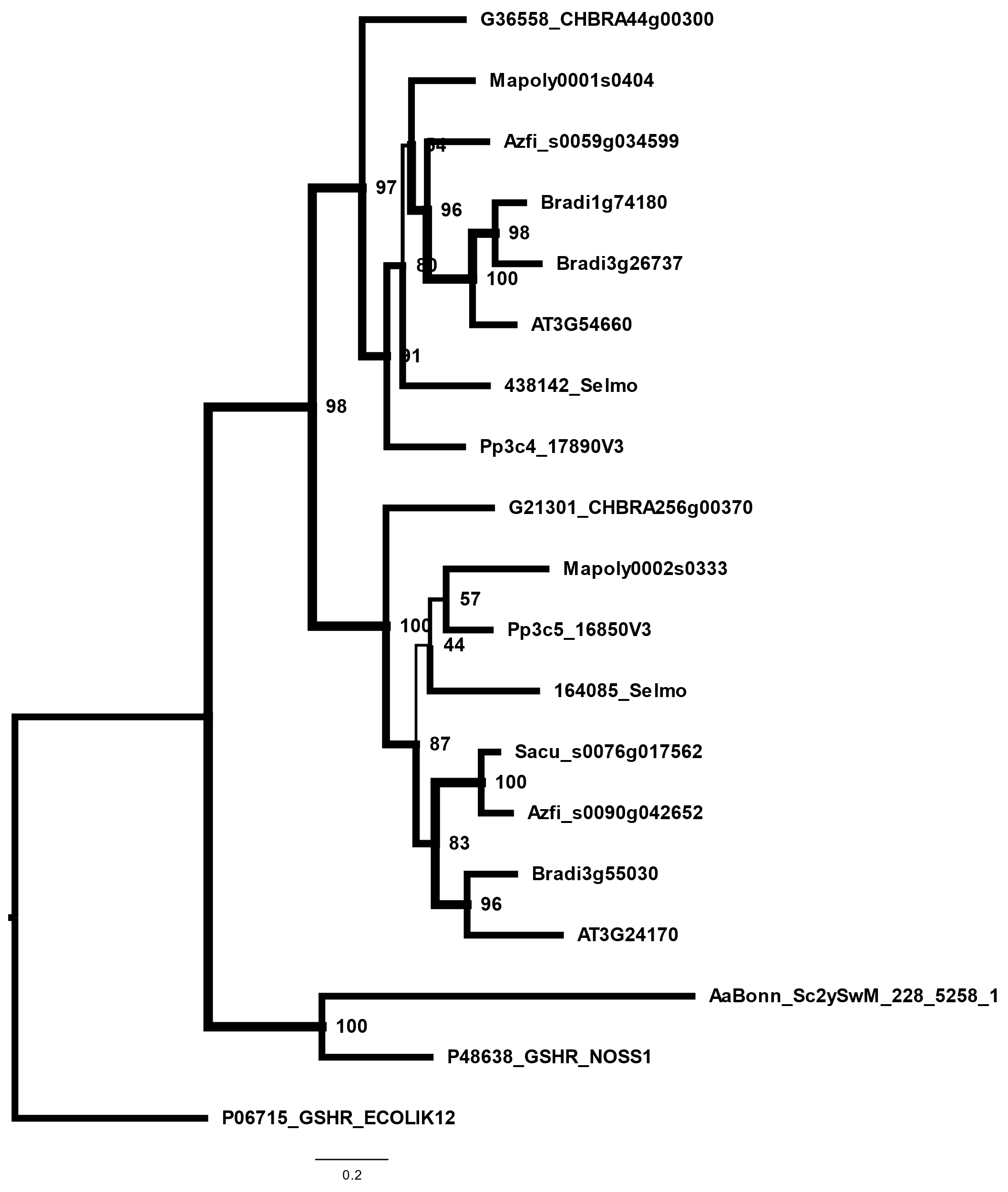
**

**Phylogenetic tree of GR (glutathione reductase)**

Phylogenetic tree of GR isoforms constructed with Maximum Likelihood using iQtree, node values and line weights depict bootstrap values (1000) The model LG+G4 was used. The gene models shown are Chara braunii (CHBRA), Marchantia polymorpha (Mapoly), Physcomitrella patens (Pp), Selaginella moellendorfii (Selmo), Salvinia cucullata (Sacu), Azolla filiculoides (Azfi), Brachipodium distachyon (Bradi) and Arabidopsis thaliana (At)
