# Supplementary material for: Plasticity in plastid redox networks: evolution of glutathione-dependent redox cascades and glutathionylation sites
Source: BMC Plant Biol. 2021 Jul 5;21:322. doi: 10.1186/s12870-021-03087-2 (PMC8256493; doi:10.1186/s12870-021-03087-2)
Supplement: Supplementary file 8 — Additional file 8: Fig. S8. Phylogenetic tree of glutathione reductases (GR). (a) Phylogenetic tree of glutathione reductase isoforms constructed using MrBayes, node values and line weights depict posterior probabilities; nodes with lower support than 50% are collapsed (run parameters: mixed protein models, rates = invgamma, number of generations: 0.5*106, burnin = 20%, split frequencies< 0.01). TargetP2.0 (T) [46], LOCALIZER (L) [47] and PredAlgo (P) [48] targeting predictions (Additional file 1 Table S1) are indicated (M, mitochondria; P, plastid; O, other; S, secretory); experimental evidence for conserved dual targeting of GR2 isoforms:1 [67], 2 [29]. The presence (check mark) or absence (X) of an N-terminal extension (ext. N) in the sequence is indicated. Gene identifiers are given according to the used gene models for Chlamydomonas reinhardtii (Cre), Chara braunii (CHBRA), Anthoceros agrestis strain Bonn (AaBonn), Marchantia polymorpha (Mapoly), Physcomitrium patens (Pp), Selaginella moellendorffii (Selmo), Salvinia cucullata (Sacu), Azolla filiculoides (Azfi), Brachypodium distachyon (Bradi) and Arabidopsis thaliana (At) and are additionally color-coded as in Fig. 1. Additional species: NOSS: Nostoc spec.; Anthoceros agrestis Oxford (AaOxford), Anthoceros punctatus (Ap), [40]. Colour legend: Cb = Chara braunii; Aa = Anthoceros agrestis; Mp = Marchantia polymorpha; Pp = Physcomitrium patens; Sm = Selaginella moellendorffii; Sc = Salvinia cucullata; Af = Azolla filiculoides; Bd = Brachypodium distachyon; At = Arabidopsis thaliana. (b) N-terminal part of GR protein alignment (Jalview) showing the presence or absence of N-terminal extensions indicative of putative N-terminal targeting peptides. Red bar marks prominent example of sequence lacking in bacterial and Anthoceros GRs, supporting an origin by horizontal gene transfer (HGT). GRs acquired by HGT possess putative N-terminal extensions, compared to bacterial GRs. Colour-scheme: ClustalX. [file 12870_2021_3087_MOESM8_ESM.pdf]

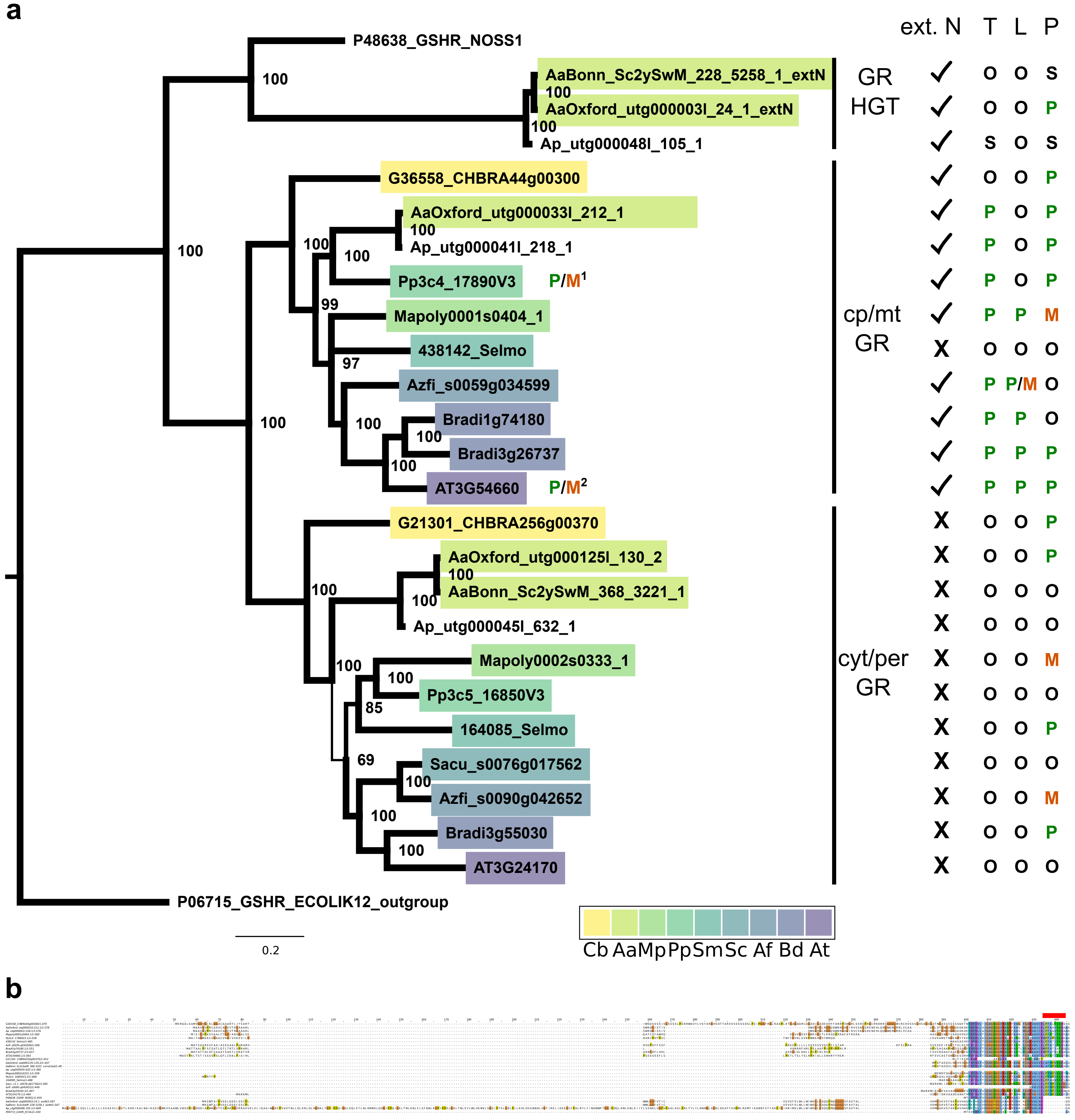
a
ext. N
T
L
P
P48638_GSHR_NOSS1
100
AaBonn_Sc2ySwM_228_5258_1_extN
100
AaOxford_utg000003l_24_1_extN
100
Ap_utg000048l_105_1
GR
HGT
G36558_CHBRA44g00300
AaOxford_utg000033l_212_1
100
Ap_utg000041l_218_1
100
100
100
Pp3c4_17890V3
P/M1
99
Mapoly0001s0404_1
438142_Selmo
97
Azfi_s0059g034599
100
Bradi1g74180
100
Bradi3g26737
100
AT3G54660
P/M2
cp/mt
GR
G21301_CHBRA256g00370
AaOxford_utg000125l_130_2
100
100
AaBonn_Sc2ySwM_368_3221_1
100
Ap_utg000045l_632_1
100
Mapoly0002s0333_1
100
Pp3c5_16850V3
85
164085_Selmo
69
Sacu_s0076g017562
100
Azfi_s0090g042652
100
Bradi3g55030
100
AT3G24170
cyt/per
GR
P06715_GSHR_ECOLIK12_outgroup
0.2
Cb Aa Mp Pp Sm Sc Af Bd At
b
